# Expression profiles of circular RNAs in sheep skeletal muscle

**DOI:** 10.5713/ajas.17.0563

**Published:** 2018-04-11

**Authors:** Yang Cao, Shuang You, Yang Yao, Zhi-Jin Liu, Wureli Hazi, Cun-Yuan Li, Xiang-Yu Zhang, Xiao-Xu Hou, Jun-Chang Wei, Xiao-Yue Li, Da-Wei Wang, Chuang-Fu Chen, Yun-Feng Zhang, Wei Ni, Sheng-Wei Hu

**Affiliations:** 1 College of Life Sciences, Shihezi University, Shihezi, Xinjiang 832003, China; 2 College of Animal Science and Technology, Shihezi University, Shihezi, Xinjiang 832003, China

**Keywords:** Sheep, circRNAs, Skeletal Muscle, Expression Profiles

## Abstract

**Objective:**

Circular RNAs (circRNAs) are a newfound class of non-coding RNA in animals and plants. Recent studies have revealed that circRNAs play important roles in cell proliferation, differentiation, autophagy and apoptosis during development. However, there are few reports about muscle development-related circRNAs in livestock.

**Methods:**

RNA sequencing analysis was employed to identify and annotate circRNAs from longissimus dorsi of sheep. Reverse transcription followed by real-time quantitative (q) polymerase chain reaction (PCR) analysis verified the presence of these circRNAs. Targetscan7.0 and miRanda were used to analyse the interaction of circRNA-microRNA (miRNA). To investigate the function of circRNAs, an experiment was conducted to perform enrichment analysis hosting genes of circRNAs using gene ontology (GO) and Kyoto encyclopedia of genes and genomes (KEGG) pathways.

**Results:**

About 75.5 million sequences were obtained from RNA libraries of sheep skeletal muscle. These sequences were mapped to 729 genes in the sheep reference genome. We identified 886 circRNAs, including numerous circular intronic RNAs and exonic circRNAs. Reverse transcription PCR (RT-PCR) and DNA sequencing analysis confirmed the presence of several circRNAs. Real-Time RT-PCR analysis exhibited resistance of sheep circRNAs to RNase R digestion. We found that many circRNAs interacted with muscle-specific miRNAs involved in growth and development of muscle, especially circ776. The GO and KEGG enrichment analysis showed that hosting genes of circRNAs was involved in muscle cell development and signaling pathway.

**Conclusion:**

The study provides comprehensive expression profiles of circRNAs in sheep skeletal muscle. Our study offers a large number of circRNAs to facilitate a better understanding of their roles in muscle growth. Meanwhile, we suggested that circ776 could be analyzed in future study.

## INTRODUCTION

Circular RNAs (circRNAs) are novel members of the non-coding RNA family [1]. CircRNAs can be generated by the direct ligation of 5′ and 3′ ends of linear RNAs, called back-splicing [2]. Although circRNAs were reported more than 35 years ago [3], the back-splicing has been considered a rare event. High throughput sequencing lately reveals that circRNAs are abundantly and stablely expressed in animals and plants in a tissue-specific manner. CircRNAs often consist of exons (exonic circRNAs), but they can also arise from introns (intronic circRNAs or circular intronic RNAs) [4]. Recent studies found that circRNAs served as microRNAs (miRNAs) sponges, sequestering miRNAs by competing interactions with targeted mRNAs [5]. CDR1as is a circRNA derived from an antisense transcript of the CDR1 protein-coding gene. With at least 60 conserved sites for miR-7, CDR1as is thought to act as a sponge to titrate miR-7 from its other targets. In addition, some circRNAs were also reported to function as positive regulators of RNA Pol II transcription in the cell nucleus [4].

Now the vast majority of circRNAs have been identified in cells of human [6], mice [7], Malaria parasite [8], Archaea [9], and rice [10]. Recently, Abdelmohsen et al. identified 12,000 circRNAs from skeletal muscle of monkeys of a range of ages, suggesting that some of these circRNAs may influence muscle growth [11]. Moreover, some studies reported that many miRNAs were expressed in a muscle-specific manner such as miR-133, miR-206, and miR-1 [12]. Intriguingly, Cesana et al [13] reported that long non-coding RNA (LINCMD1) worked as sponges for miR-133, thus regulating growth and differentiation of muscle. Similarly, it is possible that muscle-specific miRNAs also were regulated by circRNAs as competing endogenous RNA (ceRNA).

Sheep are an important animal in agriculture because of their utility in meat production, but expression and function circRNAs in skeletal muscle of sheep is not clear. Here, we provided the expression profiles of circRNAs in adult sheep muscle tissue using Illumina HiSeq 4000 technology. The data will facilitate better understanding roles of circRNAs in development and growth of skeletal muscle.

## MATERIALS AND METHODS

### Animals

Kazakh sheep belongs to a meat type breed with relatively slow growth rate and low meat yield. Improving the meat characteristics of Kazakh sheep has become the focal point of stockbreeding. All experiments involving animals were performed under the protocol approved by the Animal Care Committee of Shihezi University (XJ-2015NS842). The longissimus dorsi were collected from three adult Kazakh sheep. All of the animals were raised under the same conditions of free access to water and food in natural lighting. All tissues were immediately frozen in liquid nitrogen until RNA isolation.

### Library construction and circRNA sequencing analysis

Total RNAs were isolated using TRIzol (Invitrogen, Carlsbad, CA, USA) from frozen tissues according to the manufacturer’s procedure. The total RNA quantity and purity was analyzed with a Bioanalyzer 2100 (Agilent, Palo Alto, CA, USA) and NanoDrop ND-1000 (NanoDrop, Wilmington, DE, USA) with RIN number >7.0. The equivalent numbers of RNAs from three sheep were pooled to construct a library. Approximately 5 μg of total RNAs was used to deplete ribosomal RNAs according to the manuscript of the Ribo-Zero rRNA Removal Kit (Illumina, San Diego, CA, USA). The remaining RNAs were treated with RNase R (Illumina, USA) to remove linear RNAs and to enrich circRNAs. After removing ribosomal and linear RNAs, the enriched circRNAs were fragmented into small pieces using an RNA fragmentation kit (Ambion, Austin, TX, USA). Then the cleaved RNA fragments were reverse-transcribed to create cDNA, which was next used to synthesise U-labeled second-stranded DNAs with E. coli DNA polymerase I, RNase H and dUTP (NEB, Ipswich, MA, USA). An A-base was then added to the blunt ends of each strand, preparing them for ligation to the indexed adapters. Each adapter contains a T-base overhang for ligating the adapter to the A-tailed fragmented DNA. Single-or dual-index adapters was ligated to the fragments, and size selection was performed with AMPureXP beads (Beckman Coulter, Danvers, MA, USA). After the heat-labile uracil-DNA glycosylase (UDG) enzyme (NEB, USA) treatment of the U-labeled second-stranded DNAs, the ligated products was amplified with polymerase chain reaction (PCR). The average insert size for the final cDNA library was 300 bp (±50 bp).

We performed the 150 bp paired-end sequencing (PE150) on an Illumina Hiseq 4000 at the (LC Bio, Hangzhou, China) following the vendor’s recommended protocol. Cutadapt (http://cutadapt.readthedocs.io/) was used to remove the reads that contained adaptor contamination, low quality bases and undetermined bases. Then sequence quality was verified using FastQC (http://www.bioinformatics.babraham.ac.uk/projects/fastqc/). The subsequent analyses were based on the high-quality clean reads.

CircRNAs analysis was performed following the steps in the pipeline (Supplementary Figure S1). As described previously [14,15], we used Bowtie2 (http://bowtie-bio.sourceforge.net/) and TopHat2 (http://ccb.jhu.edu/software/tophat/) to map reads to *Ovis aries* genome (Oar_v3.1.77) from Ensemble (ftp://ftp.ensembl.org/pub/release-77/fasta/ovis_aries/dna/). Remaining reads (unmapped reads) were still mapped to genome using TopHat-Fusion (http://ccb.jhu.edu/software/tophat/fusion_index.shtml). CIRCExplorer2 (http://circexplorer2.readthedocs.io/) was used to denovo assemble the mapped reads to circRNAs at first; Then, back splicing reads were identified in unmapped reads by TopHat-fusion and CIRCExplorer2. We used the default parameters for each Software while running the analysis. Putative circRNAs were required to have more than two independent junction-spanning reads and had to correspond to the Breathnach-Chambon rule (GU/AG rule).

### Target site prediction and enrichment analysis

Targetscan7.0 (http://www.targetscan.org/) and miRanda (http://www.microrna.org/microrna/home.do) were used for analysing the interaction of circRNA-miRNA [16]. Hosting genes of circRNAs were used for listing gene names that were input into DAVID software (https://david.ncifcrf.gov/) for gene ontology (GO) analysis [17]. A Kyoto encyclopedia of genes and genomes (KEGG) enrichment analysis of hosting genes of circRNA was performed with KOBAS software (http://kobas.cbi.pku.edu.cn/) [18]. Score (p<0.05) was considered significant for enrichment analysis.

### Reverse transcription polymerase chain reaction analysis and DNA sequencing

Total RNAs were extracted from longissimus dorsi of sheep using TRIzol (Invitrogen, USA). From purified RNA, cDNA was synthesized using RT-PCR Kit (Takara, Dalian, China). To confirm the data on circRNAs was generated from RNA-seq analysis, the candidates validated in the following were selected randomly. These sequences, obtained from sequencing, were disconnected in back-splice site. Firstly, we moved a region (about 50 to 100 bp) from the end to the start of sequence. The new connection is the back-splice site. Next, the divergent primers were designed as usual, but the PCR product must include the circular junctions. PCR was carried out using these primers for circ121 (F: AAG CTC GGT CAC TTT GGA and R: CTT TGG GCT CAG GAA TCA), circ217 (F: CCA TCA ATG GAG TGA CTG AG and R: CAC AAG GGA GCC AAG ATT AT), circ252 (F: CAT TGT CAT GCA AGC TGTC and R: CTC TTT GTG CTT CCA GGTA), circ276 (F: TGG CTC CAG TAA CTC CATC and R: ATC ATC TCC ACC CCG AAA GT), circ443 (F: GTG GTC AAT AAT GCT GAT GA and R: TTC CCA GTT CCA GTC TGTC), circ465 (F: CTG GAA AAT TGC TCT TCTC and R: CCT TCT AGC TTT AGA CCT TT), circ543 (F: GAC GGA ACT TCA TCA ACAA and R: TTC CCA GTC ATC TTC AGC), circ742 (F: AGC AGG AGA TGG AGG AGG AC and R: GGT GAC CTG CCT TCT GGA CT), and circ783 (F: TAT CTG CCC TCC CAG TCC C and R: TGT GGC TGA GAA TGA GGG TTAC). PCR products were analyzed by gel electrophoresis. For DNA sequencing, PCR products were gel purified and subjected to TA cloning.

### Real-time RT-PCR analysis

The expression levels of six circRNAs (circRNA121, circRNA217, circRNA276, circRNA443, circRNA465, and circRNA783) were detected by real-time RT-PCR analysis. Total RNAs were treated with RNase R (RNR-07250, epicentre) following the manufacturer’s protocol. cDNA was synthesized from RNase R-treated RNAs and un-treated total RNAs with RT-PCR Kit (Takara, China). The primers used for the RT-PCR are listed above. Real-time PCR was performed using SYBR Green (TaKaRa Biotech, Dalian, China) according to the manufacturer’s protocol. The levels of circRNA digested by RNase R were normalized to levels of circRNA not digested. Meanwhile, linear mRNA of glyceraldehyde-3-phosphate dehydrogenase (GAPDH) (sensitive to RNase R) is a positive control [19]. Three independent experiments were performed on triplicate samples.

## RESULTS

### Deep sequencing of circRNAs in sheep skeletal muscle

**Figure 1:**
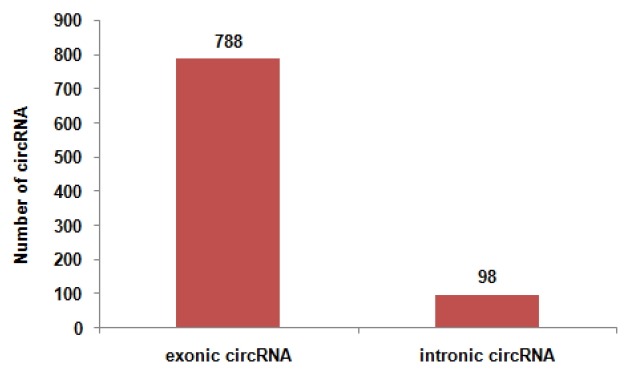
Exonic and intronic circular RNAs (circRNAs) in 886 circRNAs identified. We totally identified 886 circRNAs.778 circRNAs originate from exonic sequences (exonic circRNA). Meanwhile, 98 circRNAs originate from intronic sequences (intronic circRNA or ciRNA).

**Figure 2:**
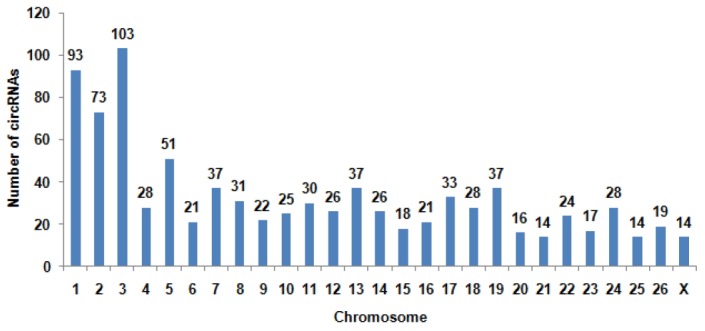
The distribution of circular RNAs (circRNAs) in sheep chromosomes. The total circRNAs originate from 27 chromosomes of sheep. The number of circRNAs in chromosome 3 is the largest of all chromosomes.

**Figure 3:**
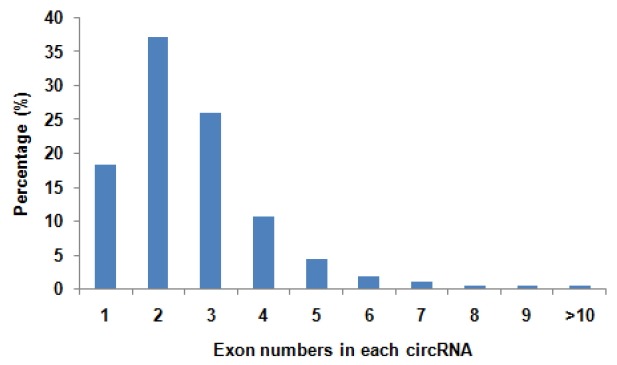
Number of exons per transcript of sheep circular RNAs (circRNAs). There are some difference in exon numbers of circRNAs. A main range of exon numbers is one to four. While, some circRNAs accounted more than ten exons.

To determine the identity and abundance of circRNAs in sheep skeletal muscle, deep sequencing approach was used for analyzing circRNAs. A total of 75.5 million paired-end reads were obtained with accuracy 57.31% that mapped to the sheep reference genome. We identified a total of 886 circRNAs from RNA-seq data in skeletal muscle, mapping in 729 host-genes. The majority of the circRNAs (88.94%) originate from exonic sequences (exonic circRNA), whereas a small part included intronic sequences (intronic circRNA or ciRNA) (Figure 1). These circRNAs were located in 26 autosomes and X chromosome (Figure 2). Most host-genes (626/729) expressed only one circRNA, meanwhile circRNAs with two and three exons accounted for 62.92% of all circRNAs (Figure 3). Full list of the circRNAs are provided in Supplementary Additional file 1, including circRNAs annotation, chromosomal location, hosting mRNA and so on.

### Validation of sheep circRNAs

**Figure 4:**
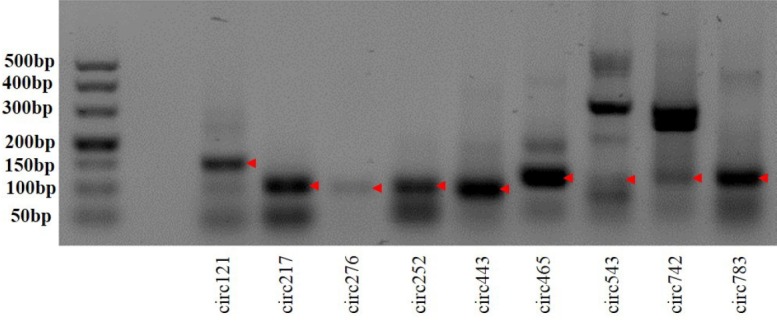
Reverse transcription polymerase chain reaction (RT-PCR) amplification of circular RNAs with divergent primers. The red arrow denotes RT-PCR products.

**Figure 5:**
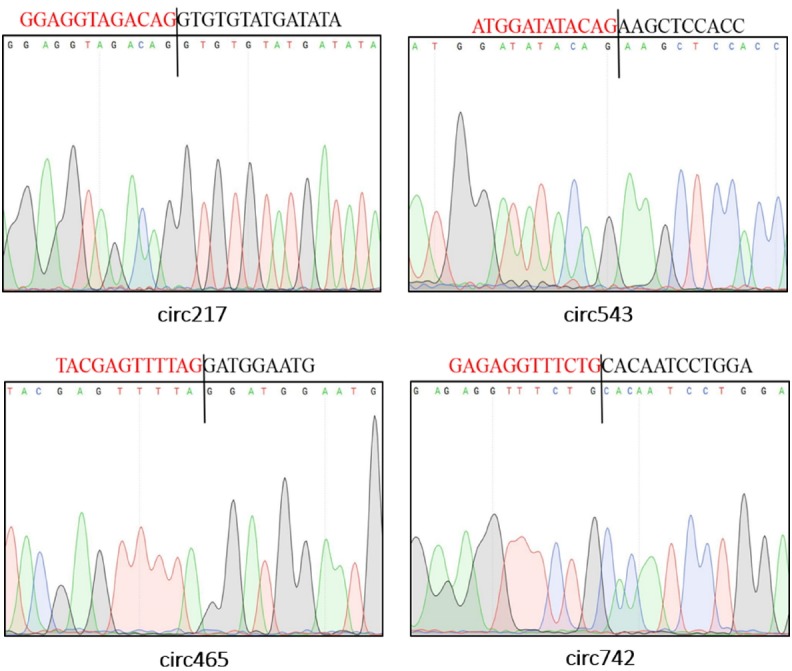
Representative sequencing results of head-to-tail junctions sequences from reverse transcription polymerase chain reaction (RT-PCR) products. The black line locates the back-splice site. And the region marked by red (or black) color is the end (or start) sequence of circular RNAs (broken in junction).

**Figure 6:**
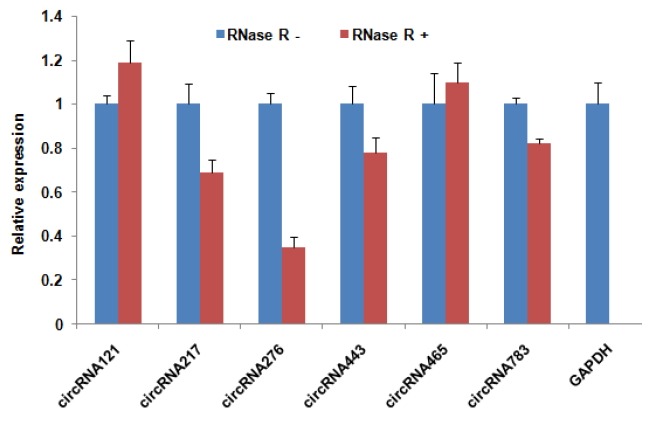
Validation of circular RNAs (circRNA) expression in sheep skeletal muscle by real-time reverse transcription polymerase chain reaction (RT-PCR). Expression of circRNA was analysed in both RNase R (−) and RNase R (+) treated RNA samples. Linear mRNA of glyceraldehyde-3-phosphate dehydrogenase (GAPDH) was used as RNase-sensitive control. Digested RNA was normalized to undigested RNA. Data are the mean±standard error of the mean.

To validate the sequencing data, we further experimentally detected expression of sheep circRNAs. Divergent primers were designed to amplify the circular junctions (Supplementary Figure S1). Nine candidates were successfully validated by RT-PCR (Figure 4). Some results had nonspecific products, which could be some isoforms from alternative splicing circularization. Head-to-tail junctions were confirmed by DNA sequencing (Figure 5). We also tested resistance of circRNAs to RNase R digestion by real-time RT-PCR. All tested circRNAs were resistant to RNase R digestion, whereas the linear mRNA of GAPDH was not detected (sensitive to RNase R) (Figure 6).

### Predicted functions of sheep circRNAs

**Figure 7:**
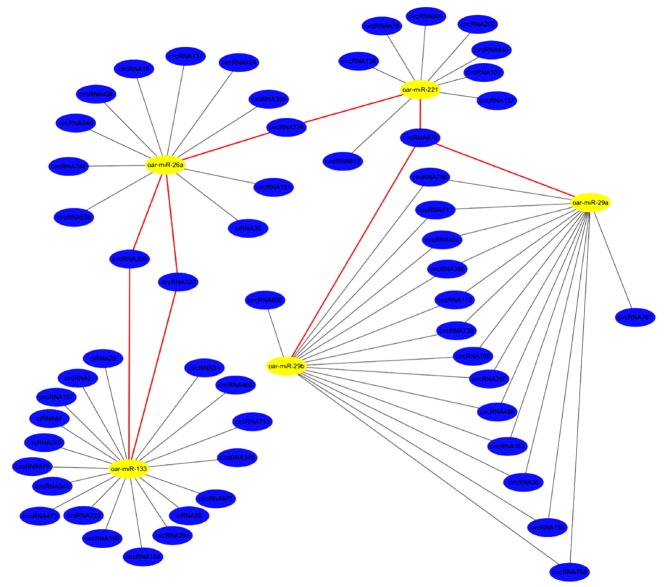
Regulatory networks of circular RNAs (circRNAs)-microRNAs (miRNAs). The yellow miRNAs were related with growth and development of muscle. These blue circRNAs had miRNA target sites.

We used Targetscan7.0 and miRanda to analyse the interaction of circRNA-miRNA. A total of 6,194 interaction relationships between 877 circRNAs and various miRNAs were identified (Supplementary Additional file 2). Interestingly, we found that some circRNAs (circRNA100, circRNA205, circRNA606, circRNA744, circRNA776, circRNA108, and circRNA678) contained at least two conserved target sites for muscle development-related miRNAs (miR-133, miR-208, miR-499, and miR-29b, respectively), thereby suggesting that growth and development of muscle may be regulated by circRNA. Five representative miRNAs are shown in Figure 7.

To investigate the function of circRNAs, we performed enrichment analysis for hosting genes of circRNAs using GO and KEGG pathways. The GO analysis showed that hosting genes of circRNAs played an important role in growth and development of muscle, including muscle cell development (GO: 0055001), positive regulation of myoblast differentiation (GO: 0045663), muscle fiber development (GO: 0048747), and muscle organ development (GO: 0007517). KEGG pathway analysis showed that hosting genes of circRNAs were related to muscle development pathway, including transforming growth factor-β signaling pathway (04350), mammalian target of rapamycin signaling pathway (04150), Wnt signaling pathway (04310), MAPK signaling pathway (04010). These full results are shown in Supplementary Additional file 1.

## DISCUSSION

CircRNAs with closed-loop structure are resistant to exonuclease RNase R and more stable than liner RNA [20]. Many circRNA have been found in HEK293 cells, leukocytes, fibroblasts of human and mouse [21,22], but little is known about the circRNAs in the sheep genome. Here, we identified that hundreds of sheep genes can express circRNAs in skeletal muscle. We identified 886 circRNAs by high-throughput sequencing and bioinformatics analysis. Some selected circRNAs were experimentally validated by divergent primer amplification and their resistance to RNase R digestion.

Previous studies showed that some host-genes of circRNAs in Guizhou miniature pig (*Sus scrofa*) potentially associated with muscle growth [23]. Sun et al [24] drew a conclusion that some host-genes of circRNAs related to myogenesis in the longissimus dorsi of Landrace and Lantang pigs. Our study also found that some host-genes, participating in the growth and development of muscle, could express circRNAs. Myocyte Enhancer Factor 2C (MEF2C), playing a significant role during muscle growth [25], can express circRNA468 as shown in our study. MEF2C knockouts in mice resulted in defects in muscle fusion [26], suggesting that circRNA468 may be involved in muscle growth. Myocyte enhancer factor 2A (MEF2A) can express circRNA235. Previous studies had demonstrated that MEF2A could influence muscle satellite cells differentiation [26–28]. Insulin-like growth factor-binding protein 5 (expressing circRNA643) contributes greatly to muscle cell development [29–31], affects muscle regeneration [32]. Akt3 (expressing circRNA53) affects the insulin-like growth factor-I-mediated signaling and survival in myoblasts [33,34]. Insulin-like growth factor 2 receptor (expressing circRNA786) also plays an important role in growth and development of muscle [35]. However, whether expression of the host-coding gene can impact circRNA expression will be analysed in the future study.

Some reports concluded that circRNA, same as ceRNA, could effect post-transcriptional regulation by targeting miRNAs [36,37]. Liang et al [23] found that circRNAs harbour well-conserved canonical miRNA seed matches, suggesting that some circRNAs act as miRNAs sponges. Through Targetscan7.0 and miRanda, some miRNA target sites were found in circRNAs. We found that circRNAs interacted with many miRNAs related to the growth and development of muscle, such as miR-208, miR-133, miR-206. Additionally, some circRNAs include simultaneously two target sites of different miRNAs. CircRNA30 has sites of miRNA-29 and miRNA-26 simultaneously. Similarly, CircRNA100 (interacting with miRNA-26 and miRNA-133), CircRNA678 (interacting with miRNA-29 and miRNA-221), circRNA205 (interacting with miRNA-133 and miRNA-26), and CircRNA606 (interacting with miRNA-27 and miRNA-29) also have sites of miRNAs. Meanwhile, these miRNAs play an important role in growth and development of muscle. Previous studies had confirmed that miRNA-29 could target Akt3 to reduce proliferation and facilitate differentiation of myoblasts in skeletal muscle development [38], and miR-26 inhibits expression of Ezh2, a known suppressor of skeletal muscle cell differentiation [39]. To our knowledge, overexpression of miR-27a in chronic kidney disease mice attenuated muscle loss, improved grip strength [40], and miR-221 increased skeletal muscle satellite cells proliferation and decreased differentiation [41]. miR-133 involved in the formation of myotubes and myoblast proliferation [42,43]. Therefore, these circRNAs, as potential ceRNA, may be a key regulator for growth and development skeletal muscle, which will be analyzed in future study.

In addition, a recent report from Legnini’s group has performed expression profiling of circRNAs during *in vitro* differentiation of murine and human myoblasts, and identified conserved species regulated in myogenesis and altered in Duchenne muscular dystrophy [44]. In studies performed by Wei et al [45], it was shown that they examined circRNA expression profiles of two developmental stages of bovine skeletal muscle (embryonic and adult musculus longissimus) to provide first insights into their potential involvement in bovine myogenesis. circRNAs also were examined in the heart from rats, mice and humans [46]. These studies also offer a novel sight which could help in the understanding of the mechanisms that underlie the function of circRNAs in organism.

## CONCLUSION

We identified a number of circRNAs in sheep skeletal muscle and validated the sequencing data by RT-PCR. Meanwhile, we found that several circRNAs take part in the pathway of development and growth of muscle by KEGG pathway analysis. Function prediction showed that circRNAs play an important role in the process of muscle growth. Our study provides a valuable resource for circRNAs biology, as well as contributes to understanding circRNAs function in growth and development of muscles.

## Supplementary Data
