# Supplementary material for: Expression profiles of circular RNAs in sheep skeletal muscle
Source: Asian-Australas J Anim Sci. 2018 Apr 11;31(10):1550–7. doi: 10.5713/ajas.17.0563 (PMC6127590; doi:10.5713/ajas.17.0563)
Supplement: Supplementary file 1 [file ajas-31-10-1550-supplementary.pdf]

Additional file 1

Accession	muscle_count	muscle_fpkm	chr	start	end	strand	exon Count	circType	circRNA category	isoformName	geneName	hosting gene type
ciRNA1	4	3.54E+02	chrX	48270847	48271032	-	1	ciRNA	-	ENSOART00000006764	ENSOARG00000006221	mRNA
ciRNA10	40	2.95E+03	chr12	39822990	39823212	-	1	ciRNA	-	ENSOART00000019330	MFN2	mRNA
ciRNA11	4	2.70E+02	chr12	47543856	47544099	+	1	ciRNA	-	ENSOART00000017537	WRAP73	mRNA
ciRNA12	7	4.88E+02	chr12	78997025	78997260	+	1	ciRNA	-	ENSOART00000000364	RNPEP	mRNA
ciRNA13	359	3.25E+04	chr11	27344371	27344552	-	1	ciRNA	-	ENSOART00000020594	MPER1	mRNA
ciRNA14	235	2.27E+04	chr11	27344383	27344552	-	1	ciRNA	-	ENSOART00000020594	MPER1	mRNA
ciRNA15	23.5	1.17E+03	chr11	42169437	42169766	+	1	ciRNA	-	ENSOART00000002909	ENSOARG00000002687	mRNA
ciRNA16	23.5	1.17E+03	chr11	42214582	42214911	+	1	ciRNA	-	ENSOART00000002994	TUBG2	mRNA
ciRNA17	5	5.45E+02	chr11	52919964	52920114	-	1	ciRNA	-	ENSOART00000005882	ENSOARG00000005400	mRNA
ciRNA18	2	2.17E+02	chr11	55658181	55658332	-	1	ciRNA	-	ENSOART00000013388	RAB37	mRNA
ciRNA19	3	2.36E+02	chr10	35877868	35878076	+	1	ciRNA	-	ENSOART00000014938	LATS2	mRNA
ciRNA2	6	2.67E+02	chrX	75296057	75296426	-	1	ciRNA	-	ENSOART00000003246	GAB3	mRNA
ciRNA20	11	3.19E+02	chr17	3874545	3875111	-	1	ciRNA	-	ENSOART00000002585	KIAA0922	mRNA
ciRNA21	3	1.47E+02	chr17	12857187	12857522	+	1	ciRNA	-	ENSOART00000010126	OTUD4	mRNA
ciRNA22	4	3.54E+02	chr17	18024026	18024211	-	1	ciRNA	-	ENSOART00000014901	NAA15	mRNA
ciRNA23	3	2.12E+02	chr17	71458697	71458929	-	1	ciRNA	-	ENSOART00000017596	PI4KA	mRNA
ciRNA24	2	2.18E+02	chr16	19315535	19315685	+	1	ciRNA	-	ENSOART00000007829	ENSOARG00000007198	mRNA
ciRNA25	7	7.43E+02	chr16	62669818	62669972	-	1	ciRNA	-	ENSOART00000015334	MARCH6	mRNA
ciRNA26	7	7.15E+02	chr15	45861046	45861206	+	1	ciRNA	-	ENSOART00000018703	TAF10	mRNA
ciRNA27	25	2.04E+03	chr14	14002172	14002373	+	1	ciRNA	-	ENSOART00000015277	SPG7	mRNA
ciRNA28	7	4.45E+02	chr14	14080008	14080266	-	1	ciRNA	-	ENSOART00000015539	CHMP1A	mRNA
ciRNA29	1	8.53E+01	chr14	14531172	14531364	+	1	ciRNA	-	ENSOART00000017024	ORC6	mRNA
ciRNA3	6	5.43E+02	chrX	77136670	77136851	+	1	ciRNA	-	ENSOART00000004636	UBL4A	mRNA
ciRNA30	2	8.44E+01	chr19	10793501	10793890	+	1	ciRNA	-	ENSOART00000000320	GOLGA4	mRNA
ciRNA31	90.5	7.64E+03	chr19	48938686	48938880	+	1	ciRNA	-	ENSOART00000007176	ENSOARG00000006603	mRNA
ciRNA32	91.5	7.53E+03	chr19	48938686	48938885	+	1	ciRNA	-	ENSOART00000007176	ENSOARG00000006603	mRNA
ciRNA33	3	3.09E+02	chr19	56030215	56030374	+	1	ciRNA	-	ENSOART00000014844	PLXND1	mRNA
ciRNA34	3	3.27E+02	chr19	58938907	58939057	+	1	ciRNA	-	ENSOART00000004079	COPG1	mRNA
ciRNA35	4	1.57E+02	chr18	25775541	25775960	+	1	ciRNA	-	ENSOART00000016904	CEMIP	mRNA
ciRNA36	17	1.01E+03	chr26	35067684	35067959	-	1	ciRNA	-	ENSOART00000002940	ANK1	mRNA
ciRNA37	22	6.66E+02	chr24	317915	318458	+	1	ciRNA	-	ENSOART00000013041	DECR2	mRNA
ciRNA38	51	3.96E+03	chr24	2417881	2418092	-	1	ciRNA	-	ENSOART00000000924	HCFC1R1	mRNA
ciRNA39	86	8.13E+03	chr24	25935695	25935868	-	1	ciRNA	-	ENSOART00000002308	ATP2A1	mRNA
ciRNA4	2	2.15E+02	chr13	3166034	3166186	-	1	ciRNA	-	ENSOART00000011225	MKKS	mRNA
ciRNA40	86	8.18E+03	chr24	25935696	25935868	-	1	ciRNA	-	ENSOART00000002308	ATP2A1	mRNA
ciRNA41	7	5.51E+02	chr24	25939102	25939310	-	1	ciRNA	-	ENSOART00000002308	ATP2A1	mRNA
ciRNA42	1	1.05E+02	chr24	25956496	25956652	-	1	ciRNA	-	ENSOART00000002489	SH2B1	mRNA
ciRNA43	1	9.14E+01	chr22	32978556	32978735	+	1	ciRNA	-	ENSOART00000013653	HABP2	mRNA
ciRNA44	4	2.27E+02	chr23	37602735	37603024	-	1	ciRNA	-	ENSOART00000011131	MYOM1	mRNA
ciRNA45	4	3.12E+02	chr23	41996778	41996988	+	1	ciRNA	-	ENSOART00000001156	RALBP1	mRNA
ciRNA46	3	2.01E+02	chr20	15159810	15160055	-	1	ciRNA	-	ENSOART00000001720	TREML1	mRNA
ciRNA47	2	1.56E+02	chr20	19936362	19936572	+	1	ciRNA	-	ENSOART00000011754	SLC25A27	mRNA
ciRNA48	10	3.29E+02	chr20	26411431	26411930	+	1	ciRNA	-	ENSOART00000001354	TNXB	mRNA
ciRNA49	6	4.49E+02	chr21	17502676	17502895	+	1	ciRNA	-	ENSOART00000007331	INTS4	mRNA
ciRNA5	6	3.05E+02	chr13	21243511	21243834	-	1	ciRNA	-	ENSOART00000000489	NEBL	mRNA
ciRNA50	36	3.22E+03	chr21	41550297	41550480	+	1	ciRNA	-	ENSOART00000003775	MARK2	mRNA
ciRNA51	10	6.35E+02	chr21	43628750	43629008	+	1	ciRNA	-	ENSOART00000003825	RAB1B	mRNA
ciRNA52	8	3.99E+02	chr21	44606059	44606388	+	1	ciRNA	-	ENSOART00000012801	RPS6KB2	mRNA
ciRNA53	6	3.06E+02	chr7	10786991	10787313	+	1	ciRNA	-	ENSOART00000019046	THBS4	mRNA
ciRNA54	22	1.53E+03	chr7	21110571	21110807	+	1	ciRNA	-	ENSOART00000021039	OMYHCS	mRNA
ciRNA55	14	1.16E+03	chr7	23233761	23233958	-	1	ciRNA	-	ENSOART00000021440	ARHGEF40	mRNA
ciRNA56	15	1.07E+03	chr7	34580709	34580938	-	1	ciRNA	-	ENSOART00000022317	VPS39	mRNA
ciRNA57	3	2.76E+02	chr7	59630366	59630544	-	1	ciRNA	-	ENSOART00000022914	SEMA6D	mRNA
ciRNA58	2.333333333	1.22E+02	chr6	17341402	17341715	-	1	ciRNA	-	ENSOART00000008964	HADH	mRNA
ciRNA59	5	3.61E+02	chr5	2425064	2425291	-	1	ciRNA	-	ENSOART00000005964	ENSOARG00000005438	mRNA
ciRNA6	5	5.11E+02	chr13	56391133	56391293	-	1	ciRNA	-	ENSOART00000017313	EDN3	mRNA
ciRNA60	18	8.09E+02	chr5	2446879	2447244	-	1	ciRNA	-	ENSOART00000005964	ENSOARG00000005438	mRNA
ciRNA61	3	2.61E+02	chr5	5167728	5167916	-	1	ciRNA	-	ENSOART00000016919	MAP1S	mRNA
ciRNA62	6	2.77E+02	chr5	14069739	14070095	+	1	ciRNA	-	ENSOART00000001168	MCOLN1	mRNA
ciRNA63	14	1.23E+03	chr5	17403995	17404181	-	1	ciRNA	-	ENSOART00000011440	ANKRD24	mRNA
ciRNA64	7	7.02E+02	chr5	40632054	40632217	-	1	ciRNA	-	ENSOART00000009694	POLRMT	mRNA
ciRNA65	8	6.07E+02	chr5	46998365	46998581	+	1	ciRNA	-	ENSOART00000017285	KIF20A	mRNA
ciRNA66	6	5.28E+02	chr4	7264160	7264346	-	1	ciRNA	-	ENSOART00000017002	ABCA13	mRNA
ciRNA67	6	5.37E+02	chr4	92927413	92927596	-	1	ciRNA	-	ENSOART00000003947	KCP	mRNA
ciRNA68	5	3.76E+02	chr4	113210500	113210718	-	1	ciRNA	-	ENSOART00000004258	SMARCD3	mRNA
ciRNA69	5	3.34E+02	chr4	118845915	118846160	-	1	ciRNA	-	ENSOART00000010589	ESYT2	mRNA
ciRNA7	4	4.33E+02	chr13	61858313	61858464	+	1	ciRNA	-	ENSOART00000006891	ENSOARG00000006335	mRNA
ciRNA70	7	5.65E+02	chr3	7322320	7322523	-	1	ciRNA	-	ENSOART00000009620	DOLPP1	mRNA
ciRNA71	16	5.96E+02	chr3	34019651	34020092	+	1	ciRNA	-	ENSOART00000020760	AGBL5	mRNA
ciRNA72	4	2.51E+02	chr3	34306177	34306438	+	1	ciRNA	-	ENSOART00000021004	SNX17	mRNA
ciRNA73	8	6.75E+02	chr3	68319190	68319384	+	1	ciRNA	-	ENSOART00000002344	PNPT1	mRNA
ciRNA74	5	1.38E+02	chr3	132067833	132068429	-	1	ciRNA	-	ENSOART00000017660	COPZ1	mRNA
ciRNA75	3	1.18E+02	chr3	147041637	147042055	+	1	ciRNA	-	ENSOART00000021772	SLC2A13	mRNA
ciRNA76	18	1.52E+03	chr3	163489520	163489714	+	1	ciRNA	-	ENSOART00000012425	ITGA7	mRNA
ciRNA77	1	9.80E+01	chr3	174853347	174853514	+	1	ciRNA	-	ENSOART00000019009	POLR3B	mRNA
ciRNA78	5	5.38E+02	chr3	205762491	205762643	-	1	ciRNA	-	ENSOART00000001639	PHC1	mRNA
ciRNA79	35	2.41E+03	chr3	207729826	207730064	+	1	ciRNA	-	ENSOART00000007750	ING4	mRNA
ciRNA8	2.5	1.37E+02	chr13	65545791	65546091	-	1	ciRNA	-	ENSOART00000018308	SAMHD1	mRNA
ciRNA80	3	2.65E+02	chr3	223455207	223455392	-	1	ciRNA	-	ENSOART00000021350	BRD1	mRNA
ciRNA81	12	4.25E+02	chr2	130457973	130458437	+	1	ciRNA	-	ENSOART00000018808	TTN	mRNA
ciRNA82	2	1.37E+02	chr2	135807669	135807909	-	1	ciRNA	-	ENSOART00000000922	RAPGEF4	mRNA
ciRNA83	8	5.72E+02	chr2	154154696	154154925	-	1	ciRNA	-	ENSOART00000008723	GALNT13	mRNA
ciRNA84	6	6.45E+02	chr2	220534409	220534561	+	1	ciRNA	-	ENSOART00000022052	SLC4A3	mRNA
ciRNA85	35.33333333	2.57E+03	chr1	20376961	20377186	+	1	ciRNA	-	ENSOART00000002245	MAST2	mRNA
ciRNA86	35.33333333	2.55E+03	chr1	20376961	20377188	+	1	ciRNA	-	ENSOART00000002245	MAST2	mRNA
ciRNA87	35.33333333	2.48E+03	chr1	20376961	20377194	+	1	ciRNA	-	ENSOART00000002245	MAST2	mRNA
ciRNA88	8	6.68E+02	chr1	20382493	20382689	+	1	ciRNA	-	ENSOART00000002245	MAST2	mRNA
ciRNA89	48	4.44E+03	chr1	20383624	20383801	+	1	ciRNA	-	ENSOART00000002245	MAST2	mRNA
ciRNA9	3	1.73E+02	chr12	21634845	21635129	-	1	ciRNA	-	ENSOART00000012871	EPRS	mRNA
ciRNA90	48	4.39E+03	chr1	20383624	20383803	+	1	ciRNA	-	ENSOART00000002245	MAST2	mRNA
ciRNA91	1	1.09E+02	chr1	65576335	65576485	-	1	ciRNA	-	ENSOART00000016384	CCBL2	mRNA
ciRNA92	7	5.62E+02	chr1	102902952	102903156	-	1	ciRNA	-	ENSOART00000001735	ENSOARG00000001610	mRNA
ciRNA93	3	2.54E+02	chr1	144679913	144680106	-	1	ciRNA	-	ENSOART00000018235	ROBO1	mRNA
ciRNA94	3	2.85E+02	chr1	182827634	182827806	+	1	ciRNA	-	ENSOART00000021549	MAATS1	mRNA
ciRNA95	4	3.83E+02	chr1	183379854	183380025	-	1	ciRNA	-	ENSOART00000021643	LRRC58	mRNA
ciRNA96	6	6.25E+02	chr9	14089544	14089701	-	1	ciRNA	-	ENSOART00000000619	GSDMD	mRNA

ciRNA97	1	8.57E+01	chr8	56468453	56468644	+	1	ciRNA	-	ENSOART00000015146	AKAP7	mRNA
ciRNA98	6	4.11E+02	chr8	56759651	56759890	-	1	ciRNA	-	ENSOART00000015253	MED23	mRNA
circRNA1	10	6.05E+02	chrX	1306179	1307128	-	2	circRNA	-	ENSOART00000009098	ARSD	mRNA
circRNA10	10	4.83E+02	chrX	115746980	115748116	+	2	circRNA	-	ENSOART00000019011	AMOT	mRNA
circRNA100	20	7.67E+02	chr10	26588680	26592574	-	3	circRNA	-	ENSOART00000011571	NBEA	mRNA
circRNA101	4	1.19E+02	chr10	28577657	28578297	-	2	circRNA	-	ENSOART00000011883	PDS5B	mRNA
circRNA102	13	6.81E+02	chr10	28988124	28990004	-	2	circRNA	-	ENSOART00000012432	FRY	mRNA
circRNA103	147	2.29E+03	chr10	28998514	29010704	-	7	circRNA	-	ENSOART00000012432	FRY	mRNA
circRNA104	14	8.56E+02	chr10	29028873	29029476	-	2	circRNA	-	ENSOART00000012432	FRY	mRNA
circRNA105	22	8.96E+02	chr10	29059910	29060791	-	2	circRNA	-	ENSOART00000012432	FRY	mRNA
circRNA106	60	2.41E+03	chr10	33160170	33166307	-	3	circRNA	-	ENSOART00000013804	WASF3	mRNA
circRNA107	96	4.82E+03	chr10	33348342	33362755	-	3	circRNA	-	ENSOART00000013843	CDK8	mRNA
circRNA108	94	2.68E+03	chr10	34879964	34891970	-	5	circRNA	-	ENSOART00000014609	SGCG	mRNA
circRNA109	42	2.01E+03	chr10	51289502	51294905	+	3	circRNA	-	ENSOART00000017226	LMO7	mRNA
circRNA11	11	5.76E+02	chrX	126686132	126686773	-	2	circRNA	-	ENSOART00000002454	SRPX2	mRNA
circRNA110	62	1.80E+03	chr10	54265130	54273188	-	4	circRNA	-	ENSOART00000000207	RNF219	mRNA
circRNA111	181	4.38E+03	chr10	83392025	83413889	+	6	circRNA	-	ENSOART00000006309	MYO16	mRNA
circRNA112	112	4.57E+03	chr10	84831774	84849824	+	4	circRNA	-	ENSOART00000007735	ARHGEF7	mRNA
circRNA113	52	3.06E+03	chr10	84831774	84841892	+	3	circRNA	-	ENSOART00000007735	ARHGEF7	mRNA
circRNA114	350	1.47E+04	chr17	251790	302748	+	3	circRNA	-	ENSOART00000017426	KLHL2	mRNA
circRNA115	279.5	9.43E+03	chr17	9946520	9975542	-	5	circRNA	-	ENSOART00000007922	ARHGAP10	mRNA
circRNA116	341.5	1.42E+04	chr17	10000921	10041116	-	5	circRNA	-	ENSOART00000007922	ARHGAP10	mRNA
circRNA117	5	5.21E+02	chr17	10112050	10112280	-	2	circRNA	-	ENSOART00000007922	ARHGAP10	mRNA
circRNA118	32	1.79E+03	chr17	12215857	12218594	+	2	circRNA	-	ENSOART00000009337	ZNF827	mRNA
circRNA119	3	1.71E+02	chr17	12868842	12869231	+	2	circRNA	-	ENSOART00000010126	OTUD4	mRNA
circRNA12	32	1.62E+03	chr13	9843122	9846344	-	3	circRNA	-	ENSOART00000013013	KIF16B	mRNA
circRNA120	20	9.65E+02	chr17	12897608	12899192	-	3	circRNA	-	ENSOART00000010578	ABCE1	mRNA
circRNA121	633	1.46E+04	chr17	16494898	16554002	+	5	circRNA	-	ENSOART00000013306	RNF150	mRNA
circRNA122	176.5	5.56E+03	chr17	17840469	17860245	+	3	circRNA	-	ENSOART00000014199	SETD7	mRNA
circRNA123	24.5	1.03E+03	chr17	17855204	17860245	+	2	circRNA	-	ENSOART00000014199	SETD7	mRNA
circRNA124	157	5.24E+03	chr17	18156331	18175228	+	3	circRNA	-	ENSOART00000015281	ELF2	mRNA
circRNA125	74	3.93E+03	chr17	39447900	39457401	-	2	circRNA	-	ENSOART00000002506	RAPGEF2	mRNA
circRNA126	19	1.28E+03	chr17	39523185	39525287	-	2	circRNA	-	ENSOART00000002506	RAPGEF2	mRNA
circRNA127	17	1.30E+03	chr17	43690210	43692203	+	2	circRNA	-	ENSOART00000009219	ZNF84	mRNA
circRNA128	118	4.95E+03	chr17	50594843	50606607	+	4	circRNA	-	ENSOART00000020025	SCARB1	mRNA
circRNA129	58	2.80E+03	chr17	51721651	51722653	+	2	circRNA	-	ENSOART00000003868	RILPL1	mRNA
circRNA13	279	1.35E+04	chr13	11504060	11529530	-	3	circRNA	-	ENSOART00000014628	SFMBT2	mRNA
circRNA130	40	2.04E+03	chr17	51941611	51947424	+	3	circRNA	-	ENSOART00000006043	MPHOSPH9	mRNA
circRNA131	24	1.17E+03	chr17	52116136	52117521	+	2	circRNA	-	ENSOART00000006847	PITPNM2	mRNA
circRNA132	19	1.89E+03	chr17	52641256	52643362	+	2	circRNA	-	ENSOART00000012144	CLIP1	mRNA
circRNA133	33	1.90E+03	chr17	56436266	56438821	-	4	circRNA	-	ENSOART00000002549	SUDS3	mRNA
circRNA134	26	1.68E+03	chr17	57831026	57834416	-	2	circRNA	-	ENSOART00000005890	RNFT2	mRNA
circRNA135	68	3.89E+03	chr17	63033632	63038809	+	2	circRNA	-	ENSOART00000015729	GLTP	mRNA
circRNA136	24	1.00E+03	chr17	63528091	63529089	-	2	circRNA	-	ENSOART00000017904	ACACB	mRNA
circRNA137	24	8.49E+02	chr17	63540204	63543573	-	4	circRNA	-	ENSOART00000017904	ACACB	mRNA
circRNA138	10	6.40E+02	chr17	63541644	63543573	-	2	circRNA	-	ENSOART00000017904	ACACB	mRNA
circRNA139	6	6.54E+02	chr17	63563879	63564029	-	1	circRNA	-	ENSOART00000017904	ACACB	mRNA
circRNA14	99	3.35E+03	chr13	11589923	11599244	-	3	circRNA	-	ENSOART00000014628	SFMBT2	mRNA
circRNA140	10	7.19E+02	chr17	65333292	65333794	+	2	circRNA	-	ENSOART00000020825	MYO18B	mRNA
circRNA141	60	3.37E+03	chr17	68582123	68590897	+	3	circRNA	-	ENSOART00000006418	MTMR3	mRNA
circRNA142	76	5.37E+03	chr17	72039847	72041111	-	2	circRNA	-	ENSOART00000019462	TXNRD2	mRNA
circRNA143	59	4.88E+03	chr16	3943582	3950906	-	2	circRNA	-	ENSOART00000004058	STK10	mRNA
circRNA144	16	7.98E+02	chr16	6809974	6812240	+	3	circRNA	-	ENSOART00000004890	GFM2	mRNA
circRNA145	79	3.28E+03	chr16	6821860	6830388	+	3	circRNA	-	ENSOART00000004890	GFM2	mRNA
circRNA146	175	7.89E+03	chr16	7849853	7871261	-	4	circRNA	-	ENSOART00000005259	ARHGEF28	mRNA
circRNA147	12	5.56E+02	chr16	8740812	8741379	-	3	circRNA	-	ENSOART00000005491	TNPO1	mRNA
circRNA148	213	1.26E+04	chr16	12833434	12858238	-	2	circRNA	-	ENSOART00000006469	MAST4	mRNA
circRNA149	71	3.74E+03	chr16	16961507	16972094	-	4	circRNA	-	ENSOART00000007362	IPO11	mRNA
circRNA15	21	1.03E+03	chr13	11589923	11593536	-	2	circRNA	-	ENSOART00000014628	SFMBT2	mRNA
circRNA150	55	4.53E+03	chr16	32628765	32630659	+	2	circRNA	-	ENSOART00000009725	OXCT1	mRNA
circRNA151	199	1.11E+04	chr16	35332036	35354339	+	3	circRNA	-	ENSOART00000010557	RICTOR	mRNA
circRNA152	42	2.66E+03	chr16	37927593	37929630	+	3	circRNA	-	ENSOART00000011840	NADK2	mRNA
circRNA153	19	1.11E+03	chr16	39996063	39997596	+	2	circRNA	-	ENSOART00000013051	ADAMTS12	mRNA
circRNA154	27	1.16E+03	chr16	40078745	40080785	+	2	circRNA	-	ENSOART00000013051	ADAMTS12	mRNA
circRNA155	21	7.96E+02	chr16	58451216	58452723	-	4	circRNA	-	ENSOART00000014306	FAM105A	mRNA
circRNA156	42	2.89E+03	chr16	58648814	58652333	-	2	circRNA	-	ENSOART00000014452	TRIO	mRNA
circRNA157	268	1.63E+04	chr16	62441360	62465984	-	2	circRNA	-	ENSOART00000015194	ANKRD33B	mRNA
circRNA158	26	1.56E+03	chr16	62663567	62665716	-	2	circRNA	-	ENSOART00000015334	MARCH6	mRNA
circRNA159	32	2.07E+03	chr16	65299099	65301617	-	2	circRNA	-	ENSOART00000016014	ADCY2	mRNA
circRNA16	26	1.33E+03	chr13	15812920	15816515	+	3	circRNA	-	ENSOART00000016159	DHTKD1	mRNA
circRNA160	31	1.75E+03	chr16	65414165	65415221	-	2	circRNA	-	ENSOART00000016014	ADCY2	mRNA
circRNA161	1073	5.66E+04	chr16	65502310	65614661	-	2	circRNA	-	ENSOART00000016014	ADCY2	mRNA
circRNA162	8	3.20E+02	chr15	14737902	14738909	+	2	circRNA	-	ENSOART00000009892	SESN3	mRNA
circRNA163	28	1.18E+03	chr15	17249708	17251492	+	3	circRNA	-	ENSOART00000012085	ACAT1	mRNA
circRNA164	107	5.43E+03	chr15	35961419	35975386	+	3	circRNA	-	ENSOART00000006335	SOX6	mRNA
circRNA165	14	9.48E+02	chr15	37837712	37839399	+	2	circRNA	-	ENSOART00000007547	COPB1	mRNA
circRNA166	171	4.92E+03	chr15	39420671	39440282	-	8	circRNA	-	ENSOART00000008788	TEAD1	mRNA
circRNA167	14	9.00E+02	chr15	39543820	39544075	-	1	circRNA	-	ENSOART00000008788	TEAD1	mRNA
circRNA168	45	1.73E+03	chr15	40359935	40368447	-	3	circRNA	-	ENSOART00000010466	USP47	mRNA
circRNA169	24	1.17E+03	chr15	42402236	42404844	+	2	circRNA	-	ENSOART00000012706	SBF2	mRNA
circRNA17	62	2.02E+03	chr13	16747680	16754570	-	4	circRNA	-	ENSOART00000017160	YME1L1	mRNA
circRNA170	74	1.32E+03	chr15	43087772	43094889	+	8	circRNA	-	ENSOART00000014753	SCUBE2	mRNA
circRNA171	166	8.06E+03	chr15	44606309	44622789	-	3	circRNA	-	ENSOART00000017299	PPFIBP2	mRNA
circRNA172	21	8.47E+02	chr15	46004429	46005272	+	3	circRNA	-	ENSOART00000019866	TRIM3	mRNA
circRNA173	38	1.35E+03	chr15	49585998	49592805	+	3	circRNA	-	ENSOART00000002376	NUP98	mRNA
circRNA174	25	1.29E+03	chr15	50699504	50704639	-	3	circRNA	-	ENSOART00000007620	FCHSD2	mRNA
circRNA175	9	4.75E+02	chr15	71641101	71642106	+	2	circRNA	-	ENSOART00000000201	API5	mRNA
circRNA176	24	1.15E+03	chr15	74659082	74660186	-	2	circRNA	-	ENSOART00000003926	AMBRA1	mRNA
circRNA177	17	8.06E+02	chr15	74929193	74930689	-	2	circRNA	-	ENSOART00000004602	CKAP5	mRNA
circRNA178	16	1.38E+03	chr15	78151534	78152483	+	2	circRNA	-	ENSOART00000011308	ENSOARG00000010387	mRNA
circRNA179	46	1.78E+03	chr14	7661148	7665709	+	5	circRNA	-	ENSOART00000009582	CMIP	mRNA
circRNA18	26	1.10E+03	chr13	17266556	17270110	-	3	circRNA	-	ENSOART00000018356	CREM	mRNA
circRNA180	30	2.03E+03	chr14	12423679	12424493	-	2	circRNA	-	ENSOART00000012763	FBXO31	mRNA
circRNA181	28	9.16E+02	chr14	14514911	14518028	-	4	circRNA	-	ENSOART00000016886	VPS35	mRNA
circRNA182	192	6.14E+03	chr14	15425776	15448081	+	5	circRNA	-	ENSOART00000017880	PHKB	mRNA
circRNA183	189	9.04E+03	chr14	21692646	21710921	+	3	circRNA	-	ENSOART00000019523	FTO	mRNA
circRNA184	42	2.34E+03	chr14	23725372	23727408	+	2	circRNA	-	ENSOART00000020099	AMFR	mRNA
circRNA185	7	3.33E+02	chr14	34573461	34573806	-	1	circRNA	-	ENSOART00000003400	GFOD2	mRNA

circRNA186	69	4.27E+03	chr14	35601264	35610399	+	2	circRNA	-	ENSOART00000003783	TANGO6	mRNA
circRNA187	12	5.72E+02	chr14	42711141	42711890	-	2	circRNA	-	ENSOART00000004725	RHPN2	mRNA
circRNA188	35	1.30E+03	chr14	44132207	44137891	+	5	circRNA	-	ENSOART00000004935	UBA2	mRNA
circRNA189	3	3.43E+02	chr14	44890347	44890578	+	2	circRNA	-	ENSOART00000005104	HPN	mRNA
circRNA19	22	1.01E+03	chr13	17850443	17852392	+	2	circRNA	-	ENSOART00000018716	PARD3	mRNA
circRNA190	10	8.40E+02	chr14	45925097	45925432	+	2	circRNA	-	ENSOART00000005756	CAPNS1	mRNA
circRNA191	89	4.48E+03	chr14	47462820	47463744	+	2	circRNA	-	ENSOART00000006242	RYR1	mRNA
circRNA192	104	3.91E+03	chr14	47501768	47504888	+	5	circRNA	-	ENSOART00000006242	RYR1	mRNA
circRNA193	413	2.36E+04	chr14	52028517	52029204	-	2	circRNA	-	ENSOART00000010645	CKM	mRNA
circRNA194	65	3.74E+03	chr14	53246131	53250332	+	2	circRNA	-	ENSOART00000011902	SAE1	mRNA
circRNA195	53	1.51E+03	chr14	53408302	53412751	+	3	circRNA	-	ENSOART00000012020	DHX34	mRNA
circRNA196	15	1.04E+03	chr14	54076293	54076819	-	2	circRNA	-	ENSOART00000012709	CCDC114	mRNA
circRNA197	37	2.20E+03	chr14	54531501	54532042	+	2	circRNA	-	ENSOART00000013519	NUCB1	mRNA
circRNA198	52	2.76E+03	chr14	54589699	54590252	-	3	circRNA	-	ENSOART00000013559	GYS1	mRNA
circRNA199	14	6.53E+02	chr14	55493496	55496140	+	3	circRNA	-	ENSOART00000015025	MYH14	mRNA
circRNA2	128	3.72E+03	chrX	7175476	7186650	-	4	circRNA	-	ENSOART00000011153	CLCN4	mRNA
circRNA20	86	2.30E+03	chr13	17867372	17876443	+	4	circRNA	-	ENSOART00000018716	PARD3	mRNA
circRNA200	73	2.22E+03	chr14	56370506	56373586	-	4	circRNA	-	ENSOART00000016035	ENSOARG00000014733	mRNA
circRNA201	11	8.12E+02	chr14	60658581	60659008	-	2	circRNA	-	ENSOART00000003595	ZNF583	mRNA
circRNA202	10	5.47E+02	chr19	10691554	10693241	+	2	circRNA	-	ENSOART00000000093	DCLK3	mRNA
circRNA203	74	4.56E+03	chr19	10805228	10812086	+	3	circRNA	-	ENSOART00000000320	GOLGA4	mRNA
circRNA204	26	1.84E+03	chr19	13304347	13305123	+	2	circRNA	-	ENSOART00000003017	ENSOARG00000002789	mRNA
circRNA205	34	1.15E+03	chr19	15427733	15432507	-	3	circRNA	-	ENSOART00000004456	ANO10	mRNA
circRNA206	20	1.05E+03	chr19	16188824	16190730	+	3	circRNA	-	ENSOART00000004809	TCAIM	mRNA
circRNA207	45	1.60E+03	chr19	21096552	21100383	-	4	circRNA	-	ENSOART00000008450	EDEM1	mRNA
circRNA208	71	4.43E+03	chr19	21576535	21582094	-	2	circRNA	-	ENSOART00000008881	ITPR1	mRNA
circRNA209	2	9.77E+01	chr19	21583982	21584431	-	2	circRNA	-	ENSOART00000008881	ITPR1	mRNA
circRNA21	12	6.95E+02	chr13	24131472	24131755	+	1	circRNA	-	ENSOART00000003020	KIAA1217	mRNA
circRNA210	131	5.36E+03	chr19	34012036	34027397	+	3	circRNA	-	ENSOART00000011350	SUCLG2	mRNA
circRNA211	16	8.20E+02	chr19	35083861	35084181	+	1	circRNA	-	ENSOART00000011480	LRIG1	mRNA
circRNA212	118	4.62E+03	chr19	35202828	35227047	-	4	circRNA	-	ENSOART00000011590	SLC25A26	mRNA
circRNA213	97	6.07E+03	chr19	35202828	35223555	-	3	circRNA	-	ENSOART00000011590	SLC25A26	mRNA
circRNA214	195	1.00E+04	chr19	43406045	43432970	-	4	circRNA	-	ENSOART00000014292	SLMAP	mRNA
circRNA215	11	9.48E+02	chr19	43629122	43629777	+	2	circRNA	-	ENSOART00000014756	ARF4	mRNA
circRNA216	32	9.25E+02	chr19	43991191	43994885	+	5	circRNA	-	ENSOART00000015978	IL17RD	mRNA
circRNA217	93	4.01E+03	chr19	47822588	47829830	+	3	circRNA	-	ENSOART00000000406	ENSOARG00000000381	mRNA
circRNA218	71	4.67E+03	chr19	47923693	47932306	+	2	circRNA	-	ENSOART00000000491	SFMBT1	mRNA
circRNA219	8	4.39E+02	chr19	48119069	48119849	+	2	circRNA	-	ENSOART00000002108	NEK4	mRNA
circRNA22	255	5.33E+03	chr13	29409258	29440886	-	9	circRNA	-	ENSOART00000009595	FAM188A	mRNA
circRNA220	17	8.94E+02	chr19	48140673	48142894	+	2	circRNA	-	ENSOART00000002355	GLT8D1	mRNA
circRNA221	74	2.24E+03	chr19	48159026	48172212	+	4	circRNA	-	ENSOART00000002985	PBRM1	mRNA
circRNA222	58	2.47E+03	chr19	50709989	50715778	+	3	circRNA	-	ENSOART00000015204	USP4	mRNA
circRNA223	34	1.82E+03	chr19	53300744	53305972	+	2	circRNA	-	ENSOART00000009958	FYCO1	mRNA
circRNA224	37	1.60E+03	chr19	53393083	53398900	+	3	circRNA	-	ENSOART00000010422	LZTFL1	mRNA
circRNA225	29	1.59E+03	chr19	53513271	53517602	-	3	circRNA	-	ENSOART00000011059	SACM1L	mRNA
circRNA226	13	1.17E+03	chr19	53543333	53544085	-	2	circRNA	-	ENSOART00000011306	LIMD1	mRNA
circRNA227	121.5	4.39E+03	chr19	54127209	54139069	+	2	circRNA	-	ENSOART00000012351	ZDHHC3	mRNA
circRNA228	4.5	2.24E+02	chr19	54127209	54127538	+	1	circRNA	-	ENSOART00000012351	ZDHHC3	mRNA
circRNA229	48	8.45E+02	chr19	55939601	55940535	+	1	circRNA	-	ENSOART00000014351	TMCC1	mRNA
circRNA23	70	5.36E+03	chr13	31291295	31297994	+	2	circRNA	-	ENSOART00000012806	TMEM236	mRNA
circRNA230	12	6.02E+02	chr19	57118730	57119820	-	2	circRNA	-	ENSOART00000018953	NR2C2	mRNA
circRNA231	59	1.86E+03	chr19	57263366	57266919	-	5	circRNA	-	ENSOART00000019364	FGD5	mRNA
circRNA232	13	6.73E+02	chr19	57584040	57584467	+	2	circRNA	-	ENSOART00000020209	GRIP2	mRNA
circRNA233	90	5.65E+03	chr19	59224897	59225941	-	2	circRNA	-	ENSOART00000005006	EEFSEC	mRNA
circRNA234	31	1.31E+03	chr18	5130912	5133227	-	2	circRNA	-	ENSOART00000010515	ASB7	mRNA
circRNA235	12	1.03E+03	chr18	6074186	6074377	-	1	circRNA	-	ENSOART00000010750	MEF2A	mRNA
circRNA236	181	4.96E+03	chr18	6609859	6628646	+	4	circRNA	-	ENSOART00000010783	TTC23	mRNA
circRNA237	20	1.33E+03	chr18	13218977	13219223	-	1	circRNA	-	ENSOART00000011273	ENSOARG00000010343	mRNA
circRNA238	93	4.02E+03	chr18	13296368	13302172	-	3	circRNA	-	ENSOART00000011273	ENSOARG00000010343	mRNA
circRNA239	3	2.36E+02	chr18	16268034	16268242	+	1	circRNA	-	ENSOART00000011692	AGBL1	mRNA
circRNA24	34	2.37E+03	chr13	33721112	33722660	-	2	circRNA	-	ENSOART00000015657	SVIL	mRNA
circRNA240	114	4.23E+03	chr18	16268034	16280779	+	3	circRNA	-	ENSOART00000011692	AGBL1	mRNA
circRNA241	45	2.44E+03	chr18	19723286	19726561	+	2	circRNA	-	ENSOART00000012273	ABHD2	mRNA
circRNA242	56	3.34E+03	chr18	20391864	20398803	-	3	circRNA	-	ENSOART00000012998	ENSOARG00000011951	mRNA
circRNA243	26	1.48E+03	chr18	20592976	20593445	-	2	circRNA	-	ENSOART00000013070	IDH2	mRNA
circRNA244	71	3.83E+03	chr18	20594294	20594689	-	2	circRNA	-	ENSOART00000013070	IDH2	mRNA
circRNA245	26	1.73E+03	chr18	21833526	21835176	+	2	circRNA	-	ENSOART00000014565	PDE8A	mRNA
circRNA246	53	1.94E+03	chr18	22146431	22151056	-	3	circRNA	-	ENSOART00000015019	FSD2	mRNA
circRNA247	41	1.11E+03	chr18	31199497	31204593	-	5	circRNA	-	ENSOART00000001819	TSPAN3	mRNA
circRNA248	6	3.70E+02	chr18	31204078	31204593	-	2	circRNA	-	ENSOART00000001819	TSPAN3	mRNA
circRNA249	357	1.15E+04	chr18	34109004	34146066	-	3	circRNA	-	ENSOART00000005297	STXBP6	mRNA
circRNA25	27	1.84E+03	chr13	37464849	37467374	+	2	circRNA	-	ENSOART00000019958	CSRP2BP	mRNA
circRNA250	14	7.43E+02	chr18	40669344	40669834	-	2	circRNA	-	ENSOART00000006564	HECTD1	mRNA
circRNA251	53	1.05E+03	chr18	40834659	40841439	-	5	circRNA	-	ENSOART00000006810	HEATR5A	mRNA
circRNA252	612	1.17E+04	chr18	41990451	42071988	+	5	circRNA	-	ENSOART00000007129	AKAP6	mRNA
circRNA253	50	1.75E+03	chr18	44755701	44763617	-	4	circRNA	-	ENSOART00000008387	RALGAPA1	mRNA
circRNA254	14	9.44E+02	chr18	48130470	48132328	+	2	circRNA	-	ENSOART00000009476	ENSOARG00000008691	mRNA
circRNA255	63	4.49E+03	chr18	56532744	56538046	-	2	circRNA	-	ENSOART00000014644	ITPK1	mRNA
circRNA256	58	4.32E+03	chr18	60358445	60363401	+	2	circRNA	-	ENSOART00000000112	VRK1	mRNA
circRNA257	118	7.89E+03	chr18	66736432	66753040	+	2	circRNA	-	ENSOART00000006210	MARK3	mRNA
circRNA258	6	5.14E+02	chr18	66736432	66736623	+	1	circRNA	-	ENSOART00000006210	MARK3	mRNA
circRNA259	46	1.23E+03	chr18	67015980	67021903	-	4	circRNA	-	ENSOART00000006954	PPP1R13B	mRNA
circRNA26	3	1.57E+02	chr13	42111566	42113242	-	3	circRNA	-	ENSOART00000009106	FAM208B	mRNA
circRNA260	42	1.37E+03	chr18	68256540	68259331	-	5	circRNA	-	ENSOART00000009207	BRF1	mRNA
circRNA261	384	1.14E+04	chr26	1190346	1197933	+	5	circRNA	-	ENSOART00000006262	MYOM2	mRNA
circRNA262	24	9.75E+02	chr26	6781132	6783170	-	2	circRNA	-	ENSOART00000007405	VEGFC	mRNA
circRNA263	17	1.24E+03	chr26	7417085	7418202	-	2	circRNA	-	ENSOART00000007460	AGA	mRNA
circRNA264	88	5.46E+03	chr26	18864724	18875180	-	3	circRNA	-	ENSOART00000010409	MICU3	mRNA
circRNA265	15	2.15E+02	chr26	22208318	22209465	+	1	circRNA	-	ENSOART00000010616	DLC1	mRNA
circRNA266	95	5.37E+03	chr26	22312260	22320214	+	2	circRNA	-	ENSOART00000010610	DLC1	mRNA
circRNA267	14	5.91E+02	chr26	23746081	23747677	+	2	circRNA	-	ENSOART00000010906	ERI1	mRNA
circRNA268	6	3.39E+02	chr26	25421023	25422828	-	2	circRNA	-	ENSOART00000011321	GTF2E2	mRNA
circRNA269	37	2.06E+03	chr26	27831505	27831799	-	1	circRNA	-	ENSOART00000000368	FUT10	mRNA
circRNA27	44	2.28E+03	chr13	42837055	42840114	+	3	circRNA	-	ENSOART00000012684	ENSOARG00000011663	mRNA
circRNA270	6	2.73E+02	chr26	31952952	31953606	+	2	circRNA	-	ENSOART00000001293	ASH2L	mRNA
circRNA271	27	1.89E+03	chr26	32125522	32126214	-	2	circRNA	-	ENSOART00000001552	WHSC1L1	mRNA
circRNA272	67	9.69E+02	chr26	32174799	32181898	+	6	circRNA	-	ENSOART00000001587	LETM2	mRNA
circRNA273	59	1.89E+03	chr26	32589681	32595801	+	5	circRNA	-	ENSOART00000001719	TACC1	mRNA

circRNA274	66	2.07E+03	chr26	32782004	32791906	+	5	circRNA	-	ENSOART00000001955	ADAM9	mRNA
circRNA275	165	2.85E+03	chr26	32788237	32812140	+	7	circRNA	-	ENSOART00000001955	ADAM9	mRNA
circRNA276	8	3.32E+02	chr26	37138728	37139123	+	1	circRNA	-	ENSOART00000004427	PSD3	mRNA
circRNA277	970.5	4.87E+04	chr26	37228705	37342527	+	3	circRNA	-	ENSOART00000004427	PSD3	mRNA
circRNA278	65	2.96E+03	chr26	38522269	38528263	+	2	circRNA	-	ENSOART00000004527	NGLY1	mRNA
circRNA279	71	3.11E+03	chr24	246959	250171	-	3	circRNA	-	ENSOART00000012651	AXIN1	mRNA
circRNA28	23	8.72E+02	chr13	46978110	46981351	-	3	circRNA	-	ENSOART00000019186	GPCPD1	mRNA
circRNA280	30	1.53E+03	chr24	3298551	3299406	-	2	circRNA	-	ENSOART00000003179	ADCY9	mRNA
circRNA281	17	5.94E+02	chr24	10566200	10568909	-	4	circRNA	-	ENSOART00000007716	GSPT1	mRNA
circRNA282	6	3.34E+02	chr24	10568511	10568909	-	2	circRNA	-	ENSOART00000007716	GSPT1	mRNA
circRNA283	219	1.48E+04	chr24	10701841	10725440	+	3	circRNA	-	ENSOART00000007788	ENSOARG00000007156	mRNA
circRNA284	18	7.88E+02	chr24	10754616	10754991	+	1	circRNA	-	ENSOART00000007788	ENSOARG00000007156	mRNA
circRNA285	23	9.91E+02	chr24	15765059	15766630	+	2	circRNA	-	ENSOART00000011275	XYLT1	mRNA
circRNA286	50	1.87E+03	chr24	15822398	15827152	+	2	circRNA	-	ENSOART00000011275	XYLT1	mRNA
circRNA287	6	2.15E+02	chr24	16430652	16431240	-	2	circRNA	-	ENSOART00000011912	ENSOARG00000010928	mRNA
circRNA288	37	1.33E+03	chr24	16477407	16480580	-	3	circRNA	-	ENSOART00000011903	ENSOARG00000010928	mRNA
circRNA289	19	9.65E+02	chr24	19967580	19968014	+	2	circRNA	-	ENSOART00000017035	EEF2K	mRNA
circRNA29	53	1.68E+03	chr13	46999890	47008442	-	3	circRNA	-	ENSOART00000019186	GPCPD1	mRNA
circRNA290	75	4.46E+03	chr24	20006108	20007527	+	3	circRNA	-	ENSOART00000016923	POLR3E	mRNA
circRNA291	23	8.30E+02	chr24	20813370	20815893	-	4	circRNA	-	ENSOART00000017909	USP31	mRNA
circRNA292	13	4.84E+02	chr24	22222817	22223369	+	2	circRNA	-	ENSOART00000019310	RBBP6	mRNA
circRNA293	9	5.53E+02	chr24	25202324	25202591	+	1	circRNA	-	ENSOART00000000479	KIAA0556	mRNA
circRNA294	158	8.02E+03	chr24	25938947	25939793	-	3	circRNA	-	ENSOART00000002308	ATP2A1	mRNA
circRNA295	485	1.32E+04	chr24	25942934	25946810	-	4	circRNA	-	ENSOART00000002308	ATP2A1	mRNA
circRNA296	20	6.34E+02	chr24	27382679	27383843	+	5	circRNA	-	ENSOART00000009807	ITGAM	mRNA
circRNA297	12	6.98E+02	chr24	34465660	34467010	+	2	circRNA	-	ENSOART00000015412	ENSOARG00000014151	mRNA
circRNA298	28	7.74E+02	chr24	34487117	34488392	-	4	circRNA	-	ENSOART00000015541	LRWD1	mRNA
circRNA299	8	5.90E+02	chr24	38386719	38387438	-	2	circRNA	-	ENSOART00000002764	ZNF12	mRNA
circRNA3	99	4.71E+03	chrX	14526431	14536166	+	3	circRNA	-	ENSOART00000014491	REPS2	mRNA
circRNA30	62	4.22E+03	chr13	47008319	47019299	-	3	circRNA	-	ENSOART00000019186	GPCPD1	mRNA
circRNA300	142	5.63E+03	chr24	39741517	39762828	-	3	circRNA	-	ENSOART00000004657	ENSOARG00000004277	mRNA
circRNA301	111	6.26E+03	chr25	4663121	4677035	+	2	circRNA	-	ENSOART00000003566	DISC1	mRNA
circRNA302	158	3.48E+03	chr25	6270279	6290921	+	4	circRNA	-	ENSOART00000003686	ENSOARG00000003390	mRNA
circRNA303	39.5	2.77E+03	chr25	9391627	9392743	+	2	circRNA	-	ENSOART00000004036	ACTN2	mRNA
circRNA304	261.5	7.54E+03	chr25	9391627	9406699	+	6	circRNA	-	ENSOART00000004036	ACTN2	mRNA
circRNA305	52	2.41E+03	chr25	14209596	14214854	+	3	circRNA	-	ENSOART00000004452	BICC1	mRNA
circRNA306	20	1.12E+03	chr25	14839142	14840864	-	2	circRNA	-	ENSOART00000004558	FAM13C	mRNA
circRNA307	341	2.14E+04	chr25	14867929	14902606	-	3	circRNA	-	ENSOART00000004558	FAM13C	mRNA
circRNA308	2	1.46E+02	chr25	17829286	17829511	+	1	circRNA	-	ENSOART00000004794	ARID5B	mRNA
circRNA309	4	2.49E+02	chr25	23563600	23563863	-	1	circRNA	-	ENSOART00000005164	ENSOARG00000004744	mRNA
circRNA31	102	1.62E+03	chr13	50620903	50632647	-	5	circRNA	-	ENSOART00000000805	PANK2	mRNA
circRNA310	150	3.62E+03	chr25	24339533	24357551	-	5	circRNA	-	ENSOART00000005279	HERC4	mRNA
circRNA311	32	2.31E+03	chr25	24440021	24443215	+	2	circRNA	-	ENSOART00000005367	MYPN	mRNA
circRNA312	190	3.85E+03	chr25	25502894	25524953	+	6	circRNA	-	ENSOART00000005999	HK1	mRNA
circRNA313	235	1.17E+04	chr25	28068316	28101367	-	3	circRNA	-	ENSOART00000007721	ASCC1	mRNA
circRNA314	23	1.15E+03	chr25	42351179	42354662	+	3	circRNA	-	ENSOART00000001574	MAPK8	mRNA
circRNA315	32	1.88E+03	chr22	261460	265317	-	4	circRNA	-	ENSOART00000014055	UBE2D1	mRNA
circRNA316	19	1.02E+03	chr22	6733643	6735823	-	2	circRNA	-	ENSOART00000014821	PRKG1	mRNA
circRNA317	223	1.12E+04	chr22	6747502	6773314	-	3	circRNA	-	ENSOART00000014821	PRKG1	mRNA
circRNA318	55	2.41E+03	chr22	13084911	13087599	+	3	circRNA	-	ENSOART00000017755	TNKS2	mRNA
circRNA319	42	1.62E+03	chr22	13188863	13199361	+	3	circRNA	-	ENSOART00000018006	BTAF1	mRNA
circRNA32	28	1.40E+03	chr13	51152014	51154493	+	3	circRNA	-	ENSOART00000003375	C20orf194	mRNA
circRNA320	31	1.98E+03	chr22	13369228	13371219	-	2	circRNA	-	ENSOART00000018157	CPEB3	mRNA
circRNA321	48	2.45E+03	chr22	14698926	14705211	-	4	circRNA	-	ENSOART00000003779	FRA10AC1	mRNA
circRNA322	130	4.39E+03	chr22	16145805	16159821	-	5	circRNA	-	ENSOART00000006595	SORBS1	mRNA
circRNA323	8	5.55E+02	chr22	16147678	16149498	-	2	circRNA	-	ENSOART00000006595	SORBS1	mRNA
circRNA324	59	1.90E+03	chr22	21584852	21595148	-	4	circRNA	-	ENSOART00000018296	FBXW4	mRNA
circRNA325	39.5	1.57E+03	chr22	21584852	21592062	-	3	circRNA	-	ENSOART00000018296	FBXW4	mRNA
circRNA326	7.5	3.87E+02	chr22	21591311	21592062	-	2	circRNA	-	ENSOART00000018296	FBXW4	mRNA
circRNA327	27	1.07E+03	chr22	21591311	21595148	-	3	circRNA	-	ENSOART00000018296	FBXW4	mRNA
circRNA328	16	1.06E+03	chr22	21732004	21732517	-	3	circRNA	-	ENSOART00000018575	KCNIP2	mRNA
circRNA329	61	5.46E+03	chr22	31999188	32010639	+	3	circRNA	-	ENSOART00000013314	VTI1A	mRNA
circRNA33	34	1.89E+03	chr13	51506900	51508527	-	3	circRNA	-	ENSOART00000005401	PTPRA	mRNA
circRNA330	47	2.50E+03	chr22	33020719	33023088	-	3	circRNA	-	ENSOART00000013855	NRAP	mRNA
circRNA331	32	2.51E+03	chr22	33081797	33083387	-	2	circRNA	-	ENSOART00000013855	NRAP	mRNA
circRNA332	101	3.41E+03	chr22	38853745	38866754	+	4	circRNA	-	ENSOART00000003464	INPP5F	mRNA
circRNA333	187	1.22E+04	chr22	40850131	40868489	+	3	circRNA	-	ENSOART00000007320	TACC2	mRNA
circRNA334	176	1.11E+04	chr22	40936900	40953529	+	2	circRNA	-	ENSOART00000007320	TACC2	mRNA
circRNA335	34	2.11E+03	chr22	41040543	41042685	+	3	circRNA	-	ENSOART00000007320	TACC2	mRNA
circRNA336	24	1.37E+03	chr22	45410286	45411717	+	2	circRNA	-	ENSOART00000014233	DOCK1	mRNA
circRNA337	529	3.03E+04	chr22	45438453	45491775	+	3	circRNA	-	ENSOART00000014233	DOCK1	mRNA
circRNA338	45.5	2.18E+03	chr23	32656398	32662441	+	3	circRNA	-	ENSOART00000008061	OSBPL1A	mRNA
circRNA339	9.5	6.77E+02	chr23	32661096	32662441	+	2	circRNA	-	ENSOART00000008061	OSBPL1A	mRNA
circRNA34	21	8.27E+02	chr13	51528470	51530566	-	2	circRNA	-	ENSOART00000005401	PTPRA	mRNA
circRNA340	22	1.50E+03	chr23	32825799	32826612	+	2	circRNA	-	ENSOART00000008061	OSBPL1A	mRNA
circRNA341	107	4.04E+03	chr23	32860807	32873765	-	4	circRNA	-	ENSOART00000008244	TTC39C	mRNA
circRNA342	45	2.07E+03	chr23	33617943	33621601	-	3	circRNA	-	ENSOART00000008876	CABLES1	mRNA
circRNA343	31	1.69E+03	chr23	34803384	34805489	-	2	circRNA	-	ENSOART00000009044	MIB1	mRNA
circRNA344	19	5.83E+02	chr23	34982391	34984997	-	3	circRNA	-	ENSOART00000009303	GREB1L	mRNA
circRNA345	32	1.33E+03	chr23	37501058	37503192	-	2	circRNA	-	ENSOART00000010970	LPIN2	mRNA
circRNA346	15	5.70E+02	chr23	43229457	43231633	+	4	circRNA	-	ENSOART00000001933	SLMO1	mRNA
circRNA347	44	2.98E+03	chr23	43282769	43284768	-	2	circRNA	-	ENSOART00000001976	SPIRE1	mRNA
circRNA348	18	1.10E+03	chr23	49192997	49194555	-	2	circRNA	-	ENSOART00000004215	DYM	mRNA
circRNA349	11	6.94E+02	chr23	54445913	54447893	+	2	circRNA	-	ENSOART00000005394	RAB27B	mRNA
circRNA35	34	1.03E+03	chr13	57012344	57015563	-	4	circRNA	-	ENSOART00000018566	ENSOARG00000017053	mRNA
circRNA350	9	5.81E+02	chr23	54505185	54507212	-	2	circRNA	-	ENSOART00000005430	CCDC68	mRNA
circRNA351	23	1.58E+03	chr23	54858761	54863253	-	2	circRNA	-	ENSOART00000005468	TCF4	mRNA
circRNA352	45	2.06E+03	chr23	58208176	58212690	+	2	circRNA	-	ENSOART00000006072	ZNF532	mRNA
circRNA353	21	1.13E+03	chr20	3100277	3103180	+	2	circRNA	-	ENSOART00000006258	RAB23	mRNA
circRNA354	142	3.66E+03	chr20	3100277	3116872	+	5	circRNA	-	ENSOART00000006258	RAB23	mRNA
circRNA355	342	1.31E+04	chr20	6379246	6418787	-	6	circRNA	-	ENSOART00000007388	MLIP	mRNA
circRNA356	152	6.21E+03	chr20	9733122	9750912	-	3	circRNA	-	ENSOART00000013346	FKBP5	mRNA
circRNA357	9	6.44E+02	chr20	11010704	11011610	+	2	circRNA	-	ENSOART00000015562	FGD2	mRNA
circRNA358	56	3.90E+03	chr20	19282511	19286771	-	2	circRNA	-	ENSOART00000011394	CLIC5	mRNA
circRNA359	93	3.89E+03	chr20	23865153	23876616	-	3	circRNA	-	ENSOART00000014476	PKHD1	mRNA
circRNA36	100	2.65E+03	chr13	59836453	59849556	+	7	circRNA	-	ENSOART00000020745	ENSOARG00000019053	mRNA
circRNA360	14	1.03E+03	chr20	23881828	23882876	-	2	circRNA	-	ENSOART00000014476	PKHD1	mRNA
circRNA361	5	3.04E+02	chr20	32222598	32223113	-	2	circRNA	-	ENSOART00000007976	MRS2	mRNA

circRNA362	249	1.33E+04	chr20	38871274	38905373	-	3	circRNA	-	ENSOART00000012878	CAP2	mRNA
circRNA363	40.5	3.53E+03	chr20	39803459	39807129	-	2	circRNA	-	ENSOART00000013290	GMPR	mRNA
circRNA364	139.5	6.32E+03	chr20	39803459	39819375	-	3	circRNA	-	ENSOART00000013290	GMPR	mRNA
circRNA365	25	6.11E+02	chr20	48568916	48569592	-	2	circRNA	-	ENSOART00000000076	ENSOARG00000000065	mRNA
circRNA366	6.5	4.63E+02	chr21	16880801	16881391	+	2	circRNA	-	ENSOART00000006830	NARS2	mRNA
circRNA367	93.5	3.40E+03	chr21	16880801	16893825	+	4	circRNA	-	ENSOART00000006830	NARS2	mRNA
circRNA368	128	5.04E+03	chr21	17701928	17714326	+	5	circRNA	-	ENSOART00000007543	RSF1	mRNA
circRNA369	33	1.56E+03	chr21	28095125	28096716	+	3	circRNA	-	ENSOART00000012373	PKNOX2	mRNA
circRNA37	61	2.68E+03	chr13	60973768	60977600	+	4	circRNA	-	ENSOART00000003322	TM9SF4	mRNA
circRNA370	39	1.49E+03	chr21	31634322	31639191	-	4	circRNA	-	ENSOART00000014554	ARHGAP32	mRNA
circRNA371	27	1.49E+03	chr21	35469791	35470928	+	2	circRNA	-	ENSOART00000016027	APLP2	mRNA
circRNA372	30	1.20E+03	chr21	39767054	39769052	+	3	circRNA	-	ENSOART00000015072	FADS2	mRNA
circRNA373	44	2.35E+03	chr21	45320692	45326050	+	3	circRNA	-	ENSOART00000017200	PPP6R3	mRNA
circRNA374	33	1.05E+03	chr21	49411885	49414425	+	4	circRNA	-	ENSOART00000006836	DEAF1	mRNA
circRNA375	16	1.01E+03	chr21	49826411	49827573	-	3	circRNA	-	ENSOART00000009443	PSMD13	mRNA
circRNA376	43	2.03E+03	chr7	10180187	10185131	-	3	circRNA	-	ENSOART00000018827	HOMER1	mRNA
circRNA377	123.5	3.89E+03	chr7	10226343	10245400	-	4	circRNA	-	ENSOART00000018827	HOMER1	mRNA
circRNA378	42.5	1.83E+03	chr7	10235060	10245400	-	3	circRNA	-	ENSOART00000018827	HOMER1	mRNA
circRNA379	155	6.47E+03	chr7	10505870	10519525	+	3	circRNA	-	ENSOART00000018985	CMYA5	mRNA
circRNA38	116.5	7.23E+03	chr13	65532712	65546143	-	3	circRNA	-	ENSOART00000018309	SAMHD1	mRNA
circRNA380	16	9.44E+02	chr7	11895572	11897186	-	2	circRNA	-	ENSOART00000019391	CLPX	mRNA
circRNA381	29	1.72E+03	chr7	12114103	12115610	-	2	circRNA	-	ENSOART00000019498	IGDCC4	mRNA
circRNA382	26	9.84E+02	chr7	12170335	12172421	-	3	circRNA	-	ENSOART00000019531	DPP8	mRNA
circRNA383	11	4.79E+02	chr7	13858209	13859053	-	3	circRNA	-	ENSOART00000019905	AAGAB	mRNA
circRNA384	78	3.29E+03	chr7	17370348	17379430	-	5	circRNA	-	ENSOART00000020402	UACA	mRNA
circRNA385	10	1.08E+03	chr7	21204458	21204609	-	1	circRNA	-	ENSOART00000021058	ENSOARG00000019336	mRNA
circRNA386	102	2.11E+03	chr7	34703909	34715052	+	6	circRNA	-	ENSOART00000022357	GANC	mRNA
circRNA387	74	3.81E+03	chr7	35126419	35134381	-	3	circRNA	-	ENSOART00000022411	TTBK2	mRNA
circRNA388	21	1.74E+03	chr7	35438999	35441681	+	2	circRNA	-	ENSOART00000022427	TMEM62	mRNA
circRNA389	20	1.47E+03	chr7	35446192	35447845	+	2	circRNA	-	ENSOART00000022427	TMEM62	mRNA
circRNA39	11	5.13E+02	chr13	72488970	72489885	-	2	circRNA	-	ENSOART00000004419	SERINC3	mRNA
circRNA390	50	5.05E+03	chr7	39808905	39810673	+	2	circRNA	-	ENSOART00000022453	KLHDC1	mRNA
circRNA391	19	3.58E+02	chr7	42641887	42642760	-	1	circRNA	-	ENSOART00000022589	ZNF609	mRNA
circRNA392	36	1.99E+03	chr7	42922577	42923952	-	3	circRNA	-	ENSOART00000022611	SNX1	mRNA
circRNA393	9	4.50E+02	chr7	43397802	43398957	+	2	circRNA	-	ENSOART00000022636	HERC1	mRNA
circRNA394	23	1.49E+03	chr7	48125999	48127213	+	2	circRNA	-	ENSOART00000022697	MYO1E	mRNA
circRNA395	31	1.45E+03	chr7	50657545	50660702	+	3	circRNA	-	ENSOART00000022745	ZNF280D	mRNA
circRNA396	42	3.51E+03	chr7	54779731	54783630	+	2	circRNA	-	ENSOART00000022804	FAM214A	mRNA
circRNA397	54	2.57E+03	chr7	56743053	56751082	+	6	circRNA	-	ENSOART00000022863	TRPM7	mRNA
circRNA398	135	4.93E+03	chr7	58013694	58028055	-	4	circRNA	-	ENSOART00000022883	GALK2	mRNA
circRNA399	40	1.76E+03	chr7	64994909	65003314	+	5	circRNA	-	ENSOART00000022984	KTN1	mRNA
circRNA4	8	5.93E+02	chrX	38488319	38489668	+	2	circRNA	-	ENSOART00000002644	DDX3X	mRNA
circRNA40	22	1.13E+03	chr13	75352548	75353846	-	3	circRNA	-	ENSOART00000010261	ZMYND8	mRNA
circRNA400	28	9.22E+02	chr7	69272808	69275655	-	3	circRNA	-	ENSOART00000023017	DHRS7	mRNA
circRNA401	13	6.66E+02	chr7	73511258	73511927	+	3	circRNA	-	ENSOART00000023046	MTHFD1	mRNA
circRNA402	29	1.79E+03	chr7	85458334	85460286	-	2	circRNA	-	ENSOART00000002586	POMT2	mRNA
circRNA403	32	1.98E+03	chr7	85650491	85652906	-	2	circRNA	-	ENSOART00000002811	SPTLC2	mRNA
circRNA404	29	2.71E+03	chr7	97010441	97013958	+	2	circRNA	-	ENSOART00000003389	SPATA7	mRNA
circRNA405	53	3.73E+03	chr7	99075743	99079247	-	2	circRNA	-	ENSOART00000003829	TTC7B	mRNA
circRNA406	148	5.36E+03	chr7	99111008	99128569	-	4	circRNA	-	ENSOART00000003829	TTC7B	mRNA
circRNA407	44	2.87E+03	chr7	99121747	99128569	-	2	circRNA	-	ENSOART00000003829	TTC7B	mRNA
circRNA408	42	2.71E+03	chr6	6731653	6735075	+	2	circRNA	-	ENSOART00000018477	SEC24D	mRNA
circRNA409	26	1.13E+03	chr6	12409083	12412260	-	2	circRNA	-	ENSOART00000000594	ANK2	mRNA
circRNA41	9	6.17E+02	chr13	76712743	76713690	+	2	circRNA	-	ENSOART00000012363	ARFGEF2	mRNA
circRNA410	160	4.56E+03	chr6	17333785	17348913	-	5	circRNA	-	ENSOART00000008964	HADH	mRNA
circRNA411	32.5	2.46E+03	chr6	17338491	17341842	-	2	circRNA	-	ENSOART00000008964	HADH	mRNA
circRNA412	28.16666667	1.62E+03	chr6	17346383	17348913	-	2	circRNA	-	ENSOART00000008964	HADH	mRNA
circRNA413	4	3.52E+02	chr6	35926599	35926785	+	1	circRNA	-	ENSOART00000020389	ENSOARG00000018727	mRNA
circRNA414	10	4.61E+02	chr6	44737720	44738468	+	2	circRNA	-	ENSOART00000007458	ZCCHC4	mRNA
circRNA415	49	1.62E+03	chr6	59111748	59117863	+	3	circRNA	-	ENSOART00000013597	N4BP2	mRNA
circRNA416	28	1.68E+03	chr6	59737976	59740606	-	2	circRNA	-	ENSOART00000014198	APBB2	mRNA
circRNA417	61	2.42E+03	chr6	60517200	60523268	+	4	circRNA	-	ENSOART00000014786	LIMCH1	mRNA
circRNA418	32	1.81E+03	chr6	66173030	66175656	+	2	circRNA	-	ENSOART00000017357	ATP10D	mRNA
circRNA419	12	7.77E+02	chr6	66319020	66320396	-	2	circRNA	-	ENSOART00000017664	CORIN	mRNA
circRNA42	193	3.26E+03	chr13	76832967	76855116	-	6	circRNA	-	ENSOART00000013274	STAU1	mRNA
circRNA420	9	7.05E+02	chr6	67849934	67850143	-	1	circRNA	-	ENSOART00000020076	SGCB	mRNA
circRNA421	20	1.07E+03	chr6	88783856	88784788	+	2	circRNA	-	ENSOART00000016194	MTHFD2L	mRNA
circRNA422	28	1.21E+03	chr6	90271559	90276893	+	4	circRNA	-	ENSOART00000017165	USO1	mRNA
circRNA423	17	1.07E+03	chr6	90297565	90300360	+	2	circRNA	-	ENSOART00000017165	USO1	mRNA
circRNA424	48	2.00E+03	chr6	92105004	92111142	-	3	circRNA	-	ENSOART00000019338	CNOT6L	mRNA
circRNA425	36	1.09E+03	chr6	92866346	92871211	+	3	circRNA	-	ENSOART00000019879	FRAS1	mRNA
circRNA426	5	3.83E+02	chr6	92880898	92881112	+	1	circRNA	-	ENSOART00000019879	FRAS1	mRNA
circRNA427	19	5.51E+02	chr6	101203864	101204430	+	1	circRNA	-	ENSOART00000004473	PTPN13	mRNA
circRNA428	72.66666667	4.53E+03	chr5	2429061	2429324	-	1	circRNA	-	ENSOART00000005964	ENSOARG00000005438	mRNA
circRNA429	120.6666667	3.76E+03	chr5	2429061	2429915	-	2	circRNA	-	ENSOART00000005964	ENSOARG00000005438	mRNA
circRNA43	19	1.32E+03	chr13	77704879	77706265	-	2	circRNA	-	ENSOART00000014407	TMEM189-UBE2V1	mRNA
circRNA430	318.6666667	2.55E+03	chr5	2429061	2437306	-	12	circRNA	-	ENSOART00000005964	ENSOARG00000005438	mRNA
circRNA431	512.2833333	2.59E+03	chr5	2469820	2558987	-	14	circRNA	-	ENSOART00000005964	ENSOARG00000005438	mRNA
circRNA432	268.2833333	1.64E+03	chr5	2486267	2558987	-	12	circRNA	-	ENSOART00000005964	ENSOARG00000005438	mRNA
circRNA433	212.7833333	1.44E+03	chr5	2495954	2558987	-	9	circRNA	-	ENSOART00000005964	ENSOARG00000005438	mRNA
circRNA434	167.45	1.28E+03	chr5	2501234	2558987	-	8	circRNA	-	ENSOART00000005964	ENSOARG00000005438	mRNA
circRNA435	20.2	6.02E+02	chr5	2554864	2558987	-	2	circRNA	-	ENSOART00000005964	ENSOARG00000005438	mRNA
circRNA436	123	2.82E+03	chr5	6350960	6360478	+	6	circRNA	-	ENSOART00000020758	EPS15L1	mRNA
circRNA437	17	1.13E+03	chr5	9399662	9400083	-	3	circRNA	-	ENSOART00000006059	PKN1	mRNA
circRNA438	33	1.31E+03	chr5	9805231	9809105	+	4	circRNA	-	ENSOART00000007679	HGNC:9982	mRNA
circRNA439	146	3.24E+03	chr5	12657272	12665631	+	9	circRNA	-	ENSOART00000017688	PDE4A	mRNA
circRNA44	61	1.93E+03	chr12	1564409	1572324	+	5	circRNA	-	ENSOART00000003016	ENSOARG00000002783	mRNA
circRNA440	19	1.31E+03	chr5	13768275	13770421	-	2	circRNA	-	ENSOART00000000304	INSR	mRNA
circRNA441	21	6.26E+02	chr5	13856978	13857529	-	1	circRNA	-	ENSOART00000000304	INSR	mRNA
circRNA442	32	1.98E+03	chr5	16276759	16277104	-	2	circRNA	-	ENSOART00000008554	ENSOARG00000007856	mRNA
circRNA443	143	7.00E+03	chr5	19169711	19186266	+	3	circRNA	-	ENSOART00000016154	KIF3A	mRNA
circRNA444	29	1.31E+03	chr5	23356559	23359010	-	3	circRNA	-	ENSOART00000019177	SLC12A2	mRNA
circRNA445	17	9.58E+02	chr5	23377338	23379191	-	3	circRNA	-	ENSOART00000019177	SLC12A2	mRNA
circRNA446	38	1.46E+03	chr5	23959368	23964802	-	3	circRNA	-	ENSOART00000019331	PRRC1	mRNA
circRNA447	48	1.76E+03	chr5	24565604	24568974	+	2	circRNA	-	ENSOART00000019653	MARCH3	mRNA
circRNA448	7	4.50E+02	chr5	26536838	26537093	+	1	circRNA	-	ENSOART00000020270	ZNF608	mRNA
circRNA449	11.5	6.96E+02	chr5	27831028	27832892	+	2	circRNA	-	ENSOART00000020487	CEP120	mRNA
circRNA45	39	1.11E+03	chr12	20017554	20021404	+	4	circRNA	-	ENSOART00000012598	TGFB2	mRNA

circRNA450	39.5	1.57E+03	chr5	27831028	27837378	+	3	circRNA	-	ENSOART00000020487	CEP120	mRNA
circRNA451	34	2.27E+03	chr5	31873577	31878007	-	3	circRNA	-	ENSOART00000000797	HSD17B4	mRNA
circRNA452	5	4.70E+02	chr5	40764128	40764302	+	1	circRNA	-	ENSOART00000010256	PTBP1	mRNA
circRNA453	13	3.74E+02	chr5	44946024	44946595	+	1	circRNA	-	ENSOART00000016250	SMAD5	mRNA
circRNA454	17	1.53E+03	chr5	46732313	46733578	+	2	circRNA	-	ENSOART00000016880	ENSOARG00000015514	mRNA
circRNA455	20	9.94E+02	chr5	46812315	46815135	-	3	circRNA	-	ENSOART00000017082	FAM13B	mRNA
circRNA456	12	7.00E+02	chr5	47018160	47019057	-	3	circRNA	-	ENSOART00000017404	CDC23	mRNA
circRNA457	146	7.24E+03	chr5	51316656	51328883	+	4	circRNA	-	ENSOART00000002101	ARHGAP26	mRNA
circRNA458	9	5.49E+02	chr5	54693026	54693295	+	1	circRNA	-	ENSOART00000002533	SH3RF2	mRNA
circRNA459	31	1.45E+03	chr5	56068029	56069775	-	2	circRNA	-	ENSOART00000003920	DPYSL3	mRNA
circRNA46	22	1.19E+03	chr12	22372186	22373858	+	2	circRNA	-	ENSOART00000014276	MARC2	mRNA
circRNA460	249	6.76E+03	chr5	58092191	58115168	+	5	circRNA	-	ENSOART00000005457	ABLIM3	mRNA
circRNA461	70	3.26E+03	chr5	58692115	58697744	+	3	circRNA	-	ENSOART00000006163	PPARGC1B	mRNA
circRNA462	43	1.21E+03	chr5	59358238	59361664	+	2	circRNA	-	ENSOART00000008049	NDST1	mRNA
circRNA463	40	2.20E+03	chr5	59846756	59850143	-	3	circRNA	-	ENSOART00000008734	TNIP1	mRNA
circRNA464	48	1.08E+03	chr5	60524714	60529139	+	6	circRNA	-	ENSOART00000009949	G3BP1	mRNA
circRNA465	694	3.68E+04	chr5	65272815	65356197	+	3	circRNA	-	ENSOART00000011437	SGCD	mRNA
circRNA466	236	1.49E+04	chr5	67701161	67726882	-	2	circRNA	-	ENSOART00000013952	ENSOARG00000012836	mRNA
circRNA467	28	1.05E+03	chr5	77937109	77940020	+	3	circRNA	-	ENSOART00000015980	ZFYVE16	mRNA
circRNA468	31	2.17E+03	chr5	85636228	85638570	-	2	circRNA	-	ENSOART00000017533	MEF2C	mRNA
circRNA469	46	2.79E+03	chr5	93736412	93738186	+	2	circRNA	-	ENSOART00000019587	LNPEP	mRNA
circRNA47	122	5.84E+03	chr12	25521972	25539415	-	4	circRNA	-	ENSOART00000017449	NVL	mRNA
circRNA470	6	2.55E+02	chr5	99388272	99388658	+	1	circRNA	-	ENSOART00000020146	PPIP5K2	mRNA
circRNA471	63	1.82E+03	chr5	106090073	106098828	+	4	circRNA	-	ENSOART00000020561	MAN2A1	mRNA
circRNA472	35	1.33E+03	chr4	13421013	13425462	-	4	circRNA	-	ENSOART00000004450	SLC25A13	mRNA
circRNA473	8	4.99E+02	chr4	27354278	27354541	+	1	circRNA	-	ENSOART00000010499	HDAC9	mRNA
circRNA474	427	2.52E+04	chr4	27458210	27501097	+	3	circRNA	-	ENSOART00000010499	HDAC9	mRNA
circRNA475	16	8.58E+02	chr4	44450597	44450903	-	1	circRNA	-	ENSOART00000020610	NAPEPLD	mRNA
circRNA476	81	2.66E+03	chr4	51584122	51594142	-	3	circRNA	-	ENSOART00000000854	C-MET	mRNA
circRNA477	260	6.44E+03	chr4	56350156	56378779	+	7	circRNA	-	ENSOART00000003200	DOCK4	mRNA
circRNA478	167	6.99E+03	chr4	68469461	68488380	+	3	circRNA	-	ENSOART00000010121	ENSOARG00000009300	mRNA
circRNA479	94	4.20E+03	chr4	68551662	68560460	+	3	circRNA	-	ENSOART00000010121	ENSOARG00000009300	mRNA
circRNA48	37	9.92E+02	chr12	25566748	25573148	-	4	circRNA	-	ENSOART00000017449	NVL	mRNA
circRNA480	9	3.38E+02	chr4	70757605	70759054	+	2	circRNA	-	ENSOART00000011285	ENSOARG00000010371	mRNA
circRNA481	44	2.59E+03	chr4	77337381	77341631	-	2	circRNA	-	ENSOART00000018431	ENSOARG00000016920	mRNA
circRNA482	275	8.29E+03	chr4	85333879	85366206	-	5	circRNA	-	ENSOART00000019664	TSPAN12	mRNA
circRNA483	23	1.25E+03	chr4	85629860	85632140	+	2	circRNA	-	ENSOART00000019828	CPED1	mRNA
circRNA484	135	4.15E+03	chr4	95095031	95110907	+	4	circRNA	-	ENSOART00000006514	MKLN1	mRNA
circRNA485	333	2.74E+04	chr4	96664575	96703243	-	2	circRNA	-	ENSOART00000007340	CHCHD3	mRNA
circRNA486	13	6.15E+02	chr4	106161866	106162478	+	2	circRNA	-	ENSOART00000019494	CLCN1	mRNA
circRNA487	33	1.44E+03	chr4	113958414	113960958	+	2	circRNA	-	ENSOART00000005306	GALNT11	mRNA
circRNA488	37	1.33E+03	chr4	114135277	114140110	-	3	circRNA	-	ENSOART00000005681	KMT2C	mRNA
circRNA489	17	1.19E+03	chr4	114538607	114539539	+	2	circRNA	-	ENSOART00000006482	ENSOARG00000005960	mRNA
circRNA49	23	1.49E+03	chr12	25635145	25637667	+	2	circRNA	-	ENSOART00000017682	CNIH4	mRNA
circRNA490	240	5.16E+03	chr4	116785835	116811876	+	4	circRNA	-	ENSOART00000007415	RBM33	mRNA
circRNA491	34	1.47E+03	chr4	116806187	116811876	+	2	circRNA	-	ENSOART00000007415	RBM33	mRNA
circRNA492	156.5	7.21E+03	chr4	117670496	117694567	-	4	circRNA	-	ENSOART00000007692	LMBR1	mRNA
circRNA493	20.5	1.33E+03	chr4	117689458	117694567	-	3	circRNA	-	ENSOART00000007692	LMBR1	mRNA
circRNA494	87	3.65E+03	chr4	117897055	117905907	+	4	circRNA	-	ENSOART00000008030	UBE3C	mRNA
circRNA495	134	5.93E+03	chr4	118046402	118049973	+	4	circRNA	-	ENSOART00000008124	DNAJB6	mRNA
circRNA496	90.5	3.12E+03	chr3	302330	310088	-	4	circRNA	-	ENSOART00000000688	EHMT1	mRNA
circRNA497	47.5	1.54E+03	chr3	317117	320335	-	3	circRNA	-	ENSOART00000000688	EHMT1	mRNA
circRNA498	15	6.57E+02	chr3	1225364	1227933	-	2	circRNA	-	ENSOART00000002137	OLFM1	mRNA
circRNA499	15	5.81E+02	chr3	3408916	3410064	+	2	circRNA	-	ENSOART00000005353	CAMSAP1	mRNA
circRNA5	23	9.42E+02	chrX	49392545	49393713	+	3	circRNA	-	ENSOART00000008481	HUWE1	mRNA
circRNA50	41	9.68E+02	chr12	28263773	28270484	-	4	circRNA	-	ENSOART00000002411	CDC42BPA	mRNA
circRNA500	45	1.68E+03	chr3	3899765	3905347	+	3	circRNA	-	ENSOART00000006164	TSC1	mRNA
circRNA501	218	1.13E+04	chr3	3955640	3974132	+	4	circRNA	-	ENSOART00000006284	AK8	mRNA
circRNA502	17	9.75E+02	chr3	5502600	5504857	-	2	circRNA	-	ENSOART00000007684	NUP214	mRNA
circRNA503	28	1.41E+03	chr3	7279339	7281560	-	3	circRNA	-	ENSOART00000009586	PPP2R4	mRNA
circRNA504	123	5.78E+03	chr3	10443949	10459257	+	2	circRNA	-	ENSOART00000013849	SIN1	mRNA
circRNA505	6	3.66E+02	chr3	10650298	10650872	-	2	circRNA	-	ENSOART00000013931	GAPVD1	mRNA
circRNA506	29	1.53E+03	chr3	10730655	10734228	-	2	circRNA	-	ENSOART00000014067	RABEPK	mRNA
circRNA507	166	4.91E+03	chr3	23607491	23623874	-	4	circRNA	-	ENSOART00000017797	NBAS	mRNA
circRNA508	126	1.07E+04	chr3	23749229	23761853	-	2	circRNA	-	ENSOART00000017797	NBAS	mRNA
circRNA509	88	3.13E+03	chr3	32567103	32573785	-	3	circRNA	-	ENSOART00000019914	DNMT3A	mRNA
circRNA51	20	6.64E+02	chr12	28263773	28267902	-	3	circRNA	-	ENSOART00000002411	CDC42BPA	mRNA
circRNA510	34	2.45E+03	chr3	33300226	33303213	-	2	circRNA	-	ENSOART00000020243	HADHA	mRNA
circRNA511	59	3.00E+03	chr3	35876030	35884332	+	3	circRNA	-	ENSOART00000021356	CLIP4	mRNA
circRNA512	24	1.52E+03	chr3	35876030	35879658	+	2	circRNA	-	ENSOART00000021356	CLIP4	mRNA
circRNA513	14	1.13E+03	chr3	38741489	38742762	+	2	circRNA	-	ENSOART00000021542	AAK1	mRNA
circRNA514	60	2.89E+03	chr3	38829991	38838191	+	3	circRNA	-	ENSOART00000021583	GFPT1	mRNA
circRNA515	17	6.63E+02	chr3	40003319	40005876	+	3	circRNA	-	ENSOART00000021676	ENSOARG00000019910	mRNA
circRNA516	14	9.76E+02	chr3	44276404	44277477	-	2	circRNA	-	ENSOART00000021839	UGP2	mRNA
circRNA517	9	4.60E+02	chr3	45282176	45285015	-	2	circRNA	-	ENSOART00000021893	EHBP1	mRNA
circRNA518	15	1.53E+03	chr3	57074346	57075453	+	2	circRNA	-	ENSOART00000022320	ELMOD3	mRNA
circRNA519	18	1.59E+03	chr3	57245293	57246551	-	2	circRNA	-	ENSOART00000022349	GGCX	mRNA
circRNA52	14	3.13E+02	chr12	30574689	30576797	+	5	circRNA	-	ENSOART00000005429	HNRNPU	mRNA
circRNA520	8	7.40E+02	chr3	57973784	57973961	-	1	circRNA	-	ENSOART00000022496	REEP1	mRNA
circRNA521	49	3.40E+03	chr3	58242986	58246666	-	2	circRNA	-	ENSOART00000022536	ENSOARG00000020684	mRNA
circRNA522	39	1.47E+03	chr3	58453063	58457016	+	3	circRNA	-	ENSOART00000022568	RMND5A	mRNA
circRNA523	69.5	3.18E+03	chr3	58652379	58657549	+	3	circRNA	-	ENSOART00000022605	SMYD1	mRNA
circRNA524	16.5	1.19E+03	chr3	58655255	58657549	+	2	circRNA	-	ENSOART00000022605	SMYD1	mRNA
circRNA525	62	2.05E+03	chr3	61885875	61889612	+	4	circRNA	-	ENSOART00000000474	ENSOARG00000000446	mRNA
circRNA526	40	1.90E+03	chr3	68927888	68931685	+	2	circRNA	-	ENSOART00000003137	RTN4	mRNA
circRNA527	48	2.15E+03	chr3	70202369	70209929	+	2	circRNA	-	ENSOART00000004457	ENSOARG00000004097	mRNA
circRNA528	93	2.49E+03	chr3	78243527	78252850	-	4	circRNA	-	ENSOART00000006370	PRKCE	mRNA
circRNA529	50	1.94E+03	chr3	78681918	78687800	+	3	circRNA	-	ENSOART00000006481	SRBD1	mRNA
circRNA53	61	3.98E+03	chr12	31727202	31734914	+	2	circRNA	-	ENSOART00000006609	AKT3	mRNA
circRNA530	247.6666667	1.40E+04	chr3	78723141	78787213	+	3	circRNA	-	ENSOART00000006481	SRBD1	mRNA
circRNA531	9.5	7.86E+02	chr3	78723141	78725703	+	2	circRNA	-	ENSOART00000006481	SRBD1	mRNA
circRNA532	731.1666667	1.80E+04	chr3	78725596	78857037	+	11	circRNA	-	ENSOART00000006481	SRBD1	mRNA
circRNA533	53	1.98E+03	chr3	87068714	87075543	+	3	circRNA	-	ENSOART00000010958	STRN	mRNA
circRNA534	18	7.21E+02	chr3	87381686	87383170	+	2	circRNA	-	ENSOART00000010997	FEZ2	mRNA
circRNA535	17	7.69E+02	chr3	87528277	87529699	-	2	circRNA	-	ENSOART00000011020	CRIM1	mRNA
circRNA536	87	4.87E+03	chr3	91821637	91831070	+	3	circRNA	-	ENSOART00000011804	ENSOARG00000010853	mRNA
circRNA537	37	2.64E+03	chr3	93345305	93347843	+	2	circRNA	-	ENSOART00000012517	DYSF	mRNA
circRNA538	41	1.83E+03	chr3	95450403	95457594	+	2	circRNA	-	ENSOART00000012930	ALMS1	mRNA

circRNA539	29	1.36E+03	chr3	97174538	97177207	+	2	circRNA	-	ENSOART00000014111	TGFBRAP1	mRNA
circRNA54	52	1.81E+03	chr12	39577785	39580801	-	3	circRNA	-	ENSOART00000017336	VPS13D	mRNA
circRNA540	87	3.03E+03	chr3	98998284	99005101	+	4	circRNA	-	ENSOART00000014168	MFSD9	mRNA
circRNA541	58	4.70E+03	chr3	99046852	99047054	-	1	circRNA	-	ENSOART00000014189	SLC9A2	mRNA
circRNA542	16	7.15E+02	chr3	100424898	100426572	-	2	circRNA	-	ENSOART00000014450	NPAS2	mRNA
circRNA543	8.5	7.44E+02	chr3	101797030	101798510	+	2	circRNA	-	ENSOART00000014544	REV1	mRNA
circRNA544	58.5	2.70E+03	chr3	101797030	101801889	+	3	circRNA	-	ENSOART00000014544	REV1	mRNA
circRNA545	174	1.04E+04	chr3	103595677	103596129	+	2	circRNA	-	ENSOART00000014963	ANKRD23	mRNA
circRNA546	20	1.19E+03	chr3	103788369	103788795	+	2	circRNA	-	ENSOART00000015103	KANSL3	mRNA
circRNA547	15	1.10E+03	chr3	112600309	112601183	-	2	circRNA	-	ENSOART00000016241	CSRP2	mRNA
circRNA548	13	9.64E+02	chr3	125721160	125722004	-	2	circRNA	-	ENSOART00000017026	POC1B	mRNA
circRNA549	26	1.57E+03	chr3	132863191	132864255	-	2	circRNA	-	ENSOART00000017901	TARBP2	mRNA
circRNA55	23	1.46E+03	chr12	39990300	39992692	-	3	circRNA	-	ENSOART00000019913	CLCN6	mRNA
circRNA550	59	1.04E+03	chr3	134951593	134956748	-	7	circRNA	-	ENSOART00000018967	LETMD1	mRNA
circRNA551	193	4.06E+03	chr3	135701318	135721269	-	7	circRNA	-	ENSOART00000019270	LARP4	mRNA
circRNA552	40	2.81E+03	chr3	136035402	136035635	-	1	circRNA	-	ENSOART00000019543	GPD1	mRNA
circRNA553	37	9.74E+02	chr3	137316983	137321553	+	4	circRNA	-	ENSOART00000020919	KANSL2	mRNA
circRNA554	10	8.35E+02	chr3	138695941	138697118	+	2	circRNA	-	ENSOART00000021185	RPAP3	mRNA
circRNA555	36	1.68E+03	chr3	140465818	140471413	-	2	circRNA	-	ENSOART00000021300	ARID2	mRNA
circRNA556	51	3.80E+03	chr3	140977372	140980594	-	2	circRNA	-	ENSOART00000021324	ANO6	mRNA
circRNA557	203	9.80E+03	chr3	144316403	144344118	-	3	circRNA	-	ENSOART00000021512	PPHLN1	mRNA
circRNA558	77	4.25E+03	chr3	144632758	144641615	+	2	circRNA	-	ENSOART00000021581	GXYLT1	mRNA
circRNA559	18	1.22E+03	chr3	149696120	149697791	-	2	circRNA	-	ENSOART00000022049	ENSOARG00000020251	mRNA
circRNA56	61	4.87E+03	chr12	41793376	41799428	+	2	circRNA	-	ENSOART00000007764	CLSTN1	mRNA
circRNA560	24	8.59E+02	chr3	150487617	150490935	-	3	circRNA	-	ENSOART00000022366	CPSF6	mRNA
circRNA561	59	2.78E+03	chr3	151073625	151077096	-	4	circRNA	-	ENSOART00000001109	RAP1B	mRNA
circRNA562	4	3.68E+02	chr3	154436164	154436342	-	1	circRNA	-	ENSOART00000003306	LEMD3	mRNA
circRNA563	106	2.74E+03	chr3	155548787	155567212	-	4	circRNA	-	ENSOART00000004259	SRGAP1	mRNA
circRNA564	28	1.49E+03	chr3	163486200	163486585	+	2	circRNA	-	ENSOART00000012425	ITGA7	mRNA
circRNA565	45	1.45E+03	chr3	167872315	167878304	+	4	circRNA	-	ENSOART00000014284	APAF-1	mRNA
circRNA566	65	3.57E+03	chr3	170498892	170500130	+	2	circRNA	-	ENSOART00000016451	MYBPC1	mRNA
circRNA567	64.5	2.65E+03	chr3	170502007	170504870	+	3	circRNA	-	ENSOART00000016451	MYBPC1	mRNA
circRNA568	165.5	4.83E+03	chr3	170506915	170514924	+	4	circRNA	-	ENSOART00000016451	MYBPC1	mRNA
circRNA569	175	6.36E+03	chr3	170523256	170530268	+	3	circRNA	-	ENSOART00000016451	MYBPC1	mRNA
circRNA57	138	7.37E+03	chr12	42986759	43003148	+	3	circRNA	-	ENSOART00000010545	RERE	mRNA
circRNA570	121	6.15E+03	chr3	170538793	170540807	+	2	circRNA	-	ENSOART00000016451	MYBPC1	mRNA
circRNA571	25	1.30E+03	chr3	173004362	173007347	+	3	circRNA	-	ENSOART00000018312	TXNRD1	mRNA
circRNA572	42	3.17E+03	chr3	174567837	174570985	-	2	circRNA	-	ENSOART00000018826	NUAK1	mRNA
circRNA573	397	2.21E+04	chr3	177116178	177160861	-	2	circRNA	-	ENSOART00000019889	LARGE	mRNA
circRNA574	59	4.47E+03	chr3	193063605	193069807	+	2	circRNA	-	ENSOART00000022021	ABCC9	mRNA
circRNA575	76	3.63E+03	chr3	193094142	193108355	+	3	circRNA	-	ENSOART00000022021	ABCC9	mRNA
circRNA576	21	1.32E+03	chr3	193504468	193506669	-	2	circRNA	-	ENSOART00000022176	PYROXD1	mRNA
circRNA577	60	3.48E+03	chr3	202201131	202206500	+	2	circRNA	-	ENSOART00000022723	DUSP16	mRNA
circRNA578	8	3.34E+02	chr3	202419143	202419536	+	1	circRNA	-	ENSOART00000022743	LRP6	mRNA
circRNA579	201	6.42E+03	chr3	203764340	203775034	+	6	circRNA	-	ENSOART00000022789	YBX3	mRNA
circRNA58	24	1.08E+03	chr12	49969690	49972664	-	2	circRNA	-	ENSOART00000010005	DDI2	mRNA
circRNA580	19	7.84E+02	chr3	207359718	207362048	-	4	circRNA	-	ENSOART00000005079	C1S	mRNA
circRNA581	14	8.35E+02	chr3	207812049	207812406	+	2	circRNA	-	ENSOART00000008438	IFFO1	mRNA
circRNA582	39	1.48E+03	chr3	208175913	208177461	+	3	circRNA	-	ENSOART00000009565	VWF	mRNA
circRNA583	44	2.10E+03	chr3	209442987	209446866	-	4	circRNA	-	ENSOART00000010273	ENSOARG00000009435	mRNA
circRNA584	318	1.22E+04	chr3	211897370	211932837	+	3	circRNA	-	ENSOART00000013668	ERC1	mRNA
circRNA585	24	1.13E+03	chr3	213213416	213214258	-	3	circRNA	-	ENSOART00000014814	MICAL3	mRNA
circRNA586	12	7.65E+02	chr3	213225798	213226560	-	2	circRNA	-	ENSOART00000014814	MICAL3	mRNA
circRNA587	24	1.48E+03	chr3	215682062	215682733	+	2	circRNA	-	ENSOART00000018835	ADSL	mRNA
circRNA588	20	1.25E+03	chr2	11127325	11128702	-	2	circRNA	-	ENSOART00000007088	HSDL2	mRNA
circRNA589	11	2.60E+02	chr2	13326868	13327563	-	1	circRNA	-	ENSOART00000007552	AKAP2	mRNA
circRNA59	73	3.56E+03	chr12	53639831	53649256	+	3	circRNA	-	ENSOART00000014378	RABGAP1L	mRNA
circRNA590	81	5.58E+03	chr2	13338433	13345874	-	3	circRNA	-	ENSOART00000007552	AKAP2	mRNA
circRNA591	21	8.53E+02	chr2	16078665	16080999	-	2	circRNA	-	ENSOART00000007848	ZNF462	mRNA
circRNA592	68	1.67E+03	chr2	18146461	18151995	+	6	circRNA	-	ENSOART00000008003	ABCA1	mRNA
circRNA593	37	1.85E+03	chr2	27958067	27961323	-	2	circRNA	-	ENSOART00000008767	FAM120A	mRNA
circRNA594	74	7.76E+03	chr2	37319269	37327793	+	2	circRNA	-	ENSOART00000010228	UBAP2	mRNA
circRNA595	12	6.72E+02	chr2	39937336	39938428	+	3	circRNA	-	ENSOART00000010752	KCTD9	mRNA
circRNA596	39	2.49E+03	chr2	42883770	42891024	-	2	circRNA	-	ENSOART00000011219	PPP3CC	mRNA
circRNA597	59	2.68E+03	chr2	48488217	48492585	-	3	circRNA	-	ENSOART00000011990	COL15A1	mRNA
circRNA598	20	1.27E+03	chr2	58573412	58576119	+	2	circRNA	-	ENSOART00000013473	GNAQ	mRNA
circRNA599	6	2.98E+02	chr2	68220290	68221257	+	2	circRNA	-	ENSOART00000014340	DOCK8	mRNA
circRNA6	67	3.60E+03	chrX	49720760	49721171	+	2	circRNA	-	ENSOART00000009744	KDM5C	mRNA
circRNA60	66	3.68E+03	chr12	55404306	55414729	-	3	circRNA	-	ENSOART00000015643	RFWD2	mRNA
circRNA600	34	1.85E+03	chr2	71062971	71067323	-	2	circRNA	-	ENSOART00000014535	RFX3	mRNA
circRNA601	348.5	1.94E+04	chr2	73270153	73310138	+	3	circRNA	-	ENSOART00000014740	RIC1	mRNA
circRNA602	197	1.11E+04	chr2	74230452	74250371	+	3	circRNA	-	ENSOART00000014915	ENSOARG00000013702	mRNA
circRNA603	13	8.73E+02	chr2	81121675	81124143	-	2	circRNA	-	ENSOART00000015100	MPDZ	mRNA
circRNA604	70	3.73E+03	chr2	82194281	82203397	-	3	circRNA	-	ENSOART00000015113	NFIB	mRNA
circRNA605	63	5.26E+02	chr2	84306748	84308717	-	1	circRNA	-	ENSOART00000015307	BNC2	mRNA
circRNA606	95	3.89E+03	chr2	102012666	102024180	+	3	circRNA	-	ENSOART00000016312	EXTL3	mRNA
circRNA607	10	7.22E+02	chr2	106274845	106275947	-	2	circRNA	-	ENSOART00000016760	GALNT7	mRNA
circRNA608	33	1.99E+03	chr2	110889448	110890954	-	2	circRNA	-	ENSOART00000016943	PALLD	mRNA
circRNA609	49	1.86E+03	chr2	111047116	111050379	-	3	circRNA	-	ENSOART00000016943	PALLD	mRNA
circRNA61	17	6.81E+02	chr12	62254484	62255015	+	2	circRNA	-	ENSOART00000020889	LAMC1	mRNA
circRNA610	83	2.11E+03	chr2	111526183	111538381	-	5	circRNA	-	ENSOART00000016971	ENSOARG00000015600	mRNA
circRNA611	40	1.83E+03	chr2	125497108	125503031	-	3	circRNA	-	ENSOART00000018216	NUP35	mRNA
circRNA612	9	3.48E+02	chr2	129550167	129551698	+	2	circRNA	-	ENSOART00000018437	ZNF385B	mRNA
circRNA613	21	7.35E+02	chr2	129854884	129857336	+	3	circRNA	-	ENSOART00000018462	SESTD1	mRNA
circRNA614	50	1.59E+03	chr2	129862227	129866959	+	4	circRNA	-	ENSOART00000018462	SESTD1	mRNA
circRNA615	41	2.32E+03	chr2	130209216	130210146	+	2	circRNA	-	ENSOART00000018808	TTN	mRNA
circRNA616	84	5.18E+03	chr2	130316982	130317668	+	3	circRNA	-	ENSOART00000018808	TTN	mRNA
circRNA617	53	2.97E+03	chr2	130323028	130324250	+	2	circRNA	-	ENSOART00000018808	TTN	mRNA
circRNA618	81	4.23E+03	chr2	130331267	130333778	+	4	circRNA	-	ENSOART00000018808	TTN	mRNA
circRNA619	46	3.00E+03	chr2	130342533	130343401	+	3	circRNA	-	ENSOART00000018808	TTN	mRNA
circRNA62	146	7.90E+03	chr12	63088372	63101384	-	3	circRNA	-	ENSOART00000002606	COLGALT2	mRNA
circRNA620	75	2.64E+03	chr2	130355567	130360167	+	6	circRNA	-	ENSOART00000018808	TTN	mRNA
circRNA621	46	3.84E+03	chr2	130387932	130388128	+	1	circRNA	-	ENSOART00000018808	TTN	mRNA
circRNA622	93	5.00E+03	chr2	130388209	130388514	+	1	circRNA	-	ENSOART00000018808	TTN	mRNA
circRNA623	91	4.67E+03	chr2	130412002	130412322	+	1	circRNA	-	ENSOART00000018808	TTN	mRNA
circRNA624	6	6.41E+02	chr2	130471451	130471604	+	1	circRNA	-	ENSOART00000018808	TTN	mRNA
circRNA625	56	1.68E+03	chr2	130568471	130576875	+	5	circRNA	-	ENSOART00000018858	PRKRA	mRNA
circRNA626	100	6.28E+03	chr2	135616303	135626785	-	3	circRNA	-	ENSOART00000000779	ENSOARG00000000725	mRNA

circRNA627	6	4.72E+02	chr2	137060335	137060543	-	1	circRNA	-	ENSOART00000002198	CYBRD1	mRNA
circRNA628	58	2.15E+03	chr2	137136964	137144454	-	4	circRNA	-	ENSOART00000002225	DCAF17	mRNA
circRNA629	57	1.90E+03	chr2	138576685	138586352	-	4	circRNA	-	ENSOART00000003042	UBR3	mRNA
circRNA63	49	2.72E+03	chr12	67382823	67386461	-	3	circRNA	-	ENSOART00000010026	ENSOARG00000009207	mRNA
circRNA630	35	2.06E+03	chr2	155840817	155846863	-	3	circRNA	-	ENSOART00000008999	FMNL2	mRNA
circRNA631	379	9.24E+03	chr2	155840817	155885603	-	7	circRNA	-	ENSOART00000008999	FMNL2	mRNA
circRNA632	26	1.61E+03	chr2	156267787	156270198	+	3	circRNA	-	ENSOART00000009094	STAM2	mRNA
circRNA633	28	1.44E+03	chr2	156824800	156826269	+	3	circRNA	-	ENSOART00000009464	NEB	mRNA
circRNA634	20	1.24E+03	chr2	160485987	160486438	-	2	circRNA	-	ENSOART00000010713	ACVR2A	mRNA
circRNA635	59	2.65E+03	chr2	174473174	174476999	-	2	circRNA	-	ENSOART00000012000	RAB3GAP1	mRNA
circRNA636	49	2.08E+03	chr2	184488928	184493138	+	2	circRNA	-	ENSOART00000014102	RALB	mRNA
circRNA637	120	2.40E+03	chr2	185627453	185641945	-	7	circRNA	-	ENSOART00000014394	CLASP1	mRNA
circRNA638	51	2.39E+03	chr2	185748154	185752456	-	4	circRNA	-	ENSOART00000014394	CLASP1	mRNA
circRNA639	71	3.33E+03	chr2	198954163	198961907	-	4	circRNA	-	ENSOART00000017019	BOLL	mRNA
circRNA64	10	8.15E+02	chr12	70866476	70866677	-	1	circRNA	-	ENSOART00000013293	HHAT	mRNA
circRNA640	59	2.23E+03	chr2	210512302	210522245	+	3	circRNA	-	ENSOART00000020838	ENSOARG00000019133	mRNA
circRNA641	14	8.53E+02	chr2	210512302	210516165	+	2	circRNA	-	ENSOART00000020838	ENSOARG00000019133	mRNA
circRNA642	22	9.50E+02	chr2	210817931	210819664	-	3	circRNA	-	ENSOART00000020884	ACADL	mRNA
circRNA643	40	1.88E+03	chr2	217652661	217653307	-	2	circRNA	-	ENSOART00000021151	IGFBP5	mRNA
circRNA644	30	1.54E+03	chr2	219581534	219583490	+	2	circRNA	-	ENSOART00000021375	PLCD4	mRNA
circRNA645	52	2.70E+03	chr2	230939295	230946217	-	3	circRNA	-	ENSOART00000022459	TRIP12	mRNA
circRNA646	19	8.76E+02	chr2	239446369	239447236	-	3	circRNA	-	ENSOART00000004784	RPS6KA1	mRNA
circRNA647	110	5.29E+03	chr2	239905371	239908744	+	2	circRNA	-	ENSOART00000005959	TRIM63	mRNA
circRNA648	39.5	1.81E+03	chr2	240135122	240138643	-	3	circRNA	-	ENSOART00000006323	MTFR1L	mRNA
circRNA649	5.5	3.63E+02	chr2	240137986	240138643	-	2	circRNA	-	ENSOART00000006323	MTFR1L	mRNA
circRNA65	98	6.48E+03	chr12	74684821	74696043	-	3	circRNA	-	ENSOART00000016698	DENND1B	mRNA
circRNA650	56	4.98E+03	chr2	241698982	241700668	+	2	circRNA	-	ENSOART00000007475	MYOM3	mRNA
circRNA651	78	1.62E+03	chr2	241712941	241717650	+	5	circRNA	-	ENSOART00000007475	MYOM3	mRNA
circRNA652	13	7.06E+02	chr2	242665880	242666511	-	2	circRNA	-	ENSOART00000008407	KDM1A	mRNA
circRNA653	16	3.32E+02	chr2	244462688	244463479	+	1	circRNA	-	ENSOART00000009489	EIF4G3	mRNA
circRNA654	20	1.49E+03	chr2	244579814	244581878	+	2	circRNA	-	ENSOART00000009489	EIF4G3	mRNA
circRNA655	10	6.23E+02	chr2	248487712	248488561	-	2	circRNA	-	ENSOART00000012809	CROCC	mRNA
circRNA656	30	1.29E+03	chr2	248658689	248661602	+	2	circRNA	-	ENSOART00000013210	FBXO42	mRNA
circRNA657	19	8.43E+02	chr1	235439	236760	+	3	circRNA	-	ENSOART00000019196	THAP4	mRNA
circRNA658	38	2.50E+03	chr1	2782846	2786648	-	3	circRNA	-	ENSOART00000020452	TRAF3IP1	mRNA
circRNA659	125	6.64E+03	chr1	3183562	3196296	-	3	circRNA	-	ENSOART00000020698	LRRFIP1	mRNA
circRNA66	39	2.33E+03	chr11	12051310	12055563	+	2	circRNA	-	ENSOART00000018671	APPBP2	mRNA
circRNA660	5	4.81E+02	chr1	3203874	3204044	-	1	circRNA	-	ENSOART00000020698	LRRFIP1	mRNA
circRNA661	338	1.77E+04	chr1	4967604	5004603	-	3	circRNA	-	ENSOART00000020837	AGAP1	mRNA
circRNA662	63	1.98E+03	chr1	7916983	7921293	-	4	circRNA	-	ENSOART00000021078	GIGYF2	mRNA
circRNA663	29	1.38E+03	chr1	10237507	10240349	-	2	circRNA	-	ENSOART00000021232	KIAA0319L	mRNA
circRNA664	56	2.64E+03	chr1	12515555	12516177	-	2	circRNA	-	ENSOART00000021721	FHL3	mRNA
circRNA665	42	1.92E+03	chr1	15358666	15362832	-	2	circRNA	-	ENSOART00000022161	SCMH1	mRNA
circRNA666	17	1.06E+03	chr1	17013189	17013896	-	3	circRNA	-	ENSOART00000022278	LEPRE1	mRNA
circRNA667	445	2.14E+04	chr1	18703746	18742496	-	4	circRNA	-	ENSOART00000000882	ERI3	mRNA
circRNA668	30	1.85E+03	chr1	20033539	20034896	-	2	circRNA	-	ENSOART00000002172	ENSOARG00000002014	mRNA
circRNA669	177.5	6.14E+03	chr1	33834229	33853171	+	3	circRNA	-	ENSOART00000009237	FGGY	mRNA
circRNA67	26	1.43E+03	chr11	12311384	12315336	+	2	circRNA	-	ENSOART00000019194	ENSOARG00000017636	mRNA
circRNA670	363.5	1.69E+04	chr1	33846699	33885518	+	3	circRNA	-	ENSOART00000009237	FGGY	mRNA
circRNA671	174	1.04E+04	chr1	36599484	36619902	-	3	circRNA	-	ENSOART00000009755	TM2D1	mRNA
circRNA672	62	4.91E+03	chr1	37809578	37816602	+	2	circRNA	-	ENSOART00000010401	ATG4C	mRNA
circRNA673	78	4.01E+03	chr1	38677406	38680533	+	2	circRNA	-	ENSOART00000010693	PGM1	mRNA
circRNA674	16	8.25E+02	chr1	39113404	39114381	+	2	circRNA	-	ENSOART00000010728	ROR1	mRNA
circRNA675	12	6.67E+02	chr1	65418525	65419799	+	2	circRNA	-	ENSOART00000016288	PKN2	mRNA
circRNA676	6	5.37E+02	chr1	69558549	69558732	-	1	circRNA	-	ENSOART00000018089	TMED5	mRNA
circRNA677	238	8.09E+03	chr1	71463521	71490453	+	5	circRNA	-	ENSOART00000019058	ENSOARG00000017506	mRNA
circRNA678	24	1.29E+03	chr1	76602590	76605509	+	3	circRNA	-	ENSOART00000019436	HIAT1	mRNA
circRNA679	45	2.24E+03	chr1	77413127	77421654	+	3	circRNA	-	ENSOART00000020019	SLC30A7	mRNA
circRNA68	5	3.05E+02	chr11	13091934	13092203	-	1	circRNA	-	ENSOART00000000909	ACACA	mRNA
circRNA680	14	8.20E+02	chr1	90229536	90230374	-	2	circRNA	-	ENSOART00000021633	RSBN1	mRNA
circRNA681	49	1.57E+03	chr1	91207832	91213153	-	3	circRNA	-	ENSOART00000021790	AMPD1	mRNA
circRNA682	16	8.31E+02	chr1	94467576	94469319	-	2	circRNA	-	ENSOART00000022125	GDAP2	mRNA
circRNA683	7	6.86E+02	chr1	95602521	95602688	-	1	circRNA	-	ENSOART00000022187	TBX15	mRNA
circRNA684	70	5.31E+03	chr1	96923238	96933943	+	2	circRNA	-	ENSOART00000022309	PDE4DIP	mRNA
circRNA685	172	6.29E+03	chr1	96923238	96939470	+	3	circRNA	-	ENSOART00000022309	PDE4DIP	mRNA
circRNA686	30	9.23E+02	chr1	99391645	99396387	+	5	circRNA	-	ENSOART00000022669	RPRD2	mRNA
circRNA687	25	9.25E+02	chr1	99991602	99992610	-	3	circRNA	-	ENSOART00000022828	VPS72	mRNA
circRNA688	32	2.45E+03	chr1	100122248	100123024	-	2	circRNA	-	ENSOART00000022860	PI4KB	mRNA
circRNA689	16	9.27E+02	chr1	100282087	100283344	-	2	circRNA	-	ENSOART00000022886	POGZ	mRNA
circRNA69	6	5.25E+02	chr11	14645365	14645552	+	1	circRNA	-	ENSOART00000008636	RFFL	mRNA
circRNA690	4	2.84E+02	chr1	100480326	100480557	+	1	circRNA	-	ENSOART00000022903	SNX27	mRNA
circRNA691	8	4.74E+02	chr1	111281469	111282769	+	2	circRNA	-	ENSOART00000011338	ATF6	mRNA
circRNA692	24	8.31E+02	chr1	118317445	118320724	+	3	circRNA	-	ENSOART00000013517	DCAF6	mRNA
circRNA693	27	8.42E+02	chr1	118358411	118360480	+	3	circRNA	-	ENSOART00000013517	DCAF6	mRNA
circRNA694	108.5	3.64E+03	chr1	118380002	118391396	+	4	circRNA	-	ENSOART00000013517	DCAF6	mRNA
circRNA695	7.5	5.02E+02	chr1	118382022	118382901	+	2	circRNA	-	ENSOART00000013517	DCAF6	mRNA
circRNA696	10	4.90E+02	chr1	120194083	120194611	-	2	circRNA	-	ENSOART00000014181	SON	mRNA
circRNA697	21	1.08E+03	chr1	120290672	120294239	+	3	circRNA	-	ENSOART00000014301	TMEM50B	mRNA
circRNA698	47	2.95E+03	chr1	125437971	125443220	-	3	circRNA	-	ENSOART00000016033	N6AMT1	mRNA
circRNA699	100	4.47E+03	chr1	128618521	128628562	+	3	circRNA	-	ENSOART00000016396	APP	mRNA
circRNA7	47	1.83E+03	chrX	61857602	61862683	-	3	circRNA	-	ENSOART00000019470	PHKA1	mRNA
circRNA70	48	2.43E+03	chr11	17350798	17354532	-	3	circRNA	-	ENSOART00000011402	PSMD11	mRNA
circRNA700	33	2.44E+03	chr1	139633065	139637907	-	2	circRNA	-	ENSOART00000017464	USP25	mRNA
circRNA701	4	2.63E+02	chr1	156826415	156826664	+	1	circRNA	-	ENSOART00000018874	ARL13B	mRNA
circRNA702	20	1.46E+03	chr1	163079654	163082264	+	2	circRNA	-	ENSOART00000019577	TBC1D23	mRNA
circRNA703	3	2.48E+02	chr1	170839965	170840163	+	1	circRNA	-	ENSOART00000020507	BBX	mRNA
circRNA704	49	2.55E+03	chr1	171897580	171904003	+	3	circRNA	-	ENSOART00000020696	DZIP3	mRNA
circRNA705	20	6.57E+02	chr1	171906336	171909255	+	4	circRNA	-	ENSOART00000020696	DZIP3	mRNA
circRNA706	40	9.38E+02	chr1	175487681	175493224	+	3	circRNA	-	ENSOART00000021015	CD200	mRNA
circRNA707	71	2.95E+03	chr1	185427283	185440201	+	2	circRNA	-	ENSOART00000021958	DIRC2	mRNA
circRNA708	141	7.61E+03	chr1	185448059	185463359	+	3	circRNA	-	ENSOART00000021958	DIRC2	mRNA
circRNA709	129	2.94E+03	chr1	188517557	188535534	-	8	circRNA	-	ENSOART00000022074	LRCH3	mRNA
circRNA71	34	1.97E+03	chr11	17792470	17796280	-	2	circRNA	-	ENSOART00000014150	SUZ12	mRNA
circRNA710	37	1.70E+03	chr1	189556628	189560278	+	4	circRNA	-	ENSOART00000022170	PAK2	mRNA
circRNA711	63	2.82E+03	chr1	189778172	189787217	-	4	circRNA	-	ENSOART00000022189	DLG1	mRNA
circRNA712	120	5.18E+03	chr1	189821879	189839939	-	3	circRNA	-	ENSOART00000022189	DLG1	mRNA
circRNA713	10	6.12E+02	chr1	192433723	192433991	-	1	circRNA	-	ENSOART00000022260	HRASLS	mRNA
circRNA714	23	1.19E+03	chr1	201490978	201494493	+	3	circRNA	-	ENSOART00000022484	PARL	mRNA

circRNA715	64	2.04E+03	chr1	202285897	202289728	+	3	circRNA	-	ENSOART00000022510	DCUN1D1	mRNA
circRNA716	83.5	2.96E+03	chr1	205555299	205560151	-	3	circRNA	-	ENSOART00000022541	USP13	mRNA
circRNA717	61.5	3.25E+03	chr1	205557188	205560151	-	2	circRNA	-	ENSOART00000022541	USP13	mRNA
circRNA718	4	3.34E+02	chr1	208169150	208169485	+	2	circRNA	-	ENSOART00000022572	TBL1XR1	mRNA
circRNA719	21	1.15E+03	chr1	213252029	213253558	-	2	circRNA	-	ENSOART00000022603	FNDC3B	mRNA
circRNA72	35	1.28E+03	chr11	17863988	17867825	+	4	circRNA	-	ENSOART00000014664	UTP6	mRNA
circRNA720	1018	5.74E+04	chr1	213353700	213466453	-	3	circRNA	-	ENSOART00000022603	FNDC3B	mRNA
circRNA721	174	9.12E+03	chr1	213905408	213925284	+	3	circRNA	-	ENSOART00000022613	PLD1	mRNA
circRNA722	33	1.63E+03	chr1	214354380	214359652	+	3	circRNA	-	ENSOART00000022619	TNIK	mRNA
circRNA723	21	1.16E+03	chr1	229869756	229873470	-	2	circRNA	-	ENSOART00000003422	GMPS	mRNA
circRNA724	8	5.40E+02	chr1	250318430	250318673	-	1	circRNA	-	ENSOART00000008488	NCK1	mRNA
circRNA725	79	4.39E+03	chr1	251237084	251244259	-	3	circRNA	-	ENSOART00000008751	PPP2R3A	mRNA
circRNA726	64	4.52E+03	chr1	251346081	251348437	-	2	circRNA	-	ENSOART00000008751	PPP2R3A	mRNA
circRNA727	82	4.35E+03	chr1	252783689	252790642	+	3	circRNA	-	ENSOART00000008870	KY	mRNA
circRNA728	3	1.82E+02	chr1	256949503	256949774	-	1	circRNA	-	ENSOART00000010131	NEK11	mRNA
circRNA729	181	1.20E+04	chr1	257667195	257691780	+	3	circRNA	-	ENSOART00000010634	SH3BGR	mRNA
circRNA73	11	5.59E+02	chr11	22889835	22890535	-	2	circRNA	-	ENSOART00000016419	SMG6	mRNA
circRNA730	46	2.91E+03	chr1	262756830	262759212	-	2	circRNA	-	ENSOART00000013319	UBE2G2	mRNA
circRNA731	15	4.07E+02	chr1	264099360	264102594	-	4	circRNA	-	ENSOART00000014250	MCM3AP	mRNA
circRNA732	16	5.38E+02	chr1	264234286	264236112	+	3	circRNA	-	ENSOART00000014436	PCNT	mRNA
circRNA733	22	1.11E+03	chr1	264471127	264472351	+	2	circRNA	-	ENSOART00000014653	PRMT2	mRNA
circRNA734	37	1.92E+03	chr1	266604470	266607548	+	3	circRNA	-	ENSOART00000015142	DOPEY2	mRNA
circRNA735	19	9.50E+02	chr1	267421116	267424249	+	3	circRNA	-	ENSOART00000015559	TTC3	mRNA
circRNA736	459	2.67E+04	chr1	267648536	267701580	+	2	circRNA	-	ENSOART00000015649	DYRK1A	mRNA
circRNA737	37	1.41E+03	chr1	267728440	267732487	+	2	circRNA	-	ENSOART00000015649	DYRK1A	mRNA
circRNA738	211	1.27E+04	chr1	275377953	275396191	+	2	circRNA	-	ENSOART00000017202	KAT2B	mRNA
circRNA739	173	6.44E+03	chr9	4760039	4782030	+	4	circRNA	-	ENSOART00000013017	LMBRD1	mRNA
circRNA74	16	1.04E+03	chr11	24768616	24769229	-	2	circRNA	-	ENSOART00000000950	XAF1	mRNA
circRNA740	11	8.15E+02	chr9	13543447	13543754	+	2	circRNA	-	ENSOART00000015170	FBXL6	mRNA
circRNA741	89	2.70E+03	chr9	15608764	15616239	-	4	circRNA	-	ENSOART00000003390	DENND3	mRNA
circRNA742	63	2.21E+03	chr9	15882223	15887761	+	4	circRNA	-	ENSOART00000003724	PTK2	mRNA
circRNA743	291	8.46E+03	chr9	32115985	32146693	-	3	circRNA	-	ENSOART00000012187	SPIDR	mRNA
circRNA744	56	1.79E+03	chr9	32333313	32342663	-	3	circRNA	-	ENSOART00000012972	PRKDC	mRNA
circRNA745	34	2.35E+03	chr9	35907687	35909654	+	2	circRNA	-	ENSOART00000015909	TGS1	mRNA
circRNA746	108	3.35E+03	chr9	44061481	44071823	+	5	circRNA	-	ENSOART00000000811	CSPP1	mRNA
circRNA747	26	1.80E+03	chr9	44906704	44910197	+	2	circRNA	-	ENSOART00000002721	PREX2	mRNA
circRNA748	22	1.18E+03	chr9	46290263	46292911	-	2	circRNA	-	ENSOART00000003798	SLCO5A1	mRNA
circRNA749	72	3.38E+03	chr9	46361048	46367632	-	2	circRNA	-	ENSOART00000003798	SLCO5A1	mRNA
circRNA75	45	1.53E+03	chr11	25814685	25820329	-	3	circRNA	-	ENSOART00000004326	RABEP1	mRNA
circRNA750	11	8.08E+02	chr9	47670868	47672528	-	2	circRNA	-	ENSOART00000004919	EYA1	mRNA
circRNA751	126	7.01E+03	chr9	49840150	49849649	-	2	circRNA	-	ENSOART00000006537	STAU2	mRNA
circRNA752	21	9.26E+02	chr9	56710274	56713189	-	2	circRNA	-	ENSOART00000009555	ZNF704	mRNA
circRNA753	109	3.71E+03	chr9	68900838	68917473	+	4	circRNA	-	ENSOART00000016096	EIF3E	mRNA
circRNA754	32	2.04E+03	chr9	77514153	77519443	-	2	circRNA	-	ENSOART00000002501	VPS13B	mRNA
circRNA755	107	3.01E+03	chr9	81892144	81907329	-	6	circRNA	-	ENSOART00000006439	NDUFAF6	mRNA
circRNA756	68	2.66E+03	chr9	82405460	82412278	+	4	circRNA	-	ENSOART00000008079	KIAA1429	mRNA
circRNA757	190	5.46E+03	chr9	85858451	85884967	-	5	circRNA	-	ENSOART00000010742	LRRC69	mRNA
circRNA758	69	2.41E+03	chr9	85872244	85884967	-	4	circRNA	-	ENSOART00000010742	LRRC69	mRNA
circRNA759	46	2.44E+03	chr9	86510710	86515005	-	2	circRNA	-	ENSOART00000012176	RIPK2	mRNA
circRNA76	20	8.57E+02	chr11	27176597	27177276	+	2	circRNA	-	ENSOART00000020012	CNTROB	mRNA
circRNA760	158	8.73E+03	chr8	2363076	2377219	+	3	circRNA	-	ENSOART00000007144	SENP6	mRNA
circRNA761	11	7.67E+02	chr8	2634673	2636165	+	2	circRNA	-	ENSOART00000007256	MYO6	mRNA
circRNA762	24.5	1.68E+03	chr8	14929289	14935594	+	2	circRNA	-	ENSOART00000008789	TRDN	mRNA
circRNA763	336.5	1.74E+04	chr8	14929289	14976153	+	3	circRNA	-	ENSOART00000008789	TRDN	mRNA
circRNA764	159	6.64E+03	chr8	20316819	20333497	-	4	circRNA	-	ENSOART00000009520	DCBLD1	mRNA
circRNA765	15	7.60E+02	chr8	29661284	29663672	-	2	circRNA	-	ENSOART00000011954	SOBP	mRNA
circRNA766	86.16666667	2.72E+03	chr8	32275644	32300159	+	5	circRNA	-	ENSOART00000012439	HACE1	mRNA
circRNA767	45.16666667	3.46E+03	chr8	32275644	32290362	+	2	circRNA	-	ENSOART00000012439	HACE1	mRNA
circRNA768	43.66666667	1.85E+03	chr8	32290280	32300159	+	4	circRNA	-	ENSOART00000012439	HACE1	mRNA
circRNA769	20	1.00E+03	chr8	37458928	37461592	+	2	circRNA	-	ENSOART00000012913	PNISR	mRNA
circRNA77	418	1.55E+04	chr11	29066024	29067275	-	3	circRNA	-	ENSOART00000013790	OMYHC2A	mRNA
circRNA770	7	4.52E+02	chr8	47391512	47391766	+	1	circRNA	-	ENSOART00000013438	BACH2	mRNA
circRNA771	30	1.53E+03	chr8	47582298	47584704	+	3	circRNA	-	ENSOART00000013649	MDN1	mRNA
circRNA772	34	1.29E+03	chr8	47645501	47646380	+	2	circRNA	-	ENSOART00000013649	MDN1	mRNA
circRNA773	25	1.11E+03	chr8	49788168	49791014	-	3	circRNA	-	ENSOART00000014272	ZNF292	mRNA
circRNA774	63	3.65E+03	chr8	54371404	54377973	+	2	circRNA	-	ENSOART00000014832	LAMA2	mRNA
circRNA775	17	4.10E+02	chr8	56233279	56234701	-	2	circRNA	-	ENSOART00000015107	EPB41L2	mRNA
circRNA776	27	3.31E+02	chr8	59206488	59207828	-	1	circRNA	-	ENSOART00000015984	SLC2A12	mRNA
circRNA777	10	4.09E+02	chr8	67875256	67875863	-	2	circRNA	-	ENSOART00000001708	FUCA2	mRNA
circRNA778	24	1.21E+03	chr8	68753799	68756231	+	2	circRNA	-	ENSOART00000002029	UTRN	mRNA
circRNA779	139	7.97E+03	chr8	68983876	69003863	+	2	circRNA	-	ENSOART00000002029	UTRN	mRNA
circRNA78	47	1.42E+03	chr11	35075217	35082570	+	4	circRNA	-	ENSOART00000003113	MBTD1	mRNA
circRNA780	2	1.22E+02	chr8	69018986	69019254	+	1	circRNA	-	ENSOART00000002029	UTRN	mRNA
circRNA781	8	3.99E+02	chr8	75897313	75897922	-	2	circRNA	-	ENSOART00000003875	SYNE1	mRNA
circRNA782	17	8.28E+02	chr8	75932244	75932581	-	1	circRNA	-	ENSOART00000003875	SYNE1	mRNA
circRNA783	338.5	1.61E+04	chr8	80106814	80181748	+	2	circRNA	-	ENSOART00000004627	ARID1B	mRNA
circRNA784	1264.5	4.18E+04	chr8	80106814	80311444	+	4	circRNA	-	ENSOART00000004627	ARID1B	mRNA
circRNA785	2	1.35E+02	chr8	80358153	80358396	+	1	circRNA	-	ENSOART00000004627	ARID1B	mRNA
circRNA786	26	1.52E+03	chr8	82909129	82910476	+	2	circRNA	-	ENSOART00000005063	IGF2R	mRNA
circRNA787	83.5	5.29E+03	chr8	85519445	85540411	+	2	circRNA	-	ENSOART00000005244	QKI	mRNA
circRNA788	399.5	1.63E+04	chr8	85519445	85585115	+	3	circRNA	-	ENSOART00000005244	QKI	mRNA
circRNA79	22	1.32E+03	chr11	41746720	41747200	-	2	circRNA	-	ENSOART00000020435	RAB5C	mRNA
circRNA8	85	3.45E+03	chrX	75521534	75530592	-	3	circRNA	-	ENSOART00000003608	F8	mRNA
circRNA80	46	1.94E+03	chr11	42067831	42071268	+	3	circRNA	-	ENSOART00000002045	ATP6V0A1	mRNA
circRNA81	19	1.28E+03	chr11	42837838	42839234	+	3	circRNA	-	ENSOART00000005902	DHX8	mRNA
circRNA82	81	5.46E+03	chr11	45482005	45493299	-	2	circRNA	-	ENSOART00000012441	KANSL1	mRNA
circRNA83	235	8.25E+03	chr11	47363437	47386038	+	4	circRNA	-	ENSOART00000014499	MAP3K3	mRNA
circRNA84	5	3.16E+02	chr11	47506194	47506557	-	2	circRNA	-	ENSOART00000015067	SMARCD2	mRNA
circRNA85	12	5.01E+02	chr11	48130276	48131461	+	3	circRNA	-	ENSOART00000016470	CEP95	mRNA
circRNA86	19	9.56E+02	chr11	50204249	50204758	+	2	circRNA	-	ENSOART00000019634	P4HB	mRNA
circRNA87	22	1.33E+03	chr11	50744824	50746057	-	3	circRNA	-	ENSOART00000000283	BAIAP2	mRNA
circRNA88	29	2.19E+03	chr11	54417060	54418064	+	2	circRNA	-	ENSOART00000008096	PRPSAP1	mRNA
circRNA89	24	2.18E+03	chr11	54605372	54607593	+	2	circRNA	-	ENSOART00000008969	SRP68	mRNA
circRNA9	27	1.22E+03	chrX	110881628	110885038	-	3	circRNA	-	ENSOART00000018300	WDR44	mRNA
circRNA90	239	1.26E+04	chr10	1970241	2006252	-	3	circRNA	-	ENSOART00000006793	TDRD3	mRNA
circRNA91	38.66666667	2.20E+03	chr10	11491039	11496390	-	5	circRNA	-	ENSOART00000007418	SUGT1	mRNA
circRNA92	35	2.09E+03	chr10	11535094	11536868	-	2	circRNA	-	ENSOART00000007455	ELF1	mRNA
circRNA93	291	8.60E+03	chr10	12272358	12310305	-	6	circRNA	-	ENSOART00000007813	VWA8	mRNA

circRNA94	89	6.15E+03	chr10	15660917	15670564	+	3	circRNA	-	ENSOART00000008283	GTF2F2	mRNA
circRNA95	28	1.15E+03	chr10	15915753	15919591	+	4	circRNA	-	ENSOART00000008296	ENSOARG00000007620	mRNA
circRNA96	44	3.06E+03	chr10	15972881	15976873	+	2	circRNA	-	ENSOART00000008352	COG3	mRNA
circRNA97	22	1.35E+03	chr10	18143993	18147238	-	2	circRNA	-	ENSOART00000008895	SUCLA2	mRNA
circRNA98	13	6.97E+02	chr10	23486091	23486683	-	2	circRNA	-	ENSOART00000010500	NHLRC3	mRNA
circRNA99	17	1.09E+03	chr10	25025452	25025708	+	1	circRNA	-	ENSOART00000011196	SMAD9	mRNA

Accession	flankIntron
ciRNA1	chrX:48268968-48271032
ciRNA10	chr12:39822959-39823212
ciRNA11	chr12:47543855-47544159
ciRNA12	chr12:78997024-78997281
ciRNA13	chr11:27344347-27344552
ciRNA14	chr11:27344347-27344552
ciRNA15	chr11:42169436-42169788
ciRNA16	chr11:42214581-42214933
ciRNA17	chr11:52919543-52920114
ciRNA18	chr11:55658153-55658332
ciRNA19	chr10:35877867-35879066
ciRNA2	chrX:75295840-75296426
ciRNA20	chr17:3874509-3875111
ciRNA21	chr17:12857186-12858244
ciRNA22	chr17:18022036-18024211
ciRNA23	chr17:71458674-71458929
ciRNA24	chr16:19315534-19409258
ciRNA25	chr16:62667372-62669972
ciRNA26	chr15:45861045-45861281
ciRNA27	chr14:14002171-14002393
ciRNA28	chr14:14079989-14080266
ciRNA29	chr14:14531171-14532380
ciRNA3	chrX:77136669-77136890
ciRNA30	chr19:10793500-10793912
ciRNA31	chr19:48938685-48938909
ciRNA32	chr19:48938685-48938909
ciRNA33	chr19:56030214-56030374
ciRNA34	chr19:58938906-58939197
ciRNA35	chr18:25775540-25776144
ciRNA36	chr26:35067642-35067959
ciRNA37	chr24:317914-318485
ciRNA38	chr24:2417852-2418092
ciRNA39	chr24:25935673-25935868
ciRNA4	chr13:3164177-3166186
ciRNA40	chr24:25935673-25935868
ciRNA41	chr24:25939078-25939310
ciRNA42	chr24:25956458-25956652
ciRNA43	chr22:32978555-32992696
ciRNA44	chr23:37600213-37603024
ciRNA45	chr23:41996777-41997012
ciRNA46	chr20:15159563-15160055
ciRNA47	chr20:19936361-19938120
ciRNA48	chr20:26411430-26411932
ciRNA49	chr21:17502675-17503899
ciRNA5	chr13:21240059-21243834
ciRNA50	chr21:41550296-41550500
ciRNA51	chr21:43628749-43629037
ciRNA52	chr21:44606058-44606985
ciRNA53	chr7:10786990-10787354
ciRNA54	chr7:21110570-21110828
ciRNA55	chr7:23233734-23233958
ciRNA56	chr7:34580686-34580938
ciRNA57	chr7:59629868-59630544
ciRNA58	chr6:17338580-17341715
ciRNA59	chr5:2425035-2425291
ciRNA6	chr13:56388588-56391293
ciRNA60	chr5:2446857-2447244
ciRNA61	chr5:5166939-5167916
ciRNA62	chr5:14069738-14070127
ciRNA63	chr5:17403398-17404181
ciRNA64	chr5:40632015-40632217
ciRNA65	chr5:46998364-46998582
ciRNA66	chr4:7207526-7264346
ciRNA67	chr4:92926797-92927596
ciRNA68	chr4:113210474-113210718
ciRNA69	chr4:118841483-118846160
ciRNA7	chr13:61858312-61859164
ciRNA70	chr3:7322298-7322523
ciRNA71	chr3:34019650-34020340
ciRNA72	chr3:34306176-34306776
ciRNA73	chr3:68319189-68323403
ciRNA74	chr3:132067790-132068429
ciRNA75	chr3:147041636-147144274
ciRNA76	chr3:163489519-163489731
ciRNA77	chr3:174853346-174861470
ciRNA78	chr3:205762392-205762643
ciRNA79	chr3:207729825-207730085
ciRNA8	chr13:65539418-65546091
ciRNA80	chr3:223455181-223455392
ciRNA81	chr2:130457972-130458450
ciRNA82	chr2:135806118-135807909
ciRNA83	chr2:154151534-154154925
ciRNA84	chr2:220534408-220534748
ciRNA85	chr1:20376960-20377212
ciRNA86	chr1:20376960-20377212
ciRNA87	chr1:20376960-20377212
ciRNA88	chr1:20382492-20382714
ciRNA89	chr1:20383623-20383826
ciRNA9	chr12:21634117-21635129
ciRNA90	chr1:20383623-20383826

ciRNA91	chr1:65575817-65576485
ciRNA92	chr1:102902644-102903156
ciRNA93	chr1:144679274-144680106
ciRNA94	chr1:182827633-182831255
ciRNA95	chr1:183376756-183380025
ciRNA96	chr9:14089128-14089701
ciRNA97	chr8:56468452-56522703
ciRNA98	chr8:56758183-56759890
circRNA1	chrX:1305509-1306178|chrX:1307128-1307974
circRNA10	chrX:115734756-115746979|chrX:115748116-115760118
circRNA100	chr10:26579923-26588679|None
circRNA101	chr10:28574583-28577656|chr10:28578297-28584926
circRNA102	chr10:28986999-28988123|chr10:28990004-28992073
circRNA103	chr10:28992091-28998513|chr10:29010704-29013637
circRNA104	chr10:29026947-29028872|chr10:29029476-29030667
circRNA105	chr10:29058037-29059909|chr10:29060791-29065605
circRNA106	chr10:33156046-33160169|chr10:33166307-33177291
circRNA107	chr10:33330976-33348341|chr10:33362755-33386414
circRNA108	chr10:34872386-34879963|chr10:34891970-34899128
circRNA109	chr10:51254858-51289501|chr10:51294905-51300567
circRNA11	chrX:126681770-126686131|chrX:126686773-126688054
circRNA110	chr10:54247861-54265129|chr10:54273188-54286004
circRNA111	chr10:83374208-83392024|chr10:83413889-83422856
circRNA112	chr10:84830199-84831773|chr10:84849824-84853854
circRNA113	chr10:84830199-84831773|chr10:84841892-84849701
circRNA114	chr17:223872-251789|chr17:302748-346457
circRNA115	chr17:9886191-9946519|chr17:9975542-10000920
circRNA116	chr17:9975542-10000920|chr17:10041116-10046212
circRNA117	chr17:10083605-10112049|chr17:10112280-10204496
circRNA118	chr17:12188885-12215856|chr17:12218594-12236814
circRNA119	chr17:12867789-12868841|chr17:12869231-12874396
circRNA12	chr13:9835597-9843121|chr13:9846344-9854023
circRNA120	chr17:12895948-12897607|chr17:12899192-12902169
circRNA121	chr17:16328316-16494897|chr17:16554002-16601167
circRNA122	chr17:17831262-17840468|chr17:17860245-17866142
circRNA123	chr17:17840598-17855203|chr17:17860245-17866142
circRNA124	chr17:18131991-18156330|chr17:18175228-18216339
circRNA125	chr17:39442242-39447899|chr17:39457401-39520809
circRNA126	chr17:39520827-39523184|chr17:39525287-39561576
circRNA127	None|chr17:43692203-43693499
circRNA128	chr17:50587889-50594842|chr17:50606607-50610528
circRNA129	chr17:51713764-51721650|chr17:51722653-51734757
circRNA13	chr13:11491256-11504059|chr13:11529530-11538162
circRNA130	chr17:51938691-51941610|chr17:51947424-51950671
circRNA131	chr17:52108388-52116135|chr17:52117521-52120540
circRNA132	chr17:52640404-52641255|chr17:52643362-52644795
circRNA133	chr17:56427131-56436265|chr17:56438821-56445838
circRNA134	chr17:57797217-57831025|chr17:57834416-57837882
circRNA135	chr17:63032667-63033631|chr17:63038809-63041154
circRNA136	chr17:63526534-63528090|chr17:63529089-63532833
circRNA137	chr17:63538093-63540203|chr17:63543573-63546207
circRNA138	chr17:63540513-63541643|chr17:63543573-63546207
circRNA139	chr17:63563772-63563878|chr17:63564029-63565536
circRNA14	chr13:11540165-11589922|None
circRNA140	chr17:65323246-65333291|chr17:65333794-65338044
circRNA141	chr17:68579412-68582122|chr17:68590897-68601513
circRNA142	chr17:72039421-72039846|chr17:72041111-72046781
circRNA143	chr16:3928087-3943581|chr16:3950906-3976580
circRNA144	chr16:6805337-6809973|chr16:6812240-6816019
circRNA145	chr16:6820348-6821859|chr16:6830388-6831589
circRNA146	chr16:7806184-7849852|chr16:7871261-7875147
circRNA147	chr16:8738334-8740811|chr16:8741379-8742704
circRNA148	chr16:12712628-12833433|chr16:12858238-13040674
circRNA149	chr16:16959840-16961506|chr16:16972094-16979478
circRNA15	chr13:11540165-11589922|chr13:11593536-11599093
circRNA150	chr16:32617504-32628764|chr16:32630659-32639529
circRNA151	chr16:35296681-35332035|chr16:35354339-35360143
circRNA152	chr16:37914328-37927592|chr16:37929630-37934358
circRNA153	chr16:39935379-39996062|chr16:39997596-40019769
circRNA154	chr16:40066809-40078744|chr16:40080785-40112622
circRNA155	chr16:58449135-58451215|chr16:58452723-58470847
circRNA156	chr16:58641689-58648813|chr16:58652333-58655800
circRNA157	chr16:62431013-62441359|chr16:62465984-62525040
circRNA158	chr16:62660512-62663566|chr16:62665716-62667234
circRNA159	chr16:65289665-65299098|chr16:65301617-65311892
circRNA16	chr13:15809704-15812919|chr13:15816515-15820312
circRNA160	chr16:65412491-65414164|chr16:65415221-65419349
circRNA161	chr16:65431138-65502309|chr16:65614661-65717374
circRNA162	chr15:14735288-14737901|chr15:14738909-14744992
circRNA163	chr15:17246378-17249707|chr15:17251492-17253501
circRNA164	chr15:35885440-35961418|chr15:35975386-36010417
circRNA165	chr15:37836422-37837711|chr15:37839399-37840817
circRNA166	chr15:39403860-39420670|chr15:39440282-39543819
circRNA167	chr15:39440282-39543819|chr15:39544075-39630751
circRNA168	chr15:40357711-40359934|chr15:40368447-40369380
circRNA169	chr15:42395585-42402235|chr15:42404844-42412458
circRNA17	chr13:16746218-16747679|chr13:16754570-16761282
circRNA170	chr15:43084004-43087771|chr15:43094889-43111224
circRNA171	chr15:44594015-44606308|chr15:44622789-44654158
circRNA172	chr15:45999323-46004428|chr15:46005272-46006278
circRNA173	chr15:49579803-49585997|chr15:49592805-49600630
circRNA174	chr15:50649034-50699503|chr15:50704639-50713411
circRNA175	chr15:71639255-71641100|chr15:71642106-71643243
circRNA176	chr15:74646026-74659081|chr15:74660186-74671407
circRNA177	chr15:74928674-74929192|chr15:74930689-74931881
circRNA178	chr15:78151302-78151533|chr15:78152483-78152743
circRNA179	chr14:7658469-7661147|chr14:7665709-7666687
circRNA18	chr13:17261269-17266555|chr13:17270110-17289955

circRNA180	chr14:12419149-12423678|chr14:12424493-12429414
circRNA181	chr14:14513089-14514910|None
circRNA182	chr14:15396906-15425775|chr14:15448081-15468345
circRNA183	chr14:21671251-21692645|chr14:21710921-21761521
circRNA184	chr14:23720082-23725371|chr14:23727408-23732772
circRNA185	chr14:34563917-34573460|None
circRNA186	chr14:35558563-35601263|chr14:35610399-35642255
circRNA187	chr14:42709425-42711140|chr14:42711890-42714497
circRNA188	None|chr14:44137891-44141337
circRNA189	chr14:44883988-44890346|chr14:44890578-44895693
circRNA19	chr13:17840795-17850442|chr13:17852392-17860768
circRNA190	chr14:45924991-45925096|chr14:45925432-45926890
circRNA191	chr14:47461882-47462819|chr14:47463744-47464385
circRNA192	chr14:47501250-47501767|chr14:47504888-47506450
circRNA193	chr14:52026502-52028516|chr14:52029204-52030228
circRNA194	chr14:53233576-53246130|chr14:53250332-53252571
circRNA195	chr14:53406644-53408301|chr14:53412751-53416343
circRNA196	chr14:54073039-54076292|chr14:54076819-54084042
circRNA197	chr14:54529116-54531500|chr14:54532042-54535828
circRNA198	chr14:54588874-54589698|chr14:54590252-54591287
circRNA199	chr14:55490969-55493495|chr14:55496140-55497568
circRNA2	chrX:7168655-7175475|chrX:7186650-7215319
circRNA20	chr13:17860919-17867371|chr13:17876443-17890489
circRNA200	chr14:56366892-56370505|chr14:56373586-56379136
circRNA201	chr14:60653354-60658580|chr14:60659008-60664948
circRNA202	chr19:10680671-10691553|chr19:10693241-10694170
circRNA203	chr19:10802542-10805227|chr19:10812086-10819039
circRNA204	chr19:13294342-13304346|chr19:13305123-13306270
circRNA205	chr19:15416918-15427732|None
circRNA206	chr19:16184196-16188823|chr19:16190730-16194231
circRNA207	chr19:21094460-21096551|chr19:21100383-21102327
circRNA208	chr19:21574025-21576534|chr19:21582094-21583238
circRNA209	chr19:21583304-21583981|chr19:21584431-21587191
circRNA21	chr13:24121046-24131471|chr13:24131755-24301564
circRNA210	chr19:34004669-34012035|chr19:34027397-34116866
circRNA211	chr19:35079290-35083860|chr19:35084181-35085485
circRNA212	chr19:35127035-35202827|chr19:35227047-35232619
circRNA213	chr19:35127035-35202827|chr19:35223555-35226890
circRNA214	chr19:43397010-43406044|chr19:43432970-43498429
circRNA215	chr19:43620677-43629121|chr19:43629777-43634852
circRNA216	chr19:43990878-43991190|chr19:43994885-43997365
circRNA217	chr19:47821713-47822587|chr19:47829830-47834828
circRNA218	chr19:47861815-47923692|chr19:47932306-47937471
circRNA219	chr19:48116946-48119068|chr19:48119849-48120854
circRNA22	chr13:29389092-29409257|chr13:29440886-29452850
circRNA220	chr19:48140398-48140672|chr19:48142894-48144394
circRNA221	chr19:48157065-48159025|chr19:48172212-48173623
circRNA222	chr19:50708103-50709988|chr19:50715778-50716364
circRNA223	chr19:53297701-53300743|chr19:53305972-53315808
circRNA224	chr19:53388676-53393082|chr19:53398900-53399937
circRNA225	chr19:53508407-53513270|chr19:53517602-53532274
circRNA226	chr19:53542349-53543332|chr19:53544085-53550206
circRNA227	None|chr19:54139069-54145142
circRNA228	None|chr19:54127538-54138944
circRNA229	chr19:55854020-55939600|chr19:55940535-55957265
circRNA23	chr13:31280529-31291294|chr13:31297994-31317917
circRNA230	chr19:57118112-57118729|chr19:57119820-57121809
circRNA231	chr19:57257628-57263365|chr19:57266919-57281112
circRNA232	chr19:57582959-57584039|chr19:57584467-57586102
circRNA233	chr19:59186391-59224896|chr19:59225941-59233877
circRNA234	chr18:5103696-5130911|chr18:5133227-5140544
circRNA235	chr18:6053041-6074185|chr18:6074377-6166066
circRNA236	None|chr18:6628646-6663766
circRNA237	chr18:13211999-13218976|chr18:13219223-13224320
circRNA238	chr18:13289794-13296367|chr18:13302172-13316361
circRNA239	chr18:16239649-16268033|chr18:16268242-16279025
circRNA24	chr13:33718945-33721111|chr13:33722660-33722963
circRNA240	chr18:16239649-16268033|chr18:16280779-16288192
circRNA241	None|chr18:19726561-19764687
circRNA242	chr18:20364218-20391863|chr18:20398803-20403358
circRNA243	chr18:20590656-20592975|chr18:20593445-20594293
circRNA244	chr18:20593445-20594293|chr18:20594689-20595158
circRNA245	chr18:21759494-21833525|chr18:21835176-21850555
circRNA246	chr18:22144258-22146430|chr18:22151056-22153037
circRNA247	chr18:31192851-31199496|chr18:31204593-31219021
circRNA248	chr18:31201037-31204077|chr18:31204593-31219021
circRNA249	chr18:33973833-34109003|None
circRNA25	chr13:37462816-37464848|chr13:37467374-37472106
circRNA250	chr18:40664846-40669343|chr18:40669834-40673571
circRNA251	chr18:40828532-40834658|chr18:40841439-40854522
circRNA252	chr18:41893639-41990450|chr18:42071988-42115639
circRNA253	chr18:44754948-44755700|chr18:44763617-44764341
circRNA254	chr18:48126173-48130469|chr18:48132328-48133764
circRNA255	chr18:56528615-56532743|chr18:56538046-56543549
circRNA256	chr18:60336586-60358444|chr18:60363401-60371710
circRNA257	chr18:66736018-66736431|chr18:66753040-66783134
circRNA258	chr18:66736018-66736431|chr18:66736623-66752986
circRNA259	chr18:67012894-67015979|chr18:67021903-67033771
circRNA26	chr13:42107363-42111565|chr13:42113242-42120034
circRNA260	chr18:68252784-68256539|chr18:68259331-68269511
circRNA261	chr26:1187603-1190345|chr26:1197933-1198635
circRNA262	chr26:6768337-6781131|chr26:6783170-6844240
circRNA263	chr26:7415630-7417084|chr26:7418202-7419239
circRNA264	chr26:18859550-18864723|chr26:18875180-18882521
circRNA265	chr26:22194833-22208317|chr26:22209465-22209698
circRNA266	chr26:22209465-22312259|chr26:22320214-22413176
circRNA267	chr26:23745945-23746080|chr26:23747677-23752226
circRNA268	chr26:25409960-25421022|chr26:25422828-25449509

circRNA269	chr26:27781268-27831504|chr26:27831799-27839470
circRNA27	chr13:42836704-42837054|chr13:42840114-42854488
circRNA270	chr26:31944749-31952951|chr26:31953606-31954436
circRNA271	chr26:32119457-32125521|chr26:32126214-32134321
circRNA272	None|chr26:32181898-32185172
circRNA273	chr26:32583273-32589680|chr26:32595801-32599293
circRNA274	chr26:32780490-32782003|chr26:32791906-32800324
circRNA275	chr26:32783096-32788236|chr26:32812140-32819091
circRNA276	chr26:37134775-37138727|chr26:37139123-37206498
circRNA277	chr26:37211587-37228704|chr26:37342527-37377077
circRNA278	chr26:38515868-38522268|chr26:38528263-38546099
circRNA279	chr24:243186-246958|chr24:250171-267787
circRNA28	chr13:46972412-46978109|chr13:46981351-46982841
circRNA280	chr24:3294006-3298550|chr24:3299406-3309105
circRNA281	chr24:10562069-10566199|chr24:10568909-10569675
circRNA282	chr24:10567215-10568510|chr24:10568909-10569675
circRNA283	chr24:10676211-10701840|chr24:10725440-10747169
circRNA284	chr24:10752652-10754615|chr24:10754991-10764964
circRNA285	chr24:15690252-15765058|chr24:15766630-15801871
circRNA286	chr24:15819344-15822397|chr24:15827152-15833900
circRNA287	chr24:16429899-16430651|chr24:16431240-16432777
circRNA288	chr24:16476319-16477406|chr24:16480580-16483820
circRNA289	chr24:19966231-19967579|chr24:19968014-19974214
circRNA29	chr13:46999393-46999889|chr13:47008442-47017172
circRNA290	chr24:20002815-20006107|chr24:20007527-20010585
circRNA291	chr24:20811094-20813369|chr24:20815893-20854244
circRNA292	chr24:22221325-22222816|chr24:22223369-22228646
circRNA293	chr24:25180196-25202323|chr24:25202591-25210603
circRNA294	chr24:25937485-25938946|chr24:25939793-25942328
circRNA295	chr24:25942495-25942933|chr24:25946810-25947717
circRNA296	chr24:27381617-27382678|chr24:27383843-27384478
circRNA297	chr24:34465430-34465659|chr24:34467010-34467037
circRNA298	chr24:34486854-34487116|chr24:34488392-34488894
circRNA299	chr24:38384560-38386718|chr24:38387438-38394164
circRNA3	chrX:14511413-14526430|chrX:14536166-14549081
circRNA30	chr13:47002093-47008318|chr13:47019299-47025437
circRNA300	chr24:39599851-39741516|chr24:39762828-39872447
circRNA301	chr25:4658127-4663120|chr25:4677035-4805551
circRNA302	chr25:6251800-6270278|chr25:6290921-6301106
circRNA303	chr25:9356700-9391626|chr25:9392743-9395834
circRNA304	chr25:9356700-9391626|chr25:9406699-9410746
circRNA305	chr25:14197462-14209595|chr25:14214854-14224357
circRNA306	chr25:14831199-14839141|chr25:14840864-14849448
circRNA307	chr25:14851425-14867928|chr25:14902606-14949986
circRNA308	chr25:17797019-17829285|chr25:17829511-17887009
circRNA309	chr25:23522818-23563599|None
circRNA31	chr13:50617014-50620902|chr13:50632647-50645112
circRNA310	chr25:24321401-24339532|chr25:24357551-24384886
circRNA311	chr25:24423457-24440020|chr25:24443215-24447283
circRNA312	chr25:25467605-25502893|chr25:25524953-25531266
circRNA313	chr25:28041891-28068315|chr25:28101367-28111194
circRNA314	chr25:42344867-42351178|chr25:42354662-42359083
circRNA315	chr22:257018-261459|chr22:265317-265577
circRNA316	chr22:6724516-6733642|chr22:6735823-6747501
circRNA317	chr22:6735823-6747501|chr22:6773314-6842739
circRNA318	chr22:13083427-13084910|chr22:13087599-13090035
circRNA319	chr22:13183627-13188862|chr22:13199361-13202109
circRNA32	chr13:51147482-51152013|chr13:51154493-51168113
circRNA320	chr22:13348191-13369227|chr22:13371219-13403200
circRNA321	chr22:14691523-14698925|chr22:14705211-14710996
circRNA322	chr22:16145489-16145804|chr22:16159821-16163841
circRNA323	chr22:16145879-16147677|chr22:16149498-16155746
circRNA324	chr22:21536317-21584851|chr22:21595148-21611424
circRNA325	chr22:21536317-21584851|chr22:21592062-21595052
circRNA326	chr22:21584946-21591310|chr22:21592062-21595052
circRNA327	chr22:21584946-21591310|chr22:21595148-21611424
circRNA328	chr22:21731813-21732003|chr22:21732517-21732884
circRNA329	chr22:31935232-31999187|chr22:32010639-32140061
circRNA33	chr13:51494557-51506899|chr13:51508527-51528469
circRNA330	chr22:33017152-33020718|chr22:33023088-33029426
circRNA331	chr22:33080036-33081796|chr22:33083387-33087598
circRNA332	chr22:38830274-38853744|chr22:38866754-38868367
circRNA333	None|chr22:40868489-40893691
circRNA334	chr22:40899638-40936899|chr22:40953529-41006249
circRNA335	chr22:41031078-41040542|chr22:41042685-41048659
circRNA336	chr22:45405443-45410285|chr22:45411717-45418061
circRNA337	chr22:45430184-45438452|chr22:45491775-45522626
circRNA338	chr23:32635254-32656397|chr23:32662441-32672430
circRNA339	chr23:32656509-32661095|chr23:32662441-32672430
circRNA34	chr13:51508527-51528469|None
circRNA340	chr23:32823216-32825798|chr23:32826612-32827632
circRNA341	chr23:32859306-32860806|chr23:32873765-32902229
circRNA342	chr23:33605785-33617942|chr23:33621601-33636224
circRNA343	chr23:34796736-34803383|chr23:34805489-34927995
circRNA344	chr23:34981377-34982390|chr23:34984997-34989005
circRNA345	chr23:37497835-37501057|chr23:37503192-37512475
circRNA346	chr23:43229183-43229456|chr23:43231633-43232851
circRNA347	chr23:43276552-43282768|chr23:43284768-43314250
circRNA348	chr23:49146505-49192996|chr23:49194555-49221839
circRNA349	chr23:54443618-54445912|chr23:54447893-54453229
circRNA35	chr13:57010974-57012343|chr13:57015563-57016166
circRNA350	chr23:54498549-54505184|chr23:54507212-54508438
circRNA351	chr23:54848909-54858760|chr23:54863253-54937154
circRNA352	chr23:58196573-58208175|chr23:58212690-58219600
circRNA353	None|chr20:3103180-3113486
circRNA354	None|chr20:3116872-3118637
circRNA355	chr20:6355634-6379245|chr20:6418787-6429379
circRNA356	chr20:9707096-9733121|chr20:9750912-9766436

circRNA357	chr20:11008346-11010703|chr20:11011610-11013346
circRNA358	chr20:19273921-19282510|chr20:19286771-19345755
circRNA359	chr20:23852000-23865152|chr20:23876616-23881827
circRNA36	chr13:59834186-59836452|chr13:59849556-59853156
circRNA360	chr20:23876616-23881827|chr20:23882876-23894560
circRNA361	chr20:32215334-32222597|chr20:32223113-32231334
circRNA362	chr20:38828839-38871273|chr20:38905373-38931243
circRNA363	chr20:39786846-39803458|chr20:39807129-39819201
circRNA364	chr20:39786846-39803458|chr20:39819375-39824250
circRNA365	chr20:48543545-48568915|chr20:48569592-48569631
circRNA366	chr21:16876188-16880800|chr21:16881391-16887018
circRNA367	chr21:16876188-16880800|chr21:16893825-16938091
circRNA368	chr21:17699528-17701927|chr21:17714326-17714954
circRNA369	chr21:28081667-28095124|chr21:28096716-28114527
circRNA37	chr13:60969121-60973767|chr13:60977600-60979400
circRNA370	chr21:31622598-31634321|chr21:31639191-31650189
circRNA371	None|chr21:35470928-35475719
circRNA372	chr21:39763925-39767053|chr21:39769052-39774519
circRNA373	chr21:45318885-45320691|chr21:45326050-45330384
circRNA374	chr21:49409061-49411884|chr21:49414425-49415182
circRNA375	chr21:49825841-49826410|chr21:49827573-49828045
circRNA376	chr7:10161155-10180186|chr7:10185131-10226342
circRNA377	chr7:10185131-10226342|chr7:10245400-10293530
circRNA378	chr7:10226482-10235059|chr7:10245400-10293530
circRNA379	chr7:10504545-10505869|chr7:10519525-10522819
circRNA38	chr13:65527827-65532711|chr13:65546143-65550297
circRNA380	chr7:11890106-11895571|chr7:11897186-11902322
circRNA381	chr7:12110908-12114102|chr7:12115610-12122345
circRNA382	chr7:12163211-12170334|chr7:12172421-12175209
circRNA383	chr7:13842288-13858208|chr7:13859053-13859097
circRNA384	chr7:17368807-17370347|chr7:17379430-17384201
circRNA385	chr7:21203535-21204457|chr7:21204609-21204691
circRNA386	chr7:34700817-34703908|chr7:34715052-34720406
circRNA387	chr7:35122946-35126418|chr7:35134381-35160220
circRNA388	chr7:35434914-35438998|chr7:35441681-35446191
circRNA389	chr7:35441681-35446191|chr7:35447845-35450836
circRNA39	chr13:72487359-72488969|chr13:72489885-72496299
circRNA390	chr7:39806012-39808904|chr7:39810673-39817015
circRNA391	chr7:42526581-42641886|None
circRNA392	chr7:42921951-42922576|chr7:42923952-42926401
circRNA393	chr7:43396811-43397801|chr7:43398957-43400275
circRNA394	chr7:48124823-48125998|chr7:48127213-48129897
circRNA395	chr7:50638014-50657544|chr7:50660702-50672430
circRNA396	chr7:54777361-54779730|chr7:54783630-54784694
circRNA397	chr7:56740804-56743052|chr7:56751082-56751698
circRNA398	chr7:57961518-58013693|chr7:58028055-58041098
circRNA399	chr7:64991701-64994908|chr7:65003314-65007752
circRNA4	chrX:38487686-38488318|chrX:38489668-38489838
circRNA40	chr13:75350909-75352547|chr13:75353846-75356440
circRNA400	chr7:69269944-69272807|chr7:69275655-69286205
circRNA401	chr7:73509791-73511257|chr7:73511927-73514421
circRNA402	chr7:85457499-85458333|chr7:85460286-85462043
circRNA403	chr7:85642389-85650490|chr7:85652906-85680673
circRNA404	chr7:97005582-97010440|chr7:97013958-97023881
circRNA405	chr7:99051717-99075742|chr7:99079247-99085930
circRNA406	chr7:99091391-99111007|chr7:99128569-99167191
circRNA407	chr7:99114534-99121746|chr7:99128569-99167191
circRNA408	chr6:6700220-6731652|chr6:6735075-6743112
circRNA409	chr6:12405541-12409082|chr6:12412260-12418935
circRNA41	chr13:76711693-76712742|chr13:76713690-76715034
circRNA410	chr6:17328451-17333784|chr6:17348913-17370050
circRNA411	chr6:17333857-17338490|chr6:17341842-17346382
circRNA412	chr6:17341842-17346382|chr6:17348913-17370050
circRNA413	chr6:35920432-35926598|chr6:35926785-35948815
circRNA414	chr6:44715937-44737719|chr6:44738468-44746885
circRNA415	chr6:59105762-59111747|chr6:59117863-59126291
circRNA416	chr6:59682065-59737975|chr6:59740606-59779466
circRNA417	chr6:60516710-60517199|chr6:60523268-60528651
circRNA418	chr6:66158278-66173029|chr6:66175656-66183188
circRNA419	chr6:66308156-66319019|chr6:66320396-66323579
circRNA42	chr13:76821311-76832966|None
circRNA420	chr6:67841803-67849933|chr6:67850143-67850510
circRNA421	None|chr6:88784788-88808569
circRNA422	chr6:90259519-90271558|chr6:90276893-90282543
circRNA423	chr6:90297101-90297564|chr6:90300360-90301470
circRNA424	chr6:92091138-92105003|chr6:92111142-92161028
circRNA425	chr6:92862410-92866345|chr6:92871211-92872576
circRNA426	chr6:92878914-92880897|chr6:92881112-92884461
circRNA427	chr6:101191889-101203863|chr6:101204430-101220046
circRNA428	chr5:2428741-2429060|chr5:2429324-2429651
circRNA429	chr5:2428741-2429060|chr5:2429915-2430448
circRNA43	chr13:77702716-77704878|chr13:77706265-77717475
circRNA430	chr5:2428741-2429060|chr5:2437306-2439275
circRNA431	chr5:2469694-2469819|chr5:2558987-2589441
circRNA432	chr5:2479998-2486266|chr5:2558987-2589441
circRNA433	chr5:2492214-2495953|chr5:2558987-2589441
circRNA434	chr5:2496229-2501233|chr5:2558987-2589441
circRNA435	chr5:2538575-2554863|chr5:2558987-2589441
circRNA436	chr5:6347413-6350959|chr5:6360478-6368152
circRNA437	chr5:9398308-9399661|chr5:9400083-9401662
circRNA438	chr5:9804252-9805230|chr5:9809105-9812857
circRNA439	chr5:12632713-12657271|chr5:12665631-12667649
circRNA44	chr12:1561222-1564408|chr12:1572324-1574019
circRNA440	chr5:13761843-13768274|chr5:13770421-13775994
circRNA441	chr5:13789032-13856977|chr5:13857529-13878484
circRNA442	chr5:16273368-16276758|chr5:16277104-16286484
circRNA443	chr5:19157285-19169710|chr5:19186266-19209168
circRNA444	chr5:23352369-23356558|chr5:23359010-23363279

circRNA445	chr5:23365897-23377337|chr5:23379191-23404667
circRNA446	chr5:23951584-23959367|chr5:23964802-23967278
circRNA447	chr5:24468725-24565603|chr5:24568974-24599416
circRNA448	chr5:26493385-26536837|chr5:26537093-26586123
circRNA449	chr5:27824038-27831027|chr5:27832892-27837236
circRNA45	chr12:19992439-20017553|chr12:20021404-20025581
circRNA450	chr5:27824038-27831027|chr5:27837378-27864926
circRNA451	chr5:31867220-31873576|chr5:31878007-31886638
circRNA452	chr5:40764055-40764127|chr5:40764302-40764424
circRNA453	chr5:44919841-44946023|chr5:44946595-44958432
circRNA454	chr5:46729830-46732312|chr5:46733578-46734829
circRNA455	chr5:46804531-46812314|chr5:46815135-46834405
circRNA456	chr5:47016380-47018159|chr5:47019057-47020421
circRNA457	chr5:51190813-51316655|chr5:51328883-51335729
circRNA458	chr5:54627954-54693025|chr5:54693295-54697085
circRNA459	chr5:56064485-56068028|chr5:56069775-56071717
circRNA46	chr12:22364232-22372185|chr12:22373858-22383286
circRNA460	chr5:58077471-58092190|chr5:58115168-58131119
circRNA461	chr5:58688484-58692114|chr5:58697744-58698902
circRNA462	chr5:59350015-59358237|chr5:59361664-59377376
circRNA463	chr5:59844357-59846755|chr5:59850143-59851330
circRNA464	chr5:60521445-60524713|chr5:60529139-60529946
circRNA465	chr5:65099467-65272814|chr5:65356197-65401047
circRNA466	chr5:67652166-67701160|chr5:67726882-67754338
circRNA467	chr5:77930559-77937108|chr5:77940020-77945074
circRNA468	chr5:85618399-85636227|chr5:85638570-85648632
circRNA469	chr5:93732117-93736411|chr5:93738186-93750370
circRNA47	chr12:25521418-25521971|chr12:25539415-25552845
circRNA470	None|chr5:99388658-99391540
circRNA471	chr5:106082721-106090072|chr5:106098828-106126627
circRNA472	chr4:13406332-13421012|chr4:13425462-13426463
circRNA473	chr4:27333587-27354277|chr4:27354541-27426417
circRNA474	chr4:27440769-27458209|chr4:27501097-27541641
circRNA475	chr4:44436540-44450596|chr4:44450903-44482250
circRNA476	chr4:51577672-51584121|chr4:51594142-51624279
circRNA477	chr4:56152763-56350155|chr4:56378779-56401988
circRNA478	chr4:68458752-68469460|chr4:68488380-68551661
circRNA479	chr4:68488380-68551661|chr4:68560460-68569154
circRNA48	chr12:25564655-25566747|chr12:25573148-25578440
circRNA480	chr4:70752857-70757604|chr4:70759054-70760532
circRNA481	chr4:77332663-77337380|chr4:77341631-77346793
circRNA482	chr4:85317170-85333878|chr4:85366206-85381044
circRNA483	chr4:85599851-85629859|chr4:85632140-85665375
circRNA484	chr4:95077111-95095030|chr4:95110907-95129306
circRNA485	chr4:96584761-96664574|chr4:96703243-96749785
circRNA486	chr4:106159959-106161865|chr4:106162478-106166276
circRNA487	chr4:113955785-113958413|chr4:113960958-113969260
circRNA488	chr4:114104718-114135276|chr4:114140110-114145955
circRNA489	chr4:114526951-114538606|chr4:114539539-114551821
circRNA49	chr12:25630178-25635144|chr12:25637667-25640991
circRNA490	chr4:116780658-116785834|chr4:116811876-116816071
circRNA491	chr4:116788390-116806186|chr4:116811876-116816071
circRNA492	chr4:117643506-117670495|None
circRNA493	chr4:117670599-117689457|None
circRNA494	chr4:117876479-117897054|chr4:117905907-117911108
circRNA495	None|chr4:118049973-118057985
circRNA496	chr3:299426-302329|chr3:310088-317116
circRNA497	chr3:310088-317116|chr3:320335-321924
circRNA498	chr3:1222780-1225363|chr3:1227933-1234048
circRNA499	chr3:3394779-3408915|chr3:3410064-3417364
circRNA5	chrX:49391726-49392544|chrX:49393713-49395012
circRNA50	chr12:28251768-28263772|chr12:28270484-28283365
circRNA500	chr3:3892753-3899764|chr3:3905347-3907502
circRNA501	chr3:3952581-3955639|chr3:3974132-4001792
circRNA502	chr3:5501656-5502599|chr3:5504857-5506586
circRNA503	chr3:7272353-7279338|chr3:7281560-7283036
circRNA504	chr3:10373730-10443948|chr3:10459257-10473468
circRNA505	chr3:10648706-10650297|chr3:10650872-10652858
circRNA506	chr3:10727867-10730654|chr3:10734228-10739392
circRNA507	chr3:23595598-23607490|chr3:23623874-23633089
circRNA508	chr3:23745497-23749228|chr3:23761853-23768194
circRNA509	chr3:32543518-32567102|chr3:32573785-32588191
circRNA51	chr12:28251768-28263772|chr12:28267902-28270283
circRNA510	chr3:33299582-33300225|chr3:33303213-33307801
circRNA511	chr3:35872593-35876029|chr3:35884332-35893705
circRNA512	chr3:35872593-35876029|chr3:35879658-35884267
circRNA513	chr3:38730248-38741488|chr3:38742762-38744149
circRNA514	None|chr3:38838191-38845737
circRNA515	chr3:39986049-40003318|chr3:40005876-40010602
circRNA516	chr3:44251129-44276403|chr3:44277477-44295862
circRNA517	chr3:45259352-45282175|chr3:45285015-45349131
circRNA518	chr3:57070506-57074345|chr3:57075453-57077335
circRNA519	chr3:57244374-57245292|chr3:57246551-57248921
circRNA52	chr12:30572769-30574688|chr12:30576797-30577116
circRNA520	chr3:57968910-57973783|chr3:57973961-57996507
circRNA521	chr3:58241572-58242985|chr3:58246666-58259589
circRNA522	chr3:58440434-58453062|chr3:58457016-58459341
circRNA523	chr3:58650473-58652378|chr3:58657549-58668120
circRNA524	chr3:58652509-58655254|chr3:58657549-58668120
circRNA525	chr3:61818702-61885874|chr3:61889612-61891066
circRNA526	chr3:68895275-68927887|chr3:68931685-68942542
circRNA527	chr3:70179536-70202368|chr3:70209929-70240740
circRNA528	chr3:78164577-78243526|chr3:78252850-78266466
circRNA529	chr3:78675297-78681917|chr3:78687800-78691749
circRNA53	chr12:31703451-31727201|chr12:31734914-31745322
circRNA530	chr3:78719556-78723140|chr3:78787213-78796420
circRNA531	chr3:78719556-78723140|chr3:78725703-78787121
circRNA532	chr3:78723231-78725595|chr3:78857037-78862705

circRNA533	chr3:87064686-87068713|chr3:87075543-87078868
circRNA534	chr3:87379363-87381685|chr3:87383170-87402529
circRNA535	chr3:87505673-87528276|chr3:87529699-87580168
circRNA536	chr3:91809760-91821636|chr3:91831070-91851350
circRNA537	chr3:93339292-93345304|chr3:93347843-93355584
circRNA538	chr3:95436615-95450402|chr3:95457594-95470046
circRNA539	chr3:97174341-97174537|chr3:97177207-97182655
circRNA54	chr12:39576761-39577784|chr12:39580801-39581901
circRNA540	chr3:98995437-98998283|chr3:99005101-99006670
circRNA541	chr3:99036940-99046851|chr3:99047054-99048456
circRNA542	chr3:100424029-100424897|chr3:100426572-100432437
circRNA543	chr3:101771846-101797029|chr3:101798510-101801720
circRNA544	chr3:101771846-101797029|chr3:101801889-101811389
circRNA545	chr3:103595481-103595676|chr3:103596129-103597337
circRNA546	chr3:103780098-103788368|chr3:103788795-103795382
circRNA547	chr3:112599286-112600308|chr3:112601183-112605534
circRNA548	chr3:125703641-125721159|chr3:125722004-125724650
circRNA549	chr3:132862598-132863190|chr3:132864255-132864660
circRNA55	chr12:39989278-39990299|chr12:39992692-39993827
circRNA550	chr3:134950432-134951592|chr3:134956748-134957579
circRNA551	chr3:135690687-135701317|chr3:135721269-135722007
circRNA552	chr3:136035173-136035401|chr3:136035635-136036304
circRNA553	chr3:137315988-137316982|chr3:137321553-137322472
circRNA554	chr3:138694787-138695940|chr3:138697118-138698642
circRNA555	chr3:140462413-140465817|chr3:140471413-140584042
circRNA556	chr3:140970961-140977371|chr3:140980594-140988849
circRNA557	chr3:144235549-144316402|chr3:144344118-144346894
circRNA558	chr3:144623586-144632757|chr3:144641615-144645440
circRNA559	chr3:149691771-149696119|chr3:149697791-149701149
circRNA56	chr12:41793254-41793375|chr12:41799428-41880541
circRNA560	chr3:150487536-150487616|chr3:150490935-150493293
circRNA561	chr3:151071642-151073624|None
circRNA562	chr3:154431770-154436163|chr3:154436342-154437007
circRNA563	chr3:155531612-155548786|chr3:155567212-155594952
circRNA564	chr3:163483723-163486199|chr3:163486585-163489405
circRNA565	chr3:167868026-167872314|chr3:167878304-167887061
circRNA566	chr3:170493022-170498891|chr3:170500130-170502006
circRNA567	chr3:170500130-170502006|chr3:170504870-170506914
circRNA568	chr3:170504870-170506914|chr3:170514924-170516786
circRNA569	chr3:170518800-170523255|chr3:170530268-170534742
circRNA57	chr12:42938375-42986758|chr12:43003148-43033773
circRNA570	chr3:170537797-170538792|chr3:170540807-170542627
circRNA571	chr3:172982755-173004361|chr3:173007347-173016385
circRNA572	chr3:174557090-174567836|chr3:174570985-174588859
circRNA573	chr3:176998520-177116177|chr3:177160861-177179642
circRNA574	chr3:193051244-193063604|chr3:193069807-193081523
circRNA575	chr3:193092639-193094141|chr3:193108355-193117780
circRNA576	chr3:193503304-193504467|chr3:193506669-193507484
circRNA577	chr3:202184665-202201130|chr3:202206500-202210022
circRNA578	chr3:202400703-202419142|chr3:202419536-202473966
circRNA579	chr3:203761503-203764339|chr3:203775034-203778218
circRNA58	chr12:49967909-49969689|chr12:49972664-49981028
circRNA580	chr3:207357504-207359717|chr3:207362048-207362729
circRNA581	chr3:207810111-207812048|chr3:207812406-207812688
circRNA582	chr3:208168864-208175912|chr3:208177461-208196017
circRNA583	chr3:209441047-209442986|chr3:209446866-209449789
circRNA584	chr3:211883414-211897369|chr3:211932837-211985706
circRNA585	chr3:213202870-213213415|chr3:213214258-213215565
circRNA586	chr3:213224914-213225797|chr3:213226560-213226974
circRNA587	chr3:215681245-215682061|chr3:215682733-215684614
circRNA588	chr2:11120840-11127324|chr2:11128702-11129146
circRNA589	chr2:13262731-13326867|chr2:13327563-13338432
circRNA59	chr12:53607078-53639830|chr12:53649256-53676423
circRNA590	chr2:13327563-13338432|chr2:13345874-13393576
circRNA591	chr2:16068406-16078664|chr2:16080999-16115556
circRNA592	chr2:18144376-18146460|chr2:18151995-18153345
circRNA593	chr2:27941246-27958066|chr2:27961323-27984534
circRNA594	chr2:37317459-37319268|chr2:37327793-37331208
circRNA595	chr2:39926774-39937335|chr2:39938428-39968804
circRNA596	chr2:42880916-42883769|chr2:42891024-42897630
circRNA597	chr2:48487220-48488216|chr2:48492585-48493312
circRNA598	chr2:58553581-58573411|chr2:58576119-58647327
circRNA599	chr2:68217194-68220289|chr2:68221257-68223981
circRNA6	chrX:49720026-49720759|chrX:49721171-49722210
circRNA60	chr12:55388256-55404305|chr12:55414729-55424169
circRNA600	chr2:71048934-71062970|chr2:71067323-71076577
circRNA601	chr2:73223408-73270152|chr2:73310138-73318845
circRNA602	chr2:74212349-74230451|chr2:74250371-74345699
circRNA603	chr2:81120840-81121674|chr2:81124143-81127629
circRNA604	chr2:82169183-82194280|chr2:82203397-82246991
circRNA605	chr2:84290999-84306747|chr2:84308717-84428529
circRNA606	chr2:102000260-102012665|chr2:102024180-102034239
circRNA607	chr2:106270852-106274844|chr2:106275947-106278170
circRNA608	chr2:110886628-110889447|chr2:110890954-110891982
circRNA609	chr2:110895160-111047115|chr2:111050379-111068068
circRNA61	chr12:62251984-62254483|chr12:62255015-62261452
circRNA610	chr2:111513335-111526182|chr2:111538381-111553676
circRNA611	chr2:125483522-125497107|chr2:125503031-125508662
circRNA612	chr2:129518907-129550166|chr2:129551698-129552732
circRNA613	chr2:129847038-129854883|chr2:129857336-129862226
circRNA614	chr2:129857336-129862226|chr2:129866959-129869489
circRNA615	chr2:130208637-130209215|chr2:130210146-130211610
circRNA616	chr2:130316837-130316981|chr2:130317668-130318101
circRNA617	chr2:130322735-130323027|chr2:130324250-130324458
circRNA618	chr2:130330489-130331266|chr2:130333778-130333931
circRNA619	chr2:130342421-130342532|chr2:130343401-130343948
circRNA62	chr12:63081881-63088371|chr12:63101384-63107970
circRNA620	chr2:130354095-130355566|chr2:130360167-130362172

circRNA621	chr2:130387843-130387931|chr2:130388128-130388208
circRNA622	chr2:130388128-130388208|chr2:130388514-130388609
circRNA623	chr2:130411674-130412001|chr2:130412322-130412417
circRNA624	chr2:130471358-130471450|chr2:130471604-130472138
circRNA625	chr2:130565154-130568470|chr2:130576875-130579265
circRNA626	chr2:135610055-135616302|chr2:135626785-135689555
circRNA627	chr2:137047182-137060334|None
circRNA628	chr2:137133288-137136963|chr2:137144454-137145692
circRNA629	chr2:138564257-138576684|chr2:138586352-138624598
circRNA63	chr12:67377326-67382822|chr12:67386461-67396676
circRNA630	chr2:155816776-155840816|chr2:155846863-155866627
circRNA631	chr2:155816776-155840816|chr2:155885603-155914820
circRNA632	chr2:156259488-156267786|chr2:156270198-156271993
circRNA633	chr2:156820462-156824799|chr2:156826269-156827790
circRNA634	chr2:160471147-160485986|chr2:160486438-160492940
circRNA635	chr2:174464293-174473173|chr2:174476999-174488770
circRNA636	chr2:184481150-184488927|chr2:184493138-184497025
circRNA637	chr2:185617578-185627452|chr2:185641945-185656032
circRNA638	chr2:185739297-185748153|chr2:185752456-185810155
circRNA639	chr2:198942643-198954162|chr2:198961907-198964803
circRNA64	chr12:70819200-70866475|chr12:70866677-70906144
circRNA640	chr2:210507258-210512301|chr2:210522245-210528656
circRNA641	chr2:210507258-210512301|chr2:210516165-210522079
circRNA642	chr2:210804944-210817930|chr2:210819664-210825502
circRNA643	chr2:217650907-217652660|chr2:217653307-217668848
circRNA644	chr2:219580723-219581533|chr2:219583490-219584259
circRNA645	chr2:230925601-230939294|chr2:230946217-230948870
circRNA646	chr2:239445151-239446368|chr2:239447236-239454382
circRNA647	chr2:239904257-239905370|chr2:239908744-239909742
circRNA648	chr2:240134691-240135121|chr2:240138643-240140502
circRNA649	chr2:240135231-240137985|chr2:240138643-240140502
circRNA65	chr12:74673184-74684820|chr12:74696043-74713226
circRNA650	chr2:241693832-241698981|chr2:241700668-241702841
circRNA651	chr2:241710201-241712940|chr2:241717650-241723531
circRNA652	chr2:242664286-242665879|chr2:242666511-242668403
circRNA653	None|chr2:244463479-244501537
circRNA654	chr2:244572280-244579813|chr2:244581878-244599983
circRNA655	chr2:248482689-248487711|chr2:248488561-248489108
circRNA656	chr2:248638653-248658688|chr2:248661602-248671716
circRNA657	chr1:216930-235438|chr1:236760-249031
circRNA658	chr1:2776609-2782845|chr1:2786648-2788793
circRNA659	chr1:3182500-3183561|chr1:3196296-3200104
circRNA66	chr11:12049107-12051309|chr11:12055563-12068804
circRNA660	chr1:3200197-3203873|chr1:3204044-3207322
circRNA661	chr1:4909753-4967603|chr1:5004603-5024258
circRNA662	chr1:7912258-7916982|chr1:7921293-7922671
circRNA663	chr1:10232191-10237506|chr1:10240349-10263654
circRNA664	chr1:12514742-12515554|chr1:12516177-12516889
circRNA665	chr1:15338436-15358665|chr1:15362832-15414157
circRNA666	chr1:17011291-17013188|chr1:17013896-17018812
circRNA667	chr1:18669647-18703745|chr1:18742496-18762832
circRNA668	chr1:20026754-20033538|chr1:20034896-20049217
circRNA669	None|chr1:33853171-33885429
circRNA67	chr11:12308146-12311383|chr11:12315336-12317327
circRNA670	chr1:33834440-33846698|chr1:33885518-33997996
circRNA671	chr1:36588143-36599483|chr1:36619902-36630096
circRNA672	chr1:37807565-37809577|chr1:37816602-37821544
circRNA673	chr1:38666365-38677405|chr1:38680533-38683420
circRNA674	chr1:39059167-39113403|chr1:39114381-39212936
circRNA675	chr1:65399109-65418524|chr1:65419799-65430567
circRNA676	chr1:69556183-69558548|chr1:69558732-69564452
circRNA677	chr1:71440608-71463520|chr1:71490453-71503615
circRNA678	chr1:76592098-76602589|chr1:76605509-76608044
circRNA679	chr1:77404638-77413126|chr1:77421654-77461546
circRNA68	chr11:13085241-13091933|chr11:13092203-13109564
circRNA680	chr1:90222009-90229535|chr1:90230374-90247535
circRNA681	chr1:91204964-91207831|chr1:91213153-91217653
circRNA682	chr1:94455906-94467575|chr1:94469319-94472738
circRNA683	chr1:95589683-95602520|chr1:95602688-95603785
circRNA684	chr1:96921861-96923237|chr1:96933943-96939237
circRNA685	chr1:96921861-96923237|chr1:96939470-96942549
circRNA686	chr1:99372993-99391644|chr1:99396387-99409856
circRNA687	chr1:99985726-99991601|chr1:99992610-99996298
circRNA688	chr1:100121668-100122247|chr1:100123024-100124572
circRNA689	chr1:100272196-100282086|None
circRNA69	chr11:14592049-14645364|chr11:14645552-14654220
circRNA690	chr1:100454078-100480325|chr1:100480557-100500109
circRNA691	chr1:111266353-111281468|chr1:111282769-111311814
circRNA692	chr1:118310489-118317444|chr1:118320724-118332072
circRNA693	chr1:118353795-118358410|chr1:118360480-118365428
circRNA694	chr1:118365470-118380001|chr1:118391396-118399536
circRNA695	chr1:118380140-118382021|chr1:118382901-118391290
circRNA696	chr1:120186984-120194082|chr1:120194611-120196534
circRNA697	chr1:120269606-120290671|chr1:120294239-120303164
circRNA698	chr1:125430563-125437970|chr1:125443220-125445910
circRNA699	chr1:128605403-128618520|chr1:128628562-128651479
circRNA7	chrX:61855824-61857601|chrX:61862683-61885829
circRNA70	chr11:17346717-17350797|chr11:17354532-17361044
circRNA700	chr1:139626549-139633064|chr1:139637907-139643572
circRNA701	chr1:156816140-156826414|chr1:156826664-156856648
circRNA702	chr1:163076731-163079653|chr1:163082264-163083861
circRNA703	chr1:170830202-170839964|chr1:170840163-170843821
circRNA704	chr1:171895747-171897579|chr1:171904003-171906335
circRNA705	chr1:171904003-171906335|chr1:171909255-171912705
circRNA706	chr1:175484429-175487680|chr1:175493224-175498486
circRNA707	chr1:185405418-185427282|chr1:185440201-185448058
circRNA708	chr1:185440201-185448058|chr1:185463359-185482681
circRNA709	chr1:188506418-188517556|chr1:188535534-188542844

circRNA71	chr11:17790064-17792469|chr11:17796280-17800446
circRNA710	chr1:189555163-189556627|chr1:189560278-189561733
circRNA711	chr1:189769463-189778171|chr1:189787217-189793248
circRNA712	chr1:189805546-189821878|chr1:189839939-189850397
circRNA713	chr1:192424458-192433722|chr1:192433991-192439533
circRNA714	chr1:201471853-201490977|chr1:201494493-201509191
circRNA715	None|chr1:202289728-202299480
circRNA716	chr1:205533629-205555298|chr1:205560151-205563587
circRNA717	chr1:205555452-205557187|chr1:205560151-205563587
circRNA718	chr1:208160743-208169149|chr1:208169485-208173341
circRNA719	chr1:213249986-213252028|chr1:213253558-213267573
circRNA72	chr11:17863399-17863987|chr11:17867825-17868801
circRNA720	chr1:213335235-213353699|None
circRNA721	chr1:213903436-213905407|chr1:213925284-213940773
circRNA722	chr1:214339094-214354379|chr1:214359652-214371875
circRNA723	chr1:229862857-229869755|chr1:229873470-229873483
circRNA724	chr1:250304919-250318429|chr1:250318673-250415095
circRNA725	chr1:251185935-251237083|chr1:251244259-251247973
circRNA726	chr1:251333292-251346080|None
circRNA727	chr1:252780274-252783688|chr1:252790642-252800036
circRNA728	chr1:256887304-256949502|chr1:256949774-256951193
circRNA729	chr1:257655722-257667194|chr1:257691780-257706020
circRNA73	chr11:22858403-22889834|chr11:22890535-22897642
circRNA730	chr1:262753661-262756829|chr1:262759212-262768801
circRNA731	chr1:264098224-264099359|chr1:264102594-264104756
circRNA732	chr1:264229978-264234285|chr1:264236112-264242231
circRNA733	chr1:264467490-264471126|chr1:264472351-264479676
circRNA734	chr1:266601763-266604469|chr1:266607548-266614298
circRNA735	chr1:267411973-267421115|chr1:267424249-267427630
circRNA736	None|chr1:267701580-267706459
circRNA737	chr1:267720948-267728439|chr1:267732487-267740117
circRNA738	chr1:275377716-275377952|chr1:275396191-275400364
circRNA739	chr9:4744444-4760038|chr9:4782030-4794279
circRNA74	chr11:24766505-24768615|chr11:24769229-24769986
circRNA740	chr9:13543372-13543446|chr9:13543754-13543822
circRNA741	chr9:15604035-15608763|chr9:15616239-15618991
circRNA742	chr9:15881860-15882222|chr9:15887761-15892642
circRNA743	chr9:32064370-32115984|chr9:32146693-32202192
circRNA744	chr9:32331923-32333312|chr9:32342663-32344622
circRNA745	chr9:35905160-35907686|chr9:35909654-35910215
circRNA746	chr9:44051729-44061480|chr9:44071823-44074183
circRNA747	chr9:44896118-44906703|chr9:44910197-44911657
circRNA748	chr9:46288031-46290262|chr9:46292911-46295345
circRNA749	chr9:46345298-46361047|chr9:46367632-46444741
circRNA75	chr11:25808019-25814684|chr11:25820329-25835067
circRNA750	chr9:47633189-47670867|chr9:47672528-47705920
circRNA751	chr9:49782379-49840149|chr9:49849649-49860225
circRNA752	chr9:56699432-56710273|chr9:56713189-56721490
circRNA753	chr9:68899450-68900837|chr9:68917473-68918483
circRNA754	chr9:77489797-77514152|chr9:77519443-77620453
circRNA755	chr9:81883144-81892143|chr9:81907329-81917193
circRNA756	chr9:82392486-82405459|chr9:82412278-82418044
circRNA757	chr9:85849865-85858450|chr9:85884967-85912024
circRNA758	chr9:85858552-85872243|chr9:85884967-85912024
circRNA759	chr9:86505649-86510709|chr9:86515005-86524575
circRNA76	chr11:27175408-27176596|chr11:27177276-27179948
circRNA760	None|chr8:2377219-2381144
circRNA761	chr8:2631060-2634672|chr8:2636165-2641564
circRNA762	chr8:14919862-14929288|chr8:14935594-14976075
circRNA763	chr8:14919862-14929288|chr8:14976153-15000208
circRNA764	chr8:20311946-20316818|chr8:20333497-20351535
circRNA765	chr8:29632473-29661283|chr8:29663672-29677385
circRNA766	chr8:32269555-32275643|chr8:32300159-32308905
circRNA767	chr8:32269555-32275643|chr8:32290362-32298089
circRNA768	chr8:32275775-32290279|chr8:32300159-32308905
circRNA769	chr8:37457790-37458927|chr8:37461592-37463245
circRNA77	chr11:29065310-29066023|chr11:29067275-29068372
circRNA770	chr8:47368512-47391511|chr8:47391766-47455865
circRNA771	chr8:47580624-47582297|chr8:47584704-47590857
circRNA772	chr8:47644210-47645500|chr8:47646380-47647155
circRNA773	chr8:49772451-49788167|None
circRNA774	None|chr8:54377973-54420530
circRNA775	chr8:56210504-56233278|None
circRNA776	chr8:59185168-59206487|chr8:59207828-59234725
circRNA777	chr8:67872703-67875255|chr8:67875863-67877435
circRNA778	chr8:68753144-68753798|chr8:68756231-68757393
circRNA779	chr8:68969402-68983875|chr8:69003863-69018985
circRNA78	chr11:35042086-35075216|chr11:35082570-35092658
circRNA780	chr8:69003863-69018985|chr8:69019254-69027619
circRNA781	chr8:75888119-75897312|chr8:75897922-75905042
circRNA782	chr8:75931589-75932243|chr8:75932581-75933957
circRNA783	None|chr8:80181748-80215118
circRNA784	None|chr8:80311444-80358152
circRNA785	chr8:80311444-80358152|chr8:80358396-80386442
circRNA786	chr8:82907567-82909128|chr8:82910476-82911254
circRNA787	None|chr8:85540411-85584971
circRNA788	None|chr8:85585115-85613415
circRNA79	chr11:41745650-41746719|chr11:41747200-41748617
circRNA8	chrX:75517939-75521533|chrX:75530592-75534171
circRNA80	chr11:42065994-42067830|chr11:42071268-42073497
circRNA81	chr11:42833535-42837837|chr11:42839234-42840458
circRNA82	chr11:45474066-45482004|chr11:45493299-45562423
circRNA83	chr11:47358131-47363436|chr11:47386038-47389724
circRNA84	chr11:47506034-47506193|chr11:47506557-47506704
circRNA85	chr11:48128672-48130275|chr11:48131461-48134156
circRNA86	chr11:50204061-50204248|chr11:50204758-50204876
circRNA87	chr11:50736188-50744823|chr11:50746057-50767409
circRNA88	chr11:54412914-54417059|chr11:54418064-54418921

circRNA89	chr11:54603530-54605371|chr11:54607593-54610505
circRNA9	chrX:110879712-110881627|chrX:110885038-110885804
circRNA90	chr10:1961432-1970240|chr10:2006252-2065280
circRNA91	chr10:11489391-11491038|chr10:11496390-11498041
circRNA92	chr10:11527492-11535093|chr10:11536868-11543649
circRNA93	chr10:12245692-12272357|chr10:12310305-12313892
circRNA94	chr10:15648142-15660916|chr10:15670564-15718996
circRNA95	chr10:15913662-15915752|chr10:15919591-15921826
circRNA96	chr10:15969021-15972880|chr10:15976873-15981373
circRNA97	chr10:18135322-18143992|chr10:18147238-18168379
circRNA98	chr10:23483695-23486090|chr10:23486683-23487083
circRNA99	[illegible]5525-25025451|chr10:2502570[illegible]

Accession	gene Description
ciRNA1	Uncharacterized protein [Source:UniProtKB/TrEMBL;Acc:W5P8A5]
ciRNA10	mitofusin 2 [Source:HGNC Symbol;Acc:HGNC:16877]
ciRNA11	WD repeat containing, antisense to TP73 [Source:HGNC Symbol;Acc:HGNC:12759]
ciRNA12	arginyl aminopeptidase (aminopeptidase B) [Source:HGNC Symbol;Acc:HGNC:10078]
ciRNA13	Uncharacterized protein [Source:UniProtKB/TrEMBL;Acc:W5QC67]
ciRNA14	Uncharacterized protein [Source:UniProtKB/TrEMBL;Acc:W5QC67]
ciRNA15	Tubulin gamma chain [Source:UniProtKB/TrEMBL;Acc:W5NXF6]
ciRNA16	tubulin, gamma 2 [Source:HGNC Symbol;Acc:HGNC:12419]
ciRNA17	Uncharacterized protein [Source:UniProtKB/TrEMBL;Acc:W5P5U0]
ciRNA18	RAB37, member RAS oncogene family [Source:HGNC Symbol;Acc:HGNC:30268]
ciRNA19	large tumor suppressor kinase 2 [Source:HGNC Symbol;Acc:HGNC:6515]
ciRNA2	GRB2-associated binding protein 3 [Source:HGNC Symbol;Acc:HGNC:17515]
ciRNA20	KIAA0922 [Source:HGNC Symbol;Acc:HGNC:29146]
ciRNA21	OTU deubiquitinase 4 [Source:HGNC Symbol;Acc:HGNC:24949]
ciRNA22	N(alpha)-acetyltransferase 15, NatA auxiliary subunit [Source:HGNC Symbol;Acc:HGNC:30782]
ciRNA23	phosphatidylinositol 4-kinase, catalytic, alpha [Source:HGNC Symbol;Acc:HGNC:8983]
ciRNA24	Uncharacterized protein [Source:UniProtKB/TrEMBL;Acc:W5PBA3]
ciRNA25	NA
ciRNA26	TAF10 RNA polymerase II, TATA box binding protein (TBP)-associated factor, 30kDa [Source:HGNC Symbol;Acc:HGNC:11543]
ciRNA27	spastic paraplegia 7 (pure and complicated autosomal recessive) [Source:HGNC Symbol;Acc:HGNC:11237]
ciRNA28	charged multivesicular body protein 1A [Source:HGNC Symbol;Acc:HGNC:8740]
ciRNA29	origin recognition complex, subunit 6 [Source:HGNC Symbol;Acc:HGNC:17151]
ciRNA3	ubiquitin-like 4A [Source:HGNC Symbol;Acc:HGNC:12505]
ciRNA30	golgin A4 [Source:HGNC Symbol;Acc:HGNC:4427]
ciRNA31	Uncharacterized protein [Source:UniProtKB/TrEMBL;Acc:W5P9G1]
ciRNA32	Uncharacterized protein [Source:UniProtKB/TrEMBL;Acc:W5P9G1]
ciRNA33	plexin D1 [Source:HGNC Symbol;Acc:HGNC:9107]
ciRNA34	coatomer protein complex, subunit gamma 1 [Source:HGNC Symbol;Acc:HGNC:2236]
ciRNA35	cell migration inducing protein, hyaluronan binding [Source:HGNC Symbol;Acc:HGNC:29213]
ciRNA36	ankyrin 1, erythrocytic [Source:HGNC Symbol;Acc:HGNC:492]
ciRNA37	2,4-dienoyl CoA reductase 2, peroxisomal [Source:HGNC Symbol;Acc:HGNC:2754]
ciRNA38	host cell factor C1 regulator 1 (XPO1 dependent) [Source:HGNC Symbol;Acc:HGNC:21198]
ciRNA39	ATPase, Ca++ transporting, cardiac muscle, fast twitch 1 [Source:HGNC Symbol;Acc:HGNC:811]
ciRNA4	McKusick-Kaufman syndrome [Source:HGNC Symbol;Acc:HGNC:7108]
ciRNA40	ATPase, Ca++ transporting, cardiac muscle, fast twitch 1 [Source:HGNC Symbol;Acc:HGNC:811]
ciRNA41	ATPase, Ca++ transporting, cardiac muscle, fast twitch 1 [Source:HGNC Symbol;Acc:HGNC:811]
ciRNA42	SH2B adaptor protein 1 [Source:HGNC Symbol;Acc:HGNC:30417]
ciRNA43	hyaluronan binding protein 2 [Source:HGNC Symbol;Acc:HGNC:4798]
ciRNA44	myomesin 1 [Source:HGNC Symbol;Acc:HGNC:7613]
ciRNA45	ralA binding protein 1 [Source:HGNC Symbol;Acc:HGNC:9841]
ciRNA46	triggering receptor expressed on myeloid cells-like 1 [Source:HGNC Symbol;Acc:HGNC:20434]
ciRNA47	solute carrier family 25, member 27 [Source:HGNC Symbol;Acc:HGNC:21065]
ciRNA48	tenascin XB [Source:HGNC Symbol;Acc:HGNC:11976]
ciRNA49	integrator complex subunit 4 [Source:HGNC Symbol;Acc:HGNC:25048]
ciRNA5	nebulette [Source:HGNC Symbol;Acc:HGNC:16932]
ciRNA50	MAP/microtubule affinity-regulating kinase 2 [Source:HGNC Symbol;Acc:HGNC:3332]
ciRNA51	RAB1B, member RAS oncogene family [Source:HGNC Symbol;Acc:HGNC:18370]
ciRNA52	ribosomal protein S6 kinase, 70kDa, polypeptide 2 [Source:HGNC Symbol;Acc:HGNC:10437]
ciRNA53	thrombospondin 4 [Source:HGNC Symbol;Acc:HGNC:11788]
ciRNA54	Uncharacterized protein [Source:UniProtKB/TrEMBL;Acc:W5QDF3]
ciRNA55	Rho guanine nucleotide exchange factor (GEF) 40 [Source:HGNC Symbol;Acc:HGNC:25516]
ciRNA56	vacuolar protein sorting 39 homolog (S. cerevisiae) [Source:HGNC Symbol;Acc:HGNC:20593]
ciRNA57	sema domain, transmembrane domain (TM), and cytoplasmic domain, (semaphorin) 6D [Source:HGNC Symbol;Acc:HGNC:16770]
ciRNA58	hydroxyacyl-CoA dehydrogenase [Source:HGNC Symbol;Acc:HGNC:4799]
ciRNA59	Uncharacterized protein [Source:UniProtKB/TrEMBL;Acc:W5P620]
ciRNA6	endothelin 3 [Source:HGNC Symbol;Acc:HGNC:3178]
ciRNA60	Uncharacterized protein [Source:UniProtKB/TrEMBL;Acc:W5P620]
ciRNA61	microtubule-associated protein 1S [Source:HGNC Symbol;Acc:HGNC:15715]
ciRNA62	mucolipin 1 [Source:HGNC Symbol;Acc:HGNC:13356]
ciRNA63	ankyrin repeat domain 24 [Source:HGNC Symbol;Acc:HGNC:29424]
ciRNA64	polymerase (RNA) mitochondrial (DNA directed) [Source:HGNC Symbol;Acc:HGNC:9200]
ciRNA65	kinesin family member 20A [Source:HGNC Symbol;Acc:HGNC:9787]
ciRNA66	ATP-binding cassette, sub-family A (ABC1), member 13 [Source:HGNC Symbol;Acc:HGNC:14638]
ciRNA67	kielin/chordin-like protein [Source:HGNC Symbol;Acc:HGNC:17585]
ciRNA68	SWI/SNF related, matrix associated, actin dependent regulator of chromatin, subfamily d, member 3 [Source:HGNC Symbol;Acc:HGNC:11108]
ciRNA69	extended synaptotagmin-like protein 2 [Source:HGNC Symbol;Acc:HGNC:22211]
ciRNA7	Uncharacterized protein [Source:UniProtKB/TrEMBL;Acc:W5P8N2]
ciRNA70	dolichyldiphosphatase 1 [Source:HGNC Symbol;Acc:HGNC:29565]
ciRNA71	ATP/GTP binding protein-like 5 [Source:HGNC Symbol;Acc:HGNC:26147]
ciRNA72	sorting nexin 17 [Source:HGNC Symbol;Acc:HGNC:14979]
ciRNA73	polyribonucleotide nucleotidyltransferase 1 [Source:HGNC Symbol;Acc:HGNC:23166]
ciRNA74	coatomer protein complex, subunit zeta 1 [Source:HGNC Symbol;Acc:HGNC:2243]
ciRNA75	solute carrier family 2 (facilitated glucose transporter), member 13 [Source:HGNC Symbol;Acc:HGNC:15956]
ciRNA76	integrin, alpha 7 [Source:HGNC Symbol;Acc:HGNC:6143]
ciRNA77	polymerase (RNA) III (DNA directed) polypeptide B [Source:HGNC Symbol;Acc:HGNC:30348]
ciRNA78	polyhomeotic homolog 1 (Drosophila) [Source:HGNC Symbol;Acc:HGNC:3182]
ciRNA79	inhibitor of growth family, member 4 [Source:HGNC Symbol;Acc:HGNC:19423]
ciRNA8	SAM domain and HD domain 1 [Source:HGNC Symbol;Acc:HGNC:15925]
ciRNA80	bromodomain containing 1 [Source:HGNC Symbol;Acc:HGNC:1102]
ciRNA81	titin [Source:HGNC Symbol;Acc:HGNC:12403]
ciRNA82	Rap guanine nucleotide exchange factor (GEF) 4 [Source:HGNC Symbol;Acc:HGNC:16626]
ciRNA83	polypeptide N-acetylgalactosaminyltransferase 13 [Source:HGNC Symbol;Acc:HGNC:23242]
ciRNA84	solute carrier family 4 (anion exchanger), member 3 [Source:HGNC Symbol;Acc:HGNC:11029]
ciRNA85	microtubule associated serine/threonine kinase 2 [Source:HGNC Symbol;Acc:HGNC:19035]

ciRNA86	microtubule associated serine/threonine kinase 2 [Source:HGNC Symbol;Acc:HGNC:19035]
ciRNA87	microtubule associated serine/threonine kinase 2 [Source:HGNC Symbol;Acc:HGNC:19035]
ciRNA88	microtubule associated serine/threonine kinase 2 [Source:HGNC Symbol;Acc:HGNC:19035]
ciRNA89	microtubule associated serine/threonine kinase 2 [Source:HGNC Symbol;Acc:HGNC:19035]
ciRNA9	glutamyl-prolyl-tRNA synthetase [Source:HGNC Symbol;Acc:HGNC:3418]
ciRNA90	microtubule associated serine/threonine kinase 2 [Source:HGNC Symbol;Acc:HGNC:19035]
ciRNA91	cysteine conjugate-beta lyase 2 [Source:HGNC Symbol;Acc:HGNC:33238]
ciRNA92	Uncharacterized protein [Source:UniProtKB/TrEMBL;Acc:W5NU48]
ciRNA93	roundabout, axon guidance receptor, homolog 1 (Drosophila) [Source:HGNC Symbol;Acc:HGNC:10249]
ciRNA94	MYCBP-associated, testis expressed 1 [Source:HGNC Symbol;Acc:HGNC:24010]
ciRNA95	leucine rich repeat containing 58 [Source:HGNC Symbol;Acc:HGNC:26968]
ciRNA96	gasdermin D [Source:HGNC Symbol;Acc:HGNC:25697]
ciRNA97	A kinase (PRKA) anchor protein 7 [Source:HGNC Symbol;Acc:HGNC:377]
ciRNA98	mediator complex subunit 23 [Source:HGNC Symbol;Acc:HGNC:2372]
circRNA1	arylsulfatase D [Source:HGNC Symbol;Acc:HGNC:717]
circRNA10	angiomotin [Source:HGNC Symbol;Acc:HGNC:17810]
circRNA100	neurobeachin [Source:HGNC Symbol;Acc:HGNC:7648]
circRNA101	PDS5, regulator of cohesion maintenance, homolog B (S. cerevisiae) [Source:HGNC Symbol;Acc:HGNC:20418]
circRNA102	furry homolog (Drosophila) [Source:HGNC Symbol;Acc:HGNC:20367]
circRNA103	furry homolog (Drosophila) [Source:HGNC Symbol;Acc:HGNC:20367]
circRNA104	furry homolog (Drosophila) [Source:HGNC Symbol;Acc:HGNC:20367]
circRNA105	furry homolog (Drosophila) [Source:HGNC Symbol;Acc:HGNC:20367]
circRNA106	WAS protein family, member 3 [Source:HGNC Symbol;Acc:HGNC:12734]
circRNA107	cyclin-dependent kinase 8 [Source:HGNC Symbol;Acc:HGNC:1779]
circRNA108	sarcoglycan, gamma (35kDa dystrophin-associated glycoprotein) [Source:HGNC Symbol;Acc:HGNC:10809]
circRNA109	LIM domain 7 [Source:HGNC Symbol;Acc:HGNC:6646]
circRNA11	sushi-repeat containing protein, X-linked 2 [Source:HGNC Symbol;Acc:HGNC:30668]
circRNA110	ring finger protein 219 [Source:HGNC Symbol;Acc:HGNC:20308]
circRNA111	myosin XVI [Source:HGNC Symbol;Acc:HGNC:29822]
circRNA112	Rho guanine nucleotide exchange factor (GEF) 7 [Source:HGNC Symbol;Acc:HGNC:15607]
circRNA113	Rho guanine nucleotide exchange factor (GEF) 7 [Source:HGNC Symbol;Acc:HGNC:15607]
circRNA114	kelch-like family member 2 [Source:HGNC Symbol;Acc:HGNC:6353]
circRNA115	Rho GTPase activating protein 10 [Source:HGNC Symbol;Acc:HGNC:26099]
circRNA116	Rho GTPase activating protein 10 [Source:HGNC Symbol;Acc:HGNC:26099]
circRNA117	Rho GTPase activating protein 10 [Source:HGNC Symbol;Acc:HGNC:26099]
circRNA118	zinc finger protein 827 [Source:HGNC Symbol;Acc:HGNC:27193]
circRNA119	OTU deubiquitinase 4 [Source:HGNC Symbol;Acc:HGNC:24949]
circRNA12	kinesin family member 16B [Source:HGNC Symbol;Acc:HGNC:15869]
circRNA120	ATP-binding cassette, sub-family E (OABP), member 1 [Source:HGNC Symbol;Acc:HGNC:69]
circRNA121	ring finger protein 150 [Source:HGNC Symbol;Acc:HGNC:23138]
circRNA122	Histone-lysine N-methyltransferase SETD7 [Source:UniProtKB/TrEMBL;Acc:W5PU61]
circRNA123	Histone-lysine N-methyltransferase SETD7 [Source:UniProtKB/TrEMBL;Acc:W5PU61]
circRNA124	E74-like factor 2 (ets domain transcription factor) [Source:HGNC Symbol;Acc:HGNC:3317]
circRNA125	Rap guanine nucleotide exchange factor (GEF) 2 [Source:HGNC Symbol;Acc:HGNC:16854]
circRNA126	Rap guanine nucleotide exchange factor (GEF) 2 [Source:HGNC Symbol;Acc:HGNC:16854]
circRNA127	zinc finger protein 84 [Source:HGNC Symbol;Acc:HGNC:13159]
circRNA128	scavenger receptor class B, member 1 [Source:HGNC Symbol;Acc:HGNC:1664]
circRNA129	Rab interacting lysosomal protein-like 1 [Source:HGNC Symbol;Acc:HGNC:26814]
circRNA13	Scm-like with four mbt domains 2 [Source:HGNC Symbol;Acc:HGNC:20256]
circRNA130	M-phase phosphoprotein 9 [Source:HGNC Symbol;Acc:HGNC:7215]
circRNA131	phosphatidylinositol transfer protein, membrane-associated 2 [Source:HGNC Symbol;Acc:HGNC:21044]
circRNA132	CAP-GLY domain containing linker protein 1 [Source:HGNC Symbol;Acc:HGNC:10461]
circRNA133	suppressor of defective silencing 3 homolog (S. cerevisiae) [Source:HGNC Symbol;Acc:HGNC:29545]
circRNA134	ring finger protein, transmembrane 2 [Source:HGNC Symbol;Acc:HGNC:25905]
circRNA135	glycolipid transfer protein [Source:HGNC Symbol;Acc:HGNC:24867]
circRNA136	acetyl-CoA carboxylase beta [Source:HGNC Symbol;Acc:HGNC:85]
circRNA137	acetyl-CoA carboxylase beta [Source:HGNC Symbol;Acc:HGNC:85]
circRNA138	acetyl-CoA carboxylase beta [Source:HGNC Symbol;Acc:HGNC:85]
circRNA139	acetyl-CoA carboxylase beta [Source:HGNC Symbol;Acc:HGNC:85]
circRNA14	Scm-like with four mbt domains 2 [Source:HGNC Symbol;Acc:HGNC:20256]
circRNA140	myosin XVIIIB [Source:HGNC Symbol;Acc:HGNC:18150]
circRNA141	myotubularin related protein 3 [Source:HGNC Symbol;Acc:HGNC:7451]
circRNA142	thioredoxin reductase 2 [Source:HGNC Symbol;Acc:HGNC:18155]
circRNA143	serine/threonine kinase 10 [Source:HGNC Symbol;Acc:HGNC:11388]
circRNA144	G elongation factor, mitochondrial 2 [Source:HGNC Symbol;Acc:HGNC:29682]
circRNA145	G elongation factor, mitochondrial 2 [Source:HGNC Symbol;Acc:HGNC:29682]
circRNA146	Rho guanine nucleotide exchange factor (GEF) 28 [Source:HGNC Symbol;Acc:HGNC:30322]
circRNA147	transportin 1 [Source:HGNC Symbol;Acc:HGNC:6401]
circRNA148	microtubule associated serine/threonine kinase family member 4 [Source:HGNC Symbol;Acc:HGNC:19037]
circRNA149	importin 11 [Source:HGNC Symbol;Acc:HGNC:20628]
circRNA15	Scm-like with four mbt domains 2 [Source:HGNC Symbol;Acc:HGNC:20256]
circRNA150	3-oxoacid CoA transferase 1 [Source:HGNC Symbol;Acc:HGNC:8527]
circRNA151	RPTOR independent companion of MTOR, complex 2 [Source:HGNC Symbol;Acc:HGNC:28611]
circRNA152	NAD kinase 2, mitochondrial [Source:HGNC Symbol;Acc:HGNC:26404]
circRNA153	ADAM metallopeptidase with thrombospondin type 1 motif, 12 [Source:HGNC Symbol;Acc:HGNC:14605]
circRNA154	ADAM metallopeptidase with thrombospondin type 1 motif, 12 [Source:HGNC Symbol;Acc:HGNC:14605]
circRNA155	family with sequence similarity 105, member A [Source:HGNC Symbol;Acc:HGNC:25629]
circRNA156	trio Rho guanine nucleotide exchange factor [Source:HGNC Symbol;Acc:HGNC:12303]
circRNA157	ankyrin repeat domain 33B [Source:HGNC Symbol;Acc:HGNC:35240]
circRNA158	NA
circRNA159	adenylate cyclase 2 (brain) [Source:HGNC Symbol;Acc:HGNC:233]
circRNA16	dehydrogenase E1 and transketolase domain containing 1 [Source:HGNC Symbol;Acc:HGNC:23537]
circRNA160	adenylate cyclase 2 (brain) [Source:HGNC Symbol;Acc:HGNC:233]
circRNA161	adenylate cyclase 2 (brain) [Source:HGNC Symbol;Acc:HGNC:233]
circRNA162	sestrin 3 [Source:HGNC Symbol;Acc:HGNC:23060]
circRNA163	acetyl-CoA acetyltransferase 1 [Source:HGNC Symbol;Acc:HGNC:93]
circRNA164	SRY (sex determining region Y)-box 6 [Source:HGNC Symbol;Acc:HGNC:16421]
circRNA165	coatomer protein complex, subunit beta 1 [Source:HGNC Symbol;Acc:HGNC:2231]
circRNA166	TEA domain family member 1 (SV40 transcriptional enhancer factor) [Source:HGNC Symbol;Acc:HGNC:11714]
circRNA167	TEA domain family member 1 (SV40 transcriptional enhancer factor) [Source:HGNC Symbol;Acc:HGNC:11714]
circRNA168	ubiquitin specific peptidase 47 [Source:HGNC Symbol;Acc:HGNC:20076]
circRNA169	SET binding factor 2 [Source:HGNC Symbol;Acc:HGNC:2135]
circRNA17	YME1-like 1 ATPase [Source:HGNC Symbol;Acc:HGNC:12843]
circRNA170	signal peptide, CUB domain, EGF-like 2 [Source:HGNC Symbol;Acc:HGNC:30425]
circRNA171	PTPRF interacting protein, binding protein 2 (liprin beta 2) [Source:HGNC Symbol;Acc:HGNC:9250]
circRNA172	tripartite motif containing 3 [Source:HGNC Symbol;Acc:HGNC:10064]
circRNA173	nucleoporin 98kDa [Source:HGNC Symbol;Acc:HGNC:8068]
circRNA174	FCH and double SH3 domains 2 [Source:HGNC Symbol;Acc:HGNC:29114]

circRNA175	apoptosis inhibitor 5 [Source:HGNC Symbol;Acc:HGNC:594]
circRNA176	autophagy/beclin-1 regulator 1 [Source:HGNC Symbol;Acc:HGNC:25990]
circRNA177	cytoskeleton associated protein 5 [Source:HGNC Symbol;Acc:HGNC:28959]
circRNA178	Uncharacterized protein [Source:UniProtKB/TrEMBL;Acc:W5PL25]
circRNA179	c-Maf inducing protein [Source:HGNC Symbol;Acc:HGNC:24319]
circRNA18	cAMP responsive element modulator [Source:HGNC Symbol;Acc:HGNC:2352]
circRNA180	F-box protein 31 [Source:HGNC Symbol;Acc:HGNC:16510]
circRNA181	vacuolar protein sorting 35 homolog (S. cerevisiae) [Source:HGNC Symbol;Acc:HGNC:13487]
circRNA182	phosphorylase kinase, beta [Source:HGNC Symbol;Acc:HGNC:8927]
circRNA183	Ovis aries fat mass and obesity associated (FTO), mRNA. [Source:RefSeq mRNA;Acc:NM_001104931]
circRNA184	autocrine motility factor receptor, E3 ubiquitin protein ligase [Source:HGNC Symbol;Acc:HGNC:463]
circRNA185	glucose-fructose oxidoreductase domain containing 2 [Source:HGNC Symbol;Acc:HGNC:28159]
circRNA186	transport and golgi organization 6 homolog (Drosophila) [Source:HGNC Symbol;Acc:HGNC:25749]
circRNA187	rhophilin, Rho GTPase binding protein 2 [Source:HGNC Symbol;Acc:HGNC:19974]
circRNA188	ubiquitin-like modifier activating enzyme 2 [Source:HGNC Symbol;Acc:HGNC:30661]
circRNA189	hepsin [Source:HGNC Symbol;Acc:HGNC:5155]
circRNA19	par-3 family cell polarity regulator [Source:HGNC Symbol;Acc:HGNC:16051]
circRNA190	calpain, small subunit 1 [Source:HGNC Symbol;Acc:HGNC:1481]
circRNA191	ryanodine receptor 1 (skeletal) [Source:HGNC Symbol;Acc:HGNC:10483]
circRNA192	ryanodine receptor 1 (skeletal) [Source:HGNC Symbol;Acc:HGNC:10483]
circRNA193	creatine kinase, muscle [Source:HGNC Symbol;Acc:HGNC:1994]
circRNA194	SUMO1 activating enzyme subunit 1 [Source:HGNC Symbol;Acc:HGNC:30660]
circRNA195	DEAH (Asp-Glu-Ala-His) box polypeptide 34 [Source:HGNC Symbol;Acc:HGNC:16719]
circRNA196	coiled-coil domain containing 114 [Source:HGNC Symbol;Acc:HGNC:26560]
circRNA197	nucleobindin 1 [Source:HGNC Symbol;Acc:HGNC:8043]
circRNA198	glycogen synthase 1 (muscle) [Source:HGNC Symbol;Acc:HGNC:4706]
circRNA199	myosin, heavy chain 14, non-muscle [Source:HGNC Symbol;Acc:HGNC:23212]
circRNA2	chloride channel, voltage-sensitive 4 [Source:HGNC Symbol;Acc:HGNC:2022]
circRNA20	par-3 family cell polarity regulator [Source:HGNC Symbol;Acc:HGNC:16051]
circRNA200	Uncharacterized protein [Source:UniProtKB/TrEMBL;Acc:W5PZB5]
circRNA201	zinc finger protein 583 [Source:HGNC Symbol;Acc:HGNC:26427]
circRNA202	doublecortin-like kinase 3 [Source:HGNC Symbol;Acc:HGNC:19005]
circRNA203	golgin A4 [Source:HGNC Symbol;Acc:HGNC:4427]
circRNA204	Uncharacterized protein [Source:UniProtKB/TrEMBL;Acc:W5NXR1]
circRNA205	anoctamin 10 [Source:HGNC Symbol;Acc:HGNC:25519]
circRNA206	T cell activation inhibitor, mitochondrial [Source:HGNC Symbol;Acc:HGNC:25241]
circRNA207	ER degradation enhancer, mannosidase alpha-like 1 [Source:HGNC Symbol;Acc:HGNC:18967]
circRNA208	inositol 1,4,5-trisphosphate receptor, type 1 [Source:HGNC Symbol;Acc:HGNC:6180]
circRNA209	inositol 1,4,5-trisphosphate receptor, type 1 [Source:HGNC Symbol;Acc:HGNC:6180]
circRNA21	KIAA1217 [Source:HGNC Symbol;Acc:HGNC:25428]
circRNA210	succinate-CoA ligase, GDP-forming, beta subunit [Source:HGNC Symbol;Acc:HGNC:11450]
circRNA211	leucine-rich repeats and immunoglobulin-like domains 1 [Source:HGNC Symbol;Acc:HGNC:17360]
circRNA212	Ovis aries solute carrier family 25 (S-adenosylmethionine carrier), member 26 (SLC25A26), nuclear gene encoding mitochondrial protein, mRNA. [Source:RefSeq mRNA;Acc:NM_001127292]
circRNA213	Ovis aries solute carrier family 25 (S-adenosylmethionine carrier), member 26 (SLC25A26), nuclear gene encoding mitochondrial protein, mRNA. [Source:RefSeq mRNA;Acc:NM_001127292]
circRNA214	sarcolemma associated protein [Source:HGNC Symbol;Acc:HGNC:16643]
circRNA215	Ovis aries ADP-ribosylation factor 4 (ARF4), mRNA. [Source:RefSeq mRNA;Acc:NM_001162546]
circRNA216	interleukin 17 receptor D [Source:HGNC Symbol;Acc:HGNC:17616]
circRNA217	Uncharacterized protein [Source:UniProtKB/TrEMBL;Acc:W5NQD8]
circRNA218	Scm-like with four mbt domains 1 [Source:HGNC Symbol;Acc:HGNC:20255]
circRNA219	NIMA-related kinase 4 [Source:HGNC Symbol;Acc:HGNC:11399]
circRNA22	family with sequence similarity 188, member A [Source:HGNC Symbol;Acc:HGNC:23578]
circRNA220	glycosyltransferase 8 domain containing 1 [Source:HGNC Symbol;Acc:HGNC:24870]
circRNA221	polybromo 1 [Source:HGNC Symbol;Acc:HGNC:30064]
circRNA222	ubiquitin specific peptidase 4 (proto-oncogene) [Source:HGNC Symbol;Acc:HGNC:12627]
circRNA223	FYVE and coiled-coil domain containing 1 [Source:HGNC Symbol;Acc:HGNC:14673]
circRNA224	leucine zipper transcription factor-like 1 [Source:HGNC Symbol;Acc:HGNC:6741]
circRNA225	SAC1 suppressor of actin mutations 1-like (yeast) [Source:HGNC Symbol;Acc:HGNC:17059]
circRNA226	LIM domains containing 1 [Source:HGNC Symbol;Acc:HGNC:6612]
circRNA227	zinc finger, DHHC-type containing 3 [Source:HGNC Symbol;Acc:HGNC:18470]
circRNA228	zinc finger, DHHC-type containing 3 [Source:HGNC Symbol;Acc:HGNC:18470]
circRNA229	transmembrane and coiled-coil domain family 1 [Source:HGNC Symbol;Acc:HGNC:29116]
circRNA23	transmembrane protein 236 [Source:HGNC Symbol;Acc:HGNC:23473]
circRNA230	nuclear receptor subfamily 2, group C, member 2 [Source:HGNC Symbol;Acc:HGNC:7972]
circRNA231	FYVE, RhoGEF and PH domain containing 5 [Source:HGNC Symbol;Acc:HGNC:19117]
circRNA232	glutamate receptor interacting protein 2 [Source:HGNC Symbol;Acc:HGNC:23841]
circRNA233	eukaryotic elongation factor, selenocysteine-tRNA-specific [Source:HGNC Symbol;Acc:HGNC:24614]
circRNA234	ankyrin repeat and SOCS box containing 7 [Source:HGNC Symbol;Acc:HGNC:17182]
circRNA235	myocyte enhancer factor 2A [Source:HGNC Symbol;Acc:HGNC:6993]
circRNA236	tetratricopeptide repeat domain 23 [Source:HGNC Symbol;Acc:HGNC:25730]
circRNA237	Uncharacterized protein [Source:UniProtKB/TrEMBL;Acc:W5PKY6]
circRNA238	Uncharacterized protein [Source:UniProtKB/TrEMBL;Acc:W5PKY6]
circRNA239	ATP/GTP binding protein-like 1 [Source:HGNC Symbol;Acc:HGNC:26504]
circRNA24	supervillin [Source:HGNC Symbol;Acc:HGNC:11480]
circRNA240	ATP/GTP binding protein-like 1 [Source:HGNC Symbol;Acc:HGNC:26504]
circRNA241	abhydrolase domain containing 2 [Source:HGNC Symbol;Acc:HGNC:18717]
circRNA242	Uncharacterized protein [Source:UniProtKB/TrEMBL;Acc:W5PQT3]
circRNA243	isocitrate dehydrogenase 2 (NADP+), mitochondrial [Source:HGNC Symbol;Acc:HGNC:5383]
circRNA244	isocitrate dehydrogenase 2 (NADP+), mitochondrial [Source:HGNC Symbol;Acc:HGNC:5383]
circRNA245	phosphodiesterase 8A [Source:HGNC Symbol;Acc:HGNC:8793]
circRNA246	fibronectin type III and SPRY domain containing 2 [Source:HGNC Symbol;Acc:HGNC:18024]
circRNA247	tetraspanin 3 [Source:HGNC Symbol;Acc:HGNC:17752]
circRNA248	tetraspanin 3 [Source:HGNC Symbol;Acc:HGNC:17752]
circRNA249	syntaxin binding protein 6 (amisyn) [Source:HGNC Symbol;Acc:HGNC:19666]
circRNA25	CSRP2 binding protein [Source:HGNC Symbol;Acc:HGNC:15904]
circRNA250	HECT domain containing E3 ubiquitin protein ligase 1 [Source:HGNC Symbol;Acc:HGNC:20157]
circRNA251	HEAT repeat containing 5A [Source:HGNC Symbol;Acc:HGNC:20276]
circRNA252	A kinase (PRKA) anchor protein 6 [Source:HGNC Symbol;Acc:HGNC:376]
circRNA253	Ral GTPase activating protein, alpha subunit 1 (catalytic) [Source:HGNC Symbol;Acc:HGNC:17770]
circRNA254	Uncharacterized protein [Source:UniProtKB/TrEMBL;Acc:W5PFX4]
circRNA255	inositol-tetrakisphosphate 1-kinase [Source:HGNC Symbol;Acc:HGNC:6177]
circRNA256	vaccinia related kinase 1 [Source:HGNC Symbol;Acc:HGNC:12718]
circRNA257	MAP/microtubule affinity-regulating kinase 3 [Source:HGNC Symbol;Acc:HGNC:6897]
circRNA258	MAP/microtubule affinity-regulating kinase 3 [Source:HGNC Symbol;Acc:HGNC:6897]
circRNA259	protein phosphatase 1, regulatory subunit 13B [Source:HGNC Symbol;Acc:HGNC:14950]
circRNA26	family with sequence similarity 208, member B [Source:HGNC Symbol;Acc:HGNC:23484]
circRNA260	BRF1, RNA polymerase III transcription initiation factor 90 kDa subunit [Source:HGNC Symbol;Acc:HGNC:11551]
circRNA261	myomesin 2 [Source:HGNC Symbol;Acc:HGNC:7614]
circRNA262	vascular endothelial growth factor C [Source:HGNC Symbol;Acc:HGNC:12682]

circRNA263	aspartylglucosaminidase [Source:HGNC Symbol;Acc:HGNC:318]
circRNA264	mitochondrial calcium uptake family, member 3 [Source:HGNC Symbol;Acc:HGNC:27820]
circRNA265	DLC1 Rho GTPase activating protein [Source:HGNC Symbol;Acc:HGNC:2897]
circRNA266	DLC1 Rho GTPase activating protein [Source:HGNC Symbol;Acc:HGNC:2897]
circRNA267	exoribonuclease 1 [Source:HGNC Symbol;Acc:HGNC:23994]
circRNA268	general transcription factor IIE, polypeptide 2, beta 34kDa [Source:HGNC Symbol;Acc:HGNC:4651]
circRNA269	fucosyltransferase 10 (alpha (1,3) fucosyltransferase) [Source:HGNC Symbol;Acc:HGNC:19234]
circRNA27	Uncharacterized protein [Source:UniProtKB/TrEMBL;Acc:W5PPX3]
circRNA270	ash2 (absent, small, or homeotic)-like (Drosophila) [Source:HGNC Symbol;Acc:HGNC:744]
circRNA271	Wolf-Hirschhorn syndrome candidate 1-like 1 [Source:HGNC Symbol;Acc:HGNC:12767]
circRNA272	leucine zipper-EF-hand containing transmembrane protein 2 [Source:HGNC Symbol;Acc:HGNC:14648]
circRNA273	transforming, acidic coiled-coil containing protein 1 [Source:HGNC Symbol;Acc:HGNC:11522]
circRNA274	ADAM metallopeptidase domain 9 [Source:HGNC Symbol;Acc:HGNC:216]
circRNA275	ADAM metallopeptidase domain 9 [Source:HGNC Symbol;Acc:HGNC:216]
circRNA276	pleckstrin and Sec7 domain containing 3 [Source:HGNC Symbol;Acc:HGNC:19093]
circRNA277	pleckstrin and Sec7 domain containing 3 [Source:HGNC Symbol;Acc:HGNC:19093]
circRNA278	N-glycanase 1 [Source:HGNC Symbol;Acc:HGNC:17646]
circRNA279	axin 1 [Source:HGNC Symbol;Acc:HGNC:903]
circRNA28	glycerophosphocholine phosphodiesterase GDE1 homolog (S. cerevisiae) [Source:HGNC Symbol;Acc:HGNC:26957]
circRNA280	adenylate cyclase 9 [Source:HGNC Symbol;Acc:HGNC:240]
circRNA281	G1 to S phase transition 1 [Source:HGNC Symbol;Acc:HGNC:4621]
circRNA282	G1 to S phase transition 1 [Source:HGNC Symbol;Acc:HGNC:4621]
circRNA283	Uncharacterized protein [Source:UniProtKB/TrEMBL;Acc:W5PB62]
circRNA284	Uncharacterized protein [Source:UniProtKB/TrEMBL;Acc:W5PB62]
circRNA285	xylosyltransferase I [Source:HGNC Symbol;Acc:HGNC:15516]
circRNA286	xylosyltransferase I [Source:HGNC Symbol;Acc:HGNC:15516]
circRNA287	Uncharacterized protein [Source:UniProtKB/TrEMBL;Acc:W5PMR0]
circRNA288	Uncharacterized protein [Source:UniProtKB/TrEMBL;Acc:W5PMR0]
circRNA289	eukaryotic elongation factor-2 kinase [Source:HGNC Symbol;Acc:HGNC:24615]
circRNA29	glycerophosphocholine phosphodiesterase GDE1 homolog (S. cerevisiae) [Source:HGNC Symbol;Acc:HGNC:26957]
circRNA290	polymerase (RNA) III (DNA directed) polypeptide E (80kD) [Source:HGNC Symbol;Acc:HGNC:30347]
circRNA291	ubiquitin specific peptidase 31 [Source:HGNC Symbol;Acc:HGNC:20060]
circRNA292	retinoblastoma binding protein 6 [Source:HGNC Symbol;Acc:HGNC:9889]
circRNA293	KIAA0556 [Source:HGNC Symbol;Acc:HGNC:29068]
circRNA294	ATPase, Ca++ transporting, cardiac muscle, fast twitch 1 [Source:HGNC Symbol;Acc:HGNC:811]
circRNA295	ATPase, Ca++ transporting, cardiac muscle, fast twitch 1 [Source:HGNC Symbol;Acc:HGNC:811]
circRNA296	integrin alpha-M precursor [Source:RefSeq peptide;Acc:NP_001076062]
circRNA297	Uncharacterized protein [Source:UniProtKB/TrEMBL;Acc:W5PXK7]
circRNA298	leucine-rich repeats and WD repeat domain containing 1 [Source:HGNC Symbol;Acc:HGNC:21769]
circRNA299	zinc finger protein 12 [Source:HGNC Symbol;Acc:HGNC:12902]
circRNA3	RALBP1 associated Eps domain containing 2 [Source:HGNC Symbol;Acc:HGNC:9963]
circRNA30	glycerophosphocholine phosphodiesterase GDE1 homolog (S. cerevisiae) [Source:HGNC Symbol;Acc:HGNC:26957]
circRNA300	Uncharacterized protein [Source:UniProtKB/TrEMBL;Acc:W5P2C7]
circRNA301	disrupted in schizophrenia 1 [Source:HGNC Symbol;Acc:HGNC:2888]
circRNA302	Uncharacterized protein [Source:UniProtKB/TrEMBL;Acc:W5NZM3]
circRNA303	actinin, alpha 2 [Source:HGNC Symbol;Acc:HGNC:164]
circRNA304	actinin, alpha 2 [Source:HGNC Symbol;Acc:HGNC:164]
circRNA305	BicC family RNA binding protein 1 [Source:HGNC Symbol;Acc:HGNC:19351]
circRNA306	family with sequence similarity 13, member C [Source:HGNC Symbol;Acc:HGNC:19371]
circRNA307	family with sequence similarity 13, member C [Source:HGNC Symbol;Acc:HGNC:19371]
circRNA308	AT rich interactive domain 5B (MRF1-like) [Source:HGNC Symbol;Acc:HGNC:17362]
circRNA309	Uncharacterized protein [Source:UniProtKB/TrEMBL;Acc:W5P3S6]
circRNA31	pantothenate kinase 2, mitochondrial [Source:RefSeq peptide;Acc:NP_001136365]
circRNA310	HECT and RLD domain containing E3 ubiquitin protein ligase 4 [Source:HGNC Symbol;Acc:HGNC:24521]
circRNA311	myopalladin [Source:HGNC Symbol;Acc:HGNC:23246]
circRNA312	hexokinase 1 [Source:HGNC Symbol;Acc:HGNC:4922]
circRNA313	activating signal cointegrator 1 complex subunit 1 [Source:HGNC Symbol;Acc:HGNC:24268]
circRNA314	mitogen-activated protein kinase 8 [Source:HGNC Symbol;Acc:HGNC:6881]
circRNA315	ubiquitin-conjugating enzyme E2D 1 [Source:HGNC Symbol;Acc:HGNC:12474]
circRNA316	protein kinase, cGMP-dependent, type I [Source:HGNC Symbol;Acc:HGNC:9414]
circRNA317	protein kinase, cGMP-dependent, type I [Source:HGNC Symbol;Acc:HGNC:9414]
circRNA318	tankyrase, TRF1-interacting ankyrin-related ADP-ribose polymerase 2 [Source:HGNC Symbol;Acc:HGNC:15677]
circRNA319	BTAF1 RNA polymerase II, B-TFIID transcription factor-associated, 170kDa [Source:HGNC Symbol;Acc:HGNC:17307]
circRNA32	chromosome 20 open reading frame 194 [Source:HGNC Symbol;Acc:HGNC:17721]
circRNA320	cytoplasmic polyadenylation element binding protein 3 [Source:HGNC Symbol;Acc:HGNC:21746]
circRNA321	fragile site, folic acid type, rare, fra(10)(q23.3) or fra(10)(q24.2) candidate 1 [Source:HGNC Symbol;Acc:HGNC:1162]
circRNA322	sorbin and SH3 domain containing 1 [Source:HGNC Symbol;Acc:HGNC:14565]
circRNA323	sorbin and SH3 domain containing 1 [Source:HGNC Symbol;Acc:HGNC:14565]
circRNA324	F-box and WD repeat domain containing 4 [Source:HGNC Symbol;Acc:HGNC:10847]
circRNA325	F-box and WD repeat domain containing 4 [Source:HGNC Symbol;Acc:HGNC:10847]
circRNA326	F-box and WD repeat domain containing 4 [Source:HGNC Symbol;Acc:HGNC:10847]
circRNA327	F-box and WD repeat domain containing 4 [Source:HGNC Symbol;Acc:HGNC:10847]
circRNA328	Kv channel interacting protein 2 [Source:HGNC Symbol;Acc:HGNC:15522]
circRNA329	vesicle transport through interaction with t-SNAREs 1A [Source:HGNC Symbol;Acc:HGNC:17792]
circRNA33	protein tyrosine phosphatase, receptor type, A [Source:HGNC Symbol;Acc:HGNC:9664]
circRNA330	nebulin-related anchoring protein [Source:HGNC Symbol;Acc:HGNC:7988]
circRNA331	nebulin-related anchoring protein [Source:HGNC Symbol;Acc:HGNC:7988]
circRNA332	inositol polyphosphate-5-phosphatase F [Source:HGNC Symbol;Acc:HGNC:17054]
circRNA333	transforming, acidic coiled-coil containing protein 2 [Source:HGNC Symbol;Acc:HGNC:11523]
circRNA334	transforming, acidic coiled-coil containing protein 2 [Source:HGNC Symbol;Acc:HGNC:11523]
circRNA335	transforming, acidic coiled-coil containing protein 2 [Source:HGNC Symbol;Acc:HGNC:11523]
circRNA336	dedicator of cytokinesis 1 [Source:HGNC Symbol;Acc:HGNC:2987]
circRNA337	dedicator of cytokinesis 1 [Source:HGNC Symbol;Acc:HGNC:2987]
circRNA338	oxysterol binding protein-like 1A [Source:HGNC Symbol;Acc:HGNC:16398]
circRNA339	oxysterol binding protein-like 1A [Source:HGNC Symbol;Acc:HGNC:16398]
circRNA34	protein tyrosine phosphatase, receptor type, A [Source:HGNC Symbol;Acc:HGNC:9664]
circRNA340	oxysterol binding protein-like 1A [Source:HGNC Symbol;Acc:HGNC:16398]
circRNA341	tetratricopeptide repeat domain 39C [Source:HGNC Symbol;Acc:HGNC:26595]
circRNA342	Cdk5 and Abl enzyme substrate 1 [Source:HGNC Symbol;Acc:HGNC:25097]
circRNA343	mindbomb E3 ubiquitin protein ligase 1 [Source:HGNC Symbol;Acc:HGNC:21086]
circRNA344	growth regulation by estrogen in breast cancer-like [Source:HGNC Symbol;Acc:HGNC:31042]
circRNA345	lipin 2 [Source:HGNC Symbol;Acc:HGNC:14450]
circRNA346	slowmo homolog 1 (Drosophila) [Source:HGNC Symbol;Acc:HGNC:24639]
circRNA347	spire-type actin nucleation factor 1 [Source:HGNC Symbol;Acc:HGNC:30622]
circRNA348	dymeclin [Source:HGNC Symbol;Acc:HGNC:21317]
circRNA349	RAB27B, member RAS oncogene family [Source:HGNC Symbol;Acc:HGNC:9767]
circRNA35	Uncharacterized protein [Source:UniProtKB/TrEMBL;Acc:W5Q6G5]
circRNA350	coiled-coil domain containing 68 [Source:HGNC Symbol;Acc:HGNC:24350]

circRNA351	transcription factor 4 [Source:HGNC Symbol;Acc:HGNC:11634]
circRNA352	zinc finger protein 532 [Source:HGNC Symbol;Acc:HGNC:30940]
circRNA353	RAB23, member RAS oncogene family [Source:HGNC Symbol;Acc:HGNC:14263]
circRNA354	RAB23, member RAS oncogene family [Source:HGNC Symbol;Acc:HGNC:14263]
circRNA355	muscular LMNA-interacting protein [Source:HGNC Symbol;Acc:HGNC:21355]
circRNA356	FK506 binding protein 5 [Source:HGNC Symbol;Acc:HGNC:3721]
circRNA357	FYVE, RhoGEF and PH domain containing 2 [Source:HGNC Symbol;Acc:HGNC:3664]
circRNA358	chloride intracellular channel 5 [Source:HGNC Symbol;Acc:HGNC:13517]
circRNA359	polycystic kidney and hepatic disease 1 (autosomal recessive) [Source:HGNC Symbol;Acc:HGNC:9016]
circRNA36	Uncharacterized protein [Source:UniProtKB/TrEMBL;Acc:W5QCL5]
circRNA360	polycystic kidney and hepatic disease 1 (autosomal recessive) [Source:HGNC Symbol;Acc:HGNC:9016]
circRNA361	MRS2 magnesium transporter [Source:HGNC Symbol;Acc:HGNC:13785]
circRNA362	CAP, adenylate cyclase-associated protein, 2 (yeast) [Source:HGNC Symbol;Acc:HGNC:20039]
circRNA363	guanosine monophosphate reductase [Source:HGNC Symbol;Acc:HGNC:4376]
circRNA364	guanosine monophosphate reductase [Source:HGNC Symbol;Acc:HGNC:4376]
circRNA365	Uncharacterized protein [Source:UniProtKB/TrEMBL;Acc:W5NPG2]
circRNA366	asparaginyl-tRNA synthetase 2, mitochondrial (putative) [Source:HGNC Symbol;Acc:HGNC:26274]
circRNA367	asparaginyl-tRNA synthetase 2, mitochondrial (putative) [Source:HGNC Symbol;Acc:HGNC:26274]
circRNA368	remodeling and spacing factor 1 [Source:HGNC Symbol;Acc:HGNC:18118]
circRNA369	PBX/knotted 1 homeobox 2 [Source:HGNC Symbol;Acc:HGNC:16714]
circRNA37	transmembrane 9 superfamily protein member 4 [Source:HGNC Symbol;Acc:HGNC:30797]
circRNA370	Rho GTPase activating protein 32 [Source:HGNC Symbol;Acc:HGNC:17399]
circRNA371	amyloid beta (A4) precursor-like protein 2 [Source:HGNC Symbol;Acc:HGNC:598]
circRNA372	fatty acid desaturase 2 [Source:HGNC Symbol;Acc:HGNC:3575]
circRNA373	protein phosphatase 6, regulatory subunit 3 [Source:HGNC Symbol;Acc:HGNC:1173]
circRNA374	DEAF1 transcription factor [Source:HGNC Symbol;Acc:HGNC:14677]
circRNA375	proteasome (prosome, macropain) 26S subunit, non-ATPase, 13 [Source:HGNC Symbol;Acc:HGNC:9558]
circRNA376	homer homolog 1 (Drosophila) [Source:HGNC Symbol;Acc:HGNC:17512]
circRNA377	homer homolog 1 (Drosophila) [Source:HGNC Symbol;Acc:HGNC:17512]
circRNA378	homer homolog 1 (Drosophila) [Source:HGNC Symbol;Acc:HGNC:17512]
circRNA379	cardiomyopathy associated 5 [Source:HGNC Symbol;Acc:HGNC:14305]
circRNA38	SAM domain and HD domain 1 [Source:HGNC Symbol;Acc:HGNC:15925]
circRNA380	caseinolytic mitochondrial matrix peptidase chaperone subunit [Source:HGNC Symbol;Acc:HGNC:2088]
circRNA381	immunoglobulin superfamily, DCC subclass, member 4 [Source:HGNC Symbol;Acc:HGNC:13770]
circRNA382	dipeptidyl-peptidase 8 [Source:HGNC Symbol;Acc:HGNC:16490]
circRNA383	alpha- and gamma-adaptin binding protein [Source:HGNC Symbol;Acc:HGNC:25662]
circRNA384	uveal autoantigen with coiled-coil domains and ankyrin repeats [Source:HGNC Symbol;Acc:HGNC:15947]
circRNA385	Uncharacterized protein [Source:UniProtKB/TrEMBL;Acc:W5QDH4]
circRNA386	glucosidase, alpha; neutral C [Source:HGNC Symbol;Acc:HGNC:4139]
circRNA387	tau tubulin kinase 2 [Source:HGNC Symbol;Acc:HGNC:19141]
circRNA388	transmembrane protein 62 [Source:HGNC Symbol;Acc:HGNC:26269]
circRNA389	transmembrane protein 62 [Source:HGNC Symbol;Acc:HGNC:26269]
circRNA39	serine incorporator 3 [Source:HGNC Symbol;Acc:HGNC:11699]
circRNA390	kelch domain containing 1 [Source:HGNC Symbol;Acc:HGNC:19836]
circRNA391	zinc finger protein 609 [Source:HGNC Symbol;Acc:HGNC:29003]
circRNA392	sorting nexin 1 [Source:HGNC Symbol;Acc:HGNC:11172]
circRNA393	HECT and RLD domain containing E3 ubiquitin protein ligase family member 1 [Source:HGNC Symbol;Acc:HGNC:4867]
circRNA394	myosin IE [Source:HGNC Symbol;Acc:HGNC:7599]
circRNA395	zinc finger protein 280D [Source:HGNC Symbol;Acc:HGNC:25953]
circRNA396	family with sequence similarity 214, member A [Source:HGNC Symbol;Acc:HGNC:25609]
circRNA397	Ovis aries transient receptor potential cation channel, subfamily M, member 7 (TRPM7), mRNA. [Source:RefSeq mRNA;Acc:NM_001093785]
circRNA398	galactokinase 2 [Source:HGNC Symbol;Acc:HGNC:4119]
circRNA399	kinectin 1 (kinesin receptor) [Source:HGNC Symbol;Acc:HGNC:6467]
circRNA4	DEAD (Asp-Glu-Ala-Asp) box helicase 3, X-linked [Source:HGNC Symbol;Acc:HGNC:2745]
circRNA40	zinc finger, MYND-type containing 8 [Source:HGNC Symbol;Acc:HGNC:9397]
circRNA400	dehydrogenase/reductase (SDR family) member 7 [Source:HGNC Symbol;Acc:HGNC:21524]
circRNA401	methylenetetrahydrofolate dehydrogenase (NADP+ dependent) 1, methenyltetrahydrofolate cyclohydrolase, formyltetrahydrofolate synthetase [Source:HGNC Symbol;Acc:HGNC:7432]
circRNA402	protein-O-mannosyltransferase 2 [Source:HGNC Symbol;Acc:HGNC:19743]
circRNA403	serine palmitoyltransferase, long chain base subunit 2 [Source:HGNC Symbol;Acc:HGNC:11278]
circRNA404	spermatogenesis associated 7 [Source:HGNC Symbol;Acc:HGNC:20423]
circRNA405	tetratricopeptide repeat domain 7B [Source:HGNC Symbol;Acc:HGNC:19858]
circRNA406	tetratricopeptide repeat domain 7B [Source:HGNC Symbol;Acc:HGNC:19858]
circRNA407	tetratricopeptide repeat domain 7B [Source:HGNC Symbol;Acc:HGNC:19858]
circRNA408	SEC24 family member D [Source:HGNC Symbol;Acc:HGNC:10706]
circRNA409	ankyrin 2, neuronal [Source:HGNC Symbol;Acc:HGNC:493]
circRNA41	ADP-ribosylation factor guanine nucleotide-exchange factor 2 (brefeldin A-inhibited) [Source:HGNC Symbol;Acc:HGNC:15853]
circRNA410	hydroxyacyl-CoA dehydrogenase [Source:HGNC Symbol;Acc:HGNC:4799]
circRNA411	hydroxyacyl-CoA dehydrogenase [Source:HGNC Symbol;Acc:HGNC:4799]
circRNA412	hydroxyacyl-CoA dehydrogenase [Source:HGNC Symbol;Acc:HGNC:4799]
circRNA413	Uncharacterized protein [Source:UniProtKB/TrEMBL;Acc:W5QBL4]
circRNA414	zinc finger, CCHC domain containing 4 [Source:HGNC Symbol;Acc:HGNC:22917]
circRNA415	NEDD4 binding protein 2 [Source:HGNC Symbol;Acc:HGNC:29851]
circRNA416	amyloid beta (A4) precursor protein-binding, family B, member 2 [Source:HGNC Symbol;Acc:HGNC:582]
circRNA417	LIM and calponin homology domains 1 [Source:HGNC Symbol;Acc:HGNC:29191]
circRNA418	ATPase, class V, type 10D [Source:HGNC Symbol;Acc:HGNC:13549]
circRNA419	corin, serine peptidase [Source:HGNC Symbol;Acc:HGNC:19012]
circRNA42	staufen double-stranded RNA binding protein 1 [Source:HGNC Symbol;Acc:HGNC:11370]
circRNA420	sarcoglycan, beta (43kDa dystrophin-associated glycoprotein) [Source:HGNC Symbol;Acc:HGNC:10806]
circRNA421	methylenetetrahydrofolate dehydrogenase (NADP+ dependent) 2-like [Source:HGNC Symbol;Acc:HGNC:31865]
circRNA422	USO1 vesicle transport factor [Source:HGNC Symbol;Acc:HGNC:30904]
circRNA423	USO1 vesicle transport factor [Source:HGNC Symbol;Acc:HGNC:30904]
circRNA424	CCR4-NOT transcription complex, subunit 6-like [Source:HGNC Symbol;Acc:HGNC:18042]
circRNA425	Fraser extracellular matrix complex subunit 1 [Source:HGNC Symbol;Acc:HGNC:19185]
circRNA426	Fraser extracellular matrix complex subunit 1 [Source:HGNC Symbol;Acc:HGNC:19185]
circRNA427	protein tyrosine phosphatase, non-receptor type 13 (APO-1/CD95 (Fas)-associated phosphatase) [Source:HGNC Symbol;Acc:HGNC:9646]
circRNA428	Uncharacterized protein [Source:UniProtKB/TrEMBL;Acc:W5P620]
circRNA429	Uncharacterized protein [Source:UniProtKB/TrEMBL;Acc:W5P620]
circRNA43	TMEM189-UBE2V1 readthrough [Source:HGNC Symbol;Acc:HGNC:33521]
circRNA430	Uncharacterized protein [Source:UniProtKB/TrEMBL;Acc:W5P620]
circRNA431	Uncharacterized protein [Source:UniProtKB/TrEMBL;Acc:W5P620]
circRNA432	Uncharacterized protein [Source:UniProtKB/TrEMBL;Acc:W5P620]
circRNA433	Uncharacterized protein [Source:UniProtKB/TrEMBL;Acc:W5P620]
circRNA434	Uncharacterized protein [Source:UniProtKB/TrEMBL;Acc:W5P620]
circRNA435	Uncharacterized protein [Source:UniProtKB/TrEMBL;Acc:W5P620]
circRNA436	epidermal growth factor receptor pathway substrate 15-like 1 [Source:HGNC Symbol;Acc:HGNC:24634]
circRNA437	protein kinase N1 [Source:HGNC Symbol;Acc:HGNC:9405]
circRNA438	MHC class II regulatory factor RFX1 [Source:UniProtKB/Swiss-Prot;Acc:P22670]
circRNA439	phosphodiesterase 4A, cAMP-specific [Source:HGNC Symbol;Acc:HGNC:8780]

circRNA44	Uncharacterized protein [Source:UniProtKB/TrEMBL;Acc:W5NXR0]
circRNA440	insulin receptor [Source:HGNC Symbol;Acc:HGNC:6091]
circRNA441	insulin receptor [Source:HGNC Symbol;Acc:HGNC:6091]
circRNA442	Uncharacterized protein [Source:UniProtKB/TrEMBL;Acc:W5PDC0]
circRNA443	kinesin family member 3A [Source:HGNC Symbol;Acc:HGNC:6319]
circRNA444	solute carrier family 12 (sodium/potassium/chloride transporter), member 2 [Source:HGNC Symbol;Acc:HGNC:10911]
circRNA445	solute carrier family 12 (sodium/potassium/chloride transporter), member 2 [Source:HGNC Symbol;Acc:HGNC:10911]
circRNA446	proline-rich coiled-coil 1 [Source:HGNC Symbol;Acc:HGNC:28164]
circRNA447	NA
circRNA448	zinc finger protein 608 [Source:HGNC Symbol;Acc:HGNC:29238]
circRNA449	centrosomal protein 120kDa [Source:HGNC Symbol;Acc:HGNC:26690]
circRNA45	Uncharacterized protein [Source:UniProtKB/TrEMBL;Acc:W5PPP2]
circRNA450	centrosomal protein 120kDa [Source:HGNC Symbol;Acc:HGNC:26690]
circRNA451	hydroxysteroid (17-beta) dehydrogenase 4 [Source:HGNC Symbol;Acc:HGNC:5213]
circRNA452	polypyrimidine tract binding protein 1 [Source:HGNC Symbol;Acc:HGNC:9583]
circRNA453	SMAD family member 5 [Source:HGNC Symbol;Acc:HGNC:6771]
circRNA454	Uncharacterized protein [Source:UniProtKB/TrEMBL;Acc:W5Q1Q5]
circRNA455	family with sequence similarity 13, member B [Source:HGNC Symbol;Acc:HGNC:1335]
circRNA456	cell division cycle 23 [Source:HGNC Symbol;Acc:HGNC:1724]
circRNA457	Rho GTPase activating protein 26 [Source:HGNC Symbol;Acc:HGNC:17073]
circRNA458	SH3 domain containing ring finger 2 [Source:HGNC Symbol;Acc:HGNC:26299]
circRNA459	dihydropyrimidinase-like 3 [Source:HGNC Symbol;Acc:HGNC:3015]
circRNA46	NA
circRNA460	actin binding LIM protein family, member 3 [Source:HGNC Symbol;Acc:HGNC:29132]
circRNA461	peroxisome proliferator-activated receptor gamma, coactivator 1 beta [Source:HGNC Symbol;Acc:HGNC:30022]
circRNA462	N-deacetylase/N-sulfotransferase (heparan glucosaminyl) 1 [Source:HGNC Symbol;Acc:HGNC:7680]
circRNA463	TNFAIP3 interacting protein 1 [Source:HGNC Symbol;Acc:HGNC:16903]
circRNA464	GTPase activating protein (SH3 domain) binding protein 1 [Source:HGNC Symbol;Acc:HGNC:30292]
circRNA465	sarcoglycan, delta (35kDa dystrophin-associated glycoprotein) [Source:HGNC Symbol;Acc:HGNC:10807]
circRNA466	Uncharacterized protein [Source:UniProtKB/TrEMBL;Acc:W5PTG6]
circRNA467	zinc finger, FYVE domain containing 16 [Source:HGNC Symbol;Acc:HGNC:20756]
circRNA468	Ovis aries myocyte enhancer factor 2C (MEF2C), mRNA. [Source:RefSeq mRNA;Acc:NM_001159277]
circRNA469	leucyl/cystinyl aminopeptidase [Source:HGNC Symbol;Acc:HGNC:6656]
circRNA47	nuclear VCP-like [Source:HGNC Symbol;Acc:HGNC:8070]
circRNA470	diphosphoinositol pentakisphosphate kinase 2 [Source:HGNC Symbol;Acc:HGNC:29035]
circRNA471	mannosidase, alpha, class 2A, member 1 [Source:HGNC Symbol;Acc:HGNC:6824]
circRNA472	solute carrier family 25 (aspartate/glutamate carrier), member 13 [Source:HGNC Symbol;Acc:HGNC:10983]
circRNA473	histone deacetylase 9 [Source:HGNC Symbol;Acc:HGNC:14065]
circRNA474	histone deacetylase 9 [Source:HGNC Symbol;Acc:HGNC:14065]
circRNA475	N-acyl phosphatidylethanolamine phospholipase D [Source:HGNC Symbol;Acc:HGNC:21683]
circRNA476	Ovis aries growth factor receptor c-met (C-MET), mRNA. [Source:RefSeq mRNA;Acc:NM_001111071]
circRNA477	dedicator of cytokinesis 4 [Source:HGNC Symbol;Acc:HGNC:19192]
circRNA478	Uncharacterized protein [Source:UniProtKB/TrEMBL;Acc:W5PHR0]
circRNA479	Uncharacterized protein [Source:UniProtKB/TrEMBL;Acc:W5PHR0]
circRNA48	nuclear VCP-like [Source:HGNC Symbol;Acc:HGNC:8070]
circRNA480	Uncharacterized protein [Source:UniProtKB/TrEMBL;Acc:W5PL01]
circRNA481	Uncharacterized protein [Source:UniProtKB/TrEMBL;Acc:W5Q633]
circRNA482	tetraspanin 12 [Source:HGNC Symbol;Acc:HGNC:21641]
circRNA483	cadherin-like and PC-esterase domain containing 1 [Source:HGNC Symbol;Acc:HGNC:26159]
circRNA484	muskelin 1, intracellular mediator containing kelch motifs [Source:HGNC Symbol;Acc:HGNC:7109]
circRNA485	Ovis aries coiled-coil-helix-coiled-coil-helix domain containing 3 (CHCHD3), mRNA. [Source:RefSeq mRNA;Acc:NM_001126357]
circRNA486	chloride channel, voltage-sensitive 1 [Source:HGNC Symbol;Acc:HGNC:2019]
circRNA487	polypeptide N-acetylgalactosaminyltransferase 11 [Source:HGNC Symbol;Acc:HGNC:19875]
circRNA488	lysine (K)-specific methyltransferase 2C [Source:HGNC Symbol;Acc:HGNC:13726]
circRNA489	Uncharacterized protein [Source:UniProtKB/TrEMBL;Acc:W5P7H8]
circRNA49	cornichon family AMPA receptor auxiliary protein 4 [Source:HGNC Symbol;Acc:HGNC:25013]
circRNA490	RNA binding motif protein 33 [Source:HGNC Symbol;Acc:HGNC:27223]
circRNA491	RNA binding motif protein 33 [Source:HGNC Symbol;Acc:HGNC:27223]
circRNA492	limb development membrane protein 1 [Source:HGNC Symbol;Acc:HGNC:13243]
circRNA493	limb development membrane protein 1 [Source:HGNC Symbol;Acc:HGNC:13243]
circRNA494	ubiquitin protein ligase E3C [Source:HGNC Symbol;Acc:HGNC:16803]
circRNA495	DnaJ (Hsp40) homolog, subfamily B, member 6 [Source:HGNC Symbol;Acc:HGNC:14888]
circRNA496	euchromatic histone-lysine N-methyltransferase 1 [Source:HGNC Symbol;Acc:HGNC:24650]
circRNA497	euchromatic histone-lysine N-methyltransferase 1 [Source:HGNC Symbol;Acc:HGNC:24650]
circRNA498	olfactomedin 1 [Source:HGNC Symbol;Acc:HGNC:17187]
circRNA499	calmodulin regulated spectrin-associated protein 1 [Source:HGNC Symbol;Acc:HGNC:19946]
circRNA5	HECT, UBA and WWE domain containing 1, E3 ubiquitin protein ligase [Source:HGNC Symbol;Acc:HGNC:30892]
circRNA50	CDC42 binding protein kinase alpha (DMPK-like) [Source:HGNC Symbol;Acc:HGNC:1737]
circRNA500	tuberous sclerosis 1 [Source:HGNC Symbol;Acc:HGNC:12362]
circRNA501	adenylate kinase 8 [Source:HGNC Symbol;Acc:HGNC:26526]
circRNA502	nucleoporin 214kDa [Source:HGNC Symbol;Acc:HGNC:8064]
circRNA503	protein phosphatase 2A activator, regulatory subunit 4 [Source:HGNC Symbol;Acc:HGNC:9308]
circRNA504	Ovis aries SAP kinase-interacting protein 1 (SIN1), mRNA. [Source:RefSeq mRNA;Acc:NM_001009768]
circRNA505	GTPase activating protein and VPS9 domains 1 [Source:HGNC Symbol;Acc:HGNC:23375]
circRNA506	Rab9 effector protein with kelch motifs [Source:HGNC Symbol;Acc:HGNC:16896]
circRNA507	neuroblastoma amplified sequence [Source:HGNC Symbol;Acc:HGNC:15625]
circRNA508	neuroblastoma amplified sequence [Source:HGNC Symbol;Acc:HGNC:15625]
circRNA509	Uncharacterized protein [Source:UniProtKB/TrEMBL;Acc:W5QA98]
circRNA51	CDC42 binding protein kinase alpha (DMPK-like) [Source:HGNC Symbol;Acc:HGNC:1737]
circRNA510	Ovis aries hydroxyacyl-CoA dehydrogenase/3-ketoacyl-CoA thiolase/enoyl-CoA hydratase (trifunctional protein), alpha subunit (HADHA), nuclear gene encoding mitochondrial protein, mRNA.
circRNA511	CAP-GLY domain containing linker protein family, member 4 [Source:HGNC Symbol;Acc:HGNC:26108]
circRNA512	CAP-GLY domain containing linker protein family, member 4 [Source:HGNC Symbol;Acc:HGNC:26108]
circRNA513	AP2 associated kinase 1 [Source:HGNC Symbol;Acc:HGNC:19679]
circRNA514	glutamine--fructose-6-phosphate transaminase 1 [Source:HGNC Symbol;Acc:HGNC:4241]
circRNA515	Uncharacterized protein [Source:UniProtKB/TrEMBL;Acc:W5QF78]
circRNA516	UDP-glucose pyrophosphorylase 2 [Source:HGNC Symbol;Acc:HGNC:12527]
circRNA517	EH domain binding protein 1 [Source:HGNC Symbol;Acc:HGNC:29144]
circRNA518	ELMO/CED-12 domain containing 3 [Source:HGNC Symbol;Acc:HGNC:26158]
circRNA519	Ovis aries gamma-glutamyl carboxylase (GGCX), mRNA. [Source:RefSeq mRNA;Acc:NM_001009750]
circRNA52	heterogeneous nuclear ribonucleoprotein U (scaffold attachment factor A) [Source:HGNC Symbol;Acc:HGNC:5048]
circRNA520	receptor accessory protein 1 [Source:HGNC Symbol;Acc:HGNC:25786]
circRNA521	Uncharacterized protein [Source:UniProtKB/TrEMBL;Acc:W5QHN1]
circRNA522	required for meiotic nuclear division 5 homolog A (S. cerevisiae) [Source:HGNC Symbol;Acc:HGNC:25850]
circRNA523	SET and MYND domain containing 1 [Source:HGNC Symbol;Acc:HGNC:20986]
circRNA524	SET and MYND domain containing 1 [Source:HGNC Symbol;Acc:HGNC:20986]
circRNA525	Uncharacterized protein [Source:UniProtKB/TrEMBL;Acc:W5NQK6]
circRNA526	reticulon-4 [Source:RefSeq peptide;Acc:NP_001138650]
circRNA527	Uncharacterized protein [Source:UniProtKB/TrEMBL;Acc:W5P1T2]

circRNA528	protein kinase C, epsilon [Source:HGNC Symbol;Acc:HGNC:9401]
circRNA529	S1 RNA binding domain 1 [Source:HGNC Symbol;Acc:HGNC:25521]
circRNA53	v-akt murine thymoma viral oncogene homolog 3 [Source:HGNC Symbol;Acc:HGNC:393]
circRNA530	S1 RNA binding domain 1 [Source:HGNC Symbol;Acc:HGNC:25521]
circRNA531	S1 RNA binding domain 1 [Source:HGNC Symbol;Acc:HGNC:25521]
circRNA532	S1 RNA binding domain 1 [Source:HGNC Symbol;Acc:HGNC:25521]
circRNA533	striatin, calmodulin binding protein [Source:HGNC Symbol;Acc:HGNC:11424]
circRNA534	fasciculation and elongation protein zeta 2 (zygin II) [Source:HGNC Symbol;Acc:HGNC:3660]
circRNA535	cysteine rich transmembrane BMP regulator 1 (chordin-like) [Source:HGNC Symbol;Acc:HGNC:2359]
circRNA536	Uncharacterized protein [Source:UniProtKB/TrEMBL;Acc:W5PMG3]
circRNA537	dysferlin [Source:HGNC Symbol;Acc:HGNC:3097]
circRNA538	Alstrom syndrome 1 [Source:HGNC Symbol;Acc:HGNC:428]
circRNA539	transforming growth factor, beta receptor associated protein 1 [Source:HGNC Symbol;Acc:HGNC:16836]
circRNA54	vacuolar protein sorting 13 homolog D (S. cerevisiae) [Source:HGNC Symbol;Acc:HGNC:23595]
circRNA540	major facilitator superfamily domain containing 9 [Source:HGNC Symbol;Acc:HGNC:28158]
circRNA541	solute carrier family 9, subfamily A (NHE2, cation proton antiporter 2), member 2 [Source:HGNC Symbol;Acc:HGNC:11072]
circRNA542	neuronal PAS domain protein 2 [Source:HGNC Symbol;Acc:HGNC:7895]
circRNA543	REV1, polymerase (DNA directed) [Source:HGNC Symbol;Acc:HGNC:14060]
circRNA544	REV1, polymerase (DNA directed) [Source:HGNC Symbol;Acc:HGNC:14060]
circRNA545	ankyrin repeat domain 23 [Source:HGNC Symbol;Acc:HGNC:24470]
circRNA546	KAT8 regulatory NSL complex subunit 3 [Source:HGNC Symbol;Acc:HGNC:25473]
circRNA547	cysteine and glycine-rich protein 2 [Source:HGNC Symbol;Acc:HGNC:2470]
circRNA548	POC1 centriolar protein B [Source:HGNC Symbol;Acc:HGNC:30836]
circRNA549	RISC-loading complex subunit TARBP2 [Source:UniProtKB/TrEMBL;Acc:W5Q4L1]
circRNA55	chloride channel, voltage-sensitive 6 [Source:HGNC Symbol;Acc:HGNC:2024]
circRNA550	LETM1 domain containing 1 [Source:HGNC Symbol;Acc:HGNC:24241]
circRNA551	La ribonucleoprotein domain family, member 4 [Source:HGNC Symbol;Acc:HGNC:24320]
circRNA552	glycerol-3-phosphate dehydrogenase 1 (soluble) [Source:HGNC Symbol;Acc:HGNC:4455]
circRNA553	KAT8 regulatory NSL complex subunit 2 [Source:HGNC Symbol;Acc:HGNC:26024]
circRNA554	RNA polymerase II associated protein 3 [Source:HGNC Symbol;Acc:HGNC:26151]
circRNA555	AT rich interactive domain 2 (ARID, RFX-like) [Source:HGNC Symbol;Acc:HGNC:18037]
circRNA556	anoctamin 6 [Source:HGNC Symbol;Acc:HGNC:25240]
circRNA557	periphilin 1 [Source:HGNC Symbol;Acc:HGNC:19369]
circRNA558	glucoside xylosyltransferase 1 [Source:HGNC Symbol;Acc:HGNC:27482]
circRNA559	Uncharacterized protein [Source:UniProtKB/TrEMBL;Acc:W5QG97]
circRNA56	calsyntenin 1 [Source:HGNC Symbol;Acc:HGNC:17447]
circRNA560	cleavage and polyadenylation specific factor 6, 68kDa [Source:HGNC Symbol;Acc:HGNC:13871]
circRNA561	RAP1B, member of RAS oncogene family [Source:HGNC Symbol;Acc:HGNC:9857]
circRNA562	LEM domain containing 3 [Source:HGNC Symbol;Acc:HGNC:28887]
circRNA563	SLIT-ROBO Rho GTPase activating protein 1 [Source:HGNC Symbol;Acc:HGNC:17382]
circRNA564	integrin, alpha 7 [Source:HGNC Symbol;Acc:HGNC:6143]
circRNA565	Uncharacterized protein [Source:UniProtKB/TrEMBL;Acc:W5PUE6]
circRNA566	myosin binding protein C, slow type [Source:HGNC Symbol;Acc:HGNC:7549]
circRNA567	myosin binding protein C, slow type [Source:HGNC Symbol;Acc:HGNC:7549]
circRNA568	myosin binding protein C, slow type [Source:HGNC Symbol;Acc:HGNC:7549]
circRNA569	myosin binding protein C, slow type [Source:HGNC Symbol;Acc:HGNC:7549]
circRNA57	arginine-glutamic acid dipeptide (RE) repeats [Source:HGNC Symbol;Acc:HGNC:9965]
circRNA570	myosin binding protein C, slow type [Source:HGNC Symbol;Acc:HGNC:7549]
circRNA571	thioredoxin reductase 1 [Source:HGNC Symbol;Acc:HGNC:12437]
circRNA572	NUAK family, SNF1-like kinase, 1 [Source:HGNC Symbol;Acc:HGNC:14311]
circRNA573	like-glycosyltransferase [Source:HGNC Symbol;Acc:HGNC:6511]
circRNA574	ATP-binding cassette, sub-family C (CFTR/MRP), member 9 [Source:HGNC Symbol;Acc:HGNC:60]
circRNA575	ATP-binding cassette, sub-family C (CFTR/MRP), member 9 [Source:HGNC Symbol;Acc:HGNC:60]
circRNA576	pyridine nucleotide-disulphide oxidoreductase domain 1 [Source:HGNC Symbol;Acc:HGNC:26162]
circRNA577	dual specificity phosphatase 16 [Source:HGNC Symbol;Acc:HGNC:17909]
circRNA578	low density lipoprotein receptor-related protein 6 [Source:HGNC Symbol;Acc:HGNC:6698]
circRNA579	Y box binding protein 3 [Source:HGNC Symbol;Acc:HGNC:2428]
circRNA58	DNA-damage inducible 1 homolog 2 (S. cerevisiae) [Source:HGNC Symbol;Acc:HGNC:24578]
circRNA580	complement component 1, s subcomponent [Source:HGNC Symbol;Acc:HGNC:1247]
circRNA581	intermediate filament family orphan 1 [Source:HGNC Symbol;Acc:HGNC:24970]
circRNA582	von Willebrand factor [Source:HGNC Symbol;Acc:HGNC:12726]
circRNA583	Uncharacterized protein [Source:UniProtKB/TrEMBL;Acc:W5PI58]
circRNA584	ELKS/RAB6-interacting/CAST family member 1 [Source:HGNC Symbol;Acc:HGNC:17072]
circRNA585	microtubule associated monooxygenase, calponin and LIM domain containing 3 [Source:HGNC Symbol;Acc:HGNC:24694]
circRNA586	microtubule associated monooxygenase, calponin and LIM domain containing 3 [Source:HGNC Symbol;Acc:HGNC:24694]
circRNA587	Uncharacterized protein [Source:UniProtKB/TrEMBL;Acc:W5Q780]
circRNA588	hydroxysteroid dehydrogenase like 2 [Source:HGNC Symbol;Acc:HGNC:18572]
circRNA589	A kinase (PRKA) anchor protein 2 [Source:HGNC Symbol;Acc:HGNC:372]
circRNA59	RAB GTPase activating protein 1-like [Source:HGNC Symbol;Acc:HGNC:24663]
circRNA590	A kinase (PRKA) anchor protein 2 [Source:HGNC Symbol;Acc:HGNC:372]
circRNA591	zinc finger protein 462 [Source:HGNC Symbol;Acc:HGNC:21684]
circRNA592	ATP-binding cassette, sub-family A (ABC1), member 1 [Source:HGNC Symbol;Acc:HGNC:29]
circRNA593	family with sequence similarity 120A [Source:HGNC Symbol;Acc:HGNC:13247]
circRNA594	ubiquitin associated protein 2 [Source:HGNC Symbol;Acc:HGNC:14185]
circRNA595	potassium channel tetramerization domain containing 9 [Source:HGNC Symbol;Acc:HGNC:22401]
circRNA596	protein phosphatase 3, catalytic subunit, gamma isozyme [Source:HGNC Symbol;Acc:HGNC:9316]
circRNA597	collagen, type XV, alpha 1 [Source:HGNC Symbol;Acc:HGNC:2192]
circRNA598	Uncharacterized protein [Source:UniProtKB/TrEMBL;Acc:W5PS49]
circRNA599	dedicator of cytokinesis 8 [Source:HGNC Symbol;Acc:HGNC:19191]
circRNA6	lysine (K)-specific demethylase 5C [Source:HGNC Symbol;Acc:HGNC:11114]
circRNA60	ring finger and WD repeat domain 2, E3 ubiquitin protein ligase [Source:HGNC Symbol;Acc:HGNC:17440]
circRNA600	regulatory factor X, 3 (influences HLA class II expression) [Source:HGNC Symbol;Acc:HGNC:9984]
circRNA601	RAB6A GEF complex partner 1 [Source:HGNC Symbol;Acc:HGNC:17686]
circRNA602	Uncharacterized protein [Source:UniProtKB/TrEMBL;Acc:W5PW64]
circRNA603	multiple PDZ domain protein [Source:HGNC Symbol;Acc:HGNC:7208]
circRNA604	nuclear factor I/B [Source:HGNC Symbol;Acc:HGNC:7785]
circRNA605	basonuclin 2 [Source:HGNC Symbol;Acc:HGNC:30988]
circRNA606	exostosin-like glycosyltransferase 3 [Source:HGNC Symbol;Acc:HGNC:3518]
circRNA607	polypeptide N-acetylgalactosaminyltransferase 7 [Source:HGNC Symbol;Acc:HGNC:4129]
circRNA608	palladin, cytoskeletal associated protein [Source:HGNC Symbol;Acc:HGNC:17068]
circRNA609	palladin, cytoskeletal associated protein [Source:HGNC Symbol;Acc:HGNC:17068]
circRNA61	laminin, gamma 1 (formerly LAMB2) [Source:HGNC Symbol;Acc:HGNC:6492]
circRNA610	Uncharacterized protein [Source:UniProtKB/TrEMBL;Acc:W5Q1Z5]
circRNA611	nucleoporin 35kDa [Source:HGNC Symbol;Acc:HGNC:29797]
circRNA612	zinc finger protein 385B [Source:HGNC Symbol;Acc:HGNC:26332]
circRNA613	SEC14 and spectrin domains 1 [Source:HGNC Symbol;Acc:HGNC:18379]
circRNA614	SEC14 and spectrin domains 1 [Source:HGNC Symbol;Acc:HGNC:18379]
circRNA615	titin [Source:HGNC Symbol;Acc:HGNC:12403]

circRNA616	titin [Source:HGNC Symbol;Acc:HGNC:12403]
circRNA617	titin [Source:HGNC Symbol;Acc:HGNC:12403]
circRNA618	titin [Source:HGNC Symbol;Acc:HGNC:12403]
circRNA619	titin [Source:HGNC Symbol;Acc:HGNC:12403]
circRNA62	collagen beta(1-O)galactosyltransferase 2 [Source:HGNC Symbol;Acc:HGNC:16790]
circRNA620	titin [Source:HGNC Symbol;Acc:HGNC:12403]
circRNA621	titin [Source:HGNC Symbol;Acc:HGNC:12403]
circRNA622	titin [Source:HGNC Symbol;Acc:HGNC:12403]
circRNA623	titin [Source:HGNC Symbol;Acc:HGNC:12403]
circRNA624	titin [Source:HGNC Symbol;Acc:HGNC:12403]
circRNA625	protein kinase, interferon-inducible double stranded RNA dependent activator [Source:HGNC Symbol;Acc:HGNC:9438]
circRNA626	Uncharacterized protein [Source:UniProtKB/TrEMBL;Acc:W5NRF7]
circRNA627	cytochrome b reductase 1 [Source:HGNC Symbol;Acc:HGNC:20797]
circRNA628	DDB1 and CUL4 associated factor 17 [Source:HGNC Symbol;Acc:HGNC:25784]
circRNA629	ubiquitin protein ligase E3 component n-recognin 3 (putative) [Source:HGNC Symbol;Acc:HGNC:30467]
circRNA63	Uncharacterized protein [Source:UniProtKB/TrEMBL;Acc:W5PHG6]
circRNA630	formin-like 2 [Source:HGNC Symbol;Acc:HGNC:18267]
circRNA631	formin-like 2 [Source:HGNC Symbol;Acc:HGNC:18267]
circRNA632	signal transducing adaptor molecule (SH3 domain and ITAM motif) 2 [Source:HGNC Symbol;Acc:HGNC:11358]
circRNA633	nebulin [Source:HGNC Symbol;Acc:HGNC:7720]
circRNA634	Ovis aries activin A receptor, type IIA (ACVR2A), mRNA. [Source:RefSeq mRNA;Acc:NM_001009293]
circRNA635	RAB3 GTPase activating protein subunit 1 (catalytic) [Source:HGNC Symbol;Acc:HGNC:17063]
circRNA636	v-ral simian leukemia viral oncogene homolog B [Source:HGNC Symbol;Acc:HGNC:9840]
circRNA637	cytoplasmic linker associated protein 1 [Source:HGNC Symbol;Acc:HGNC:17088]
circRNA638	cytoplasmic linker associated protein 1 [Source:HGNC Symbol;Acc:HGNC:17088]
circRNA639	boule-like RNA-binding protein [Source:HGNC Symbol;Acc:HGNC:14273]
circRNA64	hedgehog acyltransferase [Source:HGNC Symbol;Acc:HGNC:18270]
circRNA640	Uncharacterized protein [Source:UniProtKB/TrEMBL;Acc:W5QCV7]
circRNA641	Uncharacterized protein [Source:UniProtKB/TrEMBL;Acc:W5QCV7]
circRNA642	acyl-CoA dehydrogenase, long chain [Source:HGNC Symbol;Acc:HGNC:88]
circRNA643	Ovis aries insulin-like growth factor binding protein 5 (IGFBP5), mRNA. [Source:RefSeq mRNA;Acc:NM_001129733]
circRNA644	phospholipase C, delta 4 [Source:HGNC Symbol;Acc:HGNC:9062]
circRNA645	thyroid hormone receptor interactor 12 [Source:HGNC Symbol;Acc:HGNC:12306]
circRNA646	ribosomal protein S6 kinase, 90kDa, polypeptide 1 [Source:HGNC Symbol;Acc:HGNC:10430]
circRNA647	tripartite motif containing 63, E3 ubiquitin protein ligase [Source:HGNC Symbol;Acc:HGNC:16007]
circRNA648	mitochondrial fission regulator 1-like [Source:HGNC Symbol;Acc:HGNC:28836]
circRNA649	mitochondrial fission regulator 1-like [Source:HGNC Symbol;Acc:HGNC:28836]
circRNA65	DENN/MADD domain containing 1B [Source:HGNC Symbol;Acc:HGNC:28404]
circRNA650	myomesin 3 [Source:HGNC Symbol;Acc:HGNC:26679]
circRNA651	myomesin 3 [Source:HGNC Symbol;Acc:HGNC:26679]
circRNA652	lysine (K)-specific demethylase 1A [Source:HGNC Symbol;Acc:HGNC:29079]
circRNA653	eukaryotic translation initiation factor 4 gamma, 3 [Source:HGNC Symbol;Acc:HGNC:3298]
circRNA654	eukaryotic translation initiation factor 4 gamma, 3 [Source:HGNC Symbol;Acc:HGNC:3298]
circRNA655	ciliary rootlet coiled-coil, rootletin [Source:HGNC Symbol;Acc:HGNC:21299]
circRNA656	F-box protein 42 [Source:HGNC Symbol;Acc:HGNC:29249]
circRNA657	THAP domain containing 4 [Source:HGNC Symbol;Acc:HGNC:23187]
circRNA658	TNF receptor-associated factor 3 interacting protein 1 [Source:HGNC Symbol;Acc:HGNC:17861]
circRNA659	leucine rich repeat (in FLII) interacting protein 1 [Source:HGNC Symbol;Acc:HGNC:6702]
circRNA66	amyloid beta precursor protein (cytoplasmic tail) binding protein 2 [Source:HGNC Symbol;Acc:HGNC:622]
circRNA660	leucine rich repeat (in FLII) interacting protein 1 [Source:HGNC Symbol;Acc:HGNC:6702]
circRNA661	ArfGAP with GTPase domain, ankyrin repeat and PH domain 1 [Source:HGNC Symbol;Acc:HGNC:16922]
circRNA662	GRB10 interacting GYF protein 2 [Source:HGNC Symbol;Acc:HGNC:11960]
circRNA663	KIAA0319-like [Source:HGNC Symbol;Acc:HGNC:30071]
circRNA664	four and a half LIM domains 3 [Source:HGNC Symbol;Acc:HGNC:3704]
circRNA665	sex comb on midleg homolog 1 (Drosophila) [Source:HGNC Symbol;Acc:HGNC:19003]
circRNA666	leucine proline-enriched proteoglycan (leprecan) 1 [Source:HGNC Symbol;Acc:HGNC:19316]
circRNA667	ERI1 exoribonuclease family member 3 [Source:HGNC Symbol;Acc:HGNC:17276]
circRNA668	Uncharacterized protein [Source:UniProtKB/TrEMBL;Acc:W5NVC6]
circRNA669	FGGY carbohydrate kinase domain containing [Source:HGNC Symbol;Acc:HGNC:25610]
circRNA67	Ubiquitin carboxyl-terminal hydrolase [Source:UniProtKB/TrEMBL;Acc:W5Q884]
circRNA670	FGGY carbohydrate kinase domain containing [Source:HGNC Symbol;Acc:HGNC:25610]
circRNA671	TM2 domain containing 1 [Source:HGNC Symbol;Acc:HGNC:24142]
circRNA672	autophagy related 4C, cysteine peptidase [Source:HGNC Symbol;Acc:HGNC:16040]
circRNA673	phosphoglucomutase 1 [Source:HGNC Symbol;Acc:HGNC:8905]
circRNA674	receptor tyrosine kinase-like orphan receptor 1 [Source:HGNC Symbol;Acc:HGNC:10256]
circRNA675	protein kinase N2 [Source:HGNC Symbol;Acc:HGNC:9406]
circRNA676	transmembrane emp24 protein transport domain containing 5 [Source:HGNC Symbol;Acc:HGNC:24251]
circRNA677	Uncharacterized protein [Source:UniProtKB/TrEMBL;Acc:W5Q7V0]
circRNA678	hippocampus abundant transcript 1 [Source:HGNC Symbol;Acc:HGNC:23363]
circRNA679	Ovis aries solute carrier family 30 (zinc transporter), member 7 (SLC30A7), mRNA. [Source:RefSeq mRNA;Acc:NM_001163598]
circRNA68	Ovis aries acetyl-CoA carboxylase alpha (ACACA), mRNA. [Source:RefSeq mRNA;Acc:NM_001009256]
circRNA680	round spermatid basic protein 1 [Source:HGNC Symbol;Acc:HGNC:25642]
circRNA681	adenosine monophosphate deaminase 1 [Source:HGNC Symbol;Acc:HGNC:468]
circRNA682	ganglioside induced differentiation associated protein 2 [Source:HGNC Symbol;Acc:HGNC:18010]
circRNA683	T-box 15 [Source:HGNC Symbol;Acc:HGNC:11594]
circRNA684	phosphodiesterase 4D interacting protein [Source:HGNC Symbol;Acc:HGNC:15580]
circRNA685	phosphodiesterase 4D interacting protein [Source:HGNC Symbol;Acc:HGNC:15580]
circRNA686	regulation of nuclear pre-mRNA domain containing 2 [Source:HGNC Symbol;Acc:HGNC:29039]
circRNA687	vacuolar protein sorting 72 homolog (S. cerevisiae) [Source:HGNC Symbol;Acc:HGNC:11644]
circRNA688	phosphatidylinositol 4-kinase, catalytic, beta [Source:HGNC Symbol;Acc:HGNC:8984]
circRNA689	pogo transposable element with ZNF domain [Source:HGNC Symbol;Acc:HGNC:18801]
circRNA69	ring finger and FYVE-like domain containing E3 ubiquitin protein ligase [Source:HGNC Symbol;Acc:HGNC:24821]
circRNA690	sorting nexin family member 27 [Source:HGNC Symbol;Acc:HGNC:20073]
circRNA691	activating transcription factor 6 [Source:HGNC Symbol;Acc:HGNC:791]
circRNA692	DDB1 and CUL4 associated factor 6 [Source:HGNC Symbol;Acc:HGNC:30002]
circRNA693	DDB1 and CUL4 associated factor 6 [Source:HGNC Symbol;Acc:HGNC:30002]
circRNA694	DDB1 and CUL4 associated factor 6 [Source:HGNC Symbol;Acc:HGNC:30002]
circRNA695	DDB1 and CUL4 associated factor 6 [Source:HGNC Symbol;Acc:HGNC:30002]
circRNA696	SON DNA binding protein [Source:HGNC Symbol;Acc:HGNC:11183]
circRNA697	transmembrane protein 50B [Source:HGNC Symbol;Acc:HGNC:1280]
circRNA698	N-6 adenine-specific DNA methyltransferase 1 (putative) [Source:HGNC Symbol;Acc:HGNC:16021]
circRNA699	Amyloid beta A4 protein Soluble APP-beta CTF-alpha Beta-amyloid protein 42 Beta-amyloid protein 40 Gamma-secretase C-terminal fragment 59 Gamma-secretase C-terminal fragment 57
circRNA7	phosphorylase kinase, alpha 1 (muscle) [Source:HGNC Symbol;Acc:HGNC:8925]
circRNA70	proteasome (prosome, macropain) 26S subunit, non-ATPase, 11 [Source:HGNC Symbol;Acc:HGNC:9556]
circRNA700	ubiquitin specific peptidase 25 [Source:HGNC Symbol;Acc:HGNC:12624]
circRNA701	ADP-ribosylation factor-like 13B [Source:HGNC Symbol;Acc:HGNC:25419]
circRNA702	TBC1 domain family, member 23 [Source:HGNC Symbol;Acc:HGNC:25622]
circRNA703	bobby sox homolog (Drosophila) [Source:HGNC Symbol;Acc:HGNC:14422]

circRNA704	DAZ interacting zinc finger protein 3 [Source:HGNC Symbol;Acc:HGNC:30938]
circRNA705	DAZ interacting zinc finger protein 3 [Source:HGNC Symbol;Acc:HGNC:30938]
circRNA706	CD200 molecule [Source:HGNC Symbol;Acc:HGNC:7203]
circRNA707	disrupted in renal carcinoma 2 [Source:HGNC Symbol;Acc:HGNC:16628]
circRNA708	disrupted in renal carcinoma 2 [Source:HGNC Symbol;Acc:HGNC:16628]
circRNA709	leucine-rich repeats and calponin homology (CH) domain containing 3 [Source:HGNC Symbol;Acc:HGNC:28637]
circRNA71	SUZ12 polycomb repressive complex 2 subunit [Source:HGNC Symbol;Acc:HGNC:17101]
circRNA710	p21 protein (Cdc42/Rac)-activated kinase 2 [Source:HGNC Symbol;Acc:HGNC:8591]
circRNA711	discs, large homolog 1 (Drosophila) [Source:HGNC Symbol;Acc:HGNC:2900]
circRNA712	discs, large homolog 1 (Drosophila) [Source:HGNC Symbol;Acc:HGNC:2900]
circRNA713	HRAS-like suppressor [Source:HGNC Symbol;Acc:HGNC:14922]
circRNA714	presenilin associated, rhomboid-like [Source:HGNC Symbol;Acc:HGNC:18253]
circRNA715	DCN1, defective in cullin neddylation 1, domain containing 1 [Source:HGNC Symbol;Acc:HGNC:18184]
circRNA716	ubiquitin specific peptidase 13 (isopeptidase T-3) [Source:HGNC Symbol;Acc:HGNC:12611]
circRNA717	ubiquitin specific peptidase 13 (isopeptidase T-3) [Source:HGNC Symbol;Acc:HGNC:12611]
circRNA718	transducin (beta)-like 1 X-linked receptor 1 [Source:HGNC Symbol;Acc:HGNC:29529]
circRNA719	fibronectin type III domain containing 3B [Source:HGNC Symbol;Acc:HGNC:24670]
circRNA72	UTP6, small subunit (SSU) processome component, homolog (yeast) [Source:HGNC Symbol;Acc:HGNC:18279]
circRNA720	fibronectin type III domain containing 3B [Source:HGNC Symbol;Acc:HGNC:24670]
circRNA721	phospholipase D1, phosphatidylcholine-specific [Source:HGNC Symbol;Acc:HGNC:9067]
circRNA722	TRAF2 and NCK interacting kinase [Source:HGNC Symbol;Acc:HGNC:30765]
circRNA723	guanine monphosphate synthase [Source:HGNC Symbol;Acc:HGNC:4378]
circRNA724	cytoplasmic protein NCK1 [Source:RefSeq peptide;Acc:NP_001159671]
circRNA725	protein phosphatase 2, regulatory subunit B'', alpha [Source:HGNC Symbol;Acc:HGNC:9307]
circRNA726	protein phosphatase 2, regulatory subunit B'', alpha [Source:HGNC Symbol;Acc:HGNC:9307]
circRNA727	kyphoscoliosis peptidase [Source:HGNC Symbol;Acc:HGNC:26576]
circRNA728	NIMA-related kinase 11 [Source:HGNC Symbol;Acc:HGNC:18593]
circRNA729	SH3 domain binding glutamate-rich protein [Source:HGNC Symbol;Acc:HGNC:10822]
circRNA73	SMG6 nonsense mediated mRNA decay factor [Source:HGNC Symbol;Acc:HGNC:17809]
circRNA730	ubiquitin-conjugating enzyme E2G 2 [Source:HGNC Symbol;Acc:HGNC:12483]
circRNA731	minichromosome maintenance complex component 3 associated protein [Source:HGNC Symbol;Acc:HGNC:6946]
circRNA732	pericentrin [Source:HGNC Symbol;Acc:HGNC:16068]
circRNA733	protein arginine methyltransferase 2 [Source:HGNC Symbol;Acc:HGNC:5186]
circRNA734	dopey family member 2 [Source:HGNC Symbol;Acc:HGNC:1291]
circRNA735	tetratricopeptide repeat domain 3 [Source:HGNC Symbol;Acc:HGNC:12393]
circRNA736	dual-specificity tyrosine-(Y)-phosphorylation regulated kinase 1A [Source:HGNC Symbol;Acc:HGNC:3091]
circRNA737	dual-specificity tyrosine-(Y)-phosphorylation regulated kinase 1A [Source:HGNC Symbol;Acc:HGNC:3091]
circRNA738	K(lysine) acetyltransferase 2B [Source:HGNC Symbol;Acc:HGNC:8638]
circRNA739	LMBR1 domain containing 1 [Source:HGNC Symbol;Acc:HGNC:23038]
circRNA74	XIAP associated factor 1 [Source:HGNC Symbol;Acc:HGNC:30932]
circRNA740	F-box and leucine-rich repeat protein 6 [Source:HGNC Symbol;Acc:HGNC:13603]
circRNA741	DENN/MADD domain containing 3 [Source:HGNC Symbol;Acc:HGNC:29134]
circRNA742	protein tyrosine kinase 2 [Source:HGNC Symbol;Acc:HGNC:9611]
circRNA743	scaffolding protein involved in DNA repair [Source:HGNC Symbol;Acc:HGNC:28971]
circRNA744	protein kinase, DNA-activated, catalytic polypeptide [Source:HGNC Symbol;Acc:HGNC:9413]
circRNA745	trimethylguanosine synthase 1 [Source:HGNC Symbol;Acc:HGNC:17843]
circRNA746	centrosome and spindle pole associated protein 1 [Source:HGNC Symbol;Acc:HGNC:26193]
circRNA747	phosphatidylinositol-3,4,5-trisphosphate-dependent Rac exchange factor 2 [Source:HGNC Symbol;Acc:HGNC:22950]
circRNA748	solute carrier organic anion transporter family, member 5A1 [Source:HGNC Symbol;Acc:HGNC:19046]
circRNA749	solute carrier organic anion transporter family, member 5A1 [Source:HGNC Symbol;Acc:HGNC:19046]
circRNA75	rabaptin, RAB GTPase binding effector protein 1 [Source:HGNC Symbol;Acc:HGNC:17677]
circRNA750	EYA transcriptional coactivator and phosphatase 1 [Source:HGNC Symbol;Acc:HGNC:3519]
circRNA751	staufen double-stranded RNA binding protein 2 [Source:HGNC Symbol;Acc:HGNC:11371]
circRNA752	zinc finger protein 704 [Source:HGNC Symbol;Acc:HGNC:32291]
circRNA753	eukaryotic translation initiation factor 3, subunit E [Source:HGNC Symbol;Acc:HGNC:3277]
circRNA754	vacuolar protein sorting 13 homolog B (yeast) [Source:HGNC Symbol;Acc:HGNC:2183]
circRNA755	NADH dehydrogenase (ubiquinone) complex I, assembly factor 6 [Source:HGNC Symbol;Acc:HGNC:28625]
circRNA756	KIAA1429 [Source:HGNC Symbol;Acc:HGNC:24500]
circRNA757	leucine rich repeat containing 69 [Source:HGNC Symbol;Acc:HGNC:34303]
circRNA758	leucine rich repeat containing 69 [Source:HGNC Symbol;Acc:HGNC:34303]
circRNA759	receptor-interacting serine-threonine kinase 2 [Source:HGNC Symbol;Acc:HGNC:10020]
circRNA76	centrobin, centrosomal BRCA2 interacting protein [Source:HGNC Symbol;Acc:HGNC:29616]
circRNA760	SUMO1/sentrin specific peptidase 6 [Source:HGNC Symbol;Acc:HGNC:20944]
circRNA761	myosin VI [Source:HGNC Symbol;Acc:HGNC:7605]
circRNA762	triadin [Source:HGNC Symbol;Acc:HGNC:12261]
circRNA763	triadin [Source:HGNC Symbol;Acc:HGNC:12261]
circRNA764	discoidin, CUB and LCCL domain containing 1 [Source:HGNC Symbol;Acc:HGNC:21479]
circRNA765	sine oculis binding protein homolog (Drosophila) [Source:HGNC Symbol;Acc:HGNC:29256]
circRNA766	HECT domain and ankyrin repeat containing E3 ubiquitin protein ligase 1 [Source:HGNC Symbol;Acc:HGNC:21033]
circRNA767	HECT domain and ankyrin repeat containing E3 ubiquitin protein ligase 1 [Source:HGNC Symbol;Acc:HGNC:21033]
circRNA768	HECT domain and ankyrin repeat containing E3 ubiquitin protein ligase 1 [Source:HGNC Symbol;Acc:HGNC:21033]
circRNA769	PNN-interacting serine/arginine-rich protein [Source:HGNC Symbol;Acc:HGNC:21222]
circRNA77	Uncharacterized protein [Source:UniProtKB/TrEMBL;Acc:W5PT09]
circRNA770	BTB and CNC homology 1, basic leucine zipper transcription factor 2 [Source:HGNC Symbol;Acc:HGNC:14078]
circRNA771	MDN1, midasin homolog (yeast) [Source:HGNC Symbol;Acc:HGNC:18302]
circRNA772	MDN1, midasin homolog (yeast) [Source:HGNC Symbol;Acc:HGNC:18302]
circRNA773	zinc finger protein 292 [Source:HGNC Symbol;Acc:HGNC:18410]
circRNA774	laminin, alpha 2 [Source:HGNC Symbol;Acc:HGNC:6482]
circRNA775	erythrocyte membrane protein band 4.1-like 2 [Source:HGNC Symbol;Acc:HGNC:3379]
circRNA776	solute carrier family 2 (facilitated glucose transporter), member 12 [Source:HGNC Symbol;Acc:HGNC:18067]
circRNA777	fucosidase, alpha-L- 2, plasma [Source:HGNC Symbol;Acc:HGNC:4008]
circRNA778	utrophin [Source:HGNC Symbol;Acc:HGNC:12635]
circRNA779	utrophin [Source:HGNC Symbol;Acc:HGNC:12635]
circRNA78	mbt domain containing 1 [Source:HGNC Symbol;Acc:HGNC:19866]
circRNA780	utrophin [Source:HGNC Symbol;Acc:HGNC:12635]
circRNA781	spectrin repeat containing, nuclear envelope 1 [Source:HGNC Symbol;Acc:HGNC:17089]
circRNA782	spectrin repeat containing, nuclear envelope 1 [Source:HGNC Symbol;Acc:HGNC:17089]
circRNA783	AT rich interactive domain 1B (SWI1-like) [Source:HGNC Symbol;Acc:HGNC:18040]
circRNA784	AT rich interactive domain 1B (SWI1-like) [Source:HGNC Symbol;Acc:HGNC:18040]
circRNA785	AT rich interactive domain 1B (SWI1-like) [Source:HGNC Symbol;Acc:HGNC:18040]
circRNA786	insulin-like growth factor 2 receptor [Source:HGNC Symbol;Acc:HGNC:5467]
circRNA787	QKI, KH domain containing, RNA binding [Source:HGNC Symbol;Acc:HGNC:21100]
circRNA788	QKI, KH domain containing, RNA binding [Source:HGNC Symbol;Acc:HGNC:21100]
circRNA79	RAB5C, member RAS oncogene family [Source:HGNC Symbol;Acc:HGNC:9785]
circRNA8	Ovis aries coagulation factor VIII, procoagulant component (F8), mRNA. [Source:RefSeq mRNA;Acc:NM_001172550]
circRNA80	ATPase, H+ transporting, lysosomal V0 subunit a1 [Source:HGNC Symbol;Acc:HGNC:865]
circRNA81	DEAH (Asp-Glu-Ala-His) box polypeptide 8 [Source:HGNC Symbol;Acc:HGNC:2749]
circRNA82	KAT8 regulatory NSL complex subunit 1 [Source:HGNC Symbol;Acc:HGNC:24565]

circRNA83	mitogen-activated protein kinase kinase kinase 3 [Source:HGNC Symbol;Acc:HGNC:6855]
circRNA84	SWI/SNF related, matrix associated, actin dependent regulator of chromatin, subfamily d, member 2 [Source:HGNC Symbol;Acc:HGNC:11107]
circRNA85	centrosomal protein 95kDa [Source:HGNC Symbol;Acc:HGNC:25141]
circRNA86	Protein disulfide-isomerase [Source:UniProtKB/TrEMBL;Acc:W5Q9H2]
circRNA87	BAI1-associated protein 2 [Source:HGNC Symbol;Acc:HGNC:947]
circRNA88	phosphoribosyl pyrophosphate synthetase-associated protein 1 [Source:HGNC Symbol;Acc:HGNC:9466]
circRNA89	signal recognition particle 68kDa [Source:HGNC Symbol;Acc:HGNC:11302]
circRNA9	WD repeat domain 44 [Source:HGNC Symbol;Acc:HGNC:30512]
circRNA90	tudor domain containing 3 [Source:HGNC Symbol;Acc:HGNC:20612]
circRNA91	SGT1, suppressor of G2 allele of SKP1 (S. cerevisiae) [Source:HGNC Symbol;Acc:HGNC:16987]
circRNA92	E74-like factor 1 (ets domain transcription factor) [Source:HGNC Symbol;Acc:HGNC:3316]
circRNA93	von Willebrand factor A domain containing 8 [Source:HGNC Symbol;Acc:HGNC:29071]
circRNA94	general transcription factor IIF, polypeptide 2, 30kDa [Source:HGNC Symbol;Acc:HGNC:4653]
circRNA95	Uncharacterized protein [Source:UniProtKB/TrEMBL;Acc:W5PCL3]
circRNA96	component of oligomeric golgi complex 3 [Source:HGNC Symbol;Acc:HGNC:18619]
circRNA97	succinate-CoA ligase, ADP-forming, beta subunit [Source:HGNC Symbol;Acc:HGNC:11448]
circRNA98	NHL repeat containing 3 [Source:HGNC Symbol;Acc:HGNC:33751]
circRNA99	SMAD family member 9 [Source:HGNC Symbol;Acc:HGNC:6774]

Accession	KEGG
ciRNA1	NA
ciRNA10	NA
ciRNA11	NA
ciRNA12	NA
ciRNA13	04710(Circadian rhythm - mammal)
ciRNA14	04710(Circadian rhythm - mammal)
ciRNA15	NA
ciRNA16	NA
ciRNA17	NA
ciRNA18	NA
ciRNA19	NA
ciRNA2	NA
ciRNA20	NA
ciRNA21	NA
ciRNA22	NA
ciRNA23	00562(Inositol phosphate metabolism);04070(Phosphatidylinositol signaling system)
ciRNA24	NA
ciRNA25	NA
ciRNA26	03022(Basal transcription factors)
ciRNA27	NA
ciRNA28	04144(Endocytosis)
ciRNA29	04110(Cell cycle)
ciRNA3	NA
ciRNA30	NA
ciRNA31	NA
ciRNA32	NA
ciRNA33	NA
ciRNA34	NA
ciRNA35	NA
ciRNA36	NA
ciRNA37	04146(Peroxisome)
ciRNA38	NA
ciRNA39	04020(Calcium signaling pathway);05010(Alzheimer's disease)
ciRNA4	NA
ciRNA40	04020(Calcium signaling pathway);05010(Alzheimer's disease)
ciRNA41	04020(Calcium signaling pathway);05010(Alzheimer's disease)
ciRNA42	04722(Neurotrophin signaling pathway)
ciRNA43	NA
ciRNA44	NA
ciRNA45	05200(Pathways in cancer);05212(Pancreatic cancer)
ciRNA46	NA
ciRNA47	NA
ciRNA48	04510(Focal adhesion);04512(ECM-receptor interaction)
ciRNA49	NA
ciRNA5	NA
ciRNA50	NA
ciRNA51	NA
ciRNA52	04012(ErbB signaling pathway);04150(mTOR signaling pathway);04350(TGF-beta signaling pathway);04666(Fc gamma R-mediated phagocytosis);04910(Insulin signaling pathway);05221(Acute myeloid leu
ciRNA53	04145(Phagosome);04350(TGF-beta signaling pathway);04510(Focal adhesion);04512(ECM-receptor interaction);05144(Malaria)
ciRNA54	04530(Tight junction);05416(Viral myocarditis)
ciRNA55	NA
ciRNA56	NA
ciRNA57	04360(Axon guidance)
ciRNA58	00062(Fatty acid elongation in mitochondria);00071(Fatty acid metabolism);00280(Valine, leucine and isoleucine degradation);00310(Lysine degradation);00380(Tryptophan metabolism);00650(Butanoate m
ciRNA59	NA
ciRNA6	NA
ciRNA60	NA
ciRNA61	NA
ciRNA62	04142(Lysosome);04142(Lysosome)
ciRNA63	NA
ciRNA64	NA
ciRNA65	NA
ciRNA66	NA
ciRNA67	NA
ciRNA68	NA
ciRNA69	NA
ciRNA7	NA
ciRNA70	00510(N-Glycan biosynthesis)
ciRNA71	NA
ciRNA72	NA
ciRNA73	00230(Purine metabolism);00240(Pyrimidine metabolism);03018(RNA degradation)
ciRNA74	NA
ciRNA75	NA
ciRNA76	NA
ciRNA77	00230(Purine metabolism);00240(Pyrimidine metabolism);03020(RNA polymerase);04623(Cytosolic DNA-sensing pathway)
ciRNA78	NA
ciRNA79	NA
ciRNA8	NA

ciRNA80	NA
ciRNA81	05410(Hypertrophic cardiomyopathy (HCM));05414(Dilated cardiomyopathy)
ciRNA82	04670(Leukocyte transendothelial migration)
ciRNA83	00512(O-Glycan biosynthesis)
ciRNA84	NA
ciRNA85	NA
ciRNA86	NA
ciRNA87	NA
ciRNA88	NA
ciRNA89	NA
ciRNA9	NA
ciRNA90	NA
ciRNA91	00380(Tryptophan metabolism);00450(Selenoamino acid metabolism)
ciRNA92	NA
ciRNA93	04360(Axon guidance);04360(Axon guidance)
ciRNA94	NA
ciRNA95	NA
ciRNA96	NA
ciRNA97	NA
ciRNA98	NA
circRNA1	NA
circRNA10	NA
circRNA100	NA
circRNA101	NA
circRNA102	NA
circRNA103	NA
circRNA104	NA
circRNA105	NA
circRNA106	04520(Adherens junction);04666(Fc gamma R-mediated phagocytosis)
circRNA107	NA
circRNA108	05410(Hypertrophic cardiomyopathy (HCM));05412(Arrhythmogenic right ventricular cardiomyopathy (ARVC));05414(Dilated cardiomyopathy);05416(Viral myocarditis)
circRNA109	NA
circRNA11	NA
circRNA110	NA
circRNA111	NA
circRNA112	04810(Regulation of actin cytoskeleton)
circRNA113	04810(Regulation of actin cytoskeleton)
circRNA114	NA
circRNA115	05100(Bacterial invasion of epithelial cells)
circRNA116	05100(Bacterial invasion of epithelial cells)
circRNA117	05100(Bacterial invasion of epithelial cells)
circRNA118	NA
circRNA119	NA
circRNA12	NA
circRNA120	NA
circRNA121	NA
circRNA122	NA
circRNA123	NA
circRNA124	NA
circRNA125	04010(MAPK signaling pathway)
circRNA126	04010(MAPK signaling pathway)
circRNA127	NA
circRNA128	04145(Phagosome);04145(Phagosome)
circRNA129	NA
circRNA13	NA
circRNA130	NA
circRNA131	NA
circRNA132	NA
circRNA133	NA
circRNA134	NA
circRNA135	NA
circRNA136	00061(Fatty acid biosynthesis);00620(Pyruvate metabolism);00640(Propanoate metabolism);04910(Insulin signaling pathway)
circRNA137	00061(Fatty acid biosynthesis);00620(Pyruvate metabolism);00640(Propanoate metabolism);04910(Insulin signaling pathway)
circRNA138	00061(Fatty acid biosynthesis);00620(Pyruvate metabolism);00640(Propanoate metabolism);04910(Insulin signaling pathway)
circRNA139	00061(Fatty acid biosynthesis);00620(Pyruvate metabolism);00640(Propanoate metabolism);04910(Insulin signaling pathway)
circRNA14	NA
circRNA140	NA
circRNA141	NA
circRNA142	00240(Pyrimidine metabolism);00450(Selenoamino acid metabolism)
circRNA143	NA
circRNA144	NA
circRNA145	NA
circRNA146	NA
circRNA147	NA
circRNA148	NA
circRNA149	NA
circRNA15	NA
circRNA150	00072(Synthesis and degradation of ketone bodies);00280(Valine, leucine and isoleucine degradation);00650(Butanoate metabolism)
circRNA151	04150(mTOR signaling pathway)
circRNA152	NA
circRNA153	NA
circRNA154	NA
circRNA155	NA
circRNA156	NA
circRNA157	NA
circRNA158	NA
circRNA159	04912(GnRH signaling pathway);04914(Progesterone-mediated oocyte maturation);04916(Melanogenesis);05414(Dilated cardiomyopathy)
circRNA159	00230(Purine metabolism);04020(Calcium signaling pathway);04062(Chemokine signaling pathway);04114(Oocyte meiosis);04270(Vascular smooth muscle contraction);04540(Gap junction);
circRNA16	03060(Protein export);04145(Phagosome)
circRNA160	04912(GnRH signaling pathway);04914(Progesterone-mediated oocyte maturation);04916(Melanogenesis);05414(Dilated cardiomyopathy)
circRNA160	00230(Purine metabolism);04020(Calcium signaling pathway);04062(Chemokine signaling pathway);04114(Oocyte meiosis);04270(Vascular smooth muscle contraction);04540(Gap junction);
circRNA161	00230(Purine metabolism);04020(Calcium signaling pathway);04062(Chemokine signaling pathway);04114(Oocyte meiosis);04270(Vascular smooth muscle contraction);04540(Gap junction);
circRNA161	04912(GnRH signaling pathway);04914(Progesterone-mediated oocyte maturation);04916(Melanogenesis);05414(Dilated cardiomyopathy)
circRNA162	NA
circRNA163	00620(Pyruvate metabolism);00630(Glyoxylate and dicarboxylate metabolism);00640(Propanoate metabolism);00650(Butanoate metabolism);00900(Terpenoid backbone biosynthesis)
circRNA163	00071(Fatty acid metabolism);00072(Synthesis and degradation of ketone bodies);00280(Valine, leucine and isoleucine degradation);00310(Lysine degradation);00380(Tryptophan metabolism);
circRNA164	NA
circRNA165	NA

circRNA166	NA
circRNA167	NA
circRNA168	NA
circRNA169	NA
circRNA17	NA
circRNA170	NA
circRNA171	NA
circRNA172	NA
circRNA173	NA
circRNA174	NA
circRNA175	NA
circRNA176	NA
circRNA177	NA
circRNA178	NA
circRNA179	NA
circRNA18	NA
circRNA180	NA
circRNA181	NA
circRNA182	04020(Calcium signaling pathway);04910(Insulin signaling pathway)
circRNA183	NA
circRNA184	NA
circRNA185	NA
circRNA186	NA
circRNA187	NA
circRNA188	04120(Ubiquitin mediated proteolysis)
circRNA189	NA
circRNA19	04062(Chemokine signaling pathway);04080(Neuroactive ligand-receptor interaction);04144(Endocytosis);04520(Adherens junction);04530(Tight junction)
circRNA190	NA
circRNA191	NA
circRNA192	NA
circRNA193	00330(Arginine and proline metabolism)
circRNA194	04120(Ubiquitin mediated proteolysis)
circRNA195	NA
circRNA196	NA
circRNA197	NA
circRNA198	00500(Starch and sucrose metabolism);04910(Insulin signaling pathway)
circRNA199	04530(Tight junction);05416(Viral myocarditis)
circRNA2	NA
circRNA20	04062(Chemokine signaling pathway);04080(Neuroactive ligand-receptor interaction);04144(Endocytosis);04520(Adherens junction);04530(Tight junction)
circRNA200	NA
circRNA201	NA
circRNA202	NA
circRNA203	NA
circRNA204	NA
circRNA205	NA
circRNA206	NA
circRNA207	NA
circRNA208	04720(Long-term potentiation);04730(Long-term depression);04912(GnRH signaling pathway);05010(Alzheimer's disease);05016(Huntington's disease)
circRNA208	04020(Calcium signaling pathway);04070(Phosphatidylinositol signaling system);04114(Oocyte meiosis);04270(Vascular smooth muscle contraction);04540(Gap junction);
circRNA209	04730(Long-term depression);04912(GnRH signaling pathway);05010(Alzheimer's disease);05016(Huntington's disease)
circRNA209	04020(Calcium signaling pathway);04070(Phosphatidylinositol signaling system);04114(Oocyte meiosis);04270(Vascular smooth muscle contraction);04540(Gap junction);04720(Long-term potentiation);
circRNA21	NA
circRNA210	00020(Citrate cycle (TCA cycle));00640(Propanoate metabolism)
circRNA211	NA
circRNA212	NA
circRNA213	NA
circRNA214	NA
circRNA215	NA
circRNA216	NA
circRNA217	00510(N-Glycan biosynthesis)
circRNA218	NA
circRNA219	NA
circRNA22	NA
circRNA220	NA
circRNA221	NA
circRNA222	NA
circRNA223	NA
circRNA224	NA
circRNA225	NA
circRNA226	NA
circRNA227	NA
circRNA228	NA
circRNA229	NA
circRNA23	NA
circRNA230	NA
circRNA231	NA
circRNA232	NA
circRNA233	NA
circRNA234	NA
circRNA235	NA
circRNA236	NA
circRNA237	NA
circRNA238	NA
circRNA239	NA
circRNA24	NA
circRNA240	NA
circRNA241	NA
circRNA242	04142(Lysosome)
circRNA243	00020(Citrate cycle (TCA cycle));00480(Glutathione metabolism);04146(Peroxisome)
circRNA244	00020(Citrate cycle (TCA cycle));00480(Glutathione metabolism);04146(Peroxisome)
circRNA245	00230(Purine metabolism)
circRNA246	NA
circRNA247	NA
circRNA248	NA
circRNA249	NA
circRNA25	NA
circRNA250	NA
circRNA251	NA

circRNA252	NA
circRNA253	NA
circRNA254	NA
circRNA255	00562(Inositol phosphate metabolism);04070(Phosphatidylinositol signaling system)
circRNA256	NA
circRNA257	NA
circRNA258	NA
circRNA259	NA
circRNA26	NA
circRNA260	NA
circRNA261	NA
circRNA262	04060(Cytokine-cytokine receptor interaction);04150(mTOR signaling pathway);04510(Focal adhesion);05200(Pathways in cancer);05211(Renal cell carcinoma);05212(Pancreatic cancer);05219(Bladder canc
circRNA263	00511(Other glycan degradation);04142(Lysosome)
circRNA264	NA
circRNA265	NA
circRNA266	NA
circRNA267	NA
circRNA268	03022(Basal transcription factors)
circRNA269	NA
circRNA27	NA
circRNA270	NA
circRNA271	NA
circRNA272	NA
circRNA273	NA
circRNA274	NA
circRNA275	NA
circRNA276	NA
circRNA277	NA
circRNA278	NA
circRNA279	04310(Wnt signaling pathway);05200(Pathways in cancer);05210(Colorectal cancer);05213(Endometrial cancer);05217(Basal cell carcinoma)
circRNA28	NA
circRNA280	04912(GnRH signaling pathway);04914(Progesterone-mediated oocyte maturation);04916(Melanogenesis);04962(Vasopressin-regulated water reabsorption);05414(Dilated cardiomyopathy)
circRNA280	00230(Purine metabolism);04020(Calcium signaling pathway);04062(Chemokine signaling pathway);04114(Oocyte meiosis);04270(Vascular smooth muscle contraction);04540(Gap junction);
circRNA281	NA
circRNA282	NA
circRNA283	NA
circRNA284	NA
circRNA285	NA
circRNA286	NA
circRNA287	NA
circRNA288	NA
circRNA289	NA
circRNA29	NA
circRNA290	NA
circRNA291	NA
circRNA292	NA
circRNA293	NA
circRNA294	04020(Calcium signaling pathway);05010(Alzheimer's disease)
circRNA295	04020(Calcium signaling pathway);05010(Alzheimer's disease)
circRNA296	04145(Phagosome);04514(Cell adhesion molecules (CAMs));04640(Hematopoietic cell lineage);04670(Leukocyte transendothelial migration);04810(Regulation of actin cytoskeleton);05140(Leishmaniasis)
circRNA297	NA
circRNA298	NA
circRNA299	NA
circRNA3	NA
circRNA30	NA
circRNA300	NA
circRNA301	NA
circRNA302	NA
circRNA303	05322(Systemic lupus erythematosus);05412(Arrhythmogenic right ventricular cardiomyopathy (ARVC)
circRNA303	04510(Focal adhesion);04520(Adherens junction);04530(Tight junction);04670(Leukocyte transendothelial migration);04810(Regulation of actin cytoskeleton);
circRNA304	05322(Systemic lupus erythematosus);05412(Arrhythmogenic right ventricular cardiomyopathy (ARVC)
circRNA304	04510(Focal adhesion);04520(Adherens junction);04530(Tight junction);04670(Leukocyte transendothelial migration);04810(Regulation of actin cytoskeleton);
circRNA305	NA
circRNA306	NA
circRNA307	NA
circRNA308	NA
circRNA309	05213(Endometrial cancer);05412(Arrhythmogenic right ventricular cardiomyopathy (ARVC)
circRNA309	04520(Adherens junction);04530(Tight junction);04670(Leukocyte transendothelial migration);05100(Bacterial invasion of epithelial cells);05200(Pathways in cancer);
circRNA31	NA
circRNA310	04120(Ubiquitin mediated proteolysis)
circRNA311	NA
circRNA312	00524(Butirosin and neomycin biosynthesis);04910(Insulin signaling pathway);04930(Type II diabetes mellitus)
circRNA312	00010(Glycolysis / Gluconeogenesis);00051(Fructose and mannose metabolism);00052(Galactose metabolism);00500(Starch and sucrose metabolism);00520(Amino sugar and nucleotide sugar metabolism);
circRNA313	NA
circRNA314	05142(Chagas disease);05200(Pathways in cancer);
circRNA314	04910(Insulin signaling pathway);04912(GnRH signaling pathway);04914(Progesterone-mediated oocyte maturation);04920(Adipocytokine signaling pathway);04930(Type II diabetes mellitus);
circRNA314	04621(NOD-like receptor signaling pathway);04622(RIG-I-like receptor signaling pathway);04664(Fc epsilon RI signaling pathway);04722(Neurotrophin signaling pathway);05212(Pancreatic cancer)
circRNA314	04010(MAPK signaling pathway);04012(ErbB signaling pathway);04310(Wnt signaling pathway);04510(Focal adhesion);04620(Toll-like receptor signaling pathway);05210(Colorectal cancer);
circRNA315	04120(Ubiquitin mediated proteolysis)
circRNA316	04540(Gap junction);04730(Long-term depression);04740(Olfactory transduction)
circRNA317	04540(Gap junction);04730(Long-term depression);04740(Olfactory transduction)
circRNA318	NA
circRNA319	NA
circRNA32	NA
circRNA320	NA
circRNA321	NA
circRNA322	NA
circRNA323	NA
circRNA324	NA
circRNA325	NA
circRNA326	NA
circRNA327	NA
circRNA328	NA
circRNA329	04130(SNARE interactions in vesicular transport)
circRNA33	NA
circRNA330	NA
circRNA331	NA
circRNA332	NA

circRNA333	NA
circRNA334	NA
circRNA335	NA
circRNA336	04510(Focal adhesion);04810(Regulation of actin cytoskeleton);05100(Bacterial invasion of epithelial cells)
circRNA337	04510(Focal adhesion);04810(Regulation of actin cytoskeleton);05100(Bacterial invasion of epithelial cells)
circRNA338	NA
circRNA339	NA
circRNA34	NA
circRNA340	NA
circRNA341	NA
circRNA342	NA
circRNA343	NA
circRNA344	NA
circRNA345	NA
circRNA346	NA
circRNA347	04320(Dorso-ventral axis formation)
circRNA348	NA
circRNA349	NA
circRNA35	NA
circRNA350	NA
circRNA351	NA
circRNA352	NA
circRNA353	04340(Hedgehog signaling pathway)
circRNA354	04340(Hedgehog signaling pathway)
circRNA355	NA
circRNA356	NA
circRNA357	NA
circRNA358	NA
circRNA359	NA
circRNA36	NA
circRNA360	NA
circRNA361	NA
circRNA362	NA
circRNA363	00230(Purine metabolism)
circRNA364	00230(Purine metabolism)
circRNA365	NA
circRNA366	00970(Aminoacyl-tRNA biosynthesis)
circRNA367	00970(Aminoacyl-tRNA biosynthesis)
circRNA368	NA
circRNA369	NA
circRNA37	NA
circRNA370	NA
circRNA371	NA
circRNA372	00592(alpha-Linolenic acid metabolism);01040(Biosynthesis of unsaturated fatty acids);03320(PPAR signaling pathway)
circRNA373	NA
circRNA374	NA
circRNA375	NA
circRNA376	NA
circRNA377	NA
circRNA378	NA
circRNA379	NA
circRNA38	NA
circRNA380	NA
circRNA381	NA
circRNA382	NA
circRNA383	NA
circRNA384	NA
circRNA385	NA
circRNA386	00052(Galactose metabolism);00500(Starch and sucrose metabolism)
circRNA387	NA
circRNA388	NA
circRNA389	NA
circRNA39	NA
circRNA390	NA
circRNA391	NA
circRNA392	NA
circRNA393	NA
circRNA394	NA
circRNA395	NA
circRNA396	NA
circRNA397	NA
circRNA398	00052(Galactose metabolism);00520(Amino sugar and nucleotide sugar metabolism)
circRNA399	NA
circRNA4	04622(RIG-I-like receptor signaling pathway)
circRNA40	NA
circRNA400	NA
circRNA401	00670(One carbon pool by folate)
circRNA402	00514(O-Mannosyl glycan biosynthesis)
circRNA403	00600(Sphingolipid metabolism)
circRNA404	NA
circRNA405	NA
circRNA406	NA
circRNA407	NA
circRNA408	NA
circRNA409	NA
circRNA41	NA
circRNA410	00062(Fatty acid elongation in mitochondria);00071(Fatty acid metabolism);00280(Valine, leucine and isoleucine degradation);00310(Lysine degradation);00380(Tryptophan metabolism);00650(Butanoate m
circRNA411	00062(Fatty acid elongation in mitochondria);00071(Fatty acid metabolism);00280(Valine, leucine and isoleucine degradation);00310(Lysine degradation);00380(Tryptophan metabolism);00650(Butanoate m
circRNA412	00062(Fatty acid elongation in mitochondria);00071(Fatty acid metabolism);00280(Valine, leucine and isoleucine degradation);00310(Lysine degradation);00380(Tryptophan metabolism);00650(Butanoate m
circRNA413	NA
circRNA414	NA
circRNA415	NA
circRNA416	NA
circRNA417	NA
circRNA418	NA
circRNA419	NA
circRNA42	NA
circRNA420	05410(Hypertrophic cardiomyopathy (HCM));05412(Arrhythmogenic right ventricular cardiomyopathy (ARVC));05414(Dilated cardiomyopathy);05416(Viral myocarditis)

circRNA421	00670(One carbon pool by folate)
circRNA422	NA
circRNA423	NA
circRNA424	NA
circRNA425	NA
circRNA426	NA
circRNA427	NA
circRNA428	NA
circRNA429	NA
circRNA43	NA
circRNA430	NA
circRNA431	NA
circRNA432	NA
circRNA433	NA
circRNA434	NA
circRNA435	NA
circRNA436	NA
circRNA437	NA
circRNA438	NA
circRNA439	00230(Purine metabolism)
circRNA44	04115(p53 signaling pathway)
circRNA440	NA
circRNA441	NA
circRNA442	NA
circRNA443	NA
circRNA444	NA
circRNA445	NA
circRNA446	NA
circRNA447	NA
circRNA448	NA
circRNA449	NA
circRNA45	05410(Hypertrophic cardiomyopathy (HCM));05414(Dilated cardiomyopathy)
circRNA45	05142(Chagas disease);05144(Malaria);05200(Pathways in cancer);05210(Colorectal cancer);05211(Renal cell carcinoma);05212(Pancreatic cancer);05220(Chronic myeloid leukemia);
circRNA45	04010(MAPK signaling pathway);04060(Cytokine-cytokine receptor interaction);04110(Cell cycle);04144(Endocytosis);04350(TGF-beta signaling pathway);05140(Leishmaniasis);
circRNA450	NA
circRNA451	00120(Primary bile acid biosynthesis);04146(Peroxisome)
circRNA452	NA
circRNA453	04350(TGF-beta signaling pathway)
circRNA454	NA
circRNA455	NA
circRNA456	04110(Cell cycle);04114(Oocyte meiosis);04120(Ubiquitin mediated proteolysis);04914(Progesterone-mediated oocyte maturation)
circRNA457	NA
circRNA458	NA
circRNA459	NA
circRNA46	NA
circRNA460	04360(Axon guidance)
circRNA461	NA
circRNA462	00534(Glycosaminoglycan biosynthesis - heparan sulfate)
circRNA463	NA
circRNA464	NA
circRNA465	05410(Hypertrophic cardiomyopathy (HCM));05412(Arrhythmogenic right ventricular cardiomyopathy (ARVC));05414(Dilated cardiomyopathy);05416(Viral myocarditis)
circRNA466	NA
circRNA467	04144(Endocytosis);04350(TGF-beta signaling pathway)
circRNA468	NA
circRNA469	04614(Renin-angiotensin system)
circRNA47	NA
circRNA470	NA
circRNA471	00510(N-Glycan biosynthesis)
circRNA472	NA
circRNA473	NA
circRNA474	NA
circRNA475	NA
circRNA476	05144(Malaria);05200(Pathways in cancer);05211(Renal cell carcinoma);05218(Melanoma)
circRNA476	04060(Cytokine-cytokine receptor interaction);04144(Endocytosis);04360(Axon guidance);04510(Focal adhesion);04520(Adherens junction);05100(Bacterial invasion of epithelial cells);
circRNA477	NA
circRNA478	00280(Valine, leucine and isoleucine degradation)
circRNA479	00280(Valine, leucine and isoleucine degradation)
circRNA48	NA
circRNA480	NA
circRNA481	00860(Porphyrin and chlorophyll metabolism)
circRNA482	NA
circRNA483	NA
circRNA484	NA
circRNA485	NA
circRNA486	NA
circRNA487	00512(O-Glycan biosynthesis)
circRNA488	NA
circRNA489	NA
circRNA49	NA
circRNA490	NA
circRNA491	NA
circRNA492	NA
circRNA493	NA
circRNA494	04120(Ubiquitin mediated proteolysis)
circRNA495	NA
circRNA496	00310(Lysine degradation)
circRNA497	00310(Lysine degradation)
circRNA498	NA
circRNA499	NA
circRNA5	04120(Ubiquitin mediated proteolysis)
circRNA50	NA
circRNA500	NA
circRNA501	NA
circRNA502	NA
circRNA503	NA
circRNA504	NA
circRNA505	NA
circRNA506	NA

circRNA507	NA
circRNA508	NA
circRNA509	NA
circRNA51	NA
circRNA510	00410(beta-Alanine metabolism);00640(Propanoate metabolism);00650(Butanoate metabolism);01040(Biosynthesis of unsaturated fatty acids)
circRNA510	00062(Fatty acid elongation in mitochondria);00071(Fatty acid metabolism);00280(Valine, leucine and isoleucine degradation);00310(Lysine degradation);00380(Tryptophan metabolism);
circRNA511	NA
circRNA512	NA
circRNA513	NA
circRNA514	00250(Alanine, aspartate and glutamate metabolism);00520(Amino sugar and nucleotide sugar metabolism)
circRNA515	05010(Alzheimer's disease);05014(Amyotrophic lateral sclerosis (ALS)
circRNA515	04650(Natural killer cell mediated cytotoxicity);04660(T cell receptor signaling pathway);04662(B cell receptor signaling pathway);04720(Long-term potentiation);
circRNA515	04010(MAPK signaling pathway);04020(Calcium signaling pathway);04114(Oocyte meiosis);04210(Apoptosis);04310(Wnt signaling pathway);04360(Axon guidance);04370(VEGF signaling pathway);
circRNA516	00040(Pentose and glucuronate interconversions);00052(Galactose metabolism);00500(Starch and sucrose metabolism);00520(Amino sugar and nucleotide sugar metabolism)
circRNA517	NA
circRNA518	NA
circRNA519	NA
circRNA52	03040(Spliceosome)
circRNA520	NA
circRNA521	04144(Endocytosis)
circRNA522	NA
circRNA523	NA
circRNA524	NA
circRNA525	NA
circRNA526	NA
circRNA527	NA
circRNA528	04270(Vascular smooth muscle contraction);04530(Tight junction)
circRNA529	NA
circRNA53	05214(Glioma);05215(Prostate cancer);05218(Melanoma);05220(Chronic myeloid leukemia);05221(Acute myeloid leukemia);05222(Small cell lung cancer);
circRNA53	05223(Non-small cell lung cancer)
circRNA53	05142(Chagas disease);05200(Pathways in cancer);05210(Colorectal cancer);05211(Renal cell carcinoma);05212(Pancreatic cancer);05213(Endometrial cancer);
circRNA53	04722(Neurotrophin signaling pathway);04910(Insulin signaling pathway);04914(Progesterone-mediated oocyte maturation);04920(Adipocytokine signaling pathway);
circRNA53	04370(VEGF signaling pathway);04510(Focal adhesion);04530(Tight junction);04620(Toll-like receptor signaling pathway);04630(Jak-STAT signaling pathway);
circRNA53	04010(MAPK signaling pathway);04012(ErbB signaling pathway);04062(Chemokine signaling pathway);04150(mTOR signaling pathway);04210(Apoptosis);
circRNA53	04660(T cell receptor signaling pathway);04662(B cell receptor signaling pathway);04664(Fc epsilon RI signaling pathway);04666(Fc gamma R-mediated phagocytosis);
circRNA530	NA
circRNA531	NA
circRNA532	NA
circRNA533	NA
circRNA534	NA
circRNA535	NA
circRNA536	NA
circRNA537	NA
circRNA538	NA
circRNA539	NA
circRNA54	NA
circRNA540	NA
circRNA541	NA
circRNA542	04710(Circadian rhythm - mammal)
circRNA543	NA
circRNA544	NA
circRNA545	NA
circRNA546	NA
circRNA547	NA
circRNA548	NA
circRNA549	NA
circRNA55	NA
circRNA550	NA
circRNA551	NA
circRNA552	00564(Glycerophospholipid metabolism)
circRNA553	NA
circRNA554	NA
circRNA555	NA
circRNA556	NA
circRNA557	NA
circRNA558	00514(O-Mannosyl glycan biosynthesis)
circRNA559	NA
circRNA56	NA
circRNA560	NA
circRNA561	04722(Neurotrophin signaling pathway);05211(Renal cell carcinoma)
circRNA561	04010(MAPK signaling pathway);04062(Chemokine signaling pathway);04510(Focal adhesion);04670(Leukocyte transendothelial migration);04720(Long-term potentiation);
circRNA562	NA
circRNA563	04360(Axon guidance)
circRNA564	NA
circRNA565	04115(p53 signaling pathway);04210(Apoptosis);05010(Alzheimer's disease);05012(Parkinson's disease);05014(Amyotrophic lateral sclerosis (ALS));05016(Huntington's disease);05222(Small cell lung cance
circRNA566	NA
circRNA567	NA
circRNA568	NA
circRNA569	NA
circRNA57	NA
circRNA570	NA
circRNA571	00240(Pyrimidine metabolism);00240(Pyrimidine metabolism);00450(Selenoamino acid metabolism);00450(Selenoamino acid metabolism)
circRNA572	NA
circRNA573	NA
circRNA574	02010(ABC transporters)
circRNA575	02010(ABC transporters)
circRNA576	NA
circRNA577	NA
circRNA578	NA
circRNA579	04530(Tight junction)
circRNA58	NA
circRNA580	04610(Complement and coagulation cascades);05322(Systemic lupus erythematosus)
circRNA581	NA
circRNA582	04510(Focal adhesion);04512(ECM-receptor interaction);04610(Complement and coagulation cascades)
circRNA583	00190(Oxidative phosphorylation);05010(Alzheimer's disease);05012(Parkinson's disease);05016(Huntington's disease)
circRNA584	NA
circRNA585	NA
circRNA586	NA

circRNA587	00230(Purine metabolism);00250(Alanine, aspartate and glutamate metabolism)
circRNA588	NA
circRNA589	NA
circRNA59	NA
circRNA590	NA
circRNA591	NA
circRNA592	02010(ABC transporters)
circRNA593	NA
circRNA594	NA
circRNA595	NA
circRNA596	04370(VEGF signaling pathway);04650(Natural killer cell mediated cytotoxicity);04660(T cell receptor signaling pathway);04662(B cell receptor signaling pathway);
circRNA596	04720(Long-term potentiation);05010(Alzheimer's disease);05014(Amyotrophic lateral sclerosis (ALS)
circRNA596	04010(MAPK signaling pathway);04020(Calcium signaling pathway);04114(Oocyte meiosis);04210(Apoptosis);04310(Wnt signaling pathway);04360(Axon guidance);
circRNA597	NA
circRNA598	NA
circRNA599	NA
circRNA6	NA
circRNA60	04115(p53 signaling pathway);04120(Ubiquitin mediated proteolysis)
circRNA600	NA
circRNA601	NA
circRNA602	NA
circRNA603	04530(Tight junction)
circRNA604	NA
circRNA605	NA
circRNA606	00534(Glycosaminoglycan biosynthesis - heparan sulfate)
circRNA607	NA
circRNA608	NA
circRNA609	NA
circRNA61	04510(Focal adhesion);04512(ECM-receptor interaction);05020(Prion diseases);05200(Pathways in cancer);05222(Small cell lung cancer)
circRNA610	NA
circRNA611	NA
circRNA612	NA
circRNA613	NA
circRNA614	NA
circRNA615	05410(Hypertrophic cardiomyopathy (HCM));05414(Dilated cardiomyopathy)
circRNA616	05410(Hypertrophic cardiomyopathy (HCM));05414(Dilated cardiomyopathy)
circRNA617	05410(Hypertrophic cardiomyopathy (HCM));05414(Dilated cardiomyopathy)
circRNA618	05410(Hypertrophic cardiomyopathy (HCM));05414(Dilated cardiomyopathy)
circRNA619	05410(Hypertrophic cardiomyopathy (HCM));05414(Dilated cardiomyopathy)
circRNA62	00310(Lysine degradation);00514(O-Mannosyl glycan biosynthesis)
circRNA620	05410(Hypertrophic cardiomyopathy (HCM));05414(Dilated cardiomyopathy)
circRNA621	05410(Hypertrophic cardiomyopathy (HCM));05414(Dilated cardiomyopathy)
circRNA622	05410(Hypertrophic cardiomyopathy (HCM));05414(Dilated cardiomyopathy)
circRNA623	05410(Hypertrophic cardiomyopathy (HCM));05414(Dilated cardiomyopathy)
circRNA624	05410(Hypertrophic cardiomyopathy (HCM));05414(Dilated cardiomyopathy)
circRNA625	NA
circRNA626	04010(MAPK signaling pathway);04530(Tight junction)
circRNA627	NA
circRNA628	NA
circRNA629	NA
circRNA63	NA
circRNA630	NA
circRNA631	NA
circRNA632	NA
circRNA633	NA
circRNA634	04060(Cytokine-cytokine receptor interaction);04350(TGF-beta signaling pathway)
circRNA635	NA
circRNA636	05200(Pathways in cancer);05200(Pathways in cancer);05212(Pancreatic cancer);05212(Pancreatic cancer)
circRNA637	NA
circRNA638	NA
circRNA639	NA
circRNA64	NA
circRNA640	NA
circRNA641	NA
circRNA642	00071(Fatty acid metabolism);03320(PPAR signaling pathway)
circRNA643	NA
circRNA644	00562(Inositol phosphate metabolism);04020(Calcium signaling pathway);04070(Phosphatidylinositol signaling system)
circRNA645	04120(Ubiquitin mediated proteolysis)
circRNA646	04914(Progesterone-mediated oocyte maturation)
circRNA646	04010(MAPK signaling pathway);04114(Oocyte meiosis);04150(mTOR signaling pathway);04720(Long-term potentiation);04722(Neurotrophin signaling pathway);
circRNA647	NA
circRNA648	NA
circRNA649	NA
circRNA65	NA
circRNA650	NA
circRNA651	NA
circRNA652	NA
circRNA653	NA
circRNA654	NA
circRNA655	NA
circRNA656	NA
circRNA657	NA
circRNA658	NA
circRNA659	NA
circRNA66	NA
circRNA660	NA
circRNA661	NA
circRNA662	NA
circRNA663	NA
circRNA664	NA
circRNA665	NA
circRNA666	NA
circRNA667	NA
circRNA668	NA
circRNA669	NA
circRNA67	NA
circRNA670	NA
circRNA671	NA

circRNA672	04140(Regulation of autophagy)
circRNA673	00052(Galactose metabolism);00230(Purine metabolism);00230(Purine metabolism);00500(Starch and sucrose metabolism);00500(Starch and sucrose metabolism);
circRNA673	00010(Glycolysis / Gluconeogenesis);00010(Glycolysis / Gluconeogenesis);00030(Pentose phosphate pathway);00030(Pentose phosphate pathway);00052(Galactose metabolism);
circRNA673	00520(Amino sugar and nucleotide sugar metabolism);00520(Amino sugar and nucleotide sugar metabolism)
circRNA674	NA
circRNA675	NA
circRNA676	NA
circRNA677	NA
circRNA678	NA
circRNA679	NA
circRNA68	00061(Fatty acid biosynthesis);00620(Pyruvate metabolism);00640(Propanoate metabolism);04910(Insulin signaling pathway)
circRNA680	NA
circRNA681	00230(Purine metabolism)
circRNA682	NA
circRNA683	NA
circRNA684	NA
circRNA685	NA
circRNA686	NA
circRNA687	NA
circRNA688	00562(Inositol phosphate metabolism);04070(Phosphatidylinositol signaling system)
circRNA689	NA
circRNA69	NA
circRNA690	NA
circRNA691	NA
circRNA692	NA
circRNA693	NA
circRNA694	NA
circRNA695	NA
circRNA696	NA
circRNA697	NA
circRNA698	NA
circRNA699	05010(Alzheimer's disease)
circRNA7	04020(Calcium signaling pathway);04910(Insulin signaling pathway)
circRNA70	03050(Proteasome)
circRNA700	NA
circRNA701	NA
circRNA702	NA
circRNA703	NA
circRNA704	NA
circRNA705	NA
circRNA706	NA
circRNA707	NA
circRNA708	NA
circRNA709	NA
circRNA71	NA
circRNA710	NA
circRNA711	NA
circRNA712	NA
circRNA713	NA
circRNA714	NA
circRNA715	NA
circRNA716	NA
circRNA717	NA
circRNA718	04310(Wnt signaling pathway)
circRNA719	NA
circRNA72	NA
circRNA720	NA
circRNA721	00564(Glycerophospholipid metabolism);00565(Ether lipid metabolism);04144(Endocytosis);04666(Fc gamma R-mediated phagocytosis);04912(GnRH signaling pathway)
circRNA722	NA
circRNA723	00230(Purine metabolism);00983(Drug metabolism - other enzymes)
circRNA724	04012(ErbB signaling pathway);04660(T cell receptor signaling pathway)
circRNA725	NA
circRNA726	NA
circRNA727	NA
circRNA728	NA
circRNA729	NA
circRNA73	NA
circRNA730	NA
circRNA731	NA
circRNA732	NA
circRNA733	NA
circRNA734	NA
circRNA735	NA
circRNA736	NA
circRNA737	NA
circRNA738	NA
circRNA739	NA
circRNA74	NA
circRNA740	NA
circRNA741	NA
circRNA742	04670(Leukocyte transendothelial migration);04810(Regulation of actin cytoskeleton);05100(Bacterial invasion of epithelial cells);05222(Small cell lung cancer)
circRNA742	04012(ErbB signaling pathway);04062(Chemokine signaling pathway);04360(Axon guidance);04370(VEGF signaling pathway);04510(Focal adhesion);05200(Pathways in cancer);
circRNA743	NA
circRNA744	03450(Non-homologous end-joining);04110(Cell cycle)
circRNA745	NA
circRNA746	NA
circRNA747	NA
circRNA748	NA
circRNA749	NA
circRNA75	04144(Endocytosis)
circRNA750	NA
circRNA751	NA
circRNA752	NA
circRNA753	NA
circRNA754	NA
circRNA755	NA
circRNA756	NA
circRNA757	NA

circRNA758	NA
circRNA759	04621(NOD-like receptor signaling pathway);04722(Neurotrophin signaling pathway)
circRNA76	NA
circRNA760	NA
circRNA761	NA
circRNA762	NA
circRNA763	NA
circRNA764	NA
circRNA765	NA
circRNA766	NA
circRNA767	NA
circRNA768	NA
circRNA769	NA
circRNA77	04530(Tight junction);05416(Viral myocarditis)
circRNA770	NA
circRNA771	NA
circRNA772	NA
circRNA773	NA
circRNA774	NA
circRNA775	04530(Tight junction)
circRNA776	NA
circRNA777	00511(Other glycan degradation)
circRNA778	NA
circRNA779	NA
circRNA78	NA
circRNA780	NA
circRNA781	NA
circRNA782	NA
circRNA783	NA
circRNA784	NA
circRNA785	NA
circRNA786	04142(Lysosome)
circRNA787	NA
circRNA788	NA
circRNA79	04144(Endocytosis);04145(Phagosome);04962(Vasopressin-regulated water reabsorption)
circRNA8	04610(Complement and coagulation cascades)
circRNA80	00190(Oxidative phosphorylation);04142(Lysosome);04145(Phagosome);04966(Collecting duct acid secretion)
circRNA81	03040(Spliceosome)
circRNA82	NA
circRNA83	NA
circRNA84	NA
circRNA85	NA
circRNA86	NA
circRNA87	04520(Adherens junction);04520(Adherens junction);04810(Regulation of actin cytoskeleton);04810(Regulation of actin cytoskeleton)
circRNA88	NA
circRNA89	03060(Protein export)
circRNA9	NA
circRNA90	NA
circRNA91	04621(NOD-like receptor signaling pathway)
circRNA92	NA
circRNA93	NA
circRNA94	03022(Basal transcription factors)
circRNA95	NA
circRNA96	NA
circRNA97	00020(Citrate cycle (TCA cycle));00640(Propanoate metabolism)
circRNA98	NA
circRNA99	NA

Accession	GO
ciRNA1	GO:0000166(nucleotide binding);GO:0003924(GTPase activity);GO:0005525(GTP binding);GO:0006184(GTP catabolic process)
ciRNA10	GO:0046580(negative regulation of Ras protein signal transduction);GO:0015630(microtubule cytoskeleton);GO:0001825(blastocyst formation);GO:0048593(camera-type eye morphogenesis)
ciRNA10	GO:0008053(mitochondrial fusion);GO:0016021(integral component of membrane);GO:0048662(negative regulation of smooth muscle cell proliferation);GO:0005515(protein binding);
ciRNA10	GO:0031625(ubiquitin protein ligase binding);GO:0006626(protein targeting to mitochondrion);GO:0051646(mitochondrion localization);GO:0007006(mitochondrial membrane organization);
ciRNA10	GO:0003924(GTPase activity);GO:0005525(GTP binding);GO:0005739(mitochondrion);GO:0005741(mitochondrial outer membrane);GO:0006184(GTP catabolic process);
ciRNA11	GO:0005515(protein binding);GO:0005813(centrosome)
ciRNA12	GO:0005488(binding);GO:0070062(extracellular vesicular exosome)
ciRNA12	GO:0004177(aminopeptidase activity);GO:0005615(extracellular space);GO:0006508(proteolysis);GO:0008237(metallopeptidase activity);GO:0008270(zinc ion binding);GO:0019370(leukotriene biosynthetic
ciRNA13	GO:0042634(regulation of hair cycle);GO:0032922(circadian regulation of gene expression);GO:0007623(circadian rhythm);GO:0043967(histone H4 acetylation);
ciRNA13	GO:0045944(positive regulation of transcription from RNA polymerase II promoter);GO:0000122(negative regulation of transcription from RNA polymerase II promoter);GO:0000976(transcription regulatory region sequence-specific DNA binding);GO:0008134(transcription factor binding);GO:0031490(chromatin DNA binding);GO:0009416(response to light stimulus);GO:0010608(posttranscriptional regulation of gene expression);GO:0070932(histone H3 deacetylation);GO:0000989(transcription factor binding transcription factor activity);GO:0002028(regulation of sodium ion transport);GO:0042752(regulation of circadian rhythm);GO:0043153(entrainment of circadian clock by photoperiod);GO:2000323(negative regulation of glucocorticoid receptor signaling pathway);GO:0097167(circadian regulation of translation)
ciRNA13	GO:0043966(histone H3 acetylation);GO:0005634(nucleus);GO:0045892("negative regulation of transcription, DNA-templated");
ciRNA13	GO:0000978(RNA polymerase II core promoter proximal region sequence-specific DNA binding);GO:0031625(ubiquitin protein ligase binding);GO:0070888(E-box binding);
ciRNA13	GO:0004871(signal transducer activity);GO:0007165(signal transduction);GO:0005515(protein binding);GO:0019900(kinase binding);GO:0005737(cytoplasm);
ciRNA14	0978(RNA polymerase II core promoter proximal region sequence-specific DNA binding);GO:0031625(ubiquitin protein ligase binding);GO:0070888(E-box binding);GO:0042634(regulation of hair cycle);GO:0032922(circadian regulation of gene expression);GO:0007623(circadian rhythm);GO:0043967(histone H4 acetylation);GO:0043966(histone H3 acetylation);GO:0005634(nucleus);GO:0045892("negative regulation of transcription, DNA-templated");GO:0045944(positive regulation of transcription from RNA polymerase II promoter);GO:0000122(negative regulation of transcription from RNA polymerase II promoter);GO:0000976(transcription regulatory region sequence-specific DNA binding);GO:0008134(transcription factor binding);GO:0031490(chromatin DNA binding);GO:0009416(response to light stimulus);GO:0010608(posttranscriptional regulation of gene expression);GO:0070932(histone H3 deacetylation);GO:0000989(transcription factor binding transcription factor activity);GO:0002028(regulation of sodium ion transport);GO:0042752(regulation of circadian rhythm);GO:0043153(entrainment of circadian clock by photoperiod);GO:2000323(negative regulation of glucocorticoid receptor signaling pathway);GO:0097167(circadian regulation of translation)
ciRNA15	GO:0000166(nucleotide binding);GO:0000930(gamma-tubulin complex);GO:0003924(GTPase activity);GO:0005525(GTP binding);GO:0005874(microtubule);GO:0006184(GTP catabolic process);GO:0007017(microtubule-based process);GO:0007020(microtubule nucleation);GO:0031122(cytoplasmic microtubule organization);GO:0043234(protein complex);GO:0051258(protein

ciRNA16	GO:0000166(nucleotide binding);GO:0000930(gamma-tubulin complex);GO:0003924(GTPase activity);GO:0005525(GTP binding);GO:0005874(microtubule);GO:0006184(GTP catabolic process);GO:0007017(microtubule-based process);GO:0007020(microtubule nucleation);GO:0031122(cytoplasmic microtubule organization);GO:0043234(protein complex);GO:0051258(protein polymerization);GO:0005813(centrosome);GO:0005876(spindle microtubule);GO:0005881(cytoplasmic microtubule);GO:0000242(pericentriolar material)
ciRNA17	GO:0016021(integral component of membrane)
ciRNA18	GO:0000166(nucleotide binding);GO:0005525(GTP binding);GO:0007264(small GTPase mediated signal transduction);GO:0015031(protein transport);GO:0003924(GTPase activity);GO:0006184(GTP catabolic process);GO:0006886(intracellular protein transport);GO:0006913(nucleocytoplasmic transport);GO:0007165(signal transduction);GO:0005622(intracellular);GO:0005634(nucleus);GO:0005737(cytoplasm);GO:0016020(membrane);GO:0005793(endoplasmic reticulum-Golgi intermediate compartment);GO:0005515(protein binding);GO:0030141(secretory granule)
ciRNA19	GO:0000166(nucleotide binding);GO:0004672(protein kinase activity);GO:0004674(protein serine/threonine kinase activity);GO:0005524(ATP binding);GO:0006468(protein phosphorylation);GO:0007067(mitotic nuclear division);GO:0016301(kinase activity);GO:0016310(phosphorylation);GO:0016740(transferase activity);GO:0016772("transferase activity, transferring phosphorus-containing groups");GO:0035329(hippo signaling);GO:0005515(protein binding);GO:0004713(protein tyrosine kinase activity);GO:0005634(nucleus);GO:0035556(intracellular signal transduction);GO:0000922(spindle pole);GO:0045736(negative regulation of cyclin-dependent protein serine/threonine kinase activity);GO:0009755(hormone-mediated signaling pathway);GO:0000082(G1/S transition of mitotic cell cycle);GO:0090090(negative regulation of canonical Wnt signaling pathway);GO:0030216(keratinocyte differentiation);GO:0034613(cellular protein localization);GO:0046620(regulation of organ growth)
ciRNA2	GO:0005515(protein binding);GO:0030225(macrophage differentiation)
ciRNA20	()
ciRNA21	GO:0044822(poly(A) RNA binding);GO:0004843(ubiquitin-specific protease activity);GO:0071108(protein K48-linked deubiquitination)
ciRNA22	GO:0005515(protein binding);GO:0045893("positive regulation of transcription, DNA-templated");GO:0016020(membrane);GO:0005737(cytoplasm);GO:0005667(transcription factor complex);GO:0044822(poly(A) RNA binding);GO:0005634(nucleus);GO:0043066(negative regulation of apoptotic process);GO:0050821(protein stabilization);GO:0016407(acetyltransferase activity);GO:0043022(ribosome binding);GO:0031415(NatA complex);GO:0006474(N-terminal protein amino acid acetylation);GO:0008080(N-acetyltransferase activity)
ciRNA23	GO:0016301(kinase activity);GO:0016310(phosphorylation);GO:0016740(transferase activity);GO:0016772("transferase activity, transferring phosphorus-containing groups");GO:0016773("phosphotransferase activity, alcohol group as acceptor");GO:0046854(phosphatidylinositol phosphorylation);GO:0048015(phosphatidylinositol-mediated
ciRNA24	()
ciRNA25	NA
ciRNA26	GO:0005634(nucleus);GO:0006352("DNA-templated transcription, initiation");GO:0051260(protein homooligomerization);GO:0048471(perinuclear region of cytoplasm);GO:0005737(cytoplasm);GO:0005515(protein binding);GO:0003700(sequence-specific DNA binding transcription factor activity);GO:0030331(estrogen receptor binding);GO:0003713(transcription coactivator activity);GO:0033276(transcription factor TFTC complex);GO:0019899(enzyme binding);GO:0005669(transcription factor TFIID complex);GO:0016578(histone deubiquitination);GO:0004402(histone acetyltransferase activity);GO:0070063(RNA polymerase binding);GO:0030914(STAGA complex);GO:0000125(PCAF complex);GO:0043966(histone H3 acetylation)
ciRNA27	GO:0000166(nucleotide binding);GO:0004222(metalloendopeptidase activity);GO:0005524(ATP binding);GO:0006508(proteolysis);GO:0008270(zinc ion binding);GO:0016020(membrane);GO:0016021(integral component of membrane);GO:0017111(nucleoside-triphosphatase activity);GO:0005515(protein binding);GO:0005739(mitochondrion);GO:0007005(mitochondrion organization);GO:0008089(anterograde axon cargo transport)
ciRNA28	GO:0015031(protein transport);GO:0007034(vacuolar transport);GO:0070062(extracellular vesicular exosome);GO:0005515(protein binding);GO:0016192(vesicle-mediated transport);GO:0005815(microtubule organizing center);GO:0012505(endomembrane system);GO:0045892("negative regulation of transcription, DNA-templated");GO:0000910(cytokinesis);GO:0042803(protein homodimerization activity);GO:0019904(protein domain specific binding);GO:0005769(early endosome);GO:0016363(nuclear matrix);GO:0007076(mitotic chromosome condensation);GO:0045014(negative regulation of transcription by glucose);GO:0016458(gene silencing);GO:0000794(condensed nuclear chromosome)
ciRNA29	GO:0003677(DNA binding);GO:0005664(nuclear origin of replication recognition complex);GO:0006260(DNA replication);GO:0016020(membrane);GO:0005515(protein binding);GO:0000808(origin recognition complex);GO:0006261(DNA-dependent DNA replication)
ciRNA3	GO:0005515(protein binding);GO:0005829(cytosol);GO:0071816(tail-anchored membrane protein insertion into ER membrane);GO:0071818(BAT3 complex)
ciRNA30	GO:0000042(protein targeting to Golgi);GO:0005515(protein binding);GO:0043231(intracellular membrane-bounded organelle);GO:0005737(cytoplasm);GO:0005634(nucleus);GO:0005794(Golgi apparatus);GO:0043001(Golgi to plasma membrane protein transport);GO:0051020(GTPase binding)
ciRNA31	GO:0003723(RNA binding)
ciRNA32	GO:0003723(RNA binding)
ciRNA33	GO:0004872(receptor activity);GO:0005622(intracellular);GO:0007165(signal transduction);GO:0007275(multicellular organismal development);GO:0016020(membrane);GO:0005515(protein binding);GO:0019904(protein domain specific binding);GO:0030027(lamellipodium);GO:0005887(integral component of plasma membrane);GO:0030334(regulation of cell
ciRNA34	GO:0005198(structural molecule activity);GO:0006810(transport);GO:0006886(intracellular protein transport);GO:0015031(protein transport);GO:0016020(membrane);GO:0016192(vesicle-mediated transport);GO:0030117(membrane coat);GO:0030126(COPI vesicle coat);GO:0005488(binding);GO:0005515(protein binding)
ciRNA35	GO:0005783(endoplasmic reticulum);GO:0005737(cytoplasm);GO:0005515(protein binding);GO:0005886(plasma membrane);GO:0051281(positive regulation of release of sequestered calcium ion into cytosol);GO:0030665(clathrin-coated vesicle membrane);GO:0090314(positive regulation of protein targeting to membrane);GO:0046923(ER retention sequence binding);GO:0005540(hyaluronic acid
ciRNA36	GO:0007165(signal transduction);GO:0005515(protein binding);GO:0005886(plasma membrane);GO:0030507(spectrin binding);GO:0051117(ATPase binding);GO:0006888(ER to Golgi vesicle-mediated transport);GO:0072661(protein targeting to plasma membrane);GO:0014731(spectrin-associated cytoskeleton);GO:0016020(membrane);GO:0030863(cortical cytoskeleton);GO:0048821(erythrocyte
ciRNA37	GO:0016491(oxidoreductase activity);GO:0008152(metabolic process);GO:0005102(receptor binding);GO:0006636(unsaturated fatty acid biosynthetic process);GO:0019166(trans-2-enoyl-CoA reductase (NADPH) activity);GO:0008670("2,4-dienoyl-CoA reductase (NADPH) activity")
ciRNA38	()
ciRNA39	GO:0000166(nucleotide binding);GO:0005388(calcium-transporting ATPase activity);GO:0005524(ATP binding);GO:0006812(cation transport);GO:0006816(calcium ion transport);GO:0008152(metabolic process);GO:0016020(membrane);GO:0016021(integral component of membrane);GO:0016787(hydrolase activity);GO:0019829(cation-transporting ATPase activity);GO:0046872(metal ion binding);GO:0070588(calcium ion transmembrane transport);GO:0005789(endoplasmic reticulum membrane);GO:0005515(protein binding);GO:0006942(regulation of striated muscle contraction);GO:0042803(protein homodimerization activity);GO:0070509(calcium ion import);GO:0031674(I band);GO:0090076(relaxation of skeletal muscle);GO:0051659(maintenance of mitochondrion location);GO:0051561(positive regulation of mitochondrial calcium ion concentration);GO:0045988(negative regulation of striated muscle contraction);GO:0032471(negative regulation of endoplasmic reticulum calcium ion concentration);GO:0032470(positive regulation of endoplasmic reticulum calcium ion concentration);GO:0031673(H zone);GO:0031448(positive regulation of fast-twitch skeletal muscle fiber contraction);GO:0008637(apoptotic mitochondrial changes);GO:0070059(intrinsic apoptotic signaling pathway in response to endoplasmic reticulum stress);GO:0034976(response to endoplasmic reticulum stress)
ciRNA4	GO:0000166(nucleotide binding);GO:0005524(ATP binding);GO:0006457(protein folding);GO:0042384(cilium assembly);GO:0044267(cellular protein metabolic process);GO:0051082(unfolded protein binding);GO:0060271(cilium morphogenesis);GO:0005813(centrosome);GO:0005622(intracellular);GO:0005515(protein binding);GO:0001103(RNA polymerase II repressing transcription factor binding);GO:0007608(sensory perception of smell);GO:0010629(negative regulation of gene expression);GO:0040018(positive regulation of multicellular organism growth);GO:0048854(brain morphogenesis);GO:0051216(cartilage development);GO:0051131(chaperone-mediated protein complex assembly);GO:0045776(negative regulation of blood pressure);GO:0045444(fat cell differentiation);GO:0035176(social behavior);GO:0021987(cerebral cortex development);GO:0031514(motile cilium);GO:0007286(spermatid development);GO:0021766(hippocampus development);GO:0050910(detection of mechanical stimulus involved in sensory perception of sound);GO:0045494(photoreceptor cell maintenance);GO:0035058(nonmotile primary cilium assembly);GO:0042311(vasodilation);GO:0033210(leptin-mediated signaling pathway);GO:0021756(striatum development);GO:0014824(artery smooth muscle contraction);GO:0044321(response to leptin);GO:0060296(regulation of cilium beat frequency involved in ciliary motility);GO:0038108(negative regulation of appetite by leptin-mediated signaling pathway)
ciRNA40	GO:0000166(nucleotide binding);GO:0005388(calcium-transporting ATPase activity);GO:0005524(ATP binding);GO:0006812(cation transport);GO:0006816(calcium ion transport);GO:0008152(metabolic process);GO:0016020(membrane);GO:0016021(integral component of membrane);GO:0016787(hydrolase activity);GO:0019829(cation-transporting ATPase activity);GO:0046872(metal ion binding);GO:0070588(calcium ion transmembrane transport);GO:0005789(endoplasmic reticulum membrane);GO:0005515(protein binding);GO:0006942(regulation of striated muscle contraction);GO:0042803(protein homodimerization activity);GO:0070509(calcium ion import);GO:0031674(I band);GO:0090076(relaxation of skeletal muscle);GO:0051659(maintenance of mitochondrion location);GO:0051561(positive regulation of mitochondrial calcium ion concentration);GO:0045988(negative regulation of striated muscle contraction);GO:0032471(negative regulation of endoplasmic reticulum calcium ion concentration);GO:0032470(positive regulation of endoplasmic reticulum calcium ion concentration);GO:0031673(H zone);GO:0031448(positive regulation of fast-twitch skeletal muscle fiber contraction);GO:0008637(apoptotic mitochondrial changes);GO:0070059(intrinsic apoptotic signaling pathway in response to endoplasmic reticulum stress);GO:0034976(response to endoplasmic reticulum stress)
ciRNA41	GO:0000166(nucleotide binding);GO:0005388(calcium-transporting ATPase activity);GO:0005524(ATP binding);GO:0006812(cation transport);GO:0006816(calcium ion transport);GO:0008152(metabolic process);GO:0016020(membrane);GO:0016021(integral component of membrane);GO:0016787(hydrolase activity);GO:0019829(cation-transporting ATPase activity);GO:0046872(metal ion binding);GO:0070588(calcium ion transmembrane transport);GO:0005789(endoplasmic reticulum membrane);GO:0005515(protein binding);GO:0006942(regulation of striated muscle contraction);GO:0042803(protein homodimerization activity);GO:0070509(calcium ion import);GO:0031674(I band);GO:0090076(relaxation of skeletal muscle);GO:0051659(maintenance of mitochondrion location);GO:0051561(positive regulation of mitochondrial calcium ion concentration);GO:0045988(negative regulation of striated muscle contraction);GO:0032471(negative regulation of endoplasmic reticulum calcium ion concentration);GO:0032470(positive regulation of endoplasmic reticulum calcium ion concentration);GO:0031673(H zone);GO:0031448(positive regulation of fast-twitch skeletal muscle fiber contraction);GO:0008637(apoptotic mitochondrial changes);GO:0070059(intrinsic apoptotic signaling pathway in response to endoplasmic reticulum stress);GO:0034976(response to endoplasmic reticulum stress)
ciRNA42	GO:0004871(signal transducer activity);GO:0035556(intracellular signal transduction);GO:0005515(protein binding);GO:0030032(lamellipodium assembly);GO:0006928(cellular component movement);GO:0045840(positive regulation of mitosis);GO:2000278(regulation of DNA biosynthetic process)

ciRNA43	GO:0003824(catalytic activity);GO:0004252(serine-type endopeptidase activity);GO:0006508(proteolysis);GO:0008233(peptidase activity);GO:0008236(serine-type peptidase activity);GO:0016787(hydrolase activity);GO:0005515(protein binding);GO:0005509(calcium ion binding)
ciRNA44	GO:0005515(protein binding);GO:0042802(identical protein binding);GO:0042803(protein homodimerization activity);GO:0006936(muscle contraction);GO:0031430(M band);GO:0030017(sarcomere)
ciRNA45	GO:0005622(intracellular);GO:0007165(signal transduction);GO:0005096(GTPase activator activity);GO:0007264(small GTPase mediated signal transduction);GO:0016020(membrane);GO:0005515(protein binding);GO:0006810(transport);GO:0016887(ATPase activity);GO:0006200(ATP catabolic process);GO:0048365(Rac GTPase
ciRNA46	GO:0005515(protein binding);GO:0009986(cell surface);GO:0031091(platelet alpha granule);GO:0019722(calcium-mediated signaling);GO:0030168(platelet activation)
ciRNA47	GO:0006810(transport);GO:0016020(membrane);GO:0016021(integral component of membrane);GO:0005739(mitochondrion)
ciRNA48	GO:0005515(protein binding);GO:0031012(extracellular matrix);GO:0005622(intracellular);GO:0005615(extracellular space);GO:0070062(extracellular vesicular exosome);GO:0005583(fibrillar collagen trimer);GO:0048251(elastic fiber assembly);GO:0032963(collagen metabolic process);GO:0016337(single organismal cell-cell adhesion);GO:0005576(extracellular region);GO:0006629(lipid metabolic process);GO:0030198(extracellular matrix organization);GO:0030199(collagen fibril organization);GO:0007160(cell-matrix adhesion);GO:0008201(heparin binding);GO:0005518(collagen binding);GO:0006631(fatty acid metabolic process);GO:0006641(triglyceride metabolic process);GO:0005578(proteinaceous extracellular matrix);GO:0043506(regulation of JUN kinase activity);GO:0043206(extracellular fibril organization)
ciRNA49	GO:0005515(protein binding);GO:0005488(binding);GO:0032039(integrator complex);GO:0016180(snRNA processing)
ciRNA5	GO:0005515(protein binding);GO:0008092(cytoskeletal protein binding);GO:0070062(extracellular vesicular exosome);GO:0051015(actin filament binding);GO:0030018(Z disc);GO:0005523(tropomyosin binding);GO:0071691(cardiac muscle thin filament assembly);GO:0031005(filamin binding);GO:0001725(stress fiber)
ciRNA50	GO:0000166(nucleotide binding);GO:0004672(protein kinase activity);GO:0004674(protein serine/threonine kinase activity);GO:0005524(ATP binding);GO:0006468(protein phosphorylation);GO:0016301(kinase activity);GO:0016310(phosphorylation);GO:0016740(transferase activity);GO:0016772("transferase activity, transferring phosphorus-containing
ciRNA51	GO:0000166(nucleotide binding);GO:0005525(GTP binding);GO:0007264(small GTPase mediated signal transduction);GO:0015031(protein transport);GO:0003924(GTPase activity);GO:0006184(GTP catabolic process);GO:0006886(intracellular protein transport);GO:0006913(nucleocytoplasmic transport);GO:0007165(signal
ciRNA52	GO:0000166(nucleotide binding);GO:0004672(protein kinase activity);GO:0004674(protein serine/threonine kinase activity);GO:0005524(ATP binding);GO:0006468(protein phosphorylation);GO:0007165(signal transduction);GO:0016301(kinase activity);GO:0016310(phosphorylation);GO:0016740(transferase activity);GO:0016772("transferase activity, transferring
ciRNA53	GO:0005509(calcium ion binding);GO:0005576(extracellular region);GO:0007155(cell adhesion);GO:0005515(protein binding);GO:0016525(negative regulation of angiogenesis);GO:0008201(heparin binding);GO:0005615(extracellular space);GO:0005604(basement membrane);GO:0090023(positive regulation of neutrophil chemotaxis);GO:0001938(positive regulation of endothelial cell
ciRNA54	GO:0003774(motor activity);GO:0005524(ATP binding);GO:0016459(myosin complex);GO:0051015(actin filament binding);GO:0000166(nucleotide binding);GO:0003779(actin binding);GO:0008152(metabolic process);GO:0005515(protein binding);GO:0005737(cytoplasm);GO:0005634(nucleus);GO:0016887(ATPase activity);GO:0006200(ATP catabolic process);GO:0032982(myosin filament);GO:0005925(focal adhesion);GO:0006936(muscle contraction);GO:0030898(actin-dependent ATPase activity);GO:0002026(regulation of the force of heart contraction);GO:0000146(microfilament motor activity);GO:0030049(muscle filament sliding);GO:0007512(adult heart development);GO:0055010(ventricular cardiac muscle tissue morphogenesis);GO:0006941(striated muscle contraction);GO:0002027(regulation of heart rate);GO:0030018(Z disc);GO:0001725(stress fiber);GO:0030016(myofibril)
ciRNA55	GO:0005089(Rho guanyl-nucleotide exchange factor activity);GO:0032321(positive regulation of Rho GTPase activity);GO:0035023(regulation of Rho protein signal transduction)
ciRNA56	GO:0005083(small GTPase regulator activity);GO:0006886(intracellular protein transport);GO:0016192(vesicle-mediated transport);GO:0050790(regulation of catalytic activity);GO:0005765(lysosomal membrane);GO:0030897(HOPS complex);GO:0005515(protein binding)
ciRNA57	GO:0004872(receptor activity);GO:0007275(multicellular organismal development);GO:0016020(membrane);GO:0016021(integral component of membrane);GO:0005515(protein binding);GO:0021591(ventricular system development);GO:0014911(positive regulation of smooth muscle cell migration);GO:0014912(negative regulation of smooth muscle cell
ciRNA58	GO:0003857(3-hydroxyacyl-CoA dehydrogenase activity);GO:0006631(fatty acid metabolic process);GO:0016491(oxidoreductase activity);GO:0016616("oxidoreductase activity, acting on the CH-OH group of donors, NAD or NADP as acceptor");GO:0050662(coenzyme binding);GO:0055114(oxidation-reduction process);GO:0070403(NAD+ binding);GO:0050660(flavin adenine dinucleotide binding);GO:0051287(NAD binding);GO:0046168(glycerol-3-phosphate catabolic process);GO:0005737(cytoplasm);GO:0005739(mitochondrion);GO:0005634(nucleus);GO:0005743(mitochondrial inner membrane)
ciRNA59	GO:0005089(Rho guanyl-nucleotide exchange factor activity);GO:0032321(positive regulation of Rho GTPase activity);GO:0035023(regulation of Rho protein signal transduction);GO:0005515(protein bindi
ciRNA6	GO:0005576(extracellular region);GO:0019229(regulation of vasoconstriction);GO:0005615(extracellular space);GO:0005179(hormone activity);GO:0042310(vasoconstriction);GO:0030593(neutrophil chemotaxis);GO:0048016(inositol phosphate-mediated signaling);GO:0007166(cell surface receptor signaling pathway);GO:0046887(positive regulation of hormone secretion);GO:0030072(peptide hormone
ciRNA60	GO:0005089(Rho guanyl-nucleotide exchange factor activity);GO:0032321(positive regulation of Rho GTPase activity);GO:0035023(regulation of Rho protein signal transduction);GO:0005515(protein bindi
ciRNA61	GO:0000226(microtubule cytoskeleton organization);GO:0001578(microtubule bundle formation);GO:0005874(microtubule);GO:0006915(apoptotic process);GO:0008017(microtubule binding);GO:0008152(metabolic process);GO:0016787(hydrolase activity);GO:0005730(nucleolus);GO:0030054(cell junction);GO:0003677(DNA binding);GO:0042995(cell projection);GO:0048471(perinuclear region of cytoplasm);GO:0005829(cytosol);GO:0005737(cytoplasm);GO:0005515(protein binding);GO:0005634(nucleus);GO:0051015(actin filament binding);GO:0048812(neuron projection morphogenesis);GO:0015631(tubulin binding);GO:0045202(synapse);GO:0048487(beta-tubulin binding);GO:0097194(execution phase of apoptosis);GO:0007420(brain development);GO:0030425(dendrite);GO:0042802(identical protein binding);GO:0043025(neuronal cell body);GO:0015630(microtubule cytoskeleton);GO:0007399(nervous system development)
ciRNA62	GO:0005737(cytoplasm);GO:0005886(plasma membrane);GO:0043235(receptor complex);GO:0005765(lysosomal membrane);()
ciRNA63	GO:0005515(protein binding)
ciRNA64	GO:0003677(DNA binding);GO:0003899(DNA-directed RNA polymerase activity);GO:0006351("transcription, DNA-templated");GO:0005739(mitochondrion);GO:0005515(protein binding);GO:0044822(poly(A) RNA binding);GO:0042645(mitochondrial nucleoid)
ciRNA65	GO:0000166(nucleotide binding);GO:0000910(cytokinesis);GO:0003777(microtubule motor activity);GO:0005524(ATP binding);GO:0005794(Golgi apparatus);GO:0005871(kinesin complex);GO:0005874(microtubule);GO:0007018(microtubule-based movement);GO:0008017(microtubule binding);GO:0008152(metabolic process);GO:0005819(spindle);GO:0019901(protein kinase binding);GO:0001578(microtubule bundle formation)
ciRNA66	GO:0000166(nucleotide binding);GO:0005524(ATP binding);GO:0006200(ATP catabolic process);GO:0008152(metabolic process);GO:0016887(ATPase activity);GO:0017111(nucleoside-triphosphatase acti
ciRNA67	GO:0005515(protein binding);GO:0005615(extracellular space);GO:0002244(hematopoietic progenitor cell differentiation);GO:0030513(positive regulation of BMP signaling pathway)
ciRNA68	GO:0005515(protein binding);GO:0045893("positive regulation of transcription, DNA-templated");GO:0006357(regulation of transcription from RNA polymerase II promoter);GO:0005737(cytoplasm);GO:0006351("transcription, DNA-templated");GO:0005634(nucleus);GO:0005102(receptor binding);GO:0016922(ligand-dependent nuclear receptor binding);GO:0006337(nucleosome disassembly);GO:0000790(nuclear chromatin);GO:0035257(nuclear hormone receptor binding);GO:0030374(ligand-dependent nuclear receptor transcription coactivator activity);GO:0006338(chromatin remodeling);GO:0008134(transcription factor binding);GO:0002052(positive regulation of neuroblast proliferation);GO:0003407(neural retina development);GO:0003139(secondary heart field specification);GO:0003007(heart morphogenesis);GO:0003219(cardiac right ventricle formation);GO:0071564(npBAF complex);GO:0071565(nBAF complex);GO:0043393(regulation of protein binding);GO:0042692(muscle cell differentiation)
ciRNA69	GO:0005515(protein binding);GO:0044232(organelle membrane contact site);GO:0035091(phosphatidylinositol binding);GO:0031234(extrinsic component of cytoplasmic side of plasma membrane);GO:0031227(intrinsic component of endoplasmic reticulum membrane);GO:0031210(phosphatidylcholine binding);GO:0016020(membrane);GO:0008429(phosphatidylethanolamine
ciRNA7	GO:0008289(lipid binding)
ciRNA70	GO:0003824(catalytic activity);GO:0008152(metabolic process);GO:0016020(membrane);GO:0006487(protein N-linked glycosylation);GO:0030176(integral component of endoplasmic reticulum membrane);GO:0047874(dolichyldiphosphatase activity)
ciRNA71	GO:0004181(metallocarboxypeptidase activity);GO:0006508(proteolysis);GO:0008270(zinc ion binding);GO:0005737(cytoplasm);GO:0005634(nucleus);GO:0005829(cytosol);GO:0015631(tubulin binding);GO:0035608(protein deglutamylation);GO:0035611(protein branching point deglutamylation)
ciRNA72	GO:0007165(signal transduction);GO:0035091(phosphatidylinositol binding);GO:0043231(intracellular membrane-bounded organelle);GO:0005829(cytosol);GO:0005737(cytoplasm);GO:0005515(protein binding);GO:0010008(endosome membrane);GO:0005794(Golgi apparatus);GO:0005769(early endosome);GO:0016023(cytoplasmic membrane-bounded
ciRNA73	GO:0000175(3'-5'-exoribonuclease activity);GO:0003723(RNA binding);GO:0004654(polyribonucleotide nucleotidyltransferase activity);GO:0006396(RNA processing);GO:0006402(mRNA catabolic process);GO:0090503("RNA phosphodiester bond hydrolysis, exonucleolytic");GO:0051260(protein homooligomerization);GO:0005739(mitochondrion);GO:0005737(cytoplasm);GO:0005515(protein
ciRNA74	GO:0006810(transport);GO:0006886(intracellular protein transport);GO:0015031(protein transport);GO:0016020(membrane);GO:0016192(vesicle-mediated transport);GO:0030117(membrane coat)
ciRNA75	GO:0005215(transporter activity);GO:0006810(transport);GO:0016020(membrane);GO:0016021(integral component of membrane);GO:0022857(transmembrane transporter activity);GO:0022891(substrate-specific transmembrane transporter activity);GO:0055085(transmembrane transport)
ciRNA76	GO:0007155(cell adhesion);GO:0007229(integrin-mediated signaling pathway);GO:0008305(integrin complex);GO:0016020(membrane);GO:0016021(integral component of membrane);GO:0005515(protein binding);GO:0005737(cytoplasm);GO:0005927(muscle tendon junction);GO:0016477(cell migration);GO:0042383(sarcolemma);GO:0031594(neuromuscular junction);GO:0048514(blood vessel morphogenesis);GO:0043236(laminin binding);GO:0034677(integrin alpha7-beta1 complex)
ciRNA77	GO:0003677(DNA binding);GO:0003899(DNA-directed RNA polymerase activity);GO:0006351("transcription, DNA-templated");GO:0016740(transferase activity);GO:0016779(nucleotidyltransferase activity);GO:0032549(ribonucleoside binding);GO:0032728(positive regulation of interferon-beta production);GO:0005666(DNA-directed RNA polymerase III complex);GO:0045089(positive regulation of
ciRNA78	GO:0005634(nucleus);GO:0008270(zinc ion binding);GO:0005515(protein binding);GO:0035102(PRC1 complex);GO:0031519(PcG protein complex);GO:0016574(histone ubiquitination);GO:0003682(chromatin binding);GO:0016604(nuclear body);GO:0001739(sex chromatin);GO:0071300(cellular response to retinoic acid)

ciRNA79	GO:0005634(nucleus);GO:0008270(zinc ion binding);GO:0008285(negative regulation of cell proliferation);GO:0016568(chromatin modification);GO:0016573(histone acetylation);GO:0035064(methylated histone binding);GO:0043065(positive regulation of apoptotic process);GO:0046872(metal ion binding);GO:0005515(protein binding);GO:0006915(apoptotic process);GO:0000123(histone acetyltransferase complex);GO:0045892("negative regulation of transcription, DNA-templated");GO:0007050(cell cycle arrest);GO:0003713(transcription coactivator activity);GO:0006260(DNA replication);GO:0006473(protein acetylation);GO:0043983(histone H4-K12 acetylation);GO:0043966(histone H3 acetylation);GO:0006978("DNA damage response, signal transduction by p53 class mediator resulting in transcription of p21 class mediator");GO:0045926(negative regulation of growth);GO:0043982(histone H4-K8 acetylation);GO:0043981(histone H4-K5 acetylation)
ciRNA8	GO:0003824(catalytic activity);GO:0008081(phosphoric diester hydrolase activity);GO:0008152(metabolic process);GO:0046872(metal ion binding);GO:0005515(protein binding);GO:0003676(nucleic acid binding);GO:0008270(zinc ion binding);GO:0005886(plasma membrane);GO:0005634(nucleus);GO:0003723(RNA binding);GO:0051289(protein homotetramerization);GO:0051607(defense response to virus);GO:0032567(dGTP binding);GO:0008832(dGTPase activity);GO:0046061(dATP catabolic process);GO:0006203(dGTP catabolic process);GO:0045088(regulation of innate immune response)
ciRNA80	GO:0008270(zinc ion binding);GO:0046872(metal ion binding);GO:0005515(protein binding);GO:0005634(nucleus);GO:0042393(histone binding);GO:0070776(MOZ/MORF histone acetyltransferase complex);GO:0043966(histone H3 acetylation)
ciRNA81	GO:0004672(protein kinase activity);GO:0004713(protein tyrosine kinase activity);GO:0005524(ATP binding);GO:0006468(protein phosphorylation);GO:0016772("transferase activity, transferring phosphorus-containing groups");GO:0018108(peptidyl-tyrosine phosphorylation);GO:0005515(protein binding);GO:0003676(nucleic acid binding);GO:0004674(protein serine/threonine kinase activity);GO:0070062(extracellular vesicular exosome);GO:0005509(calcium ion binding);GO:0005737(cytoplasm);GO:0005634(nucleus);GO:0005794(Golgi apparatus);GO:0050790(regulation of catalytic activity);GO:0051015(actin filament binding);GO:0030018(Z disc);GO:0019899(enzyme binding);GO:0002020(protease binding);GO:0042802(identical protein binding);GO:0007076(mitotic chromosome condensation);GO:0045859(regulation of protein kinase activity);GO:0019901(protein kinase binding);GO:0031674(I band);GO:0031430(M band);GO:0051371(muscle alpha-actinin binding);GO:0005865(striated muscle thin filament);GO:0051592(response to calcium ion);GO:0042805(actinin binding);GO:0055008(cardiac muscle tissue morphogenesis);GO:0055003(cardiac myofibril assembly);GO:0048769(sarcomerogenesis);GO:0048739(cardiac muscle fiber development);GO:0045214(sarcomere organization);GO:0030241(skeletal muscle myosin thick filament assembly);GO:0030240(skeletal muscle thin filament assembly);GO:0003300(cardiac muscle hypertrophy);GO:0008307(structural constituent of muscle);GO:0060048(cardiac muscle contraction);GO:0000794(condensed nuclear chromosome);GO:0043621(protein self-association);GO:0001701(in utero embryonic development);GO:0031672(A band);GO:0006936(muscle contraction);GO:0007507(heart development);GO:0001756(somitogenesis);GO:0003007(heart morphogenesis);GO:0007512(adult heart development);GO:0030506(ankyrin binding);GO:1901897(regulation of relaxation of cardiac muscle);GO:0030017(sarcomere);GO:0043056(forward locomotion);GO:0055002(striated muscle cell development)
ciRNA82	GO:0001932(regulation of protein phosphorylation);GO:0005085(guanyl-nucleotide exchange factor activity);GO:0005622(intracellular);GO:0005952(cAMP-dependent protein kinase complex);GO:0007264(small GTPase mediated signal transduction);GO:0008603(cAMP-dependent protein kinase regulator activity);GO:0035556(intracellular signal transduction);GO:0043547(positive regulation of GTPase activity);GO:0045859(regulation of protein kinase activity);GO:0051056(regulation of small GTPase mediated signal transduction);GO:0005515(protein binding);GO:0005829(cytosol);GO:0043087(regulation of GTPase activity);GO:0017157(regulation of exocytosis);GO:0030073(insulin secretion);GO:0017016(Ras GTPase binding);GO:0017156(calcium ion-dependent exocytosis);GO:0019933(cAMP-mediated signaling);GO:0005088(Ras guanyl-nucleotide exchange factor activity);GO:0032318(regulation of Ras GTPase activity);GO:0030552(cAMP binding)
ciRNA83	GO:0005794(Golgi apparatus);GO:0006486(protein glycosylation);GO:0016020(membrane);GO:0016021(integral component of membrane);GO:0016740(transferase activity);GO:0016757("transferase activity, transferring glycosyl groups");GO:0046872(metal ion binding);GO:0006493(protein O-linked glycosylation);GO:0004653(polypeptide N-acetylgalactosaminyltransferase activity)
ciRNA84	GO:0005215(transporter activity);GO:0005452(inorganic anion exchanger activity);GO:0006810(transport);GO:0006820(anion transport);GO:0008509(anion transmembrane transporter activity);GO:0016020(membrane);GO:0016021(integral component of membrane);GO:0005886(plasma membrane);GO:0051453(regulation of intracellular pH);GO:0015301(anion:anion antiporter activity)
ciRNA85	GO:0000166(nucleotide binding);GO:0000287(magnesium ion binding);GO:0004672(protein kinase activity);GO:0004674(protein serine/threonine kinase activity);GO:0005524(ATP binding);GO:0006468(protein phosphorylation);GO:0016301(kinase activity);GO:0016310(phosphorylation);GO:0016740(transferase activity);GO:0016772("transferase activity, transferring phosphorus-
ciRNA86	GO:0000166(nucleotide binding);GO:0000287(magnesium ion binding);GO:0004672(protein kinase activity);GO:0004674(protein serine/threonine kinase activity);GO:0005524(ATP binding);GO:0006468(protein phosphorylation);GO:0016301(kinase activity);GO:0016310(phosphorylation);GO:0016740(transferase activity);GO:0016772("transferase activity, transferring phosphorus-
ciRNA87	GO:0000166(nucleotide binding);GO:0000287(magnesium ion binding);GO:0004672(protein kinase activity);GO:0004674(protein serine/threonine kinase activity);GO:0005524(ATP binding);GO:0006468(protein phosphorylation);GO:0016301(kinase activity);GO:0016310(phosphorylation);GO:0016740(transferase activity);GO:0016772("transferase activity, transferring phosphorus-
ciRNA88	GO:0000166(nucleotide binding);GO:0000287(magnesium ion binding);GO:0004672(protein kinase activity);GO:0004674(protein serine/threonine kinase activity);GO:0005524(ATP binding);GO:0006468(protein phosphorylation);GO:0016301(kinase activity);GO:0016310(phosphorylation);GO:0016740(transferase activity);GO:0016772("transferase activity, transferring phosphorus-
ciRNA89	GO:0000166(nucleotide binding);GO:0000287(magnesium ion binding);GO:0004672(protein kinase activity);GO:0004674(protein serine/threonine kinase activity);GO:0005524(ATP binding);GO:0006468(protein phosphorylation);GO:0016301(kinase activity);GO:0016310(phosphorylation);GO:0016740(transferase activity);GO:0016772("transferase activity, transferring phosphorus-
ciRNA9	GO:0000166(nucleotide binding);GO:0004812(aminoacyl-tRNA ligase activity);GO:0004818(glutamate-tRNA ligase activity);GO:0004827(proline-tRNA ligase activity);GO:0005524(ATP binding);GO:0005737(cytoplasm);GO:0006412(translation);GO:0006418(tRNA aminoacylation for protein translation);GO:0006424(glutamyl-tRNA aminoacylation);GO:0006433(prolyl-tRNA
ciRNA90	GO:0000166(nucleotide binding);GO:0000287(magnesium ion binding);GO:0004672(protein kinase activity);GO:0004674(protein serine/threonine kinase activity);GO:0005524(ATP binding);GO:0006468(protein phosphorylation);GO:0016301(kinase activity);GO:0016310(phosphorylation);GO:0016740(transferase activity);GO:0016772("transferase activity, transferring phosphorus-
ciRNA91	GO:0003824(catalytic activity);GO:0009058(biosynthetic process);GO:0030170(pyridoxal phosphate binding);GO:0044822(poly(A) RNA binding);GO:0042803(protein homodimerization activity);GO:0005739(mitochondrion);GO:0006520(cellular amino acid metabolic process);GO:0006103(2-oxoglutarate metabolic process);GO:0016212(kynurenine-oxoglutarate transaminase activity);GO:0047315(kynurenine-glyoxylate transaminase activity);GO:0070189(kynurenine metabolic process)
ciRNA92	GO:0005634(nucleus);GO:0005737(cytoplasm);GO:0032793(positive regulation of CREB transcription factor activity)
ciRNA93	GO:0016021(integral component of membrane);GO:0005515(protein binding);GO:0007156(homophilic cell adhesion);GO:0005737(cytoplasm);GO:0009986(cell surface);GO:0006919(activation of cysteine-type endopeptidase activity involved in apoptotic process);GO:0042802(identical protein binding);GO:0050925(negative regulation of negative chemotaxis);GO:0030275(LRR domain
ciRNA94	GO:0005515(protein binding)
ciRNA95	GO:0005515(protein binding)
ciRNA96	GO:0031668(cellular response to extracellular stimulus)
ciRNA97	GO:0003824(catalytic activity);GO:0008152(metabolic process);GO:0005829(cytosol);GO:0005515(protein binding);GO:0051018(protein kinase A binding);GO:0005737(cytoplasm);GO:0005634(nucleus);GO:0019901(protein kinase binding);GO:0034237(protein kinase A regulatory subunit binding)
ciRNA98	GO:0006355("regulation of transcription, DNA-templated");GO:0005667(transcription factor complex);GO:0005515(protein binding);GO:0016592(mediator complex)
circRNA1	GO:0003824(catalytic activity);GO:0008152(metabolic process);GO:0008484(sulfuric ester hydrolase activity);GO:0070062(extracellular vesicular exosome)
circRNA10	GO:0016525(negative regulation of angiogenesis);GO:0005737(cytoplasm);GO:0005515(protein binding);GO:0016021(integral component of membrane);GO:0009986(cell surface);GO:0004872(receptor activity);GO:0001726(ruffle);GO:0035329(hippo signaling);GO:0008180(COP9 signalosome);GO:0030139(endocytic vesicle);GO:0043116(negative regulation of vascular
circRNA100	GO:0005515(protein binding);GO:0005488(binding);GO:0005886(plasma membrane);GO:0005829(cytosol);GO:0019901(protein kinase binding);GO:0005802(trans-Golgi network);GO:0008104(protein localization);GO:0012505(endomembrane system)
circRNA101	GO:0003677(DNA binding);GO:0005488(binding);GO:0005515(protein binding);GO:0000785(chromatin);GO:0008285(negative regulation of cell proliferation);GO:0042127(regulation of cell proliferation);GO:0007064(mitotic sister chromatid cohesion)
circRNA102	()
circRNA103	()
circRNA104	()
circRNA105	()
circRNA106	GO:0003779(actin binding);GO:0005856(cytoskeleton);GO:0030036(actin cytoskeleton organization);GO:0007010(cytoskeleton organization);GO:0008360(regulation of cell shape);GO:0031643(positive regulation of myelination);GO:0014003(oligodendrocyte development);GO:0030027(lamellipodium);GO:0030032(lamellipodium assembly)
circRNA107	GO:0000166(nucleotide binding);GO:0004672(protein kinase activity);GO:0004674(protein serine/threonine kinase activity);GO:0005524(ATP binding);GO:0006468(protein phosphorylation);GO:0016772("transferase activity, transferring phosphorus-containing groups");GO:0004713(protein tyrosine kinase activity);GO:0005515(protein
circRNA108	GO:0016012(sarcoglycan complex);GO:0016021(integral component of membrane);GO:0005515(protein binding);GO:0042383(sarcolemma);GO:0016011(dystroglycan complex)
circRNA109	GO:0008270(zinc ion binding);GO:0046872(metal ion binding);GO:0005515(protein binding)
circRNA11	GO:0001525(angiogenesis);GO:0016337(single organismal cell-cell adhesion);GO:0048870(cell motility);GO:0005615(extracellular space);GO:0005737(cytoplasm);GO:0005515(protein binding);GO:0005102(receptor binding);GO:0051965(positive regulation of synapse assembly);GO:0042802(identical protein binding);GO:0042325(regulation of phosphorylation);GO:0036458(hepatocyte growth factor binding);GO:0071625(vocalization behavior);GO:0090050(positive regulation of cell migration involved in sprouting angiogenesis)
circRNA110	GO:0008270(zinc ion binding);GO:0046872(metal ion binding);GO:0005515(protein binding)
circRNA111	GO:0000166(nucleotide binding);GO:0003774(motor activity);GO:0005524(ATP binding);GO:0008152(metabolic process);GO:0016459(myosin complex);GO:0005515(protein binding);GO:0048471(perinuclear region of cytoplasm);GO:0005654(nucleoplasm);GO:0048812(neuron projection morphogenesis);GO:0014065(phosphatidylinositol 3-kinase signaling)
circRNA112	GO:0005089(Rho guanyl-nucleotide exchange factor activity);GO:0032321(positive regulation of Rho GTPase activity);GO:0035023(regulation of Rho protein signal transduction);GO:0005515(protein binding);GO:0005085(guanyl-nucleotide exchange factor activity);GO:0043547(positive regulation of GTPase activity);GO:0043065(positive regulation of apoptotic process);GO:0043025(neuronal cell

circRNA113	GO:0005089(Rho guanyl-nucleotide exchange factor activity);GO:0032321(positive regulation of Rho GTPase activity);GO:0035023(regulation of Rho protein signal transduction);GO:0005515(protein binding);GO:0005085(guanyl-nucleotide exchange factor activity);GO:0043547(positive regulation of GTPase activity);GO:0043065(positive regulation of apoptotic process);GO:0043025(neuronal cell body);GO:0043005(neuron projection);GO:0019901(protein kinase binding);GO:0043234(protein complex);GO:0002244(hematopoietic progenitor cell differentiation);GO:0030027(lamellipodium);GO:0030032(lamellipodium assembly)
circRNA114	GO:0005515(protein binding);GO:0003779(actin binding);GO:0015629(actin cytoskeleton);GO:0005737(cytoplasm);GO:0016567(protein ubiquitination);GO:0031463(Cul3-RING ubiquitin ligase complex)
circRNA115	GO:0005096(GTPase activator activity);GO:0005622(intracellular);GO:0007165(signal transduction);GO:0043547(positive regulation of GTPase activity);GO:0005515(protein binding);GO:0005829(cytosol);GO:0043066(negative regulation of apoptotic process);GO:0007010(cytoskeleton organization);GO:0032321(positive regulation of Rho GTPase activity);GO:0005100(Rho
circRNA116	GO:0005096(GTPase activator activity);GO:0005622(intracellular);GO:0007165(signal transduction);GO:0043547(positive regulation of GTPase activity);GO:0005515(protein binding);GO:0005829(cytosol);GO:0043066(negative regulation of apoptotic process);GO:0007010(cytoskeleton organization);GO:0032321(positive regulation of Rho GTPase activity);GO:0005100(Rho
circRNA117	GO:0005096(GTPase activator activity);GO:0005622(intracellular);GO:0007165(signal transduction);GO:0043547(positive regulation of GTPase activity);GO:0005515(protein binding);GO:0005829(cytosol);GO:0043066(negative regulation of apoptotic process);GO:0007010(cytoskeleton organization);GO:0032321(positive regulation of Rho GTPase activity);GO:0005100(Rho
circRNA118	GO:0003676(nucleic acid binding);GO:0046872(metal ion binding)
circRNA119	GO:0044822(poly(A) RNA binding);GO:0004843(ubiquitin-specific protease activity);GO:0071108(protein K48-linked deubiquitination)
circRNA12	GO:0000166(nucleotide binding);GO:0003777(microtubule motor activity);GO:0005524(ATP binding);GO:0005871(kinesin complex);GO:0005874(microtubule);GO:0007018(microtubule-based movement);GO:0008017(microtubule binding);GO:0008152(metabolic process);GO:0035091(phosphatidylinositol binding);GO:0005515(protein binding);GO:0080025("phosphatidylinositol-3,5-bisphosphate binding");GO:0032266(phosphatidylinositol-3-phosphate binding);GO:0005769(early endosome);GO:0007173(epidermal growth factor receptor signaling pathway);GO:0001919(regulation of receptor recycling);GO:0005768(endosome);GO:0045022(early endosome to late endosome transport);GO:0043325("phosphatidylinositol-3,4-bisphosphate binding");GO:0032801(receptor catabolic process);GO:0005547("phosphatidylinositol-3,4,5-trisphosphate binding");GO:0008574(plus-end-directed microtubule motor activity);GO:0017137(Rab GTPase binding);GO:0007492(endoderm development);GO:0001704(formation of primary germ layer);GO:0006895(Golgi to endosome transport);GO:0008543(fibroblast growth factor receptor signaling pathway)
circRNA120	GO:0000166(nucleotide binding);GO:0005524(ATP binding);GO:0006200(ATP catabolic process);GO:0008152(metabolic process);GO:0016887(ATPase activity);GO:0017111(nucleoside-triphosphatase activity);GO:0051536(iron-sulfur cluster binding);GO:0005739(mitochondrion);GO:0016020(membrane);GO:0005737(cytoplasm)
circRNA121	GO:0008270(zinc ion binding);GO:0046872(metal ion binding);GO:0005515(protein binding)
circRNA122	GO:0005694(chromosome);GO:0006355("regulation of transcription, DNA-templated");GO:0018024(histone-lysine N-methyltransferase activity);GO:0034968(histone lysine methylation);GO:0005634(nucleus);GO:0006351("transcription, DNA-templated");GO:0008168(methyltransferase activity);GO:0016568(chromatin modification);GO:0016740(transferase activity);GO:0032259(methylation);GO:0005515(protein binding);GO:0018027(peptidyl-lysine dimethylation);GO:0018026(peptidyl-lysine monomethylation);GO:0016279(protein-lysine N-methyltransferase activity);GO:0002039(p53 binding)
circRNA123	GO:0005694(chromosome);GO:0006355("regulation of transcription, DNA-templated");GO:0018024(histone-lysine N-methyltransferase activity);GO:0034968(histone lysine methylation);GO:0005634(nucleus);GO:0006351("transcription, DNA-templated");GO:0008168(methyltransferase activity);GO:0016568(chromatin modification);GO:0016740(transferase activity);GO:0032259(methylation);GO:0005515(protein binding);GO:0018027(peptidyl-lysine dimethylation);GO:0018026(peptidyl-lysine monomethylation);GO:0016279(protein-lysine N-methyltransferase activity);GO:0002039(p53 binding)
circRNA124	GO:0003677(DNA binding);GO:0003700(sequence-specific DNA binding transcription factor activity);GO:0005634(nucleus);GO:0006355("regulation of transcription, DNA-templated");GO:0043565(sequence-specific DNA binding);GO:0045893("positive regulation of transcription, DNA-templated");GO:0006357(regulation of transcription from RNA polymerase II promoter);GO:0005737(cytoplasm);GO:0005515(protein binding);GO:0045892("negative regulation of transcription, DNA-templated")
circRNA125	GO:0005085(guanyl-nucleotide exchange factor activity);GO:0005622(intracellular);GO:0007165(signal transduction);GO:0007264(small GTPase mediated signal transduction);GO:0043547(positive regulation of GTPase activity);GO:0051056(regulation of small GTPase mediated signal transduction);GO:0005515(protein binding);GO:0007218(neuropeptide signaling pathway);GO:0007186(G-protein coupled receptor signaling pathway);GO:0048471(perinuclear region of cytoplasm);GO:0016020(membrane);GO:0005737(cytoplasm);GO:0008285(negative regulation of cell proliferation);GO:0043950(positive regulation of cAMP-mediated signaling);GO:0070374(positive regulation of ERK1 and ERK2 cascade);GO:0071321(cellular response to cGMP);GO:0061028(establishment of endothelial barrier);GO:0030552(cAMP binding);GO:0030165(PDZ domain binding);GO:0032320(positive regulation of Ras GTPase activity);GO:0045860(positive regulation of protein kinase activity);GO:2000481(positive regulation of cAMP-dependent protein kinase activity);GO:0050774(negative regulation of dendrite morphogenesis);GO:0031697(beta-1 adrenergic receptor binding);GO:1901888(regulation of cell junction assembly);GO:0046582(Rap GTPase activator activity);GO:0050699(WW domain binding);GO:0071880(adenylate cyclase-activating adrenergic receptor signaling pathway);GO:2000670(positive regulation of dendritic cell apoptotic process);GO:0032854(positive regulation of Rap GTPase activity);GO:0032486(Rap protein signal transduction);GO:0017034(Rap guanyl-nucleotide exchange factor activity);GO:0071320(cellular response to cAMP);GO:0005088(Ras guanyl-nucleotide exchange factor activity);GO:0005770(late endosome);GO:0031175(neuron projection development)
circRNA126	GO:0005085(guanyl-nucleotide exchange factor activity);GO:0005622(intracellular);GO:0007165(signal transduction);GO:0007264(small GTPase mediated signal transduction);GO:0043547(positive regulation of GTPase activity);GO:0051056(regulation of small GTPase mediated signal transduction);GO:0005515(protein binding);GO:0007218(neuropeptide signaling pathway);GO:0007186(G-protein coupled receptor signaling pathway);GO:0048471(perinuclear region of cytoplasm);GO:0016020(membrane);GO:0005737(cytoplasm);GO:0008285(negative regulation of cell proliferation);GO:0043950(positive regulation of cAMP-mediated signaling);GO:0070374(positive regulation of ERK1 and ERK2 cascade);GO:0071321(cellular response to cGMP);GO:0061028(establishment of endothelial barrier);GO:0030552(cAMP binding);GO:0030165(PDZ domain binding);GO:0032320(positive regulation of Ras GTPase activity);GO:0045860(positive regulation of protein kinase activity);GO:2000481(positive regulation of cAMP-dependent protein kinase activity);GO:0050774(negative regulation of dendrite morphogenesis);GO:0031697(beta-1 adrenergic receptor binding);GO:1901888(regulation of cell junction assembly);GO:0046582(Rap GTPase activator activity);GO:0050699(WW domain binding);GO:0071880(adenylate cyclase-activating adrenergic receptor signaling pathway);GO:2000670(positive regulation of dendritic cell apoptotic process);GO:0032854(positive regulation of Rap GTPase activity);GO:0032486(Rap protein signal transduction);GO:0017034(Rap guanyl-nucleotide exchange factor activity);GO:0071320(cellular response to cAMP);GO:0005088(Ras guanyl-nucleotide exchange factor activity);GO:0005770(late endosome);GO:0031175(neuron projection development)
circRNA127	GO:0003676(nucleic acid binding);GO:0005622(intracellular);GO:0006355("regulation of transcription, DNA-templated");GO:0046872(metal ion binding)
circRNA128	GO:0007155(cell adhesion);GO:0016020(membrane);GO:0016021(integral component of membrane);GO:0006898(receptor-mediated endocytosis);GO:0070062(extracellular vesicular exosome);GO:0005515(protein binding);GO:0005886(plasma membrane);GO:0032497(detection of lipopolysaccharide);GO:0031663(lipopolysaccharide-mediated signaling
circRNA129	GO:0005737(cytoplasm);GO:0005886(plasma membrane);GO:0005634(nucleus)
circRNA13	GO:0005634(nucleus);GO:0006355("regulation of transcription, DNA-templated");GO:0005515(protein binding);GO:0043565(sequence-specific DNA binding);GO:0010629(negative regulation of gene expression);GO:0042393(histone binding)
circRNA130	GO:0005813(centrosome);GO:0016020(membrane);GO:0005737(cytoplasm);GO:0005886(plasma membrane);GO:0005814(centriole)
circRNA131	GO:0005622(intracellular);GO:0006810(transport);GO:0046872(metal ion binding);GO:0043231(intracellular membrane-bounded organelle)
circRNA132	GO:0003676(nucleic acid binding);GO:0008270(zinc ion binding);GO:0005813(centrosome);GO:0005515(protein binding);GO:0015630(microtubule cytoskeleton);GO:0008017(microtubule binding);GO:0051010(microtubule plus-end binding);GO:0015631(tubulin binding);GO:0044354(macropinosome);GO:0035371(microtubule plus-end);GO:0031116(positive regulation of microtubule
circRNA133	GO:0005737(cytoplasm);GO:0005515(protein binding);GO:0000122(negative regulation of transcription from RNA polymerase II promoter);GO:0005886(plasma membrane);GO:0005634(nucleus);GO:0016575(histone deacetylation);GO:0004407(histone deacetylase activity);GO:0043065(positive regulation of apoptotic process);GO:0019899(enzyme
circRNA134	GO:0008270(zinc ion binding);GO:0046872(metal ion binding);GO:0005515(protein binding);()
circRNA135	GO:0005737(cytoplasm);GO:0017089(glycolipid transporter activity);GO:0046836(glycolipid transport);GO:0051861(glycolipid binding);GO:0070062(extracellular vesicular exosome);GO:0008289(lipid bin
circRNA136	GO:0000166(nucleotide binding);GO:0003824(catalytic activity);GO:0003989(acetyl-CoA carboxylase activity);GO:0004075(biotin carboxylase activity);GO:0005524(ATP binding);GO:0006633(fatty acid biosynthetic process);GO:0008152(metabolic process);GO:0016874(ligase activity);GO:0046872(metal ion binding);GO:0005515(protein binding);GO:0006084(acetyl-CoA metabolic process);GO:0051289(protein homotetramerization);GO:0005739(mitochondrion)
circRNA137	GO:0000166(nucleotide binding);GO:0003824(catalytic activity);GO:0003989(acetyl-CoA carboxylase activity);GO:0004075(biotin carboxylase activity);GO:0005524(ATP binding);GO:0006633(fatty acid biosynthetic process);GO:0008152(metabolic process);GO:0016874(ligase activity);GO:0046872(metal ion binding);GO:0005515(protein binding);GO:0006084(acetyl-CoA metabolic process);GO:0051289(protein homotetramerization);GO:0005739(mitochondrion)
circRNA138	GO:0000166(nucleotide binding);GO:0003824(catalytic activity);GO:0003989(acetyl-CoA carboxylase activity);GO:0004075(biotin carboxylase activity);GO:0005524(ATP binding);GO:0006633(fatty acid biosynthetic process);GO:0008152(metabolic process);GO:0016874(ligase activity);GO:0046872(metal ion binding);GO:0005515(protein binding);GO:0006084(acetyl-CoA metabolic process);GO:0051289(protein homotetramerization);GO:0005739(mitochondrion)
circRNA139	GO:0000166(nucleotide binding);GO:0003824(catalytic activity);GO:0003989(acetyl-CoA carboxylase activity);GO:0004075(biotin carboxylase activity);GO:0005524(ATP binding);GO:0006633(fatty acid biosynthetic process);GO:0008152(metabolic process);GO:0016874(ligase activity);GO:0046872(metal ion binding);GO:0005515(protein binding);GO:0006084(acetyl-CoA metabolic process);GO:0051289(protein homotetramerization);GO:0005739(mitochondrion)
circRNA14	GO:0005634(nucleus);GO:0006355("regulation of transcription, DNA-templated");GO:0005515(protein binding);GO:0043565(sequence-specific DNA binding);GO:0010629(negative regulation of gene expression);GO:0042393(histone binding)
circRNA140	GO:0000166(nucleotide binding);GO:0003774(motor activity);GO:0005524(ATP binding);GO:0008152(metabolic process);GO:0016459(myosin complex);GO:0005515(protein binding);GO:0030018(Z disc);GO:0001570(vasculogenesis);GO:0001701(in utero embryonic development);GO:0031941(filamentous actin);GO:0048739(cardiac muscle fiber development)
circRNA141	GO:0004725(protein tyrosine phosphatase activity);GO:0016311(dephosphorylation);GO:0016791(phosphatase activity);GO:0035335(peptidyl-tyrosine dephosphorylation);GO:0046872(metal ion binding);GO:0016020(membrane);GO:0005737(cytoplasm);GO:0005634(nucleus);GO:0046856(phosphatidylinositol dephosphorylation);GO:0004722(protein serine/threonine phosphatase activity);GO:0006470(protein dephosphorylation);GO:0004438(phosphatidylinositol-3-phosphatase activity)

circRNA142	GO:0004791(thioredoxin-disulfide reductase activity);GO:0016491(oxidoreductase activity);GO:0016668("oxidoreductase activity, acting on a sulfur group of donors, NAD(P) as acceptor");GO:0045454(cell redox homeostasis);GO:0050660(flavin adenine dinucleotide binding);GO:0050661(NADP binding);GO:0055114(oxidation-reduction process);GO:0005739(mitochondrion);GO:0005515(protein binding);GO:0030097(hemopoiesis);GO:0007507(heart development)
circRNA143	GO:0000166(nucleotide binding);GO:0004672(protein kinase activity);GO:0004674(protein serine/threonine kinase activity);GO:0005524(ATP binding);GO:0006468(protein phosphorylation);GO:0016772("transferase activity, transferring phosphorus-containing groups");GO:0004713(protein tyrosine kinase activity);GO:0070062(extracellular vesicular exosome);GO:0005515(protein binding);GO:0005886(plasma membrane);GO:0042803(protein homodimerization activity);GO:0042802(identical protein binding);GO:0046777(protein autophosphorylation);GO:2000401(regulation of lymphocyte migration);GO:0071593(lymphocyte aggregation)
circRNA144	GO:0000166(nucleotide binding);GO:0003924(GTPase activity);GO:0005525(GTP binding);GO:0006184(GTP catabolic process);GO:0032543(mitochondrial translation);GO:0032790(ribosome disassembly);GO:0005739(mitochondrion)
circRNA145	GO:0000166(nucleotide binding);GO:0003924(GTPase activity);GO:0005525(GTP binding);GO:0006184(GTP catabolic process);GO:0032543(mitochondrial translation);GO:0032790(ribosome disassembly);GO:0005739(mitochondrion)
circRNA146	GO:0005089(Rho guanyl-nucleotide exchange factor activity);GO:0032321(positive regulation of Rho GTPase activity);GO:0035023(regulation of Rho protein signal transduction);GO:0035556(intracellular signal transduction);GO:0005515(protein binding);GO:0060052(neurofilament cytoskeleton organization);GO:0021955(central nervous system neuron axonogenesis)
circRNA147	GO:0005634(nucleus);GO:0006886(intracellular protein transport);GO:0008536(Ran GTPase binding);GO:0005515(protein binding);GO:0005488(binding);GO:0044822(poly(A) RNA binding)
circRNA148	GO:0000166(nucleotide binding);GO:0000287(magnesium ion binding);GO:0004672(protein kinase activity);GO:0004674(protein serine/threonine kinase activity);GO:0005524(ATP binding);GO:0006468(protein phosphorylation);GO:0016301(kinase activity);GO:0016310(phosphorylation);GO:0016740(transferase activity);GO:0016772("transferase activity, transferring phosphorus-containing groups");GO:0005515(protein binding);GO:0004713(protein tyrosine kinase activity)
circRNA149	GO:0006886(intracellular protein transport);GO:0008536(Ran GTPase binding);GO:0005488(binding);GO:0005515(protein binding);GO:0008565(protein transporter activity);GO:0006610(ribosomal protein
circRNA15	GO:0005634(nucleus);GO:0006355("regulation of transcription, DNA-templated");GO:0005515(protein binding);GO:0043565(sequence-specific DNA binding);GO:0010629(negative regulation of gene expression);GO:0042393(histone binding)
circRNA150	GO:0005739(mitochondrion);GO:0008152(metabolic process);GO:0008260(3-oxoacid CoA-transferase activity);GO:0008410(CoA-transferase activity);GO:0016740(transferase activity);GO:0046952(ketone body catabolic process);GO:0046950(cellular ketone body metabolic process);GO:0042182(ketone catabolic process)
circRNA151	GO:0031929(TOR signaling);GO:0031932(TORC2 complex);GO:0005488(binding);GO:0005515(protein binding);GO:0032008(positive regulation of TOR signaling);GO:0031532(actin cytoskeleton reorganization);GO:0032956(regulation of actin cytoskeleton organization);GO:0051896(regulation of protein kinase B signaling);GO:0009790(embryo development);GO:0050731(positive regulation of peptidyl-tyrosine phosphorylation);GO:0032314(regulation of Rac GTPase activity);GO:0043022(ribosome binding);GO:0030838(positive regulation of actin filament polymerization)
circRNA152	GO:0003951(NAD+ kinase activity);GO:0006741(NADP biosynthetic process);GO:0008152(metabolic process);GO:0016310(phosphorylation);GO:0019674(NAD metabolic process);GO:0005739(mitochondrion);GO:0042803(protein homodimerization activity)
circRNA153	GO:0004222(metalloendopeptidase activity);GO:0005578(proteinaceous extracellular matrix);GO:0006508(proteolysis);GO:0008233(peptidase activity);GO:0008237(metallopeptidase activity);GO:0008270(zinc ion binding);GO:0031012(extracellular matrix);GO:0005515(protein binding);GO:0007160(cell-matrix adhesion);GO:0051603(proteolysis involved in cellular protein catabolic process);GO:0071356(cellular response to tumor necrosis factor);GO:0071347(cellular response to interleukin-1);GO:0016477(cell migration);GO:2001113(negative regulation of cellular response to hepatocyte growth factor stimulus);GO:1902548(negative regulation of cellular response to vascular endothelial growth factor stimulus);GO:1902203(negative regulation of hepatocyte growth factor receptor signaling pathway);GO:1901509(regulation of endothelial tube morphogenesis);GO:0030167(proteoglycan catabolic process);GO:0071773(cellular response to BMP stimulus);GO:0050727(regulation of inflammatory response);GO:0032331(negative regulation of chondrocyte differentiation)
circRNA154	GO:0004222(metalloendopeptidase activity);GO:0005578(proteinaceous extracellular matrix);GO:0006508(proteolysis);GO:0008233(peptidase activity);GO:0008237(metallopeptidase activity);GO:0008270(zinc ion binding);GO:0031012(extracellular matrix);GO:0005515(protein binding);GO:0007160(cell-matrix adhesion);GO:0051603(proteolysis involved in cellular protein catabolic process);GO:0071356(cellular response to tumor necrosis factor);GO:0071347(cellular response to interleukin-1);GO:0016477(cell migration);GO:2001113(negative regulation of cellular response to hepatocyte growth factor stimulus);GO:1902548(negative regulation of cellular response to vascular endothelial growth factor stimulus);GO:1902203(negative regulation of hepatocyte growth factor receptor signaling pathway);GO:1901509(regulation of endothelial tube morphogenesis);GO:0030167(proteoglycan catabolic process);GO:0071773(cellular response to BMP stimulus);GO:0050727(regulation of inflammatory response);GO:0032331(negative regulation of chondrocyte differentiation)
circRNA155	()
circRNA156	GO:0004672(protein kinase activity);GO:0004674(protein serine/threonine kinase activity);GO:0005085(guanyl-nucleotide exchange factor activity);GO:0005089(Rho guanyl-nucleotide exchange factor activity);GO:0005524(ATP binding);GO:0006468(protein phosphorylation);GO:0016772("transferase activity, transferring phosphorus-containing groups");GO:0032321(positive regulation of Rho GTPase
circRNA157	GO:0005515(protein binding)
circRNA158	NA
circRNA159	GO:0000166(nucleotide binding);GO:0004016(adenylate cyclase activity);GO:0006171(cAMP biosynthetic process);GO:0009190(cyclic nucleotide biosynthetic process);GO:0016020(membrane);GO:0016021(integral component of membrane);GO:0016829(lyase activity);GO:0016849(phosphorus-oxygen lyase activity);GO:0035556(intracellular signal transduction);GO:0005737(cytoplasm);GO:0005886(plasma membrane);GO:0030425(dendrite)
circRNA16	GO:0004591(oxoglutarate dehydrogenase (succinyl-transferring) activity);GO:0006099(tricarboxylic acid cycle);GO:0008152(metabolic process);GO:0016624("oxidoreductase activity, acting on the aldehyde or oxo group of donors, disulfide as acceptor");GO:0030976(thiamine pyrophosphate binding);GO:0055114(oxidation-reduction process);GO:0005739(mitochondrion);GO:0006091(generation of precursor metabolites and energy);GO:0002244(hematopoietic progenitor cell differentiation);GO:0006810(transport);GO:0015031(protein transport);GO:0016020(membrane);GO:0016021(integral component of membrane)
circRNA160	GO:0000166(nucleotide binding);GO:0004016(adenylate cyclase activity);GO:0006171(cAMP biosynthetic process);GO:0009190(cyclic nucleotide biosynthetic process);GO:0016020(membrane);GO:0016021(integral component of membrane);GO:0016829(lyase activity);GO:0016849(phosphorus-oxygen lyase activity);GO:0035556(intracellular signal
circRNA161	GO:0000166(nucleotide binding);GO:0004016(adenylate cyclase activity);GO:0006171(cAMP biosynthetic process);GO:0009190(cyclic nucleotide biosynthetic process);GO:0016020(membrane);GO:0016021(integral component of membrane);GO:0016829(lyase activity);GO:0016849(phosphorus-oxygen lyase activity);GO:0035556(intracellular signal
circRNA162	GO:0005634(nucleus);GO:1901031(regulation of response to reactive oxygen species);GO:0042593(glucose homeostasis);GO:0032868(response to insulin);GO:0051896(regulation of protein kinase B signalin
circRNA163	GO:0003824(catalytic activity);GO:0008152(metabolic process);GO:0016740(transferase activity);GO:0016746("transferase activity, transferring acyl groups");GO:0016747("transferase activity, transferring acyl groups other than amino-acyl groups");GO:0005739(mitochondrion);GO:0070062(extracellular vesicular exosome);GO:0003985(acetyl-CoA C-acetyltransferase activity);GO:0005743(mitochondrial
circRNA164	GO:0003677(DNA binding);GO:0006355("regulation of transcription, DNA-templated");GO:0007275(multicellular organismal development);GO:0005515(protein binding);GO:0005634(nucleus);GO:0071560(cellular response to transforming growth factor beta stimulus);GO:2000741(positive regulation of mesenchymal stem cell differentiation);GO:0032332(positive regulation of chondrocyte differentiation);GO:0061036(positive regulation of cartilage development);GO:0003700(sequence-specific DNA binding transcription factor activity);GO:0043565(sequence-specific DNA binding);GO:0044212(transcription regulatory region DNA binding);GO:0045892("negative regulation of transcription, DNA-templated");GO:0045944(positive regulation of transcription from RNA polymerase II promoter);GO:0000122(negative regulation of transcription from RNA polymerase II promoter);GO:0045165(cell fate commitment);GO:0010468(regulation of gene expression);GO:0021778(oligodendrocyte cell fate specification);GO:0001701(in utero embryonic development);GO:0045893("positive regulation of transcription, DNA-templated");GO:0000902(cell morphogenesis);GO:0030097(hemopoiesis);GO:0009791(post-embryonic development);GO:0051216(cartilage development);GO:0030218(erythrocyte differentiation);GO:0046982(protein heterodimerization activity);GO:0048709(oligodendrocyte differentiation);GO:0048821(erythrocyte development);GO:0055007(cardiac muscle cell differentiation);GO:0042692(muscle cell differentiation);GO:0016458(gene silencing)
circRNA165	GO:0000139(Golgi membrane);GO:0005198(structural molecule activity);GO:0005737(cytoplasm);GO:0005794(Golgi apparatus);GO:0006810(transport);GO:0006886(intracellular protein transport);GO:0015031(protein transport);GO:0016020(membrane);GO:0016192(vesicle-mediated transport);GO:0030117(membrane coat);GO:0030126(COPI vesicle coat);GO:0030663(COPI-coated vesicle membrane);GO:0031410(cytoplasmic vesicle);GO:0005488(binding);GO:0043231(intracellular membrane-bounded organelle);GO:0005515(protein binding);GO:0030137(COPI-coated vesicle)
circRNA166	GO:0003700(sequence-specific DNA binding transcription factor activity);GO:0005634(nucleus);GO:0006355("regulation of transcription, DNA-templated");GO:0035329(hippo signaling);GO:0005737(cytoplasm);GO:0005794(Golgi apparatus);GO:0005515(protein binding);GO:0003677(DNA binding);GO:0045944(positive regulation of transcription from RNA polymerase II promoter);GO:0042127(regulation of cell proliferation);GO:0007507(heart development);GO:0030903(notochord development);GO:0048339(paraxial mesoderm development);GO:0003143(embryonic heart tube morphogenesis);GO:0048368(lateral mesoderm development)
circRNA167	GO:0003700(sequence-specific DNA binding transcription factor activity);GO:0005634(nucleus);GO:0006355("regulation of transcription, DNA-templated");GO:0035329(hippo signaling);GO:0005737(cytoplasm);GO:0005794(Golgi apparatus);GO:0005515(protein binding);GO:0003677(DNA binding);GO:0045944(positive regulation of transcription from RNA polymerase II promoter);GO:0042127(regulation of cell proliferation);GO:0007507(heart development);GO:0030903(notochord development);GO:0048339(paraxial mesoderm development);GO:0003143(embryonic heart tube morphogenesis);GO:0048368(lateral mesoderm development)
circRNA168	GO:0006508(proteolysis);GO:0006511(ubiquitin-dependent protein catabolic process);GO:0008233(peptidase activity);GO:0008234(cysteine-type peptidase activity);GO:0016787(hydrolase activity);GO:0036459(ubiquitinyl hydrolase activity);GO:1902230(negative regulation of intrinsic apoptotic signaling pathway in response to DNA damage);GO:0034644(cellular response to UV)
circRNA169	GO:0016311(dephosphorylation);GO:0016791(phosphatase activity);GO:0016020(membrane);GO:0005515(protein binding);GO:0032851(positive regulation of Rab GTPase activity);GO:0017112(Rab guanyl-nucleotide exchange factor activity);GO:0042803(protein homodimerization activity);GO:0035091(phosphatidylinositol binding);GO:0050790(regulation of catalytic activity);GO:0019902(phosphatase binding);GO:0005774(vacuolar membrane);GO:0051262(protein tetramerization);GO:0019208(phosphatase regulator activity)
circRNA17	GO:0000166(nucleotide binding);GO:0004222(metalloendopeptidase activity);GO:0005524(ATP binding);GO:0006508(proteolysis);GO:0016020(membrane);GO:0017111(nucleoside-triphosphatase activity);GO:0008283(cell proliferation);GO:0006515(misfolded or incompletely synthesized protein catabolic process);GO:0007005(mitochondrion organization);GO:0005743(mitochondrial inner
circRNA170	GO:0005509(calcium ion binding);GO:0005515(protein binding)
circRNA171	GO:0003677(DNA binding);GO:0008907(integrase activity);GO:0015074(DNA integration);GO:0005515(protein binding);GO:0005615(extracellular space)
circRNA172	GO:0005622(intracellular);GO:0008270(zinc ion binding);GO:0046872(metal ion binding);GO:0005515(protein binding);GO:0005737(cytoplasm);GO:0016567(protein ubiquitination);GO:0004842(ubiquitin
circRNA173	GO:0005643(nuclear pore);GO:0006810(transport);GO:0005515(protein binding);GO:0000776(kinetochore);GO:0031965(nuclear membrane);GO:0005635(nuclear envelope);GO:0017056(structural constituent of nuclear pore);GO:0006260(DNA replication);GO:0051292(nuclear pore complex assembly);GO:0044615(nuclear pore nuclear basket);GO:0034399(nuclear periphery);GO:0042405(nuclear
circRNA174	GO:0005515(protein binding)
circRNA175	GO:0005488(binding);GO:0016020(membrane);GO:0005515(protein binding);GO:0044822(poly(A) RNA binding);GO:0005634(nucleus);GO:0017134(fibroblast growth factor binding);GO:0005681(spliceosomal complex);GO:2000270(negative regulation of fibroblast apoptotic process)
circRNA176	GO:0005515(protein binding);GO:0005737(cytoplasm);GO:0006914(autophagy);GO:0008285(negative regulation of cell proliferation);GO:0005930(axoneme);GO:0043524(negative regulation of neuron apoptotic process);GO:0021915(neural tube development);GO:0045335(phagocytic vesicle)

circRNA177	GO:0005515(protein binding);GO:0005488(binding);GO:0005813(centrosome);GO:0016020(membrane);GO:0000922(spindle pole);GO:0000930(gamma-tubulin complex);GO:0007051(spindle organization);GO:0051297(centrosome organization);GO:0043234(protein complex);GO:0035371(microtubule plus-end);GO:0030951(establishment or maintenance of microtubule cytoskeleton polarity)
circRNA178	GO:0045454(cell redox homeostasis)
circRNA179	GO:0005515(protein binding)
circRNA18	GO:0003677(DNA binding);GO:0003700(sequence-specific DNA binding transcription factor activity);GO:0005634(nucleus);GO:0006355("regulation of transcription, DNA-templated");GO:0043565(sequence-specific DNA binding);GO:0005515(protein binding);GO:0005667(transcription factor complex);GO:0045944(positive regulation of transcription from RNA polymerase
circRNA180	GO:0006974(cellular response to DNA damage stimulus);GO:0019005(SCF ubiquitin ligase complex);GO:0030332(cyclin binding);GO:0031146(SCF-dependent proteasomal ubiquitin-dependent protein catabolic process);GO:0031571(mitotic G1 DNA damage checkpoint);GO:0008054(cyclin catabolic process)
circRNA181	GO:0015031(protein transport);GO:0030904(retromer complex);GO:0042147("retrograde transport, endosome to Golgi");GO:0070062(extracellular vesicular exosome);GO:0005829(cytosol);GO:0005515(protein binding);GO:0005768(endosome);GO:0005765(lysosomal membrane)
circRNA182	GO:0003824(catalytic activity);GO:0004553("hydrolase activity, hydrolyzing O-glycosyl compounds");GO:0005516(calmodulin binding);GO:0005976(polysaccharide metabolic process);GO:0005977(glycogen metabolic process);GO:0005515(protein binding)
circRNA183	GO:0016607(nuclear speck);GO:0035516(oxidative DNA demethylase activity);GO:0035552(oxidative single-stranded DNA demethylation);GO:0035553(oxidative single-stranded RNA demethylation);GO:0035515(oxidative RNA demethylase activity);GO:0043734(DNA-N1-methyladenine dioxygenase activity);GO:0042245(RNA repair);GO:0006307(DNA dealkylation involved in DNA repair);GO:0008198(ferrous iron binding);GO:0070989(oxidative demethylation);GO:0080111(DNA demethylation);GO:0005634(nucleus);GO:0060612(adipose tissue development);GO:0044065(regulation of respiratory system process);GO:0040014(regulation of multicellular organism growth);GO:0001659(temperature homeostasis);GO:0010883(regulation of lipid storage);GO:0070350(regulation of white fat cell proliferation)
circRNA184	GO:0008270(zinc ion binding);GO:0046872(metal ion binding);GO:0005515(protein binding);GO:0030176(integral component of endoplasmic reticulum membrane);GO:0048471(perinuclear region of cytoplasm);GO:0005789(endoplasmic reticulum membrane);GO:0016020(membrane);GO:0006511(ubiquitin-dependent protein catabolic process);GO:0004842(ubiquitin-protein transferase activity);GO:0004872(receptor activity);GO:0000209(protein polyubiquitination);GO:0030968(endoplasmic reticulum unfolded protein response);GO:0051259(protein oligomerization);GO:0030433(ER-associated ubiquitin-dependent protein catabolic process);GO:0043234(protein complex);GO:0000836(Hrd1p ubiquitin ligase complex)
circRNA185	GO:0016491(oxidoreductase activity);GO:0055114(oxidation-reduction process);GO:0030198(extracellular matrix organization);GO:0031012(extracellular matrix)
circRNA186	GO:0005488(binding)
circRNA187	GO:0007165(signal transduction);GO:0005515(protein binding);GO:0043231(intracellular membrane-bounded organelle);GO:0005737(cytoplasm);GO:0005634(nucleus)
circRNA188	GO:0000166(nucleotide binding);GO:0003824(catalytic activity);GO:0005524(ATP binding);GO:0006464(cellular protein modification process);GO:0008641(small protein activating enzyme activity);GO:0016874(ligase activity);GO:0005515(protein binding);GO:0046982(protein heterodimerization activity);GO:0019948(SUMO activating enzyme activity);GO:0008134(transcription factor
circRNA189	GO:0003824(catalytic activity);GO:0004252(serine-type endopeptidase activity);GO:0006508(proteolysis);GO:0016020(membrane);GO:0070008(serine-type exopeptidase activity);GO:0005789(endoplasmic reticulum membrane);GO:0070062(extracellular vesicular exosome);GO:0005887(integral component of plasma membrane);GO:0005515(protein binding);GO:0005886(plasma membrane);GO:0016021(integral component of membrane);GO:0009986(cell surface);GO:0043066(negative regulation of apoptotic process);GO:0031965(nuclear membrane);GO:0030307(positive regulation of cell growth);GO:0008236(serine-type peptidase activity);GO:0008360(regulation of cell shape);GO:0008233(peptidase activity);GO:2000347(positive regulation of hepatocyte proliferation);GO:0034769(basement membrane disassembly);GO:0043923(positive regulation by host of viral transcription);GO:0010756(positive regulation of plasminogen activation);GO:0010719(negative regulation of epithelial to mesenchymal transition);GO:0050680(negative regulation of epithelial cell proliferation);GO:0007605(sensory perception of sound);GO:0015269(calcium-activated potassium channel activity);GO:0071805(potassium ion transmembrane transport);GO:0005911(cell-cell junction);GO:0043025(neuronal cell body);GO:0042632(cholesterol homeostasis);GO:0060429(epithelium development);GO:0010628(positive regulation of gene expression);GO:0090103(cochlea morphogenesis);GO:0050910(detection of mechanical stimulus involved in sensory perception of sound);GO:0010693(negative regulation of alkaline phosphatase activity);GO:2000611(positive regulation of thyroid hormone generation);GO:0097066(response to thyroid hormone);GO:0097195(pilomotor reflex)
circRNA19	GO:0005515(protein binding);GO:0030054(cell junction);GO:0005737(cytoplasm);GO:0005911(cell-cell junction);GO:0000226(microtubule cytoskeleton organization);GO:0005819(spindle);GO:0045177(apical part of cell);GO:0005938(cell cortex);GO:0043234(protein complex);GO:0051642(centrosome localization);GO:0005912(adherens junction);GO:0005913(cell-cell adherens junction);GO:0003383(apical constriction);GO:0005080(protein kinase C binding);GO:0032970(regulation of actin filament-based process);GO:0044319("wound healing, spreading of cells")
circRNA190	GO:0005509(calcium ion binding);GO:0070062(extracellular vesicular exosome);GO:0016020(membrane);GO:0005829(cytosol);GO:0005515(protein binding);GO:0004198(calcium-dependent cysteine-type
circRNA191	GO:0005216(ion channel activity);GO:0005219(ryanodine-sensitive calcium-release channel activity);GO:0005262(calcium channel activity);GO:0005509(calcium ion binding);GO:0006810(transport);GO:0006811(ion transport);GO:0006874(cellular calcium ion homeostasis);GO:0016020(membrane);GO:0016021(integral component of membrane);GO:0055085(transmembrane transport);GO:0070588(calcium ion transmembrane transport);GO:0005515(protein binding);GO:0070062(extracellular vesicular exosome);GO:0005737(cytoplasm);GO:0005886(plasma membrane);GO:0005938(cell cortex);GO:0001666(response to hypoxia);GO:0031674(I band);GO:0043588(skin development);GO:0005245(voltage-gated calcium channel activity);GO:0006816(calcium ion transport);GO:0016529(sarcoplasmic reticulum);GO:0030315(T-tubule);GO:0006936(muscle contraction);GO:0043234(protein complex);GO:0030314(junctional membrane complex);GO:0033017(sarcoplasmic reticulum membrane);GO:0071313(cellular response to caffeine);GO:0019899(enzyme binding);GO:0048741(skeletal muscle fiber development);GO:0002020(protease binding);GO:0003151(outflow tract morphogenesis);GO:0005790(smooth endoplasmic reticulum);GO:0043931(ossification involved in bone maturation);GO:0014808(release of sequestered calcium ion into cytosol by sarcoplasmic reticulum);GO:0051209(release of sequestered calcium ion into cytosol)
circRNA192	GO:0005216(ion channel activity);GO:0005219(ryanodine-sensitive calcium-release channel activity);GO:0005262(calcium channel activity);GO:0005509(calcium ion binding);GO:0006810(transport);GO:0006811(ion transport);GO:0006874(cellular calcium ion homeostasis);GO:0016020(membrane);GO:0016021(integral component of membrane);GO:0055085(transmembrane transport);GO:0070588(calcium ion transmembrane transport);GO:0005515(protein binding);GO:0070062(extracellular vesicular exosome);GO:0005737(cytoplasm);GO:0005886(plasma membrane);GO:0005938(cell cortex);GO:0001666(response to hypoxia);GO:0031674(I band);GO:0043588(skin development);GO:0005245(voltage-gated calcium channel activity);GO:0006816(calcium ion transport);GO:0016529(sarcoplasmic reticulum);GO:0030315(T-tubule);GO:0006936(muscle contraction);GO:0043234(protein complex);GO:0030314(junctional membrane complex);GO:0033017(sarcoplasmic reticulum membrane);GO:0071313(cellular response to caffeine);GO:0019899(enzyme binding);GO:0048741(skeletal muscle fiber development);GO:0002020(protease binding);GO:0003151(outflow tract morphogenesis);GO:0005790(smooth endoplasmic reticulum);GO:0043931(ossification involved in bone maturation);GO:0014808(release of sequestered calcium ion into cytosol by sarcoplasmic reticulum);GO:0051209(release of sequestered calcium ion into cytosol)
circRNA193	GO:0000166(nucleotide binding);GO:0003824(catalytic activity);GO:0005524(ATP binding);GO:0016301(kinase activity);GO:0016310(phosphorylation);GO:0016740(transferase activity);GO:0016772("transferase activity, transferring phosphorus-containing groups");GO:0004111(creatine kinase activity);GO:0046314(phosphocreatine biosynthetic process)
circRNA194	GO:0003824(catalytic activity);GO:0006464(cellular protein modification process);GO:0008641(small protein activating enzyme activity);GO:0043231(intracellular membrane-bounded organelle);GO:0005515(protein binding);GO:0005634(nucleus);GO:0008022(protein C-terminus binding);GO:0019948(SUMO activating enzyme activity);GO:0043008(ATP-dependent protein binding)
circRNA195	GO:0000166(nucleotide binding);GO:0003676(nucleic acid binding);GO:0004386(helicase activity);GO:0005524(ATP binding);GO:0008152(metabolic process);GO:0016787(hydrolase activity);GO:0016020(membrane);GO:0044822(poly(A) RNA binding);GO:2000623("negative regulation of nuclear-transcribed mRNA catabolic process, nonsense-mediated decay");GO:0000956(nuclear-transcribed mRNA catabolic process)
circRNA196	GO:0005929(cilium);GO:0036157(outer dynein arm);GO:0036158(outer dynein arm assembly)
circRNA197	GO:0005509(calcium ion binding);GO:0005615(extracellular space);GO:0070062(extracellular vesicular exosome);GO:0016020(membrane);GO:0005515(protein binding);GO:0005793(endoplasmic reticulum-Golgi intermediate compartment)
circRNA198	GO:0004373(glycogen (starch) synthase activity);GO:0005978(glycogen biosynthetic process);GO:0016020(membrane);GO:0005515(protein binding);GO:0019901(protein kinase binding);GO:0061547("glycogen synthase activity, transferring glucose-1-phosphate");GO:0005737(cytoplasm);GO:0007507(heart development);GO:0016234(inclusion body)
circRNA199	GO:0000166(nucleotide binding);GO:0003774(motor activity);GO:0005524(ATP binding);GO:0008152(metabolic process);GO:0016459(myosin complex);GO:0005515(protein binding);GO:0070062(extracellular vesicular exosome);GO:0016020(membrane);GO:0006200(ATP catabolic process);GO:0003009(skeletal muscle contraction);GO:0051015(actin filament binding);GO:0070584(mitochondrion morphogenesis);GO:0031032(actomyosin structure organization);GO:0007605(sensory perception of sound);GO:0071625(vocalization behavior);GO:0097513(myosin II filament);GO:0042641(actomyosin);GO:0016460(myosin II complex);GO:0030898(actin-dependent ATPase activity);GO:0019228(neuronal action potential);GO:0014732(skeletal muscle atrophy);GO:0007519(skeletal muscle tissue development);GO:0005737(cytoplasm);GO:0030424(axon);GO:0030426(growth cone);GO:0001725(stress fiber);GO:0000146(microfilament motor activity);GO:0030048(actin filament-based movement)
circRNA2	GO:0005216(ion channel activity);GO:0005247(voltage-gated chloride channel activity);GO:0006821(chloride transport);GO:0016020(membrane);GO:0016021(integral component of membrane);GO:0030554(adenyl nucleotide binding);GO:0034220(ion transmembrane transport);GO:0044070(regulation of anion transport);GO:0055085(transmembrane transport);GO:1902476(chloride
circRNA20	GO:0005515(protein binding);GO:0030054(cell junction);GO:0005737(cytoplasm);GO:0005911(cell-cell junction);GO:0000226(microtubule cytoskeleton organization);GO:0005819(spindle);GO:0045177(apical part of cell);GO:0005938(cell cortex);GO:0043234(protein complex);GO:0051642(centrosome localization);GO:0005912(adherens junction);GO:0005913(cell-cell adherens junction);GO:0003383(apical constriction);GO:0005080(protein kinase C binding);GO:0032970(regulation of actin filament-based process);GO:0044319("wound healing, spreading of cells")
circRNA200	GO:0005515(protein binding);GO:0009055(electron carrier activity)
circRNA201	GO:0003676(nucleic acid binding);GO:0005622(intracellular);GO:0006355("regulation of transcription, DNA-templated");GO:0046872(metal ion binding)
circRNA202	GO:0004672(protein kinase activity);GO:0004674(protein serine/threonine kinase activity);GO:0005524(ATP binding);GO:0006468(protein phosphorylation);GO:0016772("transferase activity, transferring phosphorus-containing groups");GO:0035556(intracellular signal transduction);GO:0004713(protein tyrosine kinase activity)
circRNA203	GO:0000042(protein targeting to Golgi);GO:0005515(protein binding);GO:0043231(intracellular membrane-bounded organelle);GO:0005737(cytoplasm);GO:0005634(nucleus);GO:0005794(Golgi apparatus);GO:0043001(Golgi to plasma membrane protein transport);GO:0051020(GTPase binding)
circRNA204	GO:0003676(nucleic acid binding);GO:0005622(intracellular);GO:0006355("regulation of transcription, DNA-templated");GO:0046872(metal ion binding)
circRNA205	GO:0016020(membrane);GO:0016021(integral component of membrane);GO:0005622(intracellular);GO:1902476(chloride transmembrane transport);GO:0006821(chloride transport);GO:0005229(intracellular calcium activated chloride channel activity);GO:0005886(plasma membrane);GO:0005227(calcium activated cation channel activity);GO:0006812(cation transport)
circRNA206	GO:0005739(mitochondrion)

circRNA207	GO:0004571("mannosyl-oligosaccharide 1,2-alpha-mannosidase activity");GO:0005509(calcium ion binding);GO:0008152(metabolic process);GO:0016020(membrane);GO:0005515(protein binding);GO:0051787(misfolded protein binding);GO:0030433(ER-associated ubiquitin-dependent protein catabolic process);GO:0030176(integral component of endoplasmic reticulum membrane)
circRNA208	GO:0005216(ion channel activity);GO:0005220("inositol 1,4,5-trisphosphate-sensitive calcium-release channel activity");GO:0005262(calcium channel activity);GO:0005783(endoplasmic reticulum);GO:0006810(transport);GO:0006811(ion transport);GO:0006816(calcium ion transport);GO:0016020(membrane);GO:0016021(integral component of membrane);GO:0048016(inositol phosphate-mediated signaling);GO:0055085(transmembrane transport);GO:0070588(calcium ion transmembrane transport);GO:0005515(protein binding);GO:0001666(response to hypoxia);GO:0031094(platelet dense tubular network);GO:0031088(platelet dense granule membrane);GO:0005737(cytoplasm);GO:0005789(endoplasmic reticulum membrane);GO:0005730(nucleolus);GO:0016529(sarcoplasmic reticulum);GO:0035091(phosphatidylinositol binding);GO:0009791(post-embryonic development);GO:0005637(nuclear inner membrane);GO:0043234(protein complex);GO:0014069(postsynaptic density);GO:0005635(nuclear envelope);GO:0070059(intrinsic apoptotic signaling pathway in response to endoplasmic reticulum stress);GO:0032469(endoplasmic reticulum calcium ion homeostasis);GO:0051209(release of sequestered calcium ion into cytosol);GO:0005218(intracellular ligand-gated calcium channel activity);GO:0050882(voluntary musculoskeletal movement);GO:0005955(calcineurin complex)
circRNA209	GO:0005216(ion channel activity);GO:0005220("inositol 1,4,5-trisphosphate-sensitive calcium-release channel activity");GO:0005262(calcium channel activity);GO:0005783(endoplasmic reticulum);GO:0006810(transport);GO:0006811(ion transport);GO:0006816(calcium ion transport);GO:0016020(membrane);GO:0016021(integral component of membrane);GO:0048016(inositol phosphate-mediated signaling);GO:0055085(transmembrane transport);GO:0070588(calcium ion transmembrane transport);GO:0005515(protein binding);GO:0001666(response to hypoxia);GO:0031094(platelet dense tubular network);GO:0031088(platelet dense granule membrane);GO:0005737(cytoplasm);GO:0005789(endoplasmic reticulum membrane);GO:0005730(nucleolus);GO:0016529(sarcoplasmic reticulum);GO:0035091(phosphatidylinositol binding);GO:0009791(post-embryonic development);GO:0005637(nuclear inner membrane);GO:0043234(protein complex);GO:0014069(postsynaptic density);GO:0005635(nuclear envelope);GO:0070059(intrinsic apoptotic signaling pathway in response to endoplasmic reticulum stress);GO:0032469(endoplasmic reticulum calcium ion homeostasis);GO:0051209(release of sequestered calcium ion into cytosol);GO:0005218(intracellular ligand-gated calcium channel activity);GO:0050882(voluntary musculoskeletal movement);GO:0005955(calcineurin complex)
circRNA21	GO:0048706(embryonic skeletal system development);GO:0005737(cytoplasm)
circRNA210	GO:0003824(catalytic activity);GO:0005524(ATP binding);GO:0008152(metabolic process);GO:0016874(ligase activity);GO:0005739(mitochondrion);GO:0005886(plasma membrane)
circRNA211	GO:0005515(protein binding);GO:0007605(sensory perception of sound);GO:0060384(innervation);GO:0032474(otolith morphogenesis)
circRNA212	GO:0006810(transport);GO:0016020(membrane);GO:0016021(integral component of membrane);GO:0055085(transmembrane transport);GO:0005739(mitochondrion)
circRNA213	GO:0006810(transport);GO:0016020(membrane);GO:0016021(integral component of membrane);GO:0055085(transmembrane transport);GO:0005739(mitochondrion)
circRNA214	GO:0006457(protein folding);GO:0016272(prefoldin complex);GO:0051082(unfolded protein binding);GO:0005515(protein binding);GO:0016021(integral component of membrane)
circRNA215	GO:0000166(nucleotide binding);GO:0005525(GTP binding);GO:0005622(intracellular);GO:0007264(small GTPase mediated signal transduction);GO:0003924(GTPase activity);GO:0004871(signal transducer activity);GO:0019001(guanyl nucleotide binding);GO:0031683(G-protein beta/gamma-subunit complex binding);GO:0007186(G-protein coupled receptor signaling pathway);GO:0015031(protein transport);GO:0006886(intracellular protein transport);GO:0005634(nucleus);GO:0005737(cytoplasm);GO:0070062(extracellular vesicular exosome);GO:0016020(membrane);GO:0005829(cytosol);GO:0045944(positive regulation of transcription from RNA polymerase II promoter);GO:0005515(protein binding);GO:0043066(negative regulation of apoptotic process);GO:0006471(protein ADP-ribosylation);GO:0005154(epidermal growth factor receptor binding);GO:0016477(cell migration);GO:0031584(activation of phospholipase D activity);GO:2000377(regulation of reactive oxygen species metabolic process);GO:0032587(ruffle membrane);GO:0005886(plasma membrane)
circRNA216	GO:0007165(signal transduction);GO:0005634(nucleus);GO:0005794(Golgi apparatus)
circRNA217	GO:0005319(lipid transporter activity);GO:0006869(lipid transport);GO:0016021(integral component of membrane)
circRNA218	GO:0005634(nucleus);GO:0006355("regulation of transcription, DNA-templated");GO:0005515(protein binding);GO:0045892("negative regulation of transcription, DNA-templated");GO:0042393(histone binding);GO:0048635(negative regulation of muscle organ development)
circRNA219	GO:0004672(protein kinase activity);GO:0004674(protein serine/threonine kinase activity);GO:0005524(ATP binding);GO:0006468(protein phosphorylation);GO:0016772("transferase activity, transferring phosphorus-containing groups");GO:0004713(protein tyrosine kinase activity)
circRNA22	GO:0005509(calcium ion binding);GO:0005515(protein binding)
circRNA220	GO:0008152(metabolic process);GO:0016757("transferase activity, transferring glycosyl groups");GO:0016020(membrane)
circRNA221	GO:0003682(chromatin binding);GO:0005515(protein binding);GO:0008285(negative regulation of cell proliferation);GO:0007507(heart development);GO:0001890(placenta development);GO:0000776(kine
circRNA222	GO:0004843(ubiquitin-specific protease activity);GO:0006508(proteolysis);GO:0006511(ubiquitin-dependent protein catabolic process);GO:0008233(peptidase activity);GO:0008234(cysteine-type peptidase activity);GO:0016579(protein deubiquitination);GO:0016787(hydrolase activity);GO:0036459(ubiquitinyl hydrolase activity);GO:0005737(cytoplasm);GO:0005515(protein binding);GO:0005886(plasma membrane);GO:0005634(nucleus);GO:0004221(ubiquitin thiolesterase activity);GO:0042802(identical protein binding);GO:0031685(adenosine receptor binding);GO:0034394(protein localization to cell surface);GO:0031397(negative regulation of protein ubiquitination);GO:0031647(regulation of protein stability)
circRNA223	GO:0006810(transport);GO:0016021(integral component of membrane);GO:0046872(metal ion binding);GO:0016020(membrane);GO:0005515(protein binding);GO:0005764(lysosome);GO:0005776(autophagic vacuole);GO:0072383(plus-end-directed vesicle transport along microtubule);GO:0005770(late endosome)
circRNA224	GO:0005515(protein binding);GO:0042802(identical protein binding);GO:0034464(BBSome);GO:0005737(cytoplasm);GO:0072594(establishment of protein localization to organelle)
circRNA225	GO:0008152(metabolic process);GO:0042578(phosphoric ester hydrolase activity);GO:0005515(protein binding);GO:0005794(Golgi apparatus);GO:0032281(alpha-amino-3-hydroxy-5-methyl-4-isoxazolepropionic acid selective glutamate receptor complex)
circRNA226	GO:0006355("regulation of transcription, DNA-templated");GO:0008270(zinc ion binding);GO:0045892("negative regulation of transcription, DNA-templated");GO:0046872(metal ion binding);GO:0000932(cytoplasmic mRNA processing body);GO:0005737(cytoplasm);GO:0005515(protein binding);GO:0005634(nucleus);GO:0016310(phosphorylation);GO:2000637(positive regulation of gene silencing by miRNA);GO:0035331(negative regulation of hippo signaling);GO:0035195(gene silencing by miRNA);GO:0007010(cytoskeleton organization);GO:0005912(adherens junction);GO:0001666(response to hypoxia);GO:0005925(focal adhesion);GO:0016442(RISC complex);GO:0016477(cell migration);GO:0008360(regulation of cell shape);GO:0033962(cytoplasmic mRNA processing body assembly);GO:0090090(negative regulation of canonical Wnt signaling pathway);GO:0003714(transcription corepressor activity);GO:0045668(negative regulation of osteoblast differentiation);GO:0002076(osteoblast development)
circRNA227	GO:0008152(metabolic process);GO:0008270(zinc ion binding);GO:0016020(membrane);GO:0016021(integral component of membrane);GO:0016740(transferase activity);GO:0016746("transferase activity, transferring acyl groups");GO:0019706(protein-cysteine S-palmitoyltransferase activity);GO:0046872(metal ion binding);GO:0005794(Golgi apparatus);GO:0016409(palmitoyltransferase activity);GO:0018345(protein palmitoylation);GO:0006605(protein targeting)
circRNA228	GO:0008152(metabolic process);GO:0008270(zinc ion binding);GO:0016020(membrane);GO:0016021(integral component of membrane);GO:0016740(transferase activity);GO:0016746("transferase activity, transferring acyl groups");GO:0019706(protein-cysteine S-palmitoyltransferase activity);GO:0046872(metal ion binding);GO:0005794(Golgi apparatus);GO:0016409(palmitoyltransferase activity);GO:0018345(protein palmitoylation);GO:0006605(protein targeting)
circRNA229	()
circRNA23	()
circRNA230	GO:0003677(DNA binding);GO:0003700(sequence-specific DNA binding transcription factor activity);GO:0003707(steroid hormone receptor activity);GO:0005634(nucleus);GO:0006351("transcription, DNA-templated");GO:0006355("regulation of transcription, DNA-templated");GO:0008270(zinc ion binding);GO:0043401(steroid hormone mediated signaling pathway);GO:0043565(sequence-specific DNA binding);GO:0046872(metal ion binding);GO:0005515(protein binding);GO:0007283(spermatogenesis);GO:0021549(cerebellum development);GO:0040019(positive regulation of embryonic development);GO:0048520(positive regulation of behavior);GO:0051321(meiotic cell cycle)
circRNA231	GO:0005089(Rho guanyl-nucleotide exchange factor activity);GO:0032321(positive regulation of Rho GTPase activity);GO:0035023(regulation of Rho protein signal transduction);GO:0046872(metal ion binding);GO:0005515(protein binding)
circRNA232	GO:0005515(protein binding)
circRNA233	GO:0000166(nucleotide binding);GO:0003924(GTPase activity);GO:0005525(GTP binding);GO:0006184(GTP catabolic process);GO:0005515(protein binding);GO:0030529(ribonucleoprotein complex);GO:0005739(mitochondrion);GO:0000049(tRNA binding);GO:0003746(translation elongation factor activity);GO:0043021(ribonucleoprotein complex binding);GO:0035368(selenocysteine
circRNA234	GO:0016567(protein ubiquitination);GO:0035556(intracellular signal transduction);GO:0005515(protein binding)
circRNA235	GO:0003677(DNA binding);GO:0005634(nucleus);GO:0006351("transcription, DNA-templated");GO:0006355("regulation of transcription, DNA-templated");GO:0046983(protein dimerization activity);GO:0007507(heart development);GO:0006366(transcription from RNA polymerase II promoter);GO:0005737(cytoplasm);GO:0045944(positive regulation of transcription from RNA polymerase II promoter);GO:0005515(protein binding);GO:0003700(sequence-specific DNA binding transcription factor activity);GO:0000978(RNA polymerase II core promoter proximal region sequence-specific DNA binding);GO:0000122(negative regulation of transcription from RNA polymerase II promoter);GO:0000981(sequence-specific DNA binding RNA polymerase II transcription factor activity);GO:0000165(MAPK cascade);GO:0003682(chromatin binding);GO:0043565(sequence-specific DNA binding);GO:0046982(protein heterodimerization activity);GO:0000977(RNA polymerase II regulatory region sequence-specific DNA binding);GO:0001077(RNA polymerase II core promoter proximal region sequence-specific DNA binding transcription factor activity involved in positive regulation of transcription);GO:0001085(RNA polymerase II transcription factor binding);GO:0046332(SMAD binding);GO:0070375(ERK5 cascade);GO:0035035(histone acetyltransferase binding);GO:0033613(activating transcription factor binding);GO:0071277(cellular response to calcium ion);GO:0042826(histone deacetylase binding);GO:0001105(RNA polymerase II transcription coactivator activity);GO:0000790(nuclear chromatin);GO:0019901(protein kinase binding);GO:0045893("positive regulation of transcription, DNA-templated");GO:0003705(RNA polymerase II distal enhancer sequence-specific DNA binding transcription factor activity);GO:0048311(mitochondrion distribution);GO:0061337(cardiac conduction);GO:0000002(mitochondrial genome maintenance);GO:0055005(ventricular cardiac myofibril assembly)

circRNA236	GO:0005515(protein binding)
circRNA237	GO:0003676(nucleic acid binding);GO:0003677(DNA binding);GO:0004386(helicase activity);GO:0005524(ATP binding);GO:0005634(nucleus);GO:0008152(metabolic process);GO:0005515(protein binding);GO:0007517(muscle organ development);GO:0060218(hematopoietic stem cell differentiation);GO:0042393(histone binding);GO:0006974(cellular response to DNA damage
circRNA238	GO:0003676(nucleic acid binding);GO:0003677(DNA binding);GO:0004386(helicase activity);GO:0005524(ATP binding);GO:0005634(nucleus);GO:0008152(metabolic process);GO:0005515(protein binding);GO:0007517(muscle organ development);GO:0060218(hematopoietic stem cell differentiation);GO:0042393(histone binding);GO:0006974(cellular response to DNA damage
circRNA239	GO:0004181(metallocarboxypeptidase activity);GO:0006508(proteolysis);GO:0008270(zinc ion binding);GO:0005488(binding);GO:0005515(protein binding);GO:0015631(tubulin binding);GO:0035609(C-terminal protein deglutamylation);GO:0035610(protein side chain deglutamylation)
circRNA24	GO:0003779(actin binding);GO:0007010(cytoskeleton organization);GO:0005737(cytoplasm);GO:0005515(protein binding);GO:0005886(plasma membrane);GO:0005634(nucleus);GO:0051015(actin filament binding);GO:0015629(actin cytoskeleton);GO:0007519(skeletal muscle tissue development);GO:0043034(costamere)
circRNA240	GO:0004181(metallocarboxypeptidase activity);GO:0006508(proteolysis);GO:0008270(zinc ion binding);GO:0005488(binding);GO:0005515(protein binding);GO:0015631(tubulin binding);GO:0035609(C-terminal protein deglutamylation);GO:0035610(protein side chain deglutamylation)
circRNA241	GO:0009611(response to wounding);GO:0030336(negative regulation of cell migration)
circRNA242	GO:0006810(transport);GO:0006886(intracellular protein transport);GO:0008565(protein transporter activity);GO:0015031(protein transport);GO:0016192(vesicle-mediated transport);GO:0030117(membrane coat);GO:0030027(lamellipodium);GO:0016020(membrane)
circRNA243	GO:0004450(isocitrate dehydrogenase (NADP+) activity);GO:0006102(isocitrate metabolic process);GO:0055114(oxidation-reduction process);GO:0016616("oxidoreductase activity, acting on the CH-OH group of donors, NAD or NADP as acceptor");GO:0005739(mitochondrion);GO:0005743(mitochondrial inner membrane)
circRNA244	GO:0004450(isocitrate dehydrogenase (NADP+) activity);GO:0006102(isocitrate metabolic process);GO:0055114(oxidation-reduction process);GO:0016616("oxidoreductase activity, acting on the CH-OH group of donors, NAD or NADP as acceptor");GO:0005739(mitochondrion);GO:0005743(mitochondrial inner membrane)
circRNA245	GO:0000156(phosphorelay response regulator activity);GO:0000160(phosphorelay signal transduction system);GO:0003824(catalytic activity);GO:0004114("3',5'-cyclic-nucleotide phosphodiesterase activity");GO:0004871(signal transducer activity);GO:0007165(signal transduction);GO:0008081(phosphoric diester hydrolase activity);GO:0008152(metabolic process);GO:0016787(hydrolase activity);GO:0035556(intracellular signal transduction);GO:0046872(metal ion binding);GO:0006355("regulation of transcription, DNA-templated");GO:0070062(extracellular vesicular exosome);GO:0004115("3',5'-cyclic-AMP phosphodiesterase activity")
circRNA246	GO:0005515(protein binding)
circRNA247	GO:0016020(membrane);GO:0016021(integral component of membrane);GO:0070062(extracellular vesicular exosome)
circRNA248	GO:0016020(membrane);GO:0016021(integral component of membrane);GO:0070062(extracellular vesicular exosome)
circRNA249	GO:0016020(membrane);GO:0016021(integral component of membrane);GO:0016192(vesicle-mediated transport)
circRNA25	GO:0008080(N-acetyltransferase activity);GO:0008152(metabolic process);GO:0005515(protein binding);GO:0005634(nucleus);GO:0004402(histone acetyltransferase activity);GO:0005671(Ada2/Gcn5/Ada3 transcription activator complex);GO:0043966(histone H3 acetylation);GO:0016573(histone acetylation);GO:0000086(G2/M transition of mitotic cell cycle)
circRNA250	GO:0004842(ubiquitin-protein transferase activity);GO:0008152(metabolic process);GO:0016567(protein ubiquitination);GO:0016874(ligase activity);GO:0046872(metal ion binding);GO:0005515(protein binding);GO:0005488(binding);GO:0001843(neural tube closure)
circRNA251	GO:0005488(binding)
circRNA252	GO:0005515(protein binding);GO:0016529(sarcoplasmic reticulum);GO:0005635(nuclear envelope);GO:1902261(positive regulation of delayed rectifier potassium channel activity);GO:1901381(positive regulation of potassium ion transmembrane transport);GO:0071320(cellular response to cAMP);GO:0044325(ion channel binding);GO:0060306(regulation of membrane
circRNA253	GO:0005096(GTPase activator activity);GO:0043547(positive regulation of GTPase activity);GO:0051056(regulation of small GTPase mediated signal transduction);GO:0046982(protein heterodimerization activity);GO:0005737(cytoplasm);GO:0005634(nucleus);GO:0006355("regulation of transcription, DNA-templated")
circRNA254	GO:0005515(protein binding)
circRNA255	GO:0000287(magnesium ion binding);GO:0005524(ATP binding);GO:0005622(intracellular);GO:0016310(phosphorylation);GO:0032957(inositol trisphosphate metabolic process);GO:0046872(metal ion binding);GO:0047325(inositol tetrakisphosphate 1-kinase activity);GO:0052725("inositol-1,3,4-trisphosphate 6-kinase activity");GO:0052726("inositol-1,3,4-trisphosphate 5-kinase activity")
circRNA256	GO:0000166(nucleotide binding);GO:0004672(protein kinase activity);GO:0004674(protein serine/threonine kinase activity);GO:0005524(ATP binding);GO:0006468(protein phosphorylation);GO:0016772("transferase activity, transferring phosphorus-containing groups");GO:0005730(nucleolus);GO:0005737(cytoplasm);GO:0005515(protein binding);GO:0005634(nucleus);GO:0005795(Golgi stack);GO:0046777(protein autophosphorylation);GO:0090166(Golgi disassembly);GO:0072355(histone H3-T3 phosphorylation);GO:0035175(histone kinase activity (H3-S10 specific));GO:0031493(nucleosomal histone binding);GO:0072354(histone kinase activity (H3-T3 specific));GO:0043987(histone H3-S10 phosphorylation)
circRNA257	GO:0000166(nucleotide binding);GO:0004672(protein kinase activity);GO:0004674(protein serine/threonine kinase activity);GO:0005524(ATP binding);GO:0006468(protein phosphorylation);GO:0016301(kinase activity);GO:0016310(phosphorylation);GO:0016740(transferase activity);GO:0016772("transferase activity, transferring phosphorus-containing groups");GO:0004713(protein tyrosine kinase activity);GO:0005515(protein binding);GO:0005886(plasma membrane)
circRNA258	GO:0000166(nucleotide binding);GO:0004672(protein kinase activity);GO:0004674(protein serine/threonine kinase activity);GO:0005524(ATP binding);GO:0006468(protein phosphorylation);GO:0016301(kinase activity);GO:0016310(phosphorylation);GO:0016740(transferase activity);GO:0016772("transferase activity, transferring phosphorus-containing groups");GO:0004713(protein tyrosine kinase activity);GO:0005515(protein binding);GO:0005886(plasma membrane)
circRNA259	GO:0006915(apoptotic process);GO:0008134(transcription factor binding);GO:0045786(negative regulation of cell cycle);GO:0005515(protein binding);GO:0005737(cytoplasm);GO:0005886(plasma membrane);GO:0005634(nucleus);GO:0072332(intrinsic apoptotic signaling pathway by p53 class mediator)
circRNA26	()
circRNA260	GO:0005634(nucleus);GO:0006352("DNA-templated transcription, initiation");GO:0006355("regulation of transcription, DNA-templated");GO:0008270(zinc ion binding);GO:0017025(TBP-class protein binding);GO:0045893("positive regulation of transcription, DNA-templated");GO:0043488(regulation of mRNA stability)
circRNA261	GO:0005515(protein binding);GO:0005739(mitochondrion);GO:0006936(muscle contraction);GO:0031430(M band)
circRNA262	GO:0008083(growth factor activity);GO:0016020(membrane);GO:0051781(positive regulation of cell division);GO:0005515(protein binding);GO:0060754(positive regulation of mast cell chemotaxis);GO:0050930(induction of positive chemotaxis);GO:0042056(chemoattractant activity);GO:0050918(positive chemotaxis);GO:0005615(extracellular space);GO:0008285(negative regulation of cell proliferation);GO:0008284(positive regulation of cell proliferation);GO:0016331(morphogenesis of embryonic epithelium);GO:0001954(positive regulation of cell-matrix adhesion);GO:0009887(organ morphogenesis);GO:0050731(positive regulation of peptidyl-tyrosine phosphorylation);GO:0045766(positive regulation of angiogenesis);GO:0048010(vascular endothelial growth factor receptor signaling pathway);GO:1901492(positive regulation of lymphangiogenesis);GO:0043185(vascular endothelial growth factor receptor 3 binding)
circRNA263	GO:0008152(metabolic process);GO:0016787(hydrolase activity);GO:0005783(endoplasmic reticulum);GO:0070062(extracellular vesicular exosome);GO:0006517(protein deglycosylation);GO:0005764(lysosome);GO:0003948(N4-(beta-N-acetylglucosaminyl)-L-asparaginase activity);GO:0051604(protein maturation)
circRNA264	GO:0005509(calcium ion binding)
circRNA265	GO:0005622(intracellular);GO:0007165(signal transduction);GO:0008289(lipid binding);GO:0035023(regulation of Rho protein signal transduction);GO:0005515(protein binding);GO:0042169(SH2 domain binding);GO:0032321(positive regulation of Rho GTPase activity);GO:0006915(apoptotic process);GO:0005737(cytoplasm);GO:0005634(nucleus);GO:0008285(negative regulation of cell proliferation);GO:0005925(focal adhesion);GO:0005100(Rho GTPase activator activity);GO:0035307(positive regulation of protein dephosphorylation);GO:0006919(activation of cysteine-type endopeptidase activity involved in apoptotic process);GO:0005901(caveola);GO:0008360(regulation of cell shape);GO:0032956(regulation of actin cytoskeleton organization);GO:1900119(positive regulation of execution phase of apoptosis);GO:0035024(negative regulation of Rho protein signal transduction);GO:0051497(negative regulation of stress fiber assembly);GO:0030336(negative regulation of cell migration);GO:0030900(forebrain development);GO:0030036(actin cytoskeleton organization);GO:0001843(neural tube closure);GO:0003007(heart morphogenesis);GO:0048041(focal adhesion assembly);GO:0021575(hindbrain morphogenesis);()
circRNA266	GO:0005622(intracellular);GO:0007165(signal transduction);GO:0008289(lipid binding);GO:0035023(regulation of Rho protein signal transduction);GO:0005515(protein binding);GO:0042169(SH2 domain binding);GO:0032321(positive regulation of Rho GTPase activity);GO:0006915(apoptotic process);GO:0005737(cytoplasm);GO:0005634(nucleus);GO:0008285(negative regulation of cell proliferation);GO:0005925(focal adhesion);GO:0005100(Rho GTPase activator activity);GO:0035307(positive regulation of protein dephosphorylation);GO:0006919(activation of cysteine-type endopeptidase activity involved in apoptotic process);GO:0005901(caveola);GO:0008360(regulation of cell shape);GO:0032956(regulation of actin cytoskeleton organization);GO:1900119(positive regulation of execution phase of apoptosis);GO:0035024(negative regulation of Rho protein signal transduction);GO:0051497(negative regulation of stress fiber assembly);GO:0030336(negative regulation of cell migration);GO:0030900(forebrain development);GO:0030036(actin cytoskeleton organization);GO:0001843(neural tube closure);GO:0003007(heart morphogenesis);GO:0048041(focal adhesion assembly);GO:0021575(hindbrain morphogenesis);()
circRNA267	GO:0003676(nucleic acid binding);GO:0004527(exonuclease activity);GO:0090305(nucleic acid phosphodiester bond hydrolysis);GO:0008408(3'-5' exonuclease activity);GO:0005634(nucleus);GO:0043022(ribosome binding);GO:0019843(rRNA binding);GO:0071204(histone pre-mRNA 3'end processing complex);GO:0071207(histone pre-mRNA stem-loop
circRNA268	GO:0005673(transcription factor TFIIE complex);GO:0006367(transcription initiation from RNA polymerase II promoter);GO:0005737(cytoplasm);GO:0005515(protein binding);GO:0044822(poly(A) RNA binding);GO:0005634(nucleus)
circRNA269	GO:0000139(Golgi membrane);GO:0005794(Golgi apparatus);GO:0006486(protein glycosylation);GO:0008417(fucosyltransferase activity);GO:0016020(membrane);GO:0016021(integral component of membrane);GO:0016740(transferase activity);GO:0016757("transferase activity, transferring glycosyl groups");GO:0032580(Golgi cisterna membrane);GO:0036065(fucosylation);GO:0046920(alpha-(1-
circRNA27	GO:0016491(oxidoreductase activity);GO:0055114(oxidation-reduction process)
circRNA270	GO:0005515(protein binding);GO:0006974(cellular response to DNA damage stimulus);GO:0005634(nucleus);GO:0008284(positive regulation of cell proliferation);GO:0035097(histone methyltransferase complex);GO:0044212(transcription regulatory region DNA binding);GO:0048188(Set1C/COMPASS complex);GO:0042800(histone methyltransferase activity (H3-K4 specific));GO:0051568(histone H3-
circRNA271	GO:0005634(nucleus);GO:0008168(methyltransferase activity);GO:0008270(zinc ion binding);GO:0016740(transferase activity);GO:0018024(histone-lysine N-methyltransferase activity);GO:0032259(methylation);GO:0034968(histone lysine methylation);GO:0046872(metal ion binding);GO:0005515(protein binding);GO:0016571(histone methylation)

circRNA272	()
circRNA273	GO:0045111(intermediate filament cytoskeleton);GO:0005737(cytoplasm);GO:0005515(protein binding);GO:0015630(microtubule cytoskeleton);GO:0019904(protein domain specific binding);GO:0000226(microtubule cytoskeleton organization);GO:0008283(cell proliferation);GO:0022008(neurogenesis);GO:0021987(cerebral cortex development);GO:0022027(interkinetic nuclear
circRNA274	GO:0004222(metalloendopeptidase activity);GO:0006508(proteolysis);GO:0008237(metallopeptidase activity);GO:0008270(zinc ion binding);GO:0005515(protein binding);GO:0043236(laminin binding);GO:0005615(extracellular space);GO:0007160(cell-matrix adhesion);GO:0007155(cell adhesion);GO:0071222(cellular response to lipopolysaccharide);GO:0033630(positive regulation of cell adhesion mediated by integrin);GO:0050714(positive regulation of protein secretion);GO:0000186(activation of MAPKK activity);GO:0034612(response to tumor necrosis factor);GO:0005518(collagen binding);GO:0007179(transforming growth factor beta receptor signaling pathway);GO:0006509(membrane protein ectodomain proteolysis);GO:0017124(SH3 domain binding);GO:0034241(positive regulation of macrophage fusion);GO:0010042(response to manganese ion);GO:0033631(cell-cell adhesion mediated by integrin);GO:0033627(cell adhesion mediated by integrin);GO:0042542(response to hydrogen peroxide);GO:0051592(response to calcium ion);GO:0051549(positive regulation of keratinocyte migration);GO:0030216(keratinocyte differentiation);GO:0051088(PMA-inducible membrane protein ectodomain proteolysis);GO:0042117(monocyte activation);GO:0005886(plasma membrane);GO:0005737(cytoplasm);GO:0009986(cell surface);GO:0051384(response to glucocorticoid);GO:0005080(protein kinase C binding);GO:0051044(positive regulation of membrane protein ectodomain proteolysis);GO:0031233(intrinsic component of external side of plasma membrane)
circRNA275	GO:0004222(metalloendopeptidase activity);GO:0006508(proteolysis);GO:0008237(metallopeptidase activity);GO:0008270(zinc ion binding);GO:0005515(protein binding);GO:0043236(laminin binding);GO:0005615(extracellular space);GO:0007160(cell-matrix adhesion);GO:0007155(cell adhesion);GO:0071222(cellular response to lipopolysaccharide);GO:0033630(positive regulation of cell adhesion mediated by integrin);GO:0050714(positive regulation of protein secretion);GO:0000186(activation of MAPKK activity);GO:0034612(response to tumor necrosis factor);GO:0005518(collagen binding);GO:0007179(transforming growth factor beta receptor signaling pathway);GO:0006509(membrane protein ectodomain proteolysis);GO:0017124(SH3 domain binding);GO:0034241(positive regulation of macrophage fusion);GO:0010042(response to manganese ion);GO:0033631(cell-cell adhesion mediated by integrin);GO:0033627(cell adhesion mediated by integrin);GO:0042542(response to hydrogen peroxide);GO:0051592(response to calcium ion);GO:0051549(positive regulation of keratinocyte migration);GO:0030216(keratinocyte differentiation);GO:0051088(PMA-inducible membrane protein ectodomain proteolysis);GO:0042117(monocyte activation);GO:0005886(plasma membrane);GO:0005737(cytoplasm);GO:0009986(cell surface);GO:0051384(response to glucocorticoid);GO:0005080(protein kinase C binding);GO:0051044(positive regulation of membrane protein ectodomain proteolysis);GO:0031233(intrinsic component of external side of plasma membrane)
circRNA276	GO:0005086(ARF guanyl-nucleotide exchange factor activity);GO:0032012(regulation of ARF protein signal transduction);GO:0043547(positive regulation of GTPase activity);GO:0016020(membrane);GO:0005543(phospholipid binding)
circRNA277	GO:0005086(ARF guanyl-nucleotide exchange factor activity);GO:0032012(regulation of ARF protein signal transduction);GO:0043547(positive regulation of GTPase activity);GO:0016020(membrane);GO:0005543(phospholipid binding)
circRNA278	GO:0005737(cytoplasm);GO:0006516(glycoprotein catabolic process);GO:0003684(damaged DNA binding);GO:0006289(nucleotide-excision repair);GO:0005634(nucleus);GO:0005515(protein binding)
circRNA279	GO:0004871(signal transducer activity);GO:0005622(intracellular);GO:0007165(signal transduction);GO:0007275(multicellular organismal development);GO:0038032(termination of G-protein coupled receptor signaling pathway);GO:0045893("positive regulation of transcription, DNA-templated");GO:0048471(perinuclear region of cytoplasm);GO:0005737(cytoplasm);GO:0005515(protein binding);GO:0005634(nucleus);GO:0030178(negative regulation of Wnt signaling pathway);GO:0031625(ubiquitin protein ligase binding);GO:0019899(enzyme binding);GO:0016328(lateral plasma membrane);GO:0001934(positive regulation of protein phosphorylation);GO:0032947(protein complex scaffold);GO:0046332(SMAD binding);GO:0008013(beta-catenin binding);GO:0051443(positive regulation of ubiquitin-protein transferase activity);GO:0043623(cellular protein complex assembly);GO:0032147(activation of protein kinase activity);GO:0030877(beta-catenin destruction complex);GO:0070411(I-SMAD binding);GO:0071944(cell periphery);GO:0005886(plasma membrane);GO:0060070(canonical Wnt signaling pathway);GO:0042803(protein homodimerization activity);GO:0007605(sensory perception of sound);GO:0030163(protein catabolic process);GO:0042802(identical protein binding);GO:0009950(dorsal/ventral axis specification);GO:0001701(in utero embryonic development);GO:0051260(protein homooligomerization);GO:0019901(protein kinase binding);GO:0090090(negative regulation of canonical Wnt signaling pathway);GO:0008022(protein C-terminus binding);GO:0019904(protein domain specific binding);GO:0016023(cytoplasmic membrane-bounded vesicle);GO:0071514(genetic imprinting);GO:0005938(cell cortex);GO:0043234(protein complex);GO:0005881(cytoplasmic microtubule);GO:0035412(regulation of catenin import into nucleus);GO:0043621(protein self-association);GO:0000209(protein polyubiquitination);GO:0002039(p53 binding);GO:0046330(positive regulation of JNK cascade);GO:0016055(Wnt signaling pathway);GO:0030511(positive regulation of transforming growth factor beta receptor signaling pathway);GO:0006913(nucleocytoplasmic transport);GO:0070412(R-SMAD binding);GO:0031398(positive regulation of protein ubiquitination);GO:0031122(cytoplasmic microtubule organization);GO:0001932(regulation of protein phosphorylation);GO:2000060(positive regulation of protein ubiquitination involved in ubiquitin-dependent protein catabolic process);GO:0032436(positive regulation of proteasomal ubiquitin-dependent protein catabolic process);GO:0036342(post-anal tail morphogenesis);GO:0045599(negative regulation of fat cell differentiation);GO:0043507(positive regulation of JUN kinase activity);GO:0034244(negative regulation of transcription elongation from RNA polymerase II promoter);GO:0048318(axial mesoderm development);GO:0048320(axial mesoderm formation);GO:0051248(negative regulation of protein metabolic process)
circRNA28	GO:0006071(glycerol metabolic process);GO:0006629(lipid metabolic process);GO:0008081(phosphoric diester hydrolase activity);GO:0008889(glycerophosphodiester phosphodiesterase activity);GO:0030246(carbohydrate binding);GO:2001070(starch binding)
circRNA280	GO:0000166(nucleotide binding);GO:0009190(cyclic nucleotide biosynthetic process);GO:0016020(membrane);GO:0016021(integral component of membrane);GO:0016829(lyase activity);GO:0016849(phosphorus-oxygen lyase activity);GO:0035556(intracellular signal transduction);GO:0006171(cAMP biosynthetic process)
circRNA281	GO:0000166(nucleotide binding);GO:0003924(GTPase activity);GO:0005525(GTP binding);GO:0006184(GTP catabolic process);GO:0005515(protein binding);GO:0044822(poly(A) RNA binding);GO:0003747(translation release factor activity);GO:0006415(translational termination);GO:0006479(protein methylation)
circRNA282	GO:0000166(nucleotide binding);GO:0003924(GTPase activity);GO:0005525(GTP binding);GO:0006184(GTP catabolic process);GO:0005515(protein binding);GO:0044822(poly(A) RNA binding);GO:0003747(translation release factor activity);GO:0006415(translational termination);GO:0006479(protein methylation)
circRNA283	()
circRNA284	()
circRNA285	GO:0006024(glycosaminoglycan biosynthetic process);GO:0008375(acetylglucosaminyltransferase activity);GO:0016020(membrane);GO:0030158(protein xylosyltransferase activity);GO:0034605(cellular res
circRNA286	GO:0006024(glycosaminoglycan biosynthetic process);GO:0008375(acetylglucosaminyltransferase activity);GO:0016020(membrane);GO:0030158(protein xylosyltransferase activity);GO:0034605(cellular res
circRNA287	GO:0000166(nucleotide binding);GO:0005524(ATP binding);GO:0016301(kinase activity);GO:0016310(phosphorylation);GO:0016740(transferase activity);GO:0016772("transferase activity, transferring phosphorus-containing groups");GO:0016773("phosphotransferase activity, alcohol group as acceptor");GO:0005515(protein binding);GO:0005488(binding)
circRNA288	GO:0000166(nucleotide binding);GO:0005524(ATP binding);GO:0016301(kinase activity);GO:0016310(phosphorylation);GO:0016740(transferase activity);GO:0016772("transferase activity, transferring phosphorus-containing groups");GO:0016773("phosphotransferase activity, alcohol group as acceptor");GO:0005515(protein binding);GO:0005488(binding)
circRNA289	GO:0004674(protein serine/threonine kinase activity);GO:0004686(elongation factor-2 kinase activity);GO:0005509(calcium ion binding);GO:0005516(calmodulin binding);GO:0005524(ATP binding);GO:0006468(protein phosphorylation);GO:0016772("transferase activity, transferring phosphorus-containing groups");GO:0046777(protein autophosphorylation);GO:0031952(regulation of protein
circRNA29	GO:0006071(glycerol metabolic process);GO:0006629(lipid metabolic process);GO:0008081(phosphoric diester hydrolase activity);GO:0008889(glycerophosphodiester phosphodiesterase activity);GO:0030246(carbohydrate binding);GO:2001070(starch binding)
circRNA290	GO:0003899(DNA-directed RNA polymerase activity);GO:0005634(nucleus);GO:0006351("transcription, DNA-templated");GO:0005813(centrosome)
circRNA291	GO:0006508(proteolysis);GO:0006511(ubiquitin-dependent protein catabolic process);GO:0008233(peptidase activity);GO:0008234(cysteine-type peptidase activity);GO:0016787(hydrolase activity);GO:0036459(ubiquitinyl hydrolase activity);GO:0005634(nucleus);GO:0016579(protein deubiquitination);GO:0004843(ubiquitin-specific protease activity)
circRNA292	GO:0003676(nucleic acid binding);GO:0005634(nucleus);GO:0008270(zinc ion binding);GO:0046872(metal ion binding);GO:0005515(protein binding);GO:0004842(ubiquitin-protein transferase activity);GO:0016567(protein ubiquitination);GO:0044822(poly(A) RNA binding);GO:0042787(protein ubiquitination involved in ubiquitin-dependent protein catabolic process);GO:0035264(multicellular
circRNA293	();GO:0005615(extracellular space);GO:0005886(plasma membrane);GO:0005634(nucleus)
circRNA294	GO:0000166(nucleotide binding);GO:0005388(calcium-transporting ATPase activity);GO:0005524(ATP binding);GO:0006812(cation transport);GO:0006816(calcium ion transport);GO:0008152(metabolic process);GO:0016020(membrane);GO:0016021(integral component of membrane);GO:0016787(hydrolase activity);GO:0019829(cation-transporting ATPase activity);GO:0046872(metal ion binding);GO:0070588(calcium ion transmembrane transport);GO:0005789(endoplasmic reticulum membrane);GO:0005515(protein binding);GO:0006942(regulation of striated muscle contraction);GO:0042803(protein homodimerization activity);GO:0070509(calcium ion import);GO:0031674(I band);GO:0090076(relaxation of skeletal muscle);GO:0051659(maintenance of mitochondrion location);GO:0051561(positive regulation of mitochondrial calcium ion concentration);GO:0045988(negative regulation of striated muscle contraction);GO:0032471(negative regulation of endoplasmic reticulum calcium ion concentration);GO:0032470(positive regulation of endoplasmic reticulum calcium ion concentration);GO:0031673(H zone);GO:0031448(positive regulation of fast-twitch skeletal muscle fiber contraction);GO:0008637(apoptotic mitochondrial changes);GO:0070059(intrinsic apoptotic signaling pathway in response to endoplasmic reticulum stress);GO:0034976(response to endoplasmic reticulum stress)
circRNA295	GO:0000166(nucleotide binding);GO:0005388(calcium-transporting ATPase activity);GO:0005524(ATP binding);GO:0006812(cation transport);GO:0006816(calcium ion transport);GO:0008152(metabolic process);GO:0016020(membrane);GO:0016021(integral component of membrane);GO:0016787(hydrolase activity);GO:0019829(cation-transporting ATPase activity);GO:0046872(metal ion binding);GO:0070588(calcium ion transmembrane transport);GO:0005789(endoplasmic reticulum membrane);GO:0005515(protein binding);GO:0006942(regulation of striated muscle contraction);GO:0042803(protein homodimerization activity);GO:0070509(calcium ion import);GO:0031674(I band);GO:0090076(relaxation of skeletal muscle);GO:0051659(maintenance of mitochondrion location);GO:0051561(positive regulation of mitochondrial calcium ion concentration);GO:0045988(negative regulation of striated muscle contraction);GO:0032471(negative regulation of endoplasmic reticulum calcium ion concentration);GO:0032470(positive regulation of endoplasmic reticulum calcium ion concentration);GO:0031673(H zone);GO:0031448(positive regulation of fast-twitch skeletal muscle fiber contraction);GO:0008637(apoptotic mitochondrial changes);GO:0070059(intrinsic apoptotic signaling pathway in response to endoplasmic reticulum stress);GO:0034976(response to endoplasmic reticulum stress)
circRNA296	GO:0007155(cell adhesion);GO:0007229(integrin-mediated signaling pathway);GO:0008305(integrin complex);GO:0016020(membrane);GO:0016021(integral component of membrane);GO:0070062(extracellular vesicular exosome);GO:0005515(protein binding);GO:0009986(cell surface);GO:0001948(glycoprotein binding);GO:0016337(single organismal cell-cell adhesion);GO:0005634(nucleus);GO:0009897(external side of plasma membrane);GO:0008201(heparin binding);GO:0007159(leukocyte cell-cell adhesion);GO:0043395(heparan sulfate proteoglycan binding);GO:0030593(neutrophil chemotaxis);GO:0050798(activated T cell proliferation);GO:0002523(leukocyte migration involved in inflammatory response);GO:0045123(cellular extravasation);GO:0001846(opsonin binding);GO:0014005(microglia development)
circRNA297	GO:0005096(GTPase activator activity);GO:0005622(intracellular);GO:0007165(signal transduction);GO:0035556(intracellular signal transduction);GO:0043547(positive regulation of GTPase activity);GO:0051056(regulation of small GTPase mediated signal transduction);GO:0005515(protein binding)
circRNA298	GO:0005515(protein binding);GO:0005634(nucleus);GO:0003682(chromatin binding);GO:0035064(methylated histone binding);GO:0006325(chromatin organization);GO:0005664(nuclear origin of replication recognition complex);GO:0005721(centromeric heterochromatin);GO:0031933(telomeric heterochromatin);GO:0008327(methyl-CpG binding);GO:0071169(establishment of protein localization
circRNA299	GO:0003676(nucleic acid binding);GO:0005622(intracellular);GO:0006355("regulation of transcription, DNA-templated");GO:0046872(metal ion binding);GO:0005634(nucleus);GO:0045892("negative regulation of transcription, DNA-templated")

circRNA3	GO:0005509(calcium ion binding);GO:0005515(protein binding)
circRNA30	GO:0006071(glycerol metabolic process);GO:0006629(lipid metabolic process);GO:0008081(phosphoric diester hydrolase activity);GO:0008889(glycerophosphodiester phosphodiesterase activity);GO:0030246(carbohydrate binding);GO:2001070(starch binding)
circRNA300	GO:0005515(protein binding)
circRNA301	GO:0005813(centrosome);GO:0005739(mitochondrion);GO:0005515(protein binding);GO:0030177(positive regulation of Wnt signaling pathway);GO:0000226(microtubule cytoskeleton organization);GO:0002052(positive regulation of neuroblast proliferation);GO:0001764(neuron migration);GO:0060070(canonical Wnt signaling pathway);GO:0034613(cellular protein localization);GO:0036064(ciliary basal body);GO:0031929(TOR signaling);GO:0010975(regulation of neuron projection development);GO:0021799(cerebral cortex radially oriented cell migration);GO:0008104(protein localization);GO:2000060(positive regulation of protein ubiquitination involved in ubiquitin-dependent protein catabolic process);GO:0051560(mitochondrial calcium ion homeostasis);GO:0021846(cell proliferation in forebrain);GO:0090128(regulation of synapse maturation)
circRNA302	GO:0004672(protein kinase activity);GO:0004674(protein serine/threonine kinase activity);GO:0005524(ATP binding);GO:0006468(protein phosphorylation);GO:0016772("transferase activity, transferring phosphorus-containing groups");GO:0004713(protein tyrosine kinase activity)
circRNA303	GO:0003779(actin binding);GO:0005509(calcium ion binding);GO:0005515(protein binding);GO:0008092(cytoskeletal protein binding);GO:0070062(extracellular vesicular exosome);GO:0046983(protein dimerization activity);GO:0005925(focal adhesion);GO:0030018(Z disc);GO:0042391(regulation of membrane potential);GO:0051289(protein homotetramerization);GO:0042802(identical protein
circRNA304	GO:0003779(actin binding);GO:0005509(calcium ion binding);GO:0005515(protein binding);GO:0008092(cytoskeletal protein binding);GO:0070062(extracellular vesicular exosome);GO:0046983(protein dimerization activity);GO:0005925(focal adhesion);GO:0030018(Z disc);GO:0042391(regulation of membrane potential);GO:0051289(protein homotetramerization);GO:0042802(identical protein
circRNA305	GO:0003723(RNA binding);GO:0005515(protein binding);GO:0044822(poly(A) RNA binding);GO:0090090(negative regulation of canonical Wnt signaling pathway)
circRNA306	()
circRNA307	()
circRNA308	GO:0003677(DNA binding);GO:0005622(intracellular);GO:0005634(nucleus);GO:0005515(protein binding);GO:0003713(transcription coactivator activity);GO:0051091(positive regulation of sequence-specific DNA binding transcription factor activity);GO:0044212(transcription regulatory region DNA binding);GO:0008584(male gonad development);GO:0035264(multicellular organism growth);GO:0009791(post-embryonic development);GO:0010761(fibroblast migration);GO:0060021(palate development);GO:0045444(fat cell differentiation);GO:0060612(adipose tissue development);GO:0008585(female gonad development);GO:0048705(skeletal system morphogenesis);GO:0060325(face morphogenesis);GO:0001822(kidney development);GO:0048008(platelet-derived growth factor receptor signaling pathway);GO:0048468(cell development);GO:0048644(muscle organ morphogenesis);GO:0030325(adrenal gland development);GO:0006807(nitrogen compound metabolic process);GO:0060613(fat pad development)
circRNA309	GO:0005198(structural molecule activity);GO:0007155(cell adhesion);GO:0015629(actin cytoskeleton)
circRNA31	GO:0004594(pantothenate kinase activity);GO:0005524(ATP binding);GO:0015937(coenzyme A biosynthetic process);GO:0016310(phosphorylation);GO:0005739(mitochondrion);GO:0005829(cytosol);GO:0051881(regulation of mitochondrial membrane potential);GO:0070584(mitochondrion
circRNA310	GO:0004842(ubiquitin-protein transferase activity);GO:0008152(metabolic process);GO:0016567(protein ubiquitination);GO:0016874(ligase activity);GO:0007283(spermatogenesis)
circRNA311	GO:0005515(protein binding);GO:0008092(cytoskeletal protein binding);GO:0030018(Z disc);GO:0017124(SH3 domain binding);GO:0031674(I band);GO:0051371(muscle alpha-actinin binding);GO:00452
circRNA312	GO:0000166(nucleotide binding);GO:0004396(hexokinase activity);GO:0005524(ATP binding);GO:0005975(carbohydrate metabolic process);GO:0006096(glycolytic process);GO:0016301(kinase activity);GO:0016310(phosphorylation);GO:0016740(transferase activity);GO:0016773("phosphotransferase activity, alcohol group as acceptor");GO:0046835(carbohydrate phosphorylation);GO:0005515(protein binding);GO:0097228(sperm principal piece);GO:0005739(mitochondrion);GO:0005829(cytosol);GO:0045121(membrane raft);GO:0005929(cilium)
circRNA313	GO:0003723(RNA binding);GO:0003824(catalytic activity);GO:0005737(cytoplasm);GO:0006355("regulation of transcription, DNA-templated");GO:0008152(metabolic process);GO:0005667(transcription f
circRNA314	GO:0000165(MAPK cascade);GO:0000166(nucleotide binding);GO:0004672(protein kinase activity);GO:0004674(protein serine/threonine kinase activity);GO:0004707(MAP kinase activity);GO:0005524(ATP binding);GO:0006468(protein phosphorylation);GO:0016301(kinase activity);GO:0016310(phosphorylation);GO:0016740(transferase activity);GO:0016772("transferase activity, transferring phosphorus-containing groups");GO:0016773("phosphotransferase activity, alcohol group as acceptor");GO:0009103(lipopolysaccharide biosynthetic process);GO:0016020(membrane);GO:0004713(protein tyrosine kinase activity);GO:0005515(protein binding);GO:0005634(nucleus);GO:0010628(positive regulation of gene expression);GO:0043066(negative regulation of apoptotic process);GO:0071222(cellular response to lipopolysaccharide);GO:0032091(negative regulation of protein binding);GO:0009411(response to UV);GO:0071260(cellular response to mechanical stimulus);GO:0031063(regulation of histone deacetylation);GO:0035033(histone deacetylase regulator activity);GO:0007258(JUN phosphorylation);GO:0004705(JUN kinase activity);GO:0090045(positive regulation of deacetylase activity);GO:0032880(regulation of protein localization);GO:0042826(histone deacetylase binding);GO:0018105(peptidyl-serine phosphorylation);GO:0018107(peptidyl-threonine phosphorylation);GO:0007254(JNK cascade);GO:0005737(cytoplasm);GO:0005739(mitochondrion);GO:0010468(regulation of gene expression);GO:0070301(cellular response to hydrogen peroxide);GO:0005829(cytosol);GO:0001503(ossification);GO:0046686(response to cadmium ion);GO:2001235(positive regulation of apoptotic signaling pathway);GO:0071732(cellular response to nitric oxide);GO:0097300(programmed necrotic cell death);GO:2000017(positive regulation of determination of dorsal identity)
circRNA315	GO:0008152(metabolic process);GO:0016874(ligase activity);GO:0016881(acid-amino acid ligase activity);GO:0006511(ubiquitin-dependent protein catabolic process);GO:0005737(cytoplasm);GO:0005515(protein binding);GO:0004842(ubiquitin-protein transferase activity);GO:0000209(protein polyubiquitination);GO:0031398(positive regulation of protein ubiquitination);GO:0070936(protein K48-linked ubiquitination);GO:0000151(ubiquitin ligase complex);GO:0043234(protein complex)
circRNA316	GO:0000166(nucleotide binding);GO:0004672(protein kinase activity);GO:0004674(protein serine/threonine kinase activity);GO:0004692(cGMP-dependent protein kinase activity);GO:0005524(ATP binding);GO:0006468(protein phosphorylation);GO:0016301(kinase activity);GO:0016310(phosphorylation);GO:0016740(transferase activity);GO:0016772("transferase activity, transferring phosphorus-containing groups");GO:0004713(protein tyrosine kinase activity);GO:0005737(cytoplasm);GO:0005515(protein binding);GO:0005886(plasma membrane);GO:0005246(calcium channel regulator activity);GO:0043087(regulation of GTPase activity);GO:0090331(negative regulation of platelet aggregation);GO:0001764(neuron migration);GO:0030900(forebrain development);GO:0005794(Golgi apparatus);GO:0007165(signal transduction);GO:0016358(dendrite development);GO:0030553(cGMP binding);GO:0019934(cGMP-mediated signaling);GO:0045986(negative regulation of smooth muscle contraction);GO:0060087(relaxation of vascular smooth muscle)
circRNA317	GO:0000166(nucleotide binding);GO:0004672(protein kinase activity);GO:0004674(protein serine/threonine kinase activity);GO:0004692(cGMP-dependent protein kinase activity);GO:0005524(ATP binding);GO:0006468(protein phosphorylation);GO:0016301(kinase activity);GO:0016310(phosphorylation);GO:0016740(transferase activity);GO:0016772("transferase activity, transferring phosphorus-containing groups");GO:0004713(protein tyrosine kinase activity);GO:0005737(cytoplasm);GO:0005515(protein binding);GO:0005886(plasma membrane);GO:0005246(calcium channel regulator activity);GO:0043087(regulation of GTPase activity);GO:0090331(negative regulation of platelet aggregation);GO:0001764(neuron migration);GO:0030900(forebrain development);GO:0005794(Golgi apparatus);GO:0007165(signal transduction);GO:0016358(dendrite development);GO:0030553(cGMP binding);GO:0019934(cGMP-mediated signaling);GO:0045986(negative regulation of smooth muscle contraction);GO:0060087(relaxation of vascular smooth muscle)
circRNA318	GO:0005515(protein binding);GO:0003950(NAD+ ADP-ribosyltransferase activity);GO:0048471(perinuclear region of cytoplasm);GO:0005737(cytoplasm);GO:0005634(nucleus);GO:0000209(protein polyubiquitination);GO:0090263(positive regulation of canonical Wnt signaling pathway);GO:0019899(enzyme binding);GO:0005635(nuclear envelope);GO:0000242(pericentriolar material);GO:0006471(protein ADP-ribosylation);GO:0070213(protein auto-ADP-ribosylation);GO:0070198("protein localization to chromosome, telomeric region");GO:0035264(multicellular organism growth);GO:0040014(regulation of multicellular organism growth)
circRNA319	GO:0003676(nucleic acid binding);GO:0003677(DNA binding);GO:0004386(helicase activity);GO:0005524(ATP binding);GO:0008152(metabolic process);GO:0005515(protein binding);GO:0005488(binding);GO:0035562(negative regulation of chromatin binding)
circRNA32	()
circRNA320	GO:0000166(nucleotide binding);GO:0003676(nucleic acid binding);GO:0005515(protein binding);GO:0000122(negative regulation of transcription from RNA polymerase II promoter);GO:0044822(poly(A) RNA binding);GO:0005634(nucleus);GO:1900153("positive regulation of nuclear-transcribed mRNA catabolic process, deadenylation-dependent
circRNA321	GO:0005515(protein binding)
circRNA322	GO:0007015(actin filament organization);GO:0008286(insulin receptor signaling pathway);GO:0005515(protein binding);GO:0005634(nucleus);GO:0005158(insulin receptor binding);GO:0005899(insulin receptor complex);GO:0019901(protein kinase binding);GO:0001725(stress fiber)
circRNA323	GO:0007015(actin filament organization);GO:0008286(insulin receptor signaling pathway);GO:0005515(protein binding);GO:0005634(nucleus);GO:0005158(insulin receptor binding);GO:0005899(insulin receptor complex);GO:0019901(protein kinase binding);GO:0001725(stress fiber)
circRNA324	GO:0005515(protein binding);GO:0051216(cartilage development);GO:0042733(embryonic digit morphogenesis);GO:0002053(positive regulation of mesenchymal cell proliferation);GO:0060173(limb develo
circRNA325	GO:0005515(protein binding);GO:0051216(cartilage development);GO:0042733(embryonic digit morphogenesis);GO:0002053(positive regulation of mesenchymal cell proliferation);GO:0060173(limb develo
circRNA326	GO:0005515(protein binding);GO:0051216(cartilage development);GO:0042733(embryonic digit morphogenesis);GO:0002053(positive regulation of mesenchymal cell proliferation);GO:0060173(limb develo
circRNA327	GO:0005515(protein binding);GO:0051216(cartilage development);GO:0042733(embryonic digit morphogenesis);GO:0002053(positive regulation of mesenchymal cell proliferation);GO:0060173(limb develo
circRNA328	GO:0005509(calcium ion binding);GO:0046872(metal ion binding);GO:0071805(potassium ion transmembrane transport);GO:0005737(cytoplasm);GO:0005515(protein binding);GO:0047485(protein N-terminus binding);GO:0015459(potassium channel regulator activity);GO:0042802(identical protein binding);GO:0045163(clustering of voltage-gated potassium channels);GO:0086009(membrane
circRNA329	GO:0006886(intracellular protein transport);GO:0016020(membrane);GO:0016192(vesicle-mediated transport);GO:0005515(protein binding);GO:0005794(Golgi apparatus);GO:0042147("retrograde transport, endosome to Golgi");GO:0005484(SNAP receptor activity)
circRNA33	GO:0004725(protein tyrosine phosphatase activity);GO:0006470(protein dephosphorylation);GO:0016311(dephosphorylation);GO:0016787(hydrolase activity);GO:0016791(phosphatase activity);GO:0035335(peptidyl-tyrosine dephosphorylation);GO:0070062(extracellular vesicular exosome);GO:0005515(protein binding);GO:0043235(receptor complex);GO:0006468(protein phosphorylation);GO:0008286(insulin receptor signaling pathway);GO:0004721(phosphoprotein phosphatase activity)
circRNA330	GO:0003779(actin binding);GO:0008270(zinc ion binding);GO:0046872(metal ion binding);GO:0005515(protein binding);GO:0005916(fascia adherens);GO:0051371(muscle alpha-actinin binding);GO:0005927(muscle tendon junction);GO:0017166(vinculin binding);GO:0030016(myofibril)
circRNA331	GO:0003779(actin binding);GO:0008270(zinc ion binding);GO:0046872(metal ion binding);GO:0005515(protein binding);GO:0005916(fascia adherens);GO:0051371(muscle alpha-actinin binding);GO:0005927(muscle tendon junction);GO:0017166(vinculin binding);GO:0030016(myofibril)
circRNA332	GO:0008152(metabolic process);GO:0042578(phosphoric ester hydrolase activity);GO:0014898(cardiac muscle hypertrophy in response to stress);GO:0048015(phosphatidylinositol-mediated signaling);GO:0031161(phosphatidylinositol catabolic process)

circRNA333	();GO:0005730(nucleolus);GO:0005737(cytoplasm);GO:0005634(nucleus);GO:0005794(Golgi apparatus);GO:0035257(nuclear hormone receptor binding);GO:0019904(protein domain specific binding);GO:0000226(microtubule cytoskeleton organization);GO:0008283(cell proliferation);GO:0022008(neurogenesis);GO:0021987(cerebral cortex development);GO:0022027(interkinetic nuclear migration);GO:0030953(astral microtubule organization);GO:0032886(regulation of microtubule-based process)
circRNA334	();GO:0005730(nucleolus);GO:0005737(cytoplasm);GO:0005634(nucleus);GO:0005794(Golgi apparatus);GO:0035257(nuclear hormone receptor binding);GO:0019904(protein domain specific binding);GO:0000226(microtubule cytoskeleton organization);GO:0008283(cell proliferation);GO:0022008(neurogenesis);GO:0021987(cerebral cortex development);GO:0022027(interkinetic nuclear migration);GO:0030953(astral microtubule organization);GO:0032886(regulation of microtubule-based process)
circRNA335	();GO:0005730(nucleolus);GO:0005737(cytoplasm);GO:0005634(nucleus);GO:0005794(Golgi apparatus);GO:0035257(nuclear hormone receptor binding);GO:0019904(protein domain specific binding);GO:0000226(microtubule cytoskeleton organization);GO:0008283(cell proliferation);GO:0022008(neurogenesis);GO:0021987(cerebral cortex development);GO:0022027(interkinetic nuclear migration);GO:0030953(astral microtubule organization);GO:0032886(regulation of microtubule-based process)
circRNA336	GO:0005085(guanyl-nucleotide exchange factor activity);GO:0007264(small GTPase mediated signal transduction);GO:0043547(positive regulation of GTPase activity);GO:0005515(protein binding);GO:0005737(cytoplasm);GO:0005634(nucleus);GO:0002244(hematopoietic progenitor cell differentiation);GO:0016477(cell migration)
circRNA337	GO:0005085(guanyl-nucleotide exchange factor activity);GO:0007264(small GTPase mediated signal transduction);GO:0043547(positive regulation of GTPase activity);GO:0005515(protein binding);GO:0005737(cytoplasm);GO:0005634(nucleus);GO:0002244(hematopoietic progenitor cell differentiation);GO:0016477(cell migration)
circRNA338	GO:0006810(transport);GO:0006869(lipid transport);GO:0005515(protein binding);GO:0070062(extracellular vesicular exosome);GO:0005737(cytoplasm);GO:0005634(nucleus);GO:0015485(cholesterol binding);GO:0005770(late endosome)
circRNA339	GO:0006810(transport);GO:0006869(lipid transport);GO:0005515(protein binding);GO:0070062(extracellular vesicular exosome);GO:0005737(cytoplasm);GO:0005634(nucleus);GO:0015485(cholesterol binding);GO:0005770(late endosome)
circRNA34	GO:0004725(protein tyrosine phosphatase activity);GO:0006470(protein dephosphorylation);GO:0016311(dephosphorylation);GO:0016787(hydrolase activity);GO:0016791(phosphatase activity);GO:0035335(peptidyl-tyrosine dephosphorylation);GO:0070062(extracellular vesicular exosome);GO:0005515(protein binding);GO:0043235(receptor complex);GO:0006468(protein phosphorylation);GO:0008286(insulin receptor signaling pathway);GO:0004721(phosphoprotein phosphatase activity)
circRNA340	GO:0006810(transport);GO:0006869(lipid transport);GO:0005515(protein binding);GO:0070062(extracellular vesicular exosome);GO:0005737(cytoplasm);GO:0005634(nucleus);GO:0015485(cholesterol binding);GO:0005770(late endosome)
circRNA341	()
circRNA342	GO:0016538(cyclin-dependent protein serine/threonine kinase regulator activity);GO:0045859(regulation of protein kinase activity);GO:0051302(regulation of cell division);GO:0051726(regulation of cell cycle);GO:0005829(cytosol);GO:0005515(protein binding);GO:0007399(nervous system development)
circRNA343	GO:0004842(ubiquitin-protein transferase activity);GO:0008270(zinc ion binding);GO:0016567(protein ubiquitination);GO:0046872(metal ion binding);GO:0005515(protein binding);GO:0005813(centrosome);GO:0031410(cytoplasmic vesicle);GO:0001568(blood vessel development);GO:0045665(negative regulation of neuron differentiation);GO:0001701(in utero embryonic development);GO:0001841(neural tube formation);GO:0007507(heart development);GO:0001756(somitogenesis);GO:0001947(heart looping);GO:0007219(Notch signaling pathway);GO:0045807(positive regulation of endocytosis);()
circRNA344	()
circRNA345	GO:0003713(transcription coactivator activity);GO:0006629(lipid metabolic process);GO:0008195(phosphatidate phosphatase activity);GO:0016311(dephosphorylation);GO:0045944(positive regulation of transcription from RNA polymerase II promoter);GO:0005789(endoplasmic reticulum membrane);GO:0005829(cytosol)
circRNA346	GO:0005739(mitochondrion)
circRNA347	GO:0030036(actin cytoskeleton organization);GO:0051295(establishment of meiotic spindle localization);GO:0005794(Golgi apparatus);GO:0048193(Golgi vesicle transport)
circRNA348	GO:0060348(bone development);GO:0005737(cytoplasm);GO:0005515(protein binding);GO:0005794(Golgi apparatus);GO:0019899(enzyme binding);GO:0007030(Golgi organization);()
circRNA349	GO:0000166(nucleotide binding);GO:0005525(GTP binding);GO:0007264(small GTPase mediated signal transduction);GO:0015031(protein transport);GO:0003924(GTPase activity);GO:0006184(GTP catabolic process);GO:0006886(intracellular protein transport);GO:0006913(nucleocytoplasmic transport);GO:0007165(signal transduction);GO:0005622(intracellular);GO:0016020(membrane);GO:0070062(extracellular vesicular exosome);GO:0019904(protein domain specific binding);GO:0071985(multivesicular body sorting pathway);GO:0045921(positive regulation of exocytosis);GO:0032585(multivesicular body membrane);GO:0030140(trans-Golgi network transport vesicle);GO:0019003(GDP binding);GO:0005795(Golgi stack);GO:0005515(protein binding);GO:0005794(Golgi apparatus)
circRNA35	GO:0005484(SNAP receptor activity);GO:0006886(intracellular protein transport);GO:0006891(intra-Golgi vesicle-mediated transport);GO:0016020(membrane);GO:0016192(vesicle-mediated transport);GO:0031201(SNARE complex);GO:0005515(protein binding);GO:0005794(Golgi apparatus)
circRNA350	()
circRNA351	GO:0046983(protein dimerization activity);GO:0005515(protein binding);GO:0005634(nucleus);GO:0003677(DNA binding);GO:0005667(transcription factor complex);GO:0045944(positive regulation of transcription from RNA polymerase II promoter);GO:0006355("regulation of transcription, DNA-templated");GO:0000122(negative regulation of transcription from RNA polymerase II promoter);GO:0003714(transcription corepressor activity);GO:0046982(protein heterodimerization activity);GO:0006367(transcription initiation from RNA polymerase II promoter);GO:0070888(E-box binding);GO:0045666(positive regulation of neuron differentiation);GO:0006352("DNA-templated transcription, initiation");GO:0000978(RNA polymerase II core promoter proximal region sequence-specific DNA binding);GO:0001077(RNA polymerase II core promoter proximal region sequence-specific DNA binding transcription factor activity involved in positive regulation of transcription);GO:0065004(protein-DNA complex assembly);GO:0001011(sequence-specific DNA binding RNA polymerase recruiting transcription factor activity);GO:0001087(TFIIB-class binding transcription factor activity);GO:0001093(TFIIB-class transcription factor binding)
circRNA352	GO:0046872(metal ion binding)
circRNA353	GO:0000166(nucleotide binding);GO:0005525(GTP binding);GO:0007264(small GTPase mediated signal transduction);GO:0015031(protein transport);GO:0003924(GTPase activity);GO:0006184(GTP catabolic process);GO:0006886(intracellular protein transport);GO:0006913(nucleocytoplasmic transport);GO:0007165(signal
circRNA354	GO:0000166(nucleotide binding);GO:0005525(GTP binding);GO:0007264(small GTPase mediated signal transduction);GO:0015031(protein transport);GO:0003924(GTPase activity);GO:0006184(GTP catabolic process);GO:0006886(intracellular protein transport);GO:0006913(nucleocytoplasmic transport);GO:0007165(signal
circRNA355	GO:0005635(nuclear envelope);GO:0016605(PML body);GO:0031981(nuclear lumen)
circRNA356	GO:0006457(protein folding);GO:0005515(protein binding);GO:0070062(extracellular vesicular exosome);GO:0016020(membrane);GO:0005634(nucleus);GO:0031072(heat shock protein binding);GO:0003755(peptidyl-prolyl cis-trans isomerase activity);GO:0061077(chaperone-mediated protein folding)
circRNA357	GO:0005089(Rho guanyl-nucleotide exchange factor activity);GO:0032321(positive regulation of Rho GTPase activity);GO:0035023(regulation of Rho protein signal transduction);GO:0046872(metal ion bin
circRNA358	GO:0005247(voltage-gated chloride channel activity);GO:0006821(chloride transport);GO:0016020(membrane);GO:0044070(regulation of anion transport);GO:0070062(extracellular vesicular exosome);GO:0005515(protein binding);GO:0015629(actin cytoskeleton);GO:0007605(sensory perception of sound);GO:0050885(neuromuscular process controlling balance);GO:0032420(stereocilium);GO:0002021(response to dietary excess);GO:0008104(protein localization);GO:0060088(auditory receptor cell stereocilium organization);GO:0002024(diet induced thermogenesis)
circRNA359	GO:0010824(regulation of centrosome duplication);GO:0005515(protein binding);GO:0005813(centrosome);GO:0048471(perinuclear region of cytoplasm);GO:0070062(extracellular vesicular exosome);GO:0005737(cytoplasm);GO:0072372(primary cilium);GO:0032088(negative regulation of NF-kappaB transcription factor activity);GO:0043066(negative regulation of apoptotic process);GO:0008284(positive regulation of cell proliferation);GO:0070372(regulation of ERK1 and ERK2 cascade);GO:0016324(apical plasma membrane);GO:0036064(ciliary basal body);GO:0006874(cellular calcium ion homeostasis);GO:0072686(mitotic spindle);GO:0032006(regulation of TOR signaling);GO:0051898(negative regulation of protein kinase B
circRNA36	GO:0000166(nucleotide binding);GO:0004672(protein kinase activity);GO:0004674(protein serine/threonine kinase activity);GO:0005524(ATP binding);GO:0006468(protein phosphorylation);GO:0016772("transferase activity, transferring phosphorus-containing groups");GO:0004713(protein tyrosine kinase activity)
circRNA360	GO:0010824(regulation of centrosome duplication);GO:0005515(protein binding);GO:0005813(centrosome);GO:0048471(perinuclear region of cytoplasm);GO:0070062(extracellular vesicular exosome);GO:0005737(cytoplasm);GO:0072372(primary cilium);GO:0032088(negative regulation of NF-kappaB transcription factor activity);GO:0043066(negative regulation of apoptotic process);GO:0008284(positive regulation of cell proliferation);GO:0070372(regulation of ERK1 and ERK2 cascade);GO:0016324(apical plasma membrane);GO:0036064(ciliary basal body);GO:0006874(cellular calcium ion homeostasis);GO:0072686(mitotic spindle);GO:0032006(regulation of TOR signaling);GO:0051898(negative regulation of protein kinase B signaling);GO:0042384(cilium assembly);GO:0001822(kidney development)
circRNA361	GO:0015095(magnesium ion transmembrane transporter activity);GO:0015693(magnesium ion transport);GO:0005739(mitochondrion)
circRNA362	GO:0000902(cell morphogenesis);GO:0003779(actin binding);GO:0007010(cytoskeleton organization)
circRNA363	GO:0003824(catalytic activity);GO:0003920(GMP reductase activity);GO:0009117(nucleotide metabolic process);GO:0016491(oxidoreductase activity);GO:0046872(metal ion binding);GO:0055114(oxidation-reduction process);GO:1902560(GMP reductase complex)
circRNA364	GO:0003824(catalytic activity);GO:0003920(GMP reductase activity);GO:0009117(nucleotide metabolic process);GO:0016491(oxidoreductase activity);GO:0046872(metal ion binding);GO:0055114(oxidation-reduction process);GO:1902560(GMP reductase complex)
circRNA365	GO:0003824(catalytic activity);GO:0005634(nucleus);GO:0008152(metabolic process)
circRNA366	GO:0000166(nucleotide binding);GO:0003676(nucleic acid binding);GO:0004812(aminoacyl-tRNA ligase activity);GO:0004816(asparagine-tRNA ligase activity);GO:0005524(ATP binding);GO:0005737(cytoplasm);GO:0006412(translation);GO:0006418(tRNA aminoacylation for protein translation);GO:0006421(asparaginyl-tRNA aminoacylation);GO:0016874(ligase activity)
circRNA367	GO:0000166(nucleotide binding);GO:0003676(nucleic acid binding);GO:0004812(aminoacyl-tRNA ligase activity);GO:0004816(asparagine-tRNA ligase activity);GO:0005524(ATP binding);GO:0005737(cytoplasm);GO:0006412(translation);GO:0006418(tRNA aminoacylation for protein translation);GO:0006421(asparaginyl-tRNA aminoacylation);GO:0016874(ligase activity)
circRNA368	GO:0006355("regulation of transcription, DNA-templated");GO:0008270(zinc ion binding);GO:0031213(RSF complex);GO:0046872(metal ion binding);GO:0005515(protein binding);GO:0045893("positive regulation of transcription, DNA-templated");GO:0005634(nucleus);GO:0006334(nucleosome assembly);GO:0045892("negative regulation of transcription, DNA-templated");GO:0006352("DNA-templated transcription, initiation");GO:0043392(negative regulation of DNA binding);GO:0050434(positive regulation of viral transcription);GO:0042393(histone binding);GO:0006338(chromatin remodeling);GO:0016584(nucleosome positioning)

circRNA369	GO:0003677(DNA binding);GO:0003700(sequence-specific DNA binding transcription factor activity);GO:0005634(nucleus);GO:0006355("regulation of transcription, DNA-templated");GO:0043565(sequence-specific DNA binding);GO:0005737(cytoplasm);GO:0006357(regulation of transcription from RNA polymerase II promoter);GO:0051015(actin filament binding);GO:0015630(microtubule cytoskeleton);GO:0015629(actin cytoskeleton);GO:0003785(actin monomer binding)
circRNA37	GO:0016021(integral component of membrane)
circRNA370	GO:0005622(intracellular);GO:0007165(signal transduction);GO:0035091(phosphatidylinositol binding);GO:0005515(protein binding);GO:0015629(actin cytoskeleton);GO:0005938(cell cortex)
circRNA371	GO:0004867(serine-type endopeptidase inhibitor activity);GO:0008201(heparin binding);GO:0010951(negative regulation of endopeptidase activity);GO:0016021(integral component of membrane);GO:0046914(transition metal ion binding);GO:0070062(extracellular vesicular exosome);GO:0016020(membrane);GO:0005515(protein binding);GO:0005634(nucleus);GO:0042802(identical protein binding);GO:0007626(locomotory behavior);GO:0030900(forebrain development);GO:0030198(extracellular matrix organization);GO:0050885(neuromuscular process controlling balance);GO:0006878(cellular copper ion homeostasis);GO:0001967(suckling behavior);GO:0008203(cholesterol metabolic process);GO:0030901(midbrain development);GO:0043393(regulation of protein binding);GO:0007617(mating behavior);GO:0007176(regulation of epidermal growth factor-activated receptor activity)
circRNA372	GO:0005506(iron ion binding);GO:0006629(lipid metabolic process);GO:0006633(fatty acid biosynthetic process);GO:0016717("oxidoreductase activity, acting on paired donors, with oxidation of a pair of donors resulting in the reduction of molecular oxygen to two molecules of water");GO:0020037(heme binding);GO:0055114(oxidation-reduction process);GO:0016020(membrane);GO:0004768(stearoyl-
circRNA373	GO:0005737(cytoplasm);GO:0005515(protein binding);GO:0005886(plasma membrane);GO:0005634(nucleus);GO:0019903(protein phosphatase binding);GO:0043666(regulation of phosphoprotein phosphatase activity)
circRNA374	GO:0003677(DNA binding);GO:0005515(protein binding);GO:0033599(regulation of mammary gland epithelial cell proliferation);GO:0006357(regulation of transcription from RNA polymerase II promoter);GO:0005667(transcription factor complex);GO:0006366(transcription from RNA polymerase II promoter);GO:0048706(embryonic skeletal system development);GO:0001843(neural tube closure)
circRNA375	GO:0005515(protein binding);GO:0070062(extracellular vesicular exosome);GO:0016020(membrane);GO:0005634(nucleus);GO:0005838(proteasome regulatory particle);GO:0007127(meiosis I);GO:0022624(proteasome accessory complex)
circRNA376	GO:0051592(response to calcium ion);GO:0044325(ion channel binding);GO:0005886(plasma membrane);GO:0005515(protein binding);GO:0005737(cytoplasm);GO:0030018(Z disc);GO:0016020(membrane);GO:0043005(neuron projection);GO:0030424(axon);GO:0003009(skeletal muscle contraction);GO:0045177(apical part of cell);GO:0097481(neuronal postsynaptic density);GO:0048741(skeletal muscle fiber development);GO:0043034(costamere);GO:0048148(behavioral response to cocaine);GO:0051928(positive regulation of calcium ion transport);GO:0090279(regulation of calcium ion import);GO:0048875(chemical homeostasis within a tissue);GO:2001256(regulation of store-operated calcium entry);GO:2001257(regulation of cation channel activity)
circRNA377	GO:0051592(response to calcium ion);GO:0044325(ion channel binding);GO:0005886(plasma membrane);GO:0005515(protein binding);GO:0005737(cytoplasm);GO:0030018(Z disc);GO:0016020(membrane);GO:0043005(neuron projection);GO:0030424(axon);GO:0003009(skeletal muscle contraction);GO:0045177(apical part of cell);GO:0097481(neuronal postsynaptic density);GO:0048741(skeletal muscle fiber development);GO:0043034(costamere);GO:0048148(behavioral response to cocaine);GO:0051928(positive regulation of calcium ion transport);GO:0090279(regulation of calcium ion import);GO:0048875(chemical homeostasis within a tissue);GO:2001256(regulation of store-operated calcium entry);GO:2001257(regulation of cation channel activity)
circRNA378	GO:0051592(response to calcium ion);GO:0044325(ion channel binding);GO:0005886(plasma membrane);GO:0005515(protein binding);GO:0005737(cytoplasm);GO:0030018(Z disc);GO:0016020(membrane);GO:0043005(neuron projection);GO:0030424(axon);GO:0003009(skeletal muscle contraction);GO:0045177(apical part of cell);GO:0097481(neuronal postsynaptic density);GO:0048741(skeletal muscle fiber development);GO:0043034(costamere);GO:0048148(behavioral response to cocaine);GO:0051928(positive regulation of calcium ion transport);GO:0090279(regulation of calcium ion import);GO:0048875(chemical homeostasis within a tissue);GO:2001256(regulation of store-operated calcium entry);GO:2001257(regulation of cation channel activity)
circRNA379	GO:0005515(protein binding);GO:0042802(identical protein binding);GO:0070885(negative regulation of calcineurin-NFAT signaling cascade);GO:0043034(costamere);GO:0014733(regulation of skeletal muscle adaptation);GO:0032513(negative regulation of protein phosphatase type 2B activity)
circRNA38	GO:0003824(catalytic activity);GO:0008081(phosphoric diester hydrolase activity);GO:0008152(metabolic process);GO:0046872(metal ion binding);GO:0005515(protein binding);GO:0003676(nucleic acid binding);GO:0008270(zinc ion binding);GO:0005886(plasma membrane);GO:0005634(nucleus);GO:0003723(RNA binding);GO:0051289(protein homotetramerization);GO:0051607(defense response to virus);GO:0032567(dGTP binding);GO:0008832(dGTPase activity);GO:0046061(dATP catabolic process);GO:0006203(dGTP catabolic process);GO:0045088(regulation of innate immune response)
circRNA380	GO:0000166(nucleotide binding);GO:0005524(ATP binding);GO:0006457(protein folding);GO:0017111(nucleoside-triphosphatase activity);GO:0051082(unfolded protein binding);GO:0008134(transcription factor binding);GO:0006355("regulation of transcription, DNA-templated");GO:0016887(ATPase activity);GO:0005739(mitochondrion);GO:0005515(protein binding);GO:0005759(mitochondrial matrix);GO:0006200(ATP catabolic process);GO:0051603(proteolysis involved in cellular protein catabolic process);GO:0042645(mitochondrial nucleoid);GO:0004176(ATP-dependent peptidase activity);GO:0016504(peptidase activator activity);GO:0010952(positive regulation of peptidase activity);GO:0009841(mitochondrial endopeptidase Clp complex);GO:0009368(endopeptidase Clp complex);GO:0005743(mitochondrial inner membrane)
circRNA381	GO:0005515(protein binding)
circRNA382	GO:0006508(proteolysis);GO:0008236(serine-type peptidase activity);GO:0016020(membrane);GO:0016787(hydrolase activity);GO:0005737(cytoplasm);GO:0005634(nucleus)
circRNA383	GO:0005737(cytoplasm);GO:0005634(nucleus);()
circRNA384	GO:0005515(protein binding);GO:0005739(mitochondrion);GO:0070062(extracellular vesicular exosome);GO:0005737(cytoplasm);GO:0005634(nucleus);GO:0016020(membrane);GO:0005829(cytosol);GO:0048471(perinuclear region of cytoplasm);GO:0043065(positive regulation of apoptotic
circRNA385	GO:0042981(regulation of apoptotic process);GO:0000166(nucleotide binding);GO:0003676(nucleic acid binding)
circRNA386	GO:0003824(catalytic activity);GO:0004553("hydrolase activity, hydrolyzing O-glycosyl compounds");GO:0005975(carbohydrate metabolic process);GO:0030246(carbohydrate binding);GO:0004558(alpha-g
circRNA387	GO:0000166(nucleotide binding);GO:0004672(protein kinase activity);GO:0005524(ATP binding);GO:0006468(protein phosphorylation);GO:0016772("transferase activity, transferring phosphorus-containing groups");GO:0004713(protein tyrosine kinase activity);GO:0005615(extracellular space);GO:0004674(protein serine/threonine kinase activity);GO:0005829(cytosol);GO:0005515(protein
circRNA388	()
circRNA389	()
circRNA39	GO:0016020(membrane);GO:0005886(plasma membrane);GO:0005794(Golgi apparatus);GO:1902237(positive regulation of intrinsic apoptotic signaling pathway in response to endoplasmic reticulum stress)
circRNA390	GO:0005515(protein binding);GO:0005737(cytoplasm)
circRNA391	GO:0046872(metal ion binding)
circRNA392	GO:0006886(intracellular protein transport);GO:0030904(retromer complex);GO:0035091(phosphatidylinositol binding);GO:0042147("retrograde transport, endosome to Golgi");GO:0043231(intracellular membrane-bounded organelle);GO:0005737(cytoplasm);GO:0005515(protein binding);GO:0010008(endosome membrane);GO:0031901(early endosome membrane);GO:0034498(early endosome to Golgi
circRNA393	GO:0004842(ubiquitin-protein transferase activity);GO:0008152(metabolic process);GO:0016567(protein ubiquitination);GO:0016874(ligase activity);GO:0005515(protein binding);GO:0031175(neuron projection development);GO:0050885(neuromuscular process controlling balance);GO:0021702(cerebellar Purkinje cell differentiation);GO:0010507(negative regulation of autophagy)
circRNA394	GO:0000166(nucleotide binding);GO:0003774(motor activity);GO:0005524(ATP binding);GO:0008152(metabolic process);GO:0016459(myosin complex);GO:0005515(protein binding);GO:0005856(cytoskeleton);GO:0070062(extracellular vesicular exosome);GO:0035091(phosphatidylinositol binding);GO:0006897(endocytosis);GO:0006200(ATP catabolic process);GO:0005516(calmodulin binding);GO:0051015(actin filament binding);GO:0042623("ATPase activity, coupled");GO:0045334(clathrin-coated endocytic vesicle);GO:0005737(cytoplasm);GO:0001570(vasculogenesis);GO:0001701(in utero embryonic development);GO:0005911(cell-cell junction);GO:0030097(hemopoiesis);GO:0005912(adherens junction);GO:0001822(kidney development);GO:0048008(platelet-derived growth factor receptor signaling pathway);GO:0035166(post-embryonic hemopoiesis);GO:0003094(glomerular filtration);GO:0006807(nitrogen compound metabolic process);GO:0032836(glomerular basement membrane development);GO:0072015(glomerular visceral epithelial cell development)
circRNA395	GO:0046872(metal ion binding)
circRNA396	()
circRNA397	GO:0004674(protein serine/threonine kinase activity);GO:0005216(ion channel activity);GO:0005524(ATP binding);GO:0006468(protein phosphorylation);GO:0006810(transport);GO:0006811(ion transport);GO:0016020(membrane);GO:0016021(integral component of membrane);GO:0016772("transferase activity, transferring phosphorus-containing groups");GO:0034220(ion transmembrane transport);GO:0055085(transmembrane transport);GO:0070266(necroptotic process);GO:0005886(plasma membrane);GO:0005515(protein binding);GO:0046777(protein autophosphorylation);GO:0005262(calcium channel activity);GO:0006816(calcium ion transport);GO:0017022(myosin binding);GO:0003779(actin binding);GO:0001726(ruffle);GO:0016301(kinase activity);GO:0016340(calcium-dependent cell-matrix adhesion);GO:0031032(actomyosin structure organization)
circRNA398	GO:0000166(nucleotide binding);GO:0004335(galactokinase activity);GO:0005524(ATP binding);GO:0005737(cytoplasm);GO:0006012(galactose metabolic process);GO:0008152(metabolic process);GO:0016301(kinase activity);GO:0016310(phosphorylation);GO:0016740(transferase activity);GO:0016773("phosphotransferase activity, alcohol group as acceptor");GO:0046835(carbohydrate
circRNA399	GO:0007018(microtubule-based movement);GO:0019894(kinesin binding);GO:0005783(endoplasmic reticulum);GO:0016020(membrane);GO:0044822(poly(A) RNA binding)
circRNA4	GO:0000166(nucleotide binding);GO:0003676(nucleic acid binding);GO:0004386(helicase activity);GO:0005524(ATP binding);GO:0006200(ATP catabolic process);GO:0008026(ATP-dependent helicase activity);GO:0008152(metabolic process);GO:0016787(hydrolase activity);GO:0030308(negative regulation of cell growth);GO:0003677(DNA binding);GO:0070062(extracellular vesicular
circRNA40	GO:0008270(zinc ion binding);GO:0046872(metal ion binding);GO:0005515(protein binding)
circRNA400	GO:0008152(metabolic process);GO:0016491(oxidoreductase activity);GO:0055114(oxidation-reduction process);GO:0016020(membrane)

circRNA401	GO:0003824(catalytic activity);GO:0004329(formate-tetrahydrofolate ligase activity);GO:0004488(methylenetetrahydrofolate dehydrogenase (NADP+) activity);GO:0005524(ATP binding);GO:0009396(folic acid-containing compound biosynthetic process);GO:0055114(oxidation-reduction process);GO:0070062(extracellular vesicular
circRNA402	GO:0000030(mannosyltransferase activity);GO:0006493(protein O-linked glycosylation);GO:0016020(membrane);GO:0097502(mannosylation)
circRNA403	GO:0003824(catalytic activity);GO:0008152(metabolic process);GO:0009058(biosynthetic process);GO:0016740(transferase activity);GO:0030170(pyridoxal phosphate binding);GO:0017059(serine C-palmitoyltransferase complex);GO:0004758(serine C-palmitoyltransferase activity);GO:0005739(mitochondrion);GO:0046513(ceramide biosynthetic process);GO:0046512(sphingosine biosynthetic
circRNA404	()
circRNA405	GO:0005515(protein binding)
circRNA406	GO:0005515(protein binding)
circRNA407	GO:0005515(protein binding)
circRNA408	GO:0006810(transport);GO:0006886(intracellular protein transport);GO:0006888(ER to Golgi vesicle-mediated transport);GO:0008270(zinc ion binding);GO:0015031(protein transport);GO:0030127(COPII vesicle coat);GO:0001701(in utero embryonic development)
circRNA409	GO:0007165(signal transduction);GO:0005515(protein binding);GO:0005622(intracellular);GO:0010628(positive regulation of gene expression);GO:0016323(basolateral plasma membrane);GO:0030507(spectrin binding);GO:0051924(regulation of calcium ion transport);GO:0019899(enzyme binding);GO:0055117(regulation of cardiac muscle contraction);GO:0019901(protein kinase binding);GO:0086070(SA node cell to atrial cardiac muscle cell communication);GO:0070972(protein localization to endoplasmic reticulum);GO:0010882(regulation of cardiac muscle contraction by calcium ion signaling);GO:0033365(protein localization to organelle);GO:0003283(atrial septum development);GO:0086004(regulation of cardiac muscle cell contraction);GO:0086014(atrial cardiac muscle cell action potential);GO:0060307(regulation of ventricular cardiac muscle cell membrane repolarization);GO:0086005(ventricular cardiac muscle cell action potential);GO:0051279(regulation of release of sequestered calcium ion into cytosol);GO:0034613(cellular protein localization);GO:0010881(regulation of cardiac muscle contraction by regulation of the release of sequestered calcium ion);GO:0002027(regulation of heart rate);GO:0005886(plasma membrane);GO:0005887(integral component of plasma membrane);GO:0005737(cytoplasm);GO:0072659(protein localization to plasma membrane);GO:0030018(Z disc);GO:0030315(T-tubule);GO:0051117(ATPase binding);GO:0048471(perinuclear region of cytoplasm);GO:0031672(A band);GO:0060048(cardiac muscle contraction);GO:0050821(protein stabilization);GO:0030674("protein binding, bridging");GO:0042383(sarcolemma);GO:0031430(M band);GO:0015459(potassium channel regulator activity);GO:0006874(cellular calcium ion homeostasis);GO:0043268(positive regulation of potassium ion transport);GO:0044325(ion channel binding);GO:0014704(intercalated disc);GO:0008104(protein localization);GO:0072661(protein targeting to plasma membrane);GO:0043034(costamere);GO:0034394(protein localization to cell surface);GO:0051928(positive regulation of calcium ion transport);GO:0086091(regulation of heart rate by cardiac conduction);GO:2001259(positive regulation of cation channel activity);GO:1901019(regulation of calcium ion transmembrane transporter activity);GO:0086015(SA node cell action potential);GO:0033292(T-tubule organization);GO:0036309(protein localization to M-band);GO:0036371(protein localization to T-tubule);GO:0086036(regulation of cardiac muscle cell membrane potential);GO:0086066(atrial cardiac muscle cell to AV node cell communication);GO:1901018(positive regulation of potassium ion transmembrane transporter activity);GO:1901021(positive regulation of calcium ion transmembrane transporter activity);GO:2001257(regulation of cation channel activity)
circRNA41	GO:0005086(ARF guanyl-nucleotide exchange factor activity);GO:0032012(regulation of ARF protein signal transduction);GO:0043547(positive regulation of GTPase activity);GO:0005488(binding);GO:0005085(guanyl-nucleotide exchange factor activity);GO:0000139(Golgi membrane);GO:0016020(membrane);GO:0005829(cytosol);GO:0005515(protein binding);GO:0005815(microtubule organizing center);GO:0035556(intracellular signal transduction);GO:0055037(recycling endosome);GO:0032760(positive regulation of tumor necrosis factor production);GO:0006893(Golgi to plasma membrane transport);GO:0034237(protein kinase A regulatory subunit binding);GO:0010256(endomembrane system organization);GO:0001881(receptor recycling);GO:0007032(endosome organization)
circRNA410	GO:0003857(3-hydroxyacyl-CoA dehydrogenase activity);GO:0006631(fatty acid metabolic process);GO:0016491(oxidoreductase activity);GO:0016616("oxidoreductase activity, acting on the CH-OH group of donors, NAD or NADP as acceptor");GO:0050662(coenzyme binding);GO:0055114(oxidation-reduction process);GO:0070403(NAD+ binding);GO:0050660(flavin adenine dinucleotide binding);GO:0051287(NAD binding);GO:0046168(glycerol-3-phosphate catabolic process);GO:0005737(cytoplasm);GO:0005739(mitochondrion);GO:0005634(nucleus);GO:0005743(mitochondrial inner membrane)
circRNA411	GO:0003857(3-hydroxyacyl-CoA dehydrogenase activity);GO:0006631(fatty acid metabolic process);GO:0016491(oxidoreductase activity);GO:0016616("oxidoreductase activity, acting on the CH-OH group of donors, NAD or NADP as acceptor");GO:0050662(coenzyme binding);GO:0055114(oxidation-reduction process);GO:0070403(NAD+ binding);GO:0050660(flavin adenine dinucleotide binding);GO:0051287(NAD binding);GO:0046168(glycerol-3-phosphate catabolic process);GO:0005737(cytoplasm);GO:0005739(mitochondrion);GO:0005634(nucleus);GO:0005743(mitochondrial inner membrane)
circRNA412	GO:0003857(3-hydroxyacyl-CoA dehydrogenase activity);GO:0006631(fatty acid metabolic process);GO:0016491(oxidoreductase activity);GO:0016616("oxidoreductase activity, acting on the CH-OH group of donors, NAD or NADP as acceptor");GO:0050662(coenzyme binding);GO:0055114(oxidation-reduction process);GO:0070403(NAD+ binding);GO:0050660(flavin adenine dinucleotide binding);GO:0051287(NAD binding);GO:0046168(glycerol-3-phosphate catabolic process);GO:0005737(cytoplasm);GO:0005739(mitochondrion);GO:0005634(nucleus);GO:0005743(mitochondrial inner membrane)
circRNA413	()
circRNA414	GO:0003676(nucleic acid binding);GO:0008168(methyltransferase activity);GO:0008270(zinc ion binding);GO:0032259(methylation)
circRNA415	GO:0005515(protein binding);GO:0090305(nucleic acid phosphodiester bond hydrolysis);GO:0004519(endonuclease activity);GO:0005524(ATP binding);GO:0016310(phosphorylation);GO:0046404(ATP-dependent polydeoxyribonucleotide 5'-hydroxyl-kinase activity);GO:0005829(cytosol)
circRNA416	GO:0005515(protein binding);GO:0005737(cytoplasm);GO:0005634(nucleus);GO:0006355("regulation of transcription, DNA-templated");GO:0001764(neuron migration);GO:0007411(axon guidance);GO:0043066(negative regulation of apoptotic process);GO:0030198(extracellular matrix organization);GO:0043065(positive regulation of apoptotic process);GO:0030308(negative regulation of cell growth);GO:0008134(transcription factor binding);GO:0007050(cell cycle arrest)
circRNA417	GO:0008270(zinc ion binding);GO:0046872(metal ion binding);GO:0005515(protein binding)
circRNA418	GO:0000166(nucleotide binding);GO:0000287(magnesium ion binding);GO:0004012(phospholipid-translocating ATPase activity);GO:0005524(ATP binding);GO:0006812(cation transport);GO:0008152(metabolic process);GO:0015914(phospholipid transport);GO:0016020(membrane);GO:0016021(integral component of membrane);GO:0016787(hydrolase activity);GO:0019829(cation-transporting ATPase activity);GO:0045332(phospholipid translocation);GO:0046872(metal ion binding);GO:0005783(endoplasmic reticulum);GO:0005515(protein
circRNA419	GO:0003824(catalytic activity);GO:0004252(serine-type endopeptidase activity);GO:0006508(proteolysis);GO:0016020(membrane);GO:0070008(serine-type exopeptidase activity);GO:0005515(protein binding);GO:0005887(integral component of plasma membrane);GO:0007565(female pregnancy);GO:0016486(peptide hormone processing);GO:0009986(cell surface);GO:0008217(regulation of blood pressure);GO:0035813(regulation of renal sodium excretion);GO:0003050(regulation of systemic arterial blood pressure by atrial natriuretic peptide)
circRNA42	GO:0070062(extracellular vesicular exosome);GO:0016020(membrane);GO:0005737(cytoplasm);GO:0005515(protein binding);GO:0044822(poly(A) RNA binding);GO:0030425(dendrite);GO:0036464(cytoplasmic ribonucleoprotein granule);GO:0044297(cell body);GO:0030529(ribonucleoprotein complex);GO:0003723(RNA
circRNA420	GO:0007517(muscle organ development);GO:0016012(sarcoglycan complex);GO:0016021(integral component of membrane);GO:0016010(dystrophin-associated glycoprotein complex);GO:0042383(sarcolemma);GO:0048747(muscle fiber development);GO:0016011(dystroglycan complex);GO:0055013(cardiac muscle cell development);GO:0097084(vascular smooth muscle cell
circRNA421	GO:0003824(catalytic activity);GO:0004488(methylenetetrahydrofolate dehydrogenase (NADP+) activity);GO:0009396(folic acid-containing compound biosynthetic process);GO:0055114(oxidation-reduction
circRNA422	GO:0000139(Golgi membrane);GO:0005737(cytoplasm);GO:0006886(intracellular protein transport);GO:0008565(protein transporter activity);GO:0016020(membrane);GO:0048193(Golgi vesicle transport);GO:0048280(vesicle fusion with Golgi apparatus);GO:0005515(protein binding);GO:0005488(binding);GO:0005730(nucleolus);GO:0044822(poly(A) RNA binding);GO:0005794(Golgi apparatus);GO:0005829(cytosol);GO:0048471(perinuclear region of cytoplasm)
circRNA423	GO:0000139(Golgi membrane);GO:0005737(cytoplasm);GO:0006886(intracellular protein transport);GO:0008565(protein transporter activity);GO:0016020(membrane);GO:0048193(Golgi vesicle transport);GO:0048280(vesicle fusion with Golgi apparatus);GO:0005515(protein binding);GO:0005488(binding);GO:0005730(nucleolus);GO:0044822(poly(A) RNA binding);GO:0005794(Golgi apparatus);GO:0005829(cytosol);GO:0048471(perinuclear region of cytoplasm)
circRNA424	GO:0005515(protein binding);GO:0005737(cytoplasm);GO:0005634(nucleus);GO:0030014(CCR4-NOT complex);GO:0010606(positive regulation of cytoplasmic mRNA processing body assembly);GO:0008284(positive regulation of cell proliferation);GO:0004535(poly(A)-specific ribonuclease activity);GO:0090503("RNA phosphodiester bond hydrolysis, exonucleolytic");GO:0000288("nuclear-transcribed mRNA catabolic process, deadenylation-dependent decay");GO:0061157(mRNA destabilization)
circRNA425	GO:0007154(cell communication);GO:0016021(integral component of membrane);GO:0005515(protein binding);GO:0043588(skin development);GO:0015031(protein transport);GO:0005604(basement membrane);GO:0060021(palate development);GO:0030326(embryonic limb morphogenesis);GO:0002009(morphogenesis of an epithelium);GO:0003338(metanephros
circRNA426	GO:0007154(cell communication);GO:0016021(integral component of membrane);GO:0005515(protein binding);GO:0043588(skin development);GO:0015031(protein transport);GO:0005604(basement membrane);GO:0060021(palate development);GO:0030326(embryonic limb morphogenesis);GO:0002009(morphogenesis of an epithelium);GO:0003338(metanephros
circRNA427	GO:0004725(protein tyrosine phosphatase activity);GO:0005856(cytoskeleton);GO:0006470(protein dephosphorylation);GO:0016311(dephosphorylation);GO:0016791(phosphatase activity);GO:0035335(peptidyl-tyrosine dephosphorylation);GO:0005515(protein binding);GO:0070062(extracellular vesicular exosome);GO:0005737(cytoplasm);GO:0005886(plasma
circRNA428	GO:0005089(Rho guanyl-nucleotide exchange factor activity);GO:0032321(positive regulation of Rho GTPase activity);GO:0035023(regulation of Rho protein signal transduction);GO:0005515(protein bindir
circRNA429	GO:0005089(Rho guanyl-nucleotide exchange factor activity);GO:0032321(positive regulation of Rho GTPase activity);GO:0035023(regulation of Rho protein signal transduction);GO:0005515(protein bindir
circRNA43	GO:0008152(metabolic process);GO:0016881(acid-amino acid ligase activity)
circRNA430	GO:0005089(Rho guanyl-nucleotide exchange factor activity);GO:0032321(positive regulation of Rho GTPase activity);GO:0035023(regulation of Rho protein signal transduction);GO:0005515(protein bindir
circRNA431	GO:0005089(Rho guanyl-nucleotide exchange factor activity);GO:0032321(positive regulation of Rho GTPase activity);GO:0035023(regulation of Rho protein signal transduction);GO:0005515(protein bindir
circRNA432	GO:0005089(Rho guanyl-nucleotide exchange factor activity);GO:0032321(positive regulation of Rho GTPase activity);GO:0035023(regulation of Rho protein signal transduction);GO:0005515(protein bindir
circRNA433	GO:0005089(Rho guanyl-nucleotide exchange factor activity);GO:0032321(positive regulation of Rho GTPase activity);GO:0035023(regulation of Rho protein signal transduction);GO:0005515(protein bindir

circRNA434	GO:0005089(Rho guanyl-nucleotide exchange factor activity);GO:0032321(positive regulation of Rho GTPase activity);GO:0035023(regulation of Rho protein signal transduction);GO:0005515(protein bindin
circRNA435	GO:0005089(Rho guanyl-nucleotide exchange factor activity);GO:0032321(positive regulation of Rho GTPase activity);GO:0035023(regulation of Rho protein signal transduction);GO:0005515(protein bindin
circRNA436	GO:0005509(calcium ion binding);GO:0005515(protein binding);GO:0016020(membrane);GO:0030132(clathrin coat of coated pit)
circRNA437	GO:0000166(nucleotide binding);GO:0004672(protein kinase activity);GO:0004674(protein serine/threonine kinase activity);GO:0005524(ATP binding);GO:0006468(protein phosphorylation);GO:0007165(signal transduction);GO:0016301(kinase activity);GO:0016310(phosphorylation);GO:0016740(transferase activity);GO:0016772("transferase activity, transferring
circRNA438	GO:0003677(DNA binding);GO:0005634(nucleus);GO:0006355("regulation of transcription, DNA-templated");GO:0005515(protein binding)
circRNA439	GO:0003824(catalytic activity);GO:0004114("3',5'-cyclic-nucleotide phosphodiesterase activity");GO:0007165(signal transduction);GO:0008081(phosphoric diester hydrolase activity);GO:0008152(metabolic process);GO:0016787(hydrolase activity);GO:0046872(metal ion binding);GO:0048471(perinuclear region of cytoplasm);GO:0005515(protein binding);GO:0030552(cAMP binding);GO:0004115("3',5'-cyclic-AMP phosphodiesterase activity");GO:0005737(cytoplasm);GO:0007608(sensory perception of smell);GO:0035690(cellular response to drug);GO:0010738(regulation of protein kinase A signaling);GO:0043949(regulation of cAMP-mediated signaling)
circRNA44	GO:0005634(nucleus);GO:0008270(zinc ion binding);GO:0043066(negative regulation of apoptotic process);GO:0046872(metal ion binding);GO:0071157(negative regulation of cell cycle arrest);GO:000551
circRNA440	GO:0000166(nucleotide binding);GO:0004672(protein kinase activity);GO:0004713(protein tyrosine kinase activity);GO:0004714(transmembrane receptor protein tyrosine kinase activity);GO:0005524(ATP binding);GO:0006468(protein phosphorylation);GO:0007169(transmembrane receptor protein tyrosine kinase signaling pathway);GO:0016020(membrane);GO:0016021(integral component of membrane);GO:0016301(kinase activity);GO:0016310(phosphorylation);GO:0016740(transferase activity);GO:0016772("transferase activity, transferring phosphorus-containing groups");GO:0018108(peptidyl-tyrosine phosphorylation);GO:0043548(phosphatidylinositol 3-kinase binding);GO:0043560(insulin receptor substrate binding);GO:0046777(protein autophosphorylation);GO:0005515(protein binding);GO:0006355("regulation of transcription, DNA-templated");GO:0007186(G-protein coupled receptor signaling pathway);GO:0005525(GTP binding);GO:0070062(extracellular vesicular exosome);GO:0005887(integral component of plasma membrane);GO:0003007(heart morphogenesis);GO:0045821(positive regulation of glycolytic process);GO:0043235(receptor complex);GO:0051897(positive regulation of protein kinase B signaling);GO:0000187(activation of MAPK activity);GO:0001934(positive regulation of protein phosphorylation);GO:0045995(regulation of embryonic development);GO:0005159(insulin-like growth factor receptor binding);GO:0043410(positive regulation of MAPK cascade);GO:0004716(receptor signaling protein tyrosine kinase activity);GO:0005901(caveola);GO:0032869(cellular response to insulin stimulus);GO:0023014(signal transduction by phosphorylation);GO:0030335(positive regulation of cell migration);GO:0008286(insulin receptor signaling pathway);GO:0048639(positive regulation of developmental growth);GO:0043559(insulin binding);GO:0031995(insulin-like growth factor II binding);GO:0031994(insulin-like growth factor I binding);GO:0019087(transformation of host cell by virus);GO:0005899(insulin receptor complex);GO:0005009(insulin-activated receptor activity);GO:0051425(PTB domain binding);GO:0060267(positive regulation of respiratory burst);GO:0045725(positive regulation of glycogen biosynthetic process);GO:0051290(protein heterotetramerization);GO:0042593(glucose homeostasis);GO:0045429(positive regulation of nitric oxide biosynthetic process);GO:0032147(activation of protein kinase activity);GO:0045840(positive regulation of mitosis);GO:0045740(positive regulation of DNA replication);GO:0032148(activation of protein kinase B activity);GO:0005886(plasma membrane);GO:0009887(organ morphogenesis);GO:0008544(epidermis development);GO:0038083(peptidyl-tyrosine autophosphorylation);GO:0071363(cellular response to growth factor stimulus);GO:0030238(male sex determination);GO:0031017(exocrine pancreas development)
circRNA441	GO:0000166(nucleotide binding);GO:0004672(protein kinase activity);GO:0004713(protein tyrosine kinase activity);GO:0004714(transmembrane receptor protein tyrosine kinase activity);GO:0005524(ATP binding);GO:0006468(protein phosphorylation);GO:0007169(transmembrane receptor protein tyrosine kinase signaling pathway);GO:0016020(membrane);GO:0016021(integral component of membrane);GO:0016301(kinase activity);GO:0016310(phosphorylation);GO:0016740(transferase activity);GO:0016772("transferase activity, transferring phosphorus-containing groups");GO:0018108(peptidyl-tyrosine phosphorylation);GO:0043548(phosphatidylinositol 3-kinase binding);GO:0043560(insulin receptor substrate binding);GO:0046777(protein autophosphorylation);GO:0005515(protein binding);GO:0006355("regulation of transcription, DNA-templated");GO:0007186(G-protein coupled receptor signaling pathway);GO:0005525(GTP binding);GO:0070062(extracellular vesicular exosome);GO:0005887(integral component of plasma membrane);GO:0003007(heart morphogenesis);GO:0045821(positive regulation of glycolytic process);GO:0043235(receptor complex);GO:0051897(positive regulation of protein kinase B signaling);GO:0000187(activation of MAPK activity);GO:0001934(positive regulation of protein phosphorylation);GO:0045995(regulation of embryonic development);GO:0005159(insulin-like growth factor receptor binding);GO:0043410(positive regulation of MAPK cascade);GO:0004716(receptor signaling protein tyrosine kinase activity);GO:0005901(caveola);GO:0032869(cellular response to insulin stimulus);GO:0023014(signal transduction by phosphorylation);GO:0030335(positive regulation of cell migration);GO:0008286(insulin receptor signaling pathway);GO:0048639(positive regulation of developmental growth);GO:0043559(insulin binding);GO:0031995(insulin-like growth factor II binding);GO:0031994(insulin-like growth factor I binding);GO:0019087(transformation of host cell by virus);GO:0005899(insulin receptor complex);GO:0005009(insulin-activated receptor activity);GO:0051425(PTB domain binding);GO:0060267(positive regulation of respiratory burst);GO:0045725(positive regulation of glycogen biosynthetic process);GO:0051290(protein heterotetramerization);GO:0042593(glucose homeostasis);GO:0045429(positive regulation of nitric oxide biosynthetic process);GO:0032147(activation of protein kinase activity);GO:0045840(positive regulation of mitosis);GO:0045740(positive regulation of DNA replication);GO:0032148(activation of protein kinase B activity);GO:0005886(plasma membrane);GO:0009887(organ morphogenesis);GO:0008544(epidermis development);GO:0038083(peptidyl-tyrosine autophosphorylation);GO:0071363(cellular response to growth factor stimulus);GO:0030238(male sex determination);GO:0031017(exocrine pancreas development)
circRNA442	GO:0000166(nucleotide binding);GO:0003676(nucleic acid binding)
circRNA443	GO:0000166(nucleotide binding);GO:0003777(microtubule motor activity);GO:0005524(ATP binding);GO:0005871(kinesin complex);GO:0005874(microtubule);GO:0007018(microtubule-based movement);GO:0008017(microtubule binding);GO:0008152(metabolic process);GO:0005515(protein binding);GO:0007224(smoothened signaling pathway);GO:0009952(anterior/posterior pattern specification);GO:0009953(dorsal/ventral pattern formation);GO:0001701(in utero embryonic development);GO:0043005(neuron projection);GO:0042384(cilium assembly);GO:0007507(heart development);GO:0007368(determination of left/right symmetry);GO:0005929(cilium);GO:0008544(epidermis development);GO:0050679(positive regulation of epithelial cell proliferation);GO:0001822(kidney development);GO:0032391(photoreceptor connecting cilium);GO:0021904(dorsal/ventral neural tube patterning);GO:0072372(primary cilium);GO:0060271(cilium morphogenesis);GO:0036334(epidermal stem cell homeostasis);GO:0060122(inner ear receptor stereocilium organization);GO:0030990(intraciliary transport particle)
circRNA444	GO:0005215(transporter activity);GO:0006810(transport);GO:0006811(ion transport);GO:0006821(chloride transport);GO:0015377(cation:chloride symporter activity);GO:0016020(membrane);GO:0016021(integral component of membrane);GO:0055085(transmembrane transport);GO:1902476(chloride transmembrane transport);GO:0070062(extracellular vesicular exosome);GO:0005515(protein binding);GO:0072488(ammonium transmembrane transport);GO:0008519(ammonium transmembrane transporter activity);GO:0070634(transepithelial ammonium transport);GO:0015696(ammonium transport);GO:0030321(transepithelial chloride transport);GO:0035264(multicellular organism growth);GO:0060444(branching involved in mammary gland duct morphogenesis);GO:0016323(basolateral plasma membrane);GO:0016324(apical plasma membrane);GO:0050910(detection of mechanical stimulus involved in sensory perception of sound);GO:0060763(mammary duct terminal end bud growth)
circRNA445	GO:0005215(transporter activity);GO:0006810(transport);GO:0006811(ion transport);GO:0006821(chloride transport);GO:0015377(cation:chloride symporter activity);GO:0016020(membrane);GO:0016021(integral component of membrane);GO:0055085(transmembrane transport);GO:1902476(chloride transmembrane transport);GO:0070062(extracellular vesicular exosome);GO:0005515(protein binding);GO:0072488(ammonium transmembrane transport);GO:0008519(ammonium transmembrane transporter activity);GO:0070634(transepithelial ammonium transport);GO:0015696(ammonium transport);GO:0030321(transepithelial chloride transport);GO:0035264(multicellular organism growth);GO:0060444(branching involved in mammary gland duct morphogenesis);GO:0016323(basolateral plasma membrane);GO:0016324(apical plasma membrane);GO:0050910(detection of mechanical stimulus involved in sensory perception of sound);GO:0060763(mammary duct terminal end bud growth)
circRNA446	()
circRNA447	NA
circRNA448	GO:0046872(metal ion binding)
circRNA449	GO:0005515(protein binding);GO:0005813(centrosome);GO:0008022(protein C-terminus binding);GO:0000226(microtubule cytoskeleton organization);GO:0008283(cell proliferation);GO:0022008(neurogenesis);GO:0032880(regulation of protein localization);GO:0021987(cerebral cortex development);GO:0022027(interkinetic nuclear migration);GO:0030953(astral microtubule organization);GO:0032886(regulation of microtubule-based process)
circRNA45	GO:0005576(extracellular region);GO:0008083(growth factor activity);GO:0005160(transforming growth factor beta receptor binding);GO:0005615(extracellular space);GO:0016049(cell growth);GO:0040007(growth);GO:0030308(negative regulation of cell growth);GO:0007507(heart development);GO:0001654(eye development);GO:0031012(extracellular matrix);GO:0006468(protein phosphorylation);GO:0005515(protein binding);GO:0008285(negative regulation of cell proliferation);GO:0001540(beta-amyloid binding);GO:0008284(positive regulation of cell proliferation);GO:0042803(protein homodimerization activity);GO:0005102(receptor binding);GO:0007050(cell cycle arrest);GO:0001666(response to hypoxia);GO:0000902(cell morphogenesis);GO:0003007(heart morphogenesis);GO:0033630(positive regulation of cell adhesion mediated by integrin);GO:0050714(positive regulation of protein secretion);GO:0009611(response to wounding);GO:0097191(extrinsic apoptotic signaling pathway);GO:0014068(positive regulation of phosphatidylinositol 3-kinase signaling);GO:0030307(positive regulation of cell growth);GO:0008219(cell death);GO:0007179(transforming growth factor beta receptor signaling pathway);GO:0023014(signal transduction by phosphorylation);GO:0004702(receptor signaling protein serine/threonine kinase activity);GO:0016477(cell migration);GO:0042637(catagen);GO:0032909(regulation of transforming growth factor beta2 production);GO:0010693(negative regulation of alkaline phosphatase activity);GO:0045823(positive regulation of heart contraction);GO:0051891(positive regulation of cardioblast differentiation);GO:0051795(positive regulation of catagen);GO:0008347(glial cell migration);GO:0060317(cardiac epithelial to mesenchymal transition);GO:0045778(positive regulation of ossification);GO:0022601(menstrual cycle phase);GO:0045726(positive regulation of integrin biosynthetic process);GO:0048566(embryonic digestive tract development);GO:0032570(response to progesterone);GO:0010936(negative regulation of macrophage cytokine production);GO:0007435(salivary gland morphogenesis);GO:0005114(type II transforming growth factor beta receptor binding);GO:0007184(SMAD protein import into nucleus);GO:0060038(cardiac muscle cell proliferation);GO:0042493(response to drug);GO:0045216(cell-cell junction organization);GO:0010002(cardioblast differentiation);GO:0001837(epithelial to mesenchymal transition);GO:0043525(positive regulation of neuron apoptotic process);GO:0010718(positive regulation of epithelial to mesenchymal transition);GO:0010634(positive regulation of epithelial cell migration);GO:0032874(positive regulation of stress-activated MAPK cascade);GO:0032147(activation of protein kinase activity);GO:0060389(pathway-restricted SMAD protein phosphorylation);GO:0030199(collagen fibril organization);GO:0050680(negative regulation of epithelial cell proliferation);GO:0005768(endosome);GO:0042981(regulation of apoptotic process);GO:0001568(blood vessel development);GO:0007411(axon guidance);GO:0043025(neuronal cell body);GO:0001501(skeletal system development);GO:0030198(extracellular matrix organization);GO:0030097(hemopoiesis);GO:0030424(axon);GO:0001974(blood vessel remodeling);GO:0031069(hair follicle morphogenesis);GO:0045787(positive regulation of cell cycle);GO:0048103(somatic stem cell division);GO:0048663(neuron fate commitment);GO:0060325(face morphogenesis);GO:0010628(positive regulation of gene expression);GO:0042416(dopamine biosynthetic process);GO:2001241(positive regulation of extrinsic apoptotic signaling pathway in absence of ligand);GO:0001502(cartilage condensation);GO:0048666(neuron development);GO:0070237(positive regulation of activation-induced cell death of T cells)
circRNA450	GO:0005515(protein binding);GO:0005813(centrosome);GO:0008022(protein C-terminus binding);GO:0000226(microtubule cytoskeleton organization);GO:0008283(cell proliferation);GO:0022008(neurogenesis);GO:0032880(regulation of protein localization);GO:0021987(cerebral cortex development);GO:0022027(interkinetic nuclear migration);GO:0030953(astral microtubule organization);GO:0032886(regulation of microtubule-based process)

circRNA451	GO:0008152(metabolic process);GO:0016491(oxidoreductase activity);GO:0055114(oxidation-reduction process);GO:0043231(intracellular membrane-bounded organelle);GO:0016020(membrane);GO:0005777(peroxisome);GO:0005778(peroxisomal membrane);GO:0042803(protein homodimerization activity);GO:0005102(receptor binding);GO:0003857(3-hydroxyacyl-CoA dehydrogenase activity);GO:0006635(fatty acid beta-oxidation);GO:0044594(17-beta-hydroxysteroid dehydrogenase (NAD+) activity);GO:0036112(medium-chain fatty-acyl-CoA metabolic process);GO:0036111(very long-chain fatty-acyl-CoA metabolic process);GO:0016508(long-chain-enoyl-CoA hydratase activity);GO:0008209(androgen metabolic process);GO:0008210(estrogen metabolic process);GO:0001649(osteoblast differentiation);GO:0005739(mitochondrion);GO:0000038(very long-chain fatty acid metabolic process);GO:0060009(Sertoli cell development)
circRNA452	GO:0000166(nucleotide binding);GO:0003676(nucleic acid binding);GO:0003723(RNA binding);GO:0005634(nucleus);GO:0006397(mRNA processing);GO:0070062(extracellular vesicular exosome);GO:0016020(membrane);GO:0005515(protein binding);GO:0044822(poly(A) RNA binding);GO:0048025("negative regulation of mRNA splicing, via spliceosome");GO:0000381("regulation of alternative mRNA splicing, via spliceosome");GO:0051148(negative regulation of muscle cell differentiation);GO:0036002(pre-mRNA binding);GO:0033119(negative regulation of RNA splicing)
circRNA453	GO:0005622(intracellular);GO:0006355("regulation of transcription, DNA-templated");GO:0003700(sequence-specific DNA binding transcription factor activity);GO:0005634(nucleus);GO:0005667(transcription factor complex);GO:0006351("transcription, DNA-templated");GO:0007179(transforming growth factor beta receptor signaling pathway);GO:0005515(protein binding);GO:0031625(ubiquitin protein ligase binding);GO:0043234(protein complex);GO:0006468(protein phosphorylation);GO:0005737(cytoplasm);GO:0045944(positive regulation of transcription from RNA polymerase II promoter);GO:0007281(germ cell development);GO:0000979(RNA polymerase II core promoter sequence-specific DNA binding);GO:0045669(positive regulation of osteoblast differentiation);GO:0051216(cartilage development);GO:0030218(erythrocyte differentiation);GO:0060048(cardiac muscle contraction);GO:0060348(bone development);GO:0071407(cellular response to organic cyclic compound);GO:0001657(ureteric bud development);GO:0071773(cellular response to BMP stimulus);GO:0002051(osteoblast fate commitment);GO:1901522(positive regulation of transcription from RNA polymerase II promoter involved in cellular response to chemical stimulus)
circRNA454	GO:0005515(protein binding)
circRNA455	GO:0005622(intracellular);GO:0007165(signal transduction)
circRNA456	GO:0005680(anaphase-promoting complex);GO:0030071(regulation of mitotic metaphase/anaphase transition);GO:0005515(protein binding);GO:0005622(intracellular);GO:0007067(mitotic nuclear division);GO:0070979(protein K11-linked ubiquitination);GO:0000080(mitotic G1 phase);GO:0007080(mitotic metaphase plate congression)
circRNA457	GO:0005096(GTPase activator activity);GO:0005622(intracellular);GO:0007165(signal transduction);GO:0008093(cytoskeletal adaptor activity);GO:0017124(SH3 domain binding);GO:0043547(positive regulation of GTPase activity);GO:0046847(filopodium assembly);GO:0005515(protein binding);GO:0005543(phospholipid binding)
circRNA458	GO:0008270(zinc ion binding);GO:0046872(metal ion binding);GO:0005515(protein binding);GO:0019902(phosphatase binding);GO:0010923(negative regulation of phosphatase activity);GO:0008157(protein phosphatase 1 binding)
circRNA459	GO:0005737(cytoplasm);GO:0006208(pyrimidine nucleobase catabolic process);GO:0016787(hydrolase activity);GO:0016810("hydrolase activity, acting on carbon-nitrogen (but not peptide) bonds");GO:0016812("hydrolase activity, acting on carbon-nitrogen (but not peptide) bonds, in cyclic amides");GO:0007399(nervous system development);GO:0051219(phosphoprotein binding)
circRNA46	NA
circRNA460	GO:0003779(actin binding);GO:0006351("transcription, DNA-templated");GO:0008270(zinc ion binding);GO:0030036(actin cytoskeleton organization);GO:0046872(metal ion binding);GO:0045944(positive regulation of transcription from RNA polymerase II promoter)
circRNA461	GO:0000166(nucleotide binding);GO:0003676(nucleic acid binding);GO:0006355("regulation of transcription, DNA-templated");GO:0006366(transcription from RNA polymerase II promoter);GO:0001104(RNA polymerase II transcription cofactor activity);GO:0045944(positive regulation of transcription from RNA polymerase II promoter);GO:0005634(nucleus);GO:0030331(estrogen receptor binding);GO:0016592(mediator complex);GO:0030520(intracellular estrogen receptor signaling pathway);GO:0030374(ligand-dependent nuclear receptor transcription coactivator activity);GO:0050682(AF-2 domain binding);GO:0030546(receptor activator activity);GO:2000273(positive regulation of receptor activity);GO:0005515(protein binding);GO:0045892("negative regulation of transcription, DNA-templated");GO:0005739(mitochondrion);GO:0045893("positive regulation of transcription, DNA-templated");GO:0001503(ossification);GO:0045672(positive regulation of osteoclast differentiation);GO:0007015(actin filament organization);GO:0034614(cellular response to reactive oxygen species);GO:0042327(positive regulation of phosphorylation);GO:0010694(positive regulation of alkaline phosphatase activity);GO:0006390(transcription from mitochondrial promoter);GO:0045780(positive regulation of bone resorption);GO:0060346(bone trabecula formation)
circRNA462	GO:0008146(sulfotransferase activity);GO:0008152(metabolic process);GO:0015016([heparan sulfate]-glucosamine N-sulfotransferase activity);GO:0016787(hydrolase activity);GO:0007224(smoothened signaling pathway);GO:0030900(forebrain development);GO:0007585(respiratory gaseous exchange);GO:0000165(MAPK cascade);GO:0009887(organ morphogenesis);GO:0030901(midbrain development);GO:0008543(fibroblast growth factor receptor signaling pathway);GO:0048702(embryonic neurocranium morphogenesis);GO:0048703(embryonic viscerocranium morphogenesis);GO:0019213(deacetylase activity);GO:0030203(glycosaminoglycan metabolic process);GO:0000271(polysaccharide biosynthetic process)
circRNA463	GO:0005515(protein binding);GO:0070373(negative regulation of ERK1 and ERK2 cascade);GO:0004843(ubiquitin-specific protease activity);GO:0085032(modulation by symbiont of host I-kappaB kinase/NF-kappaB cascade);GO:0007159(leukocyte cell-cell adhesion);GO:0051019(mitogen-activated protein kinase binding);GO:0009101(glycoprotein biosynthetic process);GO:0043124(negative regulation of I-kappaB kinase/NF-kappaB signaling);GO:0005737(cytoplasm)
circRNA464	GO:0000166(nucleotide binding);GO:0003676(nucleic acid binding);GO:0005622(intracellular);GO:0006810(transport);GO:0005737(cytoplasm);GO:0005515(protein binding);GO:0044822(poly(A) RNA binding);GO:0090090(negative regulation of canonical Wnt signaling pathway);GO:0003729(mRNA binding);GO:0010494(cytoplasmic stress granule)
circRNA465	GO:0016012(sarcoglycan complex);GO:0016021(integral component of membrane);GO:0016010(dystrophin-associated glycoprotein complex);GO:0005515(protein binding);GO:0042383(sarcolemma);GO:0016011(dystroglycan complex)
circRNA466	GO:0003677(DNA binding);GO:0005634(nucleus);GO:0006351("transcription, DNA-templated");GO:0006355("regulation of transcription, DNA-templated");GO:0007275(multicellular organismal development);GO:0046872(metal ion binding);GO:0046983(protein dimerization activity);GO:0005515(protein binding)
circRNA467	GO:0046872(metal ion binding);GO:0016197(endosomal transport);GO:0043231(intracellular membrane-bounded organelle);GO:0005737(cytoplasm);GO:0005515(protein binding);GO:0008565(protein transporter activity);GO:0005769(early endosome);GO:0005545(1-phosphatidylinositol binding);GO:0006622(protein targeting to lysosome);GO:0005547("phosphatidylinositol-3,4,5-trisphosphate
circRNA468	GO:0003677(DNA binding);GO:0005634(nucleus);GO:0006351("transcription, DNA-templated");GO:0006355("regulation of transcription, DNA-templated");GO:0046983(protein dimerization activity);GO:0045893("positive regulation of transcription, DNA-templated");GO:0005737(cytoplasm);GO:0045944(positive regulation of transcription from RNA polymerase II promoter);GO:0005515(protein binding);GO:0003700(sequence-specific DNA binding transcription factor activity);GO:0000122(negative regulation of transcription from RNA polymerase II promoter);GO:0010628(positive regulation of gene expression);GO:0000165(MAPK cascade);GO:0009615(response to virus);GO:0046982(protein heterodimerization activity);GO:0000977(RNA polymerase II regulatory region sequence-specific DNA binding);GO:0001077(RNA polymerase II core promoter proximal region sequence-specific DNA binding transcription factor activity involved in positive regulation of transcription);GO:0071560(cellular response to transforming growth factor beta stimulus);GO:2001016(positive regulation of skeletal muscle cell differentiation);GO:2000727(positive regulation of cardiac muscle cell differentiation);GO:0016607(nuclear speck);GO:0043234(protein complex);GO:0071374(cellular response to parathyroid hormone stimulus);GO:0048643(positive regulation of skeletal muscle tissue development);GO:0035198(miRNA binding);GO:0003680(AT DNA binding);GO:0045663(positive regulation of myoblast differentiation);GO:0014902(myotube differentiation);GO:0000983(RNA polymerase II core promoter sequence-specific DNA binding transcription factor activity);GO:0033613(activating transcription factor binding);GO:0030279(negative regulation of ossification);GO:0030220(platelet formation);GO:0043565(sequence-specific DNA binding);GO:0003682(chromatin binding);GO:0044212(transcription regulatory region DNA binding);GO:0002062(chondrocyte differentiation);GO:0045165(cell fate commitment);GO:0071222(cellular response to lipopolysaccharide);GO:0001568(blood vessel development);GO:0001764(neuron migration);GO:0050680(negative regulation of epithelial cell proliferation);GO:0071277(cellular response to calcium ion);GO:0010629(negative regulation of gene expression);GO:0000981(sequence-specific DNA binding RNA polymerase II transcription factor activity);GO:0003705(RNA polymerase II distal enhancer sequence-specific DNA binding transcription factor activity);GO:0030890(positive regulation of B cell proliferation);GO:0042826(histone deacetylase binding);GO:0045652(regulation of megakaryocyte differentiation);GO:0001958(endochondral ossification);GO:0045669(positive regulation of osteoblast differentiation);GO:0043524(negative regulation of neuron apoptotic process);GO:0002467(germinal center formation);GO:0014898(cardiac muscle hypertrophy in response to stress);GO:0060021(palate development);GO:0007519(skeletal muscle tissue development);GO:0007507(heart development);GO:0003139(secondary heart field specification);GO:0001947(heart looping);GO:0000980(RNA polymerase II distal enhancer sequence-specific DNA binding);GO:0030182(neuron differentiation);GO:0030318(melanocyte differentiation);GO:0035690(cellular response to drug);GO:0030501(positive regulation of bone mineralization);GO:0006959(humoral immune response);GO:0001974(blood vessel remodeling);GO:0043523(regulation of neuron apoptotic process);GO:0007611(learning or memory);GO:0048167(regulation of synaptic plasticity);GO:0051963(regulation of synapse assembly);GO:0060079(regulation of excitatory postsynaptic membrane potential);GO:0030224(monocyte differentiation);GO:0045666(positive regulation of neuron differentiation);GO:0048704(embryonic skeletal system morphogenesis);GO:0055012(ventricular cardiac muscle cell differentiation);GO:0042100(B cell proliferation);GO:0000978(RNA polymerase II core promoter proximal region sequence-specific DNA binding);GO:0003151(outflow tract morphogenesis);GO:0001649(osteoblast differentiation);GO:0050853(B cell receptor signaling pathway);GO:0001782(B cell homeostasis);GO:0000987(core promoter proximal region sequence-specific DNA binding);GO:0010694(positive regulation of alkaline phosphatase activity);GO:0048667(cell morphogenesis involved in neuron differentiation);GO:0055007(cardiac muscle cell differentiation);GO:0060045(positive regulation of cardiac muscle cell proliferation);GO:0048666(neuron development);GO:0001205(RNA polymerase II distal enhancer sequence-specific DNA binding transcription factor activity involved in positive regulation of transcription);GO:0051966("regulation of synaptic transmission, glutamatergic");GO:0051145(smooth muscle cell differentiation);GO:0048703(embryonic viscerocranium morphogenesis);GO:0046928(regulation of neurotransmitter secretion);GO:0071837(HMG box domain binding);GO:2000311(regulation of alpha-amino-3-hydroxy-5-methyl-4-isoxazole propionate selective glutamate receptor activity);GO:0071864(positive regulation of cell proliferation in bone marrow);GO:2000310(regulation of N-methyl-D-aspartate selective glutamate receptor activity);GO:0001046(core promoter sequence-specific DNA binding);GO:0071498(cellular response to fluid shear stress);GO:0072102(glomerulus morphogenesis);GO:0090073(positive regulation of protein homodimerization activity);GO:0061333(renal tubule morphogenesis);GO:0060536(cartilage morphogenesis);GO:2000987(positive regulation of behavioral fear response);GO:0003211(cardiac ventricle formation);GO:2000111(positive regulation of macrophage apoptotic process);GO:0060998(regulation of dendritic spine development);GO:0060297(regulation of sarcomere organization);GO:0014033(neural crest cell differentiation);GO:0002634(regulation of germinal center formation);GO:0007521(muscle cell fate determination);GO:0003138(primary heart field specification);GO:0003185(sinoatrial valve morphogenesis);GO:0035984(cellular response to trichostatin A);GO:0060025(regulation of synaptic activity);GO:0072160(nephron tubule epithelial cell differentiation);GO:2001013(epithelial cell proliferation involved in renal tubule morphogenesis)
circRNA469	GO:0006508(proteolysis);GO:0008237(metallopeptidase activity);GO:0008270(zinc ion binding);GO:0005622(intracellular);GO:0016020(membrane);GO:0005765(lysosomal membrane);GO:0005886(plasma membrane);GO:0005515(protein binding);GO:0030163(protein catabolic process);GO:0048471(perinuclear region of cytoplasm);GO:0004177(aminopeptidase activity)
circRNA47	GO:0000166(nucleotide binding);GO:0005524(ATP binding);GO:0008152(metabolic process);GO:0017111(nucleoside-triphosphatase activity);GO:0009378(four-way junction helicase activity);GO:0006281(DNA repair);GO:0006310(DNA recombination);GO:0016301(kinase activity);GO:0016887(ATPase activity);GO:0016020(membrane);GO:0044822(poly(A) RNA binding)
circRNA470	GO:0003993(acid phosphatase activity);GO:0016311(dephosphorylation)
circRNA471	GO:0003824(catalytic activity);GO:0004553("hydrolase activity, hydrolyzing O-glycosyl compounds");GO:0004559(alpha-mannosidase activity);GO:0005975(carbohydrate metabolic process);GO:0006013(mannose metabolic process);GO:0008270(zinc ion binding);GO:0015923(mannosidase activity);GO:0030246(carbohydrate binding);GO:0070062(extracellular vesicular exosome);GO:0016020(membrane);GO:0005794(Golgi apparatus);GO:0001701(in utero embryonic development);GO:0000139(Golgi membrane);GO:0005801(cis-Golgi network);GO:0007585(respiratory gaseous exchange);GO:0048286(lung alveolus development);GO:0050769(positive regulation of neurogenesis);GO:0007005(mitochondrion organization);GO:0001889(liver development);GO:0060042(retina morphogenesis in camera-type eye);GO:0016799("hydrolase activity, hydrolyzing N-glycosyl compounds");GO:0006491(N-glycan processing);GO:0007033(vacuole organization)

circRNA472	GO:0005509(calcium ion binding);GO:0006810(transport);GO:0016020(membrane);GO:0016021(integral component of membrane);GO:0055085(transmembrane transport);GO:0005739(mitochondrion);GO:0015813(L-glutamate transport);GO:0005313(L-glutamate transmembrane transporter activity);GO:0006754(ATP biosynthetic process);GO:0043490(malate-aspartate shuttle);GO:0015810(aspartate transport);GO:0015183(L-aspartate transmembrane transporter activity);GO:0051592(response to calcium ion);GO:0045333(cellular respiration);GO:0005743(mitochondrial inner membrane)
circRNA473	GO:0000118(histone deacetylase complex);GO:0004407(histone deacetylase activity);GO:0016568(chromatin modification);GO:0016575(histone deacetylation);GO:0005737(cytoplasm);GO:0005667(transcription factor complex);GO:0005515(protein binding);GO:0000122(negative regulation of transcription from RNA polymerase II promoter);GO:0005634(nucleus);GO:0045892("negative regulation of transcription, DNA-templated");GO:0032869(cellular response to insulin stimulus);GO:0070933(histone H4 deacetylation);GO:0070932(histone H3 deacetylation);GO:0008134(transcription factor binding);GO:0034983(peptidyl-lysine deacetylation);GO:0005080(protein kinase C binding);GO:0090050(positive regulation of cell migration involved in sprouting angiogenesis);GO:0042826(histone deacetylase binding);GO:0033558(protein deacetylase activity);GO:0035097(histone methyltransferase complex);GO:0003714(transcription corepressor activity);GO:0007507(heart development);GO:0048742(regulation of skeletal muscle fiber development);GO:0051153(regulation of striated muscle cell differentiation);GO:0070491(repressing transcription factor binding)
circRNA474	GO:0000118(histone deacetylase complex);GO:0004407(histone deacetylase activity);GO:0016568(chromatin modification);GO:0016575(histone deacetylation);GO:0005737(cytoplasm);GO:0005667(transcription factor complex);GO:0005515(protein binding);GO:0000122(negative regulation of transcription from RNA polymerase II promoter);GO:0005634(nucleus);GO:0045892("negative regulation of transcription, DNA-templated");GO:0032869(cellular response to insulin stimulus);GO:0070933(histone H4 deacetylation);GO:0070932(histone H3 deacetylation);GO:0008134(transcription factor binding);GO:0034983(peptidyl-lysine deacetylation);GO:0005080(protein kinase C binding);GO:0090050(positive regulation of cell migration involved in sprouting angiogenesis);GO:0042826(histone deacetylase binding);GO:0033558(protein deacetylase activity);GO:0035097(histone methyltransferase complex);GO:0003714(transcription corepressor activity);GO:0007507(heart development);GO:0048742(regulation of skeletal muscle fiber development);GO:0051153(regulation of striated muscle cell differentiation);GO:0070491(repressing transcription factor binding)
circRNA475	GO:0008152(metabolic process);GO:0008270(zinc ion binding);GO:0016787(hydrolase activity);GO:0070290(N-acylphosphatidylethanolamine-specific phospholipase D activity);GO:0070062(extracellular vesicular exosome)
circRNA476	GO:0000166(nucleotide binding);GO:0004672(protein kinase activity);GO:0004713(protein tyrosine kinase activity);GO:0004714(transmembrane receptor protein tyrosine kinase activity);GO:0004872(receptor activity);GO:0005524(ATP binding);GO:0006468(protein phosphorylation);GO:0007169(transmembrane receptor protein tyrosine kinase signaling pathway);GO:0007275(multicellular organismal development);GO:0016020(membrane);GO:0016021(integral component of membrane);GO:0016301(kinase activity);GO:0016310(phosphorylation);GO:0016740(transferase activity);GO:0016772("transferase activity, transferring phosphorus-containing groups");GO:0018108(peptidyl-tyrosine phosphorylation);GO:0005515(protein binding);GO:0045944(positive regulation of transcription from RNA polymerase II promoter);GO:0019903(protein phosphatase binding);GO:0009925(basal plasma membrane);GO:0071526(semaphorin-plexin signaling pathway);GO:1901299(negative regulation of hydrogen peroxide-mediated programmed cell death);GO:2001028(positive regulation of endothelial cell chemotaxis);GO:0048754(branching morphogenesis of an epithelial tube);GO:0050918(positive chemotaxis);GO:0007420(brain development);GO:0046777(protein autophosphorylation);GO:0042593(glucose homeostasis);GO:0007517(muscle organ development);GO:0007519(skeletal muscle tissue development);GO:0030534(adult behavior);GO:0000187(activation of MAPK activity);GO:0001890(placenta development);GO:0001889(liver development);GO:0014902(myotube differentiation);GO:0010828(positive regulation of glucose transport);GO:0005008(hepatocyte growth factor-activated receptor activity);GO:0014812(muscle cell migration);GO:0048012(hepatocyte growth factor receptor signaling pathway);GO:0051450(myoblast proliferation);GO:0060665(regulation of branching involved in salivary gland morphogenesis by mesenchymal-epithelial signaling)
circRNA477	GO:0005085(guanyl-nucleotide exchange factor activity);GO:0007264(small GTPase mediated signal transduction);GO:0043547(positive regulation of GTPase activity);GO:0005515(protein binding);GO:0016020(membrane);GO:0005829(cytosol);GO:0048365(Rac GTPase binding);GO:0032855(positive regulation of Rac GTPase activity);GO:0030165(PDZ domain binding);GO:0060326(cell chemotaxis);GO:0030971(receptor tyrosine kinase binding);GO:0030675(Rac GTPase activator activity);GO:0032420(stereocilium);GO:0032421(stereocilium bundle)
circRNA478	GO:0004616(phosphogluconate dehydrogenase (decarboxylating) activity);GO:0006098(pentose-phosphate shunt);GO:0008442(3-hydroxyisobutyrate dehydrogenase activity);GO:0016491(oxidoreductase activity);GO:0016616("oxidoreductase activity, acting on the CH-OH group of donors, NAD or NADP as acceptor");GO:0050662(coenzyme binding);GO:0051287(NAD binding);GO:0055114(oxidation-
circRNA479	GO:0004616(phosphogluconate dehydrogenase (decarboxylating) activity);GO:0006098(pentose-phosphate shunt);GO:0008442(3-hydroxyisobutyrate dehydrogenase activity);GO:0016491(oxidoreductase activity);GO:0016616("oxidoreductase activity, acting on the CH-OH group of donors, NAD or NADP as acceptor");GO:0050662(coenzyme binding);GO:0051287(NAD binding);GO:0055114(oxidation-
circRNA48	GO:0000166(nucleotide binding);GO:0005524(ATP binding);GO:0008152(metabolic process);GO:0017111(nucleoside-triphosphatase activity);GO:0009378(four-way junction helicase activity);GO:0006281(DNA repair);GO:0006310(DNA recombination);GO:0016301(kinase activity);GO:0016887(ATPase activity);GO:0016020(membrane);GO:0044822(poly(A) RNA binding)
circRNA480	GO:0016567(protein ubiquitination);GO:0035556(intracellular signal transduction);GO:0005515(protein binding)
circRNA481	GO:0004074(biliverdin reductase activity);GO:0005737(cytoplasm);GO:0008270(zinc ion binding);GO:0016491(oxidoreductase activity);GO:0042167(heme catabolic process);GO:0055114(oxidation-reducti
circRNA482	GO:0016020(membrane);GO:0016021(integral component of membrane);GO:0005887(integral component of plasma membrane);GO:0007166(cell surface receptor signaling pathway);GO:0045765(regulation of angiogenesis);GO:0010842(retina layer formation)
circRNA483	GO:0005783(endoplasmic reticulum);()
circRNA484	GO:0007155(cell adhesion);GO:0005515(protein binding);GO:0005737(cytoplasm)
circRNA485	GO:0070062(extracellular vesicular exosome);GO:0005737(cytoplasm);GO:0005515(protein binding);GO:0000122(negative regulation of transcription from RNA polymerase II promoter);GO:0005634(nucleus);GO:0019902(phosphatase binding);GO:0008053(mitochondrial fusion);GO:0001227(RNA polymerase II transcription regulatory region sequence-specific DNA binding transcription factor activity involved in negative regulation of transcription);GO:0007007(inner mitochondrial membrane organization);GO:0005739(mitochondrion);GO:0005743(mitochondrial inner membrane);GO:0032947(protein complex scaffold)
circRNA486	GO:0005216(ion channel activity);GO:0005247(voltage-gated chloride channel activity);GO:0006821(chloride transport);GO:0016020(membrane);GO:0016021(integral component of membrane);GO:0030554(adenyl nucleotide binding);GO:0034220(ion transmembrane transport);GO:0044070(regulation of anion transport);GO:0055085(transmembrane
circRNA487	GO:0005794(Golgi apparatus);GO:0006486(protein glycosylation);GO:0016020(membrane);GO:0016021(integral component of membrane);GO:0016740(transferase activity);GO:0016757("transferase activity, transferring glycosyl groups");GO:0005112(Notch binding);GO:0018243(protein O-linked glycosylation via threonine);GO:0004653(polypeptide N-acetylgalactosaminyltransferase activity)
circRNA488	GO:0005634(nucleus);GO:0008168(methyltransferase activity);GO:0008270(zinc ion binding);GO:0016740(transferase activity);GO:0032259(methylation);GO:0035556(intracellular signal transduction);GO:0046872(metal ion binding);GO:0005515(protein binding);GO:0044822(poly(A) RNA binding);GO:0035097(histone methyltransferase complex);GO:0042800(histone methyltransferase activity (H3-K4 specific));GO:0051568(histone H3-K4 methylation);GO:0016571(histone methylation)
circRNA489	()
circRNA49	GO:0016020(membrane);GO:0035556(intracellular signal transduction);GO:0005783(endoplasmic reticulum)
circRNA490	GO:0000166(nucleotide binding);GO:0003676(nucleic acid binding);GO:0044822(poly(A) RNA binding)
circRNA491	GO:0000166(nucleotide binding);GO:0003676(nucleic acid binding);GO:0044822(poly(A) RNA binding)
circRNA492	GO:0042733(embryonic digit morphogenesis)
circRNA493	GO:0042733(embryonic digit morphogenesis)
circRNA494	GO:0004842(ubiquitin-protein transferase activity);GO:0008152(metabolic process);GO:0016567(protein ubiquitination);GO:0016874(ligase activity);GO:0005515(protein binding);GO:0000209(protein poly
circRNA495	GO:0048471(perinuclear region of cytoplasm);GO:0016020(membrane);GO:0005829(cytosol);GO:0005737(cytoplasm);GO:0005515(protein binding);GO:0005634(nucleus);GO:0031072(heat shock protein binding);GO:0051082(unfolded protein binding);GO:0006457(protein folding);GO:0030018(Z disc);GO:0043154(negative regulation of cysteine-type endopeptidase activity involved in apoptotic process);GO:0032781(positive regulation of ATPase activity);GO:0001671(ATPase activator activity);GO:0051087(chaperone binding);GO:0090084(negative regulation of inclusion body assembly);GO:0045109(intermediate filament organization);GO:0005813(centrosome);GO:0001669(acrosomal vesicle)
circRNA496	GO:0005515(protein binding);GO:0005737(cytoplasm);GO:0005886(plasma membrane);GO:0008168(methyltransferase activity);GO:0016568(chromatin modification);GO:0018027(peptidyl-lysine dimethylation);GO:0016279(protein-lysine N-methyltransferase activity);GO:0016571(histone methylation);GO:0002039(p53 binding);GO:0005634(nucleus);GO:0045892("negative regulation of transcription, DNA-templated");GO:0000122(negative regulation of transcription from RNA polymerase II promoter);GO:0006306(DNA methylation);GO:0009790(embryo development);GO:0051567(histone H3-K9 methylation);GO:0070742(C2H2 zinc finger domain binding);GO:0070734(histone H3-K27 methylation);GO:0046974(histone methyltransferase activity (H3-K9 specific));GO:0018026(peptidyl-lysine monomethylation);GO:0046976(histone methyltransferase activity (H3-K27 specific))
circRNA497	GO:0005515(protein binding);GO:0005737(cytoplasm);GO:0005886(plasma membrane);GO:0008168(methyltransferase activity);GO:0016568(chromatin modification);GO:0018027(peptidyl-lysine dimethylation);GO:0016279(protein-lysine N-methyltransferase activity);GO:0016571(histone methylation);GO:0002039(p53 binding);GO:0005634(nucleus);GO:0045892("negative regulation of transcription, DNA-templated");GO:0000122(negative regulation of transcription from RNA polymerase II promoter);GO:0006306(DNA methylation);GO:0009790(embryo development);GO:0051567(histone H3-K9 methylation);GO:0070742(C2H2 zinc finger domain binding);GO:0070734(histone H3-K27 methylation);GO:0046974(histone methyltransferase activity (H3-K9 specific));GO:0018026(peptidyl-lysine monomethylation);GO:0046976(histone methyltransferase activity (H3-K27 specific))
circRNA498	GO:0005515(protein binding);GO:0005615(extracellular space);GO:0051259(protein oligomerization);GO:0001540(beta-amyloid binding);GO:0032281(alpha-amino-3-hydroxy-5-methyl-4-isoxazolepropionic acid selective glutamate receptor complex);GO:1902003(regulation of beta-amyloid formation);GO:1902430(negative regulation of beta-amyloid formation);GO:2001223(negative
circRNA499	GO:0008017(microtubule binding);GO:0005515(protein binding);GO:0005874(microtubule);GO:0022604(regulation of cell morphogenesis);GO:0007010(cytoskeleton organization);GO:0030507(spectrin binding);GO:0031175(neuron projection development)
circRNA5	GO:0004842(ubiquitin-protein transferase activity);GO:0008152(metabolic process);GO:0016567(protein ubiquitination);GO:0016874(ligase activity);GO:0005515(protein binding);GO:0005488(binding);GO:0070062(extracellular vesicular exosome);GO:0016020(membrane);GO:0005737(cytoplasm);GO:0044822(poly(A) RNA binding);GO:0000209(protein polyubiquitination);GO:0006284(base-excision repair);GO:0016574(histone ubiquitination);GO:0006513(protein monoubiquitination)

circRNA50	GO:0000166(nucleotide binding);GO:0004672(protein kinase activity);GO:0004674(protein serine/threonine kinase activity);GO:0005083(small GTPase regulator activity);GO:0005524(ATP binding);GO:0006468(protein phosphorylation);GO:0016301(kinase activity);GO:0016310(phosphorylation);GO:0016740(transferase activity);GO:0016772("transferase activity, transferring phosphorus-containing groups");GO:0031532(actin cytoskeleton reorganization);GO:0035556(intracellular signal transduction);GO:0046872(metal ion binding);GO:0050790(regulation of catalytic activity);GO:0004713(protein tyrosine kinase activity);GO:0000287(magnesium ion binding);GO:0070062(extracellular vesicular exosome);GO:0042802(identical protein binding);GO:0031032(actomyosin structure organization);GO:0016477(cell migration);GO:0042641(actomyosin);GO:0000226(microtubule cytoskeleton organization);GO:0007097(nuclear migration)
circRNA500	GO:0016020(membrane);GO:0005829(cytosol);GO:0005737(cytoplasm);GO:0005515(protein binding);GO:0006407(rRNA export from nucleus);GO:0006417(regulation of translation);GO:0005938(cell cortex);GO:0008285(negative regulation of cell proliferation);GO:0007160(cell-matrix adhesion);GO:0051492(regulation of stress fiber assembly);GO:0032862(activation of Rho GTPase activity);GO:0047485(protein N-terminus binding);GO:0050821(protein stabilization);GO:0043666(regulation of phosphoprotein phosphatase activity);GO:0033596(TSC1-TSC2 complex);GO:0032007(negative regulation of TOR signaling);GO:0017148(negative regulation of translation);GO:0030027(lamellipodium);GO:0005884(actin filament);GO:0051087(chaperone binding);GO:0043234(protein complex);GO:0001952(regulation of cell-matrix adhesion);GO:0051894(positive regulation of focal adhesion assembly);GO:0032868(response to insulin);GO:0050808(synapse organization);GO:0006813(potassium ion transport);GO:0007399(nervous system development);GO:0001843(neural tube closure);GO:0021987(cerebral cortex development);GO:0001822(kidney development);GO:0042552(myelination);GO:0030030(cell projection organization);GO:0045859(regulation of protein kinase activity);GO:0055007(cardiac muscle cell differentiation);GO:0021766(hippocampus development);GO:0045792(negative regulation of cell size)
circRNA501	GO:0004017(adenylate kinase activity);GO:0005524(ATP binding);GO:0006139(nucleobase-containing compound metabolic process);GO:0016310(phosphorylation);GO:0019205(nucleobase-containing compound kinase activity);GO:0046939(nucleotide phosphorylation);GO:0006165(nucleoside diphosphate phosphorylation);GO:0004550(nucleoside diphosphate kinase activity);GO:0004127(cytidylate kinase activity);GO:0009142(nucleoside triphosphate biosynthetic process);GO:0021591(ventricular system development)
circRNA502	GO:0005515(protein binding);GO:0043231(intracellular membrane-bounded organelle);GO:0005634(nucleus);GO:0005925(focal adhesion);GO:0006611(protein export from nucleus);GO:0006606(protein import into nucleus);GO:0006406(mRNA export from nucleus);GO:0051726(regulation of cell cycle);GO:0005487(nucleocytoplasmic transporter activity)
circRNA503	GO:0019211(phosphatase activator activity);GO:0043085(positive regulation of catalytic activity);GO:0070062(extracellular vesicular exosome);GO:0005737(cytoplasm);GO:0005524(ATP binding);GO:0005634(nucleus);GO:0043065(positive regulation of apoptotic process);GO:0016887(ATPase activity);GO:0006200(ATP catabolic process);GO:0042803(protein homodimerization activity);GO:0005102(receptor binding);GO:0035307(positive regulation of protein dephosphorylation);GO:0043666(regulation of phosphoprotein phosphatase activity);GO:0032515(negative regulation of phosphoprotein phosphatase activity);GO:0035308(negative regulation of protein dephosphorylation);GO:0008160(protein tyrosine phosphatase activator activity);GO:0032516(positive regulation of phosphoprotein phosphatase activity);GO:0051721(protein phosphatase 2A binding);GO:0034704(calcium channel complex)
circRNA504	GO:0080025("phosphatidylinositol-3,5-bisphosphate binding");GO:0005515(protein binding);GO:0005886(plasma membrane);GO:0005794(Golgi apparatus);GO:0005546("phosphatidylinositol-4,5-bisphosphate binding");GO:0021762(substantia nigra development);GO:0070300(phosphatidic acid binding);GO:0017016(Ras GTPase binding);GO:0043325("phosphatidylinositol-3,4-bisphosphate binding");GO:0046580(negative regulation of Ras protein signal transduction);GO:0005547("phosphatidylinositol-3,4,5-trisphosphate binding")
circRNA505	GO:0005096(GTPase activator activity);GO:0005622(intracellular);GO:0007165(signal transduction);GO:0043547(positive regulation of GTPase activity);GO:0051056(regulation of small GTPase mediated signal transduction);GO:0005085(guanyl-nucleotide exchange factor activity);GO:0005829(cytosol);GO:0051223(regulation of protein transport);GO:0032794(GTPase activating protein binding);GO:0005515(protein binding);GO:0043087(regulation of GTPase activity)
circRNA506	GO:0005515(protein binding);GO:0043231(intracellular membrane-bounded organelle);GO:0005886(plasma membrane)
circRNA507	GO:0016020(membrane);GO:2000623("negative regulation of nuclear-transcribed mRNA catabolic process, nonsense-mediated decay");GO:0000956(nuclear-transcribed mRNA catabolic process);()
circRNA508	GO:0016020(membrane);GO:2000623("negative regulation of nuclear-transcribed mRNA catabolic process, nonsense-mediated decay");GO:0000956(nuclear-transcribed mRNA catabolic process);()
circRNA509	GO:0016740(transferase activity);GO:0003677(DNA binding);GO:0006306(DNA methylation);GO:0008168(methyltransferase activity);GO:0005737(cytoplasm);GO:0005515(protein binding);GO:0005634(nucleus);GO:0016363(nuclear matrix);GO:0003886(DNA (cytosine-5-)-methyltransferase activity);GO:0000791(euchromatin);GO:0003682(chromatin binding);GO:0000122(negative regulation of transcription from RNA polymerase II promoter);GO:0007283(spermatogenesis);GO:0000792(heterochromatin);GO:0005720(nuclear heterochromatin);GO:0071230(cellular response to amino acid stimulus);GO:0000775("chromosome, centromeric region");GO:0006349(regulation of gene expression by genetic imprinting);GO:0043046(DNA methylation involved in gamete generation);GO:0006346(methylation-dependent chromatin silencing);GO:0043045(DNA methylation involved in embryo development);GO:0044027(hypermethylation of CpG island)
circRNA51	GO:0000166(nucleotide binding);GO:0004672(protein kinase activity);GO:0004674(protein serine/threonine kinase activity);GO:0005083(small GTPase regulator activity);GO:0005524(ATP binding);GO:0006468(protein phosphorylation);GO:0016301(kinase activity);GO:0016310(phosphorylation);GO:0016740(transferase activity);GO:0016772("transferase activity, transferring phosphorus-containing groups");GO:0031532(actin cytoskeleton reorganization);GO:0035556(intracellular signal transduction);GO:0046872(metal ion binding);GO:0050790(regulation of catalytic activity);GO:0004713(protein tyrosine kinase activity);GO:0000287(magnesium ion binding);GO:0070062(extracellular vesicular exosome);GO:0042802(identical protein binding);GO:0031032(actomyosin structure organization);GO:0016477(cell migration);GO:0042641(actomyosin);GO:0000226(microtubule cytoskeleton organization);GO:0007097(nuclear migration)
circRNA510	GO:0003824(catalytic activity);GO:0003857(3-hydroxyacyl-CoA dehydrogenase activity);GO:0004300(enoyl-CoA hydratase activity);GO:0005739(mitochondrion);GO:0006631(fatty acid metabolic process);GO:0006635(fatty acid beta-oxidation);GO:0008152(metabolic process);GO:0016491(oxidoreductase activity);GO:0016507(mitochondrial fatty acid beta-oxidation multienzyme complex);GO:0016616("oxidoreductase activity, acting on the CH-OH group of donors, NAD or NADP as acceptor");GO:0050662(coenzyme binding);GO:0055114(oxidation-reduction process);GO:0005515(protein binding);GO:0042645(mitochondrial nucleoid);GO:0005743(mitochondrial inner membrane);GO:0032868(response to insulin);GO:0016509(long-chain-3-hydroxyacyl-CoA dehydrogenase activity)
circRNA511	GO:0005515(protein binding)
circRNA512	GO:0005515(protein binding)
circRNA513	GO:0004672(protein kinase activity);GO:0004674(protein serine/threonine kinase activity);GO:0005524(ATP binding);GO:0006468(protein phosphorylation);GO:0016772("transferase activity, transferring phosphorus-containing groups");GO:0004713(protein tyrosine kinase activity);GO:0005515(protein binding);GO:0035612(AP-2 adaptor complex binding);GO:0005112(Notch binding);GO:0050821(protein stabilization);GO:2000369(regulation of clathrin-mediated endocytosis);GO:0032880(regulation of protein localization);GO:0045747(positive regulation of Notch signaling pathway)
circRNA514	GO:0004360(glutamine-fructose-6-phosphate transaminase (isomerizing) activity);GO:0005975(carbohydrate metabolic process);GO:0008152(metabolic process);GO:0016051(carbohydrate biosynthetic process);GO:0030246(carbohydrate binding);GO:0070062(extracellular vesicular exosome);GO:0032922(circadian regulation of gene expression)
circRNA515	GO:0005509(calcium ion binding)
circRNA516	GO:0008152(metabolic process);GO:0016779(nucleotidyltransferase activity);GO:0070569(uridylyltransferase activity);GO:0003983(UTP:glucose-1-phosphate uridylyltransferase activity);GO:0006011(UDP-glucose metabolic process);GO:0070062(extracellular vesicular exosome);GO:0005515(protein binding);GO:0005634(nucleus)
circRNA517	GO:0005515(protein binding)
circRNA518	GO:0005856(cytoskeleton);GO:0006909(phagocytosis)
circRNA519	GO:0008488(gamma-glutamyl carboxylase activity);GO:0017187(peptidyl-glutamic acid carboxylation)
circRNA52	GO:0003676(nucleic acid binding);GO:0005634(nucleus);GO:0005515(protein binding);GO:0005524(ATP binding);GO:0016301(kinase activity);GO:0016020(membrane);GO:0044822(poly(A) RNA binding);GO:0030529(ribonucleoprotein complex);GO:0036464(cytoplasmic ribonucleoprotein granule);GO:0070937(CRD-mediated mRNA stability complex);GO:0070934(CRD-mediated mRNA stabilization);GO:0001649(osteoblast differentiation);GO:0071013(catalytic step 2 spliceosome)
circRNA520	GO:0005783(endoplasmic reticulum);GO:0016020(membrane);GO:0005737(cytoplasm);GO:0005515(protein binding);GO:0008017(microtubule binding);GO:0051205(protein insertion into membrane);GO:0031849(olfactory receptor binding);GO:0071786(endoplasmic reticulum tubular network organization);GO:0005789(endoplasmic reticulum membrane);GO:0032386(regulation of
circRNA521	GO:0015031(protein transport);GO:0007034(vacuolar transport)
circRNA522	GO:0005515(protein binding)
circRNA523	GO:0046872(metal ion binding);GO:0005515(protein binding);GO:0045663(positive regulation of myoblast differentiation);GO:0010831(positive regulation of myotube differentiation);GO:0005737(cytoplasm);GO:0005634(nucleus);GO:0045892("negative regulation of transcription, DNA-templated");GO:0003714(transcription corepressor activity);GO:0006338(chromatin remodeling);GO:0035914(skeletal muscle cell differentiation);GO:0007507(heart development)
circRNA524	GO:0046872(metal ion binding);GO:0005515(protein binding);GO:0045663(positive regulation of myoblast differentiation);GO:0010831(positive regulation of myotube differentiation);GO:0005737(cytoplasm);GO:0005634(nucleus);GO:0045892("negative regulation of transcription, DNA-templated");GO:0003714(transcription corepressor activity);GO:0006338(chromatin remodeling);GO:0035914(skeletal muscle cell differentiation);GO:0007507(heart development)
circRNA525	GO:0008270(zinc ion binding);GO:0046872(metal ion binding);GO:0016337(single organismal cell-cell adhesion);GO:0005515(protein binding);GO:0050680(negative regulation of epithelial cell proliferation);GO:0005925(focal adhesion);GO:0043066(negative regulation of apoptotic process);GO:0007160(cell-matrix adhesion);GO:0045216(cell-cell junction organization);GO:0007163(establishment or maintenance of cell polarity);GO:0043009(chordate embryonic development);GO:2000178(negative regulation of neural precursor cell proliferation);GO:2000346(negative regulation of hepatocyte proliferation);GO:2001046(positive regulation of integrin-mediated signaling pathway)

circRNA526	GO:0016020(membrane);GO:0016021(integral component of membrane);GO:0030176(integral component of endoplasmic reticulum membrane);GO:0030308(negative regulation of cell growth);GO:0005622(intracellular);GO:0005783(endoplasmic reticulum);GO:0070062(extracellular vesicular exosome);GO:0005515(protein binding);GO:0044822(poly(A) RNA binding);GO:0005635(nuclear envelope);GO:0030334(regulation of cell migration);GO:0030517(negative regulation of axon extension);GO:0071786(endoplasmic reticulum tubular network organization);GO:0043025(neuronal cell body);GO:0007399(nervous system development);GO:0042995(cell projection);GO:0001525(angiogenesis);GO:0007413(axonal fasciculation);GO:0060317(cardiac epithelial to mesenchymal transition);GO:2000172(regulation of branching morphogenesis of a nerve);GO:0021801(cerebral cortex radial glia guided migration);GO:0051960(regulation of nervous system development)
circRNA527	GO:0016567(protein ubiquitination);GO:0035556(intracellular signal transduction);GO:0005515(protein binding)
circRNA528	GO:0000166(nucleotide binding);GO:0004672(protein kinase activity);GO:0004674(protein serine/threonine kinase activity);GO:0004697(protein kinase C activity);GO:0004699(calcium-independent protein kinase C activity);GO:0005524(ATP binding);GO:0005634(nucleus);GO:0005737(cytoplasm);GO:0005856(cytoskeleton);GO:0005886(plasma membrane);GO:0006468(protein
circRNA529	GO:0003676(nucleic acid binding);GO:0003723(RNA binding);GO:0006139(nucleobase-containing compound metabolic process);GO:0016788("hydrolase activity, acting on ester bonds")
circRNA53	GO:0000166(nucleotide binding);GO:0004672(protein kinase activity);GO:0004674(protein serine/threonine kinase activity);GO:0005524(ATP binding);GO:0006468(protein phosphorylation);GO:0016301(kinase activity);GO:0016310(phosphorylation);GO:0016740(transferase activity);GO:0016772("transferase activity, transferring phosphorus-containing
circRNA530	GO:0003676(nucleic acid binding);GO:0003723(RNA binding);GO:0006139(nucleobase-containing compound metabolic process);GO:0016788("hydrolase activity, acting on ester bonds")
circRNA531	GO:0003676(nucleic acid binding);GO:0003723(RNA binding);GO:0006139(nucleobase-containing compound metabolic process);GO:0016788("hydrolase activity, acting on ester bonds")
circRNA532	GO:0003676(nucleic acid binding);GO:0003723(RNA binding);GO:0006139(nucleobase-containing compound metabolic process);GO:0016788("hydrolase activity, acting on ester bonds")
circRNA533	GO:0005515(protein binding);GO:0005923(tight junction);GO:0016055(Wnt signaling pathway);GO:0030331(estrogen receptor binding);GO:0008285(negative regulation of cell proliferation);GO:0032403(protein complex binding);GO:0000159(protein phosphatase type 2A complex);GO:0051721(protein phosphatase 2A binding);GO:0070016(armadillo repeat domain binding)
circRNA534	GO:0005515(protein binding)
circRNA535	GO:0001558(regulation of cell growth);GO:0004857(enzyme inhibitor activity);GO:0004867(serine-type endopeptidase inhibitor activity);GO:0005520(insulin-like growth factor binding);GO:0005576(extracellular region);GO:0010951(negative regulation of endopeptidase activity);GO:0043086(negative regulation of catalytic activity);GO:0005515(protein
circRNA536	()
circRNA537	GO:0016021(integral component of membrane);GO:0005515(protein binding);GO:0070062(extracellular vesicular exosome);GO:0005544(calcium-dependent phospholipid binding);GO:0005509(calcium ion binding);GO:0005886(plasma membrane);GO:0005543(phospholipid binding);GO:0042383(sarcolemma);GO:0030315(T-tubule);GO:0031410(cytoplasmic
circRNA538	GO:0016197(endosomal transport);GO:0051492(regulation of stress fiber assembly);GO:0005813(centrosome);GO:0005737(cytoplasm);GO:0005515(protein binding);GO:0005814(centriole)
circRNA539	GO:0005083(small GTPase regulator activity);GO:0006886(intracellular protein transport);GO:0016192(vesicle-mediated transport);GO:0050790(regulation of catalytic activity);GO:0006355("regulation of transcription, DNA-templated");GO:0016020(membrane);GO:0005515(protein binding);GO:0007165(signal transduction);GO:0005160(transforming growth factor beta receptor
circRNA54	GO:0005515(protein binding);GO:0070062(extracellular vesicular exosome)
circRNA540	GO:0005215(transporter activity);GO:0005886(plasma membrane);GO:0016021(integral component of membrane);GO:0055085(transmembrane transport);GO:0022857(transmembrane transporter activity)
circRNA541	GO:0006810(transport);GO:0006811(ion transport);GO:0006812(cation transport);GO:0006814(sodium ion transport);GO:0006885(regulation of pH);GO:0015297(antiporter activity);GO:0015299(solute:proton antiporter activity);GO:0015385(sodium:proton antiporter activity);GO:0016021(integral component of membrane);GO:0035725(sodium ion transmembrane transport);GO:0055085(transmembrane transport);GO:1902600(hydrogen ion transmembrane transport);GO:0008104(protein localization)
circRNA542	GO:0003677(DNA binding);GO:0003700(sequence-specific DNA binding transcription factor activity);GO:0004871(signal transducer activity);GO:0005634(nucleus);GO:0005667(transcription factor complex);GO:0005737(cytoplasm);GO:0006351("transcription, DNA-templated");GO:0006355("regulation of transcription, DNA-templated");GO:0007165(signal transduction);GO:0046983(protein dimerization activity);GO:0005515(protein binding);GO:0045893("positive regulation of transcription, DNA-templated");GO:0045944(positive regulation of transcription from RNA polymerase II promoter);GO:0051879(Hsp90 protein binding);GO:0051775(response to redox state);GO:0045739(positive regulation of DNA repair);GO:2001020(regulation of response to DNA damage stimulus);GO:0060548(negative regulation of cell death);GO:0045475(locomotor rhythm);GO:0042745(circadian sleep/wake cycle)
circRNA543	GO:0000287(magnesium ion binding);GO:0003684(damaged DNA binding);GO:0003887(DNA-directed DNA polymerase activity);GO:0006261(DNA-dependent DNA replication);GO:0006281(DNA repair);GO:0016779(nucleotidyltransferase activity);GO:0005515(protein binding);GO:0009411(response to UV);GO:0017125(deoxycytidyl transferase activity);GO:0042276(error-prone translesion
circRNA544	GO:0000287(magnesium ion binding);GO:0003684(damaged DNA binding);GO:0003887(DNA-directed DNA polymerase activity);GO:0006261(DNA-dependent DNA replication);GO:0006281(DNA repair);GO:0016779(nucleotidyltransferase activity);GO:0005515(protein binding);GO:0009411(response to UV);GO:0017125(deoxycytidyl transferase activity);GO:0042276(error-prone translesion
circRNA545	GO:0005515(protein binding);GO:0031432(titin binding);GO:0005634(nucleus);GO:0006631(fatty acid metabolic process);GO:0031674(I band)
circRNA546	();GO:0005730(nucleolus);GO:0043231(intracellular membrane-bounded organelle);GO:0005634(nucleus);GO:0000123(histone acetyltransferase complex);GO:0043984(histone H4-K16 acetylation);GO:0046972(histone acetyltransferase activity (H4-K16 specific));GO:0043996(histone acetyltransferase activity (H4-K8 specific));GO:0043995(histone acetyltransferase activity (H4-K5 specific));GO:0043982(histone H4-K8 acetylation);GO:0043981(histone H4-K5 acetylation)
circRNA547	GO:0008270(zinc ion binding);GO:0046872(metal ion binding);GO:0005515(protein binding)
circRNA548	GO:0005515(protein binding);GO:0005813(centrosome);GO:0042384(cilium assembly);GO:0008283(cell proliferation);GO:0005814(centriole);GO:0000922(spindle pole);GO:0036064(ciliary basal body)
circRNA549	GO:0003725(double-stranded RNA binding);GO:0016442(RISC complex);GO:0030422(production of siRNA involved in RNA interference);GO:0031054(pre-miRNA processing);GO:0035197(siRNA binding);GO:0035280(miRNA loading onto RISC involved in gene silencing by miRNA);GO:0046782(regulation of viral transcription);GO:0003723(RNA binding);GO:0005634(nucleus);GO:0005737(cytoplasm);GO:0006417(regulation of translation);GO:0030423(targeting of mRNA for destruction involved in RNA interference);GO:0031047(gene silencing by RNA);GO:0035198(miRNA binding);GO:0042803(protein homodimerization activity);GO:0048471(perinuclear region of cytoplasm);GO:0005515(protein binding);GO:0045070(positive regulation of viral genome replication);GO:0050689(negative regulation of defense response to virus by host)
circRNA55	GO:0005216(ion channel activity);GO:0005247(voltage-gated chloride channel activity);GO:0006821(chloride transport);GO:0016020(membrane);GO:0016021(integral component of membrane);GO:0030554(adenyl nucleotide binding);GO:0034220(ion transmembrane transport);GO:0044070(regulation of anion transport);GO:0055085(transmembrane transport);GO:0005765(lysosomal
circRNA550	GO:0005515(protein binding);GO:0005739(mitochondrion)
circRNA551	GO:0003676(nucleic acid binding);GO:0016020(membrane);GO:0044822(poly(A) RNA binding);GO:0022604(regulation of cell morphogenesis);GO:0007010(cytoskeleton organization)
circRNA552	GO:0004367(glycerol-3-phosphate dehydrogenase [NAD+] activity);GO:0005737(cytoplasm);GO:0005975(carbohydrate metabolic process);GO:0006072(glycerol-3-phosphate metabolic process);GO:0009331(glycerol-3-phosphate dehydrogenase complex);GO:0016491(oxidoreductase activity);GO:0016616("oxidoreductase activity, acting on the CH-OH group of donors, NAD or NADP as acceptor");GO:0042803(protein homodimerization activity);GO:0046168(glycerol-3-phosphate catabolic process);GO:0050662(coenzyme binding);GO:0051287(NAD binding);GO:0055114(oxidation-reduction process);GO:0070062(extracellular vesicular exosome);GO:0005739(mitochondrion);GO:0071320(cellular response to cAMP);GO:0006094(gluconeogenesis);GO:0071356(cellular response to tumor necrosis factor);GO:0045821(positive regulation of glycolytic process);GO:0006127(glycerophosphate shuttle)
circRNA553	GO:0000123(histone acetyltransferase complex);GO:0043984(histone H4-K16 acetylation);GO:0046972(histone acetyltransferase activity (H4-K16 specific));GO:0043996(histone acetyltransferase activity (H4-K8 specific));GO:0043995(histone acetyltransferase activity (H4-K5 specific));GO:0043982(histone H4-K8 acetylation);GO:0043981(histone H4-K5 acetylation)
circRNA554	GO:0005515(protein binding)
circRNA555	GO:0003677(DNA binding);GO:0005622(intracellular);GO:0005634(nucleus);GO:0006355("regulation of transcription, DNA-templated");GO:0046872(metal ion binding);GO:0005515(protein binding);GO:0006337(nucleosome disassembly)
circRNA556	GO:0016020(membrane);GO:0016021(integral component of membrane);GO:0044070(regulation of anion transport);GO:0005622(intracellular);GO:0006821(chloride transport);GO:0005229(intracellular calcium activated chloride channel activity);GO:0070062(extracellular vesicular exosome);GO:0005886(plasma membrane);GO:0034707(chloride channel complex);GO:0005247(voltage-gated chloride channel activity);GO:0006812(cation transport);GO:0097045(phosphatidylserine exposure on blood platelet);GO:0002543(activation of blood coagulation via clotting cascade);GO:2000353(positive regulation of endothelial cell apoptotic process);GO:0042803(protein homodimerization activity);GO:0017128(phospholipid scramblase activity);GO:0061590(calcium activated phosphatidylcholine scrambling);GO:0061591(calcium activated galactosylceramide scrambling);GO:0007596(blood coagulation);GO:0005227(calcium activated cation channel activity);GO:0061589(calcium activated phosphatidylserine scrambling);GO:0005244(voltage-gated ion channel activity);GO:0035630(bone mineralization involved in bone maturation);GO:0002407(dendritic cell chemotaxis);GO:0017121(phospholipid scrambling);GO:0061588(calcium activated phospholipid scrambling)
circRNA557	GO:0043231(intracellular membrane-bounded organelle);GO:0005737(cytoplasm);GO:0044822(poly(A) RNA binding);GO:0005634(nucleus);GO:0005794(Golgi apparatus)
circRNA558	GO:0008152(metabolic process);GO:0016757("transferase activity, transferring glycosyl groups");GO:0035252(UDP-xylosyltransferase activity);GO:0016266(O-glycan processing)
circRNA559	()
circRNA56	GO:0005509(calcium ion binding);GO:0007156(homophilic cell adhesion);GO:0008013(beta-catenin binding);GO:0016020(membrane);GO:0016021(integral component of membrane);GO:0070062(extracellular vesicular exosome);GO:0005515(protein binding);GO:0001540(beta-amyloid binding);GO:0019894(kinesin binding);GO:0042988(X11-like protein
circRNA560	GO:0000166(nucleotide binding);GO:0003676(nucleic acid binding);GO:0016020(membrane);GO:0005515(protein binding);GO:0044822(poly(A) RNA binding);GO:0005634(nucleus);GO:0006397(mRNA processing);GO:0003723(RNA binding);GO:0030529(ribonucleoprotein complex);GO:0003729(mRNA binding);GO:0006378(mRNA polyadenylation);GO:0005849(mRNA cleavage factor complex);GO:0042382(paraspeckles);GO:0051262(protein tetramerization)

circRNA561	GO:0000166(nucleotide binding);GO:0005525(GTP binding);GO:0006184(GTP catabolic process);GO:0007165(signal transduction);GO:0007264(small GTPase mediated signal transduction);GO:0016020(membrane);GO:0003924(GTPase activity);GO:0006886(intracellular protein transport);GO:0006913(nucleocytoplasmic transport);GO:0005622(intracellular);GO:0015031(protein transport);GO:0070062(extracellular vesicular exosome);GO:0005515(protein binding);GO:2000114(regulation of establishment of cell polarity);GO:0005911(cell-cell junction);GO:0061028(establishment of endothelial barrier);GO:0019003(GDP binding);GO:0005811(lipid particle);GO:0032403(protein complex binding);GO:1901888(regulation of cell junction assembly);GO:0032486(Rap protein signal transduction);GO:0071320(cellular response to cAMP);GO:0008283(cell proliferation);GO:0070374(positive regulation of ERK1 and ERK2 cascade);GO:0045955(negative regulation of calcium ion-dependent exocytosis);GO:2000301(negative regulation of synaptic vesicle exocytosis)
circRNA562	GO:0000166(nucleotide binding);GO:0005639(integral component of nuclear inner membrane);GO:0005515(protein binding);GO:0016021(integral component of membrane);GO:0030512(negative regulation of transforming growth factor beta receptor signaling pathway);GO:0030514(negative regulation of BMP signaling pathway);GO:0005637(nuclear inner membrane);GO:0032926(negative regulation of activin receptor signaling pathway);GO:0035914(skeletal muscle cell differentiation);GO:0005635(nuclear envelope);GO:0051726(regulation of cell cycle)
circRNA563	GO:0005622(intracellular);GO:0007165(signal transduction);GO:0005515(protein binding);GO:0007266(Rho protein signal transduction);GO:0032321(positive regulation of Rho GTPase activity);GO:0016477(cell migration);GO:0017048(Rho GTPase binding);GO:0005100(Rho GTPase activator activity)
circRNA564	GO:0007155(cell adhesion);GO:0007229(integrin-mediated signaling pathway);GO:0008305(integrin complex);GO:0016020(membrane);GO:0016021(integral component of membrane);GO:0005515(protein binding);GO:0005737(cytoplasm);GO:0005927(muscle tendon junction);GO:0016477(cell migration);GO:0042383(sarcolemma);GO:0031594(neuromuscular junction);GO:0048514(blood vessel morphogenesis);GO:0043236(laminin binding);GO:0034677(integrin alpha7-beta1 complex)
circRNA565	GO:0006508(proteolysis);GO:0008233(peptidase activity);GO:0005622(intracellular);GO:0006952(defense response);GO:0042981(regulation of apoptotic process);GO:0043531(ADP binding);GO:0005515(protein binding);GO:0070062(extracellular vesicular exosome);GO:0006919(activation of cysteine-type endopeptidase activity involved in apoptotic process);GO:0008635(activation of cysteine-type endopeptidase activity involved in apoptotic process by cytochrome c);GO:0005634(nucleus);GO:0007420(brain development);GO:0042802(identical protein binding);GO:0030900(forebrain development);GO:0005829(cytosol);GO:0007275(multicellular organismal development);GO:0001843(neural tube closure);GO:0051402(neuron apoptotic process);GO:2001235(positive regulation of apoptotic signaling pathway);GO:1902510(regulation of apoptotic DNA fragmentation);GO:0008656(cysteine-type endopeptidase activator activity involved in apoptotic process)
circRNA566	GO:0005515(protein binding);GO:0006936(muscle contraction)
circRNA567	GO:0005515(protein binding);GO:0006936(muscle contraction)
circRNA568	GO:0005515(protein binding);GO:0006936(muscle contraction)
circRNA569	GO:0005515(protein binding);GO:0006936(muscle contraction)
circRNA57	GO:0003677(DNA binding);GO:0003682(chromatin binding);GO:0005515(protein binding);GO:0000118(histone deacetylase complex);GO:0006338(chromatin remodeling)
circRNA570	GO:0005515(protein binding);GO:0006936(muscle contraction)
circRNA571	GO:0004791(thioredoxin-disulfide reductase activity);GO:0016491(oxidoreductase activity);GO:0016668("oxidoreductase activity, acting on a sulfur group of donors, NAD(P) as acceptor");GO:0045454(cell redox homeostasis);GO:0050660(flavin adenine dinucleotide binding);GO:0050661(NADP binding);GO:0055114(oxidation-reduction process);GO:0005730(nucleolus);GO:0005515(protein binding);GO:0005634(nucleus);GO:0005737(cytoplasm);GO:0005739(mitochondrion);GO:0005829(cytosol);GO:0008283(cell proliferation);GO:0001707(mesoderm formation);GO:0007369(gastrulation)
circRNA572	GO:0000166(nucleotide binding);GO:0004672(protein kinase activity);GO:0004674(protein serine/threonine kinase activity);GO:0005524(ATP binding);GO:0006468(protein phosphorylation);GO:0016772("transferase activity, transferring phosphorus-containing groups");GO:0016773("phosphotransferase activity, alcohol group as acceptor");GO:0009103(lipopolysaccharide biosynthetic process);GO:0016020(membrane);GO:0004713(protein tyrosine kinase activity);GO:0005730(nucleolus);GO:0005737(cytoplasm);GO:0005515(protein binding);GO:0005634(nucleus);GO:0015630(microtubule cytoskeleton);GO:2000772(regulation of cellular senescence);GO:0030155(regulation of cell adhesion);GO:0002039(p53 binding);GO:0035507(regulation of myosin-light-chain-phosphatase activity)
circRNA573	GO:0008152(metabolic process);GO:0016757("transferase activity, transferring glycosyl groups");GO:0005515(protein binding);GO:0046716(muscle cell cellular homeostasis)
circRNA574	GO:0000166(nucleotide binding);GO:0005524(ATP binding);GO:0006200(ATP catabolic process);GO:0006810(transport);GO:0006813(potassium ion transport);GO:0007165(signal transduction);GO:0008152(metabolic process);GO:0008281(sulfonylurea receptor activity);GO:0016020(membrane);GO:0016021(integral component of membrane);GO:0016887(ATPase activity);GO:0017111(nucleoside-triphosphatase activity);GO:0042626("ATPase activity, coupled to transmembrane movement of substances");GO:0055085(transmembrane transport);GO:0008076(voltage-gated potassium channel complex);GO:0051607(defense response to virus);GO:0044325(ion channel binding);GO:0005886(plasma membrane);GO:0071805(potassium ion transmembrane transport);GO:0042383(sarcolemma);GO:0005267(potassium channel activity);GO:0030017(sarcomere)
circRNA575	GO:0000166(nucleotide binding);GO:0005524(ATP binding);GO:0006200(ATP catabolic process);GO:0006810(transport);GO:0006813(potassium ion transport);GO:0007165(signal transduction);GO:0008152(metabolic process);GO:0008281(sulfonylurea receptor activity);GO:0016020(membrane);GO:0016021(integral component of membrane);GO:0016887(ATPase activity);GO:0017111(nucleoside-triphosphatase activity);GO:0042626("ATPase activity, coupled to transmembrane movement of substances");GO:0055085(transmembrane transport);GO:0008076(voltage-gated potassium channel complex);GO:0051607(defense response to virus);GO:0044325(ion channel binding);GO:0005886(plasma membrane);GO:0071805(potassium ion transmembrane transport);GO:0042383(sarcolemma);GO:0005267(potassium channel activity);GO:0030017(sarcomere)
circRNA576	GO:0016491(oxidoreductase activity);GO:0055114(oxidation-reduction process)
circRNA577	GO:0000188(inactivation of MAPK activity);GO:0004725(protein tyrosine phosphatase activity);GO:0006470(protein dephosphorylation);GO:0008138(protein tyrosine/serine/threonine phosphatase activity);GO:0016311(dephosphorylation);GO:0016787(hydrolase activity);GO:0016791(phosphatase activity);GO:0017017(MAP kinase tyrosine/serine/threonine phosphatase activity);GO:0035335(peptidyl-tyrosine dephosphorylation);GO:0005515(protein binding);GO:0005737(cytoplasm);GO:0005634(nucleus);GO:0043409(negative regulation of MAPK cascade)
circRNA578	GO:0016020(membrane);GO:0016021(integral component of membrane);GO:0005515(protein binding);GO:0045893("positive regulation of transcription, DNA-templated");GO:0045944(positive regulation of transcription from RNA polymerase II promoter);GO:0005886(plasma membrane);GO:0016055(Wnt signaling pathway);GO:0009986(cell surface);GO:0031410(cytoplasmic vesicle);GO:0060070(canonical Wnt signaling pathway);GO:0044332(Wnt signaling pathway involved in dorsal/ventral axis specification);GO:0017147(Wnt-protein binding);GO:0005794(Golgi apparatus);GO:0042803(protein homodimerization activity);GO:0005102(receptor binding);GO:0005769(early endosome);GO:0071901(negative regulation of protein serine/threonine kinase activity);GO:0006469(negative regulation of protein kinase activity);GO:0005109(frizzled binding);GO:1901998(toxin transport);GO:0019534(toxin transporter activity);GO:0051091(positive regulation of sequence-specific DNA binding transcription factor activity);GO:0090263(positive regulation of canonical Wnt signaling pathway);GO:0001933(negative regulation of protein phosphorylation);GO:0042802(identical protein binding);GO:0005901(caveola);GO:2000055(positive regulation of Wnt signaling pathway involved in dorsal/ventral axis specification);GO:0071936(coreceptor activity involved in Wnt signaling pathway);GO:0071397(cellular response to cholesterol);GO:0034392(negative regulation of smooth muscle cell apoptotic process);GO:0014033(neural crest cell differentiation);GO:0014029(neural crest formation);GO:0005041(low-density lipoprotein receptor activity);GO:0019210(kinase inhibitor
circRNA579	GO:0003676(nucleic acid binding);GO:0003677(DNA binding);GO:0006355("regulation of transcription, DNA-templated");GO:0005737(cytoplasm);GO:0044822(poly(A) RNA binding);GO:0071356(cellular response to tumor necrosis factor);GO:0060546(negative regulation of necroptotic process);GO:1902219(negative regulation of intrinsic apoptotic signaling pathway in response to osmotic stress);GO:0071474(cellular hyperosmotic response);GO:0005515(protein binding);GO:0005634(nucleus);GO:0030529(ribonucleoprotein complex);GO:0000122(negative regulation of transcription from RNA polymerase II promoter);GO:0008584(male gonad development);GO:0001701(in utero embryonic development);GO:0043066(negative regulation of apoptotic process);GO:0007283(spermatogenesis);GO:0003697(single-stranded DNA binding);GO:0009566(fertilization);GO:0000977(RNA polymerase II regulatory region sequence-specific DNA binding);GO:0001227(RNA polymerase II transcription regulatory region sequence-specific DNA binding transcription factor activity involved in negative regulation of transcription);GO:0003730(mRNA 3'-UTR binding);GO:0005844(polysome);GO:0048642(negative regulation of skeletal muscle tissue development);GO:0046622(positive regulation of organ growth)
circRNA58	GO:0004190(aspartic-type endopeptidase activity);GO:0006508(proteolysis);GO:0005515(protein binding)
circRNA580	GO:0003824(catalytic activity);GO:0004252(serine-type endopeptidase activity);GO:0005509(calcium ion binding);GO:0005576(extracellular region);GO:0006508(proteolysis);GO:0006956(complement activation);GO:0008233(peptidase activity);GO:0016787(hydrolase activity);GO:0070062(extracellular vesicular exosome);GO:0005515(protein binding);GO:0072562(blood
circRNA581	()
circRNA582	GO:0005576(extracellular region);GO:0005515(protein binding);GO:0051260(protein homooligomerization);GO:0031012(extracellular matrix);GO:0005783(endoplasmic reticulum);GO:0070062(extracellular vesicular exosome);GO:0042803(protein homodimerization activity);GO:0002020(protease binding);GO:0047485(protein N-terminus binding);GO:0042802(identical protein binding);GO:0005518(collagen binding);GO:0051087(chaperone binding);GO:0033093(Weibel-Palade body);GO:0007599(hemostasis);GO:0019865(immunoglobulin binding);GO:0001948(glycoprotein binding);GO:0031589(cell-substrate adhesion);GO:0005178(integrin binding);GO:0009897(external side of plasma membrane);GO:0001890(placenta development);GO:0001889(liver development);GO:0007596(blood coagulation);GO:0030168(platelet activation)
circRNA583	GO:0003824(catalytic activity);GO:0050662(coenzyme binding);GO:0003854(3-beta-hydroxy-delta5-steroid dehydrogenase activity);GO:0016616("oxidoreductase activity, acting on the CH-OH group of donors, NAD or NADP as acceptor");GO:0006694(steroid biosynthetic process);GO:0055114(oxidation-reduction process);GO:0005739(mitochondrion);GO:0005743(mitochondrial inner
circRNA584	GO:0005515(protein binding);GO:0007252(I-kappaB phosphorylation);GO:0008385(IkappaB kinase complex);GO:0005737(cytoplasm);GO:0017137(Rab GTPase binding);GO:0045202(synapse);GO:0042147("retrograde transport, endosome to Golgi")
circRNA585	GO:0008152(metabolic process);GO:0008270(zinc ion binding);GO:0016491(oxidoreductase activity);GO:0046872(metal ion binding);GO:0055114(oxidation-reduction process);GO:0005515(protein binding);GO:0005737(cytoplasm);GO:0005634(nucleus);GO:0007010(cytoskeleton organization)
circRNA586	GO:0008152(metabolic process);GO:0008270(zinc ion binding);GO:0016491(oxidoreductase activity);GO:0046872(metal ion binding);GO:0055114(oxidation-reduction process);GO:0005515(protein binding);GO:0005737(cytoplasm);GO:0005634(nucleus);GO:0007010(cytoskeleton organization)
circRNA587	GO:0003824(catalytic activity);GO:0016829(lyase activity);GO:0004018("N6-(1,2-dicarboxyethyl)AMP AMP-lyase (fumarate-forming) activity");GO:0009152(purine ribonucleotide biosynthetic process);GO:0008152(metabolic process);GO:0006167(AMP biosynthetic process);GO:0051262(protein tetramerization);GO:0005739(mitochondrion)
circRNA588	GO:0008152(metabolic process);GO:0016491(oxidoreductase activity);GO:0055114(oxidation-reduction process);GO:0016020(membrane);GO:0005777(peroxisome);GO:0005739(mitochondrion)
circRNA589	GO:0008360(regulation of cell shape);GO:0016020(membrane);GO:0005515(protein binding)

circRNA59	GO:0005097(Rab GTPase activator activity);GO:0032313(regulation of Rab GTPase activity);GO:0032851(positive regulation of Rab GTPase activity);GO:0005515(protein binding);GO:0005634(nucleus);GO:0005794(Golgi apparatus);GO:0005769(early endosome);GO:0017137(Rab GTPase binding);GO:0032880(regulation of protein localization)
circRNA590	GO:0008360(regulation of cell shape);GO:0016020(membrane);GO:0005515(protein binding)
circRNA591	GO:0046872(metal ion binding);GO:0005634(nucleus);GO:0045944(positive regulation of transcription from RNA polymerase II promoter);GO:0043392(negative regulation of DNA binding);GO:0006325(cl
circRNA592	GO:0000166(nucleotide binding);GO:0005524(ATP binding);GO:0006200(ATP catabolic process);GO:0008152(metabolic process);GO:0016887(ATPase activity);GO:0017111(nucleoside-triphosphatase activity);GO:0007186(G-protein coupled receptor signaling pathway);GO:0016197(endosomal transport);GO:0048471(perinuclear region of cytoplasm);GO:0005887(integral component of plasma membrane);GO:0005515(protein binding);GO:0005886(plasma membrane);GO:0005102(receptor binding);GO:0045121(membrane raft);GO:0051117(ATPase binding);GO:0030139(endocytic vesicle);GO:0043691(reverse cholesterol transport);GO:0017127(cholesterol transporter activity);GO:0008203(cholesterol metabolic process);GO:0042632(cholesterol homeostasis);GO:0007040(lysosome organization);GO:0019905(syntaxin binding);GO:0060155(platelet dense granule organization);GO:0055098(response to low-density lipoprotein particle);GO:0055091(phospholipid homeostasis);GO:0038027(apolipoprotein A-I-mediated signaling pathway);GO:0034188(apolipoprotein A-I receptor activity);GO:0034186(apolipoprotein A-I binding);GO:0033700(phospholipid efflux);GO:0032489(regulation of Cdc42 protein signal transduction);GO:0031267(small GTPase binding);GO:0005548(phospholipid transporter activity);GO:0034380(high-density lipoprotein particle assembly);GO:0034185(apolipoprotein binding);GO:0032367(intracellular cholesterol transport);GO:0045335(phagocytic vesicle);GO:0030819(positive regulation of cAMP biosynthetic process);GO:0033344(cholesterol efflux);GO:0034616(response to laminar fluid shear stress);GO:0050702(interleukin-1 beta secretion);GO:0071222(cellular response to lipopolysaccharide);GO:0009897(external side of plasma membrane);GO:0005794(Golgi apparatus);GO:0043231(intracellular membrane-bounded organelle);GO:0006911("phagocytosis, engulfment");GO:0045332(phospholipid translocation);GO:0042157(lipoprotein metabolic process);GO:0071300(cellular response to retinoic acid);GO:0042158(lipoprotein biosynthetic process);GO:0010875(positive regulation of cholesterol efflux);GO:0006497(protein lipidation);GO:0002790(peptide secretion);GO:0008509(anion transmembrane transporter activity)
circRNA593	GO:0016020(membrane);GO:0005737(cytoplasm);GO:0044822(poly(A) RNA binding)
circRNA594	GO:0005515(protein binding);GO:0044822(poly(A) RNA binding)
circRNA595	GO:0051260(protein homooligomerization);GO:0005515(protein binding)
circRNA596	GO:0004721(phosphoprotein phosphatase activity);GO:0008152(metabolic process);GO:0016311(dephosphorylation);GO:0016787(hydrolase activity);GO:0070062(extracellular vesicular exosome)
circRNA597	GO:0005198(structural molecule activity);GO:0005581(collagen trimer);GO:0007155(cell adhesion);GO:0031012(extracellular matrix);GO:0005615(extracellular space);GO:0070062(extracellular vesicular exosome);GO:0005604(basement membrane)
circRNA598	GO:0001664(G-protein coupled receptor binding);GO:0003924(GTPase activity);GO:0004871(signal transducer activity);GO:0005525(GTP binding);GO:0006184(GTP catabolic process);GO:0007186(G-protein coupled receptor signaling pathway);GO:0019001(guanyl nucleotide binding);GO:0031683(G-protein beta/gamma-subunit complex binding);GO:0000166(nucleotide binding);GO:0007165(signal transduction);GO:0043547(positive regulation of GTPase activity);GO:0005096(GTPase activator activity);GO:0070062(extracellular vesicular exosome);GO:0005515(protein binding);GO:0006469(negative regulation of protein kinase activity);GO:0050821(protein stabilization);GO:0005765(lysosomal membrane);GO:0035412(regulation of catenin import into nucleus);GO:0016020(membrane);GO:0001501(skeletal system development);GO:0007189(adenylate cyclase-activating G-protein coupled receptor signaling pathway);GO:0009791(post-embryonic development);GO:0007507(heart development);GO:0042733(embryonic digit morphogenesis);GO:0001508(action potential);GO:0048066(developmental pigmentation);GO:0016322(neuron remodeling);GO:0021884(forebrain neuron development);GO:0042711(maternal behavior);GO:0045634(regulation of melanocyte differentiation);GO:0060158(phospholipase C-activating dopamine receptor signaling pathway);GO:0007215(glutamate receptor signaling pathway)
circRNA599	GO:0005085(guanyl-nucleotide exchange factor activity);GO:0007264(small GTPase mediated signal transduction);GO:0043547(positive regulation of GTPase activity);GO:0016020(membrane);GO:0005515(protein binding);GO:0061485(memory T cell proliferation);GO:0031252(cell leading edge);GO:0070233(negative regulation of T cell apoptotic
circRNA6	GO:0003677(DNA binding);GO:0005622(intracellular);GO:0005634(nucleus);GO:0008270(zinc ion binding);GO:0016706("oxidoreductase activity, acting on paired donors, with incorporation or reduction of molecular oxygen, 2-oxoglutarate as one donor, and incorporation of one atom each of oxygen into both donors");GO:0046872(metal ion binding);GO:0055114(oxidation-reduction process);GO:0005515(protein binding);GO:0034720(histone H3-K4 demethylation);GO:0032453(histone demethylase activity (H3-K4 specific))
circRNA60	GO:0008270(zinc ion binding);GO:0046872(metal ion binding);GO:0005515(protein binding);GO:0005813(centrosome);GO:0005737(cytoplasm);GO:0005634(nucleus);GO:0005925(focal adhesion)
circRNA600	GO:0003677(DNA binding);GO:0005634(nucleus);GO:0006355("regulation of transcription, DNA-templated");GO:0045893("positive regulation of transcription, DNA-templated");GO:0005515(protein binding);GO:0045892("negative regulation of transcription, DNA-templated");GO:0000790(nuclear chromatin);GO:0044212(transcription regulatory region DNA binding);GO:0003700(sequence-specific
circRNA601	GO:0005515(protein binding)
circRNA602	GO:0005634(nucleus);GO:0008270(zinc ion binding);GO:0046872(metal ion binding);GO:0005515(protein binding);GO:0019899(enzyme binding);GO:0050681(androgen receptor binding)
circRNA603	GO:0005515(protein binding);GO:0005737(cytoplasm);GO:0008022(protein C-terminus binding);GO:0016327(apicolateral plasma membrane);GO:0031410(cytoplasmic vesicle);GO:0005923(tight junction);GO:0007155(cell adhesion);GO:0043220(Schmidt-Lanterman incisure);GO:0016324(apical plasma membrane)
circRNA604	GO:0003677(DNA binding);GO:0003700(sequence-specific DNA binding transcription factor activity);GO:0005622(intracellular);GO:0005634(nucleus);GO:0006260(DNA replication);GO:0006351("transcription, DNA-templated");GO:0006355("regulation of transcription, DNA-templated");GO:0005730(nucleolus);GO:0006366(transcription from RNA polymerase II promoter);GO:0045944(positive regulation of transcription from RNA polymerase II promoter);GO:0000978(RNA polymerase II core promoter proximal region sequence-specific DNA binding);GO:0000122(negative regulation of transcription from RNA polymerase II promoter);GO:0000981(sequence-specific DNA binding RNA polymerase II transcription factor activity);GO:0043392(negative regulation of DNA binding);GO:0001106(RNA polymerase II transcription corepressor activity);GO:0001077(RNA polymerase II core promoter proximal region sequence-specific DNA binding transcription factor activity involved in positive regulation of transcription);GO:0002062(chondrocyte differentiation);GO:0008285(negative regulation of cell proliferation);GO:0030900(forebrain development);GO:0030902(hindbrain development);GO:0030324(lung development);GO:0010001(glial cell differentiation);GO:0060509(Type I pneumocyte differentiation);GO:0044300(cerebellar mossy fiber);GO:0021960(anterior commissure morphogenesis);GO:0060510(Type II pneumocyte differentiation);GO:0060486(Clara cell differentiation);GO:2000791(negative regulation of mesenchymal cell proliferation involved in lung development);GO:0021740(principal sensory nucleus of trigeminal nerve development);GO:0071679(commissural neuron axon guidance);GO:0061141(lung ciliated cell differentiation);GO:2000795(negative regulation of epithelial cell proliferation involved in lung morphogenesis)
circRNA605	GO:0046872(metal ion binding);GO:0005737(cytoplasm);GO:0005886(plasma membrane);GO:0005634(nucleus);GO:0003677(DNA binding);GO:0043586(tongue development);GO:0060021(palate development);GO:0003416(endochondral bone growth);GO:0060485(mesenchyme development)
circRNA606	GO:0015012(heparan sulfate proteoglycan biosynthetic process);GO:0016021(integral component of membrane);GO:0016758("transferase activity, transferring hexosyl groups");GO:0031227(intrinsic component of endoplasmic reticulum membrane);GO:0005783(endoplasmic reticulum)
circRNA607	GO:0005794(Golgi apparatus);GO:0006486(protein glycosylation);GO:0016020(membrane);GO:0016021(integral component of membrane);GO:0016740(transferase activity);GO:0016757("transferase activity, transferring glycosyl groups");GO:0006493(protein O-linked glycosylation);GO:0004653(polypeptide N-acetylgalactosaminyltransferase activity)
circRNA608	GO:0005515(protein binding);GO:0005634(nucleus);GO:0005884(actin filament)
circRNA609	GO:0005515(protein binding);GO:0005634(nucleus);GO:0005884(actin filament)
circRNA61	GO:0005515(protein binding);GO:0022617(extracellular matrix disassembly);GO:0031012(extracellular matrix);GO:0070062(extracellular vesicular exosome);GO:0005604(basement membrane);GO:0007155(cell adhesion);GO:0006461(protein complex assembly);GO:0031581(hemidesmosome assembly);GO:0034446(substrate adhesion-dependent cell spreading);GO:0016477(cell
circRNA610	GO:0005215(transporter activity);GO:0005886(plasma membrane);GO:0006810(transport);GO:0016020(membrane);GO:0016021(integral component of membrane);GO:0055085(transmembrane transport)
circRNA611	GO:0005634(nucleus);GO:0005643(nuclear pore);GO:0006810(transport);GO:0015031(protein transport);GO:0031965(nuclear membrane);GO:0051028(mRNA transport);GO:0055085(transmembrane transport);GO:0045111(intermediate filament cytoskeleton);GO:0043231(intracellular membrane-bounded organelle);GO:0005886(plasma membrane)
circRNA612	GO:0003676(nucleic acid binding);GO:0008270(zinc ion binding);GO:0046872(metal ion binding);GO:0005634(nucleus);GO:0072332(intrinsic apoptotic signaling pathway by p53 class mediator);GO:0002039(p53 binding)
circRNA613	GO:0080025("phosphatidylinositol-3,5-bisphosphate binding");GO:0032266(phosphatidylinositol-3-phosphate binding);GO:0010314(phosphatidylinositol-5-phosphate binding);GO:0005515(protein binding);GO:0005546("phosphatidylinositol-4,5-bisphosphate binding");GO:0070300(phosphatidic acid binding);GO:0070273(phosphatidylinositol-4-phosphate binding);GO:0043325("phosphatidylinositol-3,4-bisphosphate binding");GO:0034704(calcium channel complex)
circRNA614	GO:0080025("phosphatidylinositol-3,5-bisphosphate binding");GO:0032266(phosphatidylinositol-3-phosphate binding);GO:0010314(phosphatidylinositol-5-phosphate binding);GO:0005515(protein binding);GO:0005546("phosphatidylinositol-4,5-bisphosphate binding");GO:0070300(phosphatidic acid binding);GO:0070273(phosphatidylinositol-4-phosphate binding);GO:0043325("phosphatidylinositol-3,4-bisphosphate binding");GO:0034704(calcium channel complex)
circRNA615	GO:0004672(protein kinase activity);GO:0004713(protein tyrosine kinase activity);GO:0005524(ATP binding);GO:0006468(protein phosphorylation);GO:0016772("transferase activity, transferring phosphorus-containing groups");GO:0018108(peptidyl-tyrosine phosphorylation);GO:0005515(protein binding);GO:0003676(nucleic acid binding);GO:0004674(protein serine/threonine kinase activity);GO:0070062(extracellular vesicular exosome);GO:0005509(calcium ion binding);GO:0005737(cytoplasm);GO:0005634(nucleus);GO:0005794(Golgi apparatus);GO:0050790(regulation of catalytic activity);GO:0051015(actin filament binding);GO:0030018(Z disc);GO:0019899(enzyme binding);GO:0002020(protease binding);GO:0042802(identical protein binding);GO:0007076(mitotic chromosome condensation);GO:0045859(regulation of protein kinase activity);GO:0019901(protein kinase binding);GO:0031674(I band);GO:0031430(M band);GO:0051371(muscle alpha-actinin binding);GO:0005865(striated muscle thin filament);GO:0051592(response to calcium ion);GO:0042805(actinin binding);GO:0055008(cardiac muscle tissue morphogenesis);GO:0055003(cardiac myofibril assembly);GO:0048769(sarcomerogenesis);GO:0048739(cardiac muscle fiber development);GO:0045214(sarcomere organization);GO:0030241(skeletal muscle myosin thick filament assembly);GO:0030240(skeletal muscle thin filament assembly);GO:0003300(cardiac muscle hypertrophy);GO:0008307(structural constituent of muscle);GO:0060048(cardiac muscle contraction);GO:0000794(condensed nuclear chromosome);GO:0043621(protein self-association);GO:0001701(in utero embryonic development);GO:0031672(A band);GO:0006936(muscle contraction);GO:0007507(heart development);GO:0001756(somitogenesis);GO:0003007(heart morphogenesis);GO:0007512(adult heart development);GO:0030506(ankyrin binding);GO:1901897(regulation of relaxation of cardiac muscle);GO:0030017(sarcomere);GO:0043056(forward locomotion);GO:0055002(striated muscle cell development)

circRNA616	GO:0004672(protein kinase activity);GO:0004713(protein tyrosine kinase activity);GO:0005524(ATP binding);GO:0006468(protein phosphorylation);GO:0016772("transferase activity, transferring phosphorus-containing groups");GO:0018108(peptidyl-tyrosine phosphorylation);GO:0005515(protein binding);GO:0003676(nucleic acid binding);GO:0004674(protein serine/threonine kinase activity);GO:0070062(extracellular vesicular exosome);GO:0005509(calcium ion binding);GO:0005737(cytoplasm);GO:0005634(nucleus);GO:0005794(Golgi apparatus);GO:0050790(regulation of catalytic activity);GO:0051015(actin filament binding);GO:0030018(Z disc);GO:0019899(enzyme binding);GO:0002020(protease binding);GO:0042802(identical protein binding);GO:0007076(mitotic chromosome condensation);GO:0045859(regulation of protein kinase activity);GO:0019901(protein kinase binding);GO:0031674(I band);GO:0031430(M band);GO:0051371(muscle alpha-actinin binding);GO:0005865(striated muscle thin filament);GO:0051592(response to calcium ion);GO:0042805(actinin binding);GO:0055008(cardiac muscle tissue morphogenesis);GO:0055003(cardiac myofibril assembly);GO:0048769(sarcomerogenesis);GO:0048739(cardiac muscle fiber development);GO:0045214(sarcomere organization);GO:0030241(skeletal muscle myosin thick filament assembly);GO:0030240(skeletal muscle thin filament assembly);GO:0003300(cardiac muscle hypertrophy);GO:0008307(structural constituent of muscle);GO:0060048(cardiac muscle contraction);GO:0000794(condensed nuclear chromosome);GO:0043621(protein self-association);GO:0001701(in utero embryonic development);GO:0031672(A band);GO:0006936(muscle contraction);GO:0007507(heart development);GO:0001756(somitogenesis);GO:0003007(heart morphogenesis);GO:0007512(adult heart development);GO:0030506(ankyrin binding);GO:1901897(regulation of relaxation of cardiac muscle);GO:0030017(sarcomere);GO:0043056(forward locomotion);GO:0055002(striated muscle cell development)
circRNA617	GO:0004672(protein kinase activity);GO:0004713(protein tyrosine kinase activity);GO:0005524(ATP binding);GO:0006468(protein phosphorylation);GO:0016772("transferase activity, transferring phosphorus-containing groups");GO:0018108(peptidyl-tyrosine phosphorylation);GO:0005515(protein binding);GO:0003676(nucleic acid binding);GO:0004674(protein serine/threonine kinase activity);GO:0070062(extracellular vesicular exosome);GO:0005509(calcium ion binding);GO:0005737(cytoplasm);GO:0005634(nucleus);GO:0005794(Golgi apparatus);GO:0050790(regulation of catalytic activity);GO:0051015(actin filament binding);GO:0030018(Z disc);GO:0019899(enzyme binding);GO:0002020(protease binding);GO:0042802(identical protein binding);GO:0007076(mitotic chromosome condensation);GO:0045859(regulation of protein kinase activity);GO:0019901(protein kinase binding);GO:0031674(I band);GO:0031430(M band);GO:0051371(muscle alpha-actinin binding);GO:0005865(striated muscle thin filament);GO:0051592(response to calcium ion);GO:0042805(actinin binding);GO:0055008(cardiac muscle tissue morphogenesis);GO:0055003(cardiac myofibril assembly);GO:0048769(sarcomerogenesis);GO:0048739(cardiac muscle fiber development);GO:0045214(sarcomere organization);GO:0030241(skeletal muscle myosin thick filament assembly);GO:0030240(skeletal muscle thin filament assembly);GO:0003300(cardiac muscle hypertrophy);GO:0008307(structural constituent of muscle);GO:0060048(cardiac muscle contraction);GO:0000794(condensed nuclear chromosome);GO:0043621(protein self-association);GO:0001701(in utero embryonic development);GO:0031672(A band);GO:0006936(muscle contraction);GO:0007507(heart development);GO:0001756(somitogenesis);GO:0003007(heart morphogenesis);GO:0007512(adult heart development);GO:0030506(ankyrin binding);GO:1901897(regulation of relaxation of cardiac muscle);GO:0030017(sarcomere);GO:0043056(forward locomotion);GO:0055002(striated muscle cell development)
circRNA618	GO:0004672(protein kinase activity);GO:0004713(protein tyrosine kinase activity);GO:0005524(ATP binding);GO:0006468(protein phosphorylation);GO:0016772("transferase activity, transferring phosphorus-containing groups");GO:0018108(peptidyl-tyrosine phosphorylation);GO:0005515(protein binding);GO:0003676(nucleic acid binding);GO:0004674(protein serine/threonine kinase activity);GO:0070062(extracellular vesicular exosome);GO:0005509(calcium ion binding);GO:0005737(cytoplasm);GO:0005634(nucleus);GO:0005794(Golgi apparatus);GO:0050790(regulation of catalytic activity);GO:0051015(actin filament binding);GO:0030018(Z disc);GO:0019899(enzyme binding);GO:0002020(protease binding);GO:0042802(identical protein binding);GO:0007076(mitotic chromosome condensation);GO:0045859(regulation of protein kinase activity);GO:0019901(protein kinase binding);GO:0031674(I band);GO:0031430(M band);GO:0051371(muscle alpha-actinin binding);GO:0005865(striated muscle thin filament);GO:0051592(response to calcium ion);GO:0042805(actinin binding);GO:0055008(cardiac muscle tissue morphogenesis);GO:0055003(cardiac myofibril assembly);GO:0048769(sarcomerogenesis);GO:0048739(cardiac muscle fiber development);GO:0045214(sarcomere organization);GO:0030241(skeletal muscle myosin thick filament assembly);GO:0030240(skeletal muscle thin filament assembly);GO:0003300(cardiac muscle hypertrophy);GO:0008307(structural constituent of muscle);GO:0060048(cardiac muscle contraction);GO:0000794(condensed nuclear chromosome);GO:0043621(protein self-association);GO:0001701(in utero embryonic development);GO:0031672(A band);GO:0006936(muscle contraction);GO:0007507(heart development);GO:0001756(somitogenesis);GO:0003007(heart morphogenesis);GO:0007512(adult heart development);GO:0030506(ankyrin binding);GO:1901897(regulation of relaxation of cardiac muscle);GO:0030017(sarcomere);GO:0043056(forward locomotion);GO:0055002(striated muscle cell development)
circRNA619	GO:0004672(protein kinase activity);GO:0004713(protein tyrosine kinase activity);GO:0005524(ATP binding);GO:0006468(protein phosphorylation);GO:0016772("transferase activity, transferring phosphorus-containing groups");GO:0018108(peptidyl-tyrosine phosphorylation);GO:0005515(protein binding);GO:0003676(nucleic acid binding);GO:0004674(protein serine/threonine kinase activity);GO:0070062(extracellular vesicular exosome);GO:0005509(calcium ion binding);GO:0005737(cytoplasm);GO:0005634(nucleus);GO:0005794(Golgi apparatus);GO:0050790(regulation of catalytic activity);GO:0051015(actin filament binding);GO:0030018(Z disc);GO:0019899(enzyme binding);GO:0002020(protease binding);GO:0042802(identical protein binding);GO:0007076(mitotic chromosome condensation);GO:0045859(regulation of protein kinase activity);GO:0019901(protein kinase binding);GO:0031674(I band);GO:0031430(M band);GO:0051371(muscle alpha-actinin binding);GO:0005865(striated muscle thin filament);GO:0051592(response to calcium ion);GO:0042805(actinin binding);GO:0055008(cardiac muscle tissue morphogenesis);GO:0055003(cardiac myofibril assembly);GO:0048769(sarcomerogenesis);GO:0048739(cardiac muscle fiber development);GO:0045214(sarcomere organization);GO:0030241(skeletal muscle myosin thick filament assembly);GO:0030240(skeletal muscle thin filament assembly);GO:0003300(cardiac muscle hypertrophy);GO:0008307(structural constituent of muscle);GO:0060048(cardiac muscle contraction);GO:0000794(condensed nuclear chromosome);GO:0043621(protein self-association);GO:0001701(in utero embryonic development);GO:0031672(A band);GO:0006936(muscle contraction);GO:0007507(heart development);GO:0001756(somitogenesis);GO:0003007(heart morphogenesis);GO:0007512(adult heart development);GO:0030506(ankyrin binding);GO:1901897(regulation of relaxation of cardiac muscle);GO:0030017(sarcomere);GO:0043056(forward locomotion);GO:0055002(striated muscle cell development)
circRNA62	GO:0009103(lipopolysaccharide biosynthetic process)
circRNA620	GO:0004672(protein kinase activity);GO:0004713(protein tyrosine kinase activity);GO:0005524(ATP binding);GO:0006468(protein phosphorylation);GO:0016772("transferase activity, transferring phosphorus-containing groups");GO:0018108(peptidyl-tyrosine phosphorylation);GO:0005515(protein binding);GO:0003676(nucleic acid binding);GO:0004674(protein serine/threonine kinase activity);GO:0070062(extracellular vesicular exosome);GO:0005509(calcium ion binding);GO:0005737(cytoplasm);GO:0005634(nucleus);GO:0005794(Golgi apparatus);GO:0050790(regulation of catalytic activity);GO:0051015(actin filament binding);GO:0030018(Z disc);GO:0019899(enzyme binding);GO:0002020(protease binding);GO:0042802(identical protein binding);GO:0007076(mitotic chromosome condensation);GO:0045859(regulation of protein kinase activity);GO:0019901(protein kinase binding);GO:0031674(I band);GO:0031430(M band);GO:0051371(muscle alpha-actinin binding);GO:0005865(striated muscle thin filament);GO:0051592(response to calcium ion);GO:0042805(actinin binding);GO:0055008(cardiac muscle tissue morphogenesis);GO:0055003(cardiac myofibril assembly);GO:0048769(sarcomerogenesis);GO:0048739(cardiac muscle fiber development);GO:0045214(sarcomere organization);GO:0030241(skeletal muscle myosin thick filament assembly);GO:0030240(skeletal muscle thin filament assembly);GO:0003300(cardiac muscle hypertrophy);GO:0008307(structural constituent of muscle);GO:0060048(cardiac muscle contraction);GO:0000794(condensed nuclear chromosome);GO:0043621(protein self-association);GO:0001701(in utero embryonic development);GO:0031672(A band);GO:0006936(muscle contraction);GO:0007507(heart development);GO:0001756(somitogenesis);GO:0003007(heart morphogenesis);GO:0007512(adult heart development);GO:0030506(ankyrin binding);GO:1901897(regulation of relaxation of cardiac muscle);GO:0030017(sarcomere);GO:0043056(forward locomotion);GO:0055002(striated muscle cell development)
circRNA621	GO:0004672(protein kinase activity);GO:0004713(protein tyrosine kinase activity);GO:0005524(ATP binding);GO:0006468(protein phosphorylation);GO:0016772("transferase activity, transferring phosphorus-containing groups");GO:0018108(peptidyl-tyrosine phosphorylation);GO:0005515(protein binding);GO:0003676(nucleic acid binding);GO:0004674(protein serine/threonine kinase activity);GO:0070062(extracellular vesicular exosome);GO:0005509(calcium ion binding);GO:0005737(cytoplasm);GO:0005634(nucleus);GO:0005794(Golgi apparatus);GO:0050790(regulation of catalytic activity);GO:0051015(actin filament binding);GO:0030018(Z disc);GO:0019899(enzyme binding);GO:0002020(protease binding);GO:0042802(identical protein binding);GO:0007076(mitotic chromosome condensation);GO:0045859(regulation of protein kinase activity);GO:0019901(protein kinase binding);GO:0031674(I band);GO:0031430(M band);GO:0051371(muscle alpha-actinin binding);GO:0005865(striated muscle thin filament);GO:0051592(response to calcium ion);GO:0042805(actinin binding);GO:0055008(cardiac muscle tissue morphogenesis);GO:0055003(cardiac myofibril assembly);GO:0048769(sarcomerogenesis);GO:0048739(cardiac muscle fiber development);GO:0045214(sarcomere organization);GO:0030241(skeletal muscle myosin thick filament assembly);GO:0030240(skeletal muscle thin filament assembly);GO:0003300(cardiac muscle hypertrophy);GO:0008307(structural constituent of muscle);GO:0060048(cardiac muscle contraction);GO:0000794(condensed nuclear chromosome);GO:0043621(protein self-association);GO:0001701(in utero embryonic development);GO:0031672(A band);GO:0006936(muscle contraction);GO:0007507(heart development);GO:0001756(somitogenesis);GO:0003007(heart morphogenesis);GO:0007512(adult heart development);GO:0030506(ankyrin binding);GO:1901897(regulation of relaxation of cardiac muscle);GO:0030017(sarcomere);GO:0043056(forward locomotion);GO:0055002(striated muscle cell development)
circRNA622	GO:0004672(protein kinase activity);GO:0004713(protein tyrosine kinase activity);GO:0005524(ATP binding);GO:0006468(protein phosphorylation);GO:0016772("transferase activity, transferring phosphorus-containing groups");GO:0018108(peptidyl-tyrosine phosphorylation);GO:0005515(protein binding);GO:0003676(nucleic acid binding);GO:0004674(protein serine/threonine kinase activity);GO:0070062(extracellular vesicular exosome);GO:0005509(calcium ion binding);GO:0005737(cytoplasm);GO:0005634(nucleus);GO:0005794(Golgi apparatus);GO:0050790(regulation of catalytic activity);GO:0051015(actin filament binding);GO:0030018(Z disc);GO:0019899(enzyme binding);GO:0002020(protease binding);GO:0042802(identical protein binding);GO:0007076(mitotic chromosome condensation);GO:0045859(regulation of protein kinase activity);GO:0019901(protein kinase binding);GO:0031674(I band);GO:0031430(M band);GO:0051371(muscle alpha-actinin binding);GO:0005865(striated muscle thin filament);GO:0051592(response to calcium ion);GO:0042805(actinin binding);GO:0055008(cardiac muscle tissue morphogenesis);GO:0055003(cardiac myofibril assembly);GO:0048769(sarcomerogenesis);GO:0048739(cardiac muscle fiber development);GO:0045214(sarcomere organization);GO:0030241(skeletal muscle myosin thick filament assembly);GO:0030240(skeletal muscle thin filament assembly);GO:0003300(cardiac muscle hypertrophy);GO:0008307(structural constituent of muscle);GO:0060048(cardiac muscle contraction);GO:0000794(condensed nuclear chromosome);GO:0043621(protein self-association);GO:0001701(in utero embryonic development);GO:0031672(A band);GO:0006936(muscle contraction);GO:0007507(heart development);GO:0001756(somitogenesis);GO:0003007(heart morphogenesis);GO:0007512(adult heart development);GO:0030506(ankyrin binding);GO:1901897(regulation of relaxation of cardiac muscle);GO:0030017(sarcomere);GO:0043056(forward locomotion);GO:0055002(striated muscle cell development)
circRNA623	GO:0004672(protein kinase activity);GO:0004713(protein tyrosine kinase activity);GO:0005524(ATP binding);GO:0006468(protein phosphorylation);GO:0016772("transferase activity, transferring phosphorus-containing groups");GO:0018108(peptidyl-tyrosine phosphorylation);GO:0005515(protein binding);GO:0003676(nucleic acid binding);GO:0004674(protein serine/threonine kinase activity);GO:0070062(extracellular vesicular exosome);GO:0005509(calcium ion binding);GO:0005737(cytoplasm);GO:0005634(nucleus);GO:0005794(Golgi apparatus);GO:0050790(regulation of catalytic activity);GO:0051015(actin filament binding);GO:0030018(Z disc);GO:0019899(enzyme binding);GO:0002020(protease binding);GO:0042802(identical protein binding);GO:0007076(mitotic chromosome condensation);GO:0045859(regulation of protein kinase activity);GO:0019901(protein kinase binding);GO:0031674(I band);GO:0031430(M band);GO:0051371(muscle alpha-actinin binding);GO:0005865(striated muscle thin filament);GO:0051592(response to calcium ion);GO:0042805(actinin binding);GO:0055008(cardiac muscle tissue morphogenesis);GO:0055003(cardiac myofibril assembly);GO:0048769(sarcomerogenesis);GO:0048739(cardiac muscle fiber development);GO:0045214(sarcomere organization);GO:0030241(skeletal muscle myosin thick filament assembly);GO:0030240(skeletal muscle thin filament assembly);GO:0003300(cardiac muscle hypertrophy);GO:0008307(structural constituent of muscle);GO:0060048(cardiac muscle contraction);GO:0000794(condensed nuclear chromosome);GO:0043621(protein self-association);GO:0001701(in utero embryonic development);GO:0031672(A band);GO:0006936(muscle contraction);GO:0007507(heart development);GO:0001756(somitogenesis);GO:0003007(heart morphogenesis);GO:0007512(adult heart development);GO:0030506(ankyrin binding);GO:1901897(regulation of relaxation of cardiac muscle);GO:0030017(sarcomere);GO:0043056(forward locomotion);GO:0055002(striated muscle cell development)

circRNA624	GO:0004672(protein kinase activity);GO:0004713(protein tyrosine kinase activity);GO:0005524(ATP binding);GO:0006468(protein phosphorylation);GO:0016772("transferase activity, transferring phosphorus-containing groups");GO:0018108(peptidyl-tyrosine phosphorylation);GO:0005515(protein binding);GO:0003676(nucleic acid binding);GO:0004674(protein serine/threonine kinase activity);GO:0070062(extracellular vesicular exosome);GO:0005509(calcium ion binding);GO:0005737(cytoplasm);GO:0005634(nucleus);GO:0005794(Golgi apparatus);GO:0050790(regulation of catalytic activity);GO:0051015(actin filament binding);GO:0030018(Z disc);GO:0019899(enzyme binding);GO:0002020(protease binding);GO:0042802(identical protein binding);GO:0007076(mitotic chromosome condensation);GO:0045859(regulation of protein kinase activity);GO:0019901(protein kinase binding);GO:0031674(I band);GO:0031430(M band);GO:0051371(muscle alpha-actinin binding);GO:0005865(striated muscle thin filament);GO:0051592(response to calcium ion);GO:0042805(actinin binding);GO:0055008(cardiac muscle tissue morphogenesis);GO:0055003(cardiac myofibril assembly);GO:0048769(sarcomerogenesis);GO:0048739(cardiac muscle fiber development);GO:0045214(sarcomere organization);GO:0030241(skeletal muscle myosin thick filament assembly);GO:0030240(skeletal muscle thin filament assembly);GO:0003300(cardiac muscle hypertrophy);GO:0008307(structural constituent of muscle);GO:0060048(cardiac muscle contraction);GO:0000794(condensed nuclear chromosome);GO:0043621(protein self-association);GO:0001701(in utero embryonic development);GO:0031672(A band);GO:0006936(muscle contraction);GO:0007507(heart development);GO:0001756(somitogenesis);GO:0003007(heart morphogenesis);GO:0007512(adult heart development);GO:0030506(ankyrin binding);GO:1901897(regulation of relaxation of cardiac muscle);GO:0030017(sarcomere);GO:0043056(forward locomotion);GO:0055002(striated muscle cell development)
circRNA625	GO:0016020(membrane);GO:0005737(cytoplasm);GO:0005515(protein binding);GO:0044822(poly(A) RNA binding);GO:0042803(protein homodimerization activity);GO:0030422(production of siRNA involved in RNA interference);GO:0006468(protein phosphorylation);GO:0005622(intracellular);GO:0034599(cellular response to oxidative stress);GO:0048705(skeletal system morphogenesis);GO:2001244(positive regulation of intrinsic apoptotic signaling pathway);GO:0042474(middle ear morphogenesis);GO:0042473(outer ear morphogenesis)
circRNA626	GO:0000166(nucleotide binding);GO:0004672(protein kinase activity);GO:0004674(protein serine/threonine kinase activity);GO:0005524(ATP binding);GO:0006468(protein phosphorylation);GO:0016772("transferase activity, transferring phosphorus-containing groups");GO:0005515(protein binding);GO:0004713(protein tyrosine kinase activity);GO:0000287(magnesium ion binding);GO:0044822(poly(A) RNA binding);GO:0043065(positive regulation of apoptotic process);GO:0035556(intracellular signal transduction);GO:0007050(cell cycle arrest);GO:0004709(MAP kinase kinase kinase activity);GO:0000077(DNA damage checkpoint);GO:0009314(response to radiation);GO:0000075(cell cycle checkpoint);GO:0007257(activation of JUN kinase activity);GO:0005737(cytoplasm);GO:0005634(nucleus);GO:0007010(cytoskeleton organization);GO:0000186(activation of MAPKK activity)
circRNA627	GO:0000293(ferric-chelate reductase activity);GO:0010039(response to iron ion);GO:0016021(integral component of membrane);GO:0031526(brush border membrane);GO:0055114(oxidation-reduction process);GO:0070062(extracellular vesicular exosome)
circRNA628	GO:0080008(Cul4-RING E3 ubiquitin ligase complex)
circRNA629	GO:0004842(ubiquitin-protein transferase activity);GO:0008270(zinc ion binding);GO:0016567(protein ubiquitination);GO:0005515(protein binding);GO:0007608(sensory perception of smell);GO:0006511(ubiquitin-dependent protein catabolic process);GO:0001701(in utero embryonic development);GO:0009790(embryo development);GO:0001967(suckling behavior);GO:0042048(olfactory
circRNA63	GO:0005515(protein binding)
circRNA630	GO:0003779(actin binding);GO:0007010(cytoskeleton organization);GO:0016043(cellular component organization);GO:0017048(Rho GTPase binding);GO:0022604(regulation of cell morphogenesis);GO:0030036(actin cytoskeleton organization);GO:0005488(binding);GO:0030866(cortical actin cytoskeleton organization)
circRNA631	GO:0003779(actin binding);GO:0007010(cytoskeleton organization);GO:0016043(cellular component organization);GO:0017048(Rho GTPase binding);GO:0022604(regulation of cell morphogenesis);GO:0030036(actin cytoskeleton organization);GO:0005488(binding);GO:0030866(cortical actin cytoskeleton organization)
circRNA632	GO:0006886(intracellular protein transport);GO:0005515(protein binding);GO:0043231(intracellular membrane-bounded organelle);GO:0005737(cytoplasm);GO:0005634(nucleus)
circRNA633	GO:0003779(actin binding);GO:0005515(protein binding);GO:0030018(Z disc);GO:0043292(contractile fiber)
circRNA634	GO:0000166(nucleotide binding);GO:0004672(protein kinase activity);GO:0004674(protein serine/threonine kinase activity);GO:0004702(receptor signaling protein serine/threonine kinase activity);GO:0005524(ATP binding);GO:0006468(protein phosphorylation);GO:0007178(transmembrane receptor protein serine/threonine kinase signaling pathway);GO:0016020(membrane);GO:0016021(integral component of membrane);GO:0016301(kinase activity);GO:0016310(phosphorylation);GO:0016740(transferase activity);GO:0016772("transferase activity, transferring phosphorus-containing groups");GO:0023014(signal transduction by phosphorylation);GO:0004675(transmembrane receptor protein serine/threonine kinase activity);GO:0005024(transforming growth factor beta-activated receptor activity);GO:0004713(protein tyrosine kinase activity);GO:0005737(cytoplasm);GO:0005515(protein binding);GO:0015026(coreceptor activity);GO:0045669(positive regulation of osteoblast differentiation);GO:0030509(BMP signaling pathway);GO:0030501(positive regulation of bone mineralization);GO:0001934(positive regulation of protein phosphorylation);GO:0017002(activin-activated receptor activity);GO:0032927(positive regulation of activin receptor signaling pathway);GO:0034673(inhibin-betaglycan-ActRII complex);GO:0071773(cellular response to BMP stimulus);GO:0032924(activin receptor signaling pathway);GO:0010862(positive regulation of pathway-restricted SMAD protein phosphorylation);GO:0045648(positive regulation of erythrocyte differentiation);GO:0048185(activin binding);GO:0009986(cell surface);GO:0008584(male gonad development);GO:0009952(anterior/posterior pattern specification);GO:0007283(spermatogenesis);GO:0030165(PDZ domain binding);GO:0048706(embryonic skeletal system development);GO:0007368(determination of left/right symmetry);GO:0043621(protein self-association);GO:0009966(regulation of signal transduction);GO:0019838(growth factor binding);GO:0001702(gastrulation with mouth forming second);GO:0007498(mesoderm development);GO:0030510(regulation of BMP signaling pathway);GO:0060011(Sertoli cell proliferation);GO:0034711(inhibin binding);GO:0042713(sperm ejaculation);GO:0043084(penile erection);GO:0050999(regulation of nitric-oxide synthase activity)
circRNA635	GO:0005097(Rab GTPase activator activity);GO:0032851(positive regulation of Rab GTPase activity);GO:0070062(extracellular vesicular exosome);GO:0005737(cytoplasm);GO:0021854(hypothalamus development);GO:0034389(lipid particle organization);GO:0043087(regulation of GTPase activity);GO:0017137(Rab GTPase binding);GO:0007420(brain development);GO:0043010(camera-type eye development);GO:0060325(face morphogenesis)
circRNA636	GO:0000166(nucleotide binding);GO:0003924(GTPase activity);GO:0005525(GTP binding);GO:0005886(plasma membrane);GO:0006184(GTP catabolic process);GO:0007165(signal transduction);GO:0007264(small GTPase mediated signal transduction);GO:0007265(Ras protein signal transduction);GO:0016020(membrane);GO:0006886(intracellular protein transport);GO:0006913(nucleocytoplasmic transport);GO:0005622(intracellular);GO:0015031(protein transport);GO:0070062(extracellular vesicular exosome);GO:0005515(protein binding);GO:0000910(cytokinesis);GO:0030496(midbody)
circRNA637	GO:0005828(kinetochore microtubule);GO:0005881(cytoplasmic microtubule);GO:0043515(kinetochore binding);GO:0051010(microtubule plus-end binding);GO:0005515(protein binding);GO:0005488(binding);GO:0008017(microtubule binding);GO:0005813(centrosome);GO:0070062(extracellular vesicular exosome);GO:0016020(membrane);GO:0000776(kinetochore);GO:0005938(cell cortex);GO:0005794(Golgi apparatus);GO:0034453(microtubule anchoring);GO:0010458(exit from mitosis);GO:0007020(microtubule nucleation);GO:0005876(spindle microtubule);GO:0031023(microtubule organizing center organization);GO:0007026(negative regulation of microtubule depolymerization);GO:0000226(microtubule cytoskeleton organization);GO:0051301(cell division);GO:0031592(centrosomal corona);GO:0030981(cortical microtubule cytoskeleton);GO:0031111(negative regulation of microtubule polymerization or depolymerization);GO:0051294(establishment of spindle orientation);GO:0001578(microtubule bundle formation)
circRNA638	GO:0005828(kinetochore microtubule);GO:0005881(cytoplasmic microtubule);GO:0043515(kinetochore binding);GO:0051010(microtubule plus-end binding);GO:0005515(protein binding);GO:0005488(binding);GO:0008017(microtubule binding);GO:0005813(centrosome);GO:0070062(extracellular vesicular
circRNA639	GO:0000166(nucleotide binding);GO:0003676(nucleic acid binding);GO:0005737(cytoplasm);GO:0005515(protein binding);GO:0045948(positive regulation of translational initiation);GO:0007126(meiotic nuclear division);GO:0008494(translation activator activity)
circRNA64	GO:0016020(membrane);GO:0016021(integral component of membrane);GO:0018345(protein palmitoylation);GO:0007224(smoothened signaling pathway);GO:0016409(palmitoyltransferase activity)
circRNA640	()
circRNA641	()
circRNA642	GO:0003995(acyl-CoA dehydrogenase activity);GO:0008152(metabolic process);GO:0016491(oxidoreductase activity);GO:0016627("oxidoreductase activity, acting on the CH-CH group of donors");GO:0050660(flavin adenine dinucleotide binding);GO:0055114(oxidation-reduction process);GO:0005739(mitochondrion);GO:0005737(cytoplasm);GO:0045717(negative regulation of fatty acid biosynthetic process);GO:0001659(temperature homeostasis);GO:0033539(fatty acid beta-oxidation using acyl-CoA dehydrogenase);GO:0090181(regulation of cholesterol metabolic process);GO:0019254("carnitine metabolic process, CoA-linked");GO:0044242(cellular lipid catabolic process);GO:0046322(negative regulation of fatty acid oxidation);GO:0004466(long-chain-acyl-CoA dehydrogenase activity);GO:0016401(palmitoyl-CoA oxidase activity);GO:0042413(carnitine catabolic process)
circRNA643	GO:0001558(regulation of cell growth);GO:0005520(insulin-like growth factor binding);GO:0005576(extracellular region);GO:0005515(protein binding);GO:0017148(negative regulation of translation);GO:0031994(insulin-like growth factor I binding);GO:0048662(negative regulation of smooth muscle cell proliferation);GO:0014912(negative regulation of smooth muscle cell migration);GO:0043569(negative regulation of insulin-like growth factor receptor signaling pathway);GO:0071320(cellular response to cAMP);GO:0071407(cellular response to organic cyclic compound);GO:0030336(negative regulation of cell migration);GO:0042593(glucose homeostasis);GO:0010906(regulation of glucose metabolic process);GO:0051897(positive regulation of protein kinase B signaling);GO:0045668(negative regulation of osteoblast differentiation);GO:0006006(glucose metabolic process);GO:0031069(hair follicle morphogenesis);GO:0043568(positive regulation of insulin-like growth factor receptor signaling pathway);GO:0001649(osteoblast differentiation);GO:0040008(regulation of growth);GO:0051146(striated muscle cell differentiation);GO:0060056(mammary gland involution);GO:0001968(fibronectin binding);GO:0044342(type B pancreatic cell proliferation)
circRNA644	GO:0004435(phosphatidylinositol phospholipase C activity);GO:0004629(phospholipase C activity);GO:0004871(signal transducer activity);GO:0005509(calcium ion binding);GO:0005622(intracellular);GO:0006629(lipid metabolic process);GO:0007165(signal transduction);GO:0007340(acrosome reaction);GO:0008081(phosphoric diester hydrolase activity);GO:0016042(lipid catabolic process);GO:0016787(hydrolase activity);GO:0035556(intracellular signal transduction);GO:0046488(phosphatidylinositol metabolic process);GO:0005515(protein binding)
circRNA645	GO:0004842(ubiquitin-protein transferase activity);GO:0008152(metabolic process);GO:0016567(protein ubiquitination);GO:0016874(ligase activity);GO:0005488(binding);GO:0008270(zinc ion binding);GO:0006974(cellular response to DNA damage stimulus);GO:0005515(protein binding);GO:0005654(nucleoplasm);GO:2000780(negative regulation of double-strand break repair);GO:1901315(negative regulation of histone H2A K63-linked ubiquitination);GO:0046966(thyroid hormone receptor binding);GO:0009790(embryo development)
circRNA646	GO:0000166(nucleotide binding);GO:0000287(magnesium ion binding);GO:0004672(protein kinase activity);GO:0004674(protein serine/threonine kinase activity);GO:0005524(ATP binding);GO:0006468(protein phosphorylation);GO:0016301(kinase activity);GO:0016310(phosphorylation);GO:0016740(transferase activity);GO:0016772("transferase activity, transferring phosphorus-containing groups");GO:0035556(intracellular signal transduction);GO:0016773("phosphotransferase activity, alcohol group as acceptor");GO:0009103(lipopolysaccharide biosynthetic process);GO:0016020(membrane);GO:0004713(protein tyrosine kinase activity);GO:0005737(cytoplasm);GO:0045944(positive regulation of transcription from RNA polymerase II promoter);GO:0005515(protein binding);GO:0005634(nucleus);GO:0043066(negative regulation of apoptotic process);GO:0043154(negative regulation of cysteine-type endopeptidase activity involved in apoptotic process);GO:0043027(cysteine-type endopeptidase inhibitor activity involved in apoptotic process);GO:0004712(protein serine/threonine/tyrosine kinase activity);GO:2000491(positive regulation of hepatic stellate cell activation);GO:0005819(spindle)
circRNA647	GO:0005622(intracellular);GO:0008270(zinc ion binding);GO:0046872(metal ion binding);GO:0005515(protein binding);GO:0031432(titin binding);GO:0010468(regulation of gene expression);GO:0004842(ubiquitin-protein transferase activity);GO:0043161(proteasome-mediated ubiquitin-dependent protein catabolic process);GO:0071549(cellular response to dexamethasone stimulus)
circRNA648	()

circRNA649	()
circRNA65	()
circRNA650	GO:0005515(protein binding);GO:0042803(protein homodimerization activity);GO:0031430(M band)
circRNA651	GO:0005515(protein binding);GO:0042803(protein homodimerization activity);GO:0031430(M band)
circRNA652	GO:0016491(oxidoreductase activity);GO:0055114(oxidation-reduction process);GO:0003677(DNA binding);GO:0005634(nucleus);GO:0006355("regulation of transcription, DNA-templated");GO:0034720(histone H3-K4 demethylation);GO:0050660(flavin adenine dinucleotide binding);GO:0005515(protein binding);GO:0006357(regulation of transcription from RNA polymerase II promoter);GO:0045944(positive regulation of transcription from RNA polymerase II promoter);GO:0003682(chromatin binding);GO:0045892("negative regulation of transcription, DNA-templated");GO:0032091(negative regulation of protein binding);GO:0043426(MRF binding);GO:0051091(positive regulation of sequence-specific DNA binding transcription factor activity);GO:0000790(nuclear chromatin);GO:0019899(enzyme binding);GO:0043433(negative regulation of sequence-specific DNA binding transcription factor activity);GO:0006482(protein demethylation);GO:0030374(ligand-dependent nuclear receptor transcription coactivator activity);GO:0008134(transcription factor binding);GO:0034648(histone demethylase activity (H3-dimethyl-K4 specific));GO:0033169(histone H3-K9 demethylation);GO:0032454(histone demethylase activity (H3-K9 specific));GO:0032453(histone demethylase activity (H3-K4 specific));GO:0032452(histone demethylase activity);GO:0032451(demethylase activity);GO:1902166(negative regulation of intrinsic apoptotic signaling pathway in response to DNA damage by p53 class mediator);GO:0043518("negative regulation of DNA damage response, signal transduction by p53 class mediator");GO:0050681(androgen receptor binding);GO:0002039(p53 binding);GO:0003700(sequence-specific DNA binding transcription factor activity);GO:0005667(transcription factor complex);GO:0044212(transcription regulatory region DNA binding);GO:0000122(negative regulation of transcription from RNA polymerase II promoter);GO:0021983(pituitary gland development);GO:0001701(in utero embryonic development);GO:0008283(cell proliferation);GO:0001085(RNA polymerase II transcription factor binding);GO:0051573(negative regulation of histone H3-K9 methylation);GO:2000179(positive regulation of neural precursor cell proliferation);GO:0046886(positive regulation of hormone biosynthetic process);GO:0045648(positive regulation of erythrocyte differentiation);GO:0030851(granulocyte differentiation);GO:2000648(positive regulation of stem cell proliferation);GO:0055001(muscle cell development);GO:0051572(negative regulation of histone H3-K4 methylation);GO:0045654(positive regulation of megakaryocyte differentiation);GO:0010725(regulation of primitive erythrocyte differentiation)
circRNA653	GO:0003723(RNA binding);GO:0005515(protein binding);GO:0005488(binding);GO:0044822(poly(A) RNA binding);GO:0007283(spermatogenesis);GO:0001934(positive regulation of protein phosphorylation);GO:0045727(positive regulation of translation);GO:0060903(positive regulation of meiosis I)
circRNA654	GO:0003723(RNA binding);GO:0005515(protein binding);GO:0005488(binding);GO:0044822(poly(A) RNA binding);GO:0007283(spermatogenesis);GO:0001934(positive regulation of protein phosphorylation);GO:0045727(positive regulation of translation);GO:0060903(positive regulation of meiosis I)
circRNA655	GO:0035253(ciliary rootlet);GO:0051297(centrosome organization);GO:0005813(centrosome);GO:0070062(extracellular vesicular exosome);GO:0005737(cytoplasm);GO:0005515(protein binding);GO:0005886(plasma membrane);GO:0015629(actin cytoskeleton);GO:0010457(centriole-centriole cohesion);GO:0033365(protein localization to organelle);GO:0008104(protein
circRNA656	GO:0005515(protein binding)
circRNA657	GO:0003676(nucleic acid binding)
circRNA658	GO:0016567(protein ubiquitination);GO:0035556(intracellular signal transduction);GO:0005515(protein binding);GO:0008017(microtubule binding);GO:0005813(centrosome);GO:0005930(axoneme);GO:0036064(ciliary basal body);GO:0042384(cilium assembly);GO:0005929(cilium);GO:0042733(embryonic digit morphogenesis);GO:0030992(intraciliary transport particle B);GO:0035050(embryonic heart tube development);GO:1901621(negative regulation of smoothened signaling pathway involved in dorsal/ventral neural tube patterning);GO:0031076(embryonic camera-type eye development);GO:0036342(post-anal tail morphogenesis);GO:0021532(neural tube patterning);GO:0050687(negative regulation of defense response to virus);GO:0032688(negative regulation of interferon-beta production)
circRNA659	();GO:0003677(DNA binding);GO:0005737(cytoplasm);GO:0005515(protein binding);GO:0005886(plasma membrane);GO:0042803(protein homodimerization activity)
circRNA66	GO:0005515(protein binding);GO:0030659(cytoplasmic vesicle membrane);GO:0046907(intracellular transport)
circRNA660	();GO:0003677(DNA binding);GO:0005737(cytoplasm);GO:0005515(protein binding);GO:0005886(plasma membrane);GO:0042803(protein homodimerization activity)
circRNA661	GO:0005525(GTP binding);GO:0005622(intracellular);GO:0007264(small GTPase mediated signal transduction);GO:0008060(ARF GTPase activator activity);GO:0008270(zinc ion binding);GO:0032312(regulation of ARF GTPase activity);GO:0043547(positive regulation of GTPase activity);GO:0046872(metal ion binding);GO:0005515(protein binding);GO:0015031(protein transport);GO:0006184(GTP catabolic process);GO:0007165(signal transduction);GO:0016020(membrane);GO:0005543(phospholipid binding)
circRNA662	GO:0005515(protein binding);GO:0016020(membrane);GO:0044822(poly(A) RNA binding);GO:0017148(negative regulation of translation);GO:0007631(feeding behavior);GO:0035264(multicellular organism growth);GO:0009791(post-embryonic development);GO:0008344(adult locomotory behavior);GO:0050885(neuromuscular process controlling balance);GO:0031571(mitotic G1 DNA damage checkpoint);GO:0021522(spinal cord motor neuron differentiation);GO:0048009(insulin-like growth factor receptor signaling pathway);GO:0048873(homeostasis of number of cells within a tissue);GO:0044267(cellular protein metabolic process);GO:0050881(musculoskeletal movement)
circRNA663	GO:0070062(extracellular vesicular exosome);GO:0005515(protein binding);GO:0031410(cytoplasmic vesicle)
circRNA664	GO:0008270(zinc ion binding);GO:0046872(metal ion binding);GO:0005515(protein binding);GO:0005634(nucleus);GO:0030018(Z disc);GO:0003779(actin binding);GO:0030036(actin cytoskeleton organization);GO:0001725(stress fiber)
circRNA665	GO:0005634(nucleus);GO:0006355("regulation of transcription, DNA-templated");GO:0005515(protein binding);GO:0043565(sequence-specific DNA binding);GO:0009952(anterior/posterior pattern specification);GO:0006338(chromatin remodeling);GO:0007283(spermatogenesis);GO:0010369(chromocenter)
circRNA666	GO:0005506(iron ion binding);GO:0016491(oxidoreductase activity);GO:0016705("oxidoreductase activity, acting on paired donors, with incorporation or reduction of molecular oxygen");GO:0016706("oxidoreductase activity, acting on paired donors, with incorporation or reduction of molecular oxygen, 2-oxoglutarate as one donor, and incorporation of one atom each of oxygen into both donors");GO:0031418(L-ascorbic acid binding);GO:0055114(oxidation-reduction process);GO:0060348(bone development);GO:0005783(endoplasmic reticulum);GO:0070062(extracellular vesicular exosome);GO:0016020(membrane);GO:0006457(protein folding);GO:0050821(protein stabilization);GO:0018126(protein hydroxylation);GO:0050708(regulation of protein secretion);GO:1901874(negative regulation of post-translational protein modification);GO:0005737(cytoplasm);GO:0005634(nucleus);GO:0016049(cell growth);GO:0030199(collagen fibril organization);GO:0030278(regulation of ossification)
circRNA667	GO:0003676(nucleic acid binding);GO:0004527(exonuclease activity);GO:0044822(poly(A) RNA binding);GO:0005515(protein binding)
circRNA668	GO:0003700(sequence-specific DNA binding transcription factor activity);GO:0005634(nucleus);GO:0006351("transcription, DNA-templated");GO:0045893("positive regulation of transcription, DNA-templa
circRNA669	GO:0005975(carbohydrate metabolic process);GO:0016773("phosphotransferase activity, alcohol group as acceptor");GO:0070050(neuron cellular homeostasis)
circRNA67	GO:0004843(ubiquitin-specific protease activity);GO:0005509(calcium ion binding);GO:0006508(proteolysis);GO:0006511(ubiquitin-dependent protein catabolic process);GO:0008233(peptidase activity);GO:0008234(cysteine-type peptidase activity);GO:0016787(hydrolase activity);GO:0036459(ubiquitinyl hydrolase activity)
circRNA670	GO:0005975(carbohydrate metabolic process);GO:0016773("phosphotransferase activity, alcohol group as acceptor");GO:0070050(neuron cellular homeostasis)
circRNA671	GO:0005887(integral component of plasma membrane);GO:0004930(G-protein coupled receptor activity);GO:0007186(G-protein coupled receptor signaling pathway);GO:0001540(beta-amyloid binding);GO:0097190(apoptotic signaling pathway)
circRNA672	GO:0005737(cytoplasm);GO:0006508(proteolysis);GO:0006810(transport);GO:0006914(autophagy);GO:0008233(peptidase activity);GO:0008234(cysteine-type peptidase activity);GO:0015031(protein transport);GO:0016787(hydrolase activity);GO:0005576(extracellular region);GO:0004197(cysteine-type endopeptidase activity);GO:0000045(autophagic vacuole assembly)
circRNA673	GO:0000287(magnesium ion binding);GO:0005975(carbohydrate metabolic process);GO:0016868("intramolecular transferase activity, phosphotransferases");GO:0046872(metal ion binding);GO:0070062(extracellular vesicular exosome);GO:0004614(phosphoglucomutase activity);GO:0006006(glucose metabolic process)
circRNA674	GO:0004672(protein kinase activity);GO:0004713(protein tyrosine kinase activity);GO:0004714(transmembrane receptor protein tyrosine kinase activity);GO:0005524(ATP binding);GO:0006468(protein phosphorylation);GO:0007169(transmembrane receptor protein tyrosine kinase signaling pathway);GO:0016020(membrane);GO:0016021(integral component of membrane);GO:0016772("transferase
circRNA675	GO:0000166(nucleotide binding);GO:0004672(protein kinase activity);GO:0004674(protein serine/threonine kinase activity);GO:0005524(ATP binding);GO:0006468(protein phosphorylation);GO:0007165(signal transduction);GO:0016301(kinase activity);GO:0016310(phosphorylation);GO:0016740(transferase activity);GO:0016772("transferase activity, transferring
circRNA676	GO:0006810(transport);GO:0016020(membrane);GO:0016021(integral component of membrane);GO:0005515(protein binding);GO:0005801(cis-Golgi network);GO:0090161(Golgi ribbon formation);GO:0070971(endoplasmic reticulum exit site);GO:0005793(endoplasmic reticulum-Golgi intermediate compartment)
circRNA677	GO:0016020(membrane);GO:0016021(integral component of membrane)
circRNA678	GO:0005215(transporter activity);GO:0005886(plasma membrane);GO:0006810(transport);GO:0016020(membrane);GO:0016021(integral component of m
circRNA679	GO:0006812(cation transport);GO:0008324(cation transmembrane transporter activity);GO:0016021(integral component of membrane);GO:0055085(transmembrane transport);GO:0070062(extracellular vesicular exosome);GO:0005737(cytoplasm);GO:0005794(Golgi apparatus);GO:0031982(vesicle);GO:0048471(perinuclear region of cytoplasm);GO:0016023(cytoplasmic membrane-bounded vesicle);GO:0006829(zinc ion transport);GO:0032119(sequestering of zinc ion)
circRNA68	GO:0000166(nucleotide binding);GO:0003824(catalytic activity);GO:0003989(acetyl-CoA carboxylase activity);GO:0004075(biotin carboxylase activity);GO:0005524(ATP binding);GO:0006633(fatty acid biosynthetic process);GO:0008152(metabolic process);GO:0016874(ligase activity);GO:0046872(metal ion binding);GO:0008716(D-alanine-D-alanine ligase activity);GO:0070062(extracellular vesicular exosome);GO:0005515(protein binding);GO:0005739(mitochondrion);GO:0005829(cytosol);GO:0006629(lipid metabolic process);GO:0001894(tissue homeostasis);GO:0051289(protein homotetramerization);GO:0055088(lipid homeostasis);GO:0006084(acetyl-CoA metabolic process);GO:0044268(multicellular organismal protein metabolic process)
circRNA680	GO:0005634(nucleus);GO:0005515(protein binding)
circRNA681	GO:0003876(AMP deaminase activity);GO:0006188(IMP biosynthetic process);GO:0009168(purine ribonucleoside monophosphate biosynthetic process);GO:0019239(deaminase activity);GO:0032264(IMP s

circRNA682	();GO:0005765(lysosomal membrane);GO:0032526(response to retinoic acid)
circRNA683	GO:0003677(DNA binding);GO:0003700(sequence-specific DNA binding transcription factor activity);GO:0005634(nucleus);GO:0006351("transcription, DNA-templated");GO:0006355("regulation of transcription, DNA-templated");GO:0042803(protein homodimerization activity);GO:0000122(negative regulation of transcription from RNA polymerase II promoter);GO:0048701(embryonic cranial skeleton morphogenesis);GO:0046982(protein heterodimerization activity);GO:0000976(transcription regulatory region sequence-specific DNA binding);GO:0048704(embryonic skeletal system morphogenesis);GO:0001106(RNA polymerase II transcription corepressor activity);GO:0070722(Tle3-Aes complex)
circRNA684	GO:0005737(cytoplasm);GO:0005515(protein binding);GO:0005634(nucleus);()
circRNA685	GO:0005737(cytoplasm);GO:0005515(protein binding);GO:0005634(nucleus);()
circRNA686	GO:0016591("DNA-directed RNA polymerase II, holoenzyme")
circRNA687	GO:0005634(nucleus);GO:0006355("regulation of transcription, DNA-templated");GO:0005515(protein binding);GO:0043234(protein complex);GO:0035019(somatic stem cell maintenance)
circRNA688	GO:0004430(1-phosphatidylinositol 4-kinase activity);GO:0006661(phosphatidylinositol biosynthetic process);GO:0016301(kinase activity);GO:0016310(phosphorylation);GO:0016740(transferase activity);GO:0016772("transferase activity, transferring phosphorus-containing groups");GO:0016773("phosphotransferase activity, alcohol group as acceptor");GO:0046854(phosphatidylinositol phosphorylation);GO:0048015(phosphatidylinositol-mediated signaling);GO:0005515(protein binding)
circRNA689	GO:0003676(nucleic acid binding);GO:0003677(DNA binding);GO:0046872(metal ion binding);GO:0005737(cytoplasm);GO:0005515(protein binding);GO:0005634(nucleus);GO:0051382(kinetochore assembly);GO:0000790(nuclear chromatin);GO:0007064(mitotic sister chromatid cohesion)
circRNA69	GO:0008270(zinc ion binding);GO:0046872(metal ion binding);GO:0005515(protein binding);GO:0016020(membrane);GO:0006511(ubiquitin-dependent protein catabolic process);GO:0005737(cytoplasm);GO:0005886(plasma membrane);GO:0004842(ubiquitin-protein transferase activity);GO:0031625(ubiquitin protein ligase binding);GO:0010008(endosome membrane);GO:0070936(protein K48-linked ubiquitination);GO:0002020(protease binding);GO:0019901(protein kinase binding);GO:0043161(proteasome-mediated ubiquitin-dependent protein catabolic process);GO:0002947(tumor necrosis factor receptor superfamily complex);GO:1901797(negative regulation of signal transduction by p53 class mediator);GO:2001271(negative regulation of cysteine-type endopeptidase activity involved in execution phase of apoptosis);GO:0010804(negative regulation of tumor necrosis factor-mediated signaling pathway);GO:1902042(negative regulation of extrinsic apoptotic signaling pathway via death domain receptors);GO:0002039(p53 binding);GO:0016023(cytoplasmic membrane-bounded vesicle);GO:0006886(intracellular protein transport);GO:0010762(regulation of fibroblast migration)
circRNA690	GO:0006886(intracellular protein transport);GO:0007165(signal transduction);GO:0016197(endosomal transport);GO:0032266(phosphatidylinositol-3-phosphate binding);GO:0035091(phosphatidylinositol binding);GO:0005515(protein binding);GO:0005737(cytoplasm);GO:0005634(nucleus);GO:0005769(early endosome);GO:0071203(WASH complex);GO:0030904(retromer
circRNA691	GO:0003700(sequence-specific DNA binding transcription factor activity);GO:0006355("regulation of transcription, DNA-templated");GO:0043565(sequence-specific DNA binding);GO:0005515(protein binding);GO:0005634(nucleus);GO:0045944(positive regulation of transcription from RNA polymerase II promoter)
circRNA692	GO:0005515(protein binding);GO:0045944(positive regulation of transcription from RNA polymerase II promoter);GO:0005634(nucleus);GO:0030374(ligand-dependent nuclear receptor transcription coactivator activity);GO:0080008(Cul4-RING E3 ubiquitin ligase complex)
circRNA693	GO:0005515(protein binding);GO:0045944(positive regulation of transcription from RNA polymerase II promoter);GO:0005634(nucleus);GO:0030374(ligand-dependent nuclear receptor transcription coactivator activity);GO:0080008(Cul4-RING E3 ubiquitin ligase complex)
circRNA694	GO:0005515(protein binding);GO:0045944(positive regulation of transcription from RNA polymerase II promoter);GO:0005634(nucleus);GO:0030374(ligand-dependent nuclear receptor transcription coactivator activity);GO:0080008(Cul4-RING E3 ubiquitin ligase complex)
circRNA695	GO:0005515(protein binding);GO:0045944(positive regulation of transcription from RNA polymerase II promoter);GO:0005634(nucleus);GO:0030374(ligand-dependent nuclear receptor transcription coactivator activity);GO:0080008(Cul4-RING E3 ubiquitin ligase complex)
circRNA696	GO:0003676(nucleic acid binding);GO:0044822(poly(A) RNA binding);GO:0005634(nucleus);GO:0006397(mRNA processing);GO:0043066(negative regulation of apoptotic process);GO:0003723(RNA binding);GO:0000910(cytokinesis);GO:0043484(regulation of RNA splicing);GO:0016607(nuclear speck);GO:0000226(microtubule cytoskeleton organization);GO:0051726(regulation of cell
circRNA697	GO:0005783(endoplasmic reticulum);GO:0005886(plasma membrane)
circRNA698	GO:0003676(nucleic acid binding);GO:0006479(protein methylation);GO:0008168(methyltransferase activity);GO:0008276(protein methyltransferase activity);GO:0032259(methylation);GO:0005515(protein binding);GO:0030307(positive regulation of cell growth);GO:0043234(protein complex)
circRNA699	GO:0004867(serine-type endopeptidase inhibitor activity);GO:0007399(nervous system development);GO:0008201(heparin binding);GO:0010951(negative regulation of endopeptidase activity);GO:0016021(integral component of membrane);GO:0046914(transition metal ion binding);GO:0016020(membrane);GO:0043231(intracellular membrane-bounded
circRNA7	GO:0003824(catalytic activity);GO:0004553("hydrolase activity, hydrolyzing O-glycosyl compounds");GO:0005516(calmodulin binding);GO:0005976(polysaccharide metabolic process);GO:0005977(glycoge
circRNA70	GO:0005515(protein binding);GO:0070062(extracellular vesicular exosome);GO:0016020(membrane);GO:0006511(ubiquitin-dependent protein catabolic process);GO:0005634(nucleus);GO:0048863(stem cell differentiation);GO:0043248(proteasome assembly);GO:0022624(proteasome accessory complex);GO:0005838(proteasome regulatory particle)
circRNA700	GO:0006508(proteolysis);GO:0006511(ubiquitin-dependent protein catabolic process);GO:0008233(peptidase activity);GO:0008234(cysteine-type peptidase activity);GO:0016787(hydrolase activity);GO:0036459(ubiquitinyl hydrolase activity);GO:0005515(protein binding);GO:0070536(protein K63-linked deubiquitination);GO:0032183(SUMO binding);GO:0004843(ubiquitin-specific protease
circRNA701	GO:0000166(nucleotide binding);GO:0005525(GTP binding);GO:0005622(intracellular);GO:0007264(small GTPase mediated signal transduction);GO:0006886(intracellular protein transport);GO:0005515(protein binding);GO:0007224(smoothened signaling pathway);GO:0060170(ciliary membrane);GO:0009953(dorsal/ventral pattern formation);GO:0042384(cilium
circRNA702	GO:0005097(Rab GTPase activator activity);GO:0032313(regulation of Rab GTPase activity);GO:0032851(positive regulation of Rab GTPase activity);GO:0032755(positive regulation of interleukin-6 production);GO:0050727(regulation of inflammatory response);GO:0032680(regulation of tumor necrosis factor production)
circRNA703	GO:0005737(cytoplasm);GO:0005634(nucleus);GO:0060348(bone development)
circRNA704	GO:0008270(zinc ion binding);GO:0046872(metal ion binding);GO:0005515(protein binding);GO:0005737(cytoplasm);GO:0044822(poly(A) RNA binding);GO:0004842(ubiquitin-protein transferase activity);GO:0000209(protein polyubiquitination);GO:0003723(RNA binding);GO:0019902(phosphatase binding);GO:0031593(polyubiquitin binding)
circRNA705	GO:0008270(zinc ion binding);GO:0046872(metal ion binding);GO:0005515(protein binding);GO:0005737(cytoplasm);GO:0044822(poly(A) RNA binding);GO:0004842(ubiquitin-protein transferase activity);GO:0000209(protein polyubiquitination);GO:0003723(RNA binding);GO:0019902(phosphatase binding);GO:0031593(polyubiquitin binding)
circRNA706	GO:0005515(protein binding)
circRNA707	()
circRNA708	()
circRNA709	GO:0005515(protein binding);GO:0005737(cytoplasm)
circRNA71	GO:0005515(protein binding);GO:0005634(nucleus);GO:0035064(methylated histone binding);GO:0045596(negative regulation of cell differentiation);GO:0042054(histone methyltransferase activity);GO:0035098(ESC/E(Z) complex);GO:0043565(sequence-specific DNA binding);GO:0003682(chromatin binding);GO:0003723(RNA binding);GO:0000122(negative regulation of transcription from RNA polymerase II promoter);GO:0008284(positive regulation of cell proliferation);GO:0016574(histone ubiquitination);GO:0016571(histone methylation);GO:0001739(sex chromatin)
circRNA710	GO:0000166(nucleotide binding);GO:0004672(protein kinase activity);GO:0004674(protein serine/threonine kinase activity);GO:0005524(ATP binding);GO:0006468(protein phosphorylation);GO:0016301(kinase activity);GO:0016310(phosphorylation);GO:0016740(transferase activity);GO:0016772("transferase activity, transferring phosphorus-containing groups");GO:0004713(protein tyrosine kinase activity);GO:0005737(cytoplasm);GO:0005515(protein binding);GO:0005634(nucleus);GO:0061098(positive regulation of protein tyrosine kinase activity);GO:0030296(protein tyrosine kinase activator activity);GO:0043066(negative regulation of apoptotic process);GO:0050731(positive regulation of peptidyl-tyrosine phosphorylation);GO:0042802(identical protein binding);GO:0046777(protein autophosphorylation);GO:0019901(protein kinase binding);GO:2001271(negative regulation of cysteine-type endopeptidase activity involved in execution phase of apoptosis);GO:2001238(positive regulation of extrinsic apoptotic signaling pathway);GO:0018105(peptidyl-serine phosphorylation)
circRNA711	GO:0005515(protein binding);GO:0030054(cell junction);GO:0005783(endoplasmic reticulum);GO:0048471(perinuclear region of cytoplasm);GO:0005923(tight junction);GO:0070062(extracellular vesicular exosome);GO:0005737(cytoplasm);GO:0005634(nucleus);GO:0005874(microtubule);GO:0016323(basolateral plasma membrane);GO:0005794(Golgi apparatus);GO:0019902(phosphatase binding);GO:0007015(actin filament organization);GO:0005911(cell-cell junction);GO:0042391(regulation of membrane potential);GO:0009898(cytoplasmic side of plasma membrane);GO:0030866(cortical actin cytoskeleton organization);GO:0016337(single organismal cell-cell adhesion);GO:0019901(protein kinase binding);GO:0008022(protein C-terminus binding);GO:0097025(MPP7-DLG1-LIN7 complex);GO:0031434(mitogen-activated protein kinase kinase binding);GO:0043268(positive regulation of potassium ion transport);GO:0001935(endothelial cell proliferation);GO:0045930(negative regulation of mitotic cell cycle);GO:0072659(protein localization to plasma membrane);GO:0070830(tight junction assembly);GO:0090004(positive regulation of establishment of protein localization to plasma membrane);GO:0044325(ion channel binding);GO:0097016(L27 domain binding);GO:0005886(plasma membrane);GO:0045202(synapse);GO:0001658(branching involved in ureteric bud morphogenesis);GO:0002088(lens development in camera-type eye);GO:0050680(negative regulation of epithelial cell proliferation);GO:0008284(positive regulation of cell proliferation);GO:0045121(membrane raft);GO:0014069(postsynaptic density);GO:0031594(neuromuscular junction);GO:0001657(ureteric bud development);GO:0048704(embryonic skeletal system morphogenesis);GO:0032147(activation of protein kinase activity);GO:0033268(node of Ranvier);GO:0048745(smooth muscle tissue development);GO:0005605(basal lamina);GO:0030432(peristalsis);GO:0001772(immunological synapse);GO:0051898(negative regulation of protein kinase B signaling);GO:0008104(protein localization);GO:0016328(lateral plasma membrane);GO:0030838(positive regulation of actin filament polymerization);GO:0031579(membrane raft organization);GO:0031253(cell projection membrane);GO:0042110(T cell activation);GO:0032947(protein complex scaffold);GO:0031641(regulation of myelination);GO:0042130(negative regulation of T cell proliferation);GO:0048608(reproductive structure development);GO:0001771(immunological synapse formation);GO:0002369(T cell cytokine production);GO:0042982(amyloid precursor protein metabolic process);GO:0048729(tissue morphogenesis);GO:0060022(hard palate development);GO:0035748(myelin sheath abaxonal region);GO:0043219(lateral loop)

circRNA712	GO:0005515(protein binding);GO:0030054(cell junction);GO:0005783(endoplasmic reticulum);GO:0048471(perinuclear region of cytoplasm);GO:0005923(tight junction);GO:0070062(extracellular vesicular exosome);GO:0005737(cytoplasm);GO:0005634(nucleus);GO:0005874(microtubule);GO:0016323(basolateral plasma membrane);GO:0005794(Golgi apparatus);GO:0019902(phosphatase binding);GO:0007015(actin filament organization);GO:0005911(cell-cell junction);GO:0042391(regulation of membrane potential);GO:0009898(cytoplasmic side of plasma membrane);GO:0030866(cortical actin cytoskeleton organization);GO:0016337(single organismal cell-cell adhesion);GO:0019901(protein kinase binding);GO:0008022(protein C-terminus binding);GO:0097025(MPP7-DLG1-LIN7 complex);GO:0031434(mitogen-activated protein kinase kinase binding);GO:0043268(positive regulation of potassium ion transport);GO:0001935(endothelial cell proliferation);GO:0045930(negative regulation of mitotic cell cycle);GO:0072659(protein localization to plasma membrane);GO:0070830(tight junction assembly);GO:0090004(positive regulation of establishment of protein localization to plasma membrane);GO:0044325(ion channel binding);GO:0097016(L27 domain binding);GO:0005886(plasma membrane);GO:0045202(synapse);GO:0001658(branching involved in ureteric bud morphogenesis);GO:0002088(lens development in camera-type eye);GO:0050680(negative regulation of epithelial cell proliferation);GO:0008284(positive regulation of cell proliferation);GO:0045121(membrane raft);GO:0014069(postsynaptic density);GO:0031594(neuromuscular junction);GO:0001657(ureteric bud development);GO:0048704(embryonic skeletal system morphogenesis);GO:0032147(activation of protein kinase activity);GO:0033268(node of Ranvier);GO:0048745(smooth muscle tissue development);GO:0005605(basal lamina);GO:0030432(peristalsis);GO:0001772(immunological synapse);GO:0051898(negative regulation of protein kinase B signaling);GO:0008104(protein localization);GO:0016328(lateral plasma membrane);GO:0030838(positive regulation of actin filament polymerization);GO:0031579(membrane raft organization);GO:0031253(cell projection membrane);GO:0042110(T cell activation);GO:0032947(protein complex scaffold);GO:0031641(regulation of myelination);GO:0042130(negative regulation of T cell proliferation);GO:0048608(reproductive structure development);GO:0001771(immunological synapse formation);GO:0002369(T cell cytokine production);GO:0042982(amyloid precursor protein metabolic process);GO:0048729(tissue morphogenesis);GO:0060022(hard palate development);GO:0035748(myelin sheath abaxonal region);GO:0043219(lateral loop)
circRNA713	GO:0005737(cytoplasm);GO:0005777(peroxisome);GO:0007031(peroxisome organization);GO:0005641(nuclear envelope lumen);GO:0004620(phospholipase activity);GO:0046485(ether lipid metabolic process)
circRNA714	GO:0004252(serine-type endopeptidase activity);GO:0006508(proteolysis);GO:0016021(integral component of membrane);GO:0005739(mitochondrion);GO:0005743(mitochondrial inner membrane);GO:0090201(negative regulation of release of cytochrome c from mitochondria);GO:2001243(negative regulation of intrinsic apoptotic signaling pathway)
circRNA715	GO:0005515(protein binding);GO:0000151(ubiquitin ligase complex)
circRNA716	GO:0006508(proteolysis);GO:0006511(ubiquitin-dependent protein catabolic process);GO:0008233(peptidase activity);GO:0008234(cysteine-type peptidase activity);GO:0008242(omega peptidase activity);GO:0008270(zinc ion binding);GO:0016787(hydrolase activity);GO:0036459(ubiquitinyl hydrolase activity);GO:0046872(metal ion binding);GO:0005515(protein binding);GO:0006355("regulation of transcription, DNA-templated");GO:0004197(cysteine-type endopeptidase activity);GO:0031625(ubiquitin protein ligase binding);GO:0008283(cell proliferation);GO:0070536(protein K63-linked deubiquitination);GO:0004221(ubiquitin thiolesterase activity);GO:0050821(protein stabilization);GO:0010506(regulation of autophagy);GO:0043130(ubiquitin binding);GO:0004843(ubiquitin-specific protease activity)
circRNA717	GO:0006508(proteolysis);GO:0006511(ubiquitin-dependent protein catabolic process);GO:0008233(peptidase activity);GO:0008234(cysteine-type peptidase activity);GO:0008242(omega peptidase activity);GO:0008270(zinc ion binding);GO:0016787(hydrolase activity);GO:0036459(ubiquitinyl hydrolase activity);GO:0046872(metal ion binding);GO:0005515(protein binding);GO:0006355("regulation of transcription, DNA-templated");GO:0004197(cysteine-type endopeptidase activity);GO:0031625(ubiquitin protein ligase binding);GO:0008283(cell proliferation);GO:0070536(protein K63-linked deubiquitination);GO:0004221(ubiquitin thiolesterase activity);GO:0050821(protein stabilization);GO:0010506(regulation of autophagy);GO:0043130(ubiquitin binding);GO:0004843(ubiquitin-specific protease activity)
circRNA718	GO:0005515(protein binding);GO:0045893("positive regulation of transcription, DNA-templated");GO:0045944(positive regulation of transcription from RNA polymerase II promoter);GO:0003714(transcription corepressor activity);GO:0000122(negative regulation of transcription from RNA polymerase II promoter);GO:0005634(nucleus);GO:0060070(canonical Wnt signaling pathway);GO:0047485(protein N-terminus binding);GO:0005876(spindle microtubule);GO:0000118(histone deacetylase complex);GO:0042393(histone binding);GO:0008013(beta-catenin binding);GO:0017053(transcriptional repressor complex);GO:0003677(DNA binding);GO:0044212(transcription regulatory region DNA binding);GO:0045892("negative regulation of transcription, DNA-templated");GO:0043161(proteasome-mediated ubiquitin-dependent protein catabolic process)
circRNA719	GO:0005515(protein binding);GO:0044822(poly(A) RNA binding);GO:0005783(endoplasmic reticulum);GO:0016477(cell migration);GO:0045600(positive regulation of fat cell differentiation);GO:0045668(negative regulation of osteoblast differentiation);GO:0048146(positive regulation of fibroblast proliferation);GO:0034446(substrate adhesion-dependent cell spreading);GO:0060510(Type II pneumocyte differentiation)
circRNA72	GO:0005622(intracellular);GO:0006396(RNA processing);GO:0005730(nucleolus)
circRNA720	GO:0005515(protein binding);GO:0044822(poly(A) RNA binding);GO:0005783(endoplasmic reticulum);GO:0016477(cell migration);GO:0045600(positive regulation of fat cell differentiation);GO:0045668(negative regulation of osteoblast differentiation);GO:0048146(positive regulation of fibroblast proliferation);GO:0034446(substrate adhesion-dependent cell spreading);GO:0060510(Type II pneumocyte differentiation)
circRNA721	GO:0003824(catalytic activity);GO:0008152(metabolic process);GO:0035091(phosphatidylinositol binding);GO:0016020(membrane);GO:0005515(protein binding);GO:0005794(Golgi apparatus);GO:0005768(endosome);GO:0005765(lysosomal membrane);GO:0050830(defense response to Gram-positive bacterium)
circRNA722	GO:0004672(protein kinase activity);GO:0004674(protein serine/threonine kinase activity);GO:0005083(small GTPase regulator activity);GO:0005524(ATP binding);GO:0006468(protein phosphorylation);GO:0016772("transferase activity, transferring phosphorus-containing groups");GO:0050790(regulation of catalytic activity);GO:0004713(protein tyrosine kinase activity);GO:0000166(nucleotide binding);GO:0005856(cytoskeleton);GO:0070062(extracellular vesicular exosome);GO:0005737(cytoplasm);GO:0005515(protein binding);GO:0005634(nucleus);GO:0035556(intracellular signal transduction);GO:0007010(cytoskeleton organization);GO:0046777(protein autophosphorylation);GO:0031532(actin cytoskeleton reorganization);GO:0007256(activation of JNKK activity);GO:0048814(regulation of dendrite morphogenesis)
circRNA723	GO:0003922(GMP synthase (glutamine-hydrolyzing) activity);GO:0005524(ATP binding);GO:0006164(purine nucleotide biosynthetic process);GO:0006177(GMP biosynthetic process);GO:0016462(pyrophosphatase activity);GO:0004066(asparagine synthase (glutamine-hydrolyzing) activity);GO:0006529(asparagine biosynthetic process);GO:0016740(transferase activity);GO:0008033(tRNA processing);GO:0005737(cytoplasm);GO:0016787(hydrolase activity);GO:0006541(glutamine metabolic process)
circRNA724	GO:0019887(protein kinase regulator activity);GO:0045859(regulation of protein kinase activity);GO:0005515(protein binding);GO:0045944(positive regulation of transcription from RNA polymerase II promoter);GO:0005102(receptor binding);GO:0006469(negative regulation of protein kinase activity);GO:0004860(protein kinase inhibitor activity);GO:0042110(T cell activation);GO:0030838(positive regulation of actin filament polymerization);GO:0030971(receptor tyrosine kinase binding);GO:0060548(negative regulation of cell death);GO:0042102(positive regulation of T cell proliferation);GO:0005911(cell-cell junction);GO:0019904(protein domain specific binding);GO:0007015(actin filament organization);GO:0016477(cell migration);GO:0012506(vesicle membrane);GO:0030032(lamellipodium assembly);GO:0051707(response to other organism)
circRNA725	GO:0005509(calcium ion binding);GO:0005515(protein binding);GO:0090263(positive regulation of canonical Wnt signaling pathway);GO:0000159(protein phosphatase type 2A complex);GO:0090090(negative regulation of canonical Wnt signaling pathway);GO:0090249(regulation of cell motility involved in somitogenic axis elongation);GO:0001754(eye photoreceptor cell differentiation);GO:0007525(somatic muscle development);GO:0045732(positive regulation of protein catabolic process)
circRNA726	GO:0005509(calcium ion binding);GO:0005515(protein binding);GO:0090263(positive regulation of canonical Wnt signaling pathway);GO:0000159(protein phosphatase type 2A complex);GO:0090090(negative regulation of canonical Wnt signaling pathway);GO:0090249(regulation of cell motility involved in somitogenic axis elongation);GO:0001754(eye photoreceptor cell differentiation);GO:0007525(somatic muscle development);GO:0045732(positive regulation of protein catabolic process)
circRNA727	GO:0007517(muscle organ development);GO:0007528(neuromuscular junction development)
circRNA728	GO:0004672(protein kinase activity);GO:0004674(protein serine/threonine kinase activity);GO:0005524(ATP binding);GO:0006468(protein phosphorylation);GO:0016772("transferase activity, transferring phosphorus-containing groups");GO:0004713(protein tyrosine kinase activity);GO:0005730(nucleolus);GO:0005515(protein binding);GO:0005634(nucleus);GO:0035556(intracellular signal transduction);GO:0031573(intra-S DNA damage checkpoint);GO:0031572(G2 DNA damage checkpoint)
circRNA729	GO:0009055(electron carrier activity);GO:0015035(protein disulfide oxidoreductase activity);GO:0045454(cell redox homeostasis);GO:0005829(cytosol)
circRNA73	GO:0005730(nucleolus);GO:0005829(cytosol);GO:0005737(cytoplasm);GO:0005515(protein binding);GO:0005634(nucleus);GO:0035145(exon-exon junction complex);GO:0042162(telomeric DNA binding);GO:0090502("RNA phosphodiester bond hydrolysis, endonucleolytic");GO:0000723(telomere maintenance);GO:0004521(endoribonuclease activity);()
circRNA730	GO:0008152(metabolic process);GO:0016874(ligase activity);GO:0016881(acid-amino acid ligase activity);GO:0005829(cytosol);GO:0005515(protein binding);GO:0004842(ubiquitin-protein transferase activity);GO:0070936(protein K48-linked ubiquitination);GO:0030433(ER-associated ubiquitin-dependent protein catabolic process);GO:0018279(protein N-linked glycosylation via asparagine);GO:0005783(endoplasmic reticulum)
circRNA731	GO:0005829(cytosol);GO:0005634(nucleus);GO:0005737(cytoplasm)
circRNA732	GO:0005813(centrosome);GO:0016020(membrane);GO:0005737(cytoplasm);GO:0005515(protein binding);GO:0042384(cilium assembly);GO:0005814(centriole);GO:0000226(microtubule cytoskeleton organization);GO:0034451(centriolar satellite);GO:0090316(positive regulation of intracellular protein transport);GO:0001764(neuron migration);GO:0035264(multicellular organism growth);GO:0001701(in utero embryonic development);GO:0043066(negative regulation of apoptotic process);GO:0048854(brain morphogenesis);GO:0005815(microtubule organizing center);GO:0036064(ciliary basal body);GO:0035108(limb morphogenesis);GO:0021772(olfactory bulb development);GO:0031514(motile cilium);GO:0007051(spindle organization);GO:0061351(neural precursor cell proliferation);GO:0021696(cerebellar cortex morphogenesis);GO:0045171(intercellular bridge);GO:0000242(pericentriolar material)
circRNA733	GO:0006479(protein methylation);GO:0008168(methyltransferase activity);GO:0016740(transferase activity);GO:0032259(methylation);GO:0005515(protein binding);GO:0008276(protein methyltransferase activity);GO:0005737(cytoplasm);GO:0045893("positive regulation of transcription, DNA-templated");GO:0005829(cytosol);GO:0005634(nucleus);GO:0032088(negative regulation of
circRNA734	GO:0070062(extracellular vesicular exosome);GO:0050890(cognition)
circRNA735	GO:0008270(zinc ion binding);GO:0046872(metal ion binding);GO:0005515(protein binding);GO:0005739(mitochondrion);GO:0006511(ubiquitin-dependent protein catabolic process);GO:0005634(nucleus);GO:0004842(ubiquitin-protein transferase activity);GO:0070936(protein K48-linked ubiquitination);GO:0045665(negative regulation of neuron differentiation);GO:0005773(vacuole);GO:0010771(negative regulation of cell morphogenesis involved in differentiation)

circRNA736	GO:0000166(nucleotide binding);GO:0004672(protein kinase activity);GO:0004674(protein serine/threonine kinase activity);GO:0004712(protein serine/threonine/tyrosine kinase activity);GO:0005524(ATP binding);GO:0006468(protein phosphorylation);GO:0016772("transferase activity, transferring phosphorus-containing groups");GO:0046777(protein autophosphorylation);GO:0004713(protein tyrosine kinase activity);GO:0005515(protein binding);GO:0004715(non-membrane spanning protein tyrosine kinase activity);GO:0030529(ribonucleoprotein complex);GO:0042802(identical protein binding);GO:0018105(peptidyl-serine phosphorylation);GO:0043621(protein self-association);GO:0007623(circadian rhythm);GO:0043518("negative regulation of DNA damage response, signal transduction by p53 class mediator");GO:0018107(peptidyl-threonine phosphorylation);GO:0048156(tau protein binding);GO:0090312(positive regulation of protein deacetylation)
circRNA737	GO:0000166(nucleotide binding);GO:0004672(protein kinase activity);GO:0004674(protein serine/threonine kinase activity);GO:0004712(protein serine/threonine/tyrosine kinase activity);GO:0005524(ATP binding);GO:0006468(protein phosphorylation);GO:0016772("transferase activity, transferring phosphorus-containing groups");GO:0046777(protein autophosphorylation);GO:0004713(protein tyrosine kinase activity);GO:0005515(protein binding);GO:0004715(non-membrane spanning protein tyrosine kinase activity);GO:0030529(ribonucleoprotein complex);GO:0042802(identical protein binding);GO:0018105(peptidyl-serine phosphorylation);GO:0043621(protein self-association);GO:0007623(circadian rhythm);GO:0043518("negative regulation of DNA damage response, signal transduction by p53 class mediator");GO:0018107(peptidyl-threonine phosphorylation);GO:0048156(tau protein binding);GO:0090312(positive regulation of protein deacetylation)
circRNA738	GO:0004402(histone acetyltransferase activity);GO:0005634(nucleus);GO:0006355("regulation of transcription, DNA-templated");GO:0008080(N-acetyltransferase activity);GO:0008152(metabolic process);GO:0016573(histone acetylation);GO:0005515(protein binding);GO:0045944(positive regulation of transcription from RNA polymerase II promoter);GO:0008285(negative regulation of cell proliferation);GO:0003713(transcription coactivator activity);GO:0032403(protein complex binding);GO:0016407(acetyltransferase activity);GO:0032869(cellular response to insulin stimulus);GO:0003712(transcription cofactor activity);GO:0008134(transcription factor binding);GO:0010835(regulation of protein ADP-ribosylation);GO:0018076(N-terminal peptidyl-lysine acetylation);GO:0018394(peptidyl-lysine acetylation);GO:0018393(internal peptidyl-lysine acetylation);GO:0005671(Ada2/Gcn5/Ada3 transcription activator complex);GO:0043966(histone H3 acetylation);GO:0042826(histone deacetylase binding);GO:0019901(protein kinase binding);GO:0045893("positive regulation of transcription, DNA-templated");GO:0000123(histone acetyltransferase complex);GO:0031672(A band);GO:0004861(cyclin-dependent protein serine/threonine kinase inhibitor activity);GO:0045736(negative regulation of cyclin-dependent protein serine/threonine kinase activity);GO:0000776(kinetochore);GO:0045722(positive regulation of gluconeogenesis);GO:0031674(I band);GO:0004468(lysine N-acetyltransferase activity);GO:0042641(actomyosin);GO:0043970(histone H3-K9 acetylation)
circRNA739	GO:0016020(membrane);GO:0005886(plasma membrane);GO:0005764(lysosome);GO:0046627(negative regulation of insulin receptor signaling pathway);GO:0051898(negative regulation of protein kinase B signaling);GO:0005158(insulin receptor binding);GO:0046325(negative regulation of glucose import);GO:0045334(clathrin-coated endocytic vesicle);GO:0038016(insulin receptor internalization)
circRNA74	GO:0008270(zinc ion binding);GO:0046872(metal ion binding);GO:0005515(protein binding);GO:0035456(response to interferon-beta)
circRNA740	GO:0005515(protein binding)
circRNA741	GO:0005515(protein binding);GO:0032851(positive regulation of Rab GTPase activity);GO:0017112(Rab guanyl-nucleotide exchange factor activity);GO:0044257(cellular protein catabolic process);GO:0032313(regulation of Rab GTPase activity);GO:0008333(endosome to lysosome transport)
circRNA742	GO:0000166(nucleotide binding);GO:0004672(protein kinase activity);GO:0004713(protein tyrosine kinase activity);GO:0004871(signal transducer activity);GO:0005524(ATP binding);GO:0005856(cytoskeleton);GO:0005925(focal adhesion);GO:0006468(protein phosphorylation);GO:0007165(signal transduction);GO:0007172(signal complex assembly);GO:0016301(kinase activity);GO:0016310(phosphorylation);GO:0016740(transferase activity);GO:0016772("transferase activity, transferring phosphorus-containing groups");GO:0018108(peptidyl-tyrosine phosphorylation);GO:0042169(SH2 domain binding);GO:0005829(cytosol);GO:0005515(protein binding);GO:0005634(nucleus);GO:0003779(actin binding);GO:0043066(negative regulation of apoptotic process);GO:0007229(integrin-mediated signaling pathway);GO:0060396(growth hormone receptor signaling pathway);GO:0022408(negative regulation of cell-cell adhesion);GO:0051897(positive regulation of protein kinase B signaling);GO:0014068(positive regulation of phosphatidylinositol 3-kinase signaling);GO:0001934(positive regulation of protein phosphorylation);GO:0004715(non-membrane spanning protein tyrosine kinase activity);GO:0046777(protein autophosphorylation);GO:0045860(positive regulation of protein kinase activity);GO:0042127(regulation of cell proliferation);GO:0019901(protein kinase binding);GO:0030335(positive regulation of cell migration);GO:0008360(regulation of cell shape);GO:0048013(ephrin receptor signaling pathway);GO:0008432(JUN kinase binding);GO:2000811(negative regulation of anoikis);GO:0045667(regulation of osteoblast differentiation);GO:0033628(regulation of cell adhesion mediated by integrin);GO:0001570(vasculogenesis);GO:0001568(blood vessel development);GO:0001764(neuron migration);GO:0008284(positive regulation of cell proliferation);GO:0030198(extracellular matrix organization);GO:0046621(negative regulation of organ growth);GO:0000226(microtubule cytoskeleton organization);GO:0001525(angiogenesis);GO:0016324(apical plasma membrane);GO:0030027(lamellipodium);GO:0050771(negative regulation of axonogenesis);GO:0040023(establishment of nucleus localization);GO:0043542(endothelial cell migration);GO:2000060(positive regulation of protein ubiquitination involved in ubiquitin-dependent protein catabolic process);GO:0021955(central nervous system neuron axonogenesis);GO:0051964(negative regulation of synapse assembly)
circRNA743	GO:0000724(double-strand break repair via homologous recombination);GO:0006974(cellular response to DNA damage stimulus);GO:0005515(protein binding);GO:0031334(positive regulation of protein complex assembly);GO:0071479(cellular response to ionizing radiation);GO:0000228(nuclear chromosome);GO:0070202(regulation of establishment of protein localization to chromosome);GO:2000781(positive regulation of double-strand break repair);GO:0072757(cellular response to camptothecin);GO:0072711(cellular response to hydroxyurea);GO:0010569(regulation of double-strand break repair via homologous recombination)
circRNA744	GO:0000166(nucleotide binding);GO:0003677(DNA binding);GO:0004677(DNA-dependent protein kinase activity);GO:0005524(ATP binding);GO:0005634(nucleus);GO:0006303(double-strand break repair via nonhomologous end joining);GO:0006468(protein phosphorylation);GO:0016301(kinase activity);GO:0016310(phosphorylation);GO:0016740(transferase activity);GO:0016772("transferase activity, transferring phosphorus-containing groups");GO:0016773("phosphotransferase activity, alcohol group as acceptor");GO:0005515(protein binding);GO:0005488(binding);GO:0004674(protein serine/threonine kinase activity);GO:0016020(membrane);GO:0045944(positive regulation of transcription from RNA polymerase II promoter);GO:0005667(transcription factor complex);GO:0044822(poly(A) RNA binding);GO:0032869(cellular response to insulin stimulus);GO:0008134(transcription factor binding);GO:0072431(signal transduction involved in mitotic G1 DNA damage checkpoint);GO:0005958(DNA-dependent protein kinase-DNA ligase 4 complex);GO:0070419(nonhomologous end joining complex);GO:0018105(peptidyl-serine phosphorylation);GO:0007420(brain development);GO:0033077(T cell differentiation in thymus);GO:0043065(positive regulation of apoptotic process);GO:0006302(double-strand break repair);GO:0007507(heart development);GO:0001756(somitogenesis);GO:0008630(intrinsic apoptotic signaling pathway in response to DNA damage);GO:0031648(protein destabilization);GO:0019899(enzyme binding);GO:0000723(telomere maintenance);GO:0001933(negative regulation of protein phosphorylation);GO:0010332(response to gamma radiation);GO:0035234(ectopic germ cell programmed cell death);GO:0042752(regulation of circadian rhythm);GO:0030098(lymphocyte differentiation);GO:0033152(immunoglobulin V(D)J recombination);GO:0033153(T cell receptor V(D)J recombination);GO:0002328(pro-B cell differentiation);GO:0002326(B cell lineage commitment);GO:0002360(T cell lineage commitment)
circRNA745	GO:0001510(RNA methylation);GO:0008168(methyltransferase activity);GO:0009452(7-methylguanosine RNA capping);GO:0016740(transferase activity);GO:0005615(extracellular space);GO:0005737(cytoplasm);GO:0005515(protein binding);GO:0005634(nucleus);GO:0071164(RNA trimethylguanosine synthase activity);GO:0036261(7-methylguanosine cap
circRNA746	GO:0000922(spindle pole);GO:0005813(centrosome);GO:0005874(microtubule);GO:0032467(positive regulation of cytokinesis);GO:0051781(positive regulation of cell division);GO:0005819(spindle);GO:00
circRNA747	GO:0005085(guanyl-nucleotide exchange factor activity);GO:0005089(Rho guanyl-nucleotide exchange factor activity);GO:0032321(positive regulation of Rho GTPase activity);GO:0035023(regulation of Rho protein signal transduction);GO:0035556(intracellular signal transduction);GO:0043547(positive regulation of GTPase activity);GO:0005515(protein binding);GO:0007186(G-protein coupled receptor signaling pathway);GO:0032855(positive regulation of Rac GTPase activity);GO:0030676(Rac guanyl-nucleotide exchange factor activity);GO:0030675(Rac GTPase activator activity);GO:0008344(adult locomotory behavior);GO:0048813(dendrite morphogenesis)
circRNA748	GO:0005215(transporter activity);GO:0006810(transport);GO:0016020(membrane);GO:0005515(protein binding);GO:0055085(transmembrane transport);GO:0016021(integral component of membrane)
circRNA749	GO:0005215(transporter activity);GO:0006810(transport);GO:0016020(membrane);GO:0005515(protein binding);GO:0055085(transmembrane transport);GO:0016021(integral component of membrane)
circRNA75	GO:0005096(GTPase activator activity);GO:0008083(growth factor activity);GO:0043547(positive regulation of GTPase activity);GO:0043231(intracellular membrane-bounded organelle);GO:0005515(protein binding);GO:0042803(protein homodimerization activity);GO:0030139(endocytic vesicle);GO:0005768(endosome)
circRNA750	GO:0004725(protein tyrosine phosphatase activity);GO:0007275(multicellular organismal development);GO:0009887(organ morphogenesis);GO:0035335(peptidyl-tyrosine dephosphorylation);GO:0042471(ear morphogenesis);GO:0005737(cytoplasm);GO:0005515(protein binding);GO:0005634(nucleus);GO:0016576(histone dephosphorylation);GO:0045739(positive regulation of DNA repair);GO:0010212(response to ionizing radiation);GO:0006302(double-strand break repair);GO:0045944(positive regulation of transcription from RNA polymerase II promoter);GO:0003723(RNA binding);GO:0042472(inner ear morphogenesis);GO:0045165(cell fate commitment);GO:0001658(branching involved in ureteric bud morphogenesis);GO:0001656(metanephros development);GO:0045747(positive regulation of Notch signaling pathway);GO:0034613(cellular protein localization);GO:0006470(protein dephosphorylation);GO:0048665(neuron fate specification);GO:0035909(aorta morphogenesis);GO:0000132(establishment of mitotic spindle orientation);GO:0060487(lung epithelial cell differentiation);GO:2001240(negative regulation of extrinsic apoptotic signaling pathway in absence of ligand);GO:0001657(ureteric bud development);GO:0007389(pattern specification process);GO:0048704(embryonic skeletal system morphogenesis);GO:0050679(positive regulation of epithelial cell proliferation);GO:0048752(semicircular canal morphogenesis);GO:0042474(middle ear morphogenesis);GO:0003151(outflow tract morphogenesis);GO:0048856(anatomical structure development);GO:0090103(cochlea morphogenesis);GO:0045664(regulation of neuron differentiation);GO:0060037(pharyngeal system development);GO:0042473(outer ear morphogenesis);GO:0071600(otic vesicle morphogenesis);GO:0072513(positive regulation of secondary heart field cardioblast proliferation);GO:0071599(otic vesicle development);GO:0014706(striated muscle tissue development);GO:0035088(establishment or maintenance of apical/basal cell polarity)
circRNA751	()
circRNA752	GO:0046872(metal ion binding)
circRNA753	GO:0001731(formation of translation preinitiation complex);GO:0003743(translation initiation factor activity);GO:0005634(nucleus);GO:0005737(cytoplasm);GO:0005852(eukaryotic translation initiation factor 3 complex);GO:0006412(translation);GO:0006413(translational initiation);GO:0006446(regulation of translational initiation);GO:0016282(eukaryotic 43S preinitiation complex);GO:0016605(PML body);GO:0033290(eukaryotic 48S preinitiation complex);GO:0005515(protein binding);GO:0070062(extracellular vesicular exosome);GO:0016020(membrane);GO:0044822(poly(A) RNA binding);GO:0047485(protein N-terminus binding);GO:0000184("nuclear-transcribed mRNA catabolic process, nonsense-mediated decay")
circRNA754	()
circRNA755	GO:0009058(biosynthetic process);GO:0016740(transferase activity);GO:0005737(cytoplasm);GO:0032981(mitochondrial respiratory chain complex I assembly);GO:0005743(mitochondrial inner membrane);GO:0005739(mitochondrion)
circRNA756	GO:0044822(poly(A) RNA binding)
circRNA757	GO:0005515(protein binding)

circRNA758	GO:0005515(protein binding)
circRNA759	GO:0004672(protein kinase activity);GO:0004674(protein serine/threonine kinase activity);GO:0005524(ATP binding);GO:0005622(intracellular);GO:0005737(cytoplasm);GO:0006468(protein phosphorylation);GO:0016772("transferase activity, transferring phosphorus-containing groups");GO:0042981(regulation of apoptotic process);GO:0043065(positive regulation of apoptotic process);GO:0004713(protein tyrosine kinase activity);GO:0005856(cytoskeleton);GO:0005515(protein binding);GO:0042803(protein homodimerization activity);GO:0031398(positive regulation of protein ubiquitination);GO:0031982(vesicle);GO:0034134(toll-like receptor 2 signaling pathway);GO:0050731(positive regulation of peptidyl-tyrosine phosphorylation);GO:0033138(positive regulation of peptidyl-serine phosphorylation);GO:0030274(LIM domain binding);GO:0051092(positive regulation of NF-kappaB transcription factor activity);GO:0043123(positive regulation of I-kappaB kinase/NF-kappaB signaling);GO:0043234(protein complex);GO:0071225(cellular response to muramyl dipeptide);GO:0070431(nucleotide-binding oligomerization domain containing 2 signaling pathway);GO:0050700(CARD domain binding);GO:0010800(positive regulation of peptidyl-threonine phosphorylation);GO:0045087(innate immune response);GO:0050830(defense response to Gram-positive bacterium);GO:0071222(cellular response to lipopolysaccharide);GO:0050852(T cell receptor signaling pathway);GO:0032760(positive regulation of tumor necrosis factor production);GO:0070555(response to interleukin-1);GO:0031663(lipopolysaccharide-mediated signaling pathway);GO:0032722(positive regulation of chemokine production);GO:0032755(positive regulation of interleukin-6 production);GO:0071223(cellular response to lipoteichoic acid);GO:0070374(positive regulation of ERK1 and ERK2 cascade);GO:0007249(I-kappaB kinase/NF-kappaB signaling);GO:0032874(positive regulation of stress-activated MAPK cascade);GO:0043330(response to exogenous dsRNA);GO:0046330(positive regulation of JNK cascade);GO:0042098(T cell proliferation);GO:0034142(toll-like receptor 4 signaling pathway);GO:0002250(adaptive immune response);GO:0032729(positive regulation of interferon-gamma production);GO:0046641(positive regulation of alpha-beta T cell proliferation);GO:0045627(positive regulation of T-helper 1 cell differentiation);GO:0071224(cellular response to peptidoglycan);GO:0002827(positive regulation of T-helper 1 type immune response);GO:0032743(positive regulation of interleukin-2 production);GO:0001961(positive regulation of cytokine-mediated signaling pathway);GO:0033091(positive regulation of immature T cell proliferation);GO:0070427(nucleotide-binding oligomerization domain containing 1 signaling pathway);GO:0070671(response to interleukin-12);GO:0070673(response to interleukin-18)
circRNA76	GO:0005813(centrosome);GO:0005737(cytoplasm);GO:0005515(protein binding);GO:0000910(cytokinesis);GO:0005814(centriole);GO:0019904(protein domain specific binding);GO:0007099(centriole replication);GO:0051299(centrosome separation)
circRNA760	GO:0006508(proteolysis);GO:0008234(cysteine-type peptidase activity);GO:0005737(cytoplasm);GO:0005515(protein binding);GO:0005634(nucleus);GO:0070646(protein modification by small protein removal);GO:0016929(SUMO-specific protease activity);GO:0016926(protein desumoylation);GO:0090234(regulation of kinetochore assembly);GO:0090169(regulation of spindle assembly)
circRNA761	GO:0000166(nucleotide binding);GO:0003774(motor activity);GO:0005524(ATP binding);GO:0008152(metabolic process);GO:0016459(myosin complex);GO:0070062(extracellular vesicular exosome);GO:0016020(membrane);GO:0045944(positive regulation of transcription from RNA polymerase II promoter);GO:0005515(protein binding);GO:0005634(nucleus);GO:0005654(nucleoplasm);GO:0001726(ruffle);GO:0005794(Golgi apparatus);GO:0031965(nuclear membrane);GO:0016023(cytoplasmic membrane-bounded vesicle);GO:0030330("DNA damage response, signal transduction by p53 class mediator");GO:0016591("DNA-directed RNA polymerase II, holoenzyme");GO:0031941(filamentous actin);GO:0051046(regulation of secretion);GO:0045334(clathrin-coated endocytic vesicle);GO:0005886(plasma membrane);GO:0007626(locomotory behavior);GO:0005737(cytoplasm);GO:0048839(inner ear development);GO:0042472(inner ear morphogenesis);GO:0042491(auditory receptor cell differentiation);GO:0007605(sensory perception of sound);GO:0043025(neuronal cell body);GO:0007268(synaptic transmission);GO:0006605(protein targeting);GO:0030424(axon);GO:0012506(vesicle membrane);GO:0016358(dendrite development);GO:0007416(synapse assembly);GO:0048167(regulation of synaptic plasticity);GO:0014047(glutamate secretion);GO:0006897(endocytosis);GO:0071257(cellular response to electrical stimulus)
circRNA762	GO:0005102(receptor binding);GO:0016021(integral component of membrane);GO:0016529(sarcoplasmic reticulum);GO:0016020(membrane);GO:0005737(cytoplasm);GO:0005515(protein binding);GO:0005886(plasma membrane);GO:0060047(heart contraction);GO:0014808(release of sequestered calcium ion into cytosol by sarcoplasmic reticulum)
circRNA763	GO:0005102(receptor binding);GO:0016021(integral component of membrane);GO:0016529(sarcoplasmic reticulum);GO:0016020(membrane);GO:0005737(cytoplasm);GO:0005515(protein binding);GO:0005886(plasma membrane);GO:0060047(heart contraction);GO:0014808(release of sequestered calcium ion into cytosol by sarcoplasmic reticulum)
circRNA764	GO:0007155(cell adhesion)
circRNA765	GO:0032184(SUMO polymer binding);GO:0007626(locomotory behavior);GO:0042472(inner ear morphogenesis);GO:0007605(sensory perception of sound)
circRNA766	GO:0004842(ubiquitin-protein transferase activity);GO:0008152(metabolic process);GO:0016567(protein ubiquitination);GO:0016874(ligase activity);GO:0005515(protein binding);GO:0000139(Golgi membrane);GO:0042787(protein ubiquitination involved in ubiquitin-dependent protein catabolic process);GO:0048365(Rac GTPase binding);GO:0070936(protein K48-linked ubiquitination);GO:0061025(membrane fusion);GO:0017137(Rab GTPase binding);GO:0007030(Golgi organization);GO:0030334(regulation of cell migration)
circRNA767	GO:0004842(ubiquitin-protein transferase activity);GO:0008152(metabolic process);GO:0016567(protein ubiquitination);GO:0016874(ligase activity);GO:0005515(protein binding);GO:0000139(Golgi membrane);GO:0042787(protein ubiquitination involved in ubiquitin-dependent protein catabolic process);GO:0048365(Rac GTPase binding);GO:0070936(protein K48-linked ubiquitination);GO:0061025(membrane fusion);GO:0017137(Rab GTPase binding);GO:0007030(Golgi organization);GO:0030334(regulation of cell migration)
circRNA768	GO:0004842(ubiquitin-protein transferase activity);GO:0008152(metabolic process);GO:0016567(protein ubiquitination);GO:0016874(ligase activity);GO:0005515(protein binding);GO:0000139(Golgi membrane);GO:0042787(protein ubiquitination involved in ubiquitin-dependent protein catabolic process);GO:0048365(Rac GTPase binding);GO:0070936(protein K48-linked ubiquitination);GO:0061025(membrane fusion);GO:0017137(Rab GTPase binding);GO:0007030(Golgi organization);GO:0030334(regulation of cell migration)
circRNA769	GO:0005737(cytoplasm);GO:0044822(poly(A) RNA binding);GO:0005634(nucleus)
circRNA77	GO:0003774(motor activity);GO:0005524(ATP binding);GO:0008152(metabolic process);GO:0016459(myosin complex);GO:0051015(actin filament binding);GO:0000166(nucleotide binding);GO:0003779(actin binding);GO:0005515(protein binding);GO:0043234(protein complex);GO:0030016(myofibril);GO:0005794(Golgi apparatus);GO:0005911(cell-cell junction);GO:0031672(A band);GO:0014823(response to activity);GO:0001778(plasma membrane repair);GO:0070252(actin-mediated cell contraction);GO:0005826(actomyosin contractile ring)
circRNA770	GO:0003677(DNA binding);GO:0003700(sequence-specific DNA binding transcription factor activity);GO:0005634(nucleus);GO:0006355("regulation of transcription, DNA-templated");GO:0043565(sequence-specific DNA binding);GO:0005515(protein binding);GO:0000122(negative regulation of transcription from RNA polymerase II promoter);GO:0000980(RNA polymerase
circRNA771	GO:0000166(nucleotide binding);GO:0005524(ATP binding);GO:0005634(nucleus);GO:0006200(ATP catabolic process);GO:0006461(protein complex assembly);GO:0008152(metabolic process);GO:0016887(ATPase activity);GO:0017111(nucleoside-triphosphatase activity);GO:0045111(intermediate filament
circRNA772	GO:0000166(nucleotide binding);GO:0005524(ATP binding);GO:0005634(nucleus);GO:0006200(ATP catabolic process);GO:0006461(protein complex assembly);GO:0008152(metabolic process);GO:0016887(ATPase activity);GO:0017111(nucleoside-triphosphatase activity);GO:0045111(intermediate filament
circRNA773	GO:0003676(nucleic acid binding);GO:0046872(metal ion binding)
circRNA774	GO:0005102(receptor binding);GO:0005604(basement membrane);GO:0005606(laminin-1 complex);GO:0007155(cell adhesion);GO:0030155(regulation of cell adhesion);GO:0030334(regulation of cell migration);GO:0045995(regulation of embryonic development);GO:0005515(protein binding);GO:0016192(vesicle-mediated transport);GO:0016020(membrane);GO:0070062(extracellular vesicular
circRNA775	GO:0003779(actin binding);GO:0005198(structural molecule activity);GO:0005737(cytoplasm);GO:0005856(cytoskeleton);GO:0008092(cytoskeletal protein binding);GO:0019898(extrinsic component of membrane);GO:0030866(cortical actin cytoskeleton organization);GO:0070062(extracellular vesicular exosome);GO:0008180(COP9 signalosome);GO:0005515(protein binding);GO:0042731(PH domain
circRNA776	GO:0005215(transporter activity);GO:0006810(transport);GO:0016020(membrane);GO:0016021(integral component of membrane);GO:0022857(transmembrane transporter activity);GO:0022891(substrate-specific transmembrane transporter activity);GO:0055085(transmembrane transport);GO:0015758(glucose transport)
circRNA777	GO:0004560(alpha-L-fucosidase activity);GO:0005975(carbohydrate metabolic process);GO:0006004(fucose metabolic process);GO:0005615(extracellular space);GO:0070062(extracellular vesicular exosome);GO:2000535(regulation of entry of bacterium into host cell);GO:0009617(response to bacterium);GO:0016139(glycoside catabolic process)
circRNA778	GO:0003779(actin binding);GO:0005509(calcium ion binding);GO:0008270(zinc ion binding);GO:0005515(protein binding);GO:0070062(extracellular vesicular exosome);GO:0005737(cytoplasm);GO:0005886(plasma membrane);GO:0005634(nucleus);GO:0001954(positive regulation of cell-matrix adhesion);GO:0031527(filopodium
circRNA779	GO:0003779(actin binding);GO:0005509(calcium ion binding);GO:0008270(zinc ion binding);GO:0005515(protein binding);GO:0070062(extracellular vesicular exosome);GO:0005737(cytoplasm);GO:0005886(plasma membrane);GO:0005634(nucleus);GO:0001954(positive regulation of cell-matrix adhesion);GO:0031527(filopodium
circRNA78	GO:0005634(nucleus);GO:0006355("regulation of transcription, DNA-templated");GO:0008270(zinc ion binding);GO:0048706(embryonic skeletal system development)
circRNA780	GO:0003779(actin binding);GO:0005509(calcium ion binding);GO:0008270(zinc ion binding);GO:0005515(protein binding);GO:0070062(extracellular vesicular exosome);GO:0005737(cytoplasm);GO:0005886(plasma membrane);GO:0005634(nucleus);GO:0001954(positive regulation of cell-matrix adhesion);GO:0031527(filopodium
circRNA781	GO:0003779(actin binding);GO:0016021(integral component of membrane);GO:0005515(protein binding);GO:0005737(cytoplasm);GO:0044822(poly(A) RNA binding);GO:0005634(nucleus);GO:0005794(Golgi apparatus);GO:0045211(postsynaptic membrane);GO:0031965(nuclear membrane);GO:0042692(muscle cell differentiation);GO:0005635(nuclear envelope);GO:0007030(Golgi organization);GO:0030017(sarcomere);GO:0090292(nuclear matrix anchoring at nuclear membrane);GO:0090286(cytoskeletal anchoring at nuclear membrane);GO:0034993(SUN-KASH complex);GO:0005521(lamin binding);GO:0051015(actin filament binding);GO:0042803(protein homodimerization activity);GO:0040023(establishment of nucleus localization)
circRNA782	GO:0003779(actin binding);GO:0016021(integral component of membrane);GO:0005515(protein binding);GO:0005737(cytoplasm);GO:0044822(poly(A) RNA binding);GO:0005634(nucleus);GO:0005794(Golgi apparatus);GO:0045211(postsynaptic membrane);GO:0031965(nuclear membrane);GO:0042692(muscle cell differentiation);GO:0005635(nuclear envelope);GO:0007030(Golgi organization);GO:0030017(sarcomere);GO:0090292(nuclear matrix anchoring at nuclear membrane);GO:0090286(cytoskeletal anchoring at nuclear membrane);GO:0034993(SUN-KASH complex);GO:0005521(lamin binding);GO:0051015(actin filament binding);GO:0042803(protein homodimerization activity);GO:0040023(establishment of nucleus localization)
circRNA783	GO:0003677(DNA binding);GO:0005622(intracellular);GO:0005634(nucleus);GO:0005737(cytoplasm);GO:0005515(protein binding);GO:0016514(SWI/SNF complex)
circRNA784	GO:0003677(DNA binding);GO:0005622(intracellular);GO:0005634(nucleus);GO:0005737(cytoplasm);GO:0005515(protein binding);GO:0016514(SWI/SNF complex)
circRNA785	GO:0003677(DNA binding);GO:0005622(intracellular);GO:0005634(nucleus);GO:0005737(cytoplasm);GO:0005515(protein binding);GO:0016514(SWI/SNF complex)
circRNA786	GO:0005215(transporter activity);GO:0005737(cytoplasm);GO:0006810(transport);GO:0016021(integral component of membrane);GO:0070062(extracellular vesicular exosome);GO:0016020(membrane);GO:0005515(protein binding);GO:0009986(cell surface);GO:0030139(endocytic vesicle);GO:0030140(trans-Golgi network transport vesicle);GO:0005768(endosome);GO:0042802(identical protein binding);GO:0001948(glycoprotein binding);GO:0051219(phosphoprotein binding);GO:0005634(nucleus);GO:0005520(insulin-like growth factor binding);GO:0005641(nuclear envelope lumen);GO:0005537(mannose binding)
circRNA787	GO:0003723(RNA binding);GO:0005515(protein binding);GO:0044822(poly(A) RNA binding);GO:0001570(vasculogenesis);GO:0042552(myelination);GO:0010628(positive regulation of gene expression);GO:0007286(spermatid development);GO:0042759(long-chain fatty acid biosynthetic process);GO:0042692(muscle cell differentiation);GO:0008366(axon ensheathment)
circRNA788	GO:0003723(RNA binding);GO:0005515(protein binding);GO:0044822(poly(A) RNA binding);GO:0001570(vasculogenesis);GO:0042552(myelination);GO:0010628(positive regulation of gene expression);GO:0007286(spermatid development);GO:0042759(long-chain fatty acid biosynthetic process);GO:0042692(muscle cell differentiation);GO:0008366(axon ensheathment)

circRNA79	GO:0000166(nucleotide binding);GO:0005525(GTP binding);GO:0007264(small GTPase mediated signal transduction);GO:0015031(protein transport);GO:0003924(GTPase activity);GO:0006184(GTP catabolic process);GO:0006886(intracellular protein transport);GO:0006913(nucleocytoplasmic transport);GO:0007165(signal transduction);GO:0005622(intracellular);GO:0005634(nucleus);GO:0005737(cytoplasm);GO:0016020(membrane);GO:0043231(intracellular membrane-bounded organelle);GO:0070062(extracellular vesicular exosome);GO:0019003(GDP binding);GO:0005811(lipid particle);GO:0005765(lysosomal membrane);GO:0005515(protein binding);GO:0007032(endosome organization);GO:0030100(regulation of endocytosis);GO:0030139(endocytic vesicle)
circRNA8	GO:0005507(copper ion binding);GO:0007155(cell adhesion);GO:0016491(oxidoreductase activity);GO:0030168(platelet activation);GO:0046872(metal ion binding);GO:0055114(oxidation-reduction process);GO:0005515(protein binding)
circRNA80	GO:0000220("vacuolar proton-transporting V-type ATPase, V0 domain");GO:0015078(hydrogen ion transmembrane transporter activity);GO:0015991(ATP hydrolysis coupled proton transport);GO:0033179("proton-transporting V-type ATPase, V0 domain");GO:0043231(intracellular membrane-bounded organelle);GO:0070062(extracellular vesicular exosome);GO:0005737(cytoplasm);GO:0005515(protein binding);GO:0005886(plasma membrane);GO:0005634(nucleus);GO:0005794(Golgi apparatus);GO:0051117(ATPase binding)
circRNA81	GO:0000166(nucleotide binding);GO:0003676(nucleic acid binding);GO:0003723(RNA binding);GO:0004386(helicase activity);GO:0005524(ATP binding);GO:0006200(ATP catabolic process);GO:0008026(ATP-dependent helicase activity);GO:0008152(metabolic process);GO:0016787(hydrolase activity);GO:0005515(protein binding);GO:0044822(poly(A) RNA binding);GO:0005634(nucleus);GO:0042802(identical protein binding);GO:0071013(catalytic step 2 spliceosome)
circRNA82	GO:0000123(histone acetyltransferase complex);GO:0005515(protein binding);GO:0005634(nucleus);GO:0071339(MLL1 complex);GO:0043984(histone H4-K16 acetylation);GO:0046972(histone acetyltransferase activity (H4-K16 specific));GO:0043996(histone acetyltransferase activity (H4-K8 specific));GO:0043995(histone acetyltransferase activity (H4-K5 specific));GO:0043982(histone H4-K8
circRNA83	GO:0004672(protein kinase activity);GO:0005524(ATP binding);GO:0006468(protein phosphorylation);GO:0016772("transferase activity, transferring phosphorus-containing groups");GO:0005515(protein binding);GO:0004713(protein tyrosine kinase activity);GO:0035556(intracellular signal transduction);GO:0046777(protein autophosphorylation);GO:0043123(positive regulation of I-kappaB kinase/NF-kappaB signaling)
circRNA84	GO:0005515(protein binding);GO:0000978(RNA polymerase II core promoter proximal region sequence-specific DNA binding);GO:0005634(nucleus);GO:0031492(nucleosomal DNA binding);GO:0016514(SWI/SNF complex);GO:0006337(nucleosome disassembly);GO:0000790(nuclear chromatin);GO:0006338(chromatin remodeling);GO:0043234(protein complex);GO:0043044(ATP-dependent chromatin remodeling);GO:0000980(RNA polymerase II distal enhancer sequence-specific DNA binding)
circRNA85	GO:0000922(spindle pole);GO:0005813(centrosome)
circRNA86	GO:0003756(protein disulfide isomerase activity);GO:0005783(endoplasmic reticulum);GO:0006457(protein folding);GO:0008152(metabolic process);GO:0016853(isomerase activity);GO:0045454(cell redox homeostasis);GO:0070062(extracellular vesicular exosome);GO:0005515(protein binding);GO:0044822(poly(A) RNA binding);GO:0004656(procollagen-proline 4-dioxygenase activity);GO:1902175(regulation of oxidative stress-induced intrinsic apoptotic signaling pathway);GO:0018401(peptidyl-proline hydroxylation to 4-hydroxy-L-proline);GO:0016222(procollagen-proline 4-dioxygenase complex);GO:0071456(cellular response to hypoxia);GO:0005793(endoplasmic reticulum-Golgi intermediate compartment);GO:0034976(response to endoplasmic reticulum stress)
circRNA87	GO:0007165(signal transduction);GO:0008093(cytoskeletal adaptor activity);GO:0017124(SH3 domain binding);GO:0032956(regulation of actin cytoskeleton organization);GO:0046847(filopodium assembly);GO:0005515(protein binding);GO:0070062(extracellular vesicular exosome);GO:0005829(cytosol);GO:0015629(actin cytoskeleton);GO:0070064(proline-rich region
circRNA88	GO:0000287(magnesium ion binding);GO:0004749(ribose phosphate diphosphokinase activity);GO:0009165(nucleotide biosynthetic process)
circRNA89	GO:0003723(RNA binding);GO:0005047(signal recognition particle binding);GO:0005737(cytoplasm);GO:0005786("signal recognition particle, endoplasmic reticulum targeting");GO:0006614(SRP-dependent cotranslational protein targeting to membrane);GO:0008312(7S RNA binding);GO:0030529(ribonucleoprotein complex);GO:0030942(endoplasmic reticulum signal peptide
circRNA9	GO:0005515(protein binding)
circRNA90	GO:0005515(protein binding);GO:0003723(RNA binding);GO:0006397(mRNA processing);GO:0005634(nucleus);GO:0005737(cytoplasm);GO:0044822(poly(A) RNA binding);GO:0003682(chromatin binding);GO:0035064(methylated histone binding);GO:0003713(transcription coactivator activity);GO:0035145(exon-exon junction complex);GO:2001141(regulation of RNA biosynthetic process)
circRNA91	GO:0005515(protein binding);GO:0005737(cytoplasm);GO:0005634(nucleus)
circRNA92	GO:0003677(DNA binding);GO:0003700(sequence-specific DNA binding transcription factor activity);GO:0005634(nucleus);GO:0006355("regulation of transcription, DNA-templated");GO:0043565(sequence-specific DNA binding);GO:0045944(positive regulation of transcription from RNA polymerase II promoter);GO:0005515(protein binding);GO:0000978(RNA polymerase II core promoter proximal region sequence-specific DNA binding);GO:0001077(RNA polymerase II core promoter proximal region sequence-specific DNA binding transcription factor activity involved in positive regulation of transcription);GO:0050860(negative regulation of T cell receptor signaling pathway);GO:0001817(regulation of cytokine production)
circRNA93	GO:0005524(ATP binding);GO:0016887(ATPase activity);GO:0005739(mitochondrion)
circRNA94	GO:0003824(catalytic activity);GO:0005524(ATP binding);GO:0005634(nucleus);GO:0005674(transcription factor TFIIF complex);GO:0006200(ATP catabolic process);GO:0006366(transcription from RNA polymerase II promoter);GO:0006367(transcription initiation from RNA polymerase II promoter);GO:0008026(ATP-dependent helicase activity);GO:0005515(protein
circRNA95	GO:0006810(transport);GO:0006839(mitochondrial transport);GO:0016020(membrane);GO:0016021(integral component of membrane);GO:0031966(mitochondrial membrane)
circRNA96	GO:0005801(cis-Golgi network);GO:0006886(intracellular protein transport);GO:0016020(membrane);GO:0005737(cytoplasm);GO:0005515(protein binding);GO:0005886(plasma membrane);GO:0005634(nucleus);GO:0008565(protein transporter activity);GO:0005794(Golgi apparatus);GO:0006486(protein glycosylation);GO:0050821(protein stabilization);GO:0017119(Golgi
circRNA97	GO:0003824(catalytic activity);GO:0005524(ATP binding);GO:0008152(metabolic process);GO:0016874(ligase activity);GO:0046872(metal ion binding);GO:0005739(mitochondrion);GO:0070062(extracellular vesicular exosome);GO:0005515(protein binding)
circRNA98	GO:0005515(protein binding);GO:0070062(extracellular vesicular exosome)
circRNA99	GO:0003700(sequence-specific DNA binding transcription factor activity);GO:0005622(intracellular);GO:0005634(nucleus);GO:0005667(transcription factor complex);GO:0006351("transcription, DNA-templated");GO:0006355("regulation of transcription, DNA-templated");GO:0007179(transforming growth factor beta receptor signaling pathway);GO:0005515(protein binding);GO:0006468(protein phosphorylation);GO:0005737(cytoplasm);GO:0030902(hindbrain development);GO:0051216(cartilage development);GO:0060348(bone development);GO:0071407(cellular response to organic cyclic compound);GO:0030509(BMP signaling pathway);GO:0001657(ureteric bud development);GO:0030901(midbrain development)

Additional file 2

circRNA	miRNA	target sites cour	muscle count	muscle_fpkm
circRNA431	oar-miR-323a-5p	13	512	2590
circRNA431	oar-miR-3957-5p	12	512	2590
circRNA432	oar-miR-323a-5p	11	268	1640
circRNA431	oar-miR-1197-5p	11	512	2590
circRNA433	oar-miR-323a-5p	10	213	1440
circRNA432	oar-miR-3957-5p	10	268	1640
circRNA432	oar-miR-1197-5p	9	268	1640
circRNA434	oar-miR-323a-5p	9	167	1280
circRNA433	oar-miR-3957-5p	9	213	1440
circRNA433	oar-miR-1197-5p	8	213	1440
circRNA434	oar-miR-3957-5p	7	167	1280
circRNA434	oar-miR-1197-5p	7	167	1280
circRNA431	oar-miR-411b-5p	7	512	2590
circRNA434	oar-miR-411b-5p	6	167	1280
circRNA431	oar-miR-412-3p	6	512	2590
circRNA433	oar-miR-411b-5p	6	213	1440
circRNA432	oar-miR-411b-5p	6	268	1640
circRNA431	oar-miR-496-5p	5	512	2590
circRNA434	oar-miR-412-3p	5	167	1280
circRNA432	oar-miR-412-3p	5	268	1640
circRNA432	oar-miR-496-5p	5	268	1640
circRNA433	oar-miR-412-3p	5	213	1440
circRNA312	oar-miR-433-3p	5	190	3850
circRNA430	oar-let-7i	5	319	2550
circRNA434	oar-miR-496-5p	4	167	1280
circRNA275	oar-miR-23a	4	165	2850
circRNA433	oar-miR-496-5p	4	213	1440
circRNA544	oar-miR-136	4	58.5	2700
circRNA430	oar-miR-3957-5p	4	319	2550

circRNA543	oar-miR-136	4	8.5	744
circRNA620	oar-miR-329b-5p	3	75	2640
circRNA783	oar-miR-370-3p	3	339	16100
circRNA742	oar-miR-134-3p	3	63	2210
circRNA181	oar-miR-26a	3	28	916
circRNA35	oar-miR-323a-5p	3	34	1030
circRNA87	oar-miR-495-5p	3	22	1330
circRNA584	oar-miR-412-5p	3	318	12200
circRNA784	oar-miR-377-5p	3	1260	41800
circRNA776	oar-miR-26b	3	27	331
circRNA275	oar-miR-23b	3	165	2850
circRNA776	oar-miR-26a	3	27	331
circRNA431	oar-miR-543-5p	3	512	2590
circRNA784	oar-miR-370-3p	3	1260	41800
circRNA251	bta-miR-499	2	53	1050
circRNA742	bta-miR-133a	2	63	2210
circRNA742	bta-miR-133	2	63	2210
circRNA775	bta-miR-208	2	17	410
circRNA322	bta-miR-208	2	130	4390
circRNA36	oar-miR-26b	2	100	2650
circRNA375	oar-let-7a	2	16	1010
circRNA380	oar-miR-654-5p	2	16	944
circRNA784	oar-miR-485-5p	2	1260	41800
circRNA205	oar-miR-654-5p	2	34	1150
circRNA550	oar-miR-412-3p	2	59	1040
circRNA154	oar-miR-136	2	27	1160
circRNA212	oar-miR-377-5p	2	118	4620
circRNA227	oar-miR-541-3p	2	122	4390
circRNA566	oar-miR-412-5p	2	65	3570
circRNA744	oar-miR-494-3p	2	56	1790
circRNA603	oar-miR-3958-5p	2	13	873
circRNA291	oar-miR-329b-5p	2	23	830
circRNA332	oar-miR-154b-5p	2	101	3410
circRNA302	oar-miR-30a-3p	2	158	3480
ciRNA17	oar-miR-1197-5p	2	5	545
circRNA2	oar-miR-133	2	128	3720
circRNA436	oar-miR-218a	2	123	2820
circRNA429	oar-let-7i	2	121	3760
circRNA609	oar-miR-323a-5p	2	49	1860
circRNA164	oar-miR-16b	2	107	5430
circRNA723	oar-miR-154b-5p	2	21	1160
circRNA775	oar-miR-495-5p	2	17	410
circRNA170	oar-miR-25	2	74	1320
circRNA168	oar-miR-376a-3p	2	45	1730
circRNA716	oar-let-7i	2	83.5	2960
circRNA294	oar-miR-323a-5p	2	158	8020
circRNA455	oar-miR-541-5p	2	20	994
circRNA727	oar-miR-1193-3p	2	82	4350
circRNA87	oar-miR-412-3p	2	22	1330
circRNA679	oar-miR-487a-5p	2	45	2240
circRNA58	oar-miR-495-5p	2	24	1080
circRNA432	oar-miR-654-5p	2	268	1640
circRNA733	oar-miR-485-5p	2	22	1110
circRNA181	oar-miR-26b	2	28	916
circRNA424	oar-miR-26a	2	48	2000
circRNA168	oar-miR-376d	2	45	1730
circRNA308	oar-miR-432	2	2	146
circRNA435	oar-miR-323a-5p	2	20.2	602
circRNA669	oar-miR-3955-5p	2	178	6140
ciRNA20	oar-let-7i	2	11	319
circRNA582	oar-miR-370-3p	2	39	1480
circRNA443	oar-miR-362	2	143	7000
circRNA640	oar-miR-654-5p	2	59	2230
circRNA105	oar-miR-323a-5p	2	22	896
circRNA89	oar-miR-154b-5p	2	24	2180
circRNA368	oar-miR-377-3p	2	128	5040
circRNA588	oar-let-7f	2	20	1250
circRNA557	oar-miR-544-5p	2	203	9800
circRNA686	oar-miR-329b-5p	2	30	923
circRNA289	oar-miR-654-5p	2	19	965
circRNA87	oar-miR-379-5p	2	22	1330
circRNA435	oar-miR-3957-5p	2	20.2	602
circRNA740	oar-miR-544-3p	2	11	815
circRNA605	oar-miR-150	2	63	526
circRNA507	oar-miR-758-5p	2	166	4910
circRNA716	oar-let-7g	2	83.5	2960
circRNA338	oar-miR-136	2	45.5	2180
circRNA588	oar-let-7a	2	20	1250
circRNA22	oar-miR-329b-5p	2	255	5330
circRNA498	oar-miR-26a	2	15	657
circRNA19	oar-miR-136	2	22	1010
circRNA456	oar-miR-154b-5p	2	12	700
circRNA375	oar-let-7i	2	16	1010
circRNA785	oar-miR-134-5p	2	2	135
circRNA767	oar-miR-181a	2	45.2	3460
circRNA434	oar-miR-543-5p	2	167	1280
circRNA783	oar-miR-377-5p	2	339	16100
circRNA587	oar-miR-370-3p	2	24	1480
circRNA652	oar-miR-329b-5p	2	13	706
circRNA721	oar-miR-16b	2	174	9120
circRNA401	oar-miR-654-3p	2	13	666
circRNA434	oar-miR-654-5p	2	167	1280
circRNA274	oar-miR-433-3p	2	66	2070
circRNA375	oar-let-7b	2	16	1010
circRNA773	oar-miR-154a-5p	2	25	1110
circRNA749	oar-miR-323a-5p	2	72	3380
circRNA44	oar-miR-16b	2	61	1930
circRNA283	oar-miR-370-3p	2	219	14800

circRNA688	oar-miR-411b-5p	2	32	2450
circRNA391	oar-miR-495-5p	2	19	358
circRNA733	oar-miR-544-3p	2	22	1110
circRNA447	oar-miR-181a	2	48	1760
circRNA589	oar-let-7f	2	11	260
circRNA679	oar-miR-29b	2	45	2240
circRNA602	oar-miR-376a-5p	2	197	11100
circRNA31	oar-miR-495-5p	2	102	1620
circRNA337	oar-miR-26b	2	529	30300
circRNA557	oar-miR-382-5p	2	203	9800
circRNA637	oar-miR-495-5p	2	120	2400
circRNA439	oar-miR-485-5p	2	146	3240
circRNA114	oar-miR-30a-5p	2	350	14700
circRNA8	oar-miR-493-3p	2	85	3450
circRNA776	oar-miR-655-5p	2	27	331
circRNA114	oar-miR-30d	2	350	14700
circRNA375	oar-let-7d	2	16	1010
ciRNA41	oar-miR-1197-5p	2	7	551
circRNA776	oar-miR-329a-5p	2	27	331
circRNA375	oar-let-7c	2	16	1010
circRNA651	oar-miR-1197-5p	2	78	1620
circRNA419	oar-let-7c	2	12	777
circRNA589	oar-miR-487b-5p	2	11	260
circRNA346	oar-miR-485-5p	2	15	570
circRNA766	oar-miR-154b-5p	2	86.2	2720
circRNA387	oar-miR-485-5p	2	74	3810
circRNA315	oar-miR-668-3p	2	32	1880
circRNA540	oar-miR-143	2	87	3030
circRNA268	oar-miR-329b-5p	2	6	339
circRNA397	oar-miR-758-3p	2	54	2570
circRNA191	oar-miR-370-3p	2	89	4480
circRNA195	oar-miR-29a	2	53	1510
circRNA168	oar-let-7c	2	45	1730
circRNA171	oar-miR-493-3p	2	166	8060
ciRNA37	oar-miR-370-3p	2	22	666
circRNA87	oar-miR-370-5p	2	22	1330
circRNA737	oar-miR-26a	2	37	1410
circRNA523	oar-miR-3955-3p	2	69.5	3180
ciRNA27	oar-miR-134-3p	2	25	2040
circRNA297	oar-miR-485-5p	2	12	698
circRNA481	oar-miR-382-5p	2	44	2590
circRNA691	oar-miR-154b-3p	2	8	474
circRNA733	oar-miR-134-3p	2	22	1110
circRNA31	oar-miR-377-5p	2	102	1620
circRNA681	oar-miR-154b-5p	2	49	1570
circRNA435	oar-miR-1197-5p	2	20.2	602
circRNA788	oar-miR-493-5p	2	400	16300
circRNA115	oar-miR-3957-5p	2	280	9430
circRNA419	oar-let-7b	2	12	777
circRNA419	oar-let-7a	2	12	777
circRNA646	oar-miR-433-5p	2	19	876
circRNA498	oar-miR-26b	2	15	657
circRNA612	oar-miR-410-5p	2	9	348
circRNA168	oar-miR-654-5p	2	45	1730
circRNA75	oar-miR-3957-5p	2	45	1530
circRNA287	oar-miR-412-3p	2	6	215
circRNA272	oar-miR-1197-5p	2	67	969
circRNA195	oar-miR-29b	2	53	1510
circRNA565	oar-miR-329b-5p	2	45	1450
circRNA17	oar-miR-654-3p	2	62	2020
circRNA532	oar-miR-200a	2	731	18000
circRNA50	oar-miR-654-5p	2	41	968
circRNA768	oar-miR-154b-5p	2	43.7	1850
circRNA14	oar-let-7i	2	99	3350
circRNA121	oar-miR-3955-5p	2	633	14600
circRNA629	oar-miR-329b-5p	2	57	1900
circRNA29	oar-miR-194	2	53	1680
circRNA367	oar-miR-27a	2	93.5	3400
circRNA744	oar-miR-493-3p	2	56	1790
circRNA40	oar-miR-3957-5p	2	22	1130
circRNA442	oar-miR-143	2	32	1980
circRNA415	oar-miR-370-3p	2	49	1620
circRNA277	oar-miR-432	2	971	48700
circRNA19	oar-miR-382-3p	2	22	1010
circRNA481	oar-let-7b	2	44	2590
circRNA343	oar-miR-654-5p	2	31	1690
circRNA140	oar-miR-134-3p	2	10	719
circRNA455	oar-let-7i	2	20	994
ciRNA62	oar-miR-3955-3p	2	6	277
circRNA641	oar-miR-654-5p	2	14	853
circRNA621	oar-miR-329b-5p	2	46	3840
circRNA743	oar-miR-1197-3p	2	291	8460
circRNA677	oar-miR-654-5p	2	238	8090
circRNA287	oar-miR-377-3p	2	6	215
circRNA52	oar-miR-1197-5p	2	14	313
circRNA190	oar-miR-370-3p	2	10	840
circRNA438	oar-miR-25	2	33	1310
circRNA162	oar-miR-382-5p	2	8	320
circRNA369	oar-miR-655-5p	2	33	1560
circRNA589	oar-let-7a	2	11	260
circRNA464	oar-miR-654-3p	2	48	1080
circRNA389	oar-miR-16b	2	20	1470
circRNA588	oar-let-7c	2	20	1250
circRNA716	oar-miR-493-3p	2	83.5	2960
circRNA172	oar-miR-654-5p	2	21	847
circRNA760	oar-miR-136	2	158	8730
circRNA50	oar-miR-323a-5p	2	41	968
circRNA628	oar-miR-655-5p	2	58	2150

circRNA354	oar-miR-376a-5p	2	142	3660
circRNA103	oar-miR-370-3p	2	147	2290
circRNA755	oar-miR-1197-5p	2	107	3010
circRNA367	oar-miR-1185-5p	2	93.5	3400
circRNA296	oar-miR-654-5p	2	20	634
circRNA610	oar-miR-668-3p	2	83	2110
circRNA429	oar-miR-3957-5p	2	121	3760
circRNA101	oar-miR-3957-5p	2	4	119
circRNA436	oar-miR-134-5p	2	123	2820
circRNA168	oar-let-7a	2	45	1730
circRNA433	oar-miR-543-5p	2	213	1440
circRNA298	oar-miR-411b-5p	2	28	774
circRNA310	oar-miR-329b-5p	2	150	3620
circRNA368	oar-miR-136	2	128	5040
circRNA391	oar-miR-654-5p	2	19	358
circRNA331	oar-miR-654-5p	2	32	2510
circRNA766	oar-miR-181a	2	86.2	2720
circRNA719	oar-miR-541-5p	2	21	1150
circRNA693	oar-miR-541-5p	2	27	842
circRNA419	oar-let-7f	2	12	777
circRNA238	oar-miR-485-5p	2	93	4020
circRNA651	oar-miR-134-3p	2	78	1620
circRNA73	oar-miR-654-5p	2	11	559
circRNA459	oar-miR-323a-5p	2	31	1450
circRNA610	oar-miR-1193-5p	2	83	2110
circRNA431	oar-miR-654-5p	2	512	2590
circRNA770	oar-miR-487a-3p	2	7	452
circRNA168	oar-let-7f	2	45	1730
circRNA168	oar-let-7i	2	45	1730
circRNA773	oar-miR-382-5p	2	25	1110
circRNA432	oar-miR-543-5p	2	268	1640
circRNA302	oar-miR-654-5p	2	158	3480
circRNA736	oar-miR-16b	2	459	26700
circRNA55	oar-miR-154b-5p	2	23	1460
ciRNA79	oar-miR-1197-5p	2	35	2410
circRNA433	oar-miR-654-5p	2	213	1440
circRNA617	oar-miR-329b-5p	2	53	2970
circRNA783	oar-miR-485-5p	2	339	16100
circRNA524	oar-miR-3955-3p	2	16.5	1190
circRNA659	oar-miR-493-3p	2	125	6640
circRNA642	oar-miR-3957-5p	2	22	950
circRNA275	oar-miR-433-3p	2	165	2850
circRNA605	oar-miR-3956-5p	2	63	526
circRNA377	oar-miR-1197-3p	2	124	3890
circRNA315	oar-miR-411b-3p	2	32	1880
circRNA603	oar-miR-493-3p	2	13	873
circRNA442	oar-let-7c	2	32	1980
circRNA431	oar-miR-3956-5p	2	512	2590
circRNA129	oar-miR-380-5p	2	58	2800
circRNA705	oar-miR-1197-5p	2	20	657
circRNA87	oar-miR-411b-5p	2	22	1330
circRNA589	oar-let-7i	2	11	260
circRNA706	oar-miR-27a	2	40	938
circRNA168	oar-miR-376b-3p	2	45	1730
circRNA788	oar-miR-150	2	400	16300
circRNA182	oar-miR-16b	2	192	6140
ciRNA20	oar-miR-133	2	11	319
circRNA651	oar-miR-154b-5p	2	78	1620
circRNA168	oar-let-7g	2	45	1730
circRNA460	oar-miR-370-5p	2	249	6760
circRNA473	bta-miR-133	1	8	499
circRNA103	bta-miR-1	1	147	2290
circRNA439	bta-miR-206	1	146	3240
circRNA477	bta-miR-486	1	260	6440
ciRNA21	bta-miR-499	1	3	147
circRNA394	bta-miR-133	1	23	1490
circRNA358	bta-miR-133a	1	56	3900
circRNA290	bta-miR-1	1	75	4460
circRNA734	bta-miR-133a	1	37	1920
circRNA360	bta-miR-208	1	14	1030
circRNA63	bta-miR-1	1	49	2720
circRNA679	bta-miR-1	1	45	2240
ciRNA28	bta-miR-133	1	7	445
circRNA93	bta-miR-1	1	291	8600
circRNA497	bta-miR-133	1	47.5	1540
circRNA298	bta-miR-486	1	28	774
circRNA287	bta-miR-208	1	6	215
circRNA625	bta-miR-1	1	56	1680
ciRNA59	bta-miR-133	1	5	361
ciRNA28	bta-miR-133a	1	7	445
circRNA447	bta-miR-486	1	48	1760
circRNA486	bta-miR-486	1	13	615
circRNA409	bta-miR-133	1	26	1130
circRNA497	bta-miR-133a	1	47.5	1540
circRNA610	bta-miR-208	1	83	2110
circRNA384	bta-miR-133a	1	78	3290
circRNA48	bta-miR-1	1	37	992
circRNA209	bta-miR-133a	1	2	97.7
circRNA101	bta-miR-499	1	4	119
ciRNA20	bta-miR-133a	1	11	319
circRNA18	bta-miR-208	1	26	1100
circRNA506	bta-miR-133a	1	29	1530
circRNA196	bta-miR-206	1	15	1040
circRNA309	bta-miR-499	1	4	249
circRNA171	bta-miR-208	1	166	8060
circRNA472	bta-miR-208	1	35	1330
circRNA100	bta-miR-133a	1	20	767
circRNA473	bta-miR-133a	1	8	499

circRNA66	bta-miR-499	1	39	2330
circRNA723	bta-miR-133a	1	21	1160
circRNA700	bta-miR-499	1	33	2440
circRNA679	bta-miR-206	1	45	2240
circRNA290	bta-miR-206	1	75	4460
circRNA668	bta-miR-133	1	30	1850
circRNA22	bta-miR-206	1	255	5330
circRNA233	bta-miR-486	1	90	5650
circRNA753	bta-miR-486	1	109	3710
circRNA20	bta-miR-486	1	86	2300
circRNA282	bta-miR-1	1	6	334
ciRNA20	bta-miR-133	1	11	319
circRNA266	bta-miR-133a	1	95	5370
circRNA409	bta-miR-133a	1	26	1130
circRNA249	bta-miR-133a	1	357	11500
circRNA668	bta-miR-133a	1	30	1850
circRNA506	bta-miR-133	1	29	1530
circRNA323	bta-miR-133	1	8	555
circRNA656	bta-miR-133a	1	30	1290
circRNA208	bta-miR-208	1	71	4430
circRNA87	bta-miR-499	1	22	1330
circRNA316	bta-miR-499	1	19	1020
circRNA98	bta-miR-499	1	13	697
circRNA671	bta-miR-206	1	174	10400
circRNA653	bta-miR-499	1	16	332
circRNA209	bta-miR-133	1	2	97.7
circRNA723	bta-miR-133	1	21	1160
circRNA114	bta-miR-486	1	350	14700
circRNA663	bta-miR-133	1	29	1380
circRNA687	bta-miR-206	1	25	925
circRNA766	bta-miR-486	1	86.2	2720
circRNA71	bta-miR-133	1	34	1970
circRNA648	bta-miR-486	1	39.5	1810
circRNA781	bta-miR-208	1	8	399
circRNA718	bta-miR-206	1	4	334
circRNA72	bta-miR-486	1	35	1280
circRNA150	bta-miR-1	1	55	4530
circRNA150	bta-miR-206	1	55	4530
ciRNA83	bta-miR-499	1	8	572
circRNA322	bta-miR-133	1	130	4390
circRNA498	bta-miR-206	1	15	657
circRNA50	bta-miR-208	1	41	968
circRNA709	bta-miR-208	1	129	2940
circRNA144	bta-miR-499	1	16	798
circRNA281	bta-miR-206	1	17	594
circRNA266	bta-miR-133	1	95	5370
circRNA231	bta-miR-486	1	59	1860
circRNA717	bta-miR-133a	1	61.5	3250
circRNA166	bta-miR-133	1	171	4920
ciRNA2	bta-miR-486	1	6	267
ciRNA19	bta-miR-499	1	3	236
ciRNA23	bta-miR-133a	1	3	212
circRNA157	bta-miR-133	1	268	16300
circRNA42	bta-miR-1	1	193	3260
circRNA89	bta-miR-486	1	24	2180
ciRNA81	bta-miR-499	1	12	425
circRNA649	bta-miR-486	1	5.5	363
circRNA323	bta-miR-133a	1	8	555
circRNA424	bta-miR-208	1	48	2000
circRNA32	bta-miR-486	1	28	1400
circRNA173	bta-miR-486	1	38	1350
circRNA104	bta-miR-1	1	14	856
circRNA37	bta-miR-486	1	61	2680
ciRNA23	bta-miR-133	1	3	212
circRNA491	bta-miR-1	1	34	1470
circRNA439	bta-miR-1	1	146	3240
ciRNA62	bta-miR-486	1	6	277
circRNA499	bta-miR-133a	1	15	581
circRNA349	bta-miR-133a	1	11	694
circRNA282	bta-miR-206	1	6	334
circRNA728	bta-miR-486	1	3	182
circRNA91	bta-miR-208	1	38.7	2200
circRNA352	bta-miR-499	1	45	2060
circRNA637	bta-miR-133a	1	120	2400
circRNA166	bta-miR-133a	1	171	4920
circRNA83	bta-miR-499	1	235	8250
circRNA741	bta-miR-486	1	89	2700
circRNA681	bta-miR-133a	1	49	1570
circRNA91	bta-miR-1	1	38.7	2200
circRNA103	bta-miR-206	1	147	2290
circRNA717	bta-miR-133	1	61.5	3250
circRNA498	bta-miR-499	1	15	657
circRNA277	bta-miR-1	1	971	48700
circRNA349	bta-miR-133	1	11	694
circRNA1	bta-miR-133a	1	10	605
circRNA20	bta-miR-1	1	86	2300
ciRNA11	bta-miR-499	1	4	270
circRNA611	bta-miR-133	1	40	1830
circRNA637	bta-miR-133	1	120	2400
circRNA499	bta-miR-133	1	15	581
circRNA675	bta-miR-208	1	12	667
circRNA269	bta-miR-208	1	37	2060
circRNA341	bta-miR-499	1	107	4040
circRNA491	bta-miR-206	1	34	1470
circRNA101	bta-miR-208	1	4	119
circRNA391	bta-miR-133	1	19	358
circRNA586	bta-miR-499	1	12	765
circRNA71	bta-miR-133a	1	34	1970

circRNA743	bta-miR-499	1	291	8460
circRNA277	bta-miR-206	1	971	48700
circRNA517	bta-miR-1	1	9	460
circRNA182	bta-miR-486	1	192	6140
circRNA378	bta-miR-486	1	42.5	1830
circRNA205	bta-miR-133	1	34	1150
circRNA52	bta-miR-133	1	14	313
circRNA490	bta-miR-206	1	240	5160
circRNA20	bta-miR-206	1	86	2300
circRNA712	bta-miR-499	1	120	5180
circRNA551	bta-miR-1	1	193	4060
circRNA495	bta-miR-206	1	134	5930
circRNA157	bta-miR-133a	1	268	16300
circRNA752	bta-miR-133a	1	21	926
circRNA422	bta-miR-208	1	28	1210
circRNA28	bta-miR-208	1	23	872
circRNA22	bta-miR-208	1	255	5330
circRNA609	bta-miR-133a	1	49	1860
circRNA42	bta-miR-499	1	193	3260
ciRNA10	bta-miR-208	1	40	2950
circRNA752	bta-miR-133	1	21	926
circRNA275	bta-miR-206	1	165	2850
circRNA605	bta-miR-499	1	63	526
circRNA275	bta-miR-1	1	165	2850
circRNA1	bta-miR-133	1	10	605
circRNA281	bta-miR-1	1	17	594
ciRNA17	bta-miR-499	1	5	545
circRNA136	bta-miR-499	1	24	1000
circRNA663	bta-miR-133a	1	29	1380
circRNA590	bta-miR-499	1	81	5580
circRNA268	bta-miR-208	1	6	339
circRNA105	bta-miR-133	1	22	896
circRNA146	bta-miR-206	1	175	7890
circRNA42	bta-miR-206	1	193	3260
circRNA63	bta-miR-206	1	49	2720
ciRNA59	bta-miR-133a	1	5	361
circRNA642	bta-miR-208	1	22	950
circRNA716	bta-miR-133a	1	83.5	2960
circRNA272	bta-miR-499	1	67	969
circRNA656	bta-miR-133	1	30	1290
circRNA731	bta-miR-486	1	15	407
circRNA391	bta-miR-133a	1	19	358
circRNA496	bta-miR-486	1	90.5	3120
circRNA704	bta-miR-206	1	49	2550
circRNA1	bta-miR-486	1	10	605
circRNA169	bta-miR-486	1	24	1170
circRNA391	bta-miR-208	1	19	358
circRNA205	bta-miR-133a	1	34	1150
circRNA105	bta-miR-133a	1	22	896
circRNA54	bta-miR-133a	1	52	1810
circRNA261	bta-miR-486	1	384	11400
circRNA251	bta-miR-208	1	53	1050
circRNA91	bta-miR-499	1	38.7	2200
circRNA87	bta-miR-208	1	22	1330
circRNA582	bta-miR-499	1	39	1480
circRNA713	bta-miR-208	1	10	612
circRNA322	bta-miR-133a	1	130	4390
circRNA507	bta-miR-133a	1	166	4910
circRNA607	bta-miR-206	1	10	722
circRNA180	bta-miR-486	1	30	2030
circRNA768	bta-miR-486	1	43.7	1850
circRNA54	bta-miR-133	1	52	1810
circRNA772	bta-miR-486	1	34	1290
circRNA400	bta-miR-208	1	28	922
circRNA716	bta-miR-133	1	83.5	2960
circRNA168	bta-miR-208	1	45	1730
circRNA377	bta-miR-486	1	124	3890
circRNA495	bta-miR-1	1	134	5930
circRNA188	bta-miR-499	1	35	1300
ciRNA2	bta-miR-208	1	6	267
circRNA431	bta-miR-133a	1	512	2590
circRNA34	bta-miR-133	1	21	827
circRNA124	bta-miR-1	1	157	5240
circRNA517	bta-miR-206	1	9	460
circRNA184	bta-miR-206	1	42	2340
circRNA551	bta-miR-206	1	193	4060
circRNA204	bta-miR-486	1	26	1840
ciRNA8	bta-miR-499	1	2.5	137
circRNA249	bta-miR-133	1	357	11500
circRNA124	bta-miR-206	1	157	5240
circRNA681	bta-miR-133	1	49	1570
circRNA34	bta-miR-133a	1	21	827
circRNA100	bta-miR-133	1	20	767
circRNA207	bta-miR-486	1	45	1600
circRNA443	bta-miR-208	1	143	7000
circRNA358	bta-miR-133	1	56	3900
circRNA104	bta-miR-206	1	14	856
circRNA507	bta-miR-133	1	166	4910
circRNA734	bta-miR-133	1	37	1920
circRNA137	bta-miR-499	1	24	849
circRNA704	bta-miR-1	1	49	2550
circRNA17	bta-miR-208	1	62	2020
circRNA563	bta-miR-208	1	106	2740
circRNA609	bta-miR-133	1	49	1860
circRNA345	bta-miR-133a	1	32	1330
circRNA455	bta-miR-499	1	20	994
circRNA718	bta-miR-1	1	4	334
circRNA611	bta-miR-133a	1	40	1830

circRNA52	bta-miR-133a	1	14	313
circRNA711	bta-miR-499	1	63	2820
circRNA558	bta-miR-499	1	77	4250
circRNA775	bta-miR-1	1	17	410
circRNA743	bta-miR-208	1	291	8460
circRNA394	bta-miR-133a	1	23	1490
circRNA490	bta-miR-1	1	240	5160
circRNA120	bta-miR-499	1	20	965
circRNA91	bta-miR-206	1	38.7	2200
circRNA384	bta-miR-133	1	78	3290
circRNA146	bta-miR-1	1	175	7890
circRNA431	bta-miR-133	1	512	2590
circRNA22	bta-miR-1	1	255	5330
ciRNA74	bta-miR-206	1	5	138
circRNA345	bta-miR-133	1	32	1330
circRNA128	bta-miR-206	1	118	4950
circRNA111	oar-miR-376a-5p	1	181	4380
circRNA418	oar-miR-539-3p	1	32	1810
circRNA31	oar-miR-191	1	102	1620
circRNA279	oar-miR-143	1	71	3110
circRNA291	oar-miR-329a-5p	1	23	830
circRNA278	oar-miR-485-5p	1	65	2960
circRNA37	oar-miR-493-3p	1	61	2680
ciRNA16	oar-miR-431	1	23.5	1170
circRNA265	oar-miR-21	1	15	215
circRNA781	oar-miR-26b	1	8	399
circRNA662	oar-miR-379-5p	1	63	1980
circRNA410	oar-miR-329b-3p	1	160	4560
circRNA647	oar-miR-323a-5p	1	110	5290
circRNA746	oar-miR-665-3p	1	108	3350
circRNA431	oar-miR-323a-3p	1	512	2590
ciRNA20	oar-miR-379-5p	1	11	319
circRNA346	oar-miR-654-5p	1	15	570
circRNA706	oar-miR-1197-5p	1	40	938
circRNA742	oar-miR-376c-5p	1	63	2210
circRNA377	oar-miR-411a-5p	1	124	3890
circRNA211	oar-miR-654-5p	1	16	820
circRNA273	oar-miR-329b-5p	1	59	1890
circRNA247	oar-miR-3957-5p	1	41	1110
circRNA16	oar-miR-539-3p	1	26	1330
circRNA78	oar-miR-487b-5p	1	47	1420
circRNA464	oar-miR-25	1	48	1080
circRNA314	oar-miR-30c	1	23	1150
circRNA487	oar-miR-27a	1	33	1440
circRNA89	oar-miR-493-3p	1	24	2180
circRNA338	oar-miR-107	1	45.5	2180
circRNA588	oar-let-7i	1	20	1250
circRNA445	oar-miR-299-3p	1	17	958
ciRNA59	oar-miR-133	1	5	361
circRNA320	oar-miR-143	1	31	1980
circRNA182	oar-miR-148a	1	192	6140
circRNA407	oar-miR-412-3p	1	44	2870
circRNA111	oar-miR-143	1	181	4380
circRNA291	oar-miR-99a	1	23	830
circRNA279	oar-let-7c	1	71	3110
circRNA352	oar-miR-3956-5p	1	45	2060
circRNA734	oar-miR-329a-5p	1	37	1920
circRNA14	oar-miR-411b-5p	1	99	3350
circRNA262	oar-miR-134-5p	1	24	975
ciRNA47	oar-miR-125b	1	2	156
circRNA602	oar-miR-544-5p	1	197	11100
circRNA451	oar-miR-381-5p	1	34	2270
circRNA142	oar-miR-485-5p	1	76	5370
circRNA515	oar-miR-376d	1	17	663
circRNA248	oar-miR-3958-5p	1	6	370
circRNA151	oar-miR-200b	1	199	11100
circRNA687	oar-let-7d	1	25	925
circRNA389	oar-miR-200a	1	20	1470
circRNA16	oar-miR-136	1	26	1330
circRNA597	oar-miR-409-3p	1	59	2680
circRNA764	oar-let-7b	1	159	6640
circRNA250	oar-miR-1197-5p	1	14	743
circRNA181	oar-let-7a	1	28	916
circRNA211	oar-miR-381-5p	1	16	820
circRNA349	oar-miR-668-5p	1	11	694
circRNA742	oar-miR-136	1	63	2210
circRNA367	oar-miR-30a-3p	1	93.5	3400
circRNA678	oar-miR-194	1	24	1290
circRNA106	oar-miR-411b-5p	1	60	2410
circRNA777	oar-miR-329a-3p	1	10	409
circRNA754	oar-miR-494-5p	1	32	2040
circRNA777	oar-miR-493-5p	1	10	409
circRNA295	oar-miR-496-5p	1	485	13200
circRNA23	oar-miR-1185-5p	1	70	5360
circRNA146	oar-miR-329a-3p	1	175	7890
circRNA97	oar-let-7i	1	22	1350
circRNA557	oar-miR-539-5p	1	203	9800
circRNA705	oar-miR-377-3p	1	20	657
ciRNA8	oar-miR-544-5p	1	2.5	137
circRNA140	oar-miR-376a-5p	1	10	719
circRNA393	oar-miR-376a-5p	1	9	450
circRNA262	oar-miR-154a-5p	1	24	975
circRNA434	oar-miR-323a-3p	1	167	1280
circRNA119	oar-let-7i	1	3	171
circRNA265	oar-miR-143	1	15	215
circRNA363	oar-miR-654-5p	1	40.5	3530
circRNA551	oar-miR-380-3p	1	193	4060
circRNA103	oar-let-7d	1	147	2290

circRNA685	oar-miR-299-3p	1	172	6290
circRNA234	oar-miR-433-5p	1	31	1310
circRNA55	oar-miR-494-5p	1	23	1460
circRNA355	oar-miR-380-5p	1	342	13100
circRNA302	oar-miR-541-5p	1	158	3480
circRNA116	oar-miR-487a-3p	1	342	14200
circRNA229	oar-miR-432	1	48	845
circRNA766	oar-miR-200b	1	86.2	2720
circRNA304	oar-miR-487b-3p	1	262	7540
circRNA116	oar-let-7c	1	342	14200
circRNA31	oar-miR-412-3p	1	102	1620
circRNA364	oar-miR-154b-3p	1	140	6320
circRNA476	oar-miR-29b	1	81	2660
ciRNA27	oar-miR-665-3p	1	25	2040
circRNA747	oar-miR-409-3p	1	26	1800
circRNA361	oar-let-7c	1	5	304
circRNA478	oar-miR-221	1	167	6990
circRNA108	oar-miR-3959-3p	1	94	2680
circRNA230	oar-let-7d	1	12	602
circRNA15	oar-miR-487a-3p	1	21	1030
circRNA2	oar-miR-1193-5p	1	128	3720
circRNA173	oar-miR-370-3p	1	38	1350
circRNA201	oar-let-7b	1	11	812
circRNA5	oar-miR-17-5p	1	23	942
circRNA680	oar-miR-380-5p	1	14	820
circRNA403	oar-miR-27a	1	32	1980
circRNA97	oar-miR-432	1	22	1350
circRNA361	oar-let-7g	1	5	304
circRNA239	oar-miR-125b	1	3	236
circRNA639	oar-miR-1197-3p	1	71	3330
ciRNA27	oar-miR-154a-5p	1	25	2040
circRNA744	oar-miR-181a	1	56	1790
circRNA230	oar-let-7a	1	12	602
circRNA442	oar-miR-665-5p	1	32	1980
circRNA584	oar-miR-29a	1	318	12200
circRNA660	oar-miR-3957-3p	1	5	481
circRNA305	oar-miR-30a-3p	1	52	2410
circRNA629	oar-miR-369-5p	1	57	1900
circRNA465	oar-miR-377-5p	1	694	36800
circRNA550	oar-miR-495-5p	1	59	1040
circRNA662	oar-miR-134-5p	1	63	1980
circRNA310	oar-miR-200b	1	150	3620
circRNA669	oar-miR-3959-5p	1	178	6140
circRNA286	oar-miR-218a	1	50	1870
circRNA444	oar-miR-1185-5p	1	29	1310
circRNA221	oar-miR-376d	1	74	2240
circRNA427	oar-miR-3958-3p	1	19	551
circRNA504	oar-let-7f	1	123	5780
ciRNA44	oar-miR-329a-3p	1	4	227
circRNA271	oar-miR-362	1	27	1890
circRNA495	oar-miR-376d	1	134	5930
circRNA276	oar-miR-541-5p	1	8	332
circRNA37	oar-miR-539-3p	1	61	2680
circRNA369	oar-miR-381-5p	1	33	1560
circRNA649	oar-miR-133	1	5.5	363
circRNA353	oar-miR-29b	1	21	1130
circRNA744	oar-let-7b	1	56	1790
circRNA298	oar-miR-370-5p	1	28	774
circRNA381	oar-let-7d	1	29	1720
circRNA137	oar-miR-379-5p	1	24	849
circRNA446	oar-miR-103	1	38	1460
circRNA319	oar-let-7a	1	42	1620
circRNA115	oar-miR-369-5p	1	280	9430
circRNA623	oar-miR-433-5p	1	91	4670
circRNA416	oar-miR-22-3p	1	28	1680
ciRNA44	oar-miR-127	1	4	227
circRNA256	oar-miR-381-3p	1	58	4320
circRNA23	oar-miR-26b	1	70	5360
circRNA183	oar-miR-654-5p	1	189	9040
circRNA517	oar-miR-411a-5p	1	9	460
circRNA200	oar-miR-370-5p	1	73	2220
circRNA687	oar-miR-380-5p	1	25	925
circRNA186	oar-miR-668-5p	1	69	4270
circRNA351	oar-miR-369-5p	1	23	1580
circRNA94	oar-miR-376a-5p	1	89	6150
circRNA636	oar-miR-23a	1	49	2080
circRNA607	oar-miR-103	1	10	722
ciRNA20	oar-miR-154b-5p	1	11	319
circRNA641	oar-miR-376d	1	14	853
circRNA96	oar-miR-1185-5p	1	44	3060
ciRNA86	oar-miR-329b-3p	1	35.3	2550
ciRNA62	oar-miR-10a	1	6	277
circRNA10	oar-miR-412-5p	1	10	483
circRNA297	oar-miR-1193-5p	1	12	698
circRNA662	oar-let-7d	1	63	1980
circRNA581	oar-miR-411b-3p	1	14	835
circRNA628	oar-miR-543-5p	1	58	2150
ciRNA93	oar-miR-30a-3p	1	3	254
circRNA613	oar-miR-370-3p	1	21	735
circRNA198	oar-miR-3957-5p	1	52	2760
circRNA215	oar-miR-1185-3p	1	11	948
circRNA565	oar-let-7c	1	45	1450
circRNA444	oar-miR-26a	1	29	1310
circRNA59	oar-miR-493-3p	1	73	3560
circRNA756	oar-miR-23b	1	68	2660
circRNA602	oar-miR-200b	1	197	11100
circRNA145	oar-miR-376a-5p	1	79	3280
circRNA58	oar-miR-431	1	24	1080

circRNA220	oar-miR-665-3p	1	17	894
circRNA403	oar-miR-495-5p	1	32	1980
ciRNA57	oar-miR-493-3p	1	3	276
circRNA145	oar-miR-3959-3p	1	79	3280
circRNA288	oar-miR-485-5p	1	37	1330
circRNA75	oar-miR-495-5p	1	45	1530
circRNA678	oar-miR-200c	1	24	1290
circRNA628	oar-miR-323a-3p	1	58	2150
circRNA383	oar-let-7g	1	11	479
circRNA773	oar-miR-3958-5p	1	25	1110
ciRNA62	oar-miR-494-3p	1	6	277
circRNA367	oar-miR-148a	1	93.5	3400
circRNA240	oar-miR-125b	1	114	4230
ciRNA30	oar-miR-16b	1	2	84.4
circRNA576	oar-miR-541-3p	1	21	1320
circRNA588	oar-miR-495-5p	1	20	1250
circRNA57	oar-miR-25	1	138	7370
circRNA527	oar-miR-30c	1	48	2150
circRNA33	oar-miR-493-5p	1	34	1890
circRNA16	oar-miR-30a-3p	1	26	1330
circRNA224	oar-miR-154b-5p	1	37	1600
circRNA471	oar-miR-148a	1	63	1820
circRNA599	oar-miR-29b	1	6	298
circRNA188	oar-miR-181a	1	35	1300
circRNA611	oar-miR-1185-5p	1	40	1830
circRNA773	oar-miR-136	1	25	1110
circRNA606	oar-miR-3955-5p	1	95	3890
circRNA354	oar-miR-29b	1	142	3660
circRNA577	oar-miR-148a	1	60	3480
circRNA522	oar-miR-376d	1	39	1470
circRNA95	oar-miR-376b-3p	1	28	1150
ciRNA37	oar-miR-1193-3p	1	22	666
circRNA342	oar-miR-30a-5p	1	45	2070
circRNA718	oar-miR-181a	1	4	334
circRNA292	oar-let-7i	1	13	484
circRNA406	oar-miR-150	1	148	5360
circRNA646	oar-let-7i	1	19	876
circRNA464	oar-miR-134-5p	1	48	1080
circRNA73	oar-miR-1197-5p	1	11	559
circRNA706	oar-miR-10b	1	40	938
circRNA539	oar-miR-433-5p	1	29	1360
circRNA41	oar-miR-107	1	9	617
circRNA78	oar-miR-409-5p	1	47	1420
circRNA266	oar-miR-29b	1	95	5370
circRNA21	oar-miR-376e-5p	1	12	695
circRNA361	oar-miR-377-5p	1	5	304
circRNA744	oar-miR-411a-5p	1	56	1790
circRNA657	oar-miR-412-5p	1	19	843
circRNA67	oar-miR-376a-3p	1	26	1430
circRNA269	oar-let-7i	1	37	2060
circRNA30	oar-miR-329a-5p	1	62	4220
circRNA391	oar-miR-1193-5p	1	19	358
ciRNA96	oar-miR-432	1	6	625
circRNA536	oar-miR-106b	1	87	4870
circRNA641	oar-let-7f	1	14	853
circRNA197	oar-miR-433-3p	1	37	2200
circRNA170	oar-miR-154a-5p	1	74	1320
circRNA272	oar-miR-544-3p	1	67	969
circRNA430	oar-miR-539-5p	1	319	2550
circRNA534	oar-miR-3955-5p	1	18	721
circRNA773	oar-let-7b	1	25	1110
circRNA49	oar-miR-143	1	23	1490
circRNA314	oar-miR-3955-3p	1	23	1150
circRNA111	oar-miR-493-5p	1	181	4380
circRNA762	oar-miR-3959-3p	1	24.5	1680
circRNA628	oar-miR-194	1	58	2150
circRNA175	oar-miR-376a-3p	1	9	475
circRNA24	oar-miR-1193-3p	1	34	2370
circRNA484	oar-miR-370-3p	1	135	4150
circRNA47	oar-miR-376a-5p	1	122	5840
circRNA560	oar-miR-323a-5p	1	24	859
ciRNA28	oar-miR-665-5p	1	7	445
circRNA241	oar-miR-154b-5p	1	45	2440
circRNA338	oar-miR-199a-3p	1	45.5	2180
ciRNA54	oar-miR-134-3p	1	22	1530
circRNA398	oar-miR-495-5p	1	135	4930
circRNA346	oar-miR-487a-5p	1	15	570
circRNA110	oar-miR-655-5p	1	62	1800
circRNA22	oar-miR-758-3p	1	255	5330
ciRNA90	oar-miR-1197-5p	1	48	4390
circRNA323	oar-let-7g	1	8	555
circRNA716	oar-miR-1193-3p	1	83.5	2960
circRNA560	oar-miR-329b-5p	1	24	859
circRNA522	oar-miR-411b-3p	1	39	1470
circRNA11	oar-let-7b	1	11	576
circRNA540	oar-miR-29b	1	87	3030
circRNA734	oar-miR-329b-3p	1	37	1920
circRNA467	oar-miR-1197-3p	1	28	1050
circRNA170	oar-miR-543-5p	1	74	1320
circRNA279	oar-miR-25	1	71	3110
circRNA96	oar-miR-23a	1	44	3060
circRNA319	oar-miR-544-3p	1	42	1620
circRNA354	oar-miR-329b-5p	1	142	3660
circRNA107	oar-miR-411a-5p	1	96	4820
circRNA252	oar-miR-323a-5p	1	612	11700
circRNA233	oar-miR-199a-3p	1	90	5650
circRNA468	oar-miR-668-3p	1	31	2170
circRNA274	oar-miR-323c	1	66	2070

ciRNA2	oar-miR-376e-5p	1	6	267
circRNA723	oar-miR-133	1	21	1160
circRNA721	oar-miR-103	1	174	9120
circRNA592	oar-miR-134-5p	1	68	1670
circRNA478	oar-miR-376e-5p	1	167	6990
circRNA101	oar-miR-665-3p	1	4	119
circRNA272	oar-miR-411a-3p	1	67	969
circRNA636	oar-let-7i	1	49	2080
circRNA49	oar-miR-22-3p	1	23	1490
circRNA42	oar-miR-3957-5p	1	193	3260
circRNA728	oar-miR-381-5p	1	3	182
circRNA640	oar-let-7i	1	59	2230
circRNA507	oar-miR-411a-5p	1	166	4910
circRNA164	oar-miR-3956-3p	1	107	5430
circRNA347	oar-miR-376e-3p	1	44	2980
circRNA244	oar-miR-409-5p	1	71	3830
circRNA787	oar-miR-493-5p	1	83.5	5290
circRNA172	oar-miR-410-5p	1	21	847
circRNA322	oar-miR-412-3p	1	130	4390
circRNA112	oar-miR-544-5p	1	112	4570
circRNA57	oar-miR-133	1	138	7370
circRNA114	oar-miR-30b	1	350	14700
circRNA307	oar-miR-3956-5p	1	341	21400
circRNA341	oar-miR-154b-5p	1	107	4040
circRNA221	oar-miR-154b-3p	1	74	2240
circRNA512	oar-miR-200b	1	24	1520
circRNA463	oar-miR-410-5p	1	40	2200
circRNA621	oar-miR-541-5p	1	46	3840
ciRNA1	oar-miR-409-3p	1	4	354
circRNA764	oar-let-7i	1	159	6640
circRNA740	oar-miR-148a	1	11	815
circRNA406	oar-miR-134-5p	1	148	5360
circRNA61	oar-miR-27a	1	17	681
circRNA180	oar-let-7a	1	30	2030
circRNA192	oar-miR-494-3p	1	104	3910
circRNA252	oar-miR-22-3p	1	612	11700
circRNA783	oar-miR-665-5p	1	339	16100
circRNA311	oar-miR-544-5p	1	32	2310
ciRNA49	oar-miR-411a-3p	1	6	449
circRNA35	oar-miR-1197-3p	1	34	1030
circRNA422	oar-miR-380-3p	1	28	1210
circRNA318	oar-miR-194	1	55	2410
circRNA775	oar-let-7f	1	17	410
circRNA697	oar-miR-329a-3p	1	21	1080
circRNA582	oar-miR-107	1	39	1480
circRNA24	oar-let-7d	1	34	2370
circRNA522	oar-miR-433-5p	1	39	1470
circRNA106	oar-miR-493-3p	1	60	2410
ciRNA52	oar-let-7c	1	8	399
circRNA381	oar-miR-377-5p	1	29	1720
circRNA391	oar-let-7f	1	19	358
circRNA410	oar-miR-668-5p	1	160	4560
circRNA39	oar-let-7d	1	11	513
circRNA287	oar-miR-654-5p	1	6	215
circRNA336	oar-miR-379-5p	1	24	1370
circRNA631	oar-miR-431	1	379	9240
circRNA639	oar-miR-379-3p	1	71	3330
circRNA540	oar-miR-758-3p	1	87	3030
circRNA42	oar-miR-136	1	193	3260
circRNA34	oar-miR-194	1	21	827
circRNA630	oar-miR-654-3p	1	35	2060
circRNA651	oar-miR-26a	1	78	1620
circRNA154	oar-miR-3959-3p	1	27	1160
circRNA149	oar-miR-376c-5p	1	71	3740
circRNA314	oar-miR-218a	1	23	1150
circRNA176	oar-miR-668-5p	1	24	1150
circRNA308	oar-miR-329b-5p	1	2	146
circRNA368	oar-miR-199a-3p	1	128	5040
ciRNA82	oar-miR-665-5p	1	2	137
circRNA565	oar-miR-134-5p	1	45	1450
circRNA264	oar-miR-221	1	88	5460
circRNA11	oar-miR-329b-5p	1	11	576
circRNA591	oar-miR-30c	1	21	853
circRNA372	oar-let-7i	1	30	1200
ciRNA30	oar-miR-487a-3p	1	2	84.4
circRNA48	oar-miR-381-5p	1	37	992
circRNA572	oar-miR-200a	1	42	3170
circRNA496	oar-let-7a	1	90.5	3120
circRNA225	oar-miR-411a-5p	1	29	1590
circRNA236	oar-miR-362	1	181	4960
circRNA499	oar-miR-125b	1	15	581
circRNA51	oar-miR-377-3p	1	20	664
circRNA280	oar-miR-154b-5p	1	30	1530
ciRNA81	oar-miR-194	1	12	425
circRNA560	oar-miR-758-3p	1	24	859
circRNA646	oar-miR-496-5p	1	19	876
circRNA500	oar-let-7d	1	45	1680
circRNA304	oar-miR-487a-5p	1	262	7540
circRNA325	oar-miR-369-5p	1	39.5	1570
circRNA310	oar-miR-3957-5p	1	150	3620
circRNA6	oar-miR-377-5p	1	67	3600
circRNA440	oar-miR-485-5p	1	19	1310
circRNA380	oar-miR-541-5p	1	16	944
circRNA705	oar-miR-539-3p	1	20	657
circRNA713	oar-miR-133	1	10	612
circRNA452	oar-miR-107	1	5	470
circRNA671	oar-miR-409-3p	1	174	10400
circRNA26	oar-miR-494-3p	1	3	157

circRNA448	oar-miR-411b-3p	1	7	450
ciRNA33	oar-miR-654-5p	1	3	309
circRNA322	oar-miR-376b-3p	1	130	4390
circRNA680	oar-miR-487b-3p	1	14	820
circRNA217	oar-miR-136	1	93	4010
circRNA536	oar-miR-106a	1	87	4870
circRNA188	oar-miR-496-3p	1	35	1300
circRNA310	oar-miR-106b	1	150	3620
circRNA436	oar-miR-3956-3p	1	123	2820
circRNA501	oar-miR-16b	1	218	11300
circRNA51	oar-miR-655-5p	1	20	664
circRNA252	oar-miR-1193-3p	1	612	11700
circRNA413	oar-miR-299-5p	1	4	352
circRNA713	oar-miR-487a-3p	1	10	612
circRNA287	oar-miR-154b-3p	1	6	215
circRNA713	oar-miR-493-5p	1	10	612
circRNA214	oar-miR-541-5p	1	195	10000
circRNA52	oar-miR-380-3p	1	14	313
circRNA550	oar-let-7c	1	59	1040
circRNA148	oar-miR-495-5p	1	213	12600
circRNA441	oar-miR-134-3p	1	21	626
circRNA610	oar-miR-381-5p	1	83	2110
circRNA670	oar-miR-3955-5p	1	364	16900
circRNA154	oar-let-7g	1	27	1160
circRNA279	oar-let-7f	1	71	3110
circRNA685	oar-miR-410-5p	1	172	6290
circRNA377	oar-miR-1193-5p	1	124	3890
circRNA452	oar-miR-432	1	5	470
circRNA154	oar-let-7c	1	27	1160
circRNA568	oar-miR-377-5p	1	166	4830
circRNA725	oar-miR-329b-5p	1	79	4390
circRNA724	oar-miR-323a-5p	1	8	540
circRNA483	oar-miR-411b-3p	1	23	1250
circRNA410	oar-miR-654-5p	1	160	4560
circRNA527	oar-miR-543-3p	1	48	2150
ciRNA27	oar-miR-493-5p	1	25	2040
circRNA67	oar-miR-376c-5p	1	26	1430
circRNA646	oar-miR-3957-3p	1	19	876
circRNA785	oar-miR-154b-5p	1	2	135
circRNA435	oar-miR-758-3p	1	20.2	602
circRNA3	oar-miR-191	1	99	4710
circRNA691	oar-miR-154a-3p	1	8	474
ciRNA77	oar-miR-29a	1	1	98
circRNA778	oar-miR-10a	1	24	1210
circRNA651	oar-miR-377-5p	1	78	1620
circRNA200	oar-miR-134-3p	1	73	2220
circRNA600	oar-miR-329b-5p	1	34	1850
circRNA640	oar-miR-23a	1	59	2230
circRNA593	oar-miR-412-3p	1	37	1850
circRNA512	oar-miR-3955-5p	1	24	1520
circRNA246	oar-miR-99a	1	53	1940
circRNA693	oar-miR-134-5p	1	27	842
circRNA481	oar-miR-493-3p	1	44	2590
circRNA481	oar-let-7g	1	44	2590
circRNA254	oar-miR-3957-5p	1	14	944
circRNA25	oar-miR-411a-5p	1	27	1840
circRNA31	oar-miR-665-5p	1	102	1620
circRNA366	oar-miR-30a-3p	1	6.5	463
circRNA539	oar-let-7i	1	29	1360
circRNA604	oar-miR-154b-3p	1	70	3730
circRNA78	oar-miR-485-5p	1	47	1420
circRNA747	oar-miR-382-3p	1	26	1800
circRNA172	oar-miR-323a-5p	1	21	847
circRNA593	oar-let-7i	1	37	1850
circRNA770	oar-miR-376a-3p	1	7	452
circRNA686	oar-miR-376a-3p	1	30	923
circRNA180	oar-miR-432	1	30	2030
circRNA678	oar-miR-3955-3p	1	24	1290
circRNA640	oar-let-7a	1	59	2230
circRNA129	oar-miR-431	1	58	2800
circRNA205	oar-let-7f	1	34	1150
ciRNA93	oar-miR-329a-5p	1	3	254
circRNA355	oar-miR-411b-5p	1	342	13100
circRNA445	oar-miR-3957-5p	1	17	958
circRNA707	oar-miR-380-3p	1	71	2950
circRNA100	oar-miR-3959-5p	1	20	767
circRNA498	oar-miR-432	1	15	657
circRNA52	oar-miR-30a-3p	1	14	313
circRNA538	oar-miR-485-5p	1	41	1830
circRNA398	oar-miR-410-3p	1	135	4930
circRNA234	oar-miR-412-3p	1	31	1310
circRNA199	oar-miR-377-5p	1	14	653
circRNA182	oar-miR-487a-3p	1	192	6140
circRNA362	oar-miR-655-5p	1	249	13300
circRNA95	oar-miR-3955-5p	1	28	1150
circRNA219	oar-miR-411a-3p	1	8	439
circRNA522	oar-miR-221	1	39	1470
circRNA209	oar-miR-495-3p	1	2	97.7
ciRNA46	oar-miR-493-3p	1	3	201
circRNA485	oar-miR-377-5p	1	333	27400
circRNA91	oar-miR-23b	1	38.7	2200
circRNA565	oar-let-7a	1	45	1450
circRNA486	oar-miR-495-3p	1	13	615
circRNA245	oar-miR-493-5p	1	26	1730
circRNA30	oar-let-7g	1	62	4220
circRNA296	oar-miR-541-3p	1	20	634
circRNA477	oar-miR-362	1	260	6440
circRNA648	oar-miR-10b	1	39.5	1810

circRNA408	oar-miR-25	1	42	2710
circRNA459	oar-miR-152	1	31	1450
circRNA471	oar-miR-379-3p	1	63	1820
circRNA582	oar-miR-376e-5p	1	39	1480
circRNA68	oar-miR-134-3p	1	5	305
circRNA698	oar-miR-154b-5p	1	47	2950
circRNA491	oar-miR-541-3p	1	34	1470
circRNA637	oar-miR-496-5p	1	120	2400
circRNA637	oar-miR-379-5p	1	120	2400
circRNA512	oar-miR-432	1	24	1520
ciRNA68	oar-miR-543-5p	1	5	376
circRNA108	oar-let-7c	1	94	2680
circRNA353	oar-miR-106a	1	21	1130
circRNA206	oar-miR-134-3p	1	20	1050
circRNA706	oar-miR-134-3p	1	40	938
circRNA300	oar-miR-541-3p	1	142	5630
ciRNA45	oar-let-7a	1	4	312
circRNA691	oar-miR-485-3p	1	8	474
circRNA21	oar-miR-376b-5p	1	12	695
circRNA163	oar-miR-299-5p	1	28	1180
circRNA272	oar-miR-329b-5p	1	67	969
circRNA533	oar-miR-25	1	53	1980
circRNA559	oar-miR-17-5p	1	18	1220
circRNA128	oar-miR-107	1	118	4950
circRNA722	oar-miR-665-3p	1	33	1630
circRNA265	oar-miR-23b	1	15	215
circRNA44	oar-miR-221	1	61	1930
circRNA634	oar-miR-299-3p	1	20	1240
ciRNA85	oar-miR-1197-5p	1	35.3	2570
circRNA742	oar-miR-221	1	63	2210
circRNA744	oar-miR-323a-3p	1	56	1790
circRNA765	oar-let-7c	1	15	760
circRNA141	oar-miR-362	1	60	3370
circRNA120	oar-miR-377-5p	1	20	965
circRNA102	oar-miR-221	1	13	681
circRNA396	oar-miR-380-5p	1	42	3510
circRNA273	oar-miR-329a-5p	1	59	1890
circRNA272	oar-miR-22-3p	1	67	969
circRNA122	oar-miR-154b-5p	1	177	5560
circRNA520	oar-miR-154a-5p	1	8	740
circRNA254	oar-miR-539-3p	1	14	944
circRNA608	oar-miR-3957-5p	1	33	1990
circRNA691	oar-miR-487a-3p	1	8	474
circRNA573	oar-let-7a	1	397	22100
circRNA781	oar-miR-3956-3p	1	8	399
circRNA471	oar-miR-541-5p	1	63	1820
ciRNA81	oar-miR-329a-3p	1	12	425
circRNA266	oar-miR-29a	1	95	5370
circRNA72	oar-miR-412-5p	1	35	1280
circRNA674	oar-miR-543-5p	1	16	825
circRNA266	oar-miR-3957-5p	1	95	5370
circRNA640	oar-miR-181a	1	59	2230
circRNA684	oar-miR-3958-3p	1	70	5310
circRNA472	oar-miR-495-5p	1	35	1330
circRNA422	oar-miR-199a-3p	1	28	1210
circRNA384	oar-miR-27a	1	78	3290
circRNA42	oar-miR-668-3p	1	193	3260
circRNA71	oar-miR-370-5p	1	34	1970
circRNA460	oar-miR-362	1	249	6760
circRNA471	oar-miR-3955-3p	1	63	1820
ciRNA52	oar-miR-370-3p	1	8	399
circRNA728	oar-miR-376e-3p	1	3	182
circRNA29	oar-let-7i	1	53	1680
circRNA493	oar-miR-133	1	20.5	1330
circRNA706	oar-miR-200c	1	40	938
circRNA153	oar-miR-541-5p	1	19	1110
circRNA439	oar-miR-1193-5p	1	146	3240
circRNA558	oar-miR-432	1	77	4250
circRNA516	oar-miR-154b-3p	1	14	976
circRNA586	oar-miR-133	1	12	765
circRNA248	oar-miR-107	1	6	370
circRNA31	oar-miR-382-5p	1	102	1620
circRNA372	oar-let-7b	1	30	1200
circRNA97	oar-miR-654-5p	1	22	1350
circRNA384	oar-miR-495-3p	1	78	3290
circRNA167	oar-miR-194	1	14	900
circRNA415	oar-miR-154a-3p	1	49	1620
circRNA425	oar-miR-370-5p	1	36	1090
circRNA534	oar-miR-21	1	18	721
circRNA732	oar-miR-370-3p	1	16	538
circRNA206	oar-miR-493-5p	1	20	1050
circRNA136	oar-miR-411b-5p	1	24	1000
circRNA44	oar-let-7g	1	61	1930
circRNA104	oar-miR-3956-3p	1	14	856
circRNA341	oar-miR-758-3p	1	107	4040
circRNA200	oar-miR-143	1	73	2220
ciRNA92	oar-let-7a	1	7	562
circRNA374	oar-miR-1197-5p	1	33	1050
circRNA59	oar-miR-668-3p	1	73	3560
circRNA562	oar-miR-376b-5p	1	4	368
circRNA205	oar-let-7b	1	34	1150
circRNA313	oar-miR-1193-5p	1	235	11700
circRNA423	oar-miR-362	1	17	1070
circRNA653	oar-miR-3958-5p	1	16	332
circRNA419	oar-let-7i	1	12	777
circRNA294	oar-miR-412-5p	1	158	8020
circRNA525	oar-miR-1197-5p	1	62	2050
circRNA244	oar-miR-99a	1	71	3830

ciRNA88	oar-miR-665-5p	1	8	668
circRNA281	oar-let-7c	1	17	594
circRNA356	oar-let-7f	1	152	6210
circRNA138	oar-miR-379-5p	1	10	640
circRNA10	oar-miR-485-3p	1	10	483
circRNA288	oar-miR-539-5p	1	37	1330
circRNA751	oar-miR-494-3p	1	126	7010
circRNA48	oar-miR-487b-5p	1	37	992
circRNA17	oar-miR-1193-5p	1	62	2020
circRNA321	oar-miR-1197-3p	1	48	2450
circRNA613	oar-miR-221	1	21	735
ciRNA98	oar-miR-376e-3p	1	6	411
ciRNA67	oar-miR-494-5p	1	6	537
circRNA451	oar-miR-377-5p	1	34	2270
circRNA498	oar-miR-3955-5p	1	15	657
circRNA147	oar-miR-1197-5p	1	12	556
circRNA571	oar-miR-181a	1	25	1300
circRNA180	oar-let-7g	1	30	2030
circRNA181	oar-miR-411a-5p	1	28	916
circRNA686	oar-miR-30a-3p	1	30	923
circRNA419	oar-let-7d	1	12	777
circRNA24	oar-miR-411b-3p	1	34	2370
circRNA366	oar-miR-23a	1	6.5	463
circRNA205	oar-miR-26a	1	34	1150
circRNA346	oar-miR-3959-3p	1	15	570
circRNA40	oar-miR-411b-5p	1	22	1130
circRNA522	oar-miR-152	1	39	1470
circRNA678	oar-miR-495-3p	1	24	1290
circRNA125	oar-let-7i	1	74	3930
ciRNA13	oar-miR-127	1	359	32500
circRNA600	oar-miR-218a	1	34	1850
circRNA640	oar-miR-376b-3p	1	59	2230
circRNA113	oar-miR-544-5p	1	52	3060
circRNA568	oar-miR-299-5p	1	166	4830
circRNA402	oar-miR-431	1	29	1790
circRNA90	oar-let-7c	1	239	12600
circRNA614	oar-miR-329b-5p	1	50	1590
circRNA29	oar-miR-665-3p	1	53	1680
circRNA275	oar-miR-181a	1	165	2850
circRNA462	oar-miR-16b	1	43	1210
circRNA427	oar-miR-106b	1	19	551
ciRNA25	oar-miR-199a-3p	1	7	743
circRNA574	oar-miR-382-3p	1	59	4470
ciRNA91	oar-miR-370-5p	1	1	109
circRNA214	oar-let-7b	1	195	10000
circRNA354	oar-miR-370-3p	1	142	3660
circRNA219	oar-miR-3955-5p	1	8	439
circRNA34	oar-miR-493-5p	1	21	827
circRNA225	oar-miR-323a-5p	1	29	1590
circRNA649	oar-miR-23a	1	5.5	363
circRNA776	oar-miR-654-3p	1	27	331
circRNA198	oar-miR-432	1	52	2760
circRNA243	oar-miR-3956-3p	1	26	1480
circRNA776	oar-miR-323a-5p	1	27	331
circRNA78	oar-miR-3955-5p	1	47	1420
circRNA655	oar-miR-3958-5p	1	10	623
circRNA696	oar-let-7i	1	10	490
circRNA559	oar-miR-329a-5p	1	18	1220
circRNA499	oar-miR-485-5p	1	15	581
circRNA442	oar-miR-181a	1	32	1980
circRNA395	oar-miR-23a	1	31	1450
circRNA221	oar-let-7g	1	74	2240
circRNA605	oar-miR-1193-3p	1	63	526
circRNA453	oar-miR-329b-5p	1	13	374
circRNA354	oar-miR-106a	1	142	3660
circRNA490	oar-miR-541-3p	1	240	5160
circRNA262	oar-miR-655-5p	1	24	975
circRNA99	oar-miR-485-5p	1	17	1090
ciRNA30	oar-miR-412-5p	1	2	84.4
circRNA400	oar-miR-3956-5p	1	28	922
circRNA613	oar-miR-29b	1	21	735
circRNA207	oar-let-7b	1	45	1600
ciRNA51	oar-miR-125b	1	10	635
ciRNA35	oar-miR-665-5p	1	4	157
circRNA169	oar-let-7b	1	24	1170
circRNA743	oar-miR-26b	1	291	8460
circRNA296	oar-miR-1193-5p	1	20	634
circRNA229	oar-miR-1197-5p	1	48	845
circRNA637	oar-miR-329a-5p	1	120	2400
circRNA598	oar-miR-133	1	20	1270
circRNA93	oar-let-7b	1	291	8600
circRNA482	oar-miR-30a-3p	1	275	8290
circRNA295	oar-miR-134-3p	1	485	13200
circRNA447	oar-miR-3958-5p	1	48	1760
circRNA21	oar-miR-411b-3p	1	12	695
ciRNA67	oar-miR-665-5p	1	6	537
circRNA151	oar-miR-154a-5p	1	199	11100
circRNA207	oar-miR-493-3p	1	45	1600
circRNA310	oar-miR-362	1	150	3620
circRNA563	oar-miR-654-5p	1	106	2740
circRNA223	oar-miR-433-5p	1	34	1820
circRNA18	oar-miR-432	1	26	1100
circRNA374	oar-miR-487a-5p	1	33	1050
circRNA737	oar-miR-26b	1	37	1410
circRNA473	oar-miR-3956-3p	1	8	499
circRNA382	oar-miR-376e-5p	1	26	984
circRNA776	oar-miR-1185-3p	1	27	331
circRNA44	oar-miR-376a-5p	1	61	1930

circRNA166	oar-miR-323a-5p	1	171	4920
circRNA592	oar-miR-362	1	68	1670
circRNA156	oar-miR-495-5p	1	42	2890
circRNA504	oar-let-7a	1	123	5780
ciRNA2	oar-miR-412-5p	1	6	267
circRNA101	oar-miR-150	1	4	119
circRNA663	oar-miR-493-3p	1	29	1380
circRNA196	oar-miR-411b-5p	1	15	1040
circRNA313	oar-miR-487b-3p	1	235	11700
ciRNA55	oar-miR-654-5p	1	14	1160
ciRNA15	oar-miR-370-3p	1	23.5	1170
circRNA1	oar-miR-541-3p	1	10	605
circRNA116	oar-miR-539-3p	1	342	14200
circRNA230	oar-miR-495-5p	1	12	602
circRNA177	oar-miR-411b-5p	1	17	806
circRNA550	oar-miR-380-3p	1	59	1040
circRNA728	oar-miR-376c-3p	1	3	182
circRNA757	oar-miR-494-3p	1	190	5460
circRNA204	oar-miR-370-5p	1	26	1840
circRNA135	oar-miR-493-3p	1	68	3890
circRNA498	oar-let-7d	1	15	657
circRNA162	oar-miR-544-3p	1	8	320
circRNA671	oar-miR-494-3p	1	174	10400
circRNA119	oar-let-7f	1	3	171
circRNA17	oar-miR-299-5p	1	62	2020
circRNA525	oar-miR-1193-5p	1	62	2050
circRNA35	oar-miR-3955-3p	1	34	1030
circRNA103	oar-miR-136	1	147	2290
circRNA599	oar-miR-1193-5p	1	6	298
circRNA432	oar-miR-411a-3p	1	268	1640
circRNA32	oar-miR-194	1	28	1400
circRNA78	oar-miR-299-5p	1	47	1420
circRNA236	oar-miR-19b	1	181	4960
circRNA299	oar-miR-377-5p	1	8	590
circRNA337	oar-miR-370-5p	1	529	30300
circRNA502	oar-miR-3957-5p	1	17	975
circRNA707	oar-miR-299-3p	1	71	2950
circRNA692	oar-miR-494-3p	1	24	831
circRNA584	oar-miR-29b	1	318	12200
circRNA755	oar-miR-369-5p	1	107	3010
circRNA410	oar-miR-3957-5p	1	160	4560
circRNA708	oar-miR-107	1	141	7610
circRNA766	oar-miR-200a	1	86.2	2720
circRNA680	oar-miR-539-3p	1	14	820
circRNA776	oar-miR-3958-5p	1	27	331
circRNA776	oar-miR-370-3p	1	27	331
circRNA322	oar-miR-16b	1	130	4390
circRNA599	oar-miR-1193-3p	1	6	298
circRNA14	oar-miR-496-5p	1	99	3350
circRNA577	oar-miR-382-3p	1	60	3480
circRNA555	oar-miR-299-3p	1	36	1680
circRNA217	oar-miR-3955-5p	1	93	4010
circRNA556	oar-miR-329a-5p	1	51	3800
circRNA539	oar-miR-1197-5p	1	29	1360
circRNA406	oar-miR-376a-3p	1	148	5360
circRNA743	oar-miR-154b-5p	1	291	8460
ciRNA69	oar-miR-1197-3p	1	5	334
circRNA202	oar-miR-133	1	10	547
circRNA387	oar-miR-22-3p	1	74	3810
circRNA289	oar-miR-376a-5p	1	19	965
circRNA294	oar-miR-432	1	158	8020
circRNA201	oar-let-7c	1	11	812
circRNA584	oar-miR-487b-5p	1	318	12200
circRNA453	oar-miR-1185-3p	1	13	374
circRNA776	oar-let-7f	1	27	331
circRNA129	oar-miR-136	1	58	2800
circRNA119	oar-let-7a	1	3	171
circRNA611	oar-miR-758-5p	1	40	1830
circRNA183	oar-miR-152	1	189	9040
circRNA634	oar-let-7d	1	20	1240
circRNA92	oar-let-7a	1	35	2090
circRNA181	oar-miR-758-5p	1	28	916
circRNA353	oar-miR-410-5p	1	21	1130
circRNA233	oar-miR-543-5p	1	90	5650
circRNA750	oar-miR-432	1	11	808
circRNA235	oar-miR-539-5p	1	12	1030
circRNA548	oar-miR-221	1	13	964
circRNA487	oar-miR-154b-5p	1	33	1440
circRNA783	oar-miR-485-3p	1	339	16100
circRNA646	oar-miR-27a	1	19	876
circRNA229	oar-miR-410-5p	1	48	845
circRNA742	oar-miR-299-3p	1	63	2210
circRNA744	oar-let-7c	1	56	1790
ciRNA1	oar-miR-544-3p	1	4	354
circRNA23	oar-miR-26a	1	70	5360
circRNA524	oar-miR-495-5p	1	16.5	1190
circRNA496	oar-let-7f	1	90.5	3120
circRNA39	oar-miR-376d	1	11	513
circRNA278	oar-miR-30b	1	65	2960
circRNA273	oar-miR-665-5p	1	59	1890
circRNA54	oar-miR-154b-5p	1	52	1810
circRNA565	oar-let-7d	1	45	1450
circRNA586	oar-miR-323a-5p	1	12	765
circRNA103	oar-miR-26b	1	147	2290
circRNA35	oar-miR-376e-5p	1	34	1030
circRNA2	oar-miR-380-3p	1	128	3720
circRNA62	oar-let-7i	1	146	7900
circRNA486	oar-miR-134-3p	1	13	615

circRNA480	oar-miR-412-5p	1	9	338
circRNA712	oar-miR-329a-3p	1	120	5180
circRNA603	oar-miR-433-5p	1	13	873
circRNA737	oar-miR-27a	1	37	1410
circRNA13	oar-miR-19b	1	279	13500
circRNA31	oar-miR-381-5p	1	102	1620
circRNA93	oar-miR-376c-3p	1	291	8600
circRNA343	oar-miR-377-5p	1	31	1690
circRNA775	oar-miR-376e-3p	1	17	410
circRNA491	oar-miR-544-3p	1	34	1470
circRNA444	oar-miR-26b	1	29	1310
circRNA186	oar-miR-329b-5p	1	69	4270
ciRNA68	oar-miR-665-5p	1	5	376
circRNA28	oar-miR-329a-5p	1	23	872
circRNA631	oar-miR-150	1	379	9240
circRNA312	oar-miR-99a	1	190	3850
circRNA565	oar-miR-3957-3p	1	45	1450
circRNA111	oar-miR-494-5p	1	181	4380
circRNA253	oar-miR-133	1	50	1750
circRNA116	oar-miR-485-3p	1	342	14200
circRNA376	oar-miR-3958-5p	1	43	2030
circRNA384	oar-miR-1193-3p	1	78	3290
circRNA785	oar-miR-154a-5p	1	2	135
circRNA569	oar-miR-409-3p	1	175	6360
circRNA479	oar-miR-194	1	94	4200
circRNA231	oar-miR-410-5p	1	59	1860
circRNA495	oar-miR-376a-3p	1	134	5930
circRNA573	oar-let-7c	1	397	22100
circRNA221	oar-miR-433-3p	1	74	2240
circRNA544	oar-miR-376d	1	58.5	2700
circRNA166	oar-miR-654-5p	1	171	4920
circRNA707	oar-miR-194	1	71	2950
ciRNA57	oar-miR-493-5p	1	3	276
circRNA214	oar-miR-299-3p	1	195	10000
circRNA191	oar-miR-370-5p	1	89	4480
circRNA50	oar-let-7b	1	41	968
circRNA362	oar-miR-26a	1	249	13300
circRNA245	oar-miR-3959-5p	1	26	1730
circRNA400	oar-miR-433-5p	1	28	922
circRNA739	oar-miR-412-3p	1	173	6440
circRNA354	oar-miR-494-5p	1	142	3660
circRNA464	oar-miR-323b	1	48	1080
circRNA292	oar-miR-377-5p	1	13	484
circRNA330	oar-miR-410-5p	1	47	2500
ciRNA14	oar-miR-370-5p	1	235	22700
circRNA260	oar-miR-411b-5p	1	42	1370
circRNA462	oar-miR-377-5p	1	43	1210
circRNA592	oar-miR-432	1	68	1670
circRNA567	oar-miR-218a	1	64.5	2650
ciRNA97	oar-miR-494-3p	1	1	85.7
ciRNA53	oar-miR-154b-5p	1	6	306
circRNA10	oar-miR-3957-5p	1	10	483
circRNA177	oar-miR-194	1	17	806
circRNA571	oar-miR-377-3p	1	25	1300
circRNA361	oar-let-7b	1	5	304
circRNA393	oar-miR-485-5p	1	9	450
circRNA610	oar-miR-329a-5p	1	83	2110
circRNA305	oar-miR-323a-5p	1	52	2410
circRNA686	oar-miR-380-5p	1	30	923
circRNA481	oar-let-7d	1	44	2590
circRNA285	oar-miR-485-5p	1	23	991
circRNA438	oar-miR-362	1	33	1310
circRNA398	oar-miR-758-3p	1	135	4930
ciRNA5	oar-miR-494-3p	1	6	305
circRNA443	oar-miR-329b-5p	1	143	7000
ciRNA37	oar-miR-544-3p	1	22	666
circRNA332	oar-miR-30a-3p	1	101	3410
circRNA647	oar-let-7f	1	110	5290
circRNA397	oar-miR-432	1	54	2570
ciRNA7	oar-miR-668-5p	1	4	433
circRNA658	oar-miR-379-5p	1	38	2500
ciRNA61	oar-miR-199a-3p	1	3	261
circRNA8	oar-miR-494-5p	1	85	3450
circRNA378	oar-miR-494-3p	1	42.5	1830
circRNA121	oar-miR-27a	1	633	14600
circRNA135	oar-miR-377-5p	1	68	3890
circRNA200	oar-miR-27a	1	73	2220
circRNA382	oar-miR-329a-5p	1	26	984
circRNA154	oar-miR-329a-5p	1	27	1160
circRNA60	oar-miR-10a	1	66	3680
circRNA181	oar-miR-3956-3p	1	28	916
circRNA352	oar-miR-152	1	45	2060
circRNA57	oar-miR-16b	1	138	7370
circRNA77	oar-miR-369-5p	1	418	15500
circRNA662	oar-miR-376a-3p	1	63	1980
circRNA687	oar-let-7c	1	25	925
circRNA537	oar-miR-103	1	37	2640
circRNA458	oar-miR-370-5p	1	9	549
circRNA413	oar-miR-410-3p	1	4	352
circRNA34	oar-miR-410-5p	1	21	827
circRNA290	oar-miR-323a-5p	1	75	4460
circRNA738	oar-miR-199a-3p	1	211	12700
circRNA379	oar-miR-134-3p	1	155	6470
ciRNA28	oar-miR-154b-5p	1	7	445
circRNA446	oar-miR-323a-5p	1	38	1460
circRNA478	oar-miR-541-3p	1	167	6990
circRNA542	oar-miR-411a-5p	1	16	715
circRNA285	oar-miR-377-5p	1	23	991

ciRNA20	oar-miR-134-5p	1	11	319
circRNA493	oar-miR-377-5p	1	20.5	1330
circRNA354	oar-miR-134-3p	1	142	3660
circRNA771	oar-miR-16b	1	30	1530
circRNA377	oar-miR-191	1	124	3890
circRNA552	oar-miR-1197-3p	1	40	2810
circRNA687	oar-miR-3955-5p	1	25	925
circRNA192	oar-miR-381-3p	1	104	3910
circRNA648	oar-miR-362	1	39.5	1810
circRNA544	oar-miR-16b	1	58.5	2700
circRNA1	oar-miR-654-5p	1	10	605
circRNA398	oar-miR-411b-5p	1	135	4930
circRNA50	oar-miR-485-3p	1	41	968
circRNA139	oar-miR-758-5p	1	6	654
circRNA324	oar-miR-487a-5p	1	59	1900
circRNA697	oar-miR-299-3p	1	21	1080
circRNA401	oar-miR-29a	1	13	666
circRNA52	oar-miR-409-3p	1	14	313
circRNA325	oar-miR-3957-5p	1	39.5	1570
circRNA388	oar-miR-218a	1	21	1740
circRNA609	oar-miR-496-5p	1	49	1860
circRNA545	oar-let-7i	1	174	10400
circRNA225	oar-let-7b	1	29	1590
circRNA334	oar-miR-329a-5p	1	176	11100
circRNA741	oar-miR-495-5p	1	89	2700
circRNA115	oar-miR-433-5p	1	280	9430
circRNA291	oar-miR-3958-5p	1	23	830
circRNA487	oar-miR-495-5p	1	33	1440
circRNA182	oar-miR-543-5p	1	192	6140
circRNA496	oar-miR-329a-5p	1	90.5	3120
circRNA399	oar-miR-30a-3p	1	40	1760
circRNA229	oar-miR-541-3p	1	48	845
circRNA222	oar-miR-370-3p	1	58	2470
circRNA401	oar-miR-495-3p	1	13	666
circRNA124	oar-miR-134-3p	1	157	5240
circRNA146	oar-miR-299-3p	1	175	7890
circRNA304	oar-miR-380-3p	1	262	7540
circRNA512	oar-miR-200c	1	24	1520
ciRNA36	oar-let-7g	1	17	1010
circRNA279	oar-let-7d	1	71	3110
circRNA232	oar-miR-485-5p	1	13	673
circRNA738	oar-miR-370-3p	1	211	12700
circRNA354	oar-miR-539-3p	1	142	3660
circRNA711	oar-miR-665-3p	1	63	2820
circRNA188	oar-miR-433-3p	1	35	1300
circRNA218	oar-miR-99a	1	71	4670
circRNA103	oar-miR-665-5p	1	147	2290
circRNA16	oar-miR-665-3p	1	26	1330
circRNA753	oar-miR-200b	1	109	3710
circRNA42	oar-miR-221	1	193	3260
circRNA585	oar-let-7a	1	24	1130
circRNA728	oar-miR-329b-5p	1	3	182
circRNA647	oar-let-7d	1	110	5290
circRNA577	oar-miR-487a-3p	1	60	3480
circRNA635	oar-miR-485-5p	1	59	2650
circRNA467	oar-miR-654-3p	1	28	1050
circRNA698	oar-miR-665-5p	1	47	2950
circRNA393	oar-miR-134-3p	1	9	450
circRNA265	oar-miR-30d	1	15	215
circRNA625	oar-miR-154b-5p	1	56	1680
circRNA299	oar-miR-654-5p	1	8	590
circRNA603	oar-miR-21	1	13	873
circRNA372	oar-let-7a	1	30	1200
circRNA54	oar-miR-323a-5p	1	52	1810
circRNA772	oar-let-7b	1	34	1290
circRNA746	oar-miR-199a-3p	1	108	3350
circRNA427	oar-let-7i	1	19	551
circRNA61	oar-miR-665-3p	1	17	681
circRNA654	oar-miR-433-3p	1	20	1490
circRNA231	oar-miR-107	1	59	1860
circRNA786	oar-miR-329b-5p	1	26	1520
circRNA656	oar-miR-3959-3p	1	30	1290
circRNA583	oar-miR-3958-5p	1	44	2100
circRNA542	oar-miR-3957-5p	1	16	715
circRNA15	oar-miR-411b-5p	1	21	1030
circRNA612	oar-miR-654-5p	1	9	348
circRNA407	oar-miR-150	1	44	2870
circRNA741	oar-miR-1197-3p	1	89	2700
circRNA254	oar-miR-377-3p	1	14	944
circRNA588	oar-miR-376b-5p	1	20	1250
circRNA482	oar-miR-3956-5p	1	275	8290
circRNA600	oar-miR-411b-3p	1	34	1850
circRNA50	oar-miR-432	1	41	968
circRNA627	oar-miR-412-3p	1	6	472
circRNA683	oar-let-7i	1	7	686
circRNA7	oar-miR-377-3p	1	47	1830
circRNA706	oar-miR-665-3p	1	40	938
circRNA550	oar-miR-191	1	59	1040
circRNA189	oar-miR-103	1	3	343
circRNA731	oar-miR-103	1	15	407
circRNA759	oar-miR-487b-3p	1	46	2440
circRNA707	oar-miR-654-5p	1	71	2950
circRNA255	oar-miR-487a-5p	1	63	4490
circRNA254	oar-miR-544-3p	1	14	944
circRNA22	oar-let-7b	1	255	5330
circRNA766	oar-miR-10a	1	86.2	2720
ciRNA49	oar-miR-410-3p	1	6	449
circRNA427	oar-miR-410-5p	1	19	551

circRNA632	oar-miR-154b-5p	1	26	1610
circRNA103	oar-miR-329b-5p	1	147	2290
circRNA326	oar-miR-369-5p	1	7.5	387
circRNA235	oar-miR-3957-5p	1	12	1030
circRNA149	oar-miR-329a-3p	1	71	3740
circRNA750	oar-miR-379-5p	1	11	808
circRNA59	oar-miR-543-3p	1	73	3560
circRNA342	oar-miR-665-3p	1	45	2070
circRNA652	oar-miR-376b-5p	1	13	706
circRNA443	oar-miR-370-5p	1	143	7000
circRNA243	oar-miR-103	1	26	1480
circRNA349	oar-miR-133	1	11	694
circRNA118	oar-miR-323a-5p	1	32	1790
circRNA309	oar-miR-411a-3p	1	4	249
circRNA415	oar-miR-25	1	49	1620
circRNA664	oar-miR-3959-3p	1	56	2640
circRNA622	oar-miR-218a	1	93	5000
ciRNA70	oar-miR-496-5p	1	7	565
circRNA165	oar-miR-362	1	14	948
circRNA22	oar-miR-541-5p	1	255	5330
ciRNA27	oar-miR-654-3p	1	25	2040
circRNA10	oar-miR-370-3p	1	10	483
circRNA571	oar-miR-432	1	25	1300
circRNA607	oar-miR-1197-3p	1	10	722
ciRNA20	oar-miR-487a-5p	1	11	319
circRNA493	oar-miR-221	1	20.5	1330
circRNA359	oar-miR-3955-5p	1	93	3890
circRNA604	oar-miR-493-3p	1	70	3730
circRNA42	oar-miR-299-5p	1	193	3260
circRNA425	oar-let-7c	1	36	1090
circRNA312	oar-miR-154b-5p	1	190	3850
circRNA651	oar-miR-3955-5p	1	78	1620
circRNA246	oar-miR-10b	1	53	1940
circRNA773	oar-let-7a	1	25	1110
circRNA758	oar-miR-411b-5p	1	69	2410
circRNA138	oar-miR-125b	1	10	640
circRNA5	oar-miR-654-5p	1	23	942
circRNA630	oar-miR-3958-5p	1	35	2060
circRNA2	oar-miR-191	1	128	3720
circRNA296	oar-miR-143	1	20	634
circRNA76	oar-miR-370-3p	1	20	857
circRNA416	oar-miR-758-5p	1	28	1680
ciRNA20	oar-miR-323a-5p	1	11	319
circRNA8	oar-miR-154b-5p	1	85	3450
circRNA534	oar-miR-431	1	18	721
circRNA758	oar-miR-369-5p	1	69	2410
circRNA19	oar-miR-3959-5p	1	22	1010
circRNA528	oar-miR-370-3p	1	93	2490
circRNA614	oar-miR-103	1	50	1590
circRNA583	oar-miR-377-5p	1	44	2100
circRNA165	oar-miR-409-3p	1	14	948
circRNA502	oar-miR-329a-3p	1	17	975
circRNA326	oar-miR-487a-5p	1	7.5	387
circRNA145	oar-miR-432	1	79	3280
circRNA328	oar-miR-3957-5p	1	16	1060
circRNA314	oar-miR-543-3p	1	23	1150
circRNA399	oar-miR-493-3p	1	40	1760
circRNA378	oar-miR-191	1	42.5	1830
circRNA509	oar-miR-377-5p	1	88	3130
circRNA238	oar-miR-1193-5p	1	93	4020
circRNA179	oar-miR-541-3p	1	46	1780
circRNA410	oar-miR-329a-3p	1	160	4560
circRNA361	oar-let-7f	1	5	304
circRNA111	oar-miR-329a-5p	1	181	4380
circRNA535	oar-miR-431	1	17	769
circRNA446	oar-miR-493-3p	1	38	1460
circRNA205	oar-let-7g	1	34	1150
circRNA612	oar-miR-544-3p	1	9	348
circRNA84	oar-miR-411b-5p	1	5	316
circRNA653	oar-miR-539-3p	1	16	332
circRNA231	oar-miR-103	1	59	1860
circRNA551	oar-miR-655-5p	1	193	4060
circRNA415	oar-miR-10b	1	49	1620
circRNA361	oar-let-7a	1	5	304
circRNA613	oar-miR-154b-5p	1	21	735
circRNA51	oar-miR-411b-5p	1	20	664
ciRNA67	oar-miR-381-5p	1	6	537
circRNA555	oar-miR-380-3p	1	36	1680
circRNA352	oar-miR-381-3p	1	45	2060
circRNA431	oar-miR-125b	1	512	2590
circRNA503	oar-miR-654-5p	1	28	1410
circRNA147	oar-miR-495-5p	1	12	556
circRNA80	oar-miR-329b-5p	1	46	1940
circRNA476	oar-miR-134-5p	1	81	2660
circRNA593	oar-miR-29b	1	37	1850
circRNA173	oar-miR-654-3p	1	38	1350
circRNA293	oar-miR-495-5p	1	9	553
circRNA599	oar-miR-758-5p	1	6	298
ciRNA95	oar-miR-379-5p	1	4	383
circRNA252	oar-miR-27a	1	612	11700
circRNA224	oar-miR-3955-5p	1	37	1600
circRNA304	oar-miR-432	1	262	7540
circRNA197	oar-miR-329b-5p	1	37	2200
circRNA100	oar-miR-493-5p	1	20	767
ciRNA2	oar-miR-495-3p	1	6	267
ciRNA19	oar-miR-494-3p	1	3	236
circRNA438	oar-miR-410-5p	1	33	1310
circRNA534	oar-miR-22-3p	1	18	721

circRNA236	oar-miR-543-5p	1	181	4960
circRNA589	oar-miR-409-5p	1	11	260
circRNA639	oar-miR-411a-3p	1	71	3330
circRNA278	oar-miR-370-5p	1	65	2960
circRNA502	oar-miR-655-5p	1	17	975
circRNA59	oar-miR-758-3p	1	73	3560
circRNA447	oar-miR-22-3p	1	48	1760
circRNA605	oar-miR-431	1	63	526
circRNA687	oar-miR-30a-3p	1	25	925
circRNA219	oar-miR-23a	1	8	439
circRNA753	oar-miR-485-5p	1	109	3710
circRNA340	oar-miR-493-5p	1	22	1500
circRNA40	oar-miR-412-3p	1	22	1130
circRNA65	oar-miR-376a-5p	1	98	6480
circRNA50	oar-let-7a	1	41	968
circRNA633	oar-miR-143	1	28	1440
circRNA7	oar-let-7i	1	47	1830
circRNA299	oar-miR-134-5p	1	8	590
circRNA275	oar-miR-29b	1	165	2850
circRNA7	oar-miR-485-3p	1	47	1830
circRNA744	oar-miR-655-5p	1	56	1790
circRNA323	oar-let-7b	1	8	555
circRNA95	oar-miR-376a-3p	1	28	1150
circRNA116	oar-miR-412-3p	1	342	14200
circRNA414	oar-miR-323b	1	10	461
circRNA427	oar-let-7g	1	19	551
circRNA144	oar-miR-544-5p	1	16	798
circRNA356	oar-miR-411b-5p	1	152	6210
circRNA677	oar-miR-544-3p	1	238	8090
ciRNA2	oar-miR-362	1	6	267
circRNA93	oar-let-7d	1	291	8600
ciRNA44	oar-miR-299-3p	1	4	227
circRNA245	oar-miR-432	1	26	1730
circRNA224	oar-miR-541-3p	1	37	1600
circRNA31	oar-miR-758-5p	1	102	1620
circRNA446	oar-miR-411b-5p	1	38	1460
circRNA269	oar-miR-323a-5p	1	37	2060
ciRNA87	oar-miR-329b-3p	1	35.3	2480
circRNA163	oar-miR-154b-3p	1	28	1180
circRNA648	oar-let-7c	1	39.5	1810
circRNA582	oar-miR-376b-5p	1	39	1480
circRNA366	oar-miR-27a	1	6.5	463
circRNA342	oar-miR-409-5p	1	45	2070
circRNA114	oar-miR-103	1	350	14700
circRNA616	oar-miR-329b-5p	1	84	5180
circRNA485	oar-miR-655-5p	1	333	27400
circRNA26	oar-miR-30a-3p	1	3	157
circRNA765	oar-let-7d	1	15	760
circRNA264	oar-miR-3958-5p	1	88	5460
circRNA168	oar-miR-3956-3p	1	45	1730
circRNA253	oar-miR-412-3p	1	50	1750
circRNA436	oar-miR-432	1	123	2820
circRNA265	oar-miR-154b-5p	1	15	215
circRNA458	oar-miR-654-5p	1	9	549
circRNA553	oar-miR-665-5p	1	37	974
circRNA414	oar-miR-411a-3p	1	10	461
circRNA235	oar-miR-376e-3p	1	12	1030
circRNA202	oar-miR-3958-3p	1	10	547
circRNA369	oar-miR-377-5p	1	33	1560
circRNA151	oar-miR-382-5p	1	199	11100
circRNA101	oar-miR-329a-3p	1	4	119
circRNA612	oar-miR-154b-5p	1	9	348
circRNA714	oar-miR-377-5p	1	23	1190
circRNA50	oar-miR-487a-3p	1	41	968
circRNA124	oar-miR-3958-5p	1	157	5240
circRNA323	oar-let-7i	1	8	555
circRNA307	oar-miR-299-5p	1	341	21400
circRNA453	oar-miR-136	1	13	374
ciRNA55	oar-miR-376a-5p	1	14	1160
circRNA89	oar-miR-329b-5p	1	24	2180
circRNA647	oar-miR-665-3p	1	110	5290
circRNA138	oar-miR-654-5p	1	10	640
circRNA570	oar-miR-412-5p	1	121	6150
circRNA686	oar-miR-376e-3p	1	30	923
circRNA556	oar-miR-329b-5p	1	51	3800
circRNA581	oar-miR-150	1	14	835
circRNA550	oar-let-7d	1	59	1040
circRNA763	oar-miR-654-5p	1	337	17400
ciRNA88	oar-miR-134-3p	1	8	668
circRNA92	oar-miR-200a	1	35	2090
circRNA32	oar-miR-485-5p	1	28	1400
circRNA8	oar-let-7c	1	85	3450
circRNA482	oar-let-7f	1	275	8290
circRNA579	oar-miR-107	1	201	6420
circRNA464	oar-miR-377-5p	1	48	1080
circRNA39	oar-miR-376b-3p	1	11	513
circRNA558	oar-miR-26a	1	77	4250
circRNA360	oar-miR-379-5p	1	14	1030
circRNA229	oar-miR-154b-5p	1	48	845
circRNA556	oar-miR-665-5p	1	51	3800
circRNA628	oar-miR-329b-5p	1	58	2150
circRNA154	oar-let-7d	1	27	1160
circRNA754	oar-miR-3958-5p	1	32	2040
circRNA166	oar-miR-133	1	171	4920
circRNA707	oar-miR-154b-5p	1	71	2950
circRNA31	oar-miR-26b	1	102	1620
circRNA172	oar-miR-22-3p	1	21	847
circRNA465	oar-let-7i	1	694	36800

circRNA99	oar-miR-376d	1	17	1090
circRNA579	oar-miR-16b	1	201	6420
circRNA551	oar-miR-154b-5p	1	193	4060
circRNA94	oar-miR-412-5p	1	89	6150
circRNA247	oar-miR-3958-5p	1	41	1110
circRNA498	oar-miR-200a	1	15	657
circRNA697	oar-miR-191	1	21	1080
circRNA399	oar-miR-103	1	40	1760
circRNA308	oar-miR-758-5p	1	2	146
ciRNA48	oar-miR-370-3p	1	10	329
circRNA576	oar-miR-496-5p	1	21	1320
circRNA446	oar-miR-370-3p	1	38	1460
circRNA182	oar-miR-103	1	192	6140
circRNA773	oar-let-7i	1	25	1110
circRNA313	oar-miR-493-3p	1	235	11700
circRNA690	oar-miR-494-5p	1	4	284
circRNA652	oar-miR-376e-5p	1	13	706
circRNA127	oar-miR-412-5p	1	17	1300
circRNA280	oar-miR-1197-5p	1	30	1530
circRNA734	oar-miR-329a-3p	1	37	1920
circRNA57	oar-miR-3957-5p	1	138	7370
circRNA685	oar-miR-376b-3p	1	172	6290
circRNA19	oar-miR-431	1	22	1010
circRNA616	oar-miR-544-3p	1	84	5180
circRNA497	oar-miR-143	1	47.5	1540
circRNA734	oar-miR-431	1	37	1920
circRNA565	oar-let-7i	1	45	1450
circRNA398	oar-miR-26b	1	135	4930
circRNA2	oar-miR-1185-3p	1	128	3720
circRNA254	oar-miR-136	1	14	944
ciRNA24	oar-miR-1193-3p	1	2	218
circRNA302	oar-miR-1197-5p	1	158	3480
circRNA661	oar-miR-381-5p	1	338	17700
circRNA362	oar-miR-3956-5p	1	249	13300
circRNA168	oar-miR-668-3p	1	45	1730
circRNA95	oar-miR-329b-3p	1	28	1150
circRNA286	oar-miR-665-3p	1	50	1870
circRNA575	oar-miR-376a-5p	1	76	3630
circRNA407	oar-miR-154b-5p	1	44	2870
circRNA30	oar-miR-329b-5p	1	62	4220
circRNA613	oar-miR-107	1	21	735
circRNA593	oar-miR-411b-5p	1	37	1850
circRNA612	oar-miR-136	1	9	348
circRNA561	oar-miR-382-5p	1	59	2780
circRNA123	oar-miR-494-5p	1	24.5	1030
circRNA675	oar-miR-133	1	12	667
ciRNA43	oar-miR-1197-5p	1	1	91.4
circRNA464	oar-miR-485-5p	1	48	1080
circRNA781	oar-miR-194	1	8	399
circRNA784	oar-miR-3955-5p	1	1260	41800
circRNA224	oar-miR-181a	1	37	1600
ciRNA45	oar-miR-323a-5p	1	4	312
circRNA703	oar-miR-1185-5p	1	3	248
circRNA136	oar-miR-432	1	24	1000
circRNA615	oar-miR-3958-5p	1	41	2320
circRNA704	oar-miR-541-5p	1	49	2550
circRNA575	oar-miR-3958-3p	1	76	3630
circRNA92	oar-miR-22-3p	1	35	2090
circRNA200	oar-miR-3957-5p	1	73	2220
circRNA479	oar-miR-668-3p	1	94	4200
circRNA50	oar-miR-3958-5p	1	41	968
circRNA240	oar-miR-22-3p	1	114	4230
circRNA550	oar-miR-379-5p	1	59	1040
circRNA304	oar-miR-143	1	262	7540
circRNA435	oar-miR-411a-3p	1	20.2	602
circRNA287	oar-miR-134-5p	1	6	215
circRNA391	oar-miR-154b-5p	1	19	358
circRNA216	oar-miR-487a-5p	1	32	925
circRNA382	oar-let-7a	1	26	984
circRNA455	oar-miR-382-3p	1	20	994
circRNA456	oar-miR-370-5p	1	12	700
circRNA771	oar-miR-200b	1	30	1530
circRNA317	oar-miR-1197-3p	1	223	11200
circRNA573	oar-miR-496-5p	1	397	22100
circRNA281	oar-miR-758-5p	1	17	594
circRNA579	oar-miR-544-3p	1	201	6420
circRNA229	oar-miR-370-5p	1	48	845
circRNA550	oar-let-7g	1	59	1040
circRNA425	oar-miR-376b-5p	1	36	1090
circRNA32	oar-miR-30a-3p	1	28	1400
ciRNA31	oar-miR-485-5p	1	90.5	7640
circRNA775	oar-miR-362	1	17	410
circRNA225	oar-let-7i	1	29	1590
circRNA357	oar-miR-665-3p	1	9	644
circRNA182	oar-miR-380-3p	1	192	6140
circRNA114	oar-miR-29b	1	350	14700
circRNA253	oar-miR-143	1	50	1750
circRNA10	oar-miR-541-5p	1	10	483
circRNA664	oar-miR-103	1	56	2640
circRNA786	oar-miR-377-5p	1	26	1520
circRNA353	oar-miR-3955-5p	1	21	1130
circRNA284	oar-miR-143	1	18	788
circRNA265	oar-miR-23a	1	15	215
circRNA595	oar-miR-17-5p	1	12	672
circRNA613	oar-miR-29a	1	21	735
ciRNA92	oar-let-7f	1	7	562
circRNA358	oar-miR-411b-5p	1	56	3900
ciRNA64	oar-miR-154b-3p	1	7	702

circRNA504	oar-miR-106b	1	123	5780
circRNA686	oar-miR-541-5p	1	30	923
circRNA31	oar-miR-487b-5p	1	102	1620
circRNA100	oar-miR-26a	1	20	767
circRNA89	oar-miR-199a-3p	1	24	2180
ciRNA60	oar-miR-299-3p	1	18	809
circRNA123	oar-miR-409-3p	1	24.5	1030
circRNA440	oar-miR-3958-5p	1	19	1310
circRNA13	oar-miR-665-5p	1	279	13500
circRNA714	oar-miR-409-3p	1	23	1190
circRNA93	oar-let-7a	1	291	8600
ciRNA4	oar-miR-1197-5p	1	2	215
circRNA653	oar-miR-107	1	16	332
circRNA685	oar-miR-1197-5p	1	172	6290
circRNA647	oar-let-7g	1	110	5290
circRNA455	oar-let-7c	1	20	994
circRNA107	oar-miR-136	1	96	4820
ciRNA24	oar-miR-411b-3p	1	2	218
circRNA170	oar-miR-377-3p	1	74	1320
circRNA237	oar-miR-3957-5p	1	20	1330
circRNA50	oar-miR-411b-5p	1	41	968
circRNA360	oar-let-7d	1	14	1030
circRNA465	oar-miR-541-5p	1	694	36800
circRNA277	oar-miR-362	1	971	48700
circRNA175	oar-miR-148a	1	9	475
circRNA16	oar-miR-381-3p	1	26	1330
circRNA495	oar-miR-376b-3p	1	134	5930
circRNA105	oar-miR-496-5p	1	22	896
circRNA166	oar-miR-221	1	171	4920
circRNA599	oar-miR-370-5p	1	6	298
circRNA518	oar-miR-22-3p	1	15	1530
circRNA336	oar-miR-323a-5p	1	24	1370
ciRNA84	oar-miR-487a-5p	1	6	645
circRNA623	oar-miR-485-5p	1	91	4670
circRNA234	oar-miR-3959-3p	1	31	1310
circRNA162	oar-miR-487b-5p	1	8	320
circRNA415	oar-miR-654-5p	1	49	1620
circRNA759	oar-miR-3957-5p	1	46	2440
circRNA373	oar-miR-370-3p	1	44	2350
circRNA205	oar-miR-19b	1	34	1150
circRNA274	oar-miR-758-3p	1	66	2070
circRNA44	oar-let-7i	1	61	1930
circRNA423	oar-miR-412-5p	1	17	1070
circRNA188	oar-miR-22-3p	1	35	1300
circRNA353	oar-miR-494-5p	1	21	1130
circRNA200	oar-miR-22-3p	1	73	2220
circRNA738	oar-miR-25	1	211	12700
circRNA221	oar-miR-376a-3p	1	74	2240
circRNA606	oar-miR-29a	1	95	3890
circRNA634	oar-let-7c	1	20	1240
ciRNA82	oar-miR-21	1	2	137
circRNA290	oar-miR-103	1	75	4460
circRNA111	oar-miR-154b-5p	1	181	4380
circRNA207	oar-let-7a	1	45	1600
circRNA198	oar-miR-544-3p	1	52	2760
ciRNA27	oar-miR-323a-5p	1	25	2040
circRNA248	oar-miR-200a	1	6	370
circRNA242	oar-miR-654-5p	1	56	3340
circRNA111	oar-miR-323a-5p	1	181	4380
circRNA247	oar-miR-150	1	41	1110
circRNA515	oar-miR-329b-5p	1	17	663
circRNA500	oar-miR-376a-5p	1	45	1680
circRNA412	oar-miR-3957-5p	1	28.2	1620
circRNA520	oar-miR-10b	1	8	740
circRNA196	oar-miR-412-3p	1	15	1040
circRNA677	oar-miR-323b	1	238	8090
circRNA349	oar-miR-370-3p	1	11	694
circRNA416	oar-miR-3957-3p	1	28	1680
circRNA726	oar-miR-495-5p	1	64	4520
circRNA175	oar-miR-376d	1	9	475
circRNA345	oar-miR-194	1	32	1330
circRNA770	oar-miR-485-3p	1	7	452
circRNA6	oar-miR-1197-5p	1	67	3600
circRNA521	oar-let-7b	1	49	3400
circRNA771	oar-miR-329a-5p	1	30	1530
circRNA205	oar-let-7c	1	34	1150
circRNA535	oar-miR-369-5p	1	17	769
circRNA671	oar-miR-758-3p	1	174	10400
circRNA775	oar-miR-758-5p	1	17	410
circRNA180	oar-let-7d	1	30	2030
circRNA321	oar-miR-191	1	48	2450
circRNA604	oar-miR-154b-5p	1	70	3730
circRNA748	oar-miR-654-3p	1	22	1180
circRNA724	oar-miR-30a-3p	1	8	540
circRNA124	oar-miR-125b	1	157	5240
circRNA229	oar-miR-1193-5p	1	48	845
circRNA536	oar-miR-16b	1	87	4870
circRNA408	oar-miR-299-5p	1	42	2710
circRNA400	oar-miR-369-5p	1	28	922
circRNA678	oar-miR-143	1	24	1290
circRNA528	oar-miR-1197-5p	1	93	2490
circRNA209	oar-miR-758-5p	1	2	97.7
circRNA739	oar-miR-29a	1	173	6440
circRNA477	oar-miR-376e-3p	1	260	6440
ciRNA75	oar-miR-154a-5p	1	3	118
circRNA516	oar-miR-376b-3p	1	14	976
circRNA116	oar-let-7d	1	342	14200
circRNA114	oar-miR-3957-3p	1	350	14700

circRNA52	oar-miR-329b-3p	1	14	313
circRNA269	oar-let-7a	1	37	2060
circRNA211	oar-miR-329a-5p	1	16	820
circRNA438	oar-miR-221	1	33	1310
circRNA691	oar-miR-377-3p	1	8	474
circRNA199	oar-miR-654-3p	1	14	653
circRNA612	oar-miR-370-3p	1	9	348
circRNA444	oar-miR-3957-5p	1	29	1310
circRNA312	oar-miR-377-3p	1	190	3850
circRNA391	oar-miR-369-5p	1	19	358
circRNA705	oar-miR-409-5p	1	20	657
circRNA421	oar-miR-494-3p	1	20	1070
circRNA331	oar-miR-323a-5p	1	32	2510
ciRNA63	oar-miR-329b-3p	1	14	1230
circRNA499	oar-miR-3959-5p	1	15	581
circRNA128	oar-miR-136	1	118	4950
ciRNA73	oar-miR-127	1	8	675
circRNA154	oar-miR-323a-5p	1	27	1160
circRNA646	oar-let-7g	1	19	876
circRNA777	oar-miR-409-3p	1	10	409
circRNA292	oar-let-7b	1	13	484
circRNA720	oar-let-7i	1	1018	57400
circRNA382	oar-miR-409-3p	1	26	984
circRNA291	oar-miR-654-3p	1	23	830
circRNA303	oar-miR-143	1	39.5	2770
ciRNA74	oar-miR-496-3p	1	5	138
circRNA576	oar-miR-665-3p	1	21	1320
circRNA7	oar-let-7d	1	47	1830
circRNA83	oar-miR-25	1	235	8250
circRNA297	oar-miR-654-5p	1	12	698
circRNA660	oar-miR-411b-5p	1	5	481
ciRNA22	oar-miR-432	1	4	354
circRNA423	oar-miR-323c	1	17	1070
circRNA675	oar-miR-3957-3p	1	12	667
circRNA31	oar-miR-376e-5p	1	102	1620
circRNA356	oar-let-7b	1	152	6210
circRNA651	oar-miR-380-3p	1	78	1620
circRNA314	oar-miR-30a-5p	1	23	1150
circRNA756	oar-miR-29b	1	68	2660
circRNA686	oar-miR-412-5p	1	30	923
circRNA745	oar-miR-665-5p	1	34	2350
circRNA643	oar-miR-218a	1	40	1880
circRNA91	oar-miR-3957-5p	1	38.7	2200
circRNA337	oar-miR-376a-3p	1	529	30300
circRNA611	oar-miR-99a	1	40	1830
ciRNA36	oar-let-7f	1	17	1010
circRNA270	oar-miR-409-5p	1	6	273
circRNA714	oar-miR-485-5p	1	23	1190
circRNA170	oar-miR-380-5p	1	74	1320
circRNA766	oar-miR-10b	1	86.2	2720
circRNA203	oar-miR-329b-5p	1	74	4560
circRNA766	oar-miR-194	1	86.2	2720
circRNA125	oar-let-7d	1	74	3930
circRNA425	oar-let-7a	1	36	1090
circRNA57	oar-miR-143	1	138	7370
ciRNA60	oar-miR-496-5p	1	18	809
circRNA179	oar-miR-370-5p	1	46	1780
circRNA355	oar-miR-154b-5p	1	342	13100
circRNA401	oar-miR-29b	1	13	666
circRNA255	oar-miR-125b	1	63	4490
circRNA275	oar-miR-323c	1	165	2850
circRNA558	oar-miR-134-3p	1	77	4250
circRNA515	oar-miR-376b-3p	1	17	663
circRNA310	oar-miR-665-5p	1	150	3620
circRNA379	oar-miR-3955-5p	1	155	6470
circRNA776	oar-miR-200a	1	27	331
circRNA311	oar-miR-1185-5p	1	32	2310
ciRNA85	oar-miR-329b-3p	1	35.3	2570
circRNA151	oar-miR-23a	1	199	11100
circRNA671	oar-miR-136	1	174	10400
circRNA716	oar-miR-30a-3p	1	83.5	2960
circRNA152	oar-miR-26a	1	42	2660
circRNA683	oar-let-7a	1	7	686
circRNA189	oar-miR-16b	1	3	343
circRNA192	oar-miR-665-5p	1	104	3910
circRNA328	oar-miR-3959-3p	1	16	1060
circRNA541	oar-miR-200b	1	58	4700
circRNA568	oar-miR-136	1	166	4830
circRNA784	oar-miR-1197-5p	1	1260	41800
circRNA638	oar-miR-487b-3p	1	51	2390
circRNA378	oar-miR-134-5p	1	42.5	1830
circRNA678	oar-miR-29b	1	24	1290
circRNA334	oar-miR-154b-5p	1	176	11100
circRNA229	oar-miR-654-5p	1	48	845
circRNA260	oar-miR-412-3p	1	42	1370
circRNA286	oar-miR-541-3p	1	50	1870
circRNA720	oar-miR-665-3p	1	1018	57400
circRNA544	oar-miR-655-3p	1	58.5	2700
circRNA735	oar-miR-107	1	19	950
circRNA590	oar-let-7a	1	81	5580
circRNA155	oar-miR-134-5p	1	21	796
circRNA192	oar-miR-494-5p	1	104	3910
circRNA601	oar-miR-200a	1	349	19400
circRNA401	oar-miR-200c	1	13	666
circRNA328	oar-miR-1193-5p	1	16	1060
circRNA699	oar-miR-323a-5p	1	100	4470
circRNA694	oar-miR-30a-5p	1	109	3640
circRNA341	oar-miR-3956-5p	1	107	4040

circRNA235	oar-miR-376c-3p	1	12	1030
circRNA473	oar-miR-382-5p	1	8	499
circRNA614	oar-miR-29a	1	50	1590
circRNA101	oar-miR-380-5p	1	4	119
circRNA542	oar-miR-665-5p	1	16	715
circRNA586	oar-miR-323a-3p	1	12	765
circRNA647	oar-let-7i	1	110	5290
circRNA739	oar-miR-148a	1	173	6440
circRNA539	oar-miR-23b	1	29	1360
circRNA81	oar-miR-541-5p	1	19	1280
circRNA480	oar-miR-10a	1	9	338
circRNA11	oar-let-7i	1	11	576
circRNA26	oar-miR-106a	1	3	157
circRNA709	oar-miR-10a	1	129	2940
ciRNA8	oar-miR-362	1	2.5	137
circRNA777	oar-miR-494-5p	1	10	409
circRNA139	oar-miR-412-3p	1	6	654
circRNA541	oar-miR-493-3p	1	58	4700
circRNA738	oar-miR-543-5p	1	211	12700
circRNA536	oar-miR-154b-3p	1	87	4870
circRNA254	oar-miR-494-5p	1	14	944
circRNA523	oar-miR-495-5p	1	69.5	3180
circRNA391	oar-let-7d	1	19	358
circRNA592	oar-miR-107	1	68	1670
circRNA744	oar-miR-412-3p	1	56	1790
circRNA329	oar-miR-143	1	61	5460
circRNA94	oar-miR-1197-5p	1	89	6150
circRNA504	oar-let-7c	1	123	5780
circRNA35	oar-miR-329a-3p	1	34	1030
circRNA195	oar-miR-543-5p	1	53	1510
circRNA20	oar-miR-1197-5p	1	86	2300
circRNA498	oar-let-7c	1	15	657
circRNA448	oar-miR-485-5p	1	7	450
circRNA614	oar-miR-668-5p	1	50	1590
ciRNA6	oar-miR-299-5p	1	5	511
circRNA246	oar-miR-133	1	53	1940
circRNA456	oar-miR-3957-5p	1	12	700
circRNA383	oar-let-7i	1	11	479
circRNA33	oar-miR-493-3p	1	34	1890
circRNA508	oar-miR-411a-5p	1	126	10700
ciRNA24	oar-miR-329b-5p	1	2	218
circRNA660	oar-miR-487b-3p	1	5	481
circRNA100	oar-miR-329a-5p	1	20	767
circRNA24	oar-let-7c	1	34	2370
circRNA372	oar-miR-134-3p	1	30	1200
circRNA476	oar-miR-668-5p	1	81	2660
circRNA575	oar-miR-382-3p	1	76	3630
circRNA579	oar-miR-370-3p	1	201	6420
circRNA418	oar-miR-654-3p	1	32	1810
circRNA499	oar-miR-654-5p	1	15	581
circRNA503	oar-miR-665-5p	1	28	1410
circRNA140	oar-miR-382-3p	1	10	719
circRNA52	oar-miR-329a-3p	1	14	313
circRNA346	oar-miR-409-3p	1	15	570
circRNA124	oar-miR-376c-5p	1	157	5240
circRNA105	oar-let-7b	1	22	896
circRNA453	oar-miR-410-3p	1	13	374
ciRNA31	oar-miR-134-3p	1	90.5	7640
circRNA471	oar-miR-487a-5p	1	63	1820
circRNA267	oar-miR-496-3p	1	14	591
circRNA101	oar-miR-376c-3p	1	4	119
circRNA26	oar-miR-380-5p	1	3	157
circRNA574	oar-miR-3958-5p	1	59	4470
circRNA14	oar-miR-487a-3p	1	99	3350
ciRNA4	oar-miR-154b-5p	1	2	215
circRNA225	oar-miR-199a-3p	1	29	1590
circRNA775	oar-let-7d	1	17	410
circRNA391	oar-miR-134-5p	1	19	358
circRNA460	oar-miR-25	1	249	6760
circRNA605	oar-miR-3957-5p	1	63	526
circRNA359	oar-miR-541-5p	1	93	3890
circRNA637	oar-miR-199a-3p	1	120	2400
circRNA620	oar-miR-194	1	75	2640
ciRNA86	oar-miR-329a-3p	1	35.3	2550
ciRNA60	oar-miR-154b-5p	1	18	809
circRNA54	oar-miR-411a-5p	1	52	1810
circRNA169	oar-let-7g	1	24	1170
circRNA322	oar-let-7c	1	130	4390
circRNA8	oar-miR-106b	1	85	3450
circRNA220	oar-miR-496-3p	1	17	894
circRNA775	oar-miR-376c-3p	1	17	410
circRNA166	oar-miR-495-5p	1	171	4920
circRNA413	oar-miR-380-3p	1	4	352
circRNA319	oar-let-7i	1	42	1620
circRNA78	oar-miR-1197-5p	1	47	1420
circRNA350	oar-miR-377-5p	1	9	581
circRNA653	oar-miR-99a	1	16	332
circRNA505	oar-miR-181a	1	6	366
circRNA234	oar-miR-181a	1	31	1310
ciRNA60	oar-miR-665-5p	1	18	809
circRNA561	oar-miR-541-3p	1	59	2780
circRNA145	oar-miR-410-5p	1	79	3280
circRNA98	oar-miR-410-5p	1	13	697
ciRNA83	oar-miR-143	1	8	572
circRNA405	oar-miR-3957-5p	1	53	3730
circRNA38	oar-miR-362	1	117	7230
circRNA257	oar-miR-431	1	118	7890
circRNA655	oar-miR-541-3p	1	10	623

circRNA320	oar-miR-377-5p	1	31	1980
ciRNA52	oar-let-7a	1	8	399
circRNA768	oar-miR-136	1	43.7	1850
circRNA681	oar-miR-665-5p	1	49	1570
circRNA754	oar-miR-654-3p	1	32	2040
circRNA534	oar-miR-370-3p	1	18	721
circRNA168	oar-miR-154a-5p	1	45	1730
circRNA130	oar-miR-485-5p	1	40	2040
circRNA304	oar-miR-544-3p	1	262	7540
circRNA139	oar-miR-380-5p	1	6	654
circRNA36	oar-miR-493-3p	1	100	2650
circRNA195	oar-miR-412-5p	1	53	1510
circRNA664	oar-miR-125b	1	56	2640
circRNA140	oar-miR-370-5p	1	10	719
circRNA90	oar-miR-323a-5p	1	239	12600
circRNA655	oar-miR-154b-5p	1	10	623
circRNA499	oar-miR-410-5p	1	15	581
circRNA265	oar-miR-30c	1	15	215
circRNA168	oar-let-7b	1	45	1730
circRNA358	oar-miR-412-3p	1	56	3900
circRNA285	oar-miR-22-3p	1	23	991
circRNA631	oar-miR-379-5p	1	379	9240
circRNA398	oar-let-7a	1	135	4930
circRNA529	oar-miR-23a	1	50	1940
circRNA425	oar-let-7b	1	36	1090
circRNA701	oar-miR-150	1	4	263
circRNA381	oar-miR-1197-5p	1	29	1720
circRNA68	oar-miR-329a-5p	1	5	305
circRNA756	oar-miR-412-5p	1	68	2660
circRNA605	oar-miR-362	1	63	526
circRNA689	oar-miR-377-5p	1	16	927
circRNA669	oar-miR-380-5p	1	178	6140
circRNA382	oar-miR-665-3p	1	26	984
circRNA466	oar-miR-30a-5p	1	236	14900
circRNA550	oar-miR-3957-5p	1	59	1040
circRNA552	oar-miR-411a-5p	1	40	2810
circRNA222	oar-miR-21	1	58	2470
circRNA397	oar-miR-329a-5p	1	54	2570
circRNA195	oar-miR-107	1	53	1510
circRNA687	oar-let-7f	1	25	925
circRNA406	oar-miR-376d	1	148	5360
circRNA354	oar-miR-3956-3p	1	142	3660
circRNA180	oar-miR-323a-5p	1	30	2030
circRNA410	oar-miR-539-5p	1	160	4560
circRNA195	oar-miR-377-5p	1	53	1510
circRNA422	oar-miR-136	1	28	1210
ciRNA97	oar-miR-411b-3p	1	1	85.7
circRNA29	oar-let-7d	1	53	1680
circRNA493	oar-let-7a	1	20.5	1330
circRNA599	oar-miR-493-3p	1	6	298
circRNA253	oar-miR-25	1	50	1750
circRNA563	oar-miR-154a-5p	1	106	2740
circRNA614	oar-miR-106b	1	50	1590
ciRNA45	oar-miR-485-5p	1	4	312
circRNA177	oar-miR-541-3p	1	17	806
circRNA286	oar-miR-654-3p	1	50	1870
circRNA653	oar-miR-494-5p	1	16	332
circRNA494	oar-miR-127	1	87	3650
circRNA577	oar-miR-1197-3p	1	60	3480
circRNA305	oar-miR-654-5p	1	52	2410
circRNA97	oar-let-7g	1	22	1350
circRNA253	oar-miR-411b-5p	1	50	1750
circRNA260	oar-miR-29a	1	42	1370
ciRNA30	oar-miR-26b	1	2	84.4
circRNA724	oar-miR-329a-5p	1	8	540
circRNA361	oar-let-7i	1	5	304
circRNA744	oar-miR-27a	1	56	1790
ciRNA72	oar-miR-758-3p	1	4	251
circRNA686	oar-miR-107	1	30	923
circRNA712	oar-let-7g	1	120	5180
circRNA634	oar-let-7a	1	20	1240
circRNA776	oar-miR-665-5p	1	27	331
circRNA260	oar-miR-3957-3p	1	42	1370
circRNA456	oar-miR-409-5p	1	12	700
circRNA717	oar-let-7g	1	61.5	3250
circRNA704	oar-miR-432	1	49	2550
circRNA280	oar-miR-25	1	30	1530
circRNA496	oar-let-7c	1	90.5	3120
circRNA608	oar-miR-323a-3p	1	33	1990
circRNA105	oar-miR-362	1	22	896
ciRNA81	oar-miR-1197-5p	1	12	425
circRNA226	oar-miR-410-5p	1	13	1170
circRNA685	oar-miR-376e-3p	1	172	6290
circRNA86	oar-miR-21	1	19	956
circRNA414	oar-miR-494-3p	1	10	461
circRNA744	oar-miR-125b	1	56	1790
circRNA551	oar-miR-411b-5p	1	193	4060
circRNA761	oar-miR-194	1	11	767
circRNA629	oar-miR-26b	1	57	1900
circRNA252	oar-miR-30a-3p	1	612	11700
circRNA728	oar-miR-493-5p	1	3	182
circRNA613	oar-miR-495-3p	1	21	735
circRNA417	oar-miR-370-3p	1	61	2420
circRNA198	oar-miR-10a	1	52	2760
circRNA481	oar-let-7a	1	44	2590
circRNA119	oar-let-7b	1	3	171
circRNA423	oar-miR-200c	1	17	1070
circRNA125	oar-miR-3956-3p	1	74	3930

circRNA662	oar-let-7b	1	63	1980
circRNA533	oar-let-7g	1	53	1980
circRNA93	oar-miR-329a-5p	1	291	8600
circRNA245	oar-miR-10b	1	26	1730
circRNA319	oar-miR-411a-5p	1	42	1620
circRNA137	oar-miR-665-3p	1	24	849
circRNA776	oar-miR-494-5p	1	27	331
circRNA690	oar-miR-127	1	4	284
circRNA413	oar-miR-181a	1	4	352
ciRNA36	oar-let-7d	1	17	1010
circRNA283	oar-miR-10a	1	219	14800
circRNA317	oar-miR-495-5p	1	223	11200
circRNA435	oar-miR-3956-5p	1	20.2	602
circRNA676	oar-miR-493-3p	1	6	537
circRNA296	oar-miR-377-5p	1	20	634
circRNA498	oar-miR-411a-5p	1	15	657
circRNA218	oar-miR-376e-3p	1	71	4670
circRNA544	oar-miR-369-3p	1	58.5	2700
circRNA252	oar-miR-1197-5p	1	612	11700
circRNA415	oar-miR-154b-3p	1	49	1620
ciRNA63	oar-miR-329a-3p	1	14	1230
circRNA744	oar-let-7d	1	56	1790
circRNA66	oar-miR-543-3p	1	39	2330
circRNA519	oar-miR-1193-3p	1	18	1590
circRNA317	oar-miR-27a	1	223	11200
circRNA122	oar-miR-487a-3p	1	177	5560
circRNA381	oar-let-7f	1	29	1720
circRNA148	oar-let-7i	1	213	12600
ciRNA13	oar-miR-370-5p	1	359	32500
circRNA638	oar-miR-200a	1	51	2390
circRNA186	oar-miR-16b	1	69	4270
circRNA495	oar-miR-544-3p	1	134	5930
circRNA223	oar-miR-154b-5p	1	34	1820
circRNA524	oar-miR-655-5p	1	16.5	1190
circRNA312	oar-miR-103	1	190	3850
circRNA776	oar-let-7a	1	27	331
circRNA240	oar-miR-143	1	114	4230
circRNA128	oar-miR-665-5p	1	118	4950
circRNA629	oar-miR-494-3p	1	57	1900
circRNA720	oar-let-7d	1	1018	57400
circRNA246	oar-miR-3956-5p	1	53	1940
circRNA520	oar-miR-485-5p	1	8	740
circRNA642	oar-miR-412-3p	1	22	950
circRNA354	oar-miR-30a-3p	1	142	3660
circRNA75	oar-miR-3955-3p	1	45	1530
circRNA428	oar-miR-3957-5p	1	72.7	4530
circRNA773	oar-let-7d	1	25	1110
circRNA696	oar-miR-411a-5p	1	10	490
circRNA460	oar-miR-134-3p	1	249	6760
circRNA660	oar-miR-194	1	5	481
circRNA724	oar-miR-376e-3p	1	8	540
circRNA151	oar-miR-200c	1	199	11100
circRNA432	oar-miR-323a-3p	1	268	1640
ciRNA49	oar-miR-181a	1	6	449
circRNA277	oar-miR-150	1	971	48700
circRNA128	oar-miR-3955-3p	1	118	4950
circRNA2	oar-miR-485-5p	1	128	3720
circRNA674	oar-miR-148a	1	16	825
circRNA228	oar-miR-541-3p	1	4.5	224
circRNA365	oar-miR-107	1	25	611
circRNA25	oar-miR-382-5p	1	27	1840
circRNA49	oar-miR-26a	1	23	1490
circRNA160	oar-miR-494-3p	1	31	1750
circRNA785	oar-miR-200c	1	2	135
circRNA640	oar-miR-27a	1	59	2230
circRNA73	oar-miR-412-5p	1	11	559
circRNA36	oar-miR-154a-3p	1	100	2650
circRNA44	oar-let-7f	1	61	1930
circRNA39	oar-let-7f	1	11	513
circRNA230	oar-let-7f	1	12	602
circRNA744	oar-miR-382-5p	1	56	1790
circRNA759	oar-miR-432	1	46	2440
circRNA477	oar-miR-370-3p	1	260	6440
circRNA349	oar-miR-3959-5p	1	11	694
circRNA697	oar-miR-410-5p	1	21	1080
circRNA367	oar-miR-432	1	93.5	3400
circRNA216	oar-miR-487b-5p	1	32	925
circRNA568	oar-miR-30a-5p	1	166	4830
circRNA426	oar-miR-495-5p	1	5	383
circRNA772	oar-let-7a	1	34	1290
ciRNA82	oar-miR-200a	1	2	137
circRNA3	oar-miR-432	1	99	4710
ciRNA31	oar-miR-1193-5p	1	90.5	7640
circRNA540	oar-miR-379-5p	1	87	3030
circRNA323	oar-miR-133	1	8	555
circRNA705	oar-miR-134-3p	1	20	657
circRNA114	oar-miR-29a	1	350	14700
circRNA625	oar-miR-107	1	56	1680
circRNA499	oar-miR-1193-5p	1	15	581
circRNA772	oar-miR-299-3p	1	34	1290
circRNA143	oar-miR-665-5p	1	59	4880
circRNA642	oar-miR-134-5p	1	22	950
circRNA196	oar-miR-758-5p	1	15	1040
circRNA421	oar-let-7d	1	20	1070
circRNA569	oar-miR-362	1	175	6360
circRNA290	oar-miR-485-5p	1	75	4460
ciRNA31	oar-miR-323a-5p	1	90.5	7640
circRNA559	oar-miR-329b-5p	1	18	1220

circRNA487	oar-miR-3959-5p	1	33	1440
circRNA430	oar-miR-127	1	319	2550
circRNA229	oar-miR-323a-5p	1	48	845
circRNA490	oar-miR-323a-5p	1	240	5160
circRNA290	oar-miR-16b	1	75	4460
circRNA298	oar-miR-323a-5p	1	28	774
circRNA486	oar-miR-323a-5p	1	13	615
circRNA420	oar-let-7f	1	9	705
circRNA374	oar-miR-3957-3p	1	33	1050
circRNA316	oar-miR-376a-5p	1	19	1020
circRNA275	oar-miR-758-3p	1	165	2850
circRNA466	oar-miR-154b-5p	1	236	14900
circRNA205	oar-miR-133	1	34	1150
circRNA241	oar-let-7a	1	45	2440
circRNA207	oar-miR-125b	1	45	1600
circRNA643	oar-miR-495-5p	1	40	1880
circRNA739	oar-miR-29b	1	173	6440
circRNA610	oar-miR-3958-3p	1	83	2110
circRNA170	oar-miR-432	1	74	1320
circRNA18	oar-miR-30d	1	26	1100
ciRNA20	oar-miR-3957-5p	1	11	319
circRNA251	oar-miR-329b-5p	1	53	1050
circRNA319	oar-miR-412-5p	1	42	1620
circRNA414	oar-miR-200b	1	10	461
circRNA354	oar-miR-544-3p	1	142	3660
circRNA140	oar-miR-136	1	10	719
circRNA712	oar-miR-758-3p	1	120	5180
circRNA625	oar-miR-3959-5p	1	56	1680
circRNA523	oar-miR-148a	1	69.5	3180
circRNA185	oar-miR-10b	1	7	333
ciRNA29	oar-miR-1193-5p	1	1	85.3
ciRNA39	oar-miR-379-5p	1	86	8130
circRNA623	oar-miR-154b-3p	1	91	4670
circRNA32	oar-miR-544-3p	1	28	1400
circRNA195	oar-miR-125b	1	53	1510
circRNA739	oar-miR-543-3p	1	173	6440
circRNA463	oar-miR-370-5p	1	40	2200
circRNA656	oar-miR-1197-5p	1	30	1290
circRNA590	oar-miR-125b	1	81	5580
circRNA262	oar-miR-665-5p	1	24	975
circRNA116	oar-let-7a	1	342	14200
ciRNA21	oar-miR-1193-3p	1	3	147
circRNA752	oar-let-7c	1	21	926
circRNA783	oar-miR-1185-3p	1	339	16100
circRNA475	oar-let-7g	1	16	858
circRNA685	oar-miR-376d	1	172	6290
circRNA341	oar-miR-3959-3p	1	107	4040
circRNA508	oar-miR-665-3p	1	126	10700
circRNA587	oar-miR-1197-5p	1	24	1480
circRNA651	oar-miR-376d	1	78	1620
circRNA252	oar-miR-3958-5p	1	612	11700
circRNA20	oar-miR-191	1	86	2300
circRNA68	oar-miR-181a	1	5	305
circRNA106	oar-miR-541-3p	1	60	2410
circRNA374	oar-miR-3958-5p	1	33	1050
circRNA788	oar-miR-27a	1	400	16300
circRNA219	oar-miR-181a	1	8	439
circRNA369	oar-miR-3958-5p	1	33	1560
circRNA199	oar-miR-29a	1	14	653
circRNA236	oar-miR-1197-5p	1	181	4960
circRNA42	oar-miR-382-5p	1	193	3260
circRNA614	oar-miR-106a	1	50	1590
circRNA136	oar-miR-654-5p	1	24	1000
circRNA458	oar-miR-362	1	9	549
circRNA548	oar-miR-26b	1	13	964
circRNA252	oar-let-7c	1	612	11700
circRNA1	oar-miR-432	1	10	605
circRNA739	oar-miR-544-5p	1	173	6440
circRNA360	oar-miR-143	1	14	1030
circRNA474	oar-miR-410-5p	1	427	25200
circRNA597	oar-miR-379-3p	1	59	2680
circRNA322	oar-miR-541-3p	1	130	4390
circRNA297	oar-miR-143	1	12	698
circRNA230	oar-miR-1193-3p	1	12	602
circRNA276	oar-miR-17-5p	1	8	332
circRNA628	oar-miR-376c-3p	1	58	2150
circRNA354	oar-miR-3955-5p	1	142	3660
circRNA71	oar-miR-362	1	34	1970
circRNA457	oar-miR-362	1	146	7240
circRNA733	oar-miR-382-5p	1	22	1110
circRNA351	oar-miR-323a-3p	1	23	1580
ciRNA60	oar-miR-654-3p	1	18	809
ciRNA48	oar-miR-329b-5p	1	10	329
circRNA360	oar-let-7g	1	14	1030
circRNA35	oar-miR-369-5p	1	34	1030
circRNA176	oar-miR-496-5p	1	24	1150
circRNA425	oar-miR-379-5p	1	36	1090
circRNA239	oar-miR-22-3p	1	3	236
circRNA444	oar-let-7f	1	29	1310
circRNA523	oar-miR-433-3p	1	69.5	3180
circRNA360	oar-let-7i	1	14	1030
circRNA529	oar-miR-134-3p	1	50	1940
circRNA523	oar-miR-494-5p	1	69.5	3180
circRNA744	oar-miR-26a	1	56	1790
circRNA31	oar-miR-376b-5p	1	102	1620
circRNA160	oar-miR-543-3p	1	31	1750
circRNA72	oar-miR-1185-5p	1	35	1280
circRNA249	oar-miR-194	1	357	11500

circRNA501	oar-miR-103	1	218	11300
circRNA592	oar-miR-410-5p	1	68	1670
circRNA587	oar-miR-22-3p	1	24	1480
ciRNA45	oar-let-7c	1	4	312
circRNA251	oar-miR-412-3p	1	53	1050
circRNA758	oar-miR-379-5p	1	69	2410
circRNA202	oar-miR-134-3p	1	10	547
circRNA740	oar-miR-380-5p	1	11	815
circRNA221	oar-let-7i	1	74	2240
circRNA550	oar-miR-433-5p	1	59	1040
circRNA342	oar-miR-30d	1	45	2070
circRNA7	oar-miR-329a-5p	1	47	1830
circRNA659	oar-miR-412-3p	1	125	6640
circRNA359	oar-miR-136	1	93	3890
circRNA547	oar-miR-154a-5p	1	15	1100
circRNA48	oar-miR-152	1	37	992
circRNA103	oar-miR-409-3p	1	147	2290
circRNA549	oar-miR-370-5p	1	26	1570
circRNA596	oar-miR-493-5p	1	39	2490
circRNA207	oar-let-7d	1	45	1600
circRNA327	oar-miR-382-5p	1	27	1070
circRNA280	oar-miR-380-5p	1	30	1530
circRNA434	oar-miR-758-3p	1	167	1280
circRNA491	oar-miR-26a	1	34	1470
circRNA44	oar-miR-103	1	61	1930
circRNA116	oar-let-7i	1	342	14200
circRNA444	oar-miR-409-3p	1	29	1310
circRNA406	oar-miR-412-3p	1	148	5360
circRNA229	oar-miR-665-3p	1	48	845
circRNA337	oar-miR-376d	1	529	30300
circRNA724	oar-miR-376d	1	8	540
circRNA645	oar-miR-758-5p	1	52	2700
circRNA281	oar-miR-496-5p	1	17	594
circRNA422	oar-miR-125b	1	28	1210
circRNA229	oar-miR-433-5p	1	48	845
circRNA272	oar-miR-194	1	67	969
ciRNA19	oar-miR-377-3p	1	3	236
circRNA740	oar-miR-665-5p	1	11	815
circRNA627	oar-miR-411b-5p	1	6	472
circRNA105	oar-let-7g	1	22	896
circRNA31	oar-miR-329b-5p	1	102	1620
circRNA238	oar-miR-362	1	93	4020
circRNA207	oar-miR-495-5p	1	45	1600
circRNA492	oar-miR-133	1	157	7210
circRNA583	oar-miR-544-3p	1	44	2100
circRNA640	oar-miR-376d	1	59	2230
circRNA188	oar-miR-487b-5p	1	35	1300
circRNA180	oar-let-7b	1	30	2030
circRNA566	oar-miR-370-3p	1	65	3570
circRNA780	oar-miR-134-3p	1	2	122
circRNA367	oar-miR-191	1	93.5	3400
circRNA327	oar-miR-3957-5p	1	27	1070
circRNA364	oar-miR-539-3p	1	140	6320
circRNA354	oar-miR-10b	1	142	3660
circRNA742	oar-miR-127	1	63	2210
circRNA274	oar-miR-539-3p	1	66	2070
circRNA167	oar-miR-412-3p	1	14	900
circRNA743	oar-miR-329a-5p	1	291	8460
circRNA492	oar-miR-221	1	157	7210
circRNA249	oar-miR-665-5p	1	357	11500
circRNA459	oar-miR-30d	1	31	1450
circRNA354	oar-miR-17-5p	1	142	3660
ciRNA14	oar-miR-370-3p	1	235	22700
circRNA733	oar-miR-369-3p	1	22	1110
circRNA701	oar-miR-221	1	4	263
circRNA103	oar-miR-29a	1	147	2290
circRNA417	oar-miR-3955-5p	1	61	2420
circRNA118	oar-miR-106b	1	32	1790
circRNA257	oar-miR-485-5p	1	118	7890
circRNA52	oar-miR-668-5p	1	14	313
circRNA621	oar-miR-329a-5p	1	46	3840
circRNA194	oar-miR-665-5p	1	65	3740
circRNA570	oar-miR-30a-5p	1	121	6150
circRNA271	oar-miR-323a-3p	1	27	1890
circRNA629	oar-miR-329a-5p	1	57	1900
ciRNA58	oar-miR-1193-5p	1	2.33	122
circRNA167	oar-miR-381-5p	1	14	900
circRNA657	oar-miR-3957-3p	1	19	843
circRNA537	oar-miR-10b	1	37	2640
circRNA103	oar-let-7b	1	147	2290
circRNA436	oar-miR-329b-5p	1	123	2820
circRNA430	oar-miR-496-5p	1	319	2550
circRNA48	oar-miR-493-3p	1	37	992
circRNA93	oar-miR-99a	1	291	8600
circRNA247	oar-miR-16b	1	41	1110
circRNA287	oar-miR-154b-5p	1	6	215
circRNA464	oar-miR-200b	1	48	1080
circRNA216	oar-miR-3956-5p	1	32	925
circRNA543	oar-miR-382-5p	1	8.5	744
circRNA629	oar-miR-181a	1	57	1900
circRNA704	oar-miR-376a-5p	1	49	2550
circRNA97	oar-let-7d	1	22	1350
circRNA605	oar-miR-3956-3p	1	63	526
circRNA354	oar-miR-3959-3p	1	142	3660
circRNA96	oar-miR-654-5p	1	44	3060
circRNA260	oar-miR-30a-3p	1	42	1370
circRNA689	oar-miR-194	1	16	927
circRNA702	oar-miR-1197-3p	1	20	1460

circRNA543	oar-miR-376c-3p	1	8.5	744
circRNA479	oar-miR-376a-5p	1	94	4200
circRNA251	oar-miR-411b-5p	1	53	1050
circRNA118	oar-miR-3958-5p	1	32	1790
circRNA459	oar-miR-493-3p	1	31	1450
circRNA680	oar-miR-487a-3p	1	14	820
circRNA591	oar-miR-30b	1	21	853
circRNA706	oar-miR-329b-5p	1	40	938
circRNA437	oar-miR-362	1	17	1130
circRNA118	oar-miR-1197-5p	1	32	1790
circRNA717	oar-miR-412-3p	1	61.5	3250
circRNA587	oar-miR-487b-5p	1	24	1480
circRNA328	oar-miR-485-3p	1	16	1060
circRNA305	oar-miR-544-3p	1	52	2410
circRNA191	oar-miR-377-5p	1	89	4480
ciRNA78	oar-miR-665-3p	1	5	538
circRNA462	oar-miR-1197-3p	1	43	1210
circRNA354	oar-miR-410-5p	1	142	3660
circRNA24	oar-miR-3957-5p	1	34	2370
circRNA15	oar-miR-496-5p	1	21	1030
circRNA6	oar-miR-134-3p	1	67	3600
circRNA582	oar-miR-329a-5p	1	39	1480
circRNA202	oar-miR-654-5p	1	10	547
circRNA279	oar-miR-125b	1	71	3110
circRNA221	oar-let-7b	1	74	2240
circRNA712	oar-let-7a	1	120	5180
circRNA51	oar-miR-370-5p	1	20	664
circRNA602	oar-miR-362	1	197	11100
circRNA53	oar-miR-376a-5p	1	61	3980
circRNA339	oar-miR-103	1	9.5	677
circRNA354	oar-miR-541-5p	1	142	3660
circRNA780	oar-miR-194	1	2	122
circRNA93	oar-miR-1197-3p	1	291	8600
circRNA114	oar-miR-758-3p	1	350	14700
circRNA370	oar-let-7c	1	39	1490
circRNA515	oar-miR-544-3p	1	17	663
circRNA36	oar-miR-381-3p	1	100	2650
circRNA67	oar-miR-376d	1	26	1430
circRNA144	oar-miR-218a	1	16	798
circRNA401	oar-miR-377-5p	1	13	666
circRNA251	oar-miR-485-3p	1	53	1050
circRNA150	oar-miR-329b-5p	1	55	4530
ciRNA48	oar-miR-133	1	10	329
circRNA693	oar-miR-758-3p	1	27	842
circRNA97	oar-let-7f	1	22	1350
circRNA582	oar-miR-376e-3p	1	39	1480
circRNA466	oar-miR-493-3p	1	236	14900
circRNA265	oar-miR-495-5p	1	15	215
circRNA771	oar-miR-376e-5p	1	30	1530
circRNA150	oar-miR-29b	1	55	4530
circRNA592	oar-miR-668-3p	1	68	1670
circRNA616	oar-miR-376e-5p	1	84	5180
ciRNA40	oar-miR-3958-5p	1	86	8180
circRNA291	oar-miR-329b-3p	1	23	830
ciRNA49	oar-miR-493-5p	1	6	449
circRNA265	oar-miR-329b-3p	1	15	215
circRNA146	oar-miR-299-5p	1	175	7890
circRNA144	oar-miR-329a-5p	1	16	798
circRNA114	oar-miR-323a-3p	1	350	14700
circRNA678	oar-miR-541-5p	1	24	1290
circRNA67	oar-miR-143	1	26	1430
circRNA28	oar-miR-485-3p	1	23	872
circRNA527	oar-miR-136	1	48	2150
circRNA431	oar-miR-411a-3p	1	512	2590
ciRNA50	oar-miR-10a	1	36	3220
circRNA756	oar-miR-654-5p	1	68	2660
circRNA367	oar-miR-3958-3p	1	93.5	3400
ciRNA85	oar-miR-329a-3p	1	35.3	2570
circRNA274	oar-miR-154b-3p	1	66	2070
circRNA225	oar-let-7g	1	29	1590
circRNA406	oar-miR-3958-5p	1	148	5360
circRNA500	oar-let-7g	1	45	1680
circRNA60	oar-miR-541-3p	1	66	3680
ciRNA56	oar-miR-411b-5p	1	15	1070
circRNA636	oar-miR-3955-3p	1	49	2080
circRNA780	oar-miR-3957-3p	1	2	122
circRNA279	oar-let-7g	1	71	3110
circRNA628	oar-miR-1193-3p	1	58	2150
circRNA266	oar-miR-103	1	95	5370
circRNA230	oar-miR-409-5p	1	12	602
circRNA294	oar-miR-382-5p	1	158	8020
circRNA7	oar-let-7c	1	47	1830
circRNA11	oar-miR-134-5p	1	11	576
circRNA692	oar-miR-665-5p	1	24	831
circRNA92	oar-let-7c	1	35	2090
circRNA500	oar-let-7f	1	45	1680
circRNA346	oar-miR-136	1	15	570
circRNA251	oar-miR-432	1	53	1050
circRNA716	oar-miR-21	1	83.5	2960
circRNA463	oar-miR-329a-5p	1	40	2200
circRNA733	oar-miR-133	1	22	1110
circRNA577	oar-miR-152	1	60	3480
circRNA370	oar-miR-382-5p	1	39	1490
circRNA743	oar-miR-3955-3p	1	291	8460
circRNA146	oar-miR-329b-3p	1	175	7890
circRNA48	oar-miR-411a-5p	1	37	992
circRNA50	oar-miR-299-5p	1	41	968
circRNA136	oar-miR-412-3p	1	24	1000

ciRNA11	oar-miR-494-3p	1	4	270
circRNA571	oar-miR-99a	1	25	1300
circRNA700	oar-miR-134-3p	1	33	2440
ciRNA24	oar-miR-668-3p	1	2	218
circRNA686	oar-miR-194	1	30	923
circRNA597	oar-miR-665-5p	1	59	2680
circRNA756	oar-miR-433-3p	1	68	2660
circRNA18	oar-miR-26b	1	26	1100
circRNA670	oar-miR-3959-5p	1	364	16900
circRNA668	oar-miR-3958-5p	1	30	1850
circRNA299	oar-miR-655-5p	1	8	590
circRNA78	oar-miR-370-3p	1	47	1420
circRNA153	oar-miR-433-3p	1	19	1110
circRNA1	oar-miR-410-5p	1	10	605
circRNA195	oar-miR-154b-5p	1	53	1510
circRNA588	oar-let-7g	1	20	1250
circRNA253	oar-miR-369-5p	1	50	1750
circRNA613	oar-miR-487b-5p	1	21	735
circRNA149	oar-miR-26b	1	71	3740
circRNA565	oar-let-7f	1	45	1450
circRNA305	oar-miR-410-5p	1	52	2410
circRNA689	oar-miR-200a	1	16	927
circRNA414	oar-miR-3957-3p	1	10	461
circRNA497	oar-miR-668-5p	1	47.5	1540
circRNA185	oar-miR-544-3p	1	7	333
circRNA458	oar-miR-493-3p	1	9	549
circRNA398	oar-miR-26a	1	135	4930
circRNA692	oar-miR-125b	1	24	831
circRNA707	oar-miR-323a-3p	1	71	2950
circRNA679	oar-miR-433-3p	1	45	2240
ciRNA49	oar-miR-409-3p	1	6	449
circRNA276	oar-miR-106a	1	8	332
circRNA669	oar-miR-218a	1	178	6140
circRNA641	oar-miR-376a-3p	1	14	853
circRNA701	oar-miR-136	1	4	263
circRNA707	oar-let-7g	1	71	2950
circRNA555	oar-miR-494-5p	1	36	1680
circRNA754	oar-miR-380-3p	1	32	2040
circRNA432	oar-miR-3956-5p	1	268	1640
circRNA443	oar-miR-1193-3p	1	143	7000
circRNA22	oar-miR-3959-5p	1	255	5330
ciRNA92	oar-let-7g	1	7	562
circRNA518	oar-miR-200a	1	15	1530
circRNA492	oar-miR-154b-5p	1	157	7210
circRNA527	oar-miR-379-3p	1	48	2150
circRNA490	oar-miR-409-3p	1	240	5160
circRNA135	oar-miR-544-3p	1	68	3890
circRNA592	oar-miR-411b-3p	1	68	1670
circRNA369	oar-miR-370-3p	1	33	1560
circRNA300	oar-miR-194	1	142	5630
circRNA729	oar-miR-410-5p	1	181	12000
circRNA639	oar-miR-380-3p	1	71	3330
circRNA466	oar-miR-30c	1	236	14900
circRNA93	oar-let-7c	1	291	8600
circRNA116	oar-miR-432	1	342	14200
circRNA37	oar-miR-323a-5p	1	61	2680
circRNA183	oar-miR-541-5p	1	189	9040
circRNA325	oar-miR-487a-5p	1	39.5	1570
circRNA550	oar-let-7b	1	59	1040
circRNA272	oar-miR-485-5p	1	67	969
circRNA563	oar-miR-134-5p	1	106	2740
circRNA513	oar-miR-1193-3p	1	14	1130
circRNA728	oar-miR-3957-5p	1	3	182
circRNA166	oar-miR-3956-3p	1	171	4920
circRNA343	oar-miR-299-3p	1	31	1690
circRNA191	oar-miR-668-3p	1	89	4480
circRNA167	oar-miR-409-5p	1	14	900
circRNA353	oar-miR-154a-3p	1	21	1130
circRNA604	oar-miR-655-5p	1	70	3730
circRNA161	oar-miR-323a-5p	1	1073	56600
circRNA49	oar-miR-152	1	23	1490
circRNA458	oar-miR-541-5p	1	9	549
circRNA158	oar-miR-382-5p	1	26	1560
circRNA536	oar-miR-17-5p	1	87	4870
circRNA251	oar-miR-21	1	53	1050
circRNA732	oar-miR-200a	1	16	538
circRNA23	oar-miR-485-5p	1	70	5360
circRNA381	oar-let-7g	1	29	1720
circRNA599	oar-miR-495-3p	1	6	298
circRNA663	oar-miR-432	1	29	1380
circRNA21	oar-miR-323a-5p	1	12	695
circRNA244	oar-miR-323a-5p	1	71	3830
circRNA765	oar-let-7a	1	15	760
circRNA324	oar-miR-668-3p	1	59	1900
circRNA451	oar-let-7i	1	34	2270
circRNA205	oar-let-7d	1	34	1150
circRNA623	oar-miR-107	1	91	4670
circRNA479	oar-miR-154a-3p	1	94	4200
circRNA778	oar-miR-181a	1	24	1210
circRNA712	oar-miR-200a	1	120	5180
circRNA39	oar-let-7c	1	11	513
circRNA776	oar-miR-493-5p	1	27	331
circRNA466	oar-miR-133	1	236	14900
circRNA668	oar-miR-150	1	30	1850
circRNA24	oar-let-7b	1	34	2370
circRNA705	oar-miR-154a-3p	1	20	657
circRNA522	oar-miR-376a-3p	1	39	1470
circRNA258	oar-miR-10b	1	6	514

ciRNA36	oar-miR-323a-5p	1	17	1010
circRNA168	oar-miR-432	1	45	1730
circRNA490	oar-miR-543-5p	1	240	5160
circRNA671	oar-miR-493-3p	1	174	10400
circRNA327	oar-miR-668-3p	1	27	1070
circRNA504	oar-let-7b	1	123	5780
circRNA428	oar-let-7i	1	72.7	4530
circRNA81	oar-miR-654-5p	1	19	1280
circRNA604	oar-miR-411a-3p	1	70	3730
ciRNA58	oar-miR-433-5p	1	2.33	122
circRNA17	oar-miR-410-5p	1	62	2020
circRNA394	oar-miR-1197-5p	1	23	1490
circRNA206	oar-miR-25	1	20	1050
circRNA119	oar-miR-329b-5p	1	3	171
circRNA648	oar-miR-23a	1	39.5	1810
circRNA168	oar-miR-382-5p	1	45	1730
circRNA715	oar-miR-25	1	64	2040
circRNA66	oar-miR-376a-5p	1	39	2330
circRNA229	oar-miR-541-5p	1	48	845
circRNA200	oar-miR-493-3p	1	73	2220
circRNA37	oar-miR-154b-3p	1	61	2680
circRNA689	oar-miR-221	1	16	927
circRNA286	oar-miR-654-5p	1	50	1870
circRNA207	oar-let-7i	1	45	1600
circRNA322	oar-let-7b	1	130	4390
circRNA12	oar-miR-379-5p	1	32	1620
circRNA291	oar-miR-154a-5p	1	23	830
circRNA122	oar-miR-377-5p	1	177	5560
circRNA767	oar-miR-29b	1	45.2	3460
circRNA596	oar-let-7b	1	39	2490
circRNA778	oar-miR-370-3p	1	24	1210
ciRNA7	oar-miR-150	1	4	433
circRNA245	oar-miR-494-5p	1	26	1730
circRNA441	oar-miR-3955-5p	1	21	626
circRNA229	oar-miR-134-3p	1	48	845
circRNA731	oar-miR-107	1	15	407
circRNA8	oar-miR-487b-3p	1	85	3450
circRNA773	oar-let-7c	1	25	1110
circRNA707	oar-miR-655-3p	1	71	2950
circRNA35	oar-miR-654-5p	1	34	1030
ciRNA38	oar-miR-125b	1	51	3960
circRNA162	oar-miR-665-3p	1	8	320
circRNA703	oar-miR-3955-3p	1	3	248
circRNA370	oar-let-7d	1	39	1490
circRNA31	oar-miR-3957-5p	1	102	1620
circRNA711	oar-miR-30c	1	63	2820
circRNA11	oar-let-7a	1	11	576
circRNA779	oar-miR-329b-5p	1	139	7970
circRNA620	oar-miR-412-5p	1	75	2640
circRNA448	oar-miR-668-3p	1	7	450
circRNA218	oar-miR-376c-3p	1	71	4670
circRNA653	oar-miR-485-3p	1	16	332
circRNA173	oar-miR-134-3p	1	38	1350
circRNA670	oar-miR-323a-5p	1	364	16900
circRNA172	oar-miR-3958-5p	1	21	847
circRNA644	oar-miR-485-5p	1	30	1540
circRNA624	oar-miR-199a-3p	1	6	641
circRNA168	oar-let-7d	1	45	1730
circRNA372	oar-let-7g	1	30	1200
circRNA256	oar-miR-410-5p	1	58	4320
circRNA640	oar-let-7c	1	59	2230
circRNA391	oar-miR-10b	1	19	358
circRNA125	oar-miR-411a-5p	1	74	3930
circRNA742	oar-miR-376e-5p	1	63	2210
circRNA427	oar-miR-544-5p	1	19	551
circRNA284	oar-miR-1197-5p	1	18	788
circRNA648	oar-miR-133	1	39.5	1810
circRNA591	oar-miR-665-5p	1	21	853
circRNA79	oar-miR-3957-5p	1	22	1320
circRNA73	oar-miR-412-3p	1	11	559
circRNA328	oar-miR-496-5p	1	16	1060
circRNA220	oar-miR-3957-5p	1	17	894
circRNA356	oar-miR-379-5p	1	152	6210
circRNA652	oar-miR-329a-5p	1	13	706
circRNA152	oar-miR-26b	1	42	2660
circRNA52	oar-miR-487b-5p	1	14	313
circRNA95	oar-miR-23a	1	28	1150
circRNA9	oar-miR-665-5p	1	27	1220
circRNA647	oar-let-7a	1	110	5290
circRNA739	oar-miR-152	1	173	6440
circRNA641	oar-miR-376b-3p	1	14	853
circRNA22	oar-miR-380-3p	1	255	5330
circRNA178	oar-miR-1185-3p	1	16	1380
circRNA246	oar-miR-668-3p	1	53	1940
circRNA22	oar-miR-136	1	255	5330
ciRNA81	oar-miR-329b-3p	1	12	425
circRNA216	oar-miR-3955-5p	1	32	925
circRNA538	oar-miR-134-5p	1	41	1830
circRNA678	oar-miR-29a	1	24	1290
ciRNA84	oar-miR-541-3p	1	6	645
circRNA537	oar-miR-544-5p	1	37	2640
circRNA163	oar-miR-376c-5p	1	28	1180
circRNA626	oar-miR-200a	1	100	6280
circRNA771	oar-miR-329b-3p	1	30	1530
circRNA683	oar-let-7b	1	7	686
circRNA275	oar-miR-106b	1	165	2850
circRNA122	oar-miR-1185-3p	1	177	5560
circRNA115	oar-miR-329a-5p	1	280	9430

ciRNA84	oar-miR-654-5p	1	6	645
circRNA707	oar-miR-374a	1	71	2950
ciRNA2	oar-miR-376a-5p	1	6	267
circRNA756	oar-miR-29a	1	68	2660
circRNA356	oar-let-7c	1	152	6210
circRNA599	oar-miR-134-3p	1	6	298
circRNA693	oar-miR-544-3p	1	27	842
circRNA362	oar-miR-381-5p	1	249	13300
circRNA101	oar-miR-329b-3p	1	4	119
circRNA50	oar-miR-381-3p	1	41	968
circRNA513	oar-miR-107	1	14	1130
circRNA74	oar-miR-412-3p	1	16	1040
circRNA342	oar-miR-541-3p	1	45	2070
circRNA344	oar-miR-107	1	19	583
circRNA628	oar-miR-154b-5p	1	58	2150
circRNA474	oar-miR-665-5p	1	427	25200
circRNA658	oar-miR-329b-5p	1	38	2500
circRNA752	oar-let-7i	1	21	926
circRNA605	oar-miR-655-3p	1	63	526
circRNA52	oar-miR-544-3p	1	14	313
circRNA761	oar-miR-3955-5p	1	11	767
circRNA551	oar-miR-3956-3p	1	193	4060
circRNA215	oar-miR-103	1	11	948
circRNA464	oar-miR-127	1	48	1080
circRNA114	oar-miR-30c	1	350	14700
circRNA2	oar-miR-665-3p	1	128	3720
circRNA679	oar-miR-29a	1	45	2240
circRNA503	oar-miR-665-3p	1	28	1410
ciRNA48	oar-miR-329a-5p	1	10	329
circRNA678	oar-miR-19b	1	24	1290
circRNA125	oar-let-7c	1	74	3930
circRNA641	oar-miR-21	1	14	853
circRNA40	oar-miR-654-5p	1	22	1130
circRNA204	oar-miR-10b	1	26	1840
circRNA323	oar-miR-541-3p	1	8	555
circRNA184	oar-miR-154b-5p	1	42	2340
circRNA252	oar-miR-143	1	612	11700
circRNA543	oar-miR-376a-3p	1	8.5	744
circRNA12	oar-miR-125b	1	32	1620
circRNA752	oar-miR-199a-3p	1	21	926
circRNA584	oar-miR-218a	1	318	12200
circRNA482	oar-miR-410-5p	1	275	8290
circRNA765	oar-let-7g	1	15	760
circRNA400	oar-miR-543-5p	1	28	922
circRNA121	oar-miR-370-3p	1	633	14600
circRNA705	oar-miR-21	1	20	657
circRNA610	oar-miR-377-5p	1	83	2110
circRNA37	oar-miR-485-3p	1	61	2680
circRNA400	oar-miR-544-5p	1	28	922
circRNA471	oar-miR-431	1	63	1820
circRNA765	oar-miR-379-3p	1	15	760
circRNA728	oar-miR-3956-5p	1	3	182
circRNA191	oar-miR-485-5p	1	89	4480
circRNA304	oar-miR-125b	1	262	7540
circRNA83	oar-miR-380-3p	1	235	8250
circRNA532	oar-miR-541-3p	1	731	18000
circRNA711	oar-miR-496-3p	1	63	2820
circRNA244	oar-miR-495-5p	1	71	3830
circRNA670	oar-miR-433-5p	1	364	16900
circRNA91	oar-miR-323a-3p	1	38.7	2200
circRNA772	oar-miR-654-3p	1	34	1290
circRNA644	oar-miR-493-3p	1	30	1540
circRNA487	oar-miR-382-3p	1	33	1440
circRNA183	oar-miR-379-5p	1	189	9040
circRNA644	oar-miR-134-3p	1	30	1540
circRNA632	oar-miR-665-5p	1	26	1610
circRNA593	oar-miR-29a	1	37	1850
circRNA85	oar-let-7i	1	12	501
circRNA634	oar-let-7i	1	20	1240
circRNA275	oar-miR-1185-5p	1	165	2850
circRNA592	oar-miR-199a-3p	1	68	1670
circRNA764	oar-let-7g	1	159	6640
circRNA590	oar-let-7g	1	81	5580
circRNA481	oar-let-7f	1	44	2590
circRNA238	oar-miR-3957-5p	1	93	4020
circRNA464	oar-miR-543-3p	1	48	1080
circRNA414	oar-miR-379-3p	1	10	461
circRNA119	oar-let-7c	1	3	171
circRNA662	oar-let-7c	1	63	1980
circRNA498	oar-let-7b	1	15	657
circRNA41	oar-miR-29b	1	9	617
circRNA339	oar-miR-136	1	9.5	677
circRNA463	oar-miR-379-5p	1	40	2200
ciRNA77	oar-miR-103	1	1	98
circRNA508	oar-miR-379-5p	1	126	10700
ciRNA36	oar-let-7c	1	17	1010
circRNA752	oar-let-7b	1	21	926
circRNA365	oar-miR-125b	1	25	611
circRNA373	oar-miR-19b	1	44	2350
circRNA7	oar-miR-199a-3p	1	47	1830
circRNA379	oar-miR-323b	1	155	6470
circRNA231	oar-miR-433-5p	1	59	1860
circRNA256	oar-miR-1185-3p	1	58	4320
circRNA637	oar-miR-30a-3p	1	120	2400
circRNA749	oar-miR-1197-3p	1	72	3380
circRNA498	oar-miR-3957-5p	1	15	657
circRNA433	oar-miR-411a-3p	1	213	1440
circRNA116	oar-let-7b	1	342	14200

circRNA223	oar-miR-381-5p	1	34	1820
circRNA575	oar-miR-3956-3p	1	76	3630
circRNA50	oar-miR-377-3p	1	41	968
circRNA29	oar-miR-154b-5p	1	53	1680
circRNA663	oar-miR-543-5p	1	29	1380
circRNA324	oar-miR-369-5p	1	59	1900
circRNA378	oar-miR-1197-3p	1	42.5	1830
circRNA659	oar-miR-411b-5p	1	125	6640
circRNA361	oar-let-7d	1	5	304
circRNA341	oar-miR-654-3p	1	107	4040
circRNA623	oar-miR-487a-3p	1	91	4670
circRNA469	oar-miR-376a-5p	1	46	2790
circRNA9	oar-miR-496-3p	1	27	1220
circRNA785	oar-miR-370-3p	1	2	135
circRNA332	oar-miR-541-5p	1	101	3410
circRNA544	oar-miR-374a	1	58.5	2700
circRNA502	oar-miR-377-5p	1	17	975
circRNA757	oar-miR-379-5p	1	190	5460
circRNA135	oar-miR-410-5p	1	68	3890
circRNA608	oar-miR-412-3p	1	33	1990
circRNA762	oar-miR-329a-5p	1	24.5	1680
circRNA42	oar-miR-362	1	193	3260
circRNA625	oar-miR-376e-5p	1	56	1680
circRNA731	oar-miR-152	1	15	407
circRNA52	oar-miR-376a-5p	1	14	313
circRNA319	oar-miR-370-3p	1	42	1620
circRNA398	oar-let-7i	1	135	4930
circRNA194	oar-miR-376a-5p	1	65	3740
circRNA569	oar-miR-3959-5p	1	175	6360
circRNA434	oar-miR-3956-5p	1	167	1280
circRNA310	oar-miR-3958-5p	1	150	3620
circRNA776	oar-miR-154a-5p	1	27	331
circRNA124	oar-miR-380-3p	1	157	5240
circRNA704	oar-miR-654-5p	1	49	2550
circRNA764	oar-miR-411b-5p	1	159	6640
circRNA495	oar-miR-410-5p	1	134	5930
circRNA765	oar-miR-1197-3p	1	15	760
circRNA750	oar-miR-539-5p	1	11	808
circRNA772	oar-miR-134-3p	1	34	1290
circRNA245	oar-miR-379-5p	1	26	1730
circRNA762	oar-miR-412-5p	1	24.5	1680
circRNA334	oar-miR-134-3p	1	176	11100
circRNA230	oar-let-7c	1	12	602
circRNA660	oar-miR-369-5p	1	5	481
circRNA302	oar-miR-370-5p	1	158	3480
circRNA785	oar-miR-655-5p	1	2	135
circRNA563	oar-miR-431	1	106	2740
circRNA224	oar-miR-544-3p	1	37	1600
circRNA539	oar-miR-30a-5p	1	29	1360
circRNA13	oar-miR-758-5p	1	279	13500
circRNA521	oar-let-7c	1	49	3400
circRNA292	oar-miR-3958-3p	1	13	484
circRNA36	oar-miR-654-3p	1	100	2650
circRNA123	oar-miR-1197-5p	1	24.5	1030
circRNA383	oar-let-7a	1	11	479
circRNA651	oar-miR-323a-5p	1	78	1620
circRNA399	oar-miR-107	1	40	1760
ciRNA53	oar-miR-299-5p	1	6	306
circRNA139	oar-miR-329b-5p	1	6	654
circRNA247	oar-miR-3956-5p	1	41	1110
circRNA766	oar-miR-654-3p	1	86.2	2720
circRNA252	oar-miR-370-5p	1	612	11700
circRNA40	oar-miR-1193-3p	1	22	1130
circRNA468	oar-miR-654-5p	1	31	2170
circRNA371	oar-miR-23a	1	27	1490
circRNA606	oar-miR-107	1	95	3890
circRNA209	oar-miR-3959-5p	1	2	97.7
circRNA657	oar-miR-485-5p	1	19	843
circRNA198	oar-miR-381-5p	1	52	2760
circRNA479	oar-miR-16b	1	94	4200
circRNA354	oar-miR-487a-3p	1	142	3660
circRNA439	oar-miR-544-3p	1	146	3240
circRNA491	oar-miR-323a-5p	1	34	1470
circRNA776	oar-miR-199a-3p	1	27	331
ciRNA53	oar-miR-410-5p	1	6	306
ciRNA2	oar-miR-411a-5p	1	6	267
circRNA427	oar-miR-541-3p	1	19	551
circRNA295	oar-miR-200a	1	485	13200
circRNA50	oar-let-7d	1	41	968
ciRNA81	oar-miR-544-5p	1	12	425
circRNA175	oar-miR-3959-3p	1	9	475
circRNA420	oar-let-7g	1	9	705
circRNA108	oar-miR-29a	1	94	2680
circRNA537	oar-miR-3958-5p	1	37	2640
circRNA285	oar-miR-3956-5p	1	23	991
circRNA775	oar-miR-1197-3p	1	17	410
circRNA720	oar-miR-199a-3p	1	1018	57400
circRNA441	oar-miR-381-5p	1	21	626
circRNA65	oar-miR-370-5p	1	98	6480
circRNA290	oar-miR-654-3p	1	75	4460
circRNA499	oar-miR-133	1	15	581
circRNA177	oar-miR-654-5p	1	17	806
circRNA697	oar-miR-148a	1	21	1080
circRNA121	oar-miR-3956-3p	1	633	14600
circRNA21	oar-miR-544-3p	1	12	695
circRNA477	oar-miR-376a-5p	1	260	6440
circRNA444	oar-let-7g	1	29	1310
circRNA29	oar-let-7c	1	53	1680

circRNA170	oar-miR-30a-3p	1	74	1320
ciRNA44	oar-miR-370-5p	1	4	227
circRNA550	oar-miR-411b-5p	1	59	1040
circRNA651	oar-miR-376b-3p	1	78	1620
circRNA380	oar-miR-3955-3p	1	16	944
circRNA236	oar-miR-136	1	181	4960
circRNA19	oar-miR-376a-5p	1	22	1010
circRNA234	oar-miR-409-3p	1	31	1310
circRNA697	oar-miR-23b	1	21	1080
circRNA367	oar-miR-152	1	93.5	3400
circRNA614	oar-miR-29b	1	50	1590
circRNA482	oar-let-7a	1	275	8290
circRNA648	oar-let-7a	1	39.5	1810
circRNA201	oar-miR-654-5p	1	11	812
ciRNA52	oar-miR-148a	1	8	399
circRNA108	oar-miR-379-5p	1	94	2680
circRNA275	oar-miR-19b	1	165	2850
circRNA273	oar-miR-3957-5p	1	59	1890
circRNA601	oar-miR-433-3p	1	349	19400
circRNA314	oar-miR-30d	1	23	1150
circRNA634	oar-let-7b	1	20	1240
circRNA292	oar-miR-432	1	13	484
circRNA500	oar-miR-431	1	45	1680
circRNA645	oar-miR-412-3p	1	52	2700
circRNA765	oar-miR-26b	1	15	760
circRNA453	oar-miR-668-5p	1	13	374
circRNA386	oar-miR-412-5p	1	102	2110
circRNA295	oar-miR-1197-3p	1	485	13200
circRNA285	oar-let-7i	1	23	991
circRNA52	oar-miR-541-5p	1	14	313
circRNA73	oar-miR-665-5p	1	11	559
circRNA326	oar-miR-379-5p	1	7.5	387
circRNA772	oar-let-7i	1	34	1290
circRNA231	oar-let-7b	1	59	1860
circRNA426	oar-miR-323a-5p	1	5	383
circRNA296	oar-miR-654-3p	1	20	634
circRNA599	oar-miR-376e-5p	1	6	298
ciRNA3	oar-miR-134-3p	1	6	543
circRNA706	oar-miR-665-5p	1	40	938
ciRNA45	oar-let-7f	1	4	312
circRNA697	oar-miR-329b-3p	1	21	1080
circRNA457	oar-miR-323c	1	146	7240
circRNA621	oar-miR-154b-5p	1	46	3840
circRNA685	oar-miR-22-3p	1	172	6290
circRNA712	oar-let-7f	1	120	5180
circRNA45	oar-miR-329b-5p	1	39	1110
circRNA269	oar-miR-191	1	37	2060
circRNA489	oar-miR-412-3p	1	17	1190
circRNA551	oar-miR-654-5p	1	193	4060
circRNA690	oar-miR-665-5p	1	4	284
circRNA323	oar-let-7a	1	8	555
circRNA590	oar-let-7b	1	81	5580
circRNA442	oar-miR-125b	1	32	1980
ciRNA52	oar-miR-25	1	8	399
circRNA565	oar-miR-655-5p	1	45	1450
circRNA776	oar-miR-362	1	27	331
circRNA480	oar-miR-543-5p	1	9	338
ciRNA75	oar-miR-379-3p	1	3	118
circRNA2	oar-miR-3958-5p	1	128	3720
circRNA207	oar-let-7c	1	45	1600
circRNA306	oar-miR-485-5p	1	20	1120
circRNA212	oar-miR-382-5p	1	118	4620
circRNA186	oar-miR-382-5p	1	69	4270
circRNA171	oar-miR-1185-5p	1	166	8060
circRNA300	oar-miR-493-3p	1	142	5630
circRNA781	oar-miR-665-3p	1	8	399
circRNA721	oar-miR-107	1	174	9120
circRNA588	oar-let-7d	1	20	1250
circRNA548	oar-let-7g	1	13	964
circRNA421	oar-miR-181a	1	20	1070
circRNA371	oar-miR-23b	1	27	1490
circRNA218	oar-miR-10a	1	71	4670
circRNA360	oar-let-7f	1	14	1030
circRNA488	oar-miR-487b-5p	1	37	1330
circRNA784	oar-miR-10a	1	1260	41800
ciRNA92	oar-let-7i	1	7	562
circRNA240	oar-miR-494-5p	1	114	4230
circRNA27	oar-miR-380-5p	1	44	2280
circRNA524	oar-miR-494-5p	1	16.5	1190
circRNA565	oar-miR-127	1	45	1450
circRNA354	oar-miR-200c	1	142	3660
ciRNA83	oar-miR-495-3p	1	8	572
circRNA739	oar-miR-3955-3p	1	173	6440
circRNA565	oar-miR-30a-5p	1	45	1450
circRNA352	oar-miR-103	1	45	2060
circRNA496	oar-let-7g	1	90.5	3120
circRNA511	oar-miR-10a	1	59	3000
ciRNA32	oar-miR-134-3p	1	91.5	7530
circRNA610	oar-miR-655-5p	1	83	2110
circRNA637	oar-miR-323b	1	120	2400
circRNA284	oar-miR-3958-3p	1	18	788
circRNA81	oar-miR-29b	1	19	1280
circRNA106	oar-miR-23a	1	60	2410
circRNA607	oar-miR-323a-5p	1	10	722
circRNA197	oar-miR-487b-3p	1	37	2200
circRNA507	oar-miR-410-5p	1	166	4910
circRNA464	oar-miR-487a-5p	1	48	1080
circRNA640	oar-miR-379-5p	1	59	2230

circRNA91	oar-miR-106b	1	38.7	2200
circRNA181	oar-miR-377-5p	1	28	916
circRNA372	oar-miR-30d	1	30	1200
circRNA666	oar-miR-329a-5p	1	17	1060
circRNA367	oar-miR-23a	1	93.5	3400
circRNA103	oar-miR-29b	1	147	2290
circRNA221	oar-miR-376b-3p	1	74	2240
circRNA225	oar-let-7a	1	29	1590
circRNA335	oar-miR-487b-5p	1	34	2110
circRNA767	oar-miR-654-3p	1	45.2	3460
circRNA757	oar-miR-369-5p	1	190	5460
circRNA578	oar-miR-103	1	8	334
circRNA543	oar-miR-103	1	8.5	744
circRNA65	oar-miR-655-5p	1	98	6480
circRNA464	oar-miR-200a	1	48	1080
circRNA192	oar-miR-1197-5p	1	104	3910
circRNA742	oar-miR-370-3p	1	63	2210
circRNA122	oar-miR-409-3p	1	177	5560
circRNA201	oar-miR-654-3p	1	11	812
circRNA125	oar-miR-376e-5p	1	74	3930
circRNA122	oar-miR-494-5p	1	177	5560
circRNA612	oar-miR-432	1	9	348
circRNA166	oar-miR-323a-3p	1	171	4920
circRNA553	oar-miR-329b-5p	1	37	974
circRNA439	oar-miR-3956-3p	1	146	3240
circRNA745	oar-miR-1197-5p	1	34	2350
circRNA570	oar-miR-487a-3p	1	121	6150
circRNA138	oar-miR-665-3p	1	10	640
circRNA247	oar-miR-3959-5p	1	41	1110
circRNA251	oar-miR-411a-5p	1	53	1050
circRNA564	oar-miR-1197-3p	1	28	1490
circRNA628	oar-miR-154a-5p	1	58	2150
circRNA259	oar-miR-382-5p	1	46	1230
circRNA10	oar-miR-30a-3p	1	10	483
circRNA610	oar-miR-25	1	83	2110
circRNA103	oar-let-7c	1	147	2290
circRNA582	oar-miR-133	1	39	1480
circRNA456	oar-miR-3959-3p	1	12	700
circRNA717	oar-miR-411b-5p	1	61.5	3250
circRNA199	oar-miR-1197-3p	1	14	653
circRNA469	oar-miR-668-5p	1	46	2790
circRNA778	oar-miR-107	1	24	1210
circRNA433	oar-miR-3956-5p	1	213	1440
circRNA706	oar-miR-200b	1	40	938
circRNA281	oar-let-7d	1	17	594
circRNA657	oar-miR-107	1	19	843
circRNA270	oar-miR-3957-5p	1	6	273
circRNA153	oar-miR-433-5p	1	19	1110
circRNA178	oar-miR-410-5p	1	16	1380
ciRNA57	oar-miR-30a-3p	1	3	276
circRNA242	oar-miR-329a-5p	1	56	3340
circRNA517	oar-miR-30a-3p	1	9	460
circRNA272	oar-miR-134-3p	1	67	969
circRNA548	oar-miR-26a	1	13	964
circRNA414	oar-miR-200c	1	10	461
circRNA59	oar-miR-668-5p	1	73	3560
circRNA267	oar-miR-25	1	14	591
circRNA75	oar-miR-412-5p	1	45	1530
ciRNA61	oar-miR-133	1	3	261
circRNA560	oar-miR-143	1	24	859
circRNA253	oar-miR-654-5p	1	50	1750
circRNA93	oar-miR-200a	1	291	8600
circRNA725	oar-miR-382-5p	1	79	4390
circRNA768	oar-miR-3957-5p	1	43.7	1850
circRNA641	oar-let-7g	1	14	853
circRNA48	oar-miR-150	1	37	992
circRNA605	oar-miR-370-5p	1	63	526
circRNA75	oar-miR-411a-5p	1	45	1530
circRNA146	oar-miR-323a-5p	1	175	7890
circRNA783	oar-miR-10a	1	339	16100
circRNA504	oar-miR-154b-5p	1	123	5780
circRNA709	oar-miR-1185-3p	1	129	2940
circRNA655	oar-miR-412-3p	1	10	623
circRNA560	oar-miR-409-3p	1	24	859
ciRNA48	oar-miR-665-5p	1	10	329
circRNA194	oar-miR-23a	1	65	3740
circRNA565	oar-miR-107	1	45	1450
circRNA85	oar-miR-3958-3p	1	12	501
circRNA214	oar-miR-3958-5p	1	195	10000
circRNA451	oar-miR-133	1	34	2270
circRNA533	oar-miR-539-5p	1	53	1980
circRNA494	oar-miR-22-3p	1	87	3650
circRNA673	oar-miR-539-3p	1	78	4010
ciRNA92	oar-let-7d	1	7	562
circRNA287	oar-miR-412-5p	1	6	215
circRNA455	oar-let-7f	1	20	994
circRNA99	oar-miR-431	1	17	1090
circRNA453	oar-miR-433-3p	1	13	374
circRNA592	oar-miR-487a-5p	1	68	1670
circRNA124	oar-miR-380-5p	1	157	5240
circRNA252	oar-miR-654-3p	1	612	11700
circRNA391	oar-miR-655-5p	1	19	358
circRNA93	oar-let-7f	1	291	8600
circRNA736	oar-miR-377-5p	1	459	26700
circRNA377	oar-miR-19b	1	124	3890
circRNA36	oar-miR-1193-5p	1	100	2650
ciRNA39	oar-miR-3958-5p	1	86	8130
circRNA416	oar-miR-323a-5p	1	28	1680

ciRNA10	oar-let-7g	1	40	2950
circRNA2	oar-miR-758-3p	1	128	3720
circRNA290	oar-miR-181a	1	75	4460
circRNA46	oar-miR-1197-5p	1	22	1190
ciRNA81	oar-miR-30d	1	12	425
circRNA766	oar-miR-29b	1	86.2	2720
circRNA620	oar-miR-410-5p	1	75	2640
circRNA339	oar-miR-16b	1	9.5	677
ciRNA11	oar-miR-485-5p	1	4	270
circRNA211	oar-miR-433-5p	1	16	820
circRNA505	oar-miR-543-3p	1	6	366
circRNA247	oar-miR-103	1	41	1110
circRNA178	oar-miR-668-3p	1	16	1380
circRNA31	oar-miR-133	1	102	1620
circRNA18	oar-miR-299-3p	1	26	1100
circRNA767	oar-miR-200a	1	45.2	3460
circRNA690	oar-miR-181a	1	4	284
circRNA105	oar-let-7i	1	22	896
circRNA99	oar-miR-376e-3p	1	17	1090
circRNA425	oar-let-7i	1	36	1090
circRNA30	oar-let-7f	1	62	4220
ciRNA94	oar-miR-370-3p	1	3	285
circRNA639	oar-miR-10a	1	71	3330
circRNA106	oar-miR-496-5p	1	60	2410
circRNA598	oar-miR-3956-3p	1	20	1270
circRNA557	oar-miR-496-5p	1	203	9800
circRNA477	oar-miR-134-5p	1	260	6440
circRNA212	oar-miR-539-5p	1	118	4620
ciRNA32	oar-miR-150	1	91.5	7530
circRNA276	oar-miR-106b	1	8	332
circRNA324	oar-miR-382-5p	1	59	1900
circRNA169	oar-let-7i	1	24	1170
circRNA744	oar-let-7a	1	56	1790
circRNA565	oar-miR-30d	1	45	1450
circRNA705	oar-miR-376a-5p	1	20	657
circRNA337	oar-miR-495-5p	1	529	30300
circRNA108	oar-let-7g	1	94	2680
circRNA744	oar-let-7f	1	56	1790
circRNA776	oar-let-7g	1	27	331
circRNA80	oar-miR-370-3p	1	46	1940
circRNA272	oar-miR-199a-3p	1	67	969
circRNA518	oar-miR-432	1	15	1530
circRNA157	oar-miR-133	1	268	16300
circRNA169	oar-miR-379-5p	1	24	1170
circRNA533	oar-miR-379-3p	1	53	1980
circRNA5	oar-miR-412-5p	1	23	942
circRNA646	oar-miR-323a-5p	1	19	876
circRNA278	oar-miR-432	1	65	2960
circRNA568	oar-miR-3959-5p	1	166	4830
circRNA773	oar-miR-655-5p	1	25	1110
circRNA251	oar-miR-369-5p	1	53	1050
circRNA753	oar-miR-544-3p	1	109	3710
circRNA257	oar-miR-10b	1	118	7890
circRNA403	oar-miR-382-5p	1	32	1980
circRNA49	oar-miR-148a	1	23	1490
circRNA147	oar-miR-432	1	12	556
circRNA7	oar-let-7g	1	47	1830
circRNA544	oar-miR-376a-3p	1	58.5	2700
circRNA758	oar-miR-494-3p	1	69	2410
circRNA445	oar-let-7g	1	17	958
circRNA211	oar-miR-143	1	16	820
circRNA120	oar-miR-3958-3p	1	20	965
circRNA646	oar-miR-221	1	19	876
circRNA205	oar-let-7a	1	34	1150
circRNA212	oar-miR-494-5p	1	118	4620
circRNA91	oar-miR-23a	1	38.7	2200
circRNA58	oar-miR-150	1	24	1080
circRNA771	oar-miR-668-3p	1	30	1530
circRNA274	oar-miR-19b	1	66	2070
circRNA37	oar-miR-487a-3p	1	61	2680
circRNA497	oar-miR-362	1	47.5	1540
circRNA163	oar-miR-3958-5p	1	28	1180
circRNA260	oar-miR-495-5p	1	42	1370
circRNA631	oar-miR-30a-3p	1	379	9240
circRNA262	oar-miR-412-3p	1	24	975
circRNA543	oar-miR-16b	1	8.5	744
circRNA693	oar-miR-1197-5p	1	27	842
circRNA240	oar-miR-493-3p	1	114	4230
circRNA280	oar-miR-432	1	30	1530
circRNA19	oar-miR-152	1	22	1010
circRNA252	oar-miR-154b-5p	1	612	11700
circRNA488	oar-miR-29a	1	37	1330
circRNA386	oar-miR-369-5p	1	102	2110
circRNA168	oar-miR-494-5p	1	45	1730
circRNA316	oar-miR-654-5p	1	19	1020
circRNA611	oar-miR-199a-3p	1	40	1830
circRNA162	oar-miR-377-3p	1	8	320
circRNA46	oar-miR-654-5p	1	22	1190
circRNA496	oar-miR-758-3p	1	90.5	3120
circRNA532	oar-miR-374b	1	731	18000
circRNA575	oar-miR-487a-5p	1	76	3630
circRNA711	oar-miR-3959-3p	1	63	2820
circRNA504	oar-let-7g	1	123	5780
circRNA364	oar-miR-154b-5p	1	140	6320
circRNA365	oar-miR-323a-5p	1	25	611
circRNA181	oar-miR-487b-3p	1	28	916
circRNA421	oar-miR-494-5p	1	20	1070
circRNA464	oar-miR-200c	1	48	1080

circRNA648	oar-miR-323a-5p	1	39.5	1810
circRNA776	oar-miR-329b-5p	1	27	331
circRNA50	oar-let-7c	1	41	968
circRNA39	oar-miR-3958-3p	1	11	513
circRNA143	oar-miR-493-3p	1	59	4880
circRNA773	oar-miR-329b-5p	1	25	1110
circRNA768	oar-miR-200a	1	43.7	1850
circRNA765	oar-let-7b	1	15	760
circRNA133	oar-miR-495-5p	1	33	1900
circRNA714	oar-miR-543-5p	1	23	1190
circRNA712	oar-let-7i	1	120	5180
circRNA489	oar-miR-411b-5p	1	17	1190
ciRNA83	oar-miR-541-5p	1	8	572
circRNA190	oar-miR-133	1	10	840
circRNA604	oar-miR-154a-3p	1	70	3730
ciRNA60	oar-miR-329a-3p	1	18	809
circRNA512	oar-miR-154b-5p	1	24	1520
circRNA505	oar-miR-541-5p	1	6	366
circRNA114	oar-miR-154b-5p	1	350	14700
circRNA622	oar-miR-23b	1	93	5000
circRNA642	oar-miR-136	1	22	950
circRNA103	oar-miR-26a	1	147	2290
circRNA407	oar-miR-411b-5p	1	44	2870
circRNA171	oar-miR-376e-3p	1	166	8060
circRNA236	oar-miR-329b-5p	1	181	4960
circRNA327	oar-miR-181a	1	27	1070
circRNA464	oar-miR-329a-5p	1	48	1080
circRNA571	oar-miR-22-3p	1	25	1300
circRNA40	oar-miR-409-5p	1	22	1130
circRNA499	oar-miR-370-3p	1	15	581
circRNA481	oar-miR-665-3p	1	44	2590
circRNA568	oar-miR-376a-5p	1	166	4830
circRNA229	oar-miR-370-3p	1	48	845
circRNA371	oar-miR-432	1	27	1490
circRNA658	oar-miR-134-5p	1	38	2500
circRNA164	oar-miR-487b-5p	1	107	5430
circRNA386	oar-miR-136	1	102	2110
circRNA707	oar-let-7f	1	71	2950
circRNA713	oar-miR-30a-3p	1	10	612
circRNA773	oar-miR-411b-5p	1	25	1110
circRNA378	oar-miR-19b	1	42.5	1830
circRNA474	oar-miR-668-3p	1	427	25200
circRNA447	oar-miR-23a	1	48	1760
circRNA285	oar-miR-382-5p	1	23	991
circRNA580	oar-miR-433-5p	1	19	784
circRNA103	oar-miR-665-3p	1	147	2290
circRNA496	oar-let-7b	1	90.5	3120
circRNA273	oar-miR-487b-5p	1	59	1890
circRNA586	oar-miR-376c-3p	1	12	765
circRNA2	oar-miR-1197-3p	1	128	3720
circRNA115	oar-let-7i	1	280	9430
circRNA764	oar-miR-99a	1	159	6640
circRNA50	oar-miR-3959-3p	1	41	968
circRNA356	oar-let-7g	1	152	6210
circRNA753	oar-miR-329a-5p	1	109	3710
circRNA4	oar-miR-10a	1	8	593
circRNA24	oar-miR-10a	1	34	2370
circRNA717	oar-miR-21	1	61.5	3250
circRNA87	oar-miR-493-3p	1	22	1330
circRNA24	oar-let-7i	1	34	2370
circRNA278	oar-miR-1193-3p	1	65	2960
circRNA592	oar-miR-154b-5p	1	68	1670
circRNA690	oar-miR-543-3p	1	4	284
circRNA170	oar-miR-655-5p	1	74	1320
circRNA426	oar-miR-10a	1	5	383
circRNA307	oar-miR-369-5p	1	341	21400
circRNA182	oar-miR-382-5p	1	192	6140
circRNA563	oar-miR-329b-5p	1	106	2740
circRNA154	oar-let-7f	1	27	1160
circRNA656	oar-miR-133	1	30	1290
circRNA188	oar-miR-329a-5p	1	35	1300
ciRNA97	oar-miR-3959-3p	1	1	85.7
circRNA241	oar-miR-134-3p	1	45	2440
circRNA463	oar-miR-3956-3p	1	40	2200
circRNA291	oar-miR-143	1	23	830
ciRNA97	oar-miR-410-3p	1	1	85.7
circRNA680	oar-miR-485-3p	1	14	820
circRNA137	oar-miR-125b	1	24	849
circRNA177	oar-miR-379-5p	1	17	806
circRNA460	oar-miR-323a-5p	1	249	6760
circRNA350	oar-miR-382-5p	1	9	581
circRNA117	oar-miR-493-3p	1	5	521
circRNA647	oar-miR-493-5p	1	110	5290
circRNA496	oar-miR-323a-5p	1	90.5	3120
circRNA263	oar-miR-409-5p	1	17	1240
circRNA167	oar-miR-191	1	14	900
circRNA669	oar-miR-433-5p	1	178	6140
circRNA265	oar-miR-3955-5p	1	15	215
circRNA50	oar-miR-377-5p	1	41	968
circRNA114	oar-miR-493-5p	1	350	14700
circRNA774	oar-miR-3958-3p	1	63	3650
circRNA317	oar-miR-381-3p	1	223	11200
circRNA709	oar-miR-380-5p	1	129	2940
circRNA631	oar-miR-3958-5p	1	379	9240
circRNA79	oar-miR-411b-5p	1	22	1320
circRNA149	oar-miR-329b-3p	1	71	3740
circRNA466	oar-miR-30b	1	236	14900
circRNA95	oar-miR-495-5p	1	28	1150

circRNA386	oar-miR-544-3p	1	102	2110
circRNA535	oar-miR-493-3p	1	17	769
circRNA29	oar-miR-3955-3p	1	53	1680
circRNA679	oar-miR-376a-5p	1	45	2240
circRNA707	oar-miR-374b	1	71	2950
circRNA352	oar-miR-16b	1	45	2060
circRNA539	oar-miR-30c	1	29	1360
circRNA311	oar-miR-654-3p	1	32	2310
circRNA106	oar-miR-411a-5p	1	60	2410
circRNA74	oar-miR-411b-5p	1	16	1040
ciRNA79	oar-miR-125b	1	35	2410
circRNA508	oar-miR-655-5p	1	126	10700
circRNA82	oar-miR-1185-3p	1	81	5460
circRNA771	oar-miR-329b-5p	1	30	1530
circRNA462	oar-miR-541-5p	1	43	1210
circRNA382	oar-miR-376b-5p	1	26	984
circRNA27	oar-miR-485-5p	1	44	2280
circRNA310	oar-miR-412-3p	1	150	3620
circRNA452	oar-miR-103	1	5	470
circRNA188	oar-miR-377-5p	1	35	1300
circRNA31	oar-miR-655-5p	1	102	1620
circRNA471	oar-miR-409-3p	1	63	1820
circRNA344	oar-miR-493-3p	1	19	583
circRNA589	oar-miR-127	1	11	260
circRNA622	oar-miR-23a	1	93	5000
circRNA345	oar-miR-493-3p	1	32	1330
circRNA679	oar-miR-323b	1	45	2240
circRNA182	oar-miR-107	1	192	6140
circRNA490	oar-miR-376a-5p	1	240	5160
circRNA229	oar-miR-381-5p	1	48	845
circRNA147	oar-miR-412-3p	1	12	556
circRNA393	oar-miR-376e-5p	1	9	450
circRNA304	oar-miR-362	1	262	7540
circRNA716	oar-let-7d	1	83.5	2960
circRNA101	oar-miR-191	1	4	119
circRNA500	oar-let-7b	1	45	1680
circRNA489	oar-miR-3956-5p	1	17	1190
circRNA747	oar-miR-377-3p	1	26	1800
circRNA216	oar-miR-494-5p	1	32	925
circRNA369	oar-miR-154a-5p	1	33	1560
circRNA116	oar-miR-1185-3p	1	342	14200
circRNA614	oar-miR-17-5p	1	50	1590
circRNA114	oar-miR-107	1	350	14700
circRNA540	oar-miR-665-3p	1	87	3030
ciRNA2	oar-miR-409-5p	1	6	267
ciRNA87	oar-miR-1197-5p	1	35.3	2480
circRNA252	oar-miR-218a	1	612	11700
circRNA676	oar-miR-374a	1	6	537
circRNA317	oar-let-7f	1	223	11200
circRNA28	oar-miR-329b-5p	1	23	872
circRNA122	oar-miR-154b-3p	1	177	5560
ciRNA7	oar-miR-487a-5p	1	4	433
circRNA139	oar-miR-154b-5p	1	6	654
circRNA498	oar-miR-377-5p	1	15	657
circRNA501	oar-miR-107	1	218	11300
circRNA763	oar-miR-412-5p	1	337	17400
circRNA613	oar-miR-3957-3p	1	21	735
circRNA595	oar-miR-3958-5p	1	12	672
circRNA430	oar-miR-369-3p	1	319	2550
circRNA325	oar-miR-668-3p	1	39.5	1570
circRNA719	oar-miR-411b-3p	1	21	1150
circRNA259	oar-miR-654-3p	1	46	1230
circRNA473	oar-miR-134-5p	1	8	499
circRNA144	oar-miR-329b-5p	1	16	798
circRNA623	oar-miR-154a-3p	1	91	4670
circRNA499	oar-miR-1197-3p	1	15	581
circRNA371	oar-miR-136	1	27	1490
circRNA221	oar-miR-1193-5p	1	74	2240
circRNA487	oar-miR-412-5p	1	33	1440
circRNA570	oar-miR-758-3p	1	121	6150
ciRNA50	oar-miR-485-5p	1	36	3220
circRNA680	oar-miR-377-5p	1	14	820
circRNA436	oar-miR-412-5p	1	123	2820
circRNA771	oar-miR-495-5p	1	30	1530
circRNA584	oar-miR-377-5p	1	318	12200
circRNA279	oar-let-7b	1	71	3110
circRNA768	oar-miR-200b	1	43.7	1850
circRNA93	oar-miR-329b-5p	1	291	8600
circRNA775	oar-miR-329a-5p	1	17	410
circRNA712	oar-miR-411a-5p	1	120	5180
circRNA355	oar-miR-487a-5p	1	342	13100
circRNA500	oar-let-7i	1	45	1680
circRNA118	oar-miR-106a	1	32	1790
ciRNA52	oar-miR-152	1	8	399
circRNA716	oar-let-7a	1	83.5	2960
circRNA360	oar-let-7b	1	14	1030
circRNA279	oar-let-7i	1	71	3110
circRNA260	oar-let-7c	1	42	1370
circRNA111	oar-miR-668-5p	1	181	4380
circRNA60	oar-miR-493-3p	1	66	3680
circRNA502	oar-miR-329b-3p	1	17	975
circRNA123	oar-miR-154b-5p	1	24.5	1030
circRNA60	oar-miR-10b	1	66	3680
circRNA778	oar-miR-103	1	24	1210
circRNA265	oar-miR-329a-3p	1	15	215
circRNA518	oar-miR-143	1	15	1530
circRNA334	oar-miR-127	1	176	11100
circRNA119	oar-miR-329a-5p	1	3	171

circRNA489	oar-miR-380-5p	1	17	1190
circRNA80	oar-miR-370-5p	1	46	1940
ciRNA53	oar-miR-1197-5p	1	6	306
circRNA338	oar-miR-200a	1	45.5	2180
circRNA246	oar-miR-3956-3p	1	53	1940
circRNA678	oar-miR-16b	1	24	1290
circRNA348	oar-miR-541-3p	1	18	1100
circRNA265	oar-miR-496-5p	1	15	215
circRNA51	oar-miR-487a-3p	1	20	664
circRNA658	oar-miR-106b	1	38	2500
circRNA346	oar-miR-495-5p	1	15	570
circRNA51	oar-miR-1193-3p	1	20	664
circRNA418	oar-miR-376a-5p	1	32	1810
circRNA199	oar-miR-22-3p	1	14	653
circRNA641	oar-let-7a	1	14	853
circRNA447	oar-miR-27a	1	48	1760
circRNA506	oar-miR-409-5p	1	29	1530
circRNA455	oar-miR-194	1	20	994
circRNA211	oar-miR-323a-5p	1	16	820
circRNA773	oar-let-7f	1	25	1110
circRNA260	oar-miR-3957-5p	1	42	1370
circRNA74	oar-miR-134-5p	1	16	1040
circRNA713	oar-miR-382-5p	1	10	612
circRNA375	oar-miR-323a-5p	1	16	1010
circRNA742	oar-miR-543-5p	1	63	2210
ciRNA79	oar-miR-136	1	35	2410
circRNA148	oar-let-7g	1	213	12600
circRNA182	oar-miR-21	1	192	6140
circRNA31	oar-miR-370-5p	1	102	1620
circRNA268	oar-miR-376e-3p	1	6	339
circRNA754	oar-miR-381-3p	1	32	2040
circRNA499	oar-miR-3955-5p	1	15	581
circRNA311	oar-miR-27a	1	32	2310
circRNA575	oar-miR-136	1	76	3630
circRNA777	oar-miR-380-5p	1	10	409
circRNA696	oar-miR-493-3p	1	10	490
circRNA578	oar-miR-3959-5p	1	8	334
circRNA321	oar-miR-1185-5p	1	48	2450
circRNA650	oar-miR-382-5p	1	56	4980
circRNA447	oar-miR-134-3p	1	48	1760
ciRNA73	oar-miR-3958-5p	1	8	675
circRNA124	oar-miR-543-3p	1	157	5240
circRNA78	oar-miR-432	1	47	1420
circRNA686	oar-miR-376d	1	30	923
circRNA605	oar-miR-495-5p	1	63	526
ciRNA2	oar-miR-376b-5p	1	6	267
circRNA458	oar-miR-382-5p	1	9	549
circRNA451	oar-let-7c	1	34	2270
circRNA726	oar-miR-154b-5p	1	64	4520
circRNA370	oar-let-7a	1	39	1490
circRNA305	oar-miR-329b-5p	1	52	2410
circRNA660	oar-miR-493-3p	1	5	481
circRNA38	oar-miR-329a-5p	1	117	7230
circRNA673	oar-miR-154a-3p	1	78	4010
circRNA539	oar-miR-30b	1	29	1360
circRNA18	oar-miR-26a	1	26	1100
circRNA126	oar-miR-431	1	19	1280
circRNA535	oar-miR-25	1	17	769
circRNA110	oar-miR-125b	1	62	1800
circRNA771	oar-miR-27a	1	30	1530
circRNA467	oar-miR-10b	1	28	1050
circRNA3	oar-miR-127	1	99	4710
circRNA29	oar-miR-3958-5p	1	53	1680
circRNA45	oar-miR-299-3p	1	39	1110
circRNA666	oar-miR-377-5p	1	17	1060
circRNA692	oar-miR-410-5p	1	24	831
circRNA538	oar-miR-665-5p	1	41	1830
circRNA315	oar-miR-412-5p	1	32	1880
circRNA354	oar-miR-154b-3p	1	142	3660
circRNA607	oar-miR-758-3p	1	10	722
circRNA19	oar-miR-409-5p	1	22	1010
circRNA183	oar-miR-134-3p	1	189	9040
circRNA381	oar-let-7i	1	29	1720
circRNA241	oar-let-7i	1	45	2440
circRNA736	oar-miR-103	1	459	26700
circRNA593	oar-let-7f	1	37	1850
circRNA689	oar-miR-494-3p	1	16	927
circRNA106	oar-miR-380-5p	1	60	2410
circRNA473	oar-miR-29b	1	8	499
circRNA105	oar-miR-432	1	22	896
circRNA781	oar-miR-154b-5p	1	8	399
circRNA247	oar-miR-107	1	41	1110
circRNA777	oar-miR-329b-3p	1	10	409
circRNA225	oar-let-7d	1	29	1590
circRNA296	oar-miR-22-3p	1	20	634
circRNA516	oar-miR-539-3p	1	14	976
circRNA137	oar-miR-221	1	24	849
circRNA776	oar-miR-30a-3p	1	27	331
ciRNA46	oar-miR-30a-5p	1	3	201
circRNA589	oar-miR-411a-5p	1	11	260
circRNA197	oar-miR-134-3p	1	37	2200
circRNA276	oar-miR-431	1	8	332
circRNA604	oar-miR-379-3p	1	70	3730
circRNA501	oar-miR-541-3p	1	218	11300
circRNA345	oar-miR-3957-5p	1	32	1330
circRNA380	oar-miR-154b-5p	1	16	944
circRNA47	oar-miR-133	1	122	5840
circRNA657	oar-miR-493-3p	1	19	843

circRNA10	oar-miR-3955-3p	1	10	483
circRNA683	oar-let-7c	1	7	686
circRNA642	oar-miR-1197-3p	1	22	950
circRNA337	oar-miR-26a	1	529	30300
circRNA109	oar-miR-541-5p	1	42	2010
circRNA534	oar-miR-25	1	18	721
circRNA519	oar-miR-329a-5p	1	18	1590
circRNA631	oar-miR-654-3p	1	379	9240
circRNA421	oar-miR-329b-5p	1	20	1070
circRNA484	oar-miR-485-5p	1	135	4150
circRNA287	oar-miR-539-3p	1	6	215
circRNA726	oar-miR-3957-5p	1	64	4520
circRNA686	oar-miR-218a	1	30	923
circRNA766	oar-miR-29a	1	86.2	2720
circRNA386	oar-miR-29a	1	102	2110
circRNA614	oar-miR-23a	1	50	1590
ciRNA11	oar-miR-1193-5p	1	4	270
circRNA139	oar-miR-329a-5p	1	6	654
circRNA421	oar-let-7f	1	20	1070
circRNA122	oar-miR-127	1	177	5560
circRNA600	oar-miR-3956-3p	1	34	1850
circRNA291	oar-miR-329a-3p	1	23	830
circRNA42	oar-miR-323b	1	193	3260
circRNA666	oar-miR-496-3p	1	17	1060
circRNA224	oar-miR-433-5p	1	37	1600
circRNA147	oar-miR-134-3p	1	12	556
circRNA479	oar-miR-154b-3p	1	94	4200
circRNA218	oar-miR-377-3p	1	71	4670
ciRNA95	oar-miR-1197-5p	1	4	383
circRNA374	oar-miR-323a-5p	1	33	1050
circRNA515	oar-miR-136	1	17	663
circRNA676	oar-miR-493-5p	1	6	537
ciRNA64	oar-miR-3959-3p	1	7	702
circRNA463	oar-miR-665-3p	1	40	2200
circRNA13	oar-miR-154b-5p	1	279	13500
circRNA715	oar-miR-654-3p	1	64	2040
circRNA41	oar-miR-16b	1	9	617
circRNA536	oar-miR-487a-3p	1	87	4870
circRNA30	oar-let-7a	1	62	4220
circRNA777	oar-miR-154a-5p	1	10	409
circRNA33	oar-miR-199a-3p	1	34	1890
circRNA353	oar-miR-200c	1	21	1130
circRNA37	oar-miR-143	1	61	2680
circRNA552	oar-miR-329a-5p	1	40	2810
circRNA13	oar-miR-3957-5p	1	279	13500
circRNA536	oar-miR-543-3p	1	87	4870
ciRNA33	oar-miR-494-3p	1	3	309
circRNA160	oar-miR-433-5p	1	31	1750
circRNA644	oar-miR-370-3p	1	30	1540
circRNA299	oar-miR-665-3p	1	8	590
circRNA342	oar-miR-30b	1	45	2070
circRNA405	oar-miR-29b	1	53	3730
circRNA331	oar-miR-154a-5p	1	32	2510
circRNA256	oar-miR-323c	1	58	4320
circRNA711	oar-miR-654-5p	1	63	2820
circRNA455	oar-let-7g	1	20	994
circRNA276	oar-miR-370-3p	1	8	332
circRNA435	oar-miR-411b-5p	1	20.2	602
circRNA51	oar-miR-485-3p	1	20	664
circRNA97	oar-let-7a	1	22	1350
circRNA496	oar-miR-362	1	90.5	3120
circRNA236	oar-miR-152	1	181	4960
circRNA421	oar-miR-329a-5p	1	20	1070
circRNA403	oar-miR-1193-5p	1	32	1980
circRNA487	oar-miR-1193-5p	1	33	1440
circRNA653	oar-miR-487a-3p	1	16	332
circRNA542	oar-miR-409-5p	1	16	715
circRNA589	oar-let-7b	1	11	260
circRNA327	oar-miR-487a-5p	1	27	1070
circRNA347	oar-miR-493-3p	1	44	2980
circRNA551	oar-miR-376b-3p	1	193	4060
circRNA441	oar-miR-26a	1	21	626
circRNA743	oar-miR-377-3p	1	291	8460
circRNA532	oar-miR-218a	1	731	18000
circRNA180	oar-miR-1197-3p	1	30	2030
circRNA223	oar-miR-3959-3p	1	34	1820
circRNA754	oar-miR-377-5p	1	32	2040
circRNA587	oar-miR-541-5p	1	24	1480
circRNA588	oar-let-7b	1	20	1250
circRNA731	oar-miR-493-5p	1	15	407
circRNA306	oar-miR-1197-5p	1	20	1120
circRNA484	oar-miR-382-3p	1	135	4150
circRNA25	oar-miR-134-3p	1	27	1840
circRNA487	oar-miR-3957-5p	1	33	1440
circRNA670	oar-miR-218a	1	364	16900
circRNA241	oar-let-7b	1	45	2440
ciRNA92	oar-let-7b	1	7	562
circRNA69	oar-miR-487a-5p	1	6	525
circRNA753	oar-miR-154b-5p	1	109	3710
circRNA44	oar-let-7c	1	61	1930
circRNA324	oar-miR-379-5p	1	59	1900
circRNA261	oar-miR-3959-5p	1	384	11400
circRNA787	oar-miR-150	1	83.5	5290
circRNA301	oar-miR-148a	1	111	6260
circRNA623	oar-miR-25	1	91	4670
circRNA532	oar-miR-154b-3p	1	731	18000
circRNA23	oar-miR-496-3p	1	70	5360
circRNA551	oar-miR-432	1	193	4060

circRNA234	oar-miR-19b	1	31	1310
circRNA541	oar-miR-758-3p	1	58	4700
ciRNA11	oar-miR-3957-5p	1	4	270
circRNA116	oar-let-7g	1	342	14200
circRNA406	oar-miR-485-5p	1	148	5360
circRNA356	oar-miR-412-3p	1	152	6210
circRNA310	oar-miR-329a-5p	1	150	3620
circRNA203	oar-miR-487b-5p	1	74	4560
circRNA183	oar-miR-381-5p	1	189	9040
circRNA746	oar-miR-665-5p	1	108	3350
circRNA116	oar-miR-411b-5p	1	342	14200
circRNA397	oar-miR-329b-5p	1	54	2570
circRNA227	oar-miR-654-5p	1	122	4390
circRNA337	oar-miR-655-5p	1	529	30300
circRNA766	oar-miR-136	1	86.2	2720
circRNA693	oar-miR-432	1	27	842
circRNA536	oar-miR-485-3p	1	87	4870
circRNA3	oar-miR-148a	1	99	4710
circRNA386	oar-miR-665-3p	1	102	2110
circRNA439	oar-miR-431	1	146	3240
circRNA757	oar-miR-380-5p	1	190	5460
circRNA365	oar-miR-3957-5p	1	25	611
circRNA5	oar-miR-377-5p	1	23	942
circRNA688	oar-miR-412-3p	1	32	2450
circRNA32	oar-miR-200a	1	28	1400
circRNA283	oar-miR-494-5p	1	219	14800
circRNA255	oar-miR-544-3p	1	63	4490
circRNA32	oar-miR-410-5p	1	28	1400
circRNA322	oar-let-7a	1	130	4390
circRNA298	oar-miR-496-5p	1	28	774
ciRNA37	oar-miR-1193-5p	1	22	666
circRNA149	oar-miR-668-5p	1	71	3740
circRNA346	oar-miR-493-5p	1	15	570
circRNA106	oar-miR-495-5p	1	60	2410
circRNA252	oar-miR-200a	1	612	11700
circRNA716	oar-miR-412-3p	1	83.5	2960
circRNA145	oar-miR-379-3p	1	79	3280
ciRNA52	oar-miR-665-3p	1	8	399
circRNA245	oar-miR-362	1	26	1730
circRNA639	oar-miR-23a	1	71	3330
circRNA31	oar-miR-30a-3p	1	102	1620
circRNA424	oar-miR-377-3p	1	48	2000
circRNA103	oar-let-7a	1	147	2290
circRNA529	oar-miR-493-3p	1	50	1940
circRNA651	oar-miR-3957-5p	1	78	1620
circRNA536	oar-miR-136	1	87	4870
circRNA182	oar-miR-136	1	192	6140
circRNA370	oar-miR-25	1	39	1490
circRNA272	oar-miR-3955-5p	1	67	969
ciRNA66	oar-miR-411a-5p	1	6	528
circRNA10	oar-miR-148a	1	10	483
circRNA290	oar-miR-107	1	75	4460
circRNA372	oar-let-7f	1	30	1200
circRNA244	oar-miR-654-3p	1	71	3830
circRNA623	oar-miR-654-3p	1	91	4670
circRNA770	oar-miR-541-3p	1	7	452
circRNA460	oar-miR-370-3p	1	249	6760
circRNA74	oar-miR-485-5p	1	16	1040
circRNA100	oar-miR-200a	1	20	767
ciRNA81	oar-miR-30b	1	12	425
circRNA454	oar-miR-495-5p	1	17	1530
circRNA742	oar-miR-376b-5p	1	63	2210
circRNA80	oar-miR-433-5p	1	46	1940
circRNA310	oar-miR-758-5p	1	150	3620
circRNA229	oar-miR-148a	1	48	845
circRNA181	oar-let-7f	1	28	916
circRNA98	oar-miR-369-5p	1	13	697
circRNA599	oar-miR-25	1	6	298
circRNA236	oar-miR-148a	1	181	4960
circRNA452	oar-miR-495-5p	1	5	470
circRNA72	oar-miR-23a	1	35	1280
ciRNA31	oar-miR-410-5p	1	90.5	7640
circRNA275	oar-miR-17-5p	1	165	2850
circRNA377	oar-miR-329a-3p	1	124	3890
circRNA463	oar-miR-10a	1	40	2200
circRNA450	oar-miR-136	1	39.5	1570
circRNA124	oar-miR-654-5p	1	157	5240
circRNA524	oar-miR-329a-5p	1	16.5	1190
circRNA645	oar-miR-495-5p	1	52	2700
circRNA605	oar-miR-496-5p	1	63	526
circRNA14	oar-miR-154a-3p	1	99	3350
circRNA75	oar-miR-409-5p	1	45	1530
circRNA20	oar-miR-487b-5p	1	86	2300
circRNA216	oar-miR-191	1	32	925
circRNA126	oar-miR-541-5p	1	19	1280
circRNA72	oar-miR-22-3p	1	35	1280
circRNA645	oar-miR-199a-3p	1	52	2700
circRNA224	oar-miR-493-3p	1	37	1600
circRNA20	oar-miR-654-3p	1	86	2300
circRNA207	oar-let-7f	1	45	1600
circRNA404	oar-miR-329a-3p	1	29	2710
circRNA768	oar-miR-29b	1	43.7	1850
circRNA495	oar-miR-541-5p	1	134	5930
circRNA747	oar-miR-30a-3p	1	26	1800
circRNA298	oar-miR-432	1	28	774
circRNA106	oar-miR-221	1	60	2410
circRNA694	oar-miR-485-5p	1	109	3640
circRNA406	oar-miR-181a	1	148	5360

circRNA217	oar-miR-487a-3p	1	93	4010
circRNA609	oar-miR-412-3p	1	49	1860
circRNA248	oar-miR-16b	1	6	370
circRNA426	oar-miR-1193-5p	1	5	383
circRNA592	oar-miR-136	1	68	1670
circRNA498	oar-let-7i	1	15	657
circRNA788	oar-miR-541-5p	1	400	16300
circRNA590	oar-let-7i	1	81	5580
circRNA592	oar-miR-409-3p	1	68	1670
circRNA122	oar-miR-22-3p	1	177	5560
circRNA647	oar-let-7b	1	110	5290
circRNA36	oar-miR-3955-5p	1	100	2650
circRNA148	oar-miR-1197-5p	1	213	12600
circRNA375	oar-let-7f	1	16	1010
circRNA251	oar-miR-496-3p	1	53	1050
circRNA774	oar-miR-200b	1	63	3650
circRNA651	oar-miR-154a-5p	1	78	1620
circRNA785	oar-miR-200b	1	2	135
circRNA109	oar-miR-376a-5p	1	42	2010
circRNA496	oar-miR-154b-5p	1	90.5	3120
circRNA171	oar-miR-199a-3p	1	166	8060
ciRNA36	oar-let-7a	1	17	1010
circRNA139	oar-miR-411b-5p	1	6	654
circRNA512	oar-miR-10a	1	24	1520
circRNA209	oar-miR-494-3p	1	2	97.7
circRNA463	oar-miR-329b-5p	1	40	2200
circRNA60	oar-miR-329b-3p	1	66	3680
circRNA462	oar-miR-125b	1	43	1210
circRNA697	oar-miR-23a	1	21	1080
circRNA301	oar-miR-3955-5p	1	111	6260
circRNA260	oar-miR-543-5p	1	42	1370
circRNA551	oar-miR-1197-3p	1	193	4060
circRNA645	oar-miR-200a	1	52	2700
circRNA203	oar-miR-19b	1	74	4560
circRNA210	oar-miR-3955-3p	1	131	5360
circRNA600	oar-miR-3958-5p	1	34	1850
circRNA195	oar-miR-103	1	53	1510
circRNA610	oar-miR-409-5p	1	83	2110
circRNA693	oar-miR-654-3p	1	27	842
circRNA666	oar-miR-329b-5p	1	17	1060
circRNA448	oar-miR-382-5p	1	7	450
circRNA77	oar-miR-154b-5p	1	418	15500
circRNA560	oar-miR-376a-5p	1	24	859
ciRNA61	oar-miR-665-5p	1	3	261
circRNA197	oar-miR-493-3p	1	37	2200
circRNA262	oar-miR-26a	1	24	975
circRNA176	oar-miR-299-5p	1	24	1150
circRNA398	oar-miR-27a	1	135	4930
circRNA666	oar-miR-1193-3p	1	17	1060
circRNA492	oar-miR-377-5p	1	157	7210
circRNA400	oar-miR-655-5p	1	28	922
circRNA110	oar-miR-323a-5p	1	62	1800
circRNA534	oar-miR-544-3p	1	18	721
circRNA391	oar-miR-433-5p	1	19	358
circRNA370	oar-miR-431	1	39	1490
ciRNA84	oar-miR-433-3p	1	6	645
circRNA716	oar-miR-494-5p	1	83.5	2960
circRNA327	oar-miR-369-5p	1	27	1070
circRNA286	oar-miR-22-3p	1	50	1870
ciRNA1	oar-miR-380-3p	1	4	354
circRNA44	oar-miR-370-3p	1	61	1930
circRNA434	oar-miR-125b	1	167	1280
circRNA11	oar-miR-329a-5p	1	11	576
circRNA39	oar-let-7g	1	11	513
circRNA683	oar-let-7d	1	7	686
circRNA3	oar-miR-494-5p	1	99	4710
circRNA646	oar-miR-30a-3p	1	19	876
circRNA124	oar-miR-758-5p	1	157	5240
circRNA327	oar-miR-495-5p	1	27	1070
circRNA65	oar-miR-1193-3p	1	98	6480
circRNA278	oar-miR-19b	1	65	2960
circRNA365	oar-miR-194	1	25	611
circRNA195	oar-miR-1197-5p	1	53	1510
circRNA229	oar-miR-665-5p	1	48	845
circRNA712	oar-miR-487a-5p	1	120	5180
ciRNA21	oar-miR-27a	1	3	147
circRNA234	oar-miR-369-5p	1	31	1310
circRNA29	oar-let-7f	1	53	1680
circRNA326	oar-miR-3957-5p	1	7.5	387
circRNA199	oar-miR-493-3p	1	14	653
ciRNA60	oar-miR-541-3p	1	18	809
circRNA445	oar-miR-1197-3p	1	17	958
circRNA451	oar-miR-493-3p	1	34	2270
circRNA98	oar-miR-1197-3p	1	13	697
circRNA471	oar-miR-16b	1	63	1820
circRNA605	oar-miR-412-3p	1	63	526
circRNA756	oar-miR-23a	1	68	2660
circRNA776	oar-miR-665-3p	1	27	331
circRNA183	oar-miR-136	1	189	9040
circRNA136	oar-miR-323a-5p	1	24	1000
circRNA283	oar-miR-655-5p	1	219	14800
circRNA125	oar-miR-409-5p	1	74	3930
circRNA704	oar-miR-27a	1	49	2550
circRNA359	oar-miR-3958-5p	1	93	3890
circRNA607	oar-miR-107	1	10	722
circRNA379	oar-miR-1193-5p	1	155	6470
circRNA744	oar-let-7g	1	56	1790
circRNA606	oar-miR-103	1	95	3890

circRNA292	oar-let-7d	1	13	484
circRNA562	oar-miR-154b-5p	1	4	368
circRNA532	oar-miR-369-3p	1	731	18000
circRNA112	oar-miR-29a	1	112	4570
circRNA500	oar-miR-665-3p	1	45	1680
circRNA437	oar-miR-541-5p	1	17	1130
circRNA236	oar-miR-432	1	181	4960
ciRNA30	oar-miR-362	1	2	84.4
circRNA324	oar-miR-409-5p	1	59	1900
circRNA571	oar-miR-487a-3p	1	25	1300
circRNA507	oar-miR-370-5p	1	166	4910
circRNA45	oar-miR-544-3p	1	39	1110
circRNA416	oar-miR-654-5p	1	28	1680
circRNA323	oar-miR-412-3p	1	8	555
circRNA553	oar-miR-133	1	37	974
circRNA223	oar-miR-370-3p	1	34	1820
circRNA320	oar-miR-376a-5p	1	31	1980
circRNA568	oar-miR-655-3p	1	166	4830
circRNA198	oar-miR-25	1	52	2760
circRNA255	oar-miR-370-3p	1	63	4490
circRNA2	oar-miR-323a-5p	1	128	3720
circRNA776	oar-let-7c	1	27	331
circRNA782	oar-miR-493-3p	1	17	828
circRNA438	oar-miR-431	1	33	1310
circRNA314	oar-miR-30b	1	23	1150
circRNA59	oar-miR-134-3p	1	73	3560
circRNA301	oar-miR-23a	1	111	6260
circRNA96	oar-miR-370-3p	1	44	3060
circRNA595	oar-miR-299-3p	1	12	672
circRNA582	oar-miR-376c-3p	1	39	1480
circRNA337	oar-miR-30d	1	529	30300
circRNA568	oar-miR-218a	1	166	4830
circRNA734	oar-miR-329b-5p	1	37	1920
circRNA60	oar-miR-329a-3p	1	66	3680
circRNA208	oar-miR-495-5p	1	71	4430
circRNA454	oar-miR-544-3p	1	17	1530
circRNA578	oar-miR-410-5p	1	8	334
circRNA221	oar-let-7d	1	74	2240
circRNA533	oar-miR-411a-3p	1	53	1980
ciRNA30	oar-miR-26a	1	2	84.4
circRNA159	oar-miR-382-5p	1	32	2070
circRNA467	oar-miR-380-3p	1	28	1050
circRNA338	oar-miR-16b	1	45.5	2180
circRNA764	oar-miR-379-5p	1	159	6640
circRNA104	oar-miR-379-3p	1	14	856
circRNA490	oar-miR-654-3p	1	240	5160
circRNA776	oar-miR-432	1	27	331
circRNA592	oar-miR-3959-3p	1	68	1670
circRNA460	oar-miR-143	1	249	6760
circRNA635	oar-miR-199a-3p	1	59	2650
circRNA628	oar-miR-370-5p	1	58	2150
circRNA337	oar-miR-30c	1	529	30300
ciRNA56	oar-miR-1197-5p	1	15	1070
circRNA95	oar-miR-376d	1	28	1150
circRNA542	oar-miR-494-5p	1	16	715
circRNA369	oar-miR-409-5p	1	33	1560
circRNA251	oar-miR-487a-3p	1	53	1050
circRNA700	oar-miR-668-5p	1	33	2440
circRNA590	oar-let-7d	1	81	5580
circRNA581	oar-miR-539-3p	1	14	835
circRNA533	oar-let-7i	1	53	1980
circRNA665	oar-miR-411b-3p	1	42	1920
circRNA784	oar-miR-654-5p	1	1260	41800
circRNA112	oar-miR-665-5p	1	112	4570
circRNA562	oar-miR-376e-5p	1	4	368
circRNA752	oar-let-7f	1	21	926
circRNA565	oar-miR-409-3p	1	45	1450
circRNA567	oar-miR-21	1	64.5	2650
circRNA46	oar-miR-433-3p	1	22	1190
circRNA345	oar-miR-495-5p	1	32	1330
circRNA104	oar-miR-409-3p	1	14	856
ciRNA37	oar-miR-22-3p	1	22	666
circRNA559	oar-miR-106b	1	18	1220
circRNA220	oar-miR-432	1	17	894
ciRNA20	oar-let-7g	1	11	319
circRNA427	oar-miR-544-3p	1	19	551
ciRNA26	oar-miR-541-3p	1	7	715
ciRNA75	oar-miR-136	1	3	118
circRNA328	oar-miR-10b	1	16	1060
ciRNA32	oar-miR-410-5p	1	91.5	7530
circRNA224	oar-miR-16b	1	37	1600
circRNA173	oar-miR-127	1	38	1350
circRNA727	oar-miR-27a	1	82	4350
circRNA46	oar-miR-410-5p	1	22	1190
circRNA277	oar-miR-493-5p	1	971	48700
circRNA673	oar-miR-154b-3p	1	78	4010
ciRNA44	oar-miR-329b-3p	1	4	227
ciRNA49	oar-miR-379-3p	1	6	449
circRNA8	oar-let-7a	1	85	3450
circRNA479	oar-miR-382-5p	1	94	4200
circRNA476	oar-miR-493-3p	1	81	2660
circRNA415	oar-miR-26a	1	49	1620
circRNA63	oar-miR-194	1	49	2720
circRNA230	oar-let-7g	1	12	602
circRNA307	oar-miR-758-3p	1	341	21400
circRNA253	oar-miR-1193-5p	1	50	1750
circRNA582	oar-miR-103	1	39	1480
circRNA778	oar-miR-432	1	24	1210

circRNA342	oar-miR-493-3p	1	45	2070
circRNA676	oar-miR-380-5p	1	6	537
circRNA374	oar-miR-218a	1	33	1050
circRNA391	oar-let-7i	1	19	358
circRNA261	oar-miR-495-5p	1	384	11400
circRNA310	oar-miR-433-3p	1	150	3620
circRNA312	oar-miR-412-5p	1	190	3850
circRNA48	oar-miR-655-5p	1	37	992
ciRNA98	oar-miR-668-3p	1	6	411
circRNA395	oar-miR-381-5p	1	31	1450
circRNA93	oar-miR-323a-5p	1	291	8600
circRNA391	oar-miR-329a-3p	1	19	358
circRNA94	oar-miR-654-3p	1	89	6150
circRNA397	oar-miR-136	1	54	2570
circRNA599	oar-miR-541-5p	1	6	298
circRNA637	oar-miR-329b-5p	1	120	2400
circRNA577	oar-miR-1185-5p	1	60	3480
circRNA668	oar-miR-494-5p	1	30	1850
circRNA229	oar-miR-496-5p	1	48	845
circRNA776	oar-miR-668-5p	1	27	331
circRNA610	oar-miR-154b-5p	1	83	2110
circRNA250	oar-miR-412-5p	1	14	743
circRNA568	oar-miR-30d	1	166	4830
circRNA166	oar-miR-409-5p	1	171	4920
circRNA167	oar-miR-411b-5p	1	14	900
circRNA427	oar-miR-17-5p	1	19	551
circRNA766	oar-miR-3957-5p	1	86.2	2720
circRNA486	oar-miR-433-3p	1	13	615
circRNA539	oar-let-7b	1	29	1360
circRNA771	oar-miR-3959-5p	1	30	1530
circRNA775	oar-let-7i	1	17	410
circRNA757	oar-miR-495-5p	1	190	5460
circRNA492	oar-let-7a	1	157	7210
circRNA78	oar-miR-136	1	47	1420
circRNA50	oar-miR-370-5p	1	41	968
circRNA634	oar-let-7f	1	20	1240
circRNA726	oar-miR-412-5p	1	64	4520
circRNA252	oar-let-7a	1	612	11700
circRNA378	oar-miR-1193-5p	1	42.5	1830
circRNA319	oar-let-7f	1	42	1620
circRNA772	oar-let-7f	1	34	1290
circRNA687	oar-miR-412-3p	1	25	925
circRNA649	oar-miR-654-5p	1	5.5	363
circRNA305	oar-miR-3956-3p	1	52	2410
circRNA45	oar-miR-432	1	39	1110
circRNA607	oar-miR-3957-5p	1	10	722
circRNA115	oar-miR-199a-3p	1	280	9430
circRNA499	oar-miR-1193-3p	1	15	581
circRNA429	oar-miR-496-5p	1	121	3760
circRNA275	oar-miR-668-5p	1	165	2850
circRNA640	oar-miR-376a-3p	1	59	2230
circRNA145	oar-miR-154b-5p	1	79	3280
circRNA11	oar-let-7g	1	11	576
circRNA278	oar-miR-543-3p	1	65	2960
circRNA463	oar-miR-411b-5p	1	40	2200
circRNA439	oar-miR-154b-5p	1	146	3240
circRNA416	oar-miR-380-5p	1	28	1680
ciRNA89	oar-miR-1197-5p	1	48	4440
circRNA54	oar-miR-668-5p	1	52	1810
circRNA105	oar-miR-380-5p	1	22	896
circRNA349	oar-miR-136	1	11	694
circRNA109	oar-miR-19b	1	42	2010
circRNA781	oar-miR-125b	1	8	399
circRNA504	oar-let-7i	1	123	5780
circRNA153	oar-miR-1185-5p	1	19	1110
circRNA587	oar-miR-409-5p	1	24	1480
circRNA6	oar-miR-493-3p	1	67	3600
circRNA20	oar-miR-370-3p	1	86	2300
ciRNA45	oar-miR-134-3p	1	4	312
circRNA299	oar-miR-200a	1	8	590
circRNA640	oar-let-7d	1	59	2230
circRNA553	oar-miR-654-3p	1	37	974
circRNA386	oar-miR-370-3p	1	102	2110
circRNA523	oar-miR-1197-3p	1	69.5	3180
ciRNA47	oar-miR-23b	1	2	156
circRNA526	oar-miR-200c	1	40	1900
circRNA729	oar-miR-543-5p	1	181	12000
circRNA252	oar-miR-3956-3p	1	612	11700
circRNA486	oar-miR-194	1	13	615
circRNA386	oar-miR-299-3p	1	102	2110
circRNA555	oar-miR-487b-5p	1	36	1680
circRNA690	oar-miR-200a	1	4	284
circRNA172	oar-miR-127	1	21	847
circRNA595	oar-miR-106a	1	12	672
circRNA668	oar-miR-329b-5p	1	30	1850
circRNA100	oar-miR-329b-5p	1	20	767
circRNA215	oar-miR-16b	1	11	948
circRNA157	oar-miR-199a-3p	1	268	16300
circRNA488	oar-miR-379-5p	1	37	1330
circRNA304	oar-miR-154b-3p	1	262	7540
circRNA266	oar-miR-16b	1	95	5370
circRNA136	oar-miR-487a-5p	1	24	1000
circRNA741	oar-miR-221	1	89	2700
circRNA42	oar-miR-495-5p	1	193	3260
circRNA149	oar-let-7g	1	71	3740
circRNA539	oar-miR-23a	1	29	1360
circRNA677	oar-miR-432	1	238	8090
circRNA35	oar-miR-133	1	34	1030

circRNA563	oar-miR-3956-3p	1	106	2740
circRNA642	oar-miR-27a	1	22	950
circRNA87	oar-miR-1193-3p	1	22	1330
circRNA438	oar-miR-543-5p	1	33	1310
circRNA770	oar-miR-376b-3p	1	7	452
circRNA778	oar-miR-382-3p	1	24	1210
circRNA459	oar-miR-30b	1	31	1450
circRNA24	oar-let-7a	1	34	2370
circRNA383	oar-miR-136	1	11	479
circRNA687	oar-let-7g	1	25	925
circRNA341	oar-let-7c	1	107	4040
circRNA192	oar-miR-493-3p	1	104	3910
circRNA340	oar-miR-382-5p	1	22	1500
circRNA482	oar-miR-329a-5p	1	275	8290
circRNA682	oar-miR-1197-5p	1	16	831
circRNA41	oar-miR-362	1	9	617
circRNA588	oar-miR-376e-5p	1	20	1250
circRNA410	oar-miR-3957-3p	1	160	4560
circRNA386	oar-miR-29b	1	102	2110
circRNA717	oar-miR-136	1	61.5	3250
circRNA412	oar-miR-329b-3p	1	28.2	1620
circRNA209	oar-miR-133	1	2	97.7
circRNA533	oar-miR-1197-5p	1	53	1980
circRNA685	oar-miR-541-3p	1	172	6290
circRNA106	oar-miR-1197-5p	1	60	2410
circRNA382	oar-miR-376c-5p	1	26	984
circRNA39	oar-let-7b	1	11	513
circRNA641	oar-miR-23a	1	14	853
circRNA772	oar-miR-665-5p	1	34	1290
circRNA373	oar-miR-3955-3p	1	44	2350
circRNA589	oar-miR-543-5p	1	11	260
circRNA41	oar-miR-125b	1	9	617
circRNA687	oar-let-7b	1	25	925
ciRNA37	oar-miR-370-5p	1	22	666
circRNA391	oar-let-7b	1	19	358
circRNA314	oar-miR-181a	1	23	1150
circRNA447	oar-miR-134-5p	1	48	1760
circRNA337	oar-miR-376b-3p	1	529	30300
circRNA778	oar-miR-544-3p	1	24	1210
circRNA570	oar-miR-485-3p	1	121	6150
circRNA111	oar-miR-370-3p	1	181	4380
circRNA765	oar-miR-379-5p	1	15	760
circRNA457	oar-miR-376a-5p	1	146	7240
circRNA575	oar-miR-376c-5p	1	76	3630
circRNA446	oar-miR-665-5p	1	38	1460
ciRNA3	oar-miR-362	1	6	543
circRNA435	oar-miR-412-3p	1	20.2	602
circRNA661	oar-miR-379-5p	1	338	17700
circRNA502	oar-miR-409-5p	1	17	975
circRNA200	oar-miR-127	1	73	2220
circRNA377	oar-miR-494-3p	1	124	3890
circRNA568	oar-miR-487a-5p	1	166	4830
circRNA628	oar-miR-377-5p	1	58	2150
circRNA618	oar-miR-410-5p	1	81	4230
circRNA55	oar-miR-381-5p	1	23	1460
circRNA364	oar-miR-493-3p	1	140	6320
circRNA458	oar-miR-544-5p	1	9	549
circRNA592	oar-miR-544-3p	1	68	1670
circRNA614	oar-miR-3957-3p	1	50	1590
circRNA323	oar-miR-541-5p	1	8	555
circRNA610	oar-miR-541-5p	1	83	2110
circRNA10	oar-miR-494-3p	1	10	483
circRNA425	oar-miR-376c-5p	1	36	1090
circRNA175	oar-miR-200a	1	9	475
ciRNA60	oar-miR-411b-3p	1	18	809
circRNA170	oar-miR-200a	1	74	1320
circRNA225	oar-miR-379-5p	1	29	1590
circRNA693	oar-miR-431	1	27	842
circRNA218	oar-miR-1185-5p	1	71	4670
circRNA693	oar-miR-495-5p	1	27	842
circRNA564	oar-miR-134-3p	1	28	1490
circRNA154	oar-miR-133	1	27	1160
circRNA548	oar-miR-668-3p	1	13	964
circRNA159	oar-miR-495-5p	1	32	2070
circRNA353	oar-miR-3956-3p	1	21	1130
circRNA445	oar-miR-181a	1	17	958
circRNA524	oar-miR-1197-3p	1	16.5	1190
circRNA121	oar-miR-411a-5p	1	633	14600
circRNA247	oar-miR-485-5p	1	41	1110
circRNA606	oar-miR-299-3p	1	95	3890
ciRNA68	oar-miR-496-5p	1	5	376
circRNA126	oar-miR-329b-5p	1	19	1280
circRNA693	oar-miR-21	1	27	842
circRNA479	oar-miR-411b-3p	1	94	4200
circRNA491	oar-miR-409-3p	1	34	1470
circRNA67	oar-miR-136	1	26	1430
circRNA776	oar-let-7i	1	27	331
circRNA398	oar-let-7g	1	135	4930
circRNA511	oar-miR-3955-5p	1	59	3000
circRNA395	oar-miR-485-5p	1	31	1450
circRNA331	oar-miR-655-5p	1	32	2510
circRNA394	oar-miR-433-3p	1	23	1490
circRNA553	oar-miR-27a	1	37	974
circRNA706	oar-miR-3955-3p	1	40	938
circRNA334	oar-miR-1193-5p	1	176	11100
circRNA477	oar-miR-382-5p	1	260	6440
circRNA415	oar-miR-541-3p	1	49	1620
circRNA272	oar-miR-29a	1	67	969

circRNA150	oar-miR-376a-5p	1	55	4530
circRNA774	oar-miR-200c	1	63	3650
circRNA391	oar-let-7c	1	19	358
circRNA142	oar-miR-99a	1	76	5370
circRNA209	oar-miR-665-3p	1	2	97.7
circRNA310	oar-miR-200c	1	150	3620
circRNA391	oar-miR-379-3p	1	19	358
circRNA389	oar-miR-323a-5p	1	20	1470
circRNA714	oar-miR-22-3p	1	23	1190
circRNA202	oar-miR-3957-3p	1	10	547
circRNA1	oar-miR-1197-3p	1	10	605
circRNA602	oar-miR-200c	1	197	11100
circRNA78	oar-miR-376e-5p	1	47	1420
circRNA113	oar-miR-29b	1	52	3060
circRNA744	oar-miR-543-3p	1	56	1790
circRNA686	oar-miR-410-5p	1	30	923
circRNA773	oar-miR-409-5p	1	25	1110
circRNA346	oar-miR-133	1	15	570
circRNA8	oar-miR-323a-3p	1	85	3450
circRNA641	oar-let-7i	1	14	853
circRNA599	oar-miR-29a	1	6	298
ciRNA60	oar-miR-323a-5p	1	18	809
circRNA782	oar-miR-99a	1	17	828
circRNA183	oar-miR-148a	1	189	9040
circRNA763	oar-miR-329a-5p	1	337	17400
circRNA82	oar-miR-493-3p	1	81	5460
circRNA532	oar-miR-758-5p	1	731	18000
circRNA549	oar-miR-668-3p	1	26	1570
circRNA192	oar-miR-30a-3p	1	104	3910
circRNA707	oar-let-7d	1	71	2950
circRNA15	oar-miR-154a-3p	1	21	1030
circRNA775	oar-let-7c	1	17	410
circRNA435	oar-miR-323a-3p	1	20.2	602
circRNA500	oar-let-7a	1	45	1680
circRNA511	oar-miR-432	1	59	3000
circRNA544	oar-miR-107	1	58.5	2700
circRNA682	oar-miR-30a-5p	1	16	831
circRNA352	oar-miR-148a	1	45	2060
circRNA303	oar-miR-487a-5p	1	39.5	2770
circRNA305	oar-miR-107	1	52	2410
circRNA687	oar-let-7a	1	25	925
circRNA459	oar-miR-148a	1	31	1450
circRNA328	oar-miR-411a-5p	1	16	1060
circRNA338	oar-miR-544-3p	1	45.5	2180
ciRNA54	oar-miR-541-3p	1	22	1530
circRNA310	oar-miR-485-5p	1	150	3620
circRNA317	oar-let-7g	1	223	11200
circRNA51	oar-miR-412-3p	1	20	664
circRNA154	oar-let-7i	1	27	1160
circRNA90	oar-miR-433-5p	1	239	12600
circRNA633	oar-miR-431	1	28	1440
circRNA116	oar-miR-758-5p	1	342	14200
circRNA105	oar-miR-3957-3p	1	22	896
circRNA492	oar-miR-381-5p	1	157	7210
circRNA717	oar-let-7i	1	61.5	3250
circRNA631	oar-miR-329b-3p	1	379	9240
circRNA720	oar-miR-154b-5p	1	1018	57400
circRNA692	oar-miR-3955-5p	1	24	831
circRNA310	oar-miR-299-3p	1	150	3620
circRNA141	oar-miR-218a	1	60	3370
circRNA370	oar-let-7b	1	39	1490
circRNA716	oar-miR-370-5p	1	83.5	2960
circRNA59	oar-miR-411b-3p	1	73	3560
circRNA535	oar-miR-134-5p	1	17	769
circRNA629	oar-miR-26a	1	57	1900
circRNA333	oar-miR-544-3p	1	187	12200
circRNA305	oar-miR-21	1	52	2410
circRNA56	oar-miR-134-3p	1	61	4870
circRNA613	oar-miR-103	1	21	735
circRNA636	oar-miR-665-5p	1	49	2080
circRNA542	oar-miR-379-5p	1	16	715
circRNA581	oar-miR-154b-3p	1	14	835
ciRNA82	oar-miR-412-5p	1	2	137
circRNA67	oar-miR-376e-3p	1	26	1430
circRNA194	oar-miR-134-3p	1	65	3740
circRNA539	oar-miR-30d	1	29	1360
circRNA122	oar-miR-1197-5p	1	177	5560
circRNA183	oar-miR-544-3p	1	189	9040
circRNA236	oar-miR-654-5p	1	181	4960
circRNA372	oar-miR-299-3p	1	30	1200
circRNA342	oar-miR-30c	1	45	2070
circRNA425	oar-miR-134-3p	1	36	1090
circRNA202	oar-miR-221	1	10	547
circRNA304	oar-miR-3957-3p	1	262	7540
circRNA426	oar-miR-433-3p	1	5	383
circRNA162	oar-miR-539-3p	1	8	320
circRNA635	oar-miR-493-3p	1	59	2650
circRNA298	oar-miR-412-3p	1	28	774
circRNA706	oar-miR-411b-3p	1	40	938
circRNA35	oar-miR-19b	1	34	1030
circRNA51	oar-miR-299-5p	1	20	664
circRNA734	oar-miR-544-5p	1	37	1920
circRNA425	oar-miR-22-3p	1	36	1090
circRNA272	oar-miR-200c	1	67	969
circRNA107	oar-miR-487b-3p	1	96	4820
circRNA246	oar-miR-3957-5p	1	53	1940
circRNA444	oar-miR-362	1	29	1310
circRNA137	oar-miR-412-3p	1	24	849

circRNA334	oar-let-7f	1	176	11100
circRNA495	oar-miR-362	1	134	5930
circRNA441	oar-miR-26b	1	21	626
circRNA352	oar-miR-107	1	45	2060
circRNA279	oar-miR-495-5p	1	71	3110
circRNA436	oar-miR-655-3p	1	123	2820
circRNA62	oar-miR-3959-3p	1	146	7900
circRNA209	oar-miR-412-5p	1	2	97.7
circRNA48	oar-miR-379-5p	1	37	992
circRNA495	oar-miR-495-5p	1	134	5930
circRNA265	oar-miR-485-5p	1	15	215
ciRNA51	oar-miR-1193-5p	1	10	635
circRNA19	oar-miR-148a	1	22	1010
circRNA42	oar-miR-409-3p	1	193	3260
circRNA14	oar-miR-485-3p	1	99	3350
circRNA223	oar-miR-485-5p	1	34	1820
circRNA569	oar-miR-3955-5p	1	175	6360
circRNA414	oar-miR-194	1	10	461
circRNA455	oar-miR-329a-5p	1	20	994
circRNA251	oar-miR-154a-5p	1	53	1050
circRNA685	oar-miR-412-5p	1	172	6290
circRNA788	oar-miR-544-3p	1	400	16300
circRNA29	oar-miR-329b-5p	1	53	1680
circRNA5	oar-miR-106a	1	23	942
circRNA776	oar-miR-27a	1	27	331
circRNA341	oar-let-7i	1	107	4040
circRNA241	oar-let-7c	1	45	2440
circRNA375	oar-miR-299-3p	1	16	1010
circRNA356	oar-let-7i	1	152	6210
circRNA570	oar-miR-154b-5p	1	121	6150
circRNA241	oar-let-7d	1	45	2440
ciRNA54	oar-miR-412-3p	1	22	1530
circRNA744	oar-miR-411b-5p	1	56	1790
ciRNA88	oar-miR-485-5p	1	8	668
circRNA507	oar-miR-369-5p	1	166	4910
circRNA207	oar-miR-654-5p	1	45	1600
circRNA247	oar-miR-200a	1	41	1110
circRNA124	oar-miR-199a-3p	1	157	5240
circRNA506	oar-miR-377-5p	1	29	1530
circRNA250	oar-miR-369-5p	1	14	743
ciRNA91	oar-miR-1193-3p	1	1	109
circRNA715	oar-miR-154b-3p	1	64	2040
circRNA336	oar-miR-543-5p	1	24	1370
circRNA602	oar-miR-544-3p	1	197	11100
circRNA337	oar-miR-30a-5p	1	529	30300
circRNA480	oar-miR-758-3p	1	9	338
circRNA294	oar-miR-370-5p	1	158	8020
circRNA526	oar-miR-154b-5p	1	40	1900
circRNA538	oar-miR-487a-5p	1	41	1830
circRNA706	oar-miR-191	1	40	938
circRNA717	oar-miR-493-3p	1	61.5	3250
circRNA757	oar-miR-411b-5p	1	190	5460
ciRNA12	oar-miR-376c-3p	1	7	488
ciRNA5	oar-miR-496-3p	1	6	305
circRNA763	oar-miR-3959-3p	1	337	17400
circRNA685	oar-miR-665-5p	1	172	6290
circRNA585	oar-miR-134-5p	1	24	1130
circRNA537	oar-miR-377-5p	1	37	2640
circRNA106	oar-miR-412-3p	1	60	2410
circRNA15	oar-miR-485-3p	1	21	1030
circRNA554	oar-miR-376c-5p	1	10	835
circRNA213	oar-miR-154b-5p	1	97	6070
circRNA511	oar-miR-200c	1	59	3000
circRNA374	oar-miR-654-3p	1	33	1050
circRNA370	oar-miR-376e-3p	1	39	1490
circRNA266	oar-miR-107	1	95	5370
circRNA265	oar-miR-181a	1	15	215
circRNA753	oar-miR-495-3p	1	109	3710
circRNA211	oar-miR-362	1	16	820
circRNA191	oar-miR-134-3p	1	89	4480
circRNA370	oar-let-7f	1	39	1490
circRNA540	oar-miR-218a	1	87	3030
circRNA585	oar-miR-485-5p	1	24	1130
circRNA439	oar-miR-136	1	146	3240
circRNA732	oar-miR-380-5p	1	16	538
circRNA753	oar-miR-329b-5p	1	109	3710
circRNA488	oar-miR-134-5p	1	37	1330
circRNA703	oar-miR-1197-3p	1	3	248
circRNA528	oar-miR-411b-5p	1	93	2490
circRNA606	oar-miR-27a	1	95	3890
circRNA318	oar-miR-1197-3p	1	55	2410
circRNA686	oar-miR-758-5p	1	30	923
circRNA609	oar-miR-411b-5p	1	49	1860
circRNA460	oar-miR-3955-5p	1	249	6760
circRNA170	oar-miR-487b-5p	1	74	1320
circRNA169	oar-let-7f	1	24	1170
circRNA44	oar-let-7d	1	61	1930
circRNA759	oar-miR-362	1	46	2440
circRNA80	oar-miR-29b	1	46	1940
circRNA103	oar-let-7i	1	147	2290
circRNA36	oar-miR-433-3p	1	100	2650
circRNA189	oar-miR-27a	1	3	343
circRNA639	oar-miR-23b	1	71	3330
circRNA341	oar-miR-1197-5p	1	107	4040
circRNA553	oar-miR-329a-5p	1	37	974
circRNA651	oar-miR-376a-3p	1	78	1620
circRNA53	oar-miR-544-3p	1	61	3980
circRNA758	oar-miR-382-3p	1	69	2410

circRNA567	oar-miR-543-3p	1	64.5	2650
circRNA114	oar-miR-539-3p	1	350	14700
circRNA475	oar-miR-154b-3p	1	16	858
circRNA555	oar-miR-19b	1	36	1680
circRNA358	oar-miR-133	1	56	3900
ciRNA37	oar-miR-376a-5p	1	22	666
circRNA322	oar-miR-133	1	130	4390
circRNA181	oar-let-7g	1	28	916
circRNA287	oar-miR-369-5p	1	6	215
ciRNA73	oar-miR-3957-3p	1	8	675
circRNA459	oar-miR-758-5p	1	31	1450
circRNA477	oar-miR-3955-3p	1	260	6440
circRNA455	oar-miR-329b-5p	1	20	994
circRNA568	oar-miR-194	1	166	4830
circRNA765	oar-let-7i	1	15	760
circRNA236	oar-miR-485-5p	1	181	4960
circRNA432	oar-miR-125b	1	268	1640
circRNA351	oar-miR-1197-5p	1	23	1580
circRNA6	oar-miR-432	1	67	3600
circRNA507	oar-miR-3958-5p	1	166	4910
circRNA7	oar-miR-487a-3p	1	47	1830
circRNA742	oar-miR-433-5p	1	63	2210
circRNA748	oar-miR-150	1	22	1180
circRNA44	oar-miR-433-3p	1	61	1930
circRNA777	oar-miR-3958-5p	1	10	409
circRNA169	oar-miR-381-3p	1	24	1170
circRNA52	oar-miR-3958-5p	1	14	313
circRNA106	oar-miR-654-5p	1	60	2410
circRNA693	oar-miR-134-3p	1	27	842
circRNA42	oar-miR-370-5p	1	193	3260
circRNA636	oar-let-7c	1	49	2080
circRNA339	oar-miR-200a	1	9.5	677
circRNA334	oar-miR-370-3p	1	176	11100
circRNA493	oar-miR-758-3p	1	20.5	1330
circRNA510	oar-miR-487b-5p	1	34	2450
ciRNA75	oar-miR-26b	1	3	118
circRNA281	oar-let-7f	1	17	594
circRNA490	oar-miR-1197-5p	1	240	5160
circRNA636	oar-miR-154b-5p	1	49	2080
circRNA48	oar-miR-10a	1	37	992
circRNA739	oar-miR-411b-5p	1	173	6440
circRNA704	oar-miR-412-5p	1	49	2550
circRNA171	oar-miR-495-5p	1	166	8060
circRNA51	oar-miR-21	1	20	664
circRNA321	oar-miR-382-5p	1	48	2450
ciRNA36	oar-miR-370-5p	1	17	1010
circRNA249	oar-miR-181a	1	357	11500
circRNA575	oar-miR-432	1	76	3630
circRNA318	oar-miR-432	1	55	2410
circRNA553	oar-miR-410-5p	1	37	974
circRNA782	oar-miR-154b-5p	1	17	828
circRNA68	oar-miR-329b-5p	1	5	305
circRNA220	oar-miR-19b	1	17	894
circRNA34	oar-miR-379-5p	1	21	827
circRNA582	oar-miR-1185-3p	1	39	1480
circRNA412	oar-miR-654-5p	1	28.2	1620
circRNA425	oar-miR-410-5p	1	36	1090
circRNA233	oar-miR-539-5p	1	90	5650
circRNA199	oar-miR-29b	1	14	653
circRNA292	oar-miR-376c-3p	1	13	484
circRNA300	oar-miR-544-5p	1	142	5630
circRNA595	oar-miR-200c	1	12	672
ciRNA37	oar-miR-134-3p	1	22	666
circRNA441	oar-miR-199a-3p	1	21	626
circRNA275	oar-miR-494-3p	1	165	2850
circRNA447	oar-miR-10b	1	48	1760
circRNA764	oar-miR-412-3p	1	159	6640
circRNA763	oar-miR-134-3p	1	337	17400
circRNA641	oar-let-7c	1	14	853
circRNA193	oar-miR-323a-5p	1	413	23600
circRNA433	oar-miR-758-3p	1	213	1440
circRNA705	oar-miR-154b-3p	1	20	657
circRNA31	oar-miR-1193-3p	1	102	1620
circRNA160	oar-miR-654-5p	1	31	1750
circRNA322	oar-let-7i	1	130	4390
circRNA488	oar-miR-199a-3p	1	37	1330
circRNA616	oar-miR-136	1	84	5180
circRNA353	oar-miR-17-5p	1	21	1130
circRNA290	oar-miR-154b-3p	1	75	4460
circRNA784	oar-miR-329a-5p	1	1260	41800
ciRNA20	oar-miR-99a	1	11	319
circRNA182	oar-miR-152	1	192	6140
circRNA168	oar-miR-3958-3p	1	45	1730
circRNA245	oar-miR-30a-5p	1	26	1730
circRNA528	oar-miR-125b	1	93	2490
circRNA743	oar-miR-3957-5p	1	291	8460
circRNA712	oar-miR-376a-5p	1	120	5180
circRNA733	oar-miR-433-5p	1	22	1110
circRNA510	oar-miR-668-3p	1	34	2450
circRNA216	oar-miR-541-3p	1	32	925
ciRNA4	oar-miR-411a-5p	1	2	215
ciRNA50	oar-miR-377-5p	1	36	3220
circRNA488	oar-miR-493-3p	1	37	1330
circRNA788	oar-miR-152	1	400	16300
circRNA420	oar-let-7a	1	9	705
circRNA469	oar-miR-23a	1	46	2790
circRNA147	oar-miR-380-3p	1	12	556
circRNA676	oar-miR-431	1	6	537

circRNA353	oar-miR-370-3p	1	21	1130
circRNA335	oar-miR-370-3p	1	34	2110
circRNA78	oar-miR-376b-5p	1	47	1420
ciRNA10	oar-let-7i	1	40	2950
circRNA97	oar-let-7b	1	22	1350
circRNA285	oar-miR-487a-5p	1	23	991
ciRNA83	oar-miR-3958-3p	1	8	572
circRNA519	oar-miR-370-5p	1	18	1590
circRNA496	oar-let-7i	1	90.5	3120
circRNA756	oar-miR-665-5p	1	68	2660
ciRNA92	oar-let-7c	1	7	562
circRNA548	oar-miR-382-5p	1	13	964
circRNA430	oar-miR-668-3p	1	319	2550
ciRNA37	oar-miR-377-5p	1	22	666
circRNA520	oar-miR-134-3p	1	8	740
circRNA353	oar-miR-382-3p	1	21	1130
circRNA285	oar-miR-134-5p	1	23	991
circRNA386	oar-miR-3957-3p	1	102	2110
circRNA417	oar-miR-134-5p	1	61	2420
circRNA284	oar-miR-543-3p	1	18	788
circRNA206	oar-miR-541-3p	1	20	1050
circRNA262	oar-miR-136	1	24	975
circRNA295	oar-miR-544-5p	1	485	13200
circRNA453	oar-miR-329a-5p	1	13	374
circRNA589	oar-let-7c	1	11	260
ciRNA67	oar-miR-27a	1	6	537
circRNA425	oar-let-7g	1	36	1090
ciRNA51	oar-miR-665-5p	1	10	635
circRNA354	oar-miR-154a-3p	1	142	3660
ciRNA43	oar-miR-410-5p	1	1	91.4
circRNA30	oar-let-7c	1	62	4220
circRNA666	oar-miR-370-5p	1	17	1060
circRNA770	oar-miR-376d	1	7	452
circRNA782	oar-miR-411a-5p	1	17	828
circRNA529	oar-miR-431	1	50	1940
circRNA14	oar-let-7d	1	99	3350
circRNA509	oar-miR-221	1	88	3130
ciRNA54	oar-miR-411b-5p	1	22	1530
circRNA289	oar-miR-493-3p	1	19	965
circRNA592	oar-miR-221	1	68	1670
circRNA432	oar-miR-758-3p	1	268	1640
circRNA622	oar-miR-103	1	93	5000
circRNA323	oar-let-7c	1	8	555
circRNA610	oar-miR-3956-5p	1	83	2110
circRNA706	oar-miR-487a-5p	1	40	938
circRNA112	oar-miR-29b	1	112	4570
circRNA372	oar-miR-323a-5p	1	30	1200
circRNA52	oar-miR-299-3p	1	14	313
circRNA154	oar-miR-665-5p	1	27	1160
circRNA622	oar-miR-3955-5p	1	93	5000
circRNA423	oar-miR-134-5p	1	17	1070
circRNA423	oar-miR-200b	1	17	1070
circRNA493	oar-miR-381-5p	1	20.5	1330
circRNA445	oar-miR-493-3p	1	17	958
circRNA67	oar-miR-181a	1	26	1430
circRNA104	oar-miR-134-5p	1	14	856
circRNA406	oar-miR-665-3p	1	148	5360
circRNA738	oar-miR-485-5p	1	211	12700
circRNA108	oar-miR-668-3p	1	94	2680
circRNA59	oar-miR-3959-3p	1	73	3560
circRNA263	oar-miR-376a-5p	1	17	1240
circRNA620	oar-miR-136	1	75	2640
circRNA50	oar-miR-1193-3p	1	41	968
circRNA385	oar-miR-29b	1	10	1080
circRNA583	oar-miR-543-5p	1	44	2100
circRNA214	oar-miR-377-3p	1	195	10000
circRNA297	oar-miR-134-3p	1	12	698
circRNA82	oar-miR-412-5p	1	81	5460
circRNA11	oar-miR-154b-5p	1	11	576
circRNA768	oar-miR-10a	1	43.7	1850
circRNA776	oar-miR-410-5p	1	27	331
circRNA567	oar-miR-544-3p	1	64.5	2650
circRNA662	oar-let-7i	1	63	1980
circRNA634	oar-let-7g	1	20	1240
circRNA776	oar-miR-10b	1	27	331
circRNA299	oar-miR-433-5p	1	8	590
circRNA589	oar-miR-379-5p	1	11	260
circRNA17	oar-miR-134-5p	1	62	2020
circRNA398	oar-miR-1185-5p	1	135	4930
circRNA292	oar-miR-655-5p	1	13	484
circRNA129	oar-miR-362	1	58	2800
circRNA391	oar-miR-493-3p	1	19	358
ciRNA93	oar-miR-329b-5p	1	3	254
circRNA694	oar-miR-362	1	109	3640
circRNA302	oar-miR-370-3p	1	158	3480
circRNA171	oar-miR-665-3p	1	166	8060
circRNA276	oar-miR-1197-5p	1	8	332
circRNA409	oar-miR-411b-5p	1	26	1130
circRNA521	oar-let-7f	1	49	3400
circRNA81	oar-miR-30a-3p	1	19	1280
circRNA247	oar-miR-376a-5p	1	41	1110
circRNA786	oar-miR-1193-3p	1	26	1520
circRNA307	oar-miR-495-5p	1	341	21400
circRNA364	oar-miR-541-3p	1	140	6320
circRNA294	oar-miR-181a	1	158	8020
circRNA521	oar-let-7d	1	49	3400
circRNA117	oar-miR-655-5p	1	5	521
circRNA708	oar-miR-103	1	141	7610

circRNA668	oar-miR-362	1	30	1850
circRNA544	oar-miR-376c-3p	1	58.5	2700
circRNA111	oar-miR-134-3p	1	181	4380
circRNA532	oar-miR-200b	1	731	18000
circRNA721	oar-miR-493-5p	1	174	9120
circRNA383	oar-miR-654-5p	1	11	479
circRNA707	oar-let-7c	1	71	2950
circRNA65	oar-miR-199a-3p	1	98	6480
circRNA619	oar-miR-362	1	46	3000
circRNA79	oar-miR-412-3p	1	22	1320
circRNA635	oar-miR-432	1	59	2650
circRNA363	oar-miR-541-3p	1	40.5	3530
circRNA226	oar-miR-654-3p	1	13	1170
circRNA659	oar-miR-655-5p	1	125	6640
circRNA539	oar-miR-665-5p	1	29	1360
circRNA446	oar-miR-323b	1	38	1460
ciRNA41	oar-miR-665-5p	1	7	551
circRNA712	oar-let-7d	1	120	5180
circRNA61	oar-miR-30a-3p	1	17	681
ciRNA51	oar-miR-410-5p	1	10	635
ciRNA60	oar-miR-134-5p	1	18	809
circRNA375	oar-let-7g	1	16	1010
circRNA20	oar-miR-494-5p	1	86	2300
ciRNA65	oar-miR-150	1	8	607
circRNA265	oar-miR-30b	1	15	215
circRNA160	oar-miR-431	1	31	1750
circRNA229	oar-miR-152	1	48	845
circRNA324	oar-miR-181a	1	59	1900
circRNA765	oar-miR-758-3p	1	15	760
circRNA532	oar-miR-380-5p	1	731	18000
circRNA317	oar-miR-377-5p	1	223	11200
circRNA567	oar-miR-369-3p	1	64.5	2650
circRNA360	oar-let-7a	1	14	1030
circRNA522	oar-miR-148a	1	39	1470
circRNA776	oar-miR-221	1	27	331
circRNA651	oar-let-7b	1	78	1620
circRNA53	oar-miR-1197-3p	1	61	3980
circRNA304	oar-miR-23b	1	262	7540
circRNA161	oar-miR-665-5p	1	1073	56600
circRNA8	oar-miR-541-5p	1	85	3450
ciRNA51	oar-miR-431	1	10	635
circRNA269	oar-let-7c	1	37	2060
circRNA403	oar-miR-487b-5p	1	32	1980
circRNA178	oar-miR-433-5p	1	16	1380
circRNA511	oar-miR-154b-5p	1	59	3000
circRNA648	oar-miR-654-5p	1	39.5	1810
circRNA691	oar-miR-539-3p	1	8	474
circRNA364	oar-miR-654-5p	1	140	6320
circRNA570	oar-miR-668-5p	1	121	6150
circRNA173	oar-miR-3959-3p	1	38	1350
circRNA481	oar-let-7i	1	44	2590
circRNA180	oar-miR-654-5p	1	30	2030
circRNA690	oar-miR-655-5p	1	4	284
circRNA259	oar-miR-376a-5p	1	46	1230
circRNA1	oar-miR-133	1	10	605
circRNA81	oar-miR-544-3p	1	19	1280
circRNA44	oar-miR-376b-3p	1	61	1930
circRNA441	oar-miR-194	1	21	626
circRNA367	oar-miR-150	1	93.5	3400
circRNA207	oar-miR-323a-5p	1	45	1600
circRNA682	oar-miR-665-3p	1	16	831
ciRNA1	oar-miR-329a-3p	1	4	354
circRNA317	oar-miR-329b-5p	1	223	11200
circRNA516	oar-miR-376d	1	14	976
circRNA781	oar-miR-379-5p	1	8	399
circRNA403	oar-miR-154b-3p	1	32	1980
circRNA261	oar-miR-22-3p	1	384	11400
circRNA31	oar-miR-127	1	102	1620
circRNA582	oar-miR-329b-5p	1	39	1480
circRNA752	oar-let-7d	1	21	926
circRNA446	oar-miR-3958-3p	1	38	1460
circRNA472	oar-miR-380-5p	1	35	1330
circRNA11	oar-miR-412-3p	1	11	576
circRNA496	oar-let-7d	1	90.5	3120
ciRNA59	oar-miR-329a-3p	1	5	361
circRNA273	oar-miR-655-5p	1	59	1890
circRNA720	oar-miR-654-5p	1	1018	57400
ciRNA13	oar-miR-125b	1	359	32500
circRNA173	oar-miR-136	1	38	1350
ciRNA51	oar-miR-362	1	10	635
circRNA745	oar-miR-25	1	34	2350
circRNA543	oar-miR-376d	1	8.5	744
circRNA642	oar-miR-323a-5p	1	22	950
circRNA776	oar-miR-544-5p	1	27	331
circRNA772	oar-let-7g	1	34	1290
circRNA105	oar-miR-133	1	22	896
circRNA595	oar-miR-106b	1	12	672
circRNA210	oar-miR-411b-3p	1	131	5360
circRNA118	oar-miR-17-5p	1	32	1790
circRNA328	oar-miR-10a	1	16	1060
circRNA354	oar-miR-181a	1	142	3660
circRNA746	oar-miR-125b	1	108	3350
circRNA464	oar-miR-299-3p	1	48	1080
ciRNA47	oar-miR-23a	1	2	156
circRNA252	oar-miR-194	1	612	11700
circRNA724	oar-miR-329b-5p	1	8	540
circRNA198	oar-miR-433-5p	1	52	2760
circRNA770	oar-miR-1185-5p	1	7	452

circRNA243	oar-miR-431	1	26	1480
circRNA352	oar-miR-1197-3p	1	45	2060
circRNA670	oar-miR-323a-3p	1	364	16900
circRNA408	oar-miR-493-5p	1	42	2710
circRNA553	oar-miR-495-5p	1	37	974
ciRNA77	oar-miR-494-3p	1	1	98
circRNA699	oar-miR-380-3p	1	100	4470
circRNA223	oar-miR-103	1	34	1820
circRNA463	oar-miR-125b	1	40	2200
circRNA600	oar-miR-329a-5p	1	34	1850
circRNA447	oar-miR-493-5p	1	48	1760
circRNA686	oar-miR-1185-3p	1	30	923
circRNA146	oar-miR-493-3p	1	175	7890
circRNA551	oar-miR-221	1	193	4060
circRNA382	oar-miR-494-5p	1	26	984
circRNA260	oar-miR-654-5p	1	42	1370
circRNA304	oar-miR-23a	1	262	7540
circRNA29	oar-miR-329a-5p	1	53	1680
circRNA701	oar-miR-654-5p	1	4	263
circRNA559	oar-miR-376a-5p	1	18	1220
circRNA451	oar-let-7g	1	34	2270
circRNA300	oar-miR-181a	1	142	5630
circRNA54	oar-miR-3956-5p	1	52	1810
circRNA597	oar-miR-1197-5p	1	59	2680
ciRNA48	oar-miR-544-3p	1	10	329
circRNA397	oar-miR-411a-3p	1	54	2570
circRNA685	oar-miR-3958-3p	1	172	6290
circRNA444	oar-let-7a	1	29	1310
ciRNA87	oar-miR-329a-3p	1	35.3	2480
circRNA735	oar-miR-27a	1	19	950
circRNA406	oar-miR-370-3p	1	148	5360
circRNA253	oar-miR-136	1	50	1750
circRNA265	oar-miR-487b-5p	1	15	215
circRNA720	oar-miR-200b	1	1018	57400
circRNA435	oar-miR-496-5p	1	20.2	602
circRNA507	oar-miR-30a-5p	1	166	4910
circRNA314	oar-miR-3957-3p	1	23	1150
circRNA456	oar-miR-376a-5p	1	12	700
circRNA589	oar-miR-370-5p	1	11	260
circRNA582	oar-miR-665-5p	1	39	1480
circRNA503	oar-miR-493-5p	1	28	1410
circRNA90	oar-miR-654-5p	1	239	12600
circRNA187	oar-miR-668-5p	1	12	572
circRNA439	oar-miR-3957-5p	1	146	3240
circRNA298	oar-miR-134-5p	1	28	774
circRNA217	oar-miR-1193-5p	1	93	4010
circRNA7	oar-let-7a	1	47	1830
circRNA97	oar-let-7c	1	22	1350
circRNA298	oar-miR-1193-3p	1	28	774
circRNA292	oar-miR-376e-3p	1	13	484
circRNA773	oar-miR-3957-5p	1	25	1110
circRNA319	oar-let-7b	1	42	1620
circRNA27	oar-miR-25	1	44	2280
circRNA550	oar-let-7a	1	59	1040
circRNA273	oar-let-7a	1	59	1890
circRNA528	oar-miR-410-5p	1	93	2490
circRNA709	oar-miR-199a-3p	1	129	2940
circRNA229	oar-miR-3958-5p	1	48	845
circRNA752	oar-let-7g	1	21	926
ciRNA62	oar-miR-654-3p	1	6	277
circRNA653	oar-miR-103	1	16	332
circRNA288	oar-miR-1197-3p	1	37	1330
circRNA609	oar-miR-133	1	49	1860
circRNA460	oar-miR-218a	1	249	6760
circRNA613	oar-miR-382-5p	1	21	735
circRNA381	oar-let-7a	1	29	1720
circRNA618	oar-miR-136	1	81	4230
circRNA169	oar-let-7a	1	24	1170
circRNA492	oar-miR-379-5p	1	157	7210
circRNA60	oar-miR-432	1	66	3680
circRNA714	oar-miR-495-3p	1	23	1190
circRNA647	oar-miR-544-3p	1	110	5290
circRNA11	oar-let-7f	1	11	576
circRNA247	oar-miR-654-5p	1	41	1110
circRNA341	oar-let-7b	1	107	4040
circRNA265	oar-miR-433-3p	1	15	215
circRNA294	oar-miR-133	1	158	8020
circRNA309	oar-miR-543-5p	1	4	249
circRNA41	oar-miR-27a	1	9	617
circRNA111	oar-miR-665-3p	1	181	4380
circRNA218	oar-miR-136	1	71	4670
circRNA645	oar-miR-191	1	52	2700
circRNA703	oar-miR-432	1	3	248
circRNA500	oar-miR-376e-5p	1	45	1680
circRNA22	oar-let-7f	1	255	5330
circRNA50	oar-miR-412-3p	1	41	968
circRNA651	oar-miR-181a	1	78	1620
circRNA252	oar-let-7d	1	612	11700
ciRNA95	oar-miR-3955-5p	1	4	383
circRNA659	oar-miR-154a-5p	1	125	6640
circRNA581	oar-miR-27a	1	14	835
circRNA686	oar-miR-181a	1	30	923
circRNA709	oar-miR-377-3p	1	129	2940
circRNA410	oar-miR-22-3p	1	160	4560
circRNA84	oar-miR-412-3p	1	5	316
circRNA637	oar-miR-493-3p	1	120	2400
circRNA291	oar-miR-494-5p	1	23	830
circRNA559	oar-miR-106a	1	18	1220

circRNA353	oar-miR-154b-3p	1	21	1130
circRNA669	oar-miR-323a-5p	1	178	6140
circRNA92	oar-miR-154b-5p	1	35	2090
circRNA319	oar-let-7g	1	42	1620
circRNA267	oar-let-7f	1	14	591
circRNA375	oar-miR-30a-3p	1	16	1010
circRNA29	oar-miR-485-5p	1	53	1680
circRNA30	oar-miR-29a	1	62	4220
circRNA349	oar-miR-10a	1	11	694
circRNA357	oar-miR-493-3p	1	9	644
circRNA764	oar-miR-487a-3p	1	159	6640
circRNA154	oar-miR-485-5p	1	27	1160
circRNA221	oar-miR-199a-3p	1	74	2240
circRNA189	oar-miR-107	1	3	343
circRNA405	oar-miR-29a	1	53	3730
circRNA283	oar-miR-154a-5p	1	219	14800
circRNA525	oar-miR-27a	1	62	2050
circRNA683	oar-miR-758-3p	1	7	686
circRNA30	oar-let-7i	1	62	4220
circRNA500	oar-miR-376b-5p	1	45	1680
circRNA221	oar-let-7a	1	74	2240
circRNA623	oar-miR-487b-3p	1	91	4670
circRNA321	oar-miR-10b	1	48	2450
circRNA231	oar-miR-3957-5p	1	59	1860
circRNA583	oar-miR-1197-5p	1	44	2100
circRNA54	oar-miR-411b-5p	1	52	1810
circRNA647	oar-let-7c	1	110	5290
circRNA658	oar-miR-106a	1	38	2500
circRNA356	oar-miR-143	1	152	6210
circRNA478	oar-miR-412-5p	1	167	6990
circRNA53	oar-miR-3957-5p	1	61	3980
circRNA601	oar-miR-136	1	349	19400
ciRNA17	oar-miR-376a-5p	1	5	545
circRNA225	oar-let-7f	1	29	1590
circRNA48	oar-miR-1197-3p	1	37	992
circRNA35	oar-miR-376b-5p	1	34	1030
circRNA322	oar-let-7g	1	130	4390
circRNA52	oar-miR-27a	1	14	313
circRNA175	oar-miR-655-5p	1	9	475
circRNA164	oar-miR-22-3p	1	107	5430
circRNA544	oar-miR-376e-3p	1	58.5	2700
circRNA103	oar-miR-411b-5p	1	147	2290
circRNA110	oar-miR-154a-5p	1	62	1800
circRNA228	oar-miR-654-5p	1	4.5	224
circRNA275	oar-miR-543-3p	1	165	2850
circRNA457	oar-miR-382-3p	1	146	7240
circRNA35	oar-miR-329b-3p	1	34	1030
circRNA365	oar-miR-668-5p	1	25	611
circRNA613	oar-miR-376a-5p	1	21	735
circRNA653	oar-miR-432	1	16	332
circRNA539	oar-let-7d	1	29	1360
circRNA771	oar-miR-380-3p	1	30	1530
circRNA42	oar-miR-21	1	193	3260
ciRNA98	oar-miR-380-3p	1	6	411
circRNA132	oar-miR-493-3p	1	19	1890
circRNA678	oar-miR-200b	1	24	1290
circRNA110	oar-miR-495-5p	1	62	1800
circRNA310	oar-miR-381-5p	1	150	3620
circRNA159	oar-miR-668-5p	1	32	2070
ciRNA69	oar-miR-541-5p	1	5	334
circRNA771	oar-miR-541-5p	1	30	1530
ciRNA8	oar-miR-30a-3p	1	2.5	137
circRNA131	oar-miR-329b-5p	1	24	1170
circRNA783	oar-miR-654-5p	1	339	16100
circRNA424	oar-miR-194	1	48	2000
circRNA472	oar-miR-487a-5p	1	35	1330
circRNA123	oar-miR-154b-3p	1	24.5	1030
circRNA784	oar-miR-329b-5p	1	1260	41800
circRNA686	oar-miR-1197-5p	1	30	923
circRNA446	oar-miR-654-5p	1	38	1460
circRNA735	oar-miR-379-5p	1	19	950
circRNA720	oar-miR-411b-3p	1	1018	57400
circRNA168	oar-miR-655-5p	1	45	1730
circRNA114	oar-miR-194	1	350	14700
circRNA539	oar-let-7a	1	29	1360
circRNA514	oar-miR-487a-5p	1	60	2890
circRNA207	oar-miR-431	1	45	1600
circRNA471	oar-miR-152	1	63	1820
circRNA553	oar-miR-29a	1	37	974
circRNA491	oar-miR-1197-5p	1	34	1470
ciRNA14	oar-miR-125b	1	235	22700
circRNA550	oar-miR-411b-3p	1	59	1040
circRNA68	oar-miR-379-5p	1	5	305
circRNA17	oar-miR-433-3p	1	62	2020
circRNA709	oar-miR-381-3p	1	129	2940
circRNA481	oar-miR-370-5p	1	44	2590
circRNA704	oar-miR-433-3p	1	49	2550
circRNA346	oar-miR-433-5p	1	15	570
circRNA447	oar-miR-485-3p	1	48	1760
circRNA628	oar-miR-379-5p	1	58	2150
circRNA239	oar-miR-493-3p	1	3	236
circRNA106	oar-miR-1185-3p	1	60	2410
circRNA99	oar-miR-376a-3p	1	17	1090
circRNA36	oar-miR-487a-3p	1	100	2650
ciRNA30	oar-miR-29b	1	2	84.4
circRNA493	oar-miR-379-5p	1	20.5	1330
circRNA633	oar-miR-3958-5p	1	28	1440
circRNA274	oar-miR-494-5p	1	66	2070

circRNA548	oar-miR-30a-3p	1	13	964
circRNA527	oar-miR-127	1	48	2150
circRNA589	oar-let-7g	1	11	260
circRNA478	oar-miR-323a-3p	1	167	6990
circRNA116	oar-miR-3957-5p	1	342	14200
circRNA61	oar-miR-25	1	17	681
circRNA310	oar-miR-493-5p	1	150	3620
circRNA128	oar-miR-103	1	118	4950
circRNA398	oar-miR-181a	1	135	4930
circRNA78	oar-miR-654-5p	1	47	1420
circRNA716	oar-miR-411b-5p	1	83.5	2960
circRNA598	oar-miR-412-3p	1	20	1270
circRNA591	oar-miR-323a-3p	1	21	853
circRNA739	oar-miR-493-3p	1	173	6440
ciRNA97	oar-miR-668-3p	1	1	85.7
circRNA438	oar-miR-370-3p	1	33	1310
circRNA687	oar-miR-411b-5p	1	25	925
circRNA766	oar-miR-432	1	86.2	2720
circRNA764	oar-miR-154b-3p	1	159	6640
circRNA606	oar-miR-29b	1	95	3890
ciRNA10	oar-let-7d	1	40	2950
ciRNA16	oar-miR-370-3p	1	23.5	1170
circRNA184	oar-miR-299-3p	1	42	2340
circRNA696	oar-miR-143	1	10	490
circRNA327	oar-miR-379-5p	1	27	1070
circRNA163	oar-miR-369-5p	1	28	1180
circRNA574	oar-miR-382-5p	1	59	4470
circRNA125	oar-miR-3957-5p	1	74	3930
circRNA379	oar-miR-30a-3p	1	155	6470
circRNA543	oar-miR-376e-3p	1	8.5	744
circRNA265	oar-miR-494-5p	1	15	215
circRNA520	oar-miR-655-5p	1	8	740
circRNA359	oar-miR-3957-5p	1	93	3890
circRNA36	oar-miR-218a	1	100	2650
circRNA713	oar-miR-654-5p	1	10	612
circRNA567	oar-miR-411b-3p	1	64.5	2650
circRNA769	oar-miR-136	1	20	1000
circRNA636	oar-miR-25	1	49	2080
circRNA388	oar-miR-376c-5p	1	21	1740
circRNA95	oar-miR-1185-5p	1	28	1150
circRNA627	oar-miR-544-3p	1	6	472
circRNA521	oar-let-7i	1	49	3400
circRNA641	oar-let-7d	1	14	853
circRNA521	oar-let-7a	1	49	3400
circRNA295	oar-miR-411b-5p	1	485	13200
circRNA360	oar-miR-493-3p	1	14	1030
circRNA511	oar-miR-200b	1	59	3000
circRNA180	oar-let-7i	1	30	2030
circRNA362	oar-miR-543-5p	1	249	13300
circRNA526	oar-miR-299-3p	1	40	1900
circRNA169	oar-let-7c	1	24	1170
circRNA489	oar-miR-134-3p	1	17	1190
circRNA119	oar-let-7g	1	3	171
circRNA125	oar-miR-376b-5p	1	74	3930
circRNA436	oar-miR-152	1	123	2820
circRNA637	oar-miR-200a	1	120	2400
circRNA154	oar-miR-25	1	27	1160
circRNA307	oar-miR-485-5p	1	341	21400
circRNA365	oar-miR-103	1	25	611
circRNA92	oar-miR-200c	1	35	2090
circRNA674	oar-miR-152	1	16	825
circRNA502	oar-miR-654-5p	1	17	975
circRNA359	oar-miR-143	1	93	3890
circRNA721	oar-miR-665-3p	1	174	9120
circRNA309	oar-miR-1197-3p	1	4	249
circRNA622	oar-miR-107	1	93	5000
circRNA115	oar-miR-665-3p	1	280	9430
circRNA447	oar-miR-487a-3p	1	48	1760
circRNA565	oar-miR-103	1	45	1450
circRNA771	oar-miR-329a-3p	1	30	1530
circRNA163	oar-miR-3958-3p	1	28	1180
circRNA99	oar-miR-668-3p	1	17	1090
circRNA372	oar-let-7d	1	30	1200
circRNA381	oar-miR-362	1	29	1720
circRNA74	oar-miR-127	1	16	1040
circRNA604	oar-miR-26a	1	70	3730
circRNA760	oar-miR-431	1	158	8730
circRNA145	oar-miR-411a-3p	1	79	3280
circRNA145	oar-miR-152	1	79	3280
circRNA416	oar-miR-376e-3p	1	28	1680
circRNA667	oar-miR-376a-5p	1	445	21400
circRNA265	oar-miR-494-3p	1	15	215
circRNA579	oar-miR-103	1	201	6420
circRNA457	oar-miR-106a	1	146	7240
ciRNA81	oar-miR-30a-5p	1	12	425
circRNA225	oar-let-7c	1	29	1590
circRNA740	oar-miR-654-5p	1	11	815
circRNA659	oar-miR-382-5p	1	125	6640
circRNA377	oar-miR-134-5p	1	124	3890
circRNA67	oar-miR-154b-3p	1	26	1430
circRNA570	oar-miR-134-3p	1	121	6150
circRNA659	oar-let-7i	1	125	6640
circRNA325	oar-miR-409-5p	1	39.5	1570
circRNA605	oar-miR-655-5p	1	63	526
circRNA560	oar-miR-200b	1	24	859
circRNA160	oar-miR-107	1	31	1750
circRNA421	oar-miR-362	1	20	1070
circRNA22	oar-miR-329a-5p	1	255	5330

circRNA654	oar-miR-1197-5p	1	20	1490
circRNA137	oar-miR-654-5p	1	24	849
circRNA92	oar-let-7g	1	35	2090
circRNA301	oar-miR-485-3p	1	111	6260
circRNA640	oar-let-7f	1	59	2230
ciRNA21	oar-miR-487a-3p	1	3	147
circRNA317	oar-let-7a	1	223	11200
circRNA604	oar-miR-377-5p	1	70	3730
circRNA407	oar-miR-181a	1	44	2870
circRNA14	oar-let-7g	1	99	3350
circRNA461	oar-miR-1197-5p	1	70	3260
ciRNA91	oar-miR-493-3p	1	1	109
circRNA709	oar-miR-25	1	129	2940
circRNA25	oar-miR-539-5p	1	27	1840
circRNA478	oar-miR-3955-3p	1	167	6990
circRNA225	oar-miR-493-3p	1	29	1590
circRNA664	oar-miR-107	1	56	2640
circRNA217	oar-miR-485-3p	1	93	4010
circRNA233	oar-miR-10a	1	90	5650
circRNA775	oar-miR-382-5p	1	17	410
circRNA391	oar-miR-191	1	19	358
circRNA576	oar-miR-369-5p	1	21	1320
circRNA22	oar-let-7a	1	255	5330
circRNA103	oar-miR-668-5p	1	147	2290
circRNA455	oar-miR-181a	1	20	994
circRNA229	oar-miR-485-5p	1	48	845
circRNA93	oar-miR-541-5p	1	291	8600
circRNA120	oar-miR-493-3p	1	20	965
circRNA374	oar-miR-485-5p	1	33	1050
circRNA746	oar-miR-154a-3p	1	108	3350
ciRNA77	oar-miR-107	1	1	98
circRNA72	oar-miR-136	1	35	1280
ciRNA37	oar-miR-3957-3p	1	22	666
circRNA427	oar-miR-380-5p	1	19	551
circRNA538	oar-miR-3955-3p	1	41	1830
circRNA539	oar-miR-370-3p	1	29	1360
circRNA491	oar-miR-543-5p	1	34	1470
circRNA86	oar-miR-3958-5p	1	19	956
circRNA87	oar-miR-485-5p	1	22	1330
circRNA651	oar-miR-665-5p	1	78	1620
circRNA768	oar-miR-194	1	43.7	1850
circRNA44	oar-let-7a	1	61	1930
circRNA381	oar-miR-495-5p	1	29	1720
circRNA22	oar-miR-487b-3p	1	255	5330
circRNA89	oar-miR-654-3p	1	24	2180
circRNA484	oar-miR-30a-3p	1	135	4150
circRNA387	oar-miR-494-5p	1	74	3810
circRNA560	oar-miR-409-5p	1	24	859
circRNA480	oar-miR-370-3p	1	9	338
circRNA507	oar-miR-99a	1	166	4910
circRNA610	oar-miR-329b-5p	1	83	2110
circRNA523	oar-miR-655-5p	1	69.5	3180
circRNA4	oar-miR-541-3p	1	8	593
circRNA268	oar-miR-154a-5p	1	6	339
circRNA400	oar-miR-412-3p	1	28	922
circRNA312	oar-miR-133	1	190	3850
circRNA568	oar-miR-23a	1	166	4830
ciRNA10	oar-miR-665-5p	1	40	2950
circRNA113	oar-miR-29a	1	52	3060
circRNA401	oar-miR-3955-5p	1	13	666
circRNA633	oar-miR-496-3p	1	28	1440
circRNA100	oar-miR-133	1	20	767
circRNA23	oar-miR-374a	1	70	5360
circRNA716	oar-let-7c	1	83.5	2960
circRNA411	oar-miR-539-5p	1	32.5	2460
circRNA321	oar-miR-494-3p	1	48	2450
circRNA597	oar-miR-299-5p	1	59	2680
circRNA759	oar-miR-329a-5p	1	46	2440
circRNA288	oar-miR-665-5p	1	37	1330
circRNA286	oar-miR-487a-3p	1	50	1870
ciRNA44	oar-miR-1193-3p	1	4	227
circRNA718	oar-miR-377-5p	1	4	334
circRNA488	oar-miR-29b	1	37	1330
circRNA304	oar-miR-494-5p	1	262	7540
circRNA716	oar-let-7f	1	83.5	2960
ciRNA58	oar-miR-409-3p	1	2.33	122
circRNA120	oar-miR-3956-5p	1	20	965
circRNA294	oar-miR-3957-5p	1	158	8020
circRNA686	oar-miR-654-5p	1	30	923
circRNA412	oar-miR-22-3p	1	28.2	1620
circRNA550	oar-miR-493-3p	1	59	1040
circRNA48	oar-miR-496-5p	1	37	992
circRNA528	oar-miR-412-3p	1	93	2490
circRNA639	oar-miR-411b-5p	1	71	3330
circRNA179	oar-miR-194	1	46	1780
circRNA623	oar-miR-485-3p	1	91	4670
circRNA757	oar-miR-362	1	190	5460
circRNA726	oar-miR-143	1	64	4520
circRNA34	oar-miR-665-5p	1	21	827
ciRNA26	oar-miR-487a-5p	1	7	715
circRNA117	oar-miR-3957-5p	1	5	521
circRNA4	oar-miR-25	1	8	593
circRNA22	oar-miR-150	1	255	5330
circRNA770	oar-miR-370-5p	1	7	452
circRNA126	oar-miR-3957-5p	1	19	1280
circRNA610	oar-miR-376c-5p	1	83	2110
circRNA50	oar-miR-655-5p	1	41	968
ciRNA20	oar-miR-3959-5p	1	11	319

ciRNA49	oar-miR-541-5p	1	6	449
circRNA579	oar-miR-3955-5p	1	201	6420
circRNA716	oar-miR-136	1	83.5	2960
circRNA24	oar-miR-668-3p	1	34	2370
circRNA174	oar-miR-379-5p	1	25	1290
circRNA599	oar-miR-376c-5p	1	6	298
circRNA500	oar-let-7c	1	45	1680
circRNA653	oar-miR-154a-3p	1	16	332
circRNA231	oar-miR-16b	1	59	1860
circRNA90	oar-miR-411a-3p	1	239	12600
circRNA39	oar-miR-376e-3p	1	11	513
circRNA464	oar-miR-134-3p	1	48	1080
circRNA427	oar-miR-106a	1	19	551
circRNA736	oar-miR-154b-5p	1	459	26700
circRNA248	oar-miR-103	1	6	370
circRNA175	oar-miR-376b-3p	1	9	475
circRNA737	oar-miR-154b-5p	1	37	1410
circRNA219	oar-miR-379-3p	1	8	439
circRNA22	oar-let-7c	1	255	5330
circRNA464	oar-miR-376e-3p	1	48	1080
ciRNA60	oar-miR-329b-3p	1	18	809
ciRNA67	oar-miR-1197-5p	1	6	537
circRNA311	oar-miR-133	1	32	2310
circRNA158	oar-miR-154a-3p	1	26	1560
circRNA34	oar-miR-362	1	21	827
circRNA249	oar-miR-199a-3p	1	357	11500
circRNA492	oar-miR-329b-5p	1	157	7210
circRNA226	oar-miR-1197-5p	1	13	1170
circRNA172	oar-miR-541-5p	1	21	847
circRNA740	oar-miR-22-3p	1	11	815
circRNA243	oar-miR-493-3p	1	26	1480
ciRNA26	oar-miR-19b	1	7	715
circRNA593	oar-miR-541-5p	1	37	1850
circRNA387	oar-miR-3955-5p	1	74	3810
circRNA705	oar-miR-485-3p	1	20	657
circRNA147	oar-miR-411b-5p	1	12	556
circRNA224	oar-miR-654-3p	1	37	1600
circRNA693	oar-miR-25	1	27	842
circRNA582	oar-miR-10b	1	39	1480
ciRNA10	oar-miR-3958-5p	1	40	2950
circRNA186	oar-miR-3956-5p	1	69	4270
circRNA765	oar-let-7f	1	15	760
circRNA365	oar-miR-654-5p	1	25	611
circRNA641	oar-miR-181a	1	14	853
circRNA475	oar-miR-199a-3p	1	16	858
circRNA151	oar-miR-370-3p	1	199	11100
circRNA419	oar-let-7g	1	12	777
circRNA544	oar-miR-382-5p	1	58.5	2700
circRNA104	oar-miR-411a-3p	1	14	856
circRNA363	oar-miR-154b-5p	1	40.5	3530
circRNA490	oar-miR-26a	1	240	5160
circRNA213	oar-miR-377-5p	1	97	6070
circRNA32	oar-miR-370-5p	1	28	1400
ciRNA77	oar-miR-200c	1	1	98
circRNA231	oar-miR-323a-5p	1	59	1860
ciRNA13	oar-miR-370-3p	1	359	32500
circRNA516	oar-miR-377-3p	1	14	976
circRNA353	oar-miR-329b-5p	1	21	1130
circRNA724	oar-miR-376a-3p	1	8	540
circRNA240	oar-miR-1197-3p	1	114	4230
circRNA243	oar-miR-199a-3p	1	26	1480
circRNA565	oar-miR-376d	1	45	1450
circRNA579	oar-miR-323a-5p	1	201	6420
circRNA241	oar-miR-412-5p	1	45	2440
circRNA753	oar-miR-181a	1	109	3710
circRNA497	oar-miR-382-5p	1	47.5	1540
circRNA438	oar-miR-299-3p	1	33	1310
circRNA536	oar-miR-668-5p	1	87	4870
circRNA580	oar-miR-154b-3p	1	19	784
circRNA370	oar-let-7g	1	39	1490
circRNA618	oar-miR-665-5p	1	81	4230
circRNA393	oar-miR-136	1	9	450
circRNA478	oar-miR-136	1	167	6990
circRNA243	oar-miR-541-3p	1	26	1480
circRNA353	oar-miR-539-3p	1	21	1130
circRNA18	oar-miR-412-3p	1	26	1100
circRNA36	oar-miR-487b-3p	1	100	2650
circRNA504	oar-let-7d	1	123	5780
circRNA692	oar-miR-200a	1	24	831
circRNA593	oar-miR-655-5p	1	37	1850
circRNA240	oar-miR-23a	1	114	4230
circRNA114	oar-miR-370-3p	1	350	14700
ciRNA33	oar-miR-143	1	3	309
circRNA256	oar-miR-487b-5p	1	58	4320
circRNA657	oar-miR-1197-5p	1	19	843
circRNA768	oar-miR-10b	1	43.7	1850
circRNA787	oar-miR-541-5p	1	83.5	5290
circRNA85	oar-miR-3957-5p	1	12	501
circRNA296	oar-miR-432	1	20	634
circRNA111	oar-miR-665-5p	1	181	4380
circRNA457	oar-miR-17-5p	1	146	7240
circRNA224	oar-miR-22-3p	1	37	1600
circRNA539	oar-let-7g	1	29	1360
circRNA476	oar-miR-377-5p	1	81	2660
circRNA544	oar-miR-103	1	58.5	2700
circRNA757	oar-miR-412-3p	1	190	5460
circRNA93	oar-let-7g	1	291	8600
circRNA99	oar-miR-21	1	17	1090

circRNA614	oar-miR-107	1	50	1590
circRNA581	oar-miR-665-5p	1	14	835
circRNA523	oar-miR-152	1	69.5	3180
circRNA35	oar-miR-3959-3p	1	34	1030
circRNA138	oar-miR-221	1	10	640
circRNA777	oar-miR-27a	1	10	409
circRNA721	oar-miR-376c-3p	1	174	9120
circRNA187	oar-miR-541-5p	1	12	572
circRNA411	oar-miR-127	1	32.5	2460
circRNA108	oar-miR-1193-5p	1	94	2680
circRNA48	oar-miR-134-3p	1	37	992
circRNA739	oar-miR-668-5p	1	173	6440
circRNA546	oar-miR-23a	1	20	1190
circRNA185	oar-miR-411b-5p	1	7	333
circRNA242	oar-miR-329b-5p	1	56	3340
circRNA582	oar-miR-544-3p	1	39	1480
circRNA692	oar-miR-136	1	24	831
circRNA651	oar-miR-3956-3p	1	78	1620
circRNA384	oar-let-7b	1	78	3290
circRNA736	oar-miR-107	1	459	26700
ciRNA59	oar-miR-377-3p	1	5	361
circRNA154	oar-let-7a	1	27	1160
circRNA31	oar-miR-654-5p	1	102	1620
ciRNA71	oar-miR-665-3p	1	16	596
circRNA245	oar-miR-3957-3p	1	26	1730
circRNA223	oar-miR-107	1	34	1820
circRNA356	oar-miR-655-5p	1	152	6210
circRNA407	oar-miR-370-3p	1	44	2870
circRNA250	oar-miR-1193-5p	1	14	743
ciRNA20	oar-miR-370-3p	1	11	319
ciRNA24	oar-miR-370-5p	1	2	218
circRNA173	oar-miR-29b	1	38	1350
circRNA727	oar-miR-21	1	82	4350
circRNA180	oar-miR-299-3p	1	30	2030
circRNA489	oar-miR-410-5p	1	17	1190
circRNA322	oar-miR-376d	1	130	4390
circRNA207	oar-miR-1185-5p	1	45	1600
circRNA279	oar-miR-541-3p	1	71	3110
circRNA565	oar-miR-329a-5p	1	45	1450
circRNA67	oar-miR-376b-3p	1	26	1430
circRNA631	oar-miR-329a-3p	1	379	9240
circRNA768	oar-miR-29a	1	43.7	1850
circRNA272	oar-miR-376a-5p	1	67	969
circRNA355	oar-miR-412-3p	1	342	13100
circRNA221	oar-let-7f	1	74	2240
circRNA468	oar-miR-541-3p	1	31	2170
ciRNA4	oar-miR-496-3p	1	2	215
circRNA662	oar-miR-3957-5p	1	63	1980
circRNA277	oar-miR-30a-3p	1	971	48700
circRNA221	oar-miR-544-5p	1	74	2240
circRNA217	oar-miR-154a-3p	1	93	4010
circRNA369	oar-miR-485-5p	1	33	1560
circRNA149	oar-miR-154b-5p	1	71	3740
circRNA222	oar-miR-3958-5p	1	58	2470
circRNA272	oar-miR-29b	1	67	969
circRNA578	oar-miR-107	1	8	334
circRNA638	oar-miR-495-5p	1	51	2390
circRNA206	oar-miR-410-5p	1	20	1050
circRNA157	oar-miR-485-5p	1	268	16300
ciRNA31	oar-miR-150	1	90.5	7640
circRNA266	oar-miR-433-3p	1	95	5370
circRNA743	oar-miR-496-5p	1	291	8460
circRNA749	oar-miR-199a-3p	1	72	3380
circRNA155	oar-miR-3955-3p	1	21	796
circRNA727	oar-miR-329b-5p	1	82	4350
circRNA173	oar-miR-3958-5p	1	38	1350
circRNA540	oar-miR-29a	1	87	3030
circRNA312	oar-miR-107	1	190	3850
circRNA571	oar-miR-485-3p	1	25	1300
circRNA495	oar-miR-133	1	134	5930
circRNA663	oar-miR-3958-5p	1	29	1380
circRNA312	oar-miR-3955-5p	1	190	3850
circRNA776	oar-miR-668-3p	1	27	331
circRNA344	oar-miR-154b-5p	1	19	583
circRNA313	oar-miR-143	1	235	11700
circRNA775	oar-let-7g	1	17	410
circRNA737	oar-miR-329b-5p	1	37	1410
circRNA564	oar-miR-370-3p	1	28	1490
circRNA29	oar-let-7g	1	53	1680
circRNA581	oar-miR-154a-3p	1	14	835
circRNA206	oar-miR-329b-5p	1	20	1050
circRNA584	oar-miR-154a-5p	1	318	12200
circRNA30	oar-miR-29b	1	62	4220
circRNA592	oar-miR-103	1	68	1670
circRNA509	oar-miR-106a	1	88	3130
ciRNA94	oar-miR-362	1	3	285
circRNA476	oar-miR-3956-3p	1	81	2660
circRNA230	oar-let-7i	1	12	602
ciRNA10	oar-let-7a	1	40	2950
circRNA575	oar-miR-1197-5p	1	76	3630
circRNA425	oar-miR-125b	1	36	1090
circRNA599	oar-miR-380-5p	1	6	298
circRNA509	oar-let-7g	1	88	3130
circRNA329	oar-miR-380-3p	1	61	5460
ciRNA53	oar-miR-665-5p	1	6	306
circRNA570	oar-miR-30c	1	121	6150
circRNA782	oar-miR-134-5p	1	17	828
circRNA748	oar-miR-30a-3p	1	22	1180

circRNA40	oar-miR-377-5p	1	22	1130
circRNA539	oar-let-7c	1	29	1360
circRNA406	oar-miR-411b-5p	1	148	5360
ciRNA2	oar-miR-495-5p	1	6	267
circRNA45	oar-let-7d	1	39	1110
circRNA306	oar-miR-495-5p	1	20	1120
circRNA530	oar-miR-200a	1	248	14000
circRNA654	oar-miR-410-5p	1	20	1490
circRNA739	oar-miR-412-5p	1	173	6440
circRNA680	oar-miR-381-5p	1	14	820
circRNA506	oar-miR-3959-3p	1	29	1530
ciRNA24	oar-miR-136	1	2	218
circRNA55	oar-miR-433-5p	1	23	1460
circRNA24	oar-let-7g	1	34	2370
circRNA147	oar-miR-1193-5p	1	12	556
ciRNA27	oar-miR-541-3p	1	25	2040
ciRNA71	oar-miR-1197-3p	1	16	596
circRNA568	oar-miR-30b	1	166	4830
circRNA28	oar-miR-10a	1	23	872
circRNA205	oar-miR-487b-5p	1	34	1150
circRNA18	oar-miR-323a-5p	1	26	1100
circRNA477	oar-miR-376c-3p	1	260	6440
circRNA504	oar-miR-379-5p	1	123	5780
circRNA307	oar-miR-543-5p	1	341	21400
circRNA647	oar-miR-150	1	110	5290
circRNA274	oar-miR-154a-5p	1	66	2070
circRNA651	oar-miR-370-3p	1	78	1620
circRNA744	oar-miR-154b-5p	1	56	1790
circRNA635	oar-miR-758-5p	1	59	2650
circRNA370	oar-miR-3958-5p	1	39	1490
circRNA482	oar-miR-329b-5p	1	275	8290
circRNA268	oar-miR-329a-5p	1	6	339
circRNA668	oar-miR-154b-5p	1	30	1850
circRNA478	oar-miR-370-3p	1	167	6990
circRNA95	oar-miR-376c-3p	1	28	1150
circRNA392	oar-miR-148a	1	36	1990
circRNA384	oar-miR-433-5p	1	78	3290
circRNA386	oar-miR-377-3p	1	102	2110
circRNA337	oar-miR-1193-3p	1	529	30300
circRNA608	oar-miR-495-5p	1	33	1990
circRNA128	oar-miR-665-3p	1	118	4950
circRNA642	oar-miR-654-5p	1	22	950
circRNA334	oar-miR-362	1	176	11100
ciRNA29	oar-miR-668-3p	1	1	85.3
circRNA72	oar-miR-26b	1	35	1280
circRNA472	oar-miR-154b-5p	1	35	1330
circRNA123	oar-miR-485-3p	1	24.5	1030
circRNA513	oar-miR-370-5p	1	14	1130
circRNA727	oar-miR-432	1	82	4350
circRNA319	oar-miR-154b-5p	1	42	1620
circRNA615	oar-miR-16b	1	41	2320
circRNA651	oar-miR-380-5p	1	78	1620
circRNA464	oar-miR-329b-5p	1	48	1080
circRNA681	oar-miR-493-3p	1	49	1570
circRNA597	oar-miR-758-3p	1	59	2680
circRNA765	oar-miR-26a	1	15	760
circRNA541	oar-miR-410-5p	1	58	4700
circRNA628	oar-miR-3957-5p	1	58	2150
circRNA344	oar-miR-30a-3p	1	19	583
circRNA366	oar-miR-191	1	6.5	463
circRNA632	oar-miR-329b-5p	1	26	1610
circRNA82	oar-miR-323c	1	81	5460
circRNA658	oar-miR-17-5p	1	38	2500
circRNA446	oar-miR-107	1	38	1460
circRNA378	oar-miR-329a-3p	1	42.5	1830
circRNA662	oar-let-7a	1	63	1980
circRNA507	oar-miR-30d	1	166	4910
circRNA604	oar-miR-539-3p	1	70	3730
circRNA242	oar-miR-544-5p	1	56	3340
circRNA195	oar-miR-21	1	53	1510
circRNA687	oar-let-7i	1	25	925
circRNA735	oar-miR-103	1	19	950
circRNA412	oar-miR-329a-3p	1	28.2	1620
circRNA38	oar-miR-758-5p	1	117	7230
circRNA590	oar-miR-379-5p	1	81	5580
circRNA427	oar-miR-19b	1	19	551
circRNA712	oar-let-7c	1	120	5180
circRNA551	oar-miR-200b	1	193	4060
circRNA738	oar-miR-181a	1	211	12700
circRNA49	oar-miR-494-3p	1	23	1490
circRNA267	oar-miR-380-3p	1	14	591
circRNA502	oar-miR-154a-5p	1	17	975
circRNA334	oar-miR-485-5p	1	176	11100
circRNA776	oar-miR-487a-5p	1	27	331
circRNA537	oar-miR-107	1	37	2640
circRNA681	oar-miR-494-5p	1	49	1570
circRNA544	oar-miR-493-5p	1	58.5	2700
circRNA117	oar-miR-668-5p	1	5	521
circRNA686	oar-miR-103	1	30	923
circRNA375	oar-miR-541-5p	1	16	1010
circRNA90	oar-miR-758-5p	1	239	12600
circRNA527	oar-miR-3957-3p	1	48	2150
circRNA416	oar-miR-299-5p	1	28	1680
circRNA382	oar-miR-154b-5p	1	26	984
circRNA373	oar-miR-654-3p	1	44	2350
circRNA96	oar-miR-493-3p	1	44	3060
circRNA102	oar-miR-3956-3p	1	13	681
circRNA647	oar-miR-410-5p	1	110	5290

circRNA575	oar-miR-539-3p	1	76	3630
circRNA180	oar-let-7c	1	30	2030
ciRNA35	oar-miR-376a-5p	1	4	157
circRNA685	oar-miR-668-5p	1	172	6290
circRNA325	oar-miR-379-5p	1	39.5	1570
circRNA776	oar-miR-495-5p	1	27	331
circRNA44	oar-miR-150	1	61	1930
circRNA406	oar-miR-376b-3p	1	148	5360
circRNA97	oar-miR-379-5p	1	22	1350
ciRNA44	oar-miR-200b	1	4	227
circRNA392	oar-miR-152	1	36	1990
circRNA236	oar-miR-544-3p	1	181	4960
circRNA82	oar-miR-382-3p	1	81	5460
circRNA276	oar-miR-136	1	8	332
circRNA30	oar-miR-487a-5p	1	62	4220
circRNA533	oar-miR-3955-3p	1	53	1980
circRNA278	oar-miR-181a	1	65	2960
circRNA782	oar-miR-374a	1	17	828
circRNA417	oar-miR-432	1	61	2420
circRNA471	oar-miR-3958-3p	1	63	1820
circRNA669	oar-miR-323a-3p	1	178	6140
circRNA721	oar-miR-21	1	174	9120
circRNA752	oar-miR-3956-3p	1	21	926
circRNA11	oar-let-7c	1	11	576
circRNA536	oar-miR-200c	1	87	4870
circRNA771	oar-miR-200c	1	30	1530
circRNA212	oar-miR-154a-5p	1	118	4620
circRNA580	oar-miR-539-3p	1	19	784
circRNA122	oar-miR-134-5p	1	177	5560
circRNA326	oar-miR-668-3p	1	7.5	387
circRNA702	oar-miR-409-3p	1	20	1460
circRNA389	oar-miR-1185-3p	1	20	1470
circRNA651	oar-miR-134-5p	1	78	1620
circRNA95	oar-miR-377-5p	1	28	1150
circRNA700	oar-miR-362	1	33	2440
circRNA111	oar-miR-431	1	181	4380
circRNA285	oar-miR-154b-5p	1	23	991
circRNA498	oar-let-7g	1	15	657
circRNA263	oar-miR-493-3p	1	17	1240
circRNA377	oar-miR-382-3p	1	124	3890
circRNA42	oar-miR-411b-3p	1	193	3260
circRNA772	oar-miR-493-3p	1	34	1290
circRNA303	oar-miR-380-3p	1	39.5	2770
circRNA638	oar-miR-154b-5p	1	51	2390
circRNA19	oar-miR-181a	1	22	1010
circRNA509	oar-miR-17-5p	1	88	3130
circRNA706	oar-miR-411a-3p	1	40	938
circRNA275	oar-miR-106a	1	165	2850
circRNA103	oar-let-7f	1	147	2290
circRNA100	oar-miR-26b	1	20	767
circRNA410	oar-miR-127	1	160	4560
circRNA376	oar-miR-668-3p	1	43	2030
circRNA260	oar-miR-29b	1	42	1370
ciRNA56	oar-miR-412-3p	1	15	1070
circRNA519	oar-miR-654-5p	1	18	1590
circRNA8	oar-let-7b	1	85	3450
circRNA486	oar-miR-329a-5p	1	13	615
circRNA407	oar-miR-665-3p	1	44	2870
circRNA761	oar-miR-376a-5p	1	11	767
circRNA281	oar-miR-380-5p	1	17	594
circRNA114	oar-miR-134-5p	1	350	14700
circRNA236	oar-miR-3957-5p	1	181	4960
circRNA604	oar-miR-26b	1	70	3730
circRNA198	oar-miR-495-3p	1	52	2760
circRNA672	oar-miR-376c-3p	1	62	4910
circRNA398	oar-miR-412-3p	1	135	4930
circRNA242	oar-miR-154b-5p	1	56	3340
circRNA352	oar-miR-27a	1	45	2060
circRNA125	oar-let-7g	1	74	3930
circRNA300	oar-miR-16b	1	142	5630
circRNA775	oar-let-7b	1	17	410
circRNA39	oar-let-7i	1	11	513
circRNA637	oar-miR-299-5p	1	120	2400
circRNA87	oar-miR-143	1	22	1330
circRNA175	oar-miR-26b	1	9	475
ciRNA71	oar-miR-380-5p	1	16	596
circRNA62	oar-miR-370-5p	1	146	7900
circRNA190	oar-miR-411b-5p	1	10	840
circRNA553	oar-miR-29b	1	37	974
circRNA167	oar-miR-154b-5p	1	14	900
circRNA179	oar-miR-432	1	46	1780
circRNA757	oar-miR-382-3p	1	190	5460
circRNA402	oar-miR-654-5p	1	29	1790
circRNA114	oar-miR-377-3p	1	350	14700
ciRNA50	oar-miR-370-3p	1	36	3220
circRNA398	oar-let-7b	1	135	4930
circRNA293	oar-miR-496-5p	1	9	553
ciRNA94	oar-miR-200c	1	3	285
circRNA706	oar-miR-380-5p	1	40	938
circRNA90	oar-miR-379-3p	1	239	12600
circRNA582	oar-miR-3957-3p	1	39	1480
circRNA323	oar-let-7f	1	8	555
circRNA115	oar-miR-329b-5p	1	280	9430
ciRNA21	oar-miR-485-3p	1	3	147
circRNA526	oar-miR-27a	1	40	1900
circRNA446	oar-miR-412-3p	1	38	1460
circRNA733	oar-miR-181a	1	22	1110
circRNA523	oar-miR-494-3p	1	69.5	3180

circRNA742	oar-miR-3956-3p	1	63	2210
circRNA759	oar-miR-218a	1	46	2440
circRNA181	oar-let-7c	1	28	916
circRNA73	oar-miR-379-5p	1	11	559
circRNA372	oar-miR-30a-5p	1	30	1200
circRNA46	oar-miR-127	1	22	1190
circRNA690	oar-miR-299-3p	1	4	284
circRNA579	oar-miR-133	1	201	6420
circRNA116	oar-miR-654-5p	1	342	14200
ciRNA46	oar-miR-379-5p	1	3	201
circRNA160	oar-miR-103	1	31	1750
circRNA442	oar-let-7b	1	32	1980
circRNA100	oar-miR-668-3p	1	20	767
circRNA662	oar-let-7f	1	63	1980
circRNA701	oar-miR-329a-5p	1	4	263
circRNA425	oar-let-7f	1	36	1090
circRNA473	oar-miR-377-3p	1	8	499
circRNA625	oar-miR-103	1	56	1680
circRNA384	oar-let-7c	1	78	3290
circRNA527	oar-miR-411a-3p	1	48	2150
circRNA460	oar-miR-1193-3p	1	249	6760
circRNA149	oar-miR-380-3p	1	71	3740
circRNA31	oar-miR-200a	1	102	1620
circRNA115	oar-miR-544-5p	1	280	9430
circRNA637	oar-miR-382-5p	1	120	2400
circRNA683	oar-let-7f	1	7	686
circRNA500	oar-miR-127	1	45	1680
circRNA463	oar-miR-150	1	40	2200
circRNA3	oar-miR-380-5p	1	99	4710
circRNA389	oar-miR-495-3p	1	20	1470
ciRNA67	oar-miR-323a-5p	1	6	537
circRNA173	oar-miR-1193-5p	1	38	1350
circRNA726	oar-miR-134-5p	1	64	4520
circRNA72	oar-miR-221	1	35	1280
circRNA503	oar-miR-154b-5p	1	28	1410
circRNA784	oar-miR-665-5p	1	1260	41800
circRNA590	oar-let-7f	1	81	5580
circRNA663	oar-miR-758-3p	1	29	1380
circRNA522	oar-miR-370-3p	1	39	1470
circRNA25	oar-miR-493-5p	1	27	1840
circRNA63	oar-miR-493-3p	1	49	2720
circRNA168	oar-miR-493-5p	1	45	1730
circRNA599	oar-miR-412-5p	1	6	298
circRNA784	oar-miR-1185-3p	1	1260	41800
ciRNA80	oar-miR-381-5p	1	3	265
circRNA258	oar-miR-431	1	6	514
circRNA581	oar-miR-16b	1	14	835
circRNA137	oar-miR-411b-5p	1	24	849
circRNA610	oar-miR-1193-3p	1	83	2110
circRNA628	oar-miR-758-3p	1	58	2150
circRNA305	oar-miR-103	1	52	2410
circRNA521	oar-miR-654-5p	1	49	3400
circRNA261	oar-miR-134-3p	1	384	11400
circRNA536	oar-miR-493-3p	1	87	4870
circRNA506	oar-miR-655-5p	1	29	1530
circRNA500	oar-miR-21	1	45	1680
circRNA395	oar-miR-23b	1	31	1450
circRNA27	oar-miR-30a-3p	1	44	2280
circRNA123	oar-miR-431	1	24.5	1030
circRNA319	oar-let-7c	1	42	1620
ciRNA21	oar-miR-370-5p	1	3	147
circRNA1	oar-miR-544-3p	1	10	605
circRNA707	oar-let-7i	1	71	2950
circRNA230	oar-let-7b	1	12	602
circRNA132	oar-miR-380-5p	1	19	1890
circRNA278	oar-miR-30c	1	65	2960
circRNA746	oar-miR-411b-3p	1	108	3350
circRNA416	oar-miR-665-5p	1	28	1680
circRNA408	oar-miR-495-5p	1	42	2710
circRNA16	oar-miR-134-3p	1	26	1330
circRNA285	oar-miR-323a-5p	1	23	991
circRNA785	oar-miR-654-5p	1	2	135
circRNA322	oar-let-7f	1	130	4390
circRNA744	oar-let-7i	1	56	1790
ciRNA41	oar-miR-654-5p	1	7	551
circRNA273	oar-miR-539-3p	1	59	1890
circRNA639	oar-miR-3957-5p	1	71	3330
circRNA623	oar-miR-544-3p	1	91	4670
circRNA338	oar-miR-103	1	45.5	2180
circRNA623	oar-miR-103	1	91	4670
circRNA41	oar-miR-665-5p	1	9	617
circRNA460	oar-miR-493-3p	1	249	6760
circRNA507	oar-miR-3959-3p	1	166	4910
ciRNA84	oar-miR-3955-3p	1	6	645
circRNA121	oar-miR-493-5p	1	633	14600
circRNA381	oar-let-7b	1	29	1720
circRNA520	oar-miR-99a	1	8	740
circRNA44	oar-miR-200b	1	61	1930
circRNA634	oar-miR-329b-5p	1	20	1240
circRNA643	oar-miR-3957-5p	1	40	1880
circRNA638	oar-miR-382-5p	1	51	2390
circRNA560	oar-miR-200c	1	24	859
circRNA500	oar-miR-654-5p	1	45	1680
circRNA103	oar-miR-758-3p	1	147	2290
circRNA12	oar-miR-370-3p	1	32	1620
circRNA42	oar-miR-3956-3p	1	193	3260
circRNA692	oar-miR-485-5p	1	24	831
circRNA422	oar-miR-494-3p	1	28	1210

circRNA695	oar-miR-362	1	7.5	502
circRNA215	oar-miR-487a-5p	1	11	948
circRNA444	oar-miR-218a	1	29	1310
circRNA265	oar-miR-412-5p	1	15	215
circRNA593	oar-let-7a	1	37	1850
circRNA345	oar-miR-654-5p	1	32	1330
circRNA543	oar-miR-107	1	8.5	744
ciRNA38	oar-miR-409-3p	1	51	3960
ciRNA9	oar-miR-3955-3p	1	3	173
circRNA457	oar-miR-106b	1	146	7240
ciRNA79	oar-miR-541-5p	1	35	2410
circRNA356	oar-let-7d	1	152	6210
circRNA552	oar-miR-654-5p	1	40	2810
circRNA674	oar-let-7i	1	16	825
circRNA393	oar-miR-376b-5p	1	9	450
circRNA358	oar-miR-323b	1	56	3900
circRNA746	oar-miR-10a	1	108	3350
circRNA93	oar-miR-30d	1	291	8600
circRNA507	oar-miR-30a-3p	1	166	4910
circRNA176	oar-miR-323b	1	24	1150
circRNA210	oar-miR-382-3p	1	131	5360
circRNA339	oar-miR-107	1	9.5	677
circRNA171	oar-miR-323a-5p	1	166	8060
circRNA692	oar-miR-758-5p	1	24	831
circRNA91	oar-miR-654-5p	1	38.7	2200
circRNA424	oar-miR-136	1	48	2000
circRNA771	oar-miR-376b-5p	1	30	1530
circRNA480	oar-miR-544-5p	1	9	338
circRNA532	oar-miR-541-5p	1	731	18000
circRNA35	oar-let-7a	1	34	1030
circRNA576	oar-miR-654-5p	1	21	1320
circRNA524	oar-miR-494-3p	1	16.5	1190
ciRNA32	oar-miR-485-5p	1	91.5	7530
ciRNA63	oar-miR-539-3p	1	14	1230
circRNA370	oar-miR-376c-3p	1	39	1490
circRNA741	oar-miR-21	1	89	2700
circRNA639	oar-miR-329b-5p	1	71	3330
circRNA615	oar-miR-433-3p	1	41	2320
circRNA772	oar-let-7d	1	34	1290
circRNA111	oar-miR-125b	1	181	4380
circRNA473	oar-miR-29a	1	8	499
circRNA184	oar-miR-1193-5p	1	42	2340
circRNA727	oar-miR-370-5p	1	82	4350
circRNA629	oar-miR-106b	1	57	1900
circRNA281	oar-let-7a	1	17	594
circRNA207	oar-let-7g	1	45	1600
circRNA114	oar-miR-541-5p	1	350	14700
circRNA399	oar-miR-539-5p	1	40	1760
circRNA409	oar-miR-412-3p	1	26	1130
circRNA381	oar-let-7c	1	29	1720
circRNA314	oar-let-7f	1	23	1150
ciRNA75	oar-miR-487a-3p	1	3	118
circRNA280	oar-miR-3957-5p	1	30	1530
circRNA599	oar-miR-376b-5p	1	6	298
circRNA35	oar-miR-377-3p	1	34	1030
circRNA44	oar-let-7b	1	61	1930
circRNA131	oar-miR-329a-5p	1	24	1170
circRNA781	oar-miR-1197-5p	1	8	399
circRNA473	oar-miR-133	1	8	499
circRNA409	oar-miR-154b-5p	1	26	1130
circRNA275	oar-miR-154a-5p	1	165	2850
circRNA324	oar-miR-3957-5p	1	59	1900
circRNA378	oar-miR-382-3p	1	42.5	1830
circRNA29	oar-let-7b	1	53	1680
ciRNA32	oar-miR-1193-5p	1	91.5	7530
circRNA784	oar-miR-125b	1	1260	41800
circRNA655	oar-miR-1185-5p	1	10	623
circRNA3	oar-miR-758-5p	1	99	4710
circRNA356	oar-let-7a	1	152	6210
circRNA207	oar-miR-136	1	45	1600
circRNA24	oar-miR-125b	1	34	2370
circRNA733	oar-miR-22-3p	1	22	1110
circRNA382	oar-let-7c	1	26	984
circRNA176	oar-miR-411b-5p	1	24	1150
circRNA219	oar-miR-329b-5p	1	8	439
circRNA711	oar-miR-654-3p	1	63	2820
circRNA526	oar-miR-665-3p	1	40	1900
circRNA653	oar-miR-154b-3p	1	16	332
circRNA57	oar-miR-3959-3p	1	138	7370
circRNA347	oar-miR-665-5p	1	44	2980
circRNA109	oar-miR-218a	1	42	2010
circRNA314	oar-miR-433-3p	1	23	1150
circRNA35	oar-miR-412-3p	1	34	1030
circRNA331	oar-miR-494-5p	1	32	2510
circRNA608	oar-miR-381-5p	1	33	1990
circRNA169	oar-let-7d	1	24	1170
circRNA631	oar-miR-154a-5p	1	379	9240
circRNA44	oar-miR-107	1	61	1930
circRNA121	oar-miR-541-3p	1	633	14600
circRNA623	oar-miR-539-3p	1	91	4670
circRNA10	oar-miR-107	1	10	483
ciRNA5	oar-miR-134-5p	1	6	305
circRNA48	oar-miR-3958-5p	1	37	992
circRNA259	oar-miR-543-3p	1	46	1230
circRNA215	oar-miR-107	1	11	948
circRNA593	oar-let-7g	1	37	1850
circRNA565	oar-miR-376a-3p	1	45	1450
circRNA180	oar-let-7f	1	30	2030

ciRNA35	oar-miR-22-3p	1	4	157
circRNA156	oar-miR-154b-5p	1	42	2890
ciRNA81	oar-miR-30c	1	12	425
circRNA287	oar-miR-379-5p	1	6	215
circRNA375	oar-miR-654-5p	1	16	1010
circRNA711	oar-miR-362	1	63	2820
circRNA71	oar-miR-299-5p	1	34	1970
circRNA409	oar-miR-654-5p	1	26	1130
circRNA366	oar-miR-150	1	6.5	463
circRNA282	oar-miR-496-5p	1	6	334
ciRNA53	oar-miR-323a-5p	1	6	306
circRNA645	oar-miR-665-5p	1	52	2700
circRNA601	oar-miR-19b	1	349	19400
circRNA409	oar-miR-194	1	26	1130
circRNA92	oar-miR-654-5p	1	35	2090
circRNA229	oar-miR-544-3p	1	48	845
circRNA708	oar-miR-3956-5p	1	141	7610
circRNA767	oar-miR-29a	1	45.2	3460
circRNA436	oar-miR-485-5p	1	123	2820
circRNA404	oar-miR-329b-3p	1	29	2710
circRNA390	oar-miR-432	1	50	5050
circRNA64	oar-miR-3958-5p	1	10	815
circRNA416	oar-miR-154b-5p	1	28	1680
circRNA346	oar-miR-3958-3p	1	15	570
circRNA611	oar-miR-377-5p	1	40	1830
ciRNA10	oar-let-7c	1	40	2950
circRNA63	oar-miR-1197-5p	1	49	2720
circRNA735	oar-miR-665-3p	1	19	950
circRNA48	oar-miR-376a-5p	1	37	992
ciRNA44	oar-miR-10b	1	4	227
circRNA283	oar-miR-409-5p	1	219	14800
circRNA716	oar-let-7b	1	83.5	2960
circRNA707	oar-miR-544-3p	1	71	2950
circRNA734	oar-miR-485-5p	1	37	1920
circRNA677	oar-miR-143	1	238	8090
circRNA265	oar-miR-30a-5p	1	15	215
circRNA739	oar-miR-22-3p	1	173	6440
circRNA85	oar-miR-409-5p	1	12	501
circRNA316	oar-miR-494-5p	1	19	1020
circRNA461	oar-miR-665-3p	1	70	3260
circRNA420	oar-let-7c	1	9	705
circRNA137	oar-miR-758-5p	1	24	849
circRNA693	oar-miR-3958-5p	1	27	842
circRNA132	oar-miR-1197-5p	1	19	1890
circRNA360	oar-let-7c	1	14	1030
circRNA92	oar-miR-200b	1	35	2090
circRNA462	oar-miR-1197-5p	1	43	1210
circRNA116	oar-let-7f	1	342	14200
circRNA686	oar-miR-376c-3p	1	30	923
circRNA696	oar-let-7g	1	10	490
circRNA287	oar-miR-411b-5p	1	6	215
circRNA194	oar-miR-218a	1	65	3740
ciRNA53	oar-miR-191	1	6	306
circRNA697	oar-miR-1193-3p	1	21	1080
circRNA447	oar-miR-194	1	48	1760
circRNA162	oar-miR-370-5p	1	8	320
circRNA163	oar-miR-758-3p	1	28	1180
circRNA184	oar-miR-409-3p	1	42	2340
circRNA212	oar-miR-433-5p	1	118	4620
circRNA752	oar-let-7a	1	21	926
circRNA687	oar-miR-1193-5p	1	25	925
circRNA406	oar-miR-154b-5p	1	148	5360
circRNA686	oar-miR-376b-3p	1	30	923
circRNA747	oar-miR-154a-5p	1	26	1800
circRNA550	oar-let-7i	1	59	1040
circRNA434	oar-miR-411a-3p	1	167	1280
circRNA787	oar-miR-544-3p	1	83.5	5290
circRNA221	oar-let-7c	1	74	2240
circRNA455	oar-let-7b	1	20	994
circRNA336	oar-miR-133	1	24	1370
circRNA455	oar-let-7a	1	20	994
circRNA424	oar-miR-26b	1	48	2000
circRNA50	oar-miR-654-3p	1	41	968
circRNA714	oar-miR-200c	1	23	1190
circRNA740	oar-miR-152	1	11	815
circRNA588	oar-miR-299-3p	1	20	1250
circRNA6	oar-miR-362	1	67	3600
circRNA205	oar-let-7i	1	34	1150
ciRNA32	oar-miR-323a-5p	1	91.5	7530
circRNA786	oar-miR-3958-5p	1	26	1520
circRNA271	oar-miR-543-5p	1	27	1890
circRNA386	oar-miR-3956-5p	1	102	2110
circRNA706	oar-miR-370-5p	1	40	938
circRNA325	oar-miR-181a	1	39.5	1570
circRNA761	oar-miR-22-3p	1	11	767
circRNA251	oar-miR-127	1	53	1050
circRNA39	oar-miR-376c-3p	1	11	513
circRNA173	oar-miR-29a	1	38	1350
circRNA387	oar-miR-154b-5p	1	74	3810
circRNA709	oar-miR-3957-5p	1	129	2940
circRNA422	oar-miR-665-3p	1	28	1210
circRNA132	oar-miR-376a-5p	1	19	1890
circRNA550	oar-miR-17-5p	1	59	1040
circRNA169	oar-miR-22-3p	1	24	1170
ciRNA43	oar-miR-199a-3p	1	1	91.4
circRNA53	oar-miR-493-3p	1	61	3980
ciRNA69	oar-miR-380-5p	1	5	334
circRNA371	oar-miR-25	1	27	1490

circRNA776	oar-miR-369-5p	1	27	331
circRNA639	oar-miR-654-3p	1	71	3330
circRNA529	oar-miR-23b	1	50	1940
circRNA298	oar-miR-494-3p	1	28	774
circRNA172	oar-miR-411b-5p	1	21	847
circRNA200	oar-miR-411a-5p	1	73	2220
circRNA357	oar-miR-654-5p	1	9	644
circRNA605	oar-miR-410-5p	1	63	526
circRNA599	oar-miR-3956-5p	1	6	298
circRNA646	oar-miR-3959-3p	1	19	876
circRNA623	oar-miR-382-5p	1	91	4670
circRNA592	oar-miR-3957-5p	1	68	1670
circRNA475	oar-miR-133	1	16	858
circRNA635	oar-miR-221	1	59	2650
circRNA507	oar-miR-299-5p	1	166	4910
circRNA267	oar-miR-369-3p	1	14	591
circRNA725	oar-miR-329a-5p	1	79	4390
circRNA753	oar-miR-370-3p	1	109	3710
circRNA461	oar-miR-411b-3p	1	70	3260
circRNA498	oar-miR-380-5p	1	15	657
circRNA350	oar-miR-381-3p	1	9	581
circRNA246	oar-miR-323a-5p	1	53	1940
circRNA546	oar-miR-23b	1	20	1190
circRNA221	oar-miR-431	1	74	2240
circRNA577	oar-miR-655-3p	1	60	3480
circRNA216	oar-miR-27a	1	32	925
circRNA304	oar-miR-154a-3p	1	262	7540
circRNA542	oar-miR-485-5p	1	16	715
circRNA184	oar-miR-381-5p	1	42	2340
circRNA567	oar-miR-23a	1	64.5	2650
circRNA386	oar-miR-433-3p	1	102	2110
circRNA194	oar-miR-433-3p	1	65	3740
circRNA248	oar-miR-485-5p	1	6	370
circRNA458	oar-miR-1193-3p	1	9	549
circRNA317	oar-let-7i	1	223	11200
circRNA684	oar-miR-412-5p	1	70	5310
circRNA477	oar-miR-654-5p	1	260	6440
circRNA354	oar-miR-381-3p	1	142	3660
circRNA639	oar-miR-329a-5p	1	71	3330
circRNA272	oar-miR-432	1	67	969
circRNA67	oar-miR-134-3p	1	26	1430
circRNA93	oar-miR-30a-5p	1	291	8600
circRNA326	oar-miR-181a	1	7.5	387
circRNA61	oar-miR-431	1	17	681
circRNA707	oar-let-7b	1	71	2950
circRNA475	oar-miR-412-3p	1	16	858
circRNA267	oar-miR-30a-3p	1	14	591
ciRNA77	oar-miR-200b	1	1	98
circRNA576	oar-miR-379-5p	1	21	1320
circRNA436	oar-miR-329b-3p	1	123	2820
circRNA631	oar-miR-376a-5p	1	379	9240
circRNA439	oar-miR-493-3p	1	146	3240
circRNA749	oar-miR-136	1	72	3380
circRNA80	oar-miR-544-3p	1	46	1940
circRNA759	oar-miR-194	1	46	2440
circRNA717	oar-miR-494-5p	1	61.5	3250
circRNA384	oar-let-7a	1	78	3290
circRNA34	oar-miR-1197-5p	1	21	827
ciRNA67	oar-miR-133	1	6	537
circRNA538	oar-miR-23a	1	41	1830
circRNA784	oar-miR-485-3p	1	1260	41800
circRNA492	oar-miR-758-3p	1	157	7210
ciRNA98	oar-miR-376c-3p	1	6	411
circRNA229	oar-miR-125b	1	48	845
circRNA422	oar-miR-544-5p	1	28	1210
circRNA298	oar-miR-654-5p	1	28	774
circRNA706	oar-miR-154a-5p	1	40	938
circRNA382	oar-miR-3959-3p	1	26	984
circRNA414	oar-miR-433-3p	1	10	461
circRNA155	oar-miR-495-5p	1	21	796
circRNA354	oar-miR-382-3p	1	142	3660
circRNA602	oar-miR-299-3p	1	197	11100
circRNA605	oar-miR-411b-5p	1	63	526
circRNA84	oar-miR-323b	1	5	316
circRNA516	oar-miR-154a-3p	1	14	976
circRNA384	oar-miR-10a	1	78	3290
circRNA680	oar-miR-411a-5p	1	14	820
ciRNA86	oar-miR-1197-5p	1	35.3	2550
circRNA558	oar-miR-3959-5p	1	77	4250
circRNA672	oar-miR-431	1	62	4910
circRNA123	oar-miR-487a-3p	1	24.5	1030
circRNA641	oar-miR-27a	1	14	853
circRNA589	oar-miR-495-3p	1	11	260
circRNA78	oar-let-7i	1	47	1420
circRNA446	oar-miR-1197-3p	1	38	1460
circRNA170	oar-miR-410-5p	1	74	1320
circRNA17	oar-miR-409-5p	1	62	2020
circRNA513	oar-miR-3955-3p	1	14	1130
circRNA143	oar-miR-3955-5p	1	59	4880
circRNA458	oar-miR-136	1	9	549
circRNA623	oar-miR-410-5p	1	91	4670
circRNA640	oar-miR-21	1	59	2230
circRNA725	oar-miR-665-5p	1	79	4390
circRNA628	oar-miR-376e-3p	1	58	2150
circRNA252	oar-miR-376a-5p	1	612	11700
circRNA238	oar-miR-1197-5p	1	93	4020
circRNA20	oar-miR-134-5p	1	86	2300
ciRNA73	oar-miR-544-5p	1	8	675

circRNA294	oar-miR-496-3p	1	158	8020
circRNA20	oar-miR-221	1	86	2300
circRNA344	oar-miR-758-3p	1	19	583
circRNA475	oar-miR-379-5p	1	16	858
circRNA147	oar-miR-496-3p	1	12	556
ciRNA1	oar-miR-329b-3p	1	4	354
circRNA199	oar-miR-3956-3p	1	14	653
circRNA75	oar-miR-496-5p	1	45	1530
circRNA476	oar-miR-655-5p	1	81	2660
circRNA550	oar-let-7f	1	59	1040
circRNA409	oar-miR-323a-5p	1	26	1130
circRNA516	oar-miR-376a-3p	1	14	976
circRNA310	oar-miR-99a	1	150	3620
ciRNA59	oar-miR-329b-3p	1	5	361
circRNA82	oar-miR-485-5p	1	81	5460
circRNA681	oar-miR-377-5p	1	49	1570
circRNA498	oar-let-7a	1	15	657
circRNA317	oar-let-7c	1	223	11200
circRNA737	oar-miR-362	1	37	1410
circRNA55	oar-miR-409-5p	1	23	1460
circRNA54	oar-miR-758-5p	1	52	1810
circRNA660	oar-miR-412-3p	1	5	481
circRNA95	oar-miR-329a-3p	1	28	1150
circRNA80	oar-miR-543-5p	1	46	1940
circRNA686	oar-miR-544-3p	1	30	923
circRNA425	oar-miR-487a-5p	1	36	1090
circRNA472	oar-miR-485-5p	1	35	1330
circRNA625	oar-miR-16b	1	56	1680
circRNA430	oar-miR-654-5p	1	319	2550
circRNA588	oar-miR-412-3p	1	20	1250
circRNA331	oar-miR-3958-5p	1	32	2510
circRNA310	oar-miR-154b-5p	1	150	3620
circRNA571	oar-miR-362	1	25	1300
circRNA723	oar-miR-493-3p	1	21	1160
circRNA551	oar-miR-200c	1	193	4060
circRNA7	oar-miR-495-3p	1	47	1830
circRNA11	oar-let-7d	1	11	576
circRNA605	oar-miR-221	1	63	526
circRNA709	oar-miR-410-5p	1	129	2940
circRNA521	oar-miR-411a-5p	1	49	3400
ciRNA14	oar-miR-127	1	235	22700
circRNA58	oar-miR-382-5p	1	24	1080
circRNA502	oar-miR-381-5p	1	17	975
circRNA383	oar-miR-495-3p	1	11	479
circRNA195	oar-miR-665-5p	1	53	1510
circRNA231	oar-miR-3957-3p	1	59	1860
circRNA221	oar-miR-374a	1	74	2240
circRNA6	oar-miR-758-5p	1	67	3600
circRNA788	oar-miR-30a-3p	1	400	16300
circRNA346	oar-miR-3955-5p	1	15	570
circRNA47	oar-miR-30a-3p	1	122	5840
circRNA744	oar-miR-26b	1	56	1790
circRNA44	oar-miR-376a-3p	1	61	1930
circRNA103	oar-miR-329a-5p	1	147	2290
circRNA101	oar-miR-134-5p	1	4	119
circRNA10	oar-miR-152	1	10	483
circRNA528	oar-miR-493-3p	1	93	2490
circRNA299	oar-miR-1185-5p	1	8	590
circRNA491	oar-miR-376a-5p	1	34	1470
circRNA165	oar-miR-668-3p	1	14	948
circRNA181	oar-miR-433-5p	1	28	916
circRNA578	oar-miR-3955-5p	1	8	334
ciRNA94	oar-miR-329b-5p	1	3	285
circRNA302	oar-miR-21	1	158	3480
circRNA643	oar-miR-134-3p	1	40	1880
circRNA616	oar-miR-329a-5p	1	84	5180
circRNA209	oar-miR-665-5p	1	2	97.7
circRNA200	oar-miR-1193-3p	1	73	2220
circRNA293	oar-miR-544-3p	1	9	553
circRNA269	oar-miR-134-5p	1	37	2060
circRNA599	oar-miR-3958-5p	1	6	298
circRNA504	oar-miR-199a-3p	1	123	5780
circRNA171	oar-miR-125b	1	166	8060
ciRNA36	oar-let-7i	1	17	1010
circRNA559	oar-miR-493-3p	1	18	1220
circRNA354	oar-miR-127	1	142	3660
circRNA391	oar-let-7a	1	19	358
ciRNA44	oar-miR-200c	1	4	227
circRNA278	oar-miR-665-5p	1	65	2960
circRNA372	oar-let-7c	1	30	1200
circRNA108	oar-miR-432	1	94	2680
circRNA687	oar-miR-485-5p	1	25	925
circRNA346	oar-miR-22-3p	1	15	570
circRNA322	oar-miR-376a-3p	1	130	4390
circRNA48	oar-miR-30a-3p	1	37	992
circRNA598	oar-miR-543-5p	1	20	1270
circRNA487	oar-miR-134-3p	1	33	1440
circRNA78	oar-miR-154b-5p	1	47	1420
circRNA88	oar-miR-668-5p	1	29	2190
circRNA720	oar-miR-495-5p	1	1018	57400
circRNA258	oar-miR-485-5p	1	6	514
circRNA641	oar-miR-379-5p	1	14	853
circRNA539	oar-miR-27a	1	29	1360
circRNA770	oar-miR-1197-5p	1	7	452
circRNA713	oar-miR-485-5p	1	10	612
circRNA591	oar-miR-3957-5p	1	21	853
circRNA365	oar-miR-369-5p	1	25	611
circRNA27	oar-miR-329b-5p	1	44	2280

circRNA597	oar-miR-143	1	59	2680
circRNA425	oar-let-7d	1	36	1090
circRNA268	oar-miR-376c-3p	1	6	339
circRNA28	oar-miR-487a-3p	1	23	872
circRNA316	oar-miR-3957-5p	1	19	1020
circRNA324	oar-miR-495-5p	1	59	1900
circRNA760	oar-miR-376a-5p	1	158	8730
circRNA317	oar-let-7b	1	223	11200
circRNA281	oar-miR-495-3p	1	17	594
circRNA384	oar-let-7i	1	78	3290
circRNA475	oar-miR-154b-5p	1	16	858
circRNA692	oar-miR-154b-5p	1	24	831
circRNA499	oar-miR-299-3p	1	15	581
circRNA538	oar-miR-329b-5p	1	41	1830
circRNA200	oar-miR-199a-3p	1	73	2220
circRNA770	oar-miR-654-3p	1	7	452
circRNA39	oar-miR-10a	1	11	513
circRNA476	oar-miR-323a-5p	1	81	2660
circRNA141	oar-miR-376a-5p	1	60	3370
circRNA313	oar-miR-495-5p	1	235	11700
circRNA402	oar-miR-412-5p	1	29	1790
ciRNA62	oar-miR-134-3p	1	6	277
circRNA70	oar-miR-654-5p	1	48	2430
circRNA48	oar-miR-148a	1	37	992
circRNA589	oar-miR-380-5p	1	11	260
circRNA578	oar-miR-543-3p	1	8	334
circRNA77	oar-miR-495-3p	1	418	15500
circRNA460	oar-miR-377-3p	1	249	6760
circRNA99	oar-miR-376b-3p	1	17	1090
circRNA691	oar-miR-496-3p	1	8	474
circRNA569	oar-miR-543-5p	1	175	6360
circRNA727	oar-miR-543-5p	1	82	4350
circRNA20	oar-miR-544-5p	1	86	2300
circRNA433	oar-miR-125b	1	213	1440
circRNA584	oar-miR-410-5p	1	318	12200
circRNA217	oar-miR-665-3p	1	93	4010
ciRNA10	oar-let-7b	1	40	2950
circRNA724	oar-miR-409-3p	1	8	540
circRNA204	oar-miR-1193-3p	1	26	1840
circRNA149	oar-let-7i	1	71	3740
circRNA199	oar-miR-433-5p	1	14	653
circRNA122	oar-miR-431	1	177	5560
ciRNA34	oar-miR-1197-3p	1	3	327
circRNA271	oar-miR-654-5p	1	27	1890
circRNA361	oar-miR-3958-5p	1	5	304
circRNA165	oar-miR-150	1	14	948
ciRNA12	oar-miR-376e-3p	1	7	488
circRNA371	oar-miR-30a-3p	1	27	1490
circRNA605	oar-let-7i	1	63	526
circRNA433	oar-miR-323a-3p	1	213	1440
circRNA108	oar-miR-299-5p	1	94	2680
circRNA467	oar-miR-148a	1	28	1050
circRNA41	oar-miR-103	1	9	617
circRNA706	oar-miR-654-5p	1	40	938
circRNA515	oar-miR-376a-3p	1	17	663
circRNA69	oar-miR-485-5p	1	6	525
circRNA303	oar-miR-125b	1	39.5	2770
ciRNA15	oar-miR-431	1	23.5	1170
circRNA441	oar-miR-221	1	21	626
circRNA402	oar-miR-323a-5p	1	29	1790
circRNA726	oar-miR-26b	1	64	4520
circRNA345	oar-miR-136	1	32	1330
circRNA539	oar-let-7f	1	29	1360
circRNA651	oar-miR-758-5p	1	78	1620
circRNA773	oar-let-7g	1	25	1110
circRNA683	oar-miR-3958-5p	1	7	686
circRNA752	oar-miR-154b-3p	1	21	926
circRNA474	oar-miR-21	1	427	25200
circRNA122	oar-miR-485-3p	1	177	5560
circRNA85	oar-miR-665-5p	1	12	501
circRNA347	oar-miR-376c-3p	1	44	2980
circRNA680	oar-miR-30a-3p	1	14	820
circRNA356	oar-miR-3956-3p	1	152	6210
circRNA257	oar-miR-655-5p	1	118	7890
circRNA24	oar-let-7f	1	34	2370
circRNA568	oar-miR-30c	1	166	4830
circRNA513	oar-miR-103	1	14	1130
circRNA505	oar-miR-495-5p	1	6	366
circRNA212	oar-miR-409-3p	1	118	4620
circRNA166	oar-miR-495-3p	1	171	4920
circRNA724	oar-miR-3957-5p	1	8	540
circRNA384	oar-miR-544-5p	1	78	3290
circRNA54	oar-miR-412-3p	1	52	1810
circRNA39	oar-miR-376a-3p	1	11	513
circRNA185	oar-miR-485-5p	1	7	333
circRNA625	oar-miR-376b-5p	1	56	1680
ciRNA10	oar-let-7f	1	40	2950
circRNA584	oar-miR-494-5p	1	318	12200
circRNA674	oar-miR-1193-5p	1	16	825
circRNA527	oar-miR-30b	1	48	2150
circRNA289	oar-miR-544-3p	1	19	965
circRNA678	oar-miR-221	1	24	1290
circRNA183	oar-miR-410-5p	1	189	9040
circRNA370	oar-let-7i	1	39	1490
circRNA477	oar-miR-539-5p	1	260	6440
circRNA249	oar-miR-133	1	357	11500
circRNA505	oar-miR-194	1	6	366
circRNA89	oar-miR-10b	1	24	2180

circRNA29	oar-let-7a	1	53	1680
circRNA555	oar-miR-411a-5p	1	36	1680
circRNA722	oar-miR-544-3p	1	33	1630
circRNA551	oar-miR-411a-5p	1	193	4060
circRNA181	oar-miR-493-3p	1	28	916
circRNA459	oar-miR-380-5p	1	31	1450
circRNA674	oar-let-7b	1	16	825
circRNA776	oar-miR-376a-5p	1	27	331
circRNA75	oar-miR-412-3p	1	45	1530
circRNA243	oar-miR-107	1	26	1480
circRNA479	oar-miR-3955-3p	1	94	4200
circRNA597	oar-miR-410-3p	1	59	2680
circRNA685	oar-miR-376a-3p	1	172	6290
circRNA119	oar-let-7d	1	3	171
circRNA200	oar-miR-541-3p	1	73	2220
circRNA118	oar-miR-329b-5p	1	32	1790
circRNA662	oar-let-7g	1	63	1980
circRNA772	oar-let-7c	1	34	1290
circRNA425	oar-miR-376e-5p	1	36	1090
circRNA732	oar-miR-143	1	16	538
circRNA231	oar-let-7i	1	59	1860
circRNA479	oar-miR-323c	1	94	4200
circRNA715	oar-miR-495-5p	1	64	2040
circRNA50	oar-miR-21	1	41	968
circRNA72	oar-miR-26a	1	35	1280
circRNA455	oar-miR-412-3p	1	20	994
circRNA507	oar-miR-1193-3p	1	166	4910
circRNA744	oar-miR-199a-3p	1	56	1790
circRNA8	oar-miR-127	1	85	3450
circRNA502	oar-miR-323a-5p	1	17	975
circRNA728	oar-miR-329a-5p	1	3	182
circRNA341	oar-miR-125b	1	107	4040
circRNA45	oar-miR-1193-5p	1	39	1110
circRNA649	oar-miR-10b	1	5.5	363
circRNA730	oar-miR-379-5p	1	46	2910
circRNA431	oar-miR-758-3p	1	512	2590
circRNA222	oar-miR-493-5p	1	58	2470
circRNA640	oar-let-7g	1	59	2230
circRNA209	oar-miR-299-3p	1	2	97.7
ciRNA48	oar-miR-431	1	10	329
circRNA18	oar-miR-3957-5p	1	26	1100
circRNA453	oar-miR-544-3p	1	13	374
circRNA768	oar-miR-432	1	43.7	1850
circRNA610	oar-miR-376b-5p	1	83	2110
circRNA322	oar-miR-541-5p	1	130	4390
circRNA701	oar-miR-329b-5p	1	4	263
circRNA585	oar-miR-1197-5p	1	24	1130
circRNA630	oar-miR-376a-5p	1	35	2060
circRNA126	oar-miR-377-5p	1	19	1280
circRNA105	oar-miR-654-5p	1	22	896
circRNA478	oar-miR-376b-5p	1	167	6990
ciRNA19	oar-miR-362	1	3	236
circRNA279	oar-let-7a	1	71	3110
circRNA81	oar-miR-3958-5p	1	19	1280
circRNA702	oar-miR-668-3p	1	20	1460
circRNA26	oar-miR-17-5p	1	3	157
circRNA299	oar-miR-1197-3p	1	8	590
circRNA712	oar-miR-433-3p	1	120	5180
circRNA547	oar-miR-655-5p	1	15	1100
circRNA679	oar-miR-544-3p	1	45	2240
circRNA329	oar-miR-3956-3p	1	61	5460
circRNA172	oar-miR-27a	1	21	847
circRNA734	oar-miR-376a-5p	1	37	1920
circRNA757	oar-miR-154b-5p	1	190	5460
circRNA459	oar-miR-30a-5p	1	31	1450
circRNA18	oar-miR-30a-5p	1	26	1100
circRNA416	oar-miR-376c-3p	1	28	1680
circRNA251	oar-miR-655-5p	1	53	1050
circRNA314	oar-miR-380-5p	1	23	1150
circRNA341	oar-let-7a	1	107	4040
ciRNA40	oar-miR-379-5p	1	86	8180
circRNA657	oar-miR-103	1	19	843
circRNA638	oar-miR-665-5p	1	51	2390
circRNA499	oar-miR-154b-5p	1	15	581
circRNA39	oar-let-7a	1	11	513
circRNA157	oar-miR-382-5p	1	268	16300
circRNA604	oar-miR-323b	1	70	3730
circRNA683	oar-let-7g	1	7	686
ciRNA3	oar-miR-3957-5p	1	6	543
circRNA200	oar-miR-329a-5p	1	73	2220
circRNA557	oar-miR-495-5p	1	203	9800
circRNA690	oar-miR-154a-5p	1	4	284
circRNA724	oar-miR-376b-3p	1	8	540
ciRNA36	oar-let-7b	1	17	1010
circRNA337	oar-miR-30b	1	529	30300
circRNA556	oar-miR-150	1	51	3800
circRNA464	oar-miR-376c-3p	1	48	1080
circRNA707	oar-let-7a	1	71	2950
ciRNA64	oar-miR-410-5p	1	7	702
circRNA590	oar-let-7c	1	81	5580
circRNA687	oar-miR-134-3p	1	25	925
circRNA128	oar-miR-409-3p	1	118	4950
circRNA694	oar-miR-136	1	109	3640
circRNA713	oar-miR-654-3p	1	10	612
circRNA758	oar-miR-412-3p	1	69	2410
circRNA207	oar-miR-299-5p	1	45	1600
circRNA523	oar-miR-154a-5p	1	69.5	3180
circRNA720	oar-let-7a	1	1018	57400

ciRNA98	oar-miR-411b-3p	1	6	411
circRNA276	oar-let-7i	1	8	332
circRNA616	oar-miR-376b-5p	1	84	5180
circRNA258	oar-miR-655-5p	1	6	514
circRNA349	oar-miR-541-3p	1	11	694
circRNA462	oar-miR-431	1	43	1210
circRNA236	oar-miR-376c-5p	1	181	4960
ciRNA46	oar-miR-433-5p	1	3	201
circRNA369	oar-miR-412-5p	1	33	1560
circRNA417	oar-miR-382-5p	1	61	2420
ciRNA84	oar-miR-541-5p	1	6	645
circRNA672	oar-miR-30a-3p	1	62	4910
circRNA10	oar-miR-103	1	10	483
circRNA692	oar-miR-3955-3p	1	24	831
circRNA183	oar-miR-133	1	189	9040
circRNA477	oar-miR-23a	1	260	6440
circRNA442	oar-miR-487b-3p	1	32	1980
circRNA103	oar-let-7g	1	147	2290
circRNA330	oar-miR-133	1	47	2500
circRNA503	oar-miR-496-3p	1	28	1410
circRNA188	oar-miR-329b-5p	1	35	1300
circRNA17	oar-miR-412-3p	1	62	2020
circRNA490	oar-miR-544-3p	1	240	5160
circRNA588	oar-miR-411b-5p	1	20	1250
circRNA331	oar-miR-543-5p	1	32	2510
circRNA639	oar-miR-412-3p	1	71	3330
ciRNA45	oar-let-7b	1	4	312
circRNA341	oar-miR-3957-5p	1	107	4040
circRNA481	oar-let-7c	1	44	2590
circRNA651	oar-miR-143	1	78	1620
circRNA344	oar-miR-103	1	19	583
circRNA545	oar-miR-30a-3p	1	174	10400
circRNA415	oar-miR-3958-3p	1	49	1620
circRNA1	oar-miR-10b	1	10	605
circRNA523	oar-miR-329a-5p	1	69.5	3180
circRNA610	oar-miR-376e-5p	1	83	2110
circRNA215	oar-miR-431	1	11	948
circRNA387	oar-miR-665-5p	1	74	3810
circRNA44	oar-miR-376d	1	61	1930
circRNA135	oar-miR-668-3p	1	68	3890
circRNA753	oar-miR-543-5p	1	109	3710
circRNA44	oar-miR-200c	1	61	1930
circRNA627	oar-miR-323a-5p	1	6	472
circRNA241	oar-miR-370-3p	1	45	2440
circRNA461	oar-miR-668-3p	1	70	3260
circRNA459	oar-miR-30c	1	31	1450
circRNA498	oar-let-7f	1	15	657
circRNA509	oar-let-7i	1	88	3130
circRNA88	oar-miR-3955-5p	1	29	2190
circRNA416	oar-miR-194	1	28	1680
circRNA212	oar-miR-154b-5p	1	118	4620
circRNA570	oar-miR-30b	1	121	6150
circRNA175	oar-miR-26a	1	9	475
circRNA272	oar-miR-379-3p	1	67	969
circRNA563	oar-miR-493-3p	1	106	2740
circRNA304	oar-miR-539-3p	1	262	7540
circRNA80	oar-miR-329a-5p	1	46	1940
circRNA259	oar-miR-495-5p	1	46	1230
circRNA262	oar-miR-26b	1	24	975
circRNA402	oar-miR-133	1	29	1790
circRNA561	oar-miR-1185-5p	1	59	2780
circRNA756	oar-miR-154b-5p	1	68	2660
circRNA374	oar-miR-382-5p	1	33	1050
circRNA224	oar-miR-410-5p	1	37	1600
circRNA712	oar-let-7b	1	120	5180
ciRNA5	oar-miR-127	1	6	305
circRNA212	oar-miR-323a-5p	1	118	4620
circRNA649	oar-miR-323a-5p	1	5.5	363
circRNA50	oar-miR-1185-3p	1	41	968
circRNA711	oar-miR-379-5p	1	63	2820
circRNA269	oar-miR-1197-3p	1	37	2060
ciRNA81	oar-miR-25	1	12	425
circRNA418	oar-miR-23a	1	32	1810
circRNA753	oar-miR-1197-3p	1	109	3710
ciRNA5	oar-miR-1197-5p	1	6	305
circRNA123	oar-miR-1185-3p	1	24.5	1030
circRNA35	oar-miR-487b-3p	1	34	1030
circRNA565	oar-let-7g	1	45	1450
circRNA102	oar-miR-3956-5p	1	13	681
circRNA524	oar-miR-154a-5p	1	16.5	1190
circRNA663	oar-miR-323a-5p	1	29	1380
circRNA446	oar-miR-154b-5p	1	38	1460
circRNA108	oar-miR-29b	1	94	2680
circRNA477	oar-miR-433-3p	1	260	6440
circRNA775	oar-let-7a	1	17	410
circRNA183	oar-miR-543-3p	1	189	9040
circRNA580	oar-miR-154a-3p	1	19	784
circRNA481	oar-miR-1193-3p	1	44	2590

Fig.S1

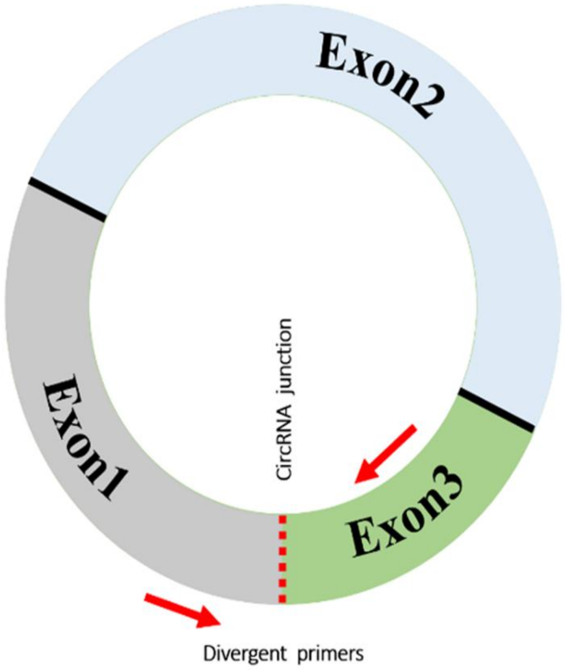

Fig.S1. Divergent primers for detecting circRNAs.
